# Copper-Catalyzed Asymmetric Cyclizative Sulfinamidation: Forging Indole-Based Stereogenic Sulfur(IV) Centers and Atropisomeric Chirality

**DOI:** 10.1021/acscentsci.5c00909

**Published:** 2025-08-01

**Authors:** Xiaowu Fang, Fengrui Xiang, Yue Zhao, Zhuangzhi Shi

**Affiliations:** † State Key Laboratory of Coordination Chemistry, Chemistry and Biomedicine Innovation Center (ChemBIC), School of Chemistry and Chemical Engineering, 12581 Nanjing University, Nanjing 210093, China; ‡ School of Chemistry and Materials Science, Nanjing Normal University, Nanjing 210023, China; § School of Chemistry and Chemical Engineering, Henan Normal University, Xinxiang 453007, China

## Abstract

The structural prominence of indole-based sulfur-containing compounds in pharmacologically relevant substances stems from their versatile biofunctional capabilities. Despite their significance, the stereogenic elements embedded in these structures have frequently been overlooked in drug discovery endeavors primarily due to the absence of efficient synthetic methodologies. Here, we introduce a groundbreaking strategy for the enantioselective synthesis of indole-based sulfinamides via a copper-catalyzed asymmetric nucleophilic cyclization and sulfinamidation reaction. Utilizing *ortho*-alkynylanilines and sulfinylamines, this method achieves a broad spectrum of sulfinamides with complete atom economy, establishing a new paradigm in synthetic efficiency. Our approach not only facilitates the formation of S-chirogenic sulfinamides but also concurrently constructs products featuring both stereogenic sulfur and atropisomeric chirality. Comprehensive mechanistic investigations, complemented by density functional theory (DFT) calculations, provide deep insights into the reaction mechanism, particularly in elucidating the S-stereogenic and atropisomeric control during the cyclization and sulfinamidation processes.

## Graphical abstract

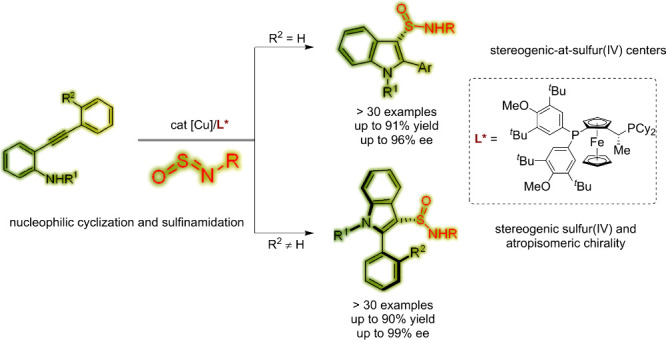

## Introduction

Atropisomeric (hetero)­biaryls constitute a privileged class of compounds with broad applications across medicinal chemistry, catalysis, and materials science.^1,2^ This significance has spurred the development of numerous advanced synthetic approaches.^3–17^ Particularly noteworthy are atropisomeric indole derivatives, which hold considerable importance due to their prevalence in natural alkaloids, bioactive molecules, chiral ligands, and organocatalysts.^18–22^ Consequently, the catalytic asymmetric synthesis of axially chiral indoles, especially those incorporating oxygen and nitrogen functional groups, has become a focal point of research (Figure 1A).^23–32^ A significant milestone in this field was achieved in 2010 when Kitagawa reported the palladium-catalyzed asymmetric cyclization of *ortho*-alkynylanilines, despite modest enantioselectivity.^33^ This strategy has since proven to be remarkably effective and versatile, enabling the construction of atropisomeric indoles through catalysis by both precious metals^34,35^ and organocatalysts.^36^ In parallel, indole-based sulfur-containing compounds have attracted considerable attention owing to their therapeutic and agrochemical potential.^37–44^ Nonetheless, the stereogenic elements are frequently overlooked in drug discovery initiatives.^45,46^ Therefore, the development of a general methodology to incorporate the backbone and central chirality into these indole frameworks holds significant promise for expanding the chemical space and enhancing the prospects of discovering novel drug leads with improved pharmacological properties.

**Figure 1:**
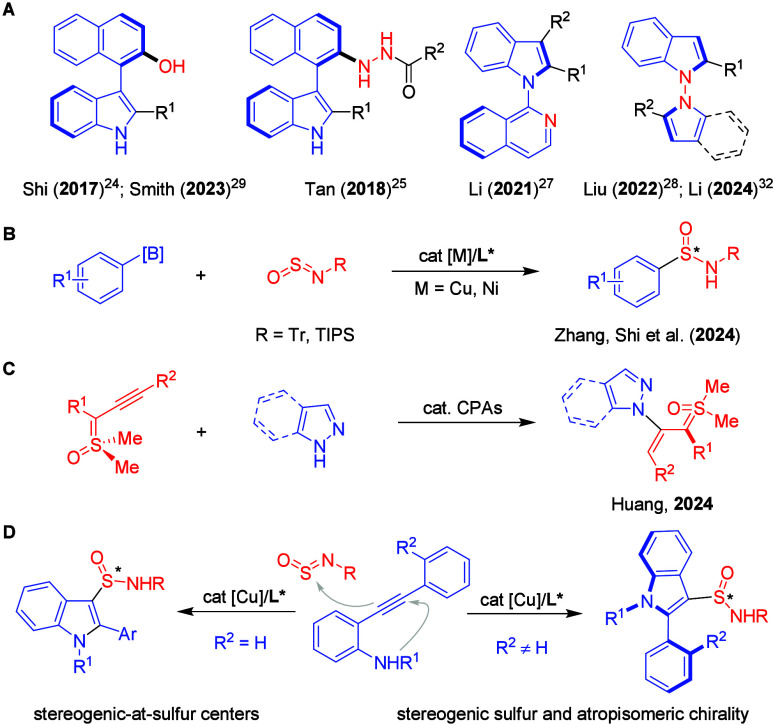
(A) Catalytic asymmetric synthesis of atropisomeric indoles. (B) Catalytic asymmetric synthesis of S-chirogenic sulfinamides by aryl-addition to sulfinylamines. (C) Catalytic asymmetric synthesis of atropisomerically chiral sulfoxonium ylides. (D) Copper-catalyzed asymmetric cyclizative sulfinamidation for indole-based stereogenic sulfur­(IV) centers and atropisomeric chirality.

Sulfur’s distinctive capacity to generate a wide array of chiral configurations, enabled by its varied oxidation states including S­(IV) and S­(VI) stereogenic centers, markedly increases the structural variety and intricacy of prospective pharmaceutical compounds.^47–62^ Among them, sulfinamides,^63–66^ particularly those featuring stereogenic sulfur­(IV) centers,^67–71^ have gained prominence as pivotal intermediates in synthetic chemistry due to their unique equilibrium between stability and reactivity. Substantial advancements have been made in their synthetic methodologies. Chiral organocatalysts, such as 4-arylpyridine *N*-oxides, and quinine derivatives, have been successfully employed in the nucleophilic substitution of sulfinates with amines.^72,73^ Additionally, these compounds can be synthesized using anionic stereogenic cobalt complexes, which involve an enantiopure sulfinimidoyl iodide intermediate.^74^ Notably, recent innovations have introduced asymmetric metal-catalyzed additions of boron compounds to sulfinylamines as a novel strategy for the preparation of aryl sulfinamides (Figure 1B).^75,76^ Moreover, aryl halides have been effectively added to sulfinylamines under reductive conditions, enabled by Earth-abundant metals.^77,78^ In contrast, the development of axially chiral organosulfur compounds has progressed at a notably slower pace. In 2024, Huang and colleagues achieved a significant breakthrough by successfully synthesizing atropisomerically chiral sulfoxonium ylides using chiral phosphoric acids (Figure 1C).^79^ Inspired by these advancements, we aimed to design and synthesize indole-based sulfinamides that incorporate both stereogenic sulfur centers and atropisomeric chirality.

Herein, we report a copper-catalyzed protocol that achieves both high enantioselectivity and diastereoselectivity in the nucleophilic cyclization of *ortho*-alkynylanilines, subsequently enabling sulfinamidation with sulfinylamines (Figure 1D). Although nucleophilic cyclization of *ortho*-alkynylanilines has been previously documented by copper catalysis,^80–82^ the development of enantioselective transformation has remained elusive. Our innovative methodology not only enables the efficient formation of S-chirogenic indole-based sulfinamides but also facilitates the construction of products that concurrently possess stereogenic sulfur­(IV) centers and atropisomeric chirality. Given the profound synthetic significance of these two pivotal stereochemical elements, their simultaneous integration into a single indole framework opens up unprecedented avenues for the assembly of structurally complex molecules with high precision and diversity.

## Results and Discussion

The feasibility of the reaction between *ortho*-alkynylaniline **1a** and sulfinylamine **I** was explored (Table 1). Initially, we developed a reaction system using 10 mol % of CuBr and 12 mol % of (*R*, *R*)-BINAP (**L1**), along with 1.2 equiv of K_2_CO_3_, in THF at 40 °C for 12 h under N_2_ atmosphere (entry 1). This approach, however, yielded the target product **1b** in only trace amounts. Switching the ligand to (*R*, *R*)-Ph-BPE (**L2**) resulted in a modest improvement, with a 10% yield of **1b** and 17% ee (entry 2). The use of (*S*)-MeO-BIPHEP (**L3**) slightly increased the yield to 16%, but the enantioselectivity decreased (entry 3). Utilizing Josiphos **L4** improved the enantioselectivity to 26% ee (entry 4). Notably, when Josiphos **L5**, featuring a bulky aryl group on the phosphorus atom, was employed, the enantioselectivity increased dramatically to 92% ee, although the yield remained low (entry 5). Further modifications to this ligand motif, such as using Josiphos **L6**, significantly reduced the enantioselectivity (entry 6). Experiments with alternative carbonates, such as Li_2_CO_3_, resulted in low conversion (entry 7), but the use of LiO^*t*^Bu as the base increased the enantioselectivity to 94% (entry 8). Increasing the amount of base to 3.0 equiv improved the yield of **1b** to 31% (entry 9). Extending the reaction time to 40 h further increased the yield to 49%, while maintaining high enantioselectivity (entry 10). The choice of solvent was critical for achieving high enantioselectivity. Notably, using 1,3-dioxolane (extra dry, stored with molecular sieves) as the solvent produced **1b** with an outstanding 95% ee and 89% yield (entry 11). Furthermore, an X-ray crystallographic analysis was performed on product **1b**, unequivocally confirming its *S* absolute configuration. Experiments with other copper sources, such as CuI, CuCl, and CuBr_2_, were less successful (entries 12–14). Reducing the catalyst loading to 5 mol % still maintained good reactivity, affording **1b** in 82% yield and 88% ee (entry 15). Finally, control reactions highlighted the essential role of the copper salt, and omitting it led to no reaction occurring (entry 16).

**Table 1:**
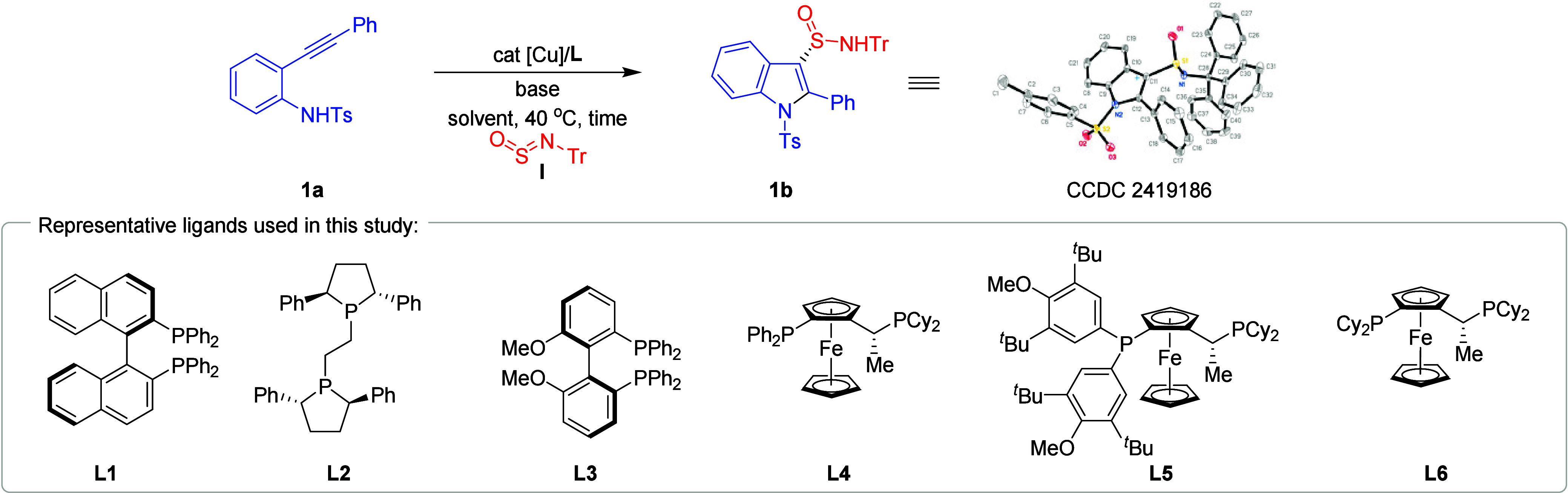
Optimization of the Reaction Conditions^a^

entry	[Cu] (mol %)	ligand (mol %)	base (equiv)	solvent	time (h)	yield of **1b** (%)^b^	ee of **1b** (%)^c^
1	CuBr (10)	**L1** (12)	K_2_CO_3_ (1.2)	THF	12	<5	-
2	CuBr (10)	**L2** (12)	K_2_CO_3_ (1.2)	THF	12	10	17
3	CuBr (10)	**L3** (12)	K_2_CO_3_ (1.2)	THF	12	16	–7
4	CuBr (10)	**L4** (12)	K_2_CO_3_ (1.2)	THF	12	12	26
5	CuBr (10)	**L5** (12)	K_2_CO_3_ (1.2)	THF	12	6	92
6	CuBr (10)	**L6** (12)	K_2_CO_3_ (1.2)	THF	12	6	72
7	CuBr (10)	**L5** (12)	Li_2_CO_3_ (1.2)	THF	12	trace	-
8	CuBr (10)	**L5** (12)	LiO^*t*^Bu (1.2)	THF	12	10	94
9	CuBr (10)	**L5** (12)	LiO^*t*^Bu (3.0)	THF	12	31	92
10	CuBr (10)	**L5** (12)	LiO^*t*^Bu (3.0)	THF	40	49	92
**11**	**CuBr (10)**	**L**5 (12)	**LiO** ^***t***^ **Bu (3.0)**	**1,3-dioxolane**	**40**	**89 (84)** ^d^	**95**
12	CuI (10)	**L5** (12)	LiO^*t*^Bu (3.0)	1,3-dioxolane	40	24	95
13	CuCl (10)	**L5** (12)	LiO^*t*^Bu (3.0)	1,3-dioxolane	40	86	84
14	CuBr_2_ (10)	**L5** (12)	LiO^*t*^Bu (3.0)	1,3-dioxolane	40	65	67
15	CuBr (5)	**L5** (6)	LiO^*t*^Bu (3.0)	1,3-dioxolane	40	82	88
16	-	**L5** (12)	LiO^*t*^Bu (3.0)	1,3-dioxolane	40	0	-

a Reaction conditions: Cu salt (5–10 mol %), ligand (6–12 mol %), **1a** (1.0 equiv), sulfinylamine **I** (1.0–3.0 equiv), base (1.2–3.0 equiv) in anhydrous solvent (0.1 M) at 40 °C under N_2_ atmosphere.

b Yield by ^1^H NMR with mesitylene as internal standard.

c Enantiomeric excess (ee) by chiral HPLC.

d Isolated yield.

With the reaction conditions set, we undertook a series of experiments to explore the scope of *ortho*-alkynylanilines with sulfinylamine **I** (Table 2). We began our exploration by examining the reactivity of arylethynyl anilines substituted with methyl (**2a**), ethyl (**3a**), isopropyl (**4a**), *tert*-butyl (**5a**), and phenyl (**6a**) groups. These reactions produced the desired products **2b**–**6b** effectively. Anilines containing methoxy (**7a**, **8a**) and NMe_2_ (**9a**) also proceeded efficiently, yielding the target compounds **7b**–**9b** with notably high enantioselectivities. Notably, a broad range of derivatives featuring halide-containing substituents, including fluorine (**10a**, **11a**), chlorine (**12a**), bromine (**13a**), and trifluoromethyl (**14a**), were well-tolerated with sulfinylamine **I**, producing the corresponding products **10b**–**14b** with excellent enantioselectivities. Additionally, the use of trimethylsilyl-containing substrate **15a** did not hinder the formation of the desired C–S bond, resulting in product **15b**. In addition to aromatic motifs, heteroaryl-substituted ethynyl anilines, such as benzofuran (**16a**) and 1-methylindole (**17a**), were successfully subjected to the system, yielding products **16b** and **17b** with outstanding results. The cyclization of anilines containing enyne motifs, exemplified by **18a**, facilitated the formation of desired product **18b**. Furthermore, alkyl-substituted alkyne **19a** produced the desired product **19b** with slightly reduced enantioselectivity (84% ee). Furthermore, a detailed examination of indoles with various substituents at the C2 position revealed significant insights. Methyl, phenethyl, isopropyl, and cyclopropyl groups all led to a notable decrease in enantioselectivity (5–66% ee), highlighting the critical influence of steric effects on the enantioselectivity of this transformation (see Table S1 in Supporting Information). We delved further into the investigation of functional groups attached to the benzene nuclei of the synthesized indoles. Compounds adorned with electron-neutral and electron-donating substituents, exemplified by methyl (**20b**, **21b**) and methoxy (**22b**, **23b**), were generated with exemplary enantioselectivities. The compatibility of the benzene core in indoles bearing halide substituents, such as fluorine (**24b**, **25b**), chlorine (**26b**), and bromine (**27a**, **28b**), was confirmed under the reaction conditions. We also evaluated the reaction tolerance of trifluoromethyl (**29b**) and ester (**30b**) groups within the indole backbone. Interestingly, the naphthalen-2-amine-derived substrate **31a** exhibited exceptional reactivity, resulting in the desired benzo­[f]­indole **31b** with a yield of 73% and an enantioselectivity of 95% ee. However, 2-(phenylethynyl)­phenol and 2-(phenylethynyl)­benzenethiol failed to undergo the desired transformations to form benzofuran- and benzothiophene-based sulfinamides at the current reaction conditions (see Table S1 in Supporting Information).

**Table 2:**
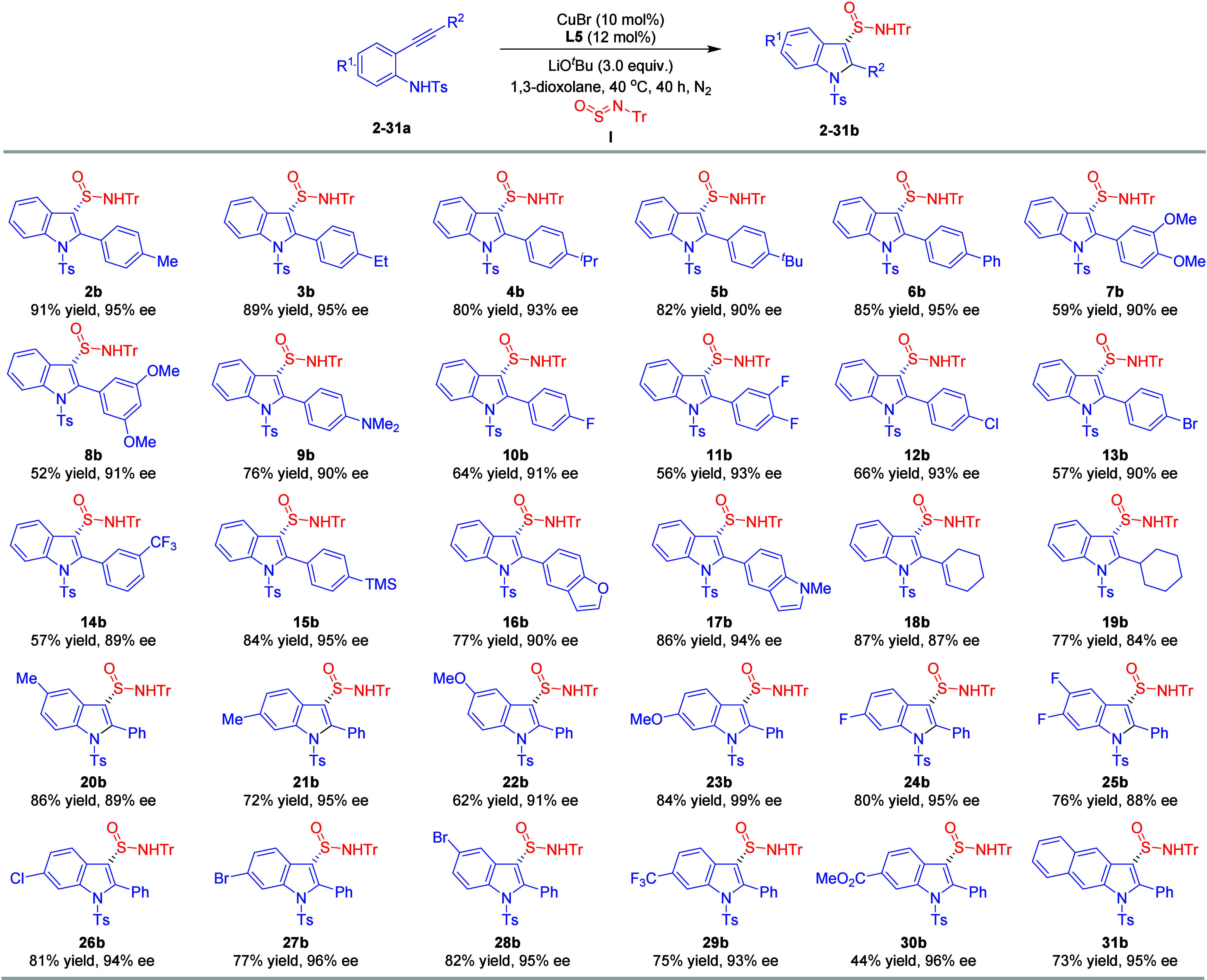
Substrate Scope of *ortho*-Alkynylanilines for Construction of S-Stereogenic Center^a^

a Reaction conditions: CuBr (10 mol %), **L5** (12 mol %), **2–31a** (0.1 mmol), sulfinylamine **I** (0.3 mmol), LiO^*t*^Bu (0.3 mmol) in 1,3-dioxolane (0.1 M) at 40 °C for 40 h under N_2_ atmosphere, isolated yield. Ee value by chiral HPLC.

To systematically evaluate the substrate scope for the simultaneous construction of S­(IV)-stereogenic centers and axial chirality, we explored the reaction of diverse *ortho*-alkynylanilines with sulfinylamine **I** under optimized conditions (Table 3). The reaction accommodates a wide range of substituents across the aniline core and arylacetylene unit, delivering products with consistently high enantio- and diastereoselectivity. The benchmark substrate **32a**, bearing an *o*-tolylethynyl group, afforded product **32b** with exceptional enantioselectivity (99% ee) and high diastereoselectivity (12/1 dr). Electron-neutral and electron-donating substituents on the aniline ring, including methyl (**33a**–**35a**) and methoxy (**36a**, **37a**), proved highly effective, delivering the corresponding products **33b–37b** with outstanding enantiocontrol and excellent diastereoselectivity. Halogenated substrates—encompassing fluorine (**38a**, **39a**), chlorine (**40a**, **41a**), and bromine (**42a**)—exhibited robust reactivity, furnishing **38b–42b** in high yields with maintained stereoselectivity. This highlights the tolerance of the reaction to both electron-withdrawing and sterically unencumbered halogens. We next investigated the influence of substituents on the arylacetylene moiety. Substrates featuring additional methyl (**43a**, **44a**), phenyl (**45a**), methoxy (**46a**), trifluoromethoxy (**47a**), fluorine (**48a**), or chlorine (**49a**) groups all underwent a smooth conversion to products **43b**–**49b** with exceptional stereoselectivity. Notably, the 2-fluorophenyl-substituted substrate **50a** provided product **50b** with moderate diastereoselectivity (1.5/1 dr), although both diastereomers retained high enantiopurity. Intriguingly, other halogens such as chlorine (**51a**), bromine (**52a**, **53a**), and iodine (**54a**) restored excellent diastereocontrol, suggesting a nuanced steric influence on selectivity. The reaction scope was extended successfully to naphthalene-based substrates. Both 1-ethynylnaphthalene (**55a–57a**) and 2-ethynylnaphthalene (**58a**, **59a**) derivatives were efficiently transformed into products **55b**–**59b** with high yields and stereoselectivity. Even the sterically congested 2-(phenanthren-9-ylethynyl)­aniline (**60a**) proved viable, yielding **60b** with good efficiency (48% yield, 97% ee, 12/1 dr). A striking example was observed with naphthalen-2-amine-derived **61a**, which exhibited remarkable reactivity to furnish atropisomeric benzo­[f]­indole **61b** in 84% yield with 86% ee. This result underscores the versatility of the method in accessing complex heterocyclic architectures with high stereocontrol.

**Table 3:**
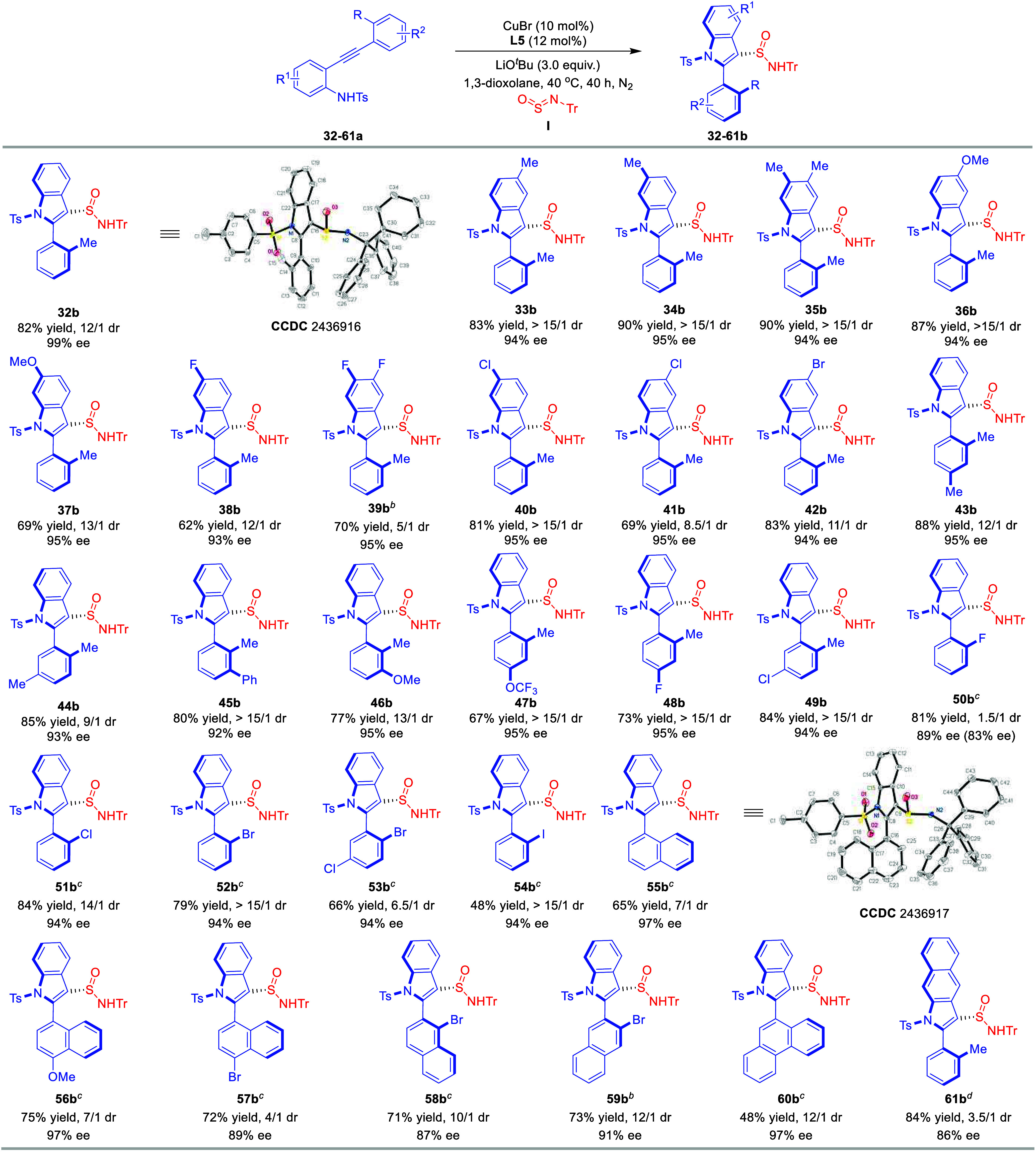
Substrate Scope of *ortho*-Alkynylanilines for Construction of Indole-Based S-Stereogenic Center and Axial Chirality^a^

a Reaction conditions: CuBr (10 mol %), **L5** (12 mol %), **32**-**61a** (0.1 mmol), sulfinylamine **I** (0.3 mmol), LiO^*t*^Bu (0.3 mmol) in 1,3-dioxolane (0.1 M) at 40 °C for 40 h under N_2_ atmosphere, isolated yield. Dr value by ^1^H NMR and ee value by chiral HPLC.

b At 40 °C for 3 days.

c At 40 °C for 5 days.

d At 25 °C for 40 h.

To demonstrate the broad applicability of this approach, a series of additional experiments were conducted (Scheme 1). When *ortho*-alkynylaniline **1a** and **52a** were reacted with reagent **I** on a 1.0 mmol scale under optimized conditions, compounds **1b** and **52b** were obtained with impressive yields, retaining their high stereoselectivity (Scheme 1A). Anhydrous methanesulfonic acid proved to be an effective deprotecting agent, converting compound **1b** and **52b** into the primary sulfinamide **62** and **63** with acceptable yields while preserving the enantioselectivity and diastereoselectivity.^75,76^ Furthermore, the formed sulfinamide **1b** served as a valuable precursor for the stereoselective synthesis of a variety of S-chiral compounds (Scheme 1B). For example, reacting compound **1b** with trichloroisocyanuric acid led to the complete conversion into sulfonimidoyl chloride, which could then be efficiently transformed into sulfonimidamides **64** and **65** by reacting with 1-aminopropan and morpholine, respectively, with good enantioselectivity.^83,84^ Using a similar procedure, the reaction of compound **1b** with NaN_3_ yielded compound **66** with 60% yield and 90% ee. Sulfonimidoyl fluorides are valuable intermediates in chemical synthesis due to their potential to be further converted into other important compound classes through sulfur­(VI) fluoride exchange reactions, a form of click chemistry.^85–87^ Treating compound **1b** with tetrabutylammonium fluoride resulted in the formation of chiral sulfonimidoyl fluoride **67**, obtained with a satisfactory yield, though with a slight decrease in enantioselectivity. Meanwhile, compound **52b** is a highly versatile precursor for the synthesis of various atropisomeric products, owing to the rich transformation potential of its aryl halide moieties (Scheme 1C). Through a sequence of halogen-magnesium exchange followed by nucleophilic substitution with allylic bromide and TsCN, compound **52b** can be efficiently converted into allylation product **68** and cyanation product **69**. These transformations proceed with modest yields while maintaining excellent enantioselectivity and diastereoselectivity. Particularly noteworthy is the efficient chiral induction observed in the reaction of **52b** with an aldehyde, which selectively affords stereoisomer **70** bearing three chiral elements with high stereocontrol. Additionally, the Sonogashira coupling of compound **54b** with 1-ethynyl-4-methoxybenzene proceeds efficiently under palladium catalysis, affording product **71** in 67% isolated yield with excellent diastereoselectivity (>15:1 dr) and high enantiopurity (93% ee).

**Scheme 1:**
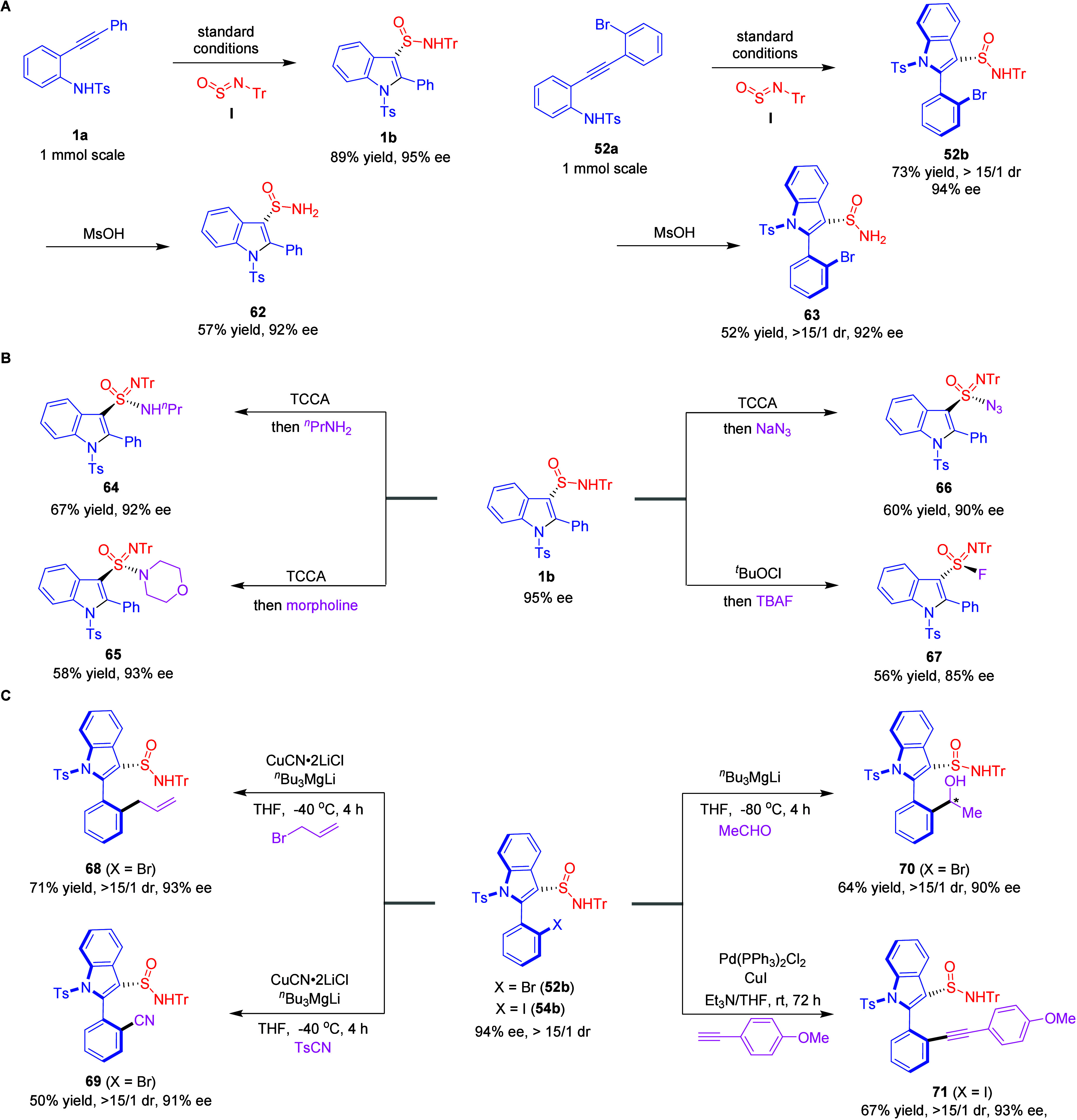
(A) Scale-up Synthesis of Sulfinamides **1b** and **52b** and Deprotection of the Trityl Group, (B) Follow-up Transformations of Sulfinamide **1b**,(C) Downstream Transformations of Sulfinamides **52b** and **54b**

Several experiments were conducted to elucidate the reaction pathway (Scheme 2). Reactions employing stoichiometric CuBr and ligand **L5** led to the formation of the complex [CuBr·**L5**] in good yield (Scheme 2A).^88^ Utilizing this complex as a catalyst in the reaction between substrates **1a** and **I** produced results of comparable efficacy, strongly suggesting that [CuBr·**L5**] serves as the active catalytic species. The nonlinear effect of the reaction was also examined (Scheme 2B). A clear linear relationship was observed when compounds **1a** and **I** were reacted using **L5** as the chiral ligand, suggesting that the ratio of Cu and **L5** is 1/1, without any self-aggregation of the catalyst.^89,90^ When indole **72** was directly used in the system, no desired product **1b** was obtained (Scheme 2C). This outcome suggests that the reaction does not proceed through the generation of free indole species. Instead, it indicates that the formation of the indolyl-Cu intermediate and the subsequent sulfinamidation step occur in a concerted manner. To further assess the likelihood of a radical-mediated mechanism, experiments were conducted with radical scavengers, including BHT and TEMPO (Scheme 2D). Our findings indicated that the synthesis of target compound **1b** was not significantly affected, effectively ruling out radical processes in the reaction. We also investigated the impact of *N*-substituents in *ortho*-alkynylanilines on the cyclization reaction (Scheme 2E). Substrates with NH_2_ (**1c**), NHMe (**1e**), and NHBoc (**1g**) substituents failed to produce the desired products. Using substrate **1i** with an NHMs substituent, we observed only trace amounts of product **1j**. Finally, the influence of different *N*-substituents on the sulfinylamines was examined (Scheme 2F). Sulfinylamines with Ts (**II**)^55^ substituents did not lead to the desired products **32c**. However, the use of sulfinylamine with phenyl (**III**)^91^ resulted in the product **32d** with 1.2/1 dr and 62% ee (38% ee). Further, the use of sulfinylamine **IV**, containing a sterically hindered triisopropylsilyl (TIPS) group, led to an improvement in both diastereoselectivity and enantioselectivity, affording product **32e** in 62% yield with 2.6/1 dr and 96% ee (26% ee). These findings suggest that the choice of sulfinylamine **I** ageous for achieving high conversion and stereoselectivity.

**Scheme 2:**
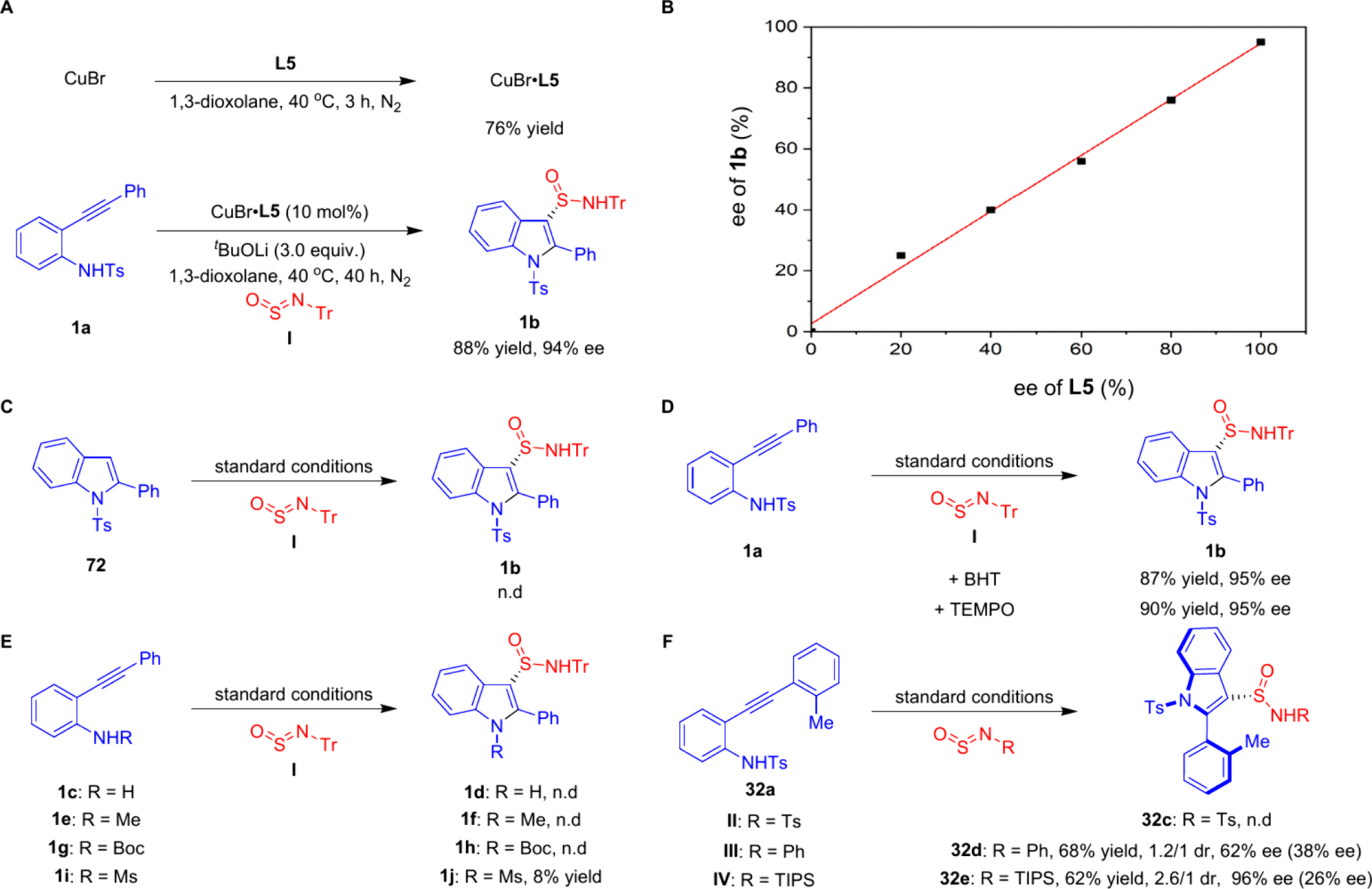
(A) Synthesis of Copper Complex and Testing Its Catalytic Activity, (B) Nonlinear Effect, (C) Direct Sulfinamidation Using Indole **72**, (D) Radical Quenching Experiments, (E) Testing Different *N*-Substituents in *ortho*-Alkynylanilines, (F) Testing Different *N*-Substituents in Sulfinylamines

Based on the aforementioned mechanistic experiments, density functional theory (DFT) was then performed to elucidate the detailed reaction pathway (Figure 2). The reaction begins with the coordination of Cu­(I) to the triple bond of substrate **32a**, forming intermediate **INT2A**. In the presence of a base, **INT2A** undergoes deprotonation to generate dipolar intermediate **INT3A**, which is characterized by a positive charge concentrated on the copper atom and a negative charge localized on the nitrogen atom. Subsequently, the nitrogen-negative center of **INT3A** performs a nucleophilic attack on the triple bond to generate the indole skeleton.^34,92^ Influenced by the chiral ligand, rotation of the aryl ring connected to the alkynyl group in the substrate is hindered, leading to the generation of axial chirality. In the unfavorable transition state **TS4A-aS**, the H···H distance between the methyl moiety of the substrate and the cyclopentadienyl ring skeleton of the chiral ligand is 2.19 Å. A notable steric repulsion exists in this configuration, thereby elevating the energy barrier associated with **TS4A-aS**. The calculation results indicate that the energy barrier of the five-membered-ring transition state **TS4A-aR** amounts to 17.1 kcal/mol relative to the zero-point energy. In comparison with the same reaction process proceeding through **TS4A-aS**, the energy barrier is diminished by 1.8 kcal/mol. This difference in energy bestows a distinct kinetic advantage, consequently determining the *aR*-configuration that is ultimately observable. The S-chiral center within the product is determined by the selective migratory insertion of the S=O bond of reagent **I** into **INT4A-aR**. The migratory insertion through transition state **TS5A-aR-S** is kinetically more favorable, with its energy being 2.0 kcal/mol lower than that of the pathway formed through transition state **TS5A-aR-R** (15.8 vs 17.8 kcal/mol), which aligns closely with the 99% ee observed in our experimental results. Subsequent investigation utilized the Independent Gradient Model (IGMH) based on Hirshfeld partitioning to analyze the noncovalent interactions between the substrate and chiral ligand fragments within the transition states **TS5A-aR-S** and **TS5A-aR-R**.^93^ Structural analysis reveals that in the transition state **TS5A-aR-S**, a wider green surface exists between the Tr group of sulfinylamine **I** and the aromatic ring of the chiral ligand. This phenomenon suggests the existence of strong dispersive interactions in the favorable transition state **TS5A-aR-S**. These dispersive forces play a crucial role in stabilizing the structure, leading to an activation energy barrier that is 2.0 kcal/mol lower than that of transition state **TS5A-aR-R**. The originally reported selective migratory insertion of the S=N bond is effectively excluded due to the significant steric space between the chiral ligand and the Tr group (see Supporting Information for details).^76^ Finally, transmetalation with an in situ generated Li complex forms the precursor for the *S*-configured product and regenerates active catalyst **INT1A**, completing the catalytic cycle.

**Figure 2:**
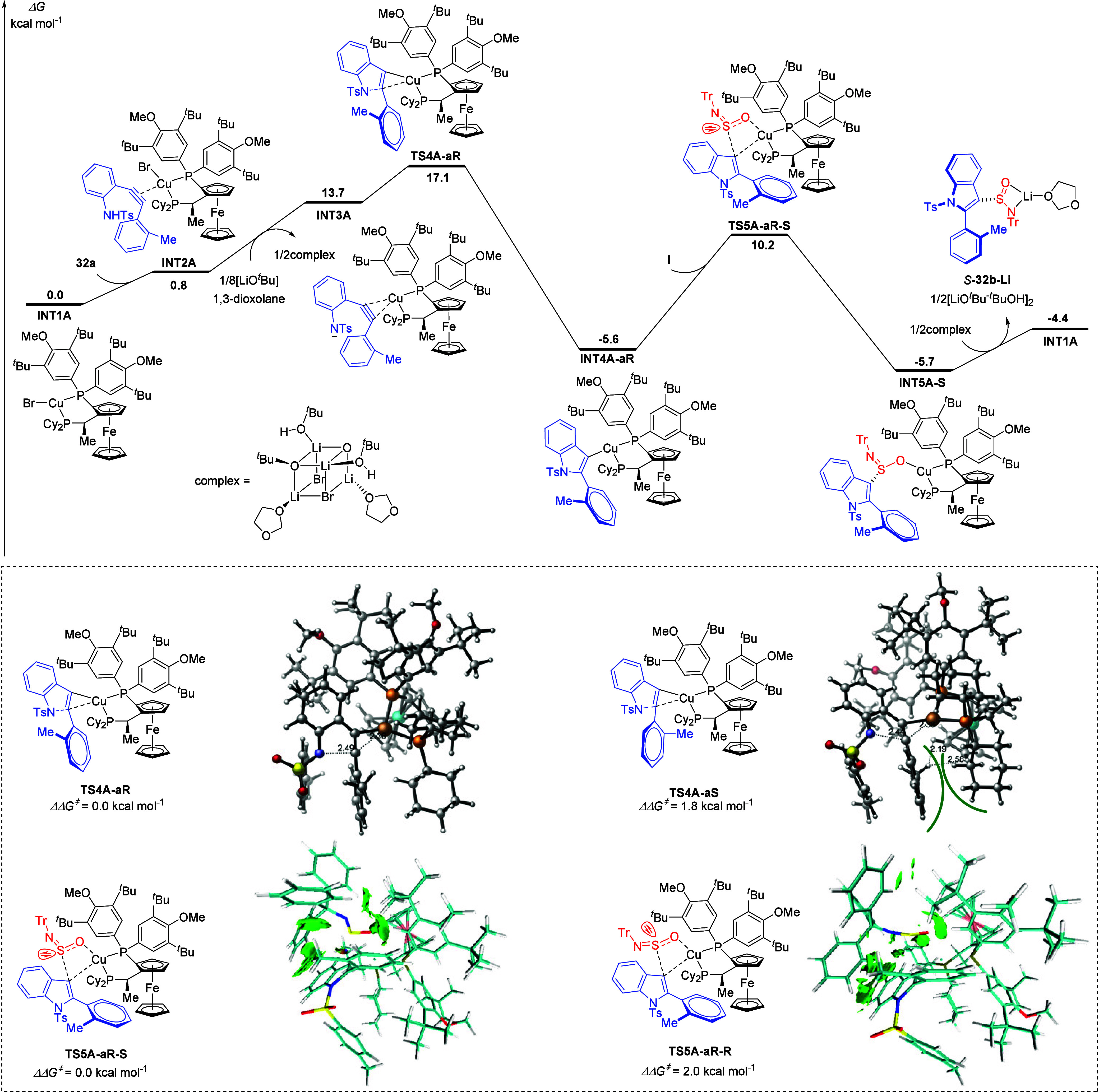
DFT-computed reaction pathways for the reaction of substrates **32a** and **I** (M06/6-311+G­(d,p)-SDD­(Cu and Fe), IEFPCM­(1,3-dioxolane)//B3LYP-D3BJ/6-31G­(d)-SDD­(Cu and Fe)).

## Conclusions

In summary, we have developed a reliable strategy for cyclizative sulfinamidation, enabling the synthesis of chiral indole-3-sulfinamides with stereogenic sulfur­(IV) centers and atropisomeric chirality from ortho-alkynylanilines and sulfinylamines using a chiral copper catalyst. The resulting compounds serve as versatile intermediates, facilitating the creation of a wide array of sulfur-containing pharmacophores through highly stereoselective conversion. This method expands the toolkit for synthesizing complex S-containing compounds, which is expected to benefit pharmaceutical and materials science.

## Supplementary Material







## Notes

Experimental procedures, characterizations, and analytical data of new compounds, and NMR and HPLC spectra for new compounds (PDF)
Cif files of **1b**, **32b**, and **55b**.(ZIP)
Transparent Peer Review report available (PDF)

## Notes

CCDC: 2419186 (**1b**), 2436916 (**32b**), and 2436917 (**55b**) contain the supplementary crystallographic data for this paper. These data can be obtained free of charge via www.ccdc.cam.ac.uk/data_request/cif, by emailing data_request@ccdc.cam.ac.uk, or by contacting The Cambridge Crystallographic Data Centre, 12 Union Road, Cambridge CB2 1EZ, UK; fax: + 44 1223 336033.
