# Supplementary material for: Copper-Catalyzed Asymmetric Cyclizative Sulfinamidation: Forging Indole-Based Stereogenic Sulfur(IV) Centers and Atropisomeric Chirality
Source: ACS Cent Sci. 2025 Aug 1;11(9):1762–72. doi: 10.1021/acscentsci.5c00909 (PMC12464752; doi:10.1021/acscentsci.5c00909)
Supplement: Supplementary file 1 [file oc5c00909_si_001.pdf]

Supporting Information

Copper-Catalyzed Asymmetric Cyclizative Sulfinamidation: Forging Indole-Based Stereogenic Sulfur(IV) Centers and Atropisomeric Chirality

Xiaowu Fang[a,+], Fengrui Xiang[a,+], Yue Zhao [a], and Zhuangzhi Shi [a,b,c]*

[a]State Key Laboratory of Coordination Chemistry, Chemistry and Biomedicine Innovation Center (ChemBIC), School of Chemistry and Chemical Engineering, Nanjing University, Nanjing 210093 (China)

[b]School of Chemistry and Materials Science, Nanjing Normal University, Nanjing 210023 (China)
[c]School of Chemistry and Chemical Engineering, Henan Normal University, Xinxiang 453007 (China)

[+]These authors contributed equally to this work.

*Corresponding author. E-mail: shiz@nju.edu.cn

Table of Contents

1 General Information....................S2

2 Experimental Procedures and Characterization of *ortho*-Alkynylanilines....................S3

3 Experimental Procedures and Characterization of Sulfinamides....................S16

4 Synthetic Transformations....................S52

5 Mechanistic Studies....................S61

6 Crystallographic Date....................S73

7 Computational Details....................S109

8 Copies of ^{1}H NMR, ^{13}C NMR , ^{19}F NMR and ^{31}P NMR Spectras....................S173

9 Copies of HPLC Spectras....................S284

10 References....................S360

1 General Information

All new compounds were fully characterized. All reactions and manipulations involving air-sensitive compounds were performed using standard Schlenk techniques or in a glovebox. Other reagents and solvents were directly used from the supplier without further purification unless noted. ^{1}H, ^{13}C and ^{19}F NMR spectra were recorded on a Bruker AVANCE III 400 MHz , 500 MHz or 600 MHz pectrometer. ^{1}H NMR spectra data were reported as δ values in ppm relative to chloroform (δ 7.26) if collected in $CDCl_3$. ^{13}C NMR spectra data were reported as δ values in ppm relative to chloroform (δ 77.16). ^{19}F NMR spectra data were reported as δ values in ppm. ^{31}P NMR spectra data were reported as δ values in ppm. Mass spectra were conducted at Micromass Q-Tof instrument (ESI) and Agilent Technologies 5973N (EI). IR spectra were recorded on a FT-IR spectrometer. Optical rotations were measured on an automatic polarimeter (Anton Paar MCP 5300) with $[\alpha]_D^{25}$ values reported in degrees; concentration (*c*) is in g/100 mL. Chiral HPLC analyses were performed on an UltiMate 3000 liquid chromatography. Unless otherwise noted, materials obtained from commercial suppliers were used without further purification. CuBr (CAS 7787-70-4) was purchased from J&K Scientific; **L5** (CAS 1453803-83-2) was purchased from Bidepharm; ^{t}BuOLi (CAS 1907-33-1) and 1,3-dioxolane (CAS 646-06-0, 99%, Extra Dry, with molecular sieves, stabilized with BHT, Water ≤50 ppm) were purchased from Energy chemical. The preparation of sulfinylamines **I**，**II**，**III** and **IV** were described according to the literatures.[1-3] The 2-phenyl-1-tosyl-1H-indole **72**[4] were described according to the literatures. Flash chromatography was performed using glass columns with silica gel (SiliaFlash® P60, particle size 40-63 μm, 230-400 mesh, Silicycle) after 1% triethylamine treatment.

2 Experimental Procedures and Characterization of *ortho*-Alkynylanilines

2.1 General Procedure to Synthesize *ortho*-Alkynylanilines

ortho-Alkynylanilines **1-61a**[5-6] were synthesized according to general procedure **A** or **B**. *ortho*-Alkynylanilines **1c**[7], **1e**[8], **1g**[9] and **1i**[8] were synthesized according to literature.

General Procedure A

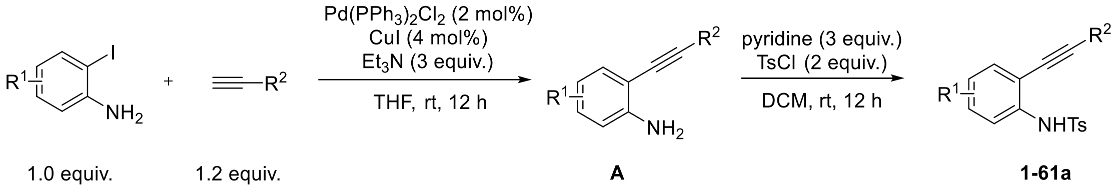

A three-necked flask charged with $Pd(PPh_3)_2Cl_2$ (28 mg, 2.0 mol%, 0.04 mmol) and CuI (15.3mg, 4 mol%, 0.08 mmol,) was evacuated and backfilled with N_2, then THF (5 mL), Et_3N (606 mg, 3.0 equiv., 6 mmol), alkyne (1.2 equiv., 2.4 mmol) and 2-iodoaniline (1.0 equiv., 2 mmol) were added sequentially. The reaction mixture was stirred at room temperature for 12 hours. H_2O was added and the aqueous layer was extracted by ethyl acetate 3 times, and the combined organic layer was dried over Na_2SO_4 and concentrated in vacuo. The residue was purified by flash column chromatography. Purification via flash column chromatography afforded the product **A**.

A three-necked flask charged with product **A** and backfilled with N_2, then DCM (5 mL), pyridine (474 mg, 3.0 equiv. 6 mmol) and TsCl (760 mg, 2.0 equiv. 4 mmol) were added sequentially. The reaction mixture was stirred at room temperature for 12 hours. H_2O was added and the aqueous layer was extracted by DCM 3 times, and the combined organic layer was dried over Na_2SO_4 and concentrated in vacuo. Purification via flash column chromatography afforded the product **1-61a**.

General Procedure B

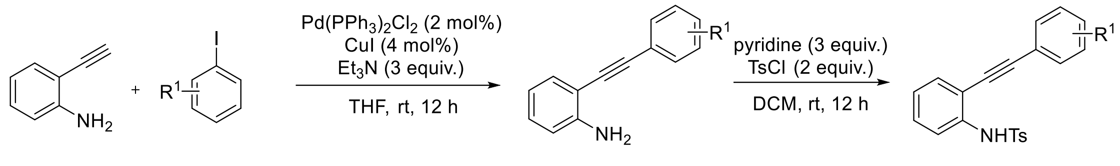

A three-necked flask charged with $Pd(PPh_3)_2Cl_2$ (28 mg, 2.0 mol%, 0.04 mmol) and CuI (15.3mg, 4 mol%, 0.08 mmol,) was evacuated and backfilled with N_2, then THF (5 mL), Et_3N (606 mg, 3.0 equiv., 6 mmol), 2-ethynylaniline (234 mg, 1.0 equiv., 2 mmol) and aryliodide (1.0 equiv., 2.4 mmol) were added sequentially. The reaction mixture was stirred at room temperature for 12 hours. H_2O was added and the aqueous layer was extracted by ethyl acetate 3 times, and the combined organic layer was dried over Na_2SO_4 and concentrated in vacuo. The residue was purified by flash column chromatography. Purification via flash column chromatography afforded the product **B**.

A three-necked flask charged with product **B** and backfilled with N_2, then DCM (5 mL), pyridine (474 mg, 3.0 equiv., 6 mmol) and TsCl (760 mg, 2.0 equiv., 4 mmol) were added sequentially. The reaction mixture was stirred at room temperature for 12 hours. H_2O was added and the aqueous layer was extracted by DCM 3 times, and the combined organic layer was dried over Na_2SO_4 and concentrated in vacuo. Purification via flash column chromatography afforded the product **1-61a**.

2.2 Characterization of *ortho*-Alkynylanilines

N-(2-((4-(dimethylamino)phenyl)ethynyl)phenyl)-4-methylbenzenesulfonamide (9a)

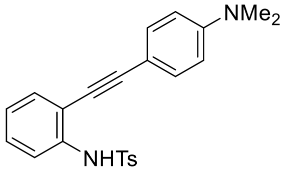

9a

According to general procedure **B**, white solid (472 mg, 61% yield); **R$_f$** = 0.5 (PE/EA = 5/1); NMR spectroscopy: **^{1}H NMR** (500 MHz, $CDCl_3$, 25 ºC) δ 7.68 (d, *J* = 8.3 Hz, 2H), 7.61 (d, *J* = 8.4 Hz, 1H), 7.37 – 7.31 (m, 3H), 7.28 (s, 1H), 7.25 – 7.21 (m, 1H), 7.16 (d, *J* = 8.1 Hz, 2H), 7.03 (td, *J* = 7.6, 1.2 Hz, 1H), 6.67 (d, *J* = 9.0 Hz,

2H), 3.02 (s, 6H), 2.33 (s, 3H); **^{13}C NMR** (126 MHz, $CDCl_3$, 25 ºC) δ 150.6, 144.0, 137.2, 136.2, 132.9, 131.6, 129.7, 128.8, 127.4, 124.6, 120.2, 115.7, 111.9, 108.5, 98.0, 81.8, 40.3, 21.6; **IR (ATR)**: 3314, 2914, 2198, 1603, 1523, 1345, 1161, 815, 667 cm^{-1}; **HRMS** (ESI, m/z): calcd for. $C_{23}H_{22}N_2O_2SNa^+$ $(M+Na)^+$: 413.1294; Found: 413.1286.

4-methyl-*N*-(2-((1-methyl-1H-indol-5-yl)ethynyl)phenyl)benzenesulfonamide (17a)

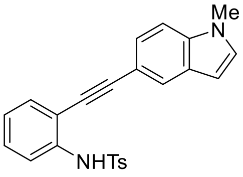

17a

According to general procedure **B**, white solid (432 mg, 54% yield); **R_f** = 0.6 (PE/EA = 5/1); NMR spectroscopy: **^{1}H NMR** (500 MHz, $CDCl_3$, 25 ºC) δ 7.78 (t, *J* = 1.2 Hz, 1H), 7.71 – 7.68 (m, 2H), 7.63 (dd, *J* = 8.3, 1.1 Hz, 1H), 7.38 (dd, *J* = 7.6, 1.6 Hz, 1H), 7.33 (d, *J* = 1.2 Hz, 3H), 7.29 – 7.23 (m, 1H), 7.16 (d, *J* = 7.7 Hz, 2H), 7.12 (d, *J* = 3.1 Hz, 1H), 7.06 (td, *J* = 7.6, 1.2 Hz, 1H), 6.53 (d, *J* = 3.1 Hz, 1H), 3.83 (s, 3H), 2.33 (s, 3H); **^{13}C NMR** (126 MHz, $CDCl_3$, 25 ºC) δ 144.0, 137.5, 136.8, 136.2, 131.8, 130.3, 129.7, 129.6, 129.1, 128.5, 127.5, 125.1, 124.7, 120.3, 115.6, 112.6, 109.7, 101.6, 98.4, 81.6, 33.1, 21.7; **IR (ATR)**: 3311, 2923, 2352, 2200, 1494, 1336, 1162, 751, 669 cm^{-1}; **HRMS** (ESI, m/z): calcd for. $C_{24}H_{20}N_2O_2SNa^+$ $(M+Na)^+$: 423.1138; Found: 423.1129.

4-methyl-*N*-(4-methyl-2-(o-tolylethynyl)phenyl)benzenesulfonamide (33a)

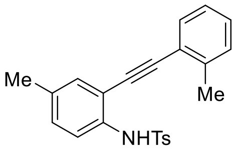

33a

According to general procedure **A**, off-white solid (546 mg, 73% yield); **R_f** = 0.6 (PE/EA = 7/1); NMR spectroscopy: **^{1}H NMR** (500 MHz, $CDCl_3$, 25 ºC) δ 7.65 (d, *J* = 8.3 Hz, 2H), 7.53 (d, *J* = 8.4 Hz, 1H), 7.42 (d, *J* = 7.6 Hz, 1H), 7.31 – 7.26 (m, 2H), 7.23 – 7.18 (m, 2H), 7.18 – 7.12 (m, 3H), 7.10 (dd, *J* = 8.4, 2.0 Hz, 1H), 2.47 (s, 3H), 2.33 (s, 3H), 2.27 (s, 3H); **^{13}C NMR** (126 MHz, $CDCl_3$, 25 ºC) δ 144.0, 140.0, 136.3, 135.1, 134.5, 132.4, 132.1, 130.6, 129.8, 129.7, 129.2, 127.4, 126.0, 122.1, 120.3, 114.8, 94.9, 88.0, 21.7, 21.1, 20.8; **IR (ATR)**: 3327, 2925, 2352, 1494, 1336, 1159, 898, 730, 667 cm^{-1}; **HRMS** (ESI, m/z): calcd for. $C_{23}H_{21}NO_2SNa^+$ $(M+Na)^+$:398.1185; Found: 398.1178.

4-methyl-*N*-(5-methyl-2-(o-tolylethynyl)phenyl)benzenesulfonamide (34a)

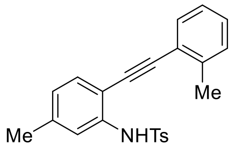

34a

According to general procedure **A**, off-white solid (501 mg, 67% yield); $\mathbf{R_f}$ = 0.6 (PE/EA = 7/1); NMR spectroscopy: **^{1}H NMR** (500 MHz, $CDCl_3$, 25 ºC) δ 7.70 (d, *J* = 8.3 Hz, 2H), 7.49 (s, 1H), 7.45 (d, *J* = 7.6 Hz, 1H), 7.33 – 7.27 (m, 3H), 7.26 – 7.17 (m, 4H), 6.90 (dd, *J* = 7.9, 2.2 Hz, 1H), 2.50 (s, 3H), 2.38 (s, 3H), 2.36 (s, 3H); **^{13}C NMR** (126 MHz, $CDCl_3$, 25 ºC) δ 144.1, 140.4, 139.9, 137.5, 136.3, 132.0, 131.8, 129.8, 129.7, 129.1, 127.4, 125.9, 125.5, 122.2, 120.4, 111.7, 94.8, 87.9, 22.0, 21.7, 21.1; **IR (ATR)**: 3326, 3055, 2951, 1395, 1161. 888, 728, 542 cm^{-1}; **HRMS** (ESI, m/z): calcd for. $C_{23}H_{22}NO_2S^+$ $(M+H)^+$: 376.1366; Found: 376.1358.

***N*-(4,5-dimethyl-2-(o-tolylethynyl)phenyl)-4-methylbenzenesulfonamide (35a)**

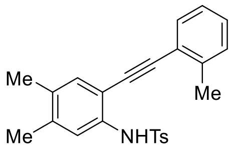

35a

According to general procedure **A**, off-white solid (597 mg, 77% yield); $\mathbf{R_f}$ = 0.6 (PE/EA = 7/1); NMR spectroscopy: **^{1}H NMR** (500 MHz, $CDCl_3$, 25 ºC) δ 7.64 (d, *J* = 8.3 Hz, 2H), 7.43 (s, 1H), 7.39 (d, *J* = 6.3 Hz, 1H), 7.29 – 7.23 (m, 2H), 7.19 (t, *J* = 6.3 Hz, 1H), 7.16 – 7.11 (m, 3H), 7.07 (s, 1H), 2.45 (s, 3H), 2.32 (s, 3H), 2.26 (s, 3H), 2.17 (s, 3H); **^{13}C NMR** (126 MHz, $CDCl_3$, 25 ºC) δ 143.9, 139.9, 139.0, 136.4, 135.2, 133.4, 132.6, 131.9, 129.8, 129.7, 128.9, 127.3, 125.9, 122.3, 121.7, 112.3, 94.3, 88.1, 21.6, 21.1, 20.3, 19.2; **IR (ATR)**: 3327, 3054, 2927, 2352, 1499, 1333, 1266, 1160, 898, 730 cm^{-1}; **HRMS** (ESI, m/z): calcd for. $C_{24}H_{24}NO_2S^+$ $(M+H)^+$: 390.1522; Found: 390.1517.

***N*- (4-methoxy-2-(o-tolylethynyl)phenyl)-4-methylbenzenesulfonamide (36a)**

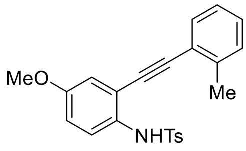

36a

According to general procedure **A**, off-white solid (435 mg, 56% yield); $\mathbf{R_f}$ = 0.4 (PE/EA = 7/1); NMR spectroscopy: **^{1}H NMR** (500 MHz, $CDCl_3$, 25 ºC) δ 7.61 (dd, *J* = 9.2, 3.5 Hz, 3H), 7.41 (d, *J* = 9.0 Hz, 1H), 7.34 – 7.26 (m, 2H), 7.23 (t, *J* = 8.0 Hz, 1H), 7.14 (d, *J* = 6.8 Hz, 2H), 6.95 (s, 1H), 6.93 – 6.88 (m, 2H), 3.80 (s, 3H), 2.46 (s, 3H), 2.34 (s, 3H); **^{13}C NMR** (126 MHz, $CDCl_3$, 25 ºC) δ 156.8, 143.9, 140.1, 136.2, 132.1, 130.7, 129.8, 129.6, 129.3, 127.4, 125.9, 123.4, 121.9, 117.1, 116.4, 116.0, 94.7, 87.9, 55.7,

21.6, 21.1; **IR (ATR)**: 3055, 2351 1418, 1264, 1164, 897, 727 cm^{-1}; **HRMS** (ESI, m/z): calcd for. $C_{23}H_{22}NO_3S^+$ $(M+H)^+$: 392.1315; Found: 392.1306.

***N*-(5-methoxy-2-(o-tolylethynyl)phenyl)-4-methylbenzenesulfonamide (37a)**

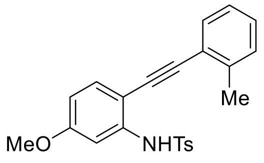

37a

According to general procedure **A**, off-white solid (535 mg, 68% yield); $\mathbf{R_f}$ = 0.4 (PE/EA = 7/1); NMR spectroscopy: **^{1}H NMR** (500 MHz, $CDCl_3$, 25 ºC) δ 7.76 – 7.70 (m, 2H), 7.45 (d, *J* = 7.3 Hz, 1H), 7.34 – 7.28 (m, 4H), 7.25 – 7.20 (m, 4H), 6.63 (dd, *J* = 8.6, 2.5 Hz, 1H), 3.84 (s, 3H), 2.51 (s, 3H), 2.37 (s, 3H); **^{13}C NMR** (126 MHz, $CDCl_3$, 25 ºC) δ 160.7, 144.2, 139.8, 139.0, 136.2, 133.0, 131.9, 129.8, 129.8, 128.9, 127.4, 125.9, 122.4, 110.9, 106.5, 104.8, 94.2, 87.8, 55.7, 21.7, 21.2; **IR (ATR)**: 3332, 2352, 1507, 1337, 1164, 1091, 902, 721 cm^{-1}; **HRMS** (ESI, m/z): calcd for. $C_{23}H_{22}NO_3S^+$ $(M+H)^+$:392.1315; Found: 392.1312.

***N*-(5-fluoro-2-(o-tolylethynyl)phenyl)-4-methylbenzenesulfonamide (38a)**

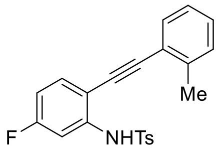

38a

According to general procedure **A**, off-white solid (464 mg, 61% yield); $\mathbf{R_f}$ = 0.75 (PE/EA = 7/1); NMR spectroscopy: **^{1}H NMR** (500 MHz, $CDCl_3$, 25 ºC) δ 7.73 (d, *J* = 8.4 Hz, 2H), 7.46 (d, *J* = 6.3 Hz, 1H), 7.42 – 7.35 (m, 3H), 7.33 – 7.26 (m, 2H), 7.23 (d, *J* = 8.7 Hz, 3H), 6.76 (td, *J* = 8.3, 2.5 Hz, 1H), 2.49 (s, 3H), 2.37 (s, 3H); **^{13}C NMR** (126 MHz, $CDCl_3$, 25 ºC) δ 163.0 (d, *J* = 249.9 Hz), 144.6, 140.0, 139.3 (d, *J* = 11.6 Hz), 136.0, 133.4 (d, *J* = 9.7 Hz), 132.1, 130.4, 129.9 (d, *J* = 10.1 Hz), 129.3, 127.4, 127.2, 126.0, 121.8, 111.5 (d, *J* = 22.5 Hz), 109.9 (d, *J* = 3.4 Hz), 95.3 (d, *J* = 1.8 Hz), 86.7, 21.7, 21.1; **^{19}F NMR** (471 MHz, $CDCl_3$, 25 ºC) δ -107.0 (s). **IR (ATR)**: 3329, 3058, 2351, 1500, 1267, 1162, 884, 729 cm^{-1}; **HRMS** (ESI, m/z): calcd for. $C_{22}H_{19}FNO_2S^+$ $(M+H)^+$: 380.1115; Found: 380.1109.

***N*-(4,5-difluoro-2-(o-tolylethynyl)phenyl)-4-methylbenzenesulfonamide (39a)**

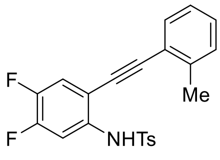
39a

According to general procedure **A**, off-white solid (387 mg, 49% yield); **R$_f$** = 0.7 (PE/EA = 10/1); NMR spectroscopy: **^{1}H NMR** (500 MHz, $CDCl_3$, 25 ºC) δ 7.70 (d, *J* = 7.9 Hz, 2H), 7.62 – 7.49 (m, 1H), 7.45 (d, *J* = 7.6 Hz, 1H), 7.27 (ddd, *J* = 43.4, 17.6, 9.3 Hz, 7H), 2.48 (s, 3H), 2.39 (s, 3H); **^{13}C NMR** (126 MHz, $CDCl_3$, 25 ºC) δ 150.7 (dd, *J* = 252.9, 13.5 Hz), 147.1 (dd, *J* = 248.1, 13.7 Hz), 144.6, 140.2, 135.8, 134.6, 134.5 (d, *J* = 2.5 Hz), 132.1, 130.0, 129.9, 129.7, 127.4, 126.1, 121.4, 120.1 (d, *J* = 19.5 Hz), 109.9 (d, *J* = 22.5 Hz), 95.9, 85.8, 21.7, 21.1; **^{19}F NMR** (471 MHz, $CDCl_3$, 25 ºC) δ -131.5 (d, *J* = 22.0 Hz), -141.3 (d, *J* = 22.0 Hz). **IR (ATR)**: 3330, 3062, 2351, 1600, 1509, 1342, 1161, 898, 7320, 667 cm^{-1}; **HRMS** (ESI, m/z): calcd for. $C_{22}H_{18}F_2NO_2S^+$ $(M+H)^+$: 398.1021; Found: 398.1019.

***N*-(5-chloro-2-(o-tolylethynyl)phenyl)-4-methylbenzenesulfonamide (40a)**

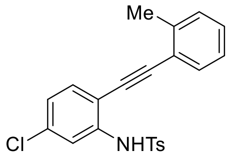
40a

According to general procedure **A**, off-white solid (620 mg, 78% yield); **R$_f$** = 0.6 (PE/EA = 7/1); NMR spectroscopy: **^{1}H NMR** (500 MHz, $CDCl_3$, 25 ºC) δ 7.71 (d, *J* = 8.1 Hz, 2H), 7.67 (s, 1H), 7.45 (d, *J* = 7.6 Hz, 1H), 7.34 – 7.26 (m, 4H), 7.25 – 7.19 (m, 3H), 7.03 (dd, *J* = 8.3, 2.1 Hz, 1H), 2.48 (s, 3H), 2.36 (s, 3H); **^{13}C NMR** (126 MHz, $CDCl_3$, 25 ºC) δ 144.5, 140.1, 138.1, 136.0, 135.5, 132.8, 132.1, 130.0, 129.9, 129.5, 127.4, 126.0, 124.6, 121.7, 119.4, 112.6, 96.4, 86.7, 21.7, 21.1; **IR (ATR)**: 2984, 2352, 1735, 1373, 1235, 1044, 921, 733 cm^{-1}; **HRMS** (ESI, m/z): calcd for. $C_{22}H_{19}ClNO_2S^+$ $(M+H)^+$: 396.0820; Found: 396.0817.

***N*-(4-chloro-2-(o-tolylethynyl)phenyl)-4-methylbenzenesulfonamide (41a)**

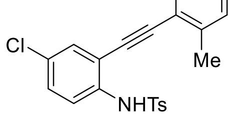
41a

According to general procedure **A**, white solid (588 mg, 74% yield); **R$_f$** = 0.7 (PE/EA = 7/1); NMR spectroscopy: **^{1}H NMR** (500 MHz, $CDCl_3$, 25 ºC) δ 7.69 (d, *J* = 8.3 Hz, 2H), 7.61 (d, *J* = 8.9 Hz, 1H), 7.46 (d, *J* = 7.7 Hz, 1H), 7.38 (d, *J* = 2.4 Hz, 1H), 7.34 (td, *J* = 7.5, 1.4 Hz, 1H), 7.32 – 7.18 (m, 6H), 2.49 (s, 3H), 2.37 (s, 3H); **^{13}C NMR** (126 MHz,

$CDCl_3$, 25 ºC) δ 144.4, 140.2, 136.2, 132.2, 131.5, 130.4, 129.9, 129.9, 129.7, 129.6, 127.3, 127.2, 126.0, 121.5, 121.1, 116.2, 96.4, 86.5, 21.7, 21.1; **IR (ATR)**: 3327, 3060, 2925, 2351, 1481 ,1389, 1337, 1160, 1091, 875, 809, 662, 541 cm^{-1}; **HRMS** (ESI, m/z): calcd for. $C_{22}H_{18}ClNO_2SK^+$ $(M+K)^+$: 434.0378; Found: 434.0372.

***N*-(4-bromo-2-(o-tolylethynyl)phenyl)-4-methylbenzenesulfonamide (42a)**

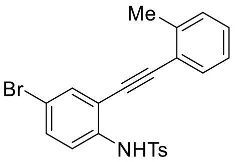

42a

According to general procedure **A**, white solid (667 mg, 76% yield); $\mathbf{R_f}$ = 0.7 (PE/EA = 7/1); NMR spectroscopy: **^{1}H NMR** (500 MHz, $CDCl_3$, 25 ºC) δ 7.59 (d, *J* = 7.9 Hz, 2H), 7.44 (d, *J* = 8.9 Hz, 2H), 7.33 (dd, *J* = 21.7, 8.2 Hz, 2H), 7.23 (t, *J* = 7.4 Hz, 1H), 7.19 (d, *J* = 7.3 Hz, 1H), 7.17 – 7.06 (m, 4H), 2.39 (s, 3H), 2.27 (s, 3H); **^{13}C NMR** (126 MHz, $CDCl_3$, 25 ºC) δ 144.4, 140.2, 136.7, 135.9, 134.4, 132.6, 132.2, 129.9, 129.6, 127.3, 126.0, 121.5, 121.2, 117.2, 116.5, 96.6, 86.3, 21.7, 21.1; **IR (ATR)**: 3056, 2352, 1483, 1265, 1165, 905, 723 cm^{-1}; **HRMS** (ESI, m/z): calcd for. $C_{22}H_{19}BrNO_2S^+$ $(M+H)^+$: 440.0314; Found: 440.0309.

***N*-(2-((2,4-dimethylphenyl)ethynyl)phenyl)-4-methylbenzenesulfonamide (43a)**

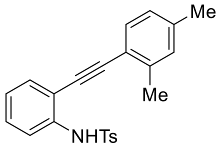

43a

According to general procedure **B**, white solid (534 mg, 71% yield); $\mathbf{R_f}$ = 0.5 (PE/EA = 7/1); NMR spectroscopy: **^{1}H NMR** (500 MHz, $CDCl_3$, 25 ºC) δ 7.67 (d, *J* = 8.4 Hz, 2H), 7.63 (d, *J* = 8.2 Hz, 1H), 7.37 (dd, *J* = 7.7, 1.5 Hz, 1H), 7.33 (d, *J* = 7.8 Hz, 1H), 7.28 (s, 1H), 7.26 (d, *J* = 7.2 Hz, 1H), 7.16 (d, *J* = 8.1 Hz, 2H), 7.08 (s, 1H), 7.03 (qd, *J* = 7.8, 1.6 Hz, 2H), 2.44 (s, 3H), 2.36 (s, 3H), 2.33 (s, 3H); **^{13}C NMR** (126 MHz, $CDCl_3$, 25 ºC) δ 144.1, 139.9, 139.5, 137.5, 136.2, 131.99, 131.97, 130.7, 129.7, 129.5, 127.4, 126.8, 124.4, 119.6, 119.0, 114.7, 95.8, 87.0, 21.7, 21.6, 21.0; **IR (ATR)**: 3328, 3055, 2352, 1402, 1265, 1162, 909, 728, 560 cm^{-1}; **HRMS** (ESI, m/z): calcd for. $C_{23}H_{21}NO_2SNa^+$ $(M+Na)^+$: 398.1185; Found: 398.1179.

***N*-(2-((2,5-dimethylphenyl)ethynyl)phenyl)-4-methylbenzenesulfonamide (44a)**

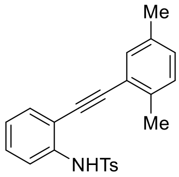

44a

According to general procedure **B**, white solid (511 mg, 69% yield); **Rf** = 0.7 (PE/EA = 7/1); NMR spectroscopy: **^{1}H NMR** (500 MHz, $CDCl_3$, 25 ºC) δ 7.60 (d, *J* = 8.4 Hz, 2H), 7.55 (d, *J* = 7.2 Hz, 1H), 7.30 (dd, *J* = 7.7, 1.6 Hz, 1H), 7.25 – 7.17 (m, 3H), 7.12 – 7.06 (m, 3H), 7.03 (t, *J* = 7.6 Hz, 1H), 6.97 (td, *J* = 7.6, 1.2 Hz, 1H), 2.35 (s, 3H), 2.25 (s, 3H), 2.24 (s, 3H); **^{13}C NMR** (126 MHz, $CDCl_3$, 25 ºC) δ 144.1, 138.5, 137.6, 137.2, 136.2, 132.0, 130.9, 130.0, 129.7, 129.6, 127.4, 125.7, 124.5, 122.1, 119.6, 114.7, 96.2, 87.1, 21.6, 20.5, 17.9; **IR (ATR)**: 3326, 3057, 2926, 2352, 1488, 1338, 1161, 1092, 915, 748, 666 cm^{-1}; **HRMS** (ESI, m/z): calcd for. $C_{23}H_{21}NO_2SK^+$ $(M+K)^+$: 414.0925; Found: 414.0917.

4-methyl-*N*-(2-((2-methyl-[1,1'-biphenyl]-3-yl)ethynyl)phenyl)benzenesulfonami-de (45a)

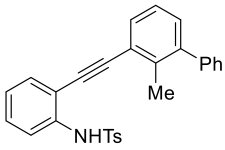

45a

According to general procedure **A**, white solid (479 mg, 55% yield); **Rf** = 0.7 (PE/EA = 7/1); NMR spectroscopy: **^{1}H NMR** (500 MHz, $CDCl_3$, 25 ºC) δ 7.69 (d, *J* = 8.4 Hz, 2H), 7.64 (d, *J* = 8.3 Hz, 1H), 7.49 – 7.42 (m, 3H), 7.42 – 7.36 (m, 2H), 7.34 – 7.24 (m, 6H), 7.18 (d, *J* = 8.0 Hz, 2H), 7.06 (t, *J* = 7.6 Hz, 1H), 2.40 (s, 3H), 2.34 (s, 3H); **^{13}C NMR** (126 MHz, $CDCl_3$, 25 ºC) δ 144.2, 142.8, 141.4, 137.7, 137.5, 136.2, 132.1, 131.3, 130.9, 129.8, 129.7, 129.3, 128.4, 127.4, 127.3, 125.8, 124.4, 122.9, 119.5, 114.4, 95.9, 87.7, 21.7, 19.2; **IR (ATR)**:3328, 3057, 2352, 1574, 1459, 1263, 1163, 1088, 921, 729 cm^{-1}; **HRMS** (ESI, m/z): calcd for. $C_{28}H_{23}NO_2SK^+$ $(M+K)^+$: 476.1081; Found: 476.1074

***N*-(2-((3-methoxy-2-methylphenyl)ethynyl)phenyl)-4-methylbenzenesulfonamidee (46a)**

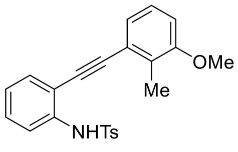

46a

According to general procedure **B**, white solid (314 mg, 40% yield); **Rf** = 0.6 (PE/EA = 7/1); NMR spectroscopy: **^{1}H NMR** (500 MHz, $CDCl_3$, 25 ºC) δ 7.75 – 7.63 (m, 3H), 7.41 (dd, *J* = 7.8, 1.6 Hz, 1H), 7.34 – 7.28 (m, 2H), 7.24 – 7.15 (m, 3H), 7.13 – 7.05 (m,

2H), 6.91 (d, J = 8.2 Hz, 1H), 3.89 (s, 3H), 2.39 (s, 3H), 2.36 (s, 3H); **^{13}C NMR** (126 MHz, $CDCl_3$, 25 ºC) δ 157.8, 144.1, 137.6, 136.2, 132.1, 129.8, 129.7, 129.0, 127.4, 126.6, 124.5, 124.3, 123.1, 119.9, 114.7, 111.2, 95.5, 87.3, 55.8, 21.7, 14.2; **IR (ATR)**: 3056, 2352, 1459, 1264, 1165, 906, 725 cm^{-1}; **HRMS** (ESI, m/z): calcd for. $C_{23}H_{22}NO_3S^+$ $(M+H)^+$: 392.1315; Found: 392.1309.

4-methyl-*N*-(2-((2-methyl-4-(trifluoromethoxy)phenyl)ethynyl)phenyl)benzenesulfonamide (47a)

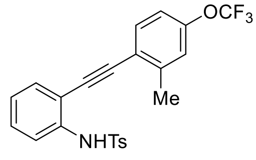

47a

According to general procedure **B**, white solid (453 mg, 51% yield); **R_f** = 0.6 (PE/EA = 7/1); NMR spectroscopy: **^{1}H NMR** (500 MHz, $CDCl_3$, 25 ºC) δ 7.68 (d, J = 8.4 Hz, 2H), 7.62 (d, J = 8.3 Hz, 1H), 7.46 (d, J = 8.4 Hz, 1H), 7.39 (dd, J = 7.7, 1.5 Hz, 1H), 7.34 – 7.28 (m, 1H), 7.21 (s, 1H), 7.19 (s, 1H), 7.18 (s, 1H), 7.12 (s, 1H), 7.07 (td, J = 7.6, 1.1 Hz, 2H), 2.49 (s, 3H), 2.35 (s, 3H); **^{13}C NMR** (126 MHz, $CDCl_3$, 25 ºC) δ 149.4, 144.3, 142.4, 137.7, 136.3, 133.5, 132.2, 130.0, 129.8, 127.4, 124.5, 122.1, 120.8, 120.5 (q, J = 257.9 Hz), 119.7, 118.4, 114.1, 93.9, 88.5, 21.7, 21.2; **^{19}F NMR** (471 MHz, $CDCl_3$, 25 ºC) δ -57.6; **IR (ATR)**: 3328, 3061, 2360, 1573, 1264, 1189, 920, 789, 630 cm^{-1}; **HRMS** (ESI, m/z): calcd for. $C_{23}H_{18}F_3NO_3SK^+$ $(M+K)^+$: 484.0591; Found: 484.0852.

***N*-(2-((4-fluoro-2-methylphenyl)ethynyl)phenyl)-4-methylbenzenesulfonamide (48a)**

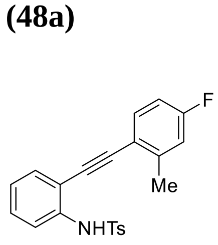

48a

According to general procedure **B**, light yellow solid (403 mg, 53% yield); **R_f** = 0.7 (PE/EA = 7/1); NMR spectroscopy: **^{1}H NMR** (500 MHz, $CDCl_3$, 25 ºC) δ 7.67 (dd, J = 29.1, 8.1 Hz, 3H), 7.48 – 7.36 (m, 2H), 7.30 (d, J = 14.8 Hz, 2H), 7.21 (d, J = 7.8 Hz, 2H), 7.13 – 6.89 (m, 3H), 2.50 (s, 3H), 2.37 (s, 3H). **^{13}C NMR** (126 MHz, $CDCl_3$, 25 ºC) δ 162.9 (d, J = 250.8 Hz), 144.2, 142.9, 142.8, 137.6, 136.3, 133.9 (d, J = 9.0 Hz), 132.1, 129.8, 127.4, 124.5, 119.7, 118.1, 117.0 (d, J = 21.9 Hz), 114.4, 113.3 (d, J = 22.3 Hz), 94.4,

87.4, 21.7, 21.2; **^{19}F NMR** (471 MHz, $CDCl_3$, 25 ºC) δ -110.0. **IR (ATR)**: 3331, 3058, 2926, 2352, 1495, 1270, 1159, 911, 732 cm^{-1}; **HRMS** (ESI, m/z): calcd for. $C_{22}H_{18}FNO_2SK^+$ $(M+K)^+$: 418.0674; Found: 418.0664.

***N*-(2-((5-chloro-2-methylphenyl)ethynyl)phenyl)-4-methylbenzenesulfonamide (49a)**

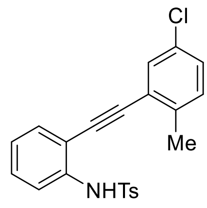

49a

According to general procedure **B**, white solid (466 mg, 59% yield); **R_f** = 0.65 (PE/EA = 7/1); NMR spectroscopy: **^{1}H NMR** (500 MHz, $CDCl_3$, 25 ºC) δ 7.70 (d, *J* = 8.4 Hz, 2H), 7.66 (d, *J* = 8.3 Hz, 1H), 7.41 (dd, *J* = 7.8, 1.6 Hz, 1H), 7.39 (d, *J* = 2.3 Hz, 1H), 7.34 (td, *J* = 7.9, 1.6 Hz, 1H), 7.29 – 7.18 (m, 5H), 7.10 (td, *J* = 7.6, 1.2 Hz, 1H), 2.46 (s, 3H), 2.37 (s, 3H). **^{13}C NMR** (126 MHz, $CDCl_3$, 25 ºC) δ 144.2, 138.4, 137.6, 136.2, 132.2, 131.5, 131.4, 131.0, 130.0, 129.8, 129.2, 127.3, 124.6, 123.6, 120.1, 114.2, 93.8, 88.7, 21.6, 20.5; **IR (ATR)**: 2984, 2352, 1735, 1373, 1234, 1044, 926, 845, 619 cm^{-1}; **HRMS** (ESI, m/z): calcd for. $C_{22}H_{18}ClNO_2SK^+$ $(M+K)^+$: 434.0378; Found: 434.0375.

***N*-(2-((2-bromo-5-chlorophenyl)ethynyl)phenyl)-4-methylbenzenesulfonamide (53a)**

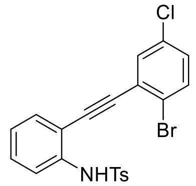

53a

According to general procedure **B**, off-white solid (589 mg, 64% yield); **R_f** = 0.7 (PE/EA = 7/1); NMR spectroscopy: **^{1}H NMR** (500 MHz, $CDCl_3$, 25 ºC) δ 7.75 (s, 1H), 7.73 (d, *J* = 8.2 Hz, 2H), 7.68 (d, *J* = 8.3 Hz, 1H), 7.58 (d, *J* = 8.6 Hz, 1H), 7.47 (d, *J* = 2.6 Hz, 1H), 7.39 (dd, *J* = 7.7, 1.6 Hz, 1H), 7.35 – 7.29 (m, 1H), 7.21 (dd, *J* = 8.6, 2.5 Hz, 1H), 7.17 (d, *J* = 8.0 Hz, 2H), 7.05 (t, *J* = 7.6 Hz, 1H), 2.33 (s, 3H); **^{13}C NMR** (126 MHz, $CDCl_3$, 25 ºC) δ 144.2, 138.7, 136.2, 133.5, 133.5, 132.5, 132.1, 130.6, 130.2, 129.8, 127.5, 126.1, 124.2, 123.5, 119.1, 112.7, 93.8, 90.0, 21.6; **IR (ATR)**: 3296, 3065, 2925, 2352, 1580, 1457, 1399, 1159, 1090, 915, 752 cm^{-1}; **HRMS** (ESI, m/z): calcd for. $C_{21}H_{15}BrClNO_2SK^+$ $(M+K)^+$: 497.9327; Found: 497.9324.

N-(2-((2-iodophenyl)ethynyl)phenyl)-4-methylbenzenesulfonamidee (54a)

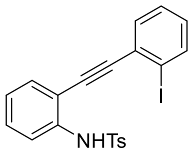

54a

According to general procedure **B**, off-white solid (386 mg, 41% yield); **R_f** = 0.6 (PE/EA = 7/1); NMR spectroscopy: **^{1}H NMR** (500 MHz, $CDCl_3$, 25 ºC) δ 7.91 (d, *J* = 8.2 Hz, 2H), 7.76 (d, *J* = 7.9 Hz, 2H), 7.68 (d, *J* = 8.3 Hz, 1H), 7.50 (d, *J* = 7.7 Hz, 1H), 7.42 (d, *J* = 7.7 Hz, 1H), 7.37 (t, *J* = 7.6 Hz, 1H), 7.30 (t, *J* = 7.8 Hz, 1H), 7.17 (d, *J* = 8.0 Hz, 2H), 7.12 – 7.00 (m, 2H), 2.33 (s, 3H); **^{13}C NMR** (126 MHz, $CDCl_3$, 25 ºC) δ 144.1, 138.7, 138.5, 136.3, 132.6, 132.3, 130.2, 130.1, 129.8, 129.2, 128.1, 127.6, 124.1, 119.1, 113.2, 101.0, 98.3, 87.9, 21.7; **IR (ATR)**: 3056, 2352, 1425, 1264, 727 cm^{-1}; **HRMS** (ESI, m/z): calcd for. $C_{21}H_{16}INO_2SK^+$ $(M+K)^+$: 511.9578; Found: 511.9571.

N-(2-((4-methoxynaphthalen-1-yl)ethynyl)phenyl)-4-methylbenzenesulfonamide (56a)

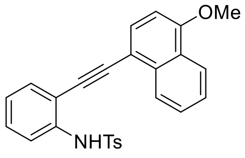

56a

According to general procedure **B**, off-white solid (386 mg, 45% yield); **R_f** = 0.5 (PE/EA = 7/1); NMR spectroscopy: **^{1}H NMR** (500 MHz, $CDCl_3$, 25 ºC) δ 8.32 (d, *J* = 8.2 Hz, 1H), 8.13 (d, *J* = 8.4 Hz, 1H), 7.70 – 7.61 (m, 5H), 7.56 (ddd, *J* = 8.2, 6.8, 1.3 Hz, 1H), 7.47 (dd, *J* = 7.7, 1.5 Hz, 1H), 7.36 – 7.28 (m, 2H), 7.15 – 7.07 (m, 3H), 6.84 (d, *J* = 8.0 Hz, 1H), 4.07 (s, 3H), 2.29 (s, 3H); **^{13}C NMR** (126 MHz, $CDCl_3$, 25 ºC) δ 156.8, 144.1, 137.5, 136.3, 134.0, 132.1, 131.8, 129.7, 129.5, 127.8, 127.4, 126.1, 125.7, 125.6, 124.7, 122.7, 120.3, 115.3, 111.8, 103.8, 95.0, 87.0, 55.9, 21.7; **IR (ATR)**: 3323, 3057, 2352, 1580, 1264, 1162, 1092, 905, 728 cm^{-1}; **HRMS** (ESI, m/z): calcd for. $C_{26}H_{21}NO_3SK^+$ $(M+K)^+$: 466.0874; Found: 466.0867.

N-(2-((4-bromonaphthalen-1-yl)ethynyl)phenyl)-4-methylbenzenesulfonamide (57a)

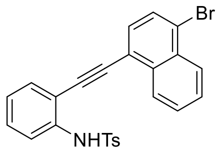

57a

According to general procedure **B**, grey solid (442 mg, 53% yield); $\mathbf{R_f}$ = 0.6 (PE/EA = 7/1); NMR spectroscopy: **^{1}H NMR** (500 MHz, $CDCl_3$, 25 ºC) δ 8.34 – 8.28 (m, 1H), 8.21 (dt, *J* = 7.9, 2.8 Hz, 1H), 7.80 (d, *J* = 7.7 Hz, 1H), 7.68 (d, *J* = 8.9 Hz, 5H), 7.53 (d, *J* = 7.8 Hz, 1H), 7.50 (dd, *J* = 7.8, 1.5 Hz, 1H), 7.35 (td, *J* = 7.9, 1.6 Hz, 1H), 7.29 (s, 1H), 7.13 (dd, *J* = 7.9, 2.2 Hz, 3H), 2.29 (s, 3H); **^{13}C NMR** (126 MHz, $CDCl_3$, 25 ºC) δ 144.2, 137.7, 136.3, 134.0, 132.3, 132.0, 130.8, 130.1, 129.8, 129.6, 128.2, 128.1, 128.0, 127.4, 126.5, 124.9, 124.8, 120.6, 119.9, 114.7, 93.6, 89.6, 21.6; **IR (ATR)**: 3056, 2352, 1425, 1264, 727 cm^{-1}; **HRMS** (ESI, m/z): calcd for. $C_{25}H_{19}BrNO_2S^+$ $(M+H)^+$: 476.0314; Found: 476.0306.

***N*-(2-((1-bromonaphthalen-2-yl)ethynyl)phenyl)-4-methylbenzenesulfonamide (58a)**

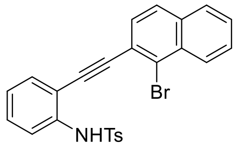

58a

According to general procedure **B**, off-white solid (585 mg, 69% yield); $\mathbf{R_f}$ = 0.65 (PE/EA = 7/1); NMR spectroscopy: **^{1}H NMR** (500 MHz, $CDCl_3$, 25 ºC) δ 8.33 (d, *J* = 7.4 Hz, 1H), 7.98 (s, 1H), 7.84 (d, *J* = 9.5 Hz, 1H), 7.81 (d, *J* = 8.4 Hz, 1H), 7.77 (d, *J* = 8.3 Hz, 2H), 7.71 (d, *J* = 7.3 Hz, 1H), 7.67 (ddd, *J* = 8.4, 6.9, 1.3 Hz, 1H), 7.58 (ddd, *J* = 8.1, 6.9, 1.2 Hz, 1H), 7.53 (d, *J* = 8.5 Hz, 1H), 7.45 (dd, *J* = 7.7, 1.5 Hz, 1H), 7.35 – 7.30 (m, 1H), 7.15 (d, *J* = 8.1 Hz, 2H), 7.06 (td, *J* = 7.6, 1.2 Hz, 1H), 2.31 (s, 3H); **^{13}C NMR** (126 MHz, $CDCl_3$, 25 ºC) δ 144.1, 138.7, 136.3, 134.0, 132.2, 132.0, 130.3, 129.8, 128.4, 128.4, 128.0, 127.9, 127.8, 127.6, 126.7, 124.1, 122.6, 119.0, 113.4, 96.6, 89.5, 21.7; **IR (ATR)**: 3056, 2352, 1425, 1264, 727 cm^{-1}; **HRMS** (ESI, m/z): calcd for. $C_{25}H_{18}BrNO_2SK^+$ $(M+K)^+$: 513.9873; Found: 513.9875.

***N*-(2-((3-bromonaphthalen-2-yl)ethynyl)phenyl)-4-methylbenzenesulfonamide 59a)**

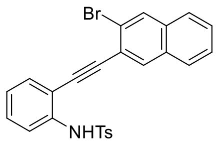

59a

According to general procedure **B**, White solid (546 mg, 64% yield); **Rf** = 0.6 (PE/EA = 7/1); NMR spectroscopy: **^{1}H NMR** (500 MHz, $CDCl_3$, 25 ºC) δ 8.17 (s, 1H), 8.05 (s, 1H), 7.90 (s, 1H), 7.83 – 7.74 (m, 4H), 7.70 (d, *J* = 8.3 Hz, 1H), 7.58 – 7.52 (m, 2H), 7.44 (dd, *J* = 7.7, 1.6 Hz, 1H), 7.33 – 7.29 (m, 1H), 7.16 (d, *J* = 8.0 Hz, 2H), 7.06 (td, *J* = 7.6, 1.1 Hz, 1H), 2.31 (s, 3H); **^{13}C NMR** (126 MHz, $CDCl_3$, 25 ºC) δ 144.1, 138.7, 136.3, 134.0, 133.1, 131.95, 131.85, 131.2, 130.2, 129.7, 128.2, 127.9, 127.6, 127.4, 127.2, 124.1, 121.8, 121.5, 119.1, 113.4, 95.5, 88.5, 21.7; **IR (ATR)**: 3293, 3056, 2352, 1416, 1265, 1162, 897, 727 cm^{-1}; **HRMS** (ESI, m/z): calcd for. $C_{25}H_{18}BrNO_2SK^+$ $(M+K)^+$: 513.9873; Found: 513.9871.

4-methyl-*N*-(2-(phenanthren-9-ylethynyl)phenyl)benzenesulfonamide (60a)

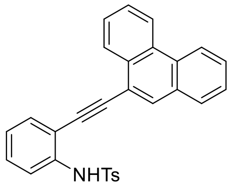

60a

According to general procedure **A**, off-white solid (423 mg, 47% yield); **Rf** = 0.7 (PE/EA = 7/1); 8.73 (dd, *J* = 6.4, 1.8 Hz, 1H), 8.69 (d, *J* = 8.3 Hz, 1H), 8.33 – 8.28 (m, 1H), 8.03 (s, 1H), 7.92 (d, *J* = 9.4 Hz, 1H), 7.77 – 7.68 (m, 6H), 7.68 – 7.64 (m, 1H), 7.54 (dd, *J* = 7.7, 1.5 Hz, 1H), 7.39 – 7.33 (m, 2H), 7.17 – 7.11 (m, 3H), 2.28 (s, 3H); **^{13}C NMR** (126 MHz, $CDCl_3$, 25 ºC) δ 144.2, 137.8, 136.3, 132.6, 132.4, 131.1, 130.7, 130.7, 130.3, 130.0, 129.8, 128.9, 128.1, 127.5, 127.4, 127.4, 127.4, 126.7, 124.9, 123.1, 122.8, 120.7, 118.6, 115.0, 94.6, 88.2, 21.6; **IR (ATR)**: 3321, 3058, 2351, 1490, 1396, 1335, 1160, 905, 731 cm^{-1}; **HRMS** (ESI, m/z): calcd for. $C_{29}H_{21}NO_2SK^+$ $(M+K)^+$: 486.0925; Found: 486.0920.

4-methyl-*N*-(3-(o-tolylethynyl)naphthalen-2-yl)benzenesulfonamide (61a)

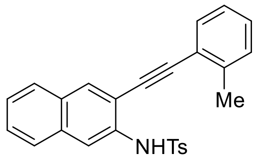

61a

According to general procedure **A**, light red solid (477 mg, 58% yield); **Rf** = 0.6 (PE/EA = 7/1); NMR spectroscopy: **^{1}H NMR** (500 MHz, $CDCl_3$, 25 ºC) δ 8.04 (s, 1H), 7.93 (s, 1H), 7.80 (d, *J* = 8.3 Hz, 1H), 7.71 (dd, *J* = 7.6, 5.7 Hz, 3H), 7.52 – 7.46 (m, 2H), 7.43 – 7.39 (m, 2H), 7.35 – 7.28 (m, 2H), 7.26 – 7.22 (m, 1H), 7.14 (d, *J* = 8.1 Hz, 2H),

2.55 (s, 3H), 2.30 (s, 3H); **^{13}C NMR** (126 MHz, $CDCl_3$, 25 ºC) δ 144.2, 140.2, 136.2, 133.6, 133.6, 132.4, 132.2, 130.4, 130.2, 129.9, 129.8, 129.4, 127.9, 127.7, 127.5, 127.4, 126.0, 122.0, 116.8, 114.4, 95.4, 88.0, 21.6, 21.2; **IR (ATR)**: 3056, 2352, 1264, 1163, 895, 727 cm^{-1}; **HRMS** (ESI, m/z): calcd for. $C_{26}H_{21}NO_2SNa^+$ $(M+Na)^+$: 434.1185; Found: 434.1176.

3 Experimental Procedures and Characterization of Sulfinamides

3.1 Incompatible substrates

Table S1. Unsuccessful substrates

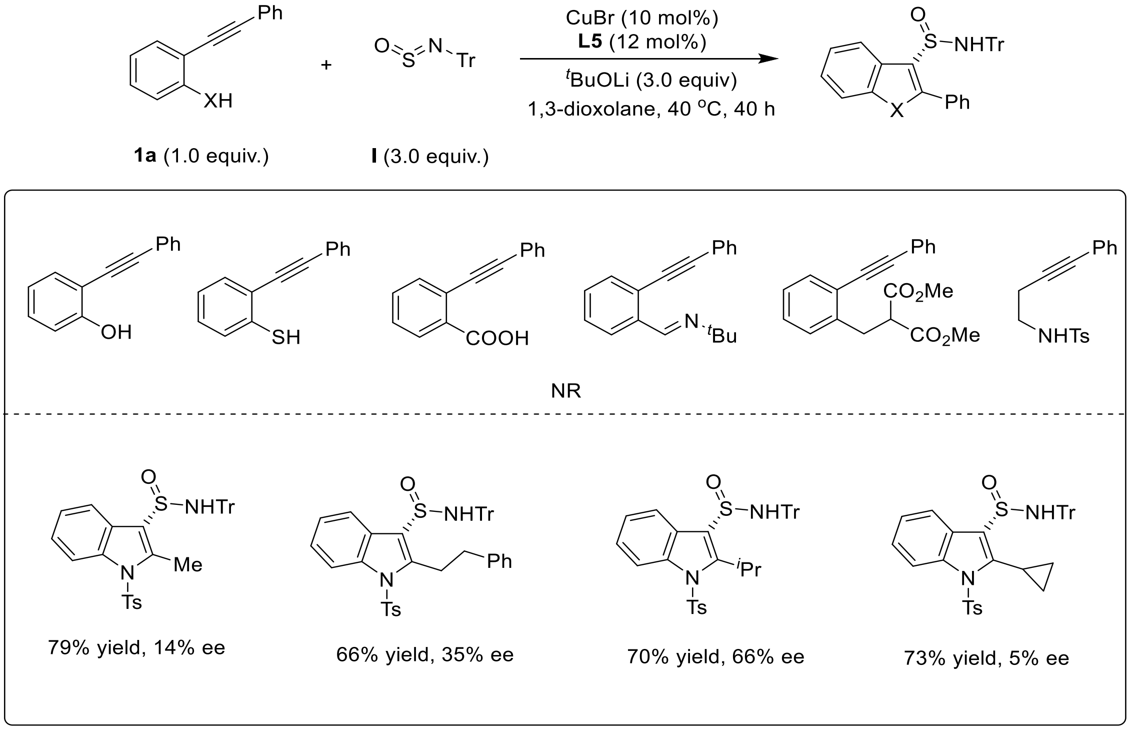

3.2 Optimization of the Reaction Conditions

Table S2. Variation of ligands[a]

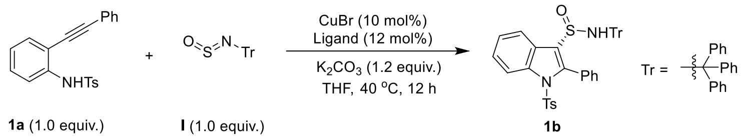

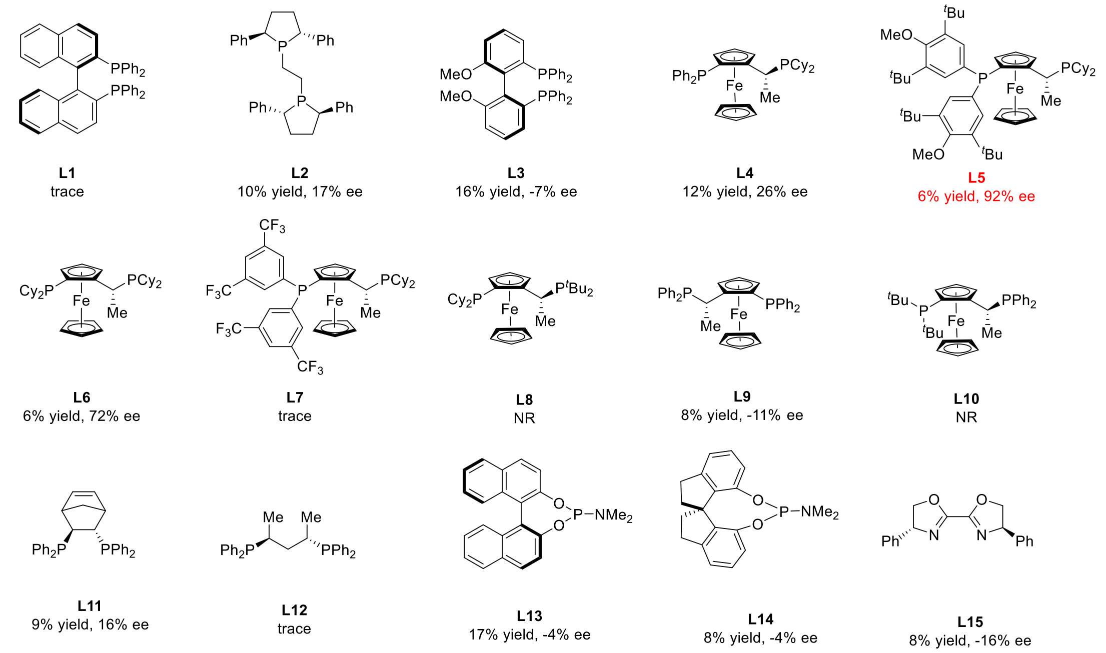

[a]Reaction conditions: CuBr (10.0 mol%), ligand (12.0 mol%), **1a** (1.0 equiv.), sulfinylamine **I** (1.0 equiv.), K_2CO_3 (1.2 equiv.) in THF solvent (0.1 M) at 40 °C under N_2 atmosphere. The yield were determined by ^{1}H NMR with mesitylene as an internal standard. Ee was determined by chiral HPLC.

Table S3. Variation of bases[a]

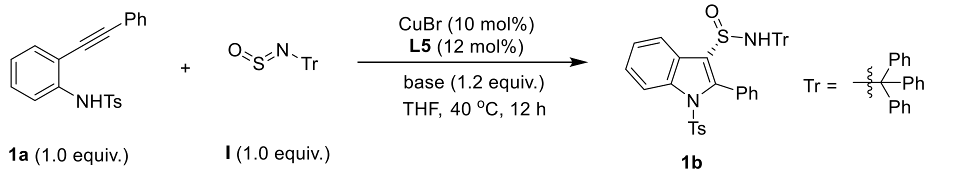

entry	Base (equiv.)	yield	ee
1	Li_2CO_3 (1.2)	trace	/
2	Na_2CO_3 (1.2)	trace	/
3	^{t}BuOLi (1.2)	10%	94%
4	^{t}BuONa (1.2)	6%	82%
5	^{t}BuOK (1.2)	12%	72%
6	LiOH (1.2)	nr	/

7	Li(acac) (1.2)	trace	/
8	LiOMe (1.2)	10%	88%
9	LiF (1.2)	nr	/

[a]Reaction conditions: CuBr (10.0 mol%), **L5** (12.0 mol%), **1a** (1.0 equiv.), sulfinylamine **I** (1.0 equiv.), base (1.2 equiv.) in THF (0.1 M) at 40 ºC under N_2 atmosphere. The yield were determined by ^1H NMR with mesitylene as an internal standard. Ee was determined by chiral HPLC.

Table S4. Variation of solvents[a]

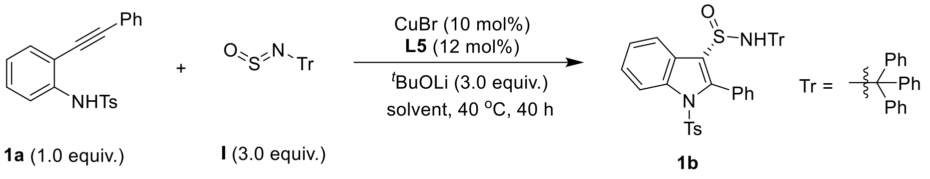

entry	solvent	yield	ee
1	MTBE	14%	80%
2	CH_3CN	8%	89%
3	toluene	10%	48%
4	*n*-hexane	12%	48%
5	EA	trace	/
6	DCM	54%	93%
7	DCE	74%	76%
8	1, 4-dioxane	46%	88%
9	1, 3-dioxolane	89% (84%)[b]	95%
10	DME	16%	93%
11	DMF	nr	/

[a]Reaction conditions: CuBr (10.0 mol%), **L5** (12.0 mol%), **1a** (1.0 equiv.), sulfinylamine **I** (3.0 equiv.), ^{t}BuOLi (3.0 equiv.) in solvent (0.1 M) under N_2 atmosphere. The yield were determined by ^1H NMR with mesitylene as an internal standard. Ee was determined by chiral HPLC.

Table S5. Variation of copper salts[a]

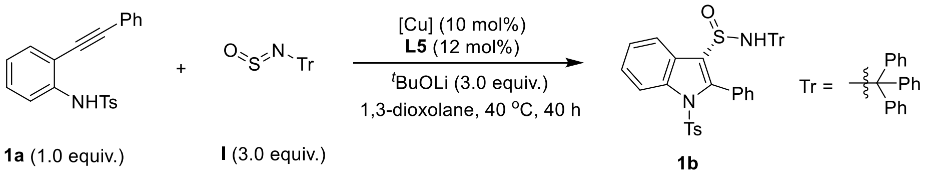

entry	[Cu]	yield	ee
1	CuCl	86%	84%
2	CuI	24%	95%
3	CuTc	80%	79%
4	$Cu(CH_3CN)_4PF_6$	90%	79%
5	CuOAc	92%	86%

6	CuMes	84%	76%
7	$CuBr_2$	82%	88%
8	$Cu(OTf)_2$	trace	/

[a]Reaction conditions: [Cu] (10.0 mol%), **L5** (12.0 mol%), **1a** (1.0 equiv.), sulfinylamine **I** (3.0 equiv.), *t*BuOLi (3.0 equiv.) in 1.3-dioxolane (0.1 M) under N_2 atmosphere. The yield were determined by ^{1}H NMR with mesitylene as an internal standard. Ee was determined by chiral HPLC.

3.3 General Procedure to Synthesize Sulfinamides

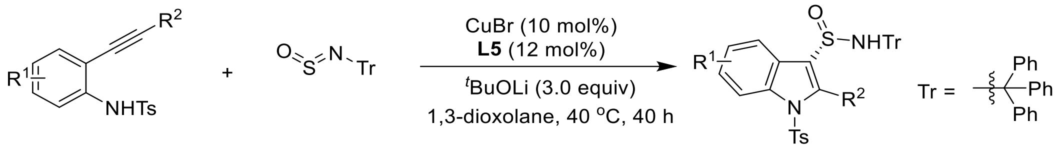

1-61a (1.0 equiv.) **I** (3.0 equiv.) **1-61b**

In a nitrogen-filled glove box, to an oven-dried 8 mL screw cap vial equipped with a magnetic stir bar was added CuBr (1.4 mg, 0.010 mmol, 10 mol%), **L5** (10.5 mg, 0.012 mmol, 12 mol%), **1-35a** (0.1 mmol, 1.0 equiv.), **I** (91.5 mg, 0.3 mmol, 3.0 equiv.), *t*BuOLi (24 mg, 0.3 mmol, 3.0 equiv.) and anhydrous 1,3-dioxolane (1 mL). The tube was sealed with a teflon-lined screw cap, removed from the glove box and the reaction was stirred at 40 °C for 40 hours. Afterwards, the mixture was cooled to room temperature. The solvent was evaporated under reduced pressure. The crude product was added DCM (~1 mL) and purified by column chromatography on silica gel (SiliaFlash® P60, particle size 40-63 µm, 230-400 mesh, Silicycle, PE/EtOAc with 1% Et_3N as eluent) to afford the corresponding product **1-61b**.

The racemate sulfinamides can be synthesized using ***rac*-L4** (***Sp, R*-L4**/***Rp, S*-L4** = 1/1), or synthesized by the following conditions: $[Rh(cod)Cl_2]_2$ (5 mol%), K_2CO_3 (1.2 equiv.), **1-61a** (1.0 equiv.), **I** (1.0 equiv.) in THF (0.1 M) at 40 °C for 12 h under N_2 atmosphere (**Note**: These synthetic methods may result in diastereoselectivity inconsistent with standard conditions).

3.4 Characterization of Sulfinamides

(*S*)-2-Phenyl-1-tosyl-*N*-trityl-1H-indole-3-sulfinamide (1b)

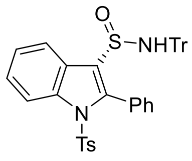

1b

White solid (55.1 mg, 84% yield, 95% ee); **Rf** = 0.4 (PE/EA = 4/1); **$[\alpha]^{25}_D$** = +82.1 (*c* = 1.9, CH_2Cl_2); NMR spectroscopy: **^{1}H NMR** (500 MHz, $CDCl_3$, 25 ºC) δ 8.44 (d, *J* = 8.5 Hz, 1H), 8.37 (d, *J* = 7.9 Hz, 1H), 7.50 (q, *J* = 8.4 Hz, 2H), 7.42 (t, *J* = 7.6 Hz, 1H), 7.37 – 7.21 (m, 15H), 7.14 (d, *J* = 8.1 Hz, 2H), 7.07 (d, *J* = 6.8 Hz, 6H), 5.71 (s, 1H), 2.37 (s, 3H); **^{13}C NMR** (126 MHz, $CDCl_3$, 25 ºC) δ 145.5, 144.1, 138.5, 137.1, 135.2, 129.72, 129.70, 129.6, 129.1, 127.9, 127.6, 127.5, 127.0, 126.0, 125.5, 124.9, 120.7, 116.3, 73.5, 21.7; **IR (ATR)**: 3311, 3059, 2923, 1596, 1445, 1376, 1178, 1075, 772, 572 cm^{-1}; **HRMS** (ESI, m/z): calcd for. $C_{40}H_{32}N_2O_3S_2Na^+$ $(M+Na)^+$: 675.1747; Found: 675.1751; **HPLC** analysis (IA column, *n*-hexane/^{i}PrOH = 85/15, 1.0 mL/min, 25 ºC, 220 nm) indicated 95% ee: t_R (minor) = 17.05 min, t_R (major) = 35.05 min.

(*S*)-2-(*p*-Tolyl)-1-tosyl-*N*-trityl-1H-indole-3-sulfinamide(2b)

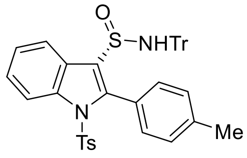

2b

White solid (60.6 mg, 91% yield, 95% ee); **Rf** = 0.5 (PE/EA = 4/1); **$[\alpha]^{25}_D$** = +48.2 (*c* = 2.1, CH_2Cl_2); NMR spectroscopy: **^{1}H NMR** (500 MHz, $CDCl_3$, 25 ºC) δ 8.39 (d, *J* = 8.4 Hz, 1H), 8.30 (d, *J* = 7.8 Hz, 1H), 7.46 (t, *J* = 7.9 Hz, 1H), 7.38 (t, *J* = 7.6 Hz, 1H), 7.34 (d, *J* = 8.3 Hz, 2H), 7.25 – 7.15 (m, 11H), 7.12 (t, *J* = 8.1 Hz, 4H), 7.04 (d, *J* = 8.0 Hz, 6H), 5.65 (s, 1H), 2.45 (s, 3H), 2.34 (s, 3H); **^{13}C NMR** (126 MHz, $CDCl_3$, 25 ºC) δ 145.4, 144.2, 139.8, 138.9, 137.1, 135.2, 129.7, 129.6, 128.4, 127.86, 127.82, 127.4, 127.0, 126.1, 125.9, 125.6, 124.9, 120.6, 116.3, 73.4, 21.7, 21.7; **IR (ATR)**: 3312, 3058, 2923, 1916, 1597, 1495, 1375, 1075, 820 , 573 cm^{-1}; **HRMS** (ESI, m/z): calcd for. $C_{41}H_{34}N_2O_3S_2Na^+$ $(M+Na)^+$: 689.1903; Found: 689.1904; **HPLC** analysis (AD-H column, *n*-hexane/^{i}PrOH = 65/35, 1.0 mL/min, 25 ºC, 300 nm) indicated 95% ee: t_R (minor) = 7.26 min, t_R (major) = 39.95 min.

(*S*)-2-(4-Ethylphenyl)-1-tosyl-*N*-trityl-1H-indole-3-sulfinamide(3b)

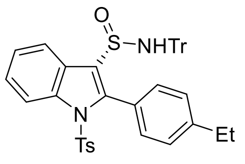

3b

White solid (60.6 mg, 89% yield, 95% ee); **R$_f$** = 0.4 (PE/EA = 4/1); **[α]$^{25}_{D}$** = +94.6 (*c* = 1.9, CH_2Cl_2); NMR spectroscopy: **^{1}H NMR** (500 MHz, $CDCl_3$, 25 ºC) δ 8.43 (d, *J* = 8.5 Hz, 1H), 8.36 (d, *J* = 8.1 Hz, 1H), 7.52 – 7.47 (m, 1H), 7.41 (t, *J* = 7.5 Hz, 1H), 7.36 (d, *J* = 8.3 Hz, 2H), 7.28 – 7.19 (m, 11H), 7.19 – 7.11 (m, 4H), 7.08 (dd, *J* = 6.6, 1.7 Hz, 6H), 5.70 (d, *J* = 2.5 Hz, 1H), 2.77 (q, *J* = 7.6 Hz, 2H), 2.37 (s, 3H), 1.34 (td, *J* = 7.6, 1.3 Hz, 3H); **^{13}C NMR** (126 MHz, $CDCl_3$, 25 ºC) δ 146.0, 145.4, 144.2, 138.9, 137.0, 135.2, 129.7, 129.6, 127.8, 127.6, 127.4, 127.1, 127.0, 126.3, 125.9, 125.7, 124.8, 120.6, 116.2, 28.9, 21.7, 15.5; **IR (ATR)**: 3311, 3057, 2927, 1915, 1597, 1495, 1375, 1177, 836, 571 cm^{-1}; **HRMS** (ESI, m/z): calcd for. $C_{42}H_{36}N_2O_3S_2Na^+$ $(M+Na)^+$: 703.2060; Found: 703.2056; **HPLC** analysis (AD-H column, *n*-hexane/^{i}PrOH = 70/30, 1.0 mL/min, 25 ºC, 300 nm) indicated 95% ee: t_R (minor) = 6.79 min, t_R (major) = 55.80 min.

(*S*)-2-(4-Isopropylphenyl)-1-tosyl-*N*-trityl-1H-indole-3-sulfinamide(4b)

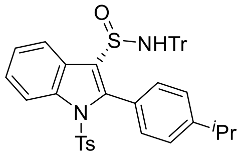

4b

White solid (55.6 mg, 80% yield, 93% ee); **R$_f$** = 0.5 (PE/EA = 4/1); **[α]$^{25}_{D}$** = +50.2 (*c* = 1.85, CH_2Cl_2); NMR spectroscopy: **^{1}H NMR** (500 MHz, $CDCl_3$, 25 ºC) δ 8.40 (d, *J* = 8.5 Hz, 1H), 8.35 (d, *J* = 7.8 Hz, 1H), 7.46 (t, *J* = 7.9 Hz, 1H), 7.38 (t, *J* = 7.6 Hz, 1H), 7.32 (d, *J* = 8.0 Hz, 2H), 7.26 – 7.13 (m, 12H), 7.10 (d, *J* = 8.1 Hz, 3H), 7.03 (d, *J* = 7.4 Hz, 6H), 5.66 (s, 1H), 2.98 (hept, *J* = 6.9 Hz, 1H), 2.34 (s, 3H), 1.31 (s, 3H), 1.30 (s, 3H); **^{13}C NMR** (126 MHz, $CDCl_3$, 25 ºC) δ 150.5, 145.4, 144.2, 138.9, 137.1, 135.2, 129.6, 127.8, 127.5, 127.4, 127.1, 126.3, 125.8, 125.7, 125.6, 124.8, 120.7, 116.2, 34.1, 24.2, 23.9, 21.7; **IR (ATR)**: 3311, 3059, 2961, 1679, 1597, 1377, 1179, 1077, 838, 574 cm^{-1}; **HRMS** (ESI, m/z): calcd for. $C_{43}H_{38}N_2O_3S_2Na^+$ $(M+Na)^+$: 717.2216; Found: 717.2219; **HPLC** analysis (IA column, *n*-hexane/^{i}PrOH = 85/15, 1.0 mL/min, 25 ºC, 254 nm) indicated 93% ee: t_R (minor) = 10.07 min, t_R (major) = 35.04 min,

(*S*)-2-(4-(Tert-butyl)phenyl)-1-tosyl-*N*-trityl-1H-indole-3-sulfinamide (5b)

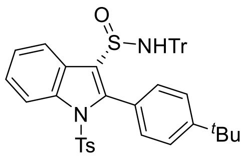

5b

White solid (58.4 mg, 82% yield, 90% ee); $\mathbf{R_f}$ = 0.5 (PE/EA = 4/1); $[\boldsymbol{\alpha}]^{25}_{\mathbf{D}}$ = +28.6 (*c* = 2.05, CH_2Cl_2); NMR spectroscopy: **^{1}H NMR** (500 MHz, $CDCl_3$, 25 ºC) δ 8.39 (d, *J* = 7.6 Hz, 1H), 8.36 (d, *J* = 9.1 Hz, 1H), 7.50 – 7.42 (m, 1H), 7.38 (t, J = 7.1 Hz, 1H), 7.30 (s, 3H), 7.25 – 7.12 (m, 12H), 7.09 (d, J = 8.1 Hz, 2H), 7.02 (d, J = 7.2 Hz, 6H), 5.65 (s, 1H), 2.34 (s, 3H), 1.36 (s, 9H); **^{13}C NMR** (126 MHz, $CDCl_3$, 25 ºC) δ 152.8, 145.4, 144.2, 138.9, 137.1, 135.3, 129.7, 129.7, 129.5, 127.9, 127.8, 127.4, 127.1, 126.0, 125.8, 125.8, 124.8, 124.5, 120.7, 116.2, 73.5, 34.9, 31.5, 21.7; **IR (ATR)**: 3311, 3060, 2960, 1739, 1597, 1375, 1178, 1076, 837, 573 cm^{-1}; **HRMS** (ESI, m/z): calcd for. $C_{44}H_{41}N_2O_3S_2^+$ $(M+H)^+$: 709.2553; Found: 709.2552; **HPLC** analysis(AD-H column, *n*-hexane/^{i}PrOH = 70/30, 1.0 mL/min, 25 ºC, 300 nm) indicated 90% ee: t_R (minor) = 5.83 min, t_R (major) = 26.73 min.

(*S*)-2-([1,1'-Biphenyl]-4-yl)-1-tosyl-*N*-trityl-1H-indole-3-sulfinamide (6b)

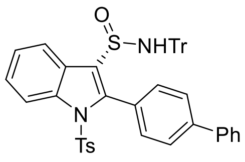

6b

White solid (62 mg, 85% yield, 95% ee); $\mathbf{R_f}$ = 0.4 (PE/EA = 4/1); $[\boldsymbol{\alpha}]^{25}_{\mathbf{D}}$ = +35.8 (*c* = 2.65, CH_2Cl_2); NMR spectroscopy: **^{1}H NMR** (500 MHz, $CDCl_3$, 25 ºC) δ 8.43 (d, *J* = 8.4 Hz, 1H), 8.36 (d, *J* = 7.9 Hz, 1H), 7.66 (d, *J* = 7.6 Hz, 2H), 7.59 – 7.47 (m, 5H), 7.46 – 7.39 (m, 2H), 7.39 – 7.27 (m, 4H), 7.19 (q, *J* = 7.9, 6.3 Hz, 9H), 7.12 (d, *J* = 8.1 Hz, 2H), 7.05 (d, *J* = 6.4 Hz, 6H), 5.71 (s, 1H), 2.35 (s, 3H); **^{13}C NMR** (126 MHz, $CDCl_3$, 25 ºC) δ 145.6, 144.1, 142.5, 140.4, 138.3, 137.2, 135.2, 129.8, 129.6, 129.1, 128.1, 128.0, 127.9, 127.5, 127.3, 127.1, 126.2, 126.1, 125.6, 125.0, 120.7, 116.3, 73.5, 21.8; **IR (ATR)**: 3313, 3058, 2924, 1699, 1597, 1445, 1376, 1178, 762, 572 cm^{-1}; **HRMS** (ESI, m/z): calcd for. $C_{46}H_{36}N_2O_3S_2Na^+$ $(M+Na)^+$: 751.2060; Found: 751.2057; **HPLC** analysis(AD-H column, *n*-hexane/^{i}PrOH = 70/30, 1.0 mL/min, 25 ºC, 220 nm) indicated 95% ee: t_R (minor) = 9.98 min, t_R (major) = 46.80 min,

(*S*)-2-(3,4-Dimethoxyphenyl)-1-tosyl-*N*-trityl-1H-indole-3-sulfinamide (7b)

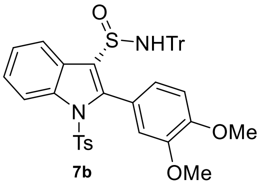

7b

White solid (42.3 mg, 59% yield, 90% ee); **R$_f$** = 0.25 (PE/EA = 4/1); **[α]$^{25}_D$** = +88.3 (*c* = 1.3, CH_2Cl_2); NMR spectroscopy: **^{1}H NMR** (500 MHz, $CDCl_3$, 25 ºC) δ 8.44 (d, *J* = 8.5 Hz, 1H), 8.33 (d, *J* = 7.8 Hz, 1H), 7.49 (t, *J* = 7.9 Hz, 1H), 7.41 (t, *J* = 7.6 Hz, 1H), 7.34 (d, *J* = 8.0 Hz, 2H), 7.27 – 7.17 (m, 9H), 7.12 (d, *J* = 8.1 Hz, 2H), 7.07 (d, *J* = 7.3 Hz, 6H), 6.95 – 6.60 (m, 3H), 5.69 (s, 1H), 3.98 (s, 3H), 3.65 (s, 3H), 2.36 (s, 3H); **^{13}C NMR** (126 MHz, $CDCl_3$, 25 ºC) δ 150.3, 148.0, 145.4, 144.2, 138.5, 137.1, 135.4, 129.6, 129.5, 128.2, 128.03, 127.97, 127.86, 127.5, 127.1, 126.7, 125.9, 124.8, 121.1, 120.6, 116.3, 110.1, 73.4, 56.1, 55.9, 21.7; **IR (ATR)**: 3310, 3057, 2931, 1598, 1504, 1375, 1177, 1075, 758, 571 cm^{-1}; **HRMS** (ESI, m/z): calcd for $C_{42}H_{36}N_2O_5S_2Na^+$ $(M+Na)^+$: 735.1958; Found: 735.1955; **HPLC** analysis (AD column, *n*-hexane/^{i}PrOH = 70/30, 1.0 mL/min, 25 ºC, 220 nm) indicated 90% ee: t_R (minor) = 8.27 min. t_R (major) = 26.47 min.

(*S*)-2-(3,5-Dimethoxyphenyl)-1-tosyl-*N*-trityl-1H-indole-3-sulfinamide (8b)

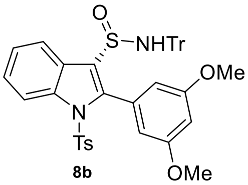

8b

White solid (37.2 mg, 52% yield, 91% ee); **R$_f$** = 0.2 (PE/EA = 4/1); **[α]$^{25}_D$** = +42.9 (*c* = 1.6, CH_2Cl_2); NMR spectroscopy: **^{1}H NMR** (500 MHz, $CDCl_3$, 25 ºC) δ 8.40 (d, *J* = 8.5 Hz, 1H), 8.34 (d, *J* = 7.9 Hz, 1H), 7.47 (t, *J* = 7.9 Hz, 1H), 7.43 – 7.34 (m, 3H), 7.26 – 7.15 (m, 9H), 7.12 (d, *J* = 8.1 Hz, 2H), 7.10 – 6.90 (m, 6H), 6.54 (d, *J* = 2.4 Hz, 1H), 6.46 (s, 1H), 6.30 (s, 1H), 5.67 (s, 1H), 3.68 (s, 3H), 3.57 (s, 3H), 2.34 (s, 3H); **^{13}C NMR** (126 MHz, $CDCl_3$, 25 ºC) δ 160.0, 145.5, 144.2, 138.2, 137.1, 135.3, 130.6, 129.7, 129.6, 127.9, 127.6, 127.5, 127.2, 126.0, 125.5, 124.8, 120.8, 116.2, 102.8, 73.6, 55.5, 21.7; **IR (ATR)**: 3311, 3063, 2929, 1597, 1450, 1377, 1071, 950, 758, 569 cm^{-1}; **HRMS** (ESI, m/z): calcd for. $C_{42}H_{36}N_2O_5S_2Na^+$ $(M+Na)^+$: 735.1958; Found: 735.1953; **HPLC** analysis (AD column, *n*-hexane/^{i}PrOH = 70/30, 1.0 mL/min, 25 ºC, 220 nm) indicated 91% ee: t_R (minor) = 8.72 min ,t_R (major) = 20.76 min.

(S)-2-(4-(dimethylamino)phenyl)-1-tosyl-*N*-trityl-1H-indole-3-sulfinamide (9b)

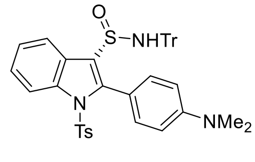

9b

White solid (52.9 mg, 76% yield, 90% ee); **R$_f$** = 0.45 (PE/EA = 2/1); **[α]$^{25}_D$** = +66.6 (*c* = 1.60, CH_2Cl_2); NMR spectroscopy: **^{1}H NMR** (500 MHz, $CDCl_3$, 25 ºC) δ 8.38 (d, *J* = 8.4 Hz, 1H), 8.20 (d, *J* = 7.8 Hz, 1H), 7.42 (t, *J* = 7.9 Hz, 1H), 7.36 – 7.30 (m, 3H), 7.23 – 7.15 (m, 11H), 7.08 (d, *J* = 6.7 Hz, 8H), 6.60 (d, *J* = 8.5 Hz, 2H), 5.68 (s, 1H), 3.05 (s, 6H), 2.32 (s, 3H); **^{13}C NMR** (126 MHz, $CDCl_3$, 25 ºC) δ 151.2, 145.1, 144.4, 140.1, 137.0, 135.2, 132.9, 132.8, 129.6, 129.5, 127.8, 127.3, 127.1, 126.1, 125.4, 124.7, 120.2, 116.5, 115.9, 110.8, 73.2, 40.3, 21.7; **IR (ATR)**: 3053, 2352, 1513, 1373, 1265, 904, 721 cm^{-1}; **HRMS** (ESI, m/z): calcd for. $C_{42}H_{37}N_3O_3S_2Na^+$ $(M+H)^+$: 718.2169; Found: 718.2160; **HPLC** analysis (AD-H column, *n*-hexane/^{i}PrOH = 70/30, 1.0 mL/min, 25 ºC, 220 nm) indicated 90% ee: t_R (minor) = 9.33 min, t_R (major) = 37.64 min.

(*S*)-2-(4-Fluorophenyl)-1-tosyl-*N*-trityl-1H-indole-3-sulfinamide (10b)

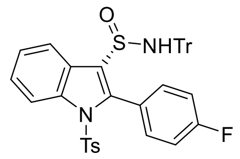

10b

White solid (43 mg, 64% yield, 91% ee); **R$_f$** = 0.5 (PE/EA = 4/1); **[α]$^{25}_D$** = +3.2 (*c* = 0.51, CH_2Cl_2); NMR spectroscopy: **^{1}H NMR** (500 MHz, $CDCl_3$, 25 ºC) δ 8.43 (d, *J* = 8.5 Hz, 1H), 8.38 (d, *J* = 7.8 Hz, 1H), 7.56 – 7.49 (m, 1H), 7.43 (t, *J* = 7.6 Hz, 1H), 7.37 (d, *J* = 8.4 Hz, 2H), 7.29 – 7.21 (m, 10H), 7.21 – 7.14 (m, 3H), 7.09 (d, *J* = 7.4 Hz, 7H), 6.98 (s, 1H), 5.74 (s, 1H), 2.37 (s, 3H); **^{13}C NMR** (126 MHz, $CDCl_3$, 25 ºC) δ 161.8 (d, *J* = 247.1 Hz), 145.8, 144.0, 137.2, 136.8 (d, *J* = 2.4 Hz), 135.0, 131.0 (d, *J* = 8.3 Hz), 129.8, 129.5, 129.2, 129.2, 127.9, 127.6, 127.0, 126.4, 125.3, 125.1, 120.9, 116.7 (d, *J* = 20.8 Hz), 116.3, 73.6, 21.7; **^{19}F NMR** (471 MHz, $CDCl_3$, 25 ºC) δ -113.0 (s, 1F); **IR (ATR)**: 3081, 2932, 1864, 1560, 1463, 1376, 1245, 1007, 831, 571 cm^{-1}; **HRMS** (ESI, m/z): calcd for. $C_{40}H_{32}FN_2O_3S_2^+$ $(M+H)^+$: 671.1833; Found: 671.1839; **HPLC** analysis (AD-H column, *n*-hexane/^{i}PrOH = 70/30, 1.0 mL/min, 25 ºC, 220 nm) indicated 91% ee: t_R (minor) = 6.44 min, t_R (major) = 22.96 min.

(*S*)-2-(3,4-Difluorophenyl)-1-tosyl-*N*-trityl-1H-indole-3-sulfinamide (11b)

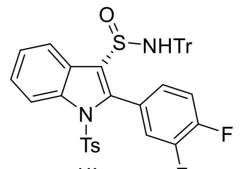

11b

White solid (38.5 mg, 56% yield, 93% ee); **R_f** = 0.5 (PE/EA = 4/1); **$[\alpha]^{25}_D$** = +72.5 (*c* = 1.05, CH_2Cl_2); NMR spectroscopy: **^{1}H NMR** (400 MHz, $CDCl_3$, 25 ºC) δ 8.32 (d, *J* = 8.5 Hz, 1H), 8.26 (d, *J* = 7.9 Hz, 1H), 7.42 (t, *J* = 7.9 Hz, 1H), 7.33 (t, *J* = 7.6 Hz, 1H), 7.25 (d, *J* = 8.1 Hz, 2H), 7.21 – 7.10 (m, 10H), 7.06 (d, *J* = 8.2 Hz, 2H), 7.00 (d, *J* = 5.6 Hz, 7H), 6.93 – 6.87 (m, 1H), 5.63 (s, 1H), 2.27 (s, 3H); **^{13}C NMR** (126 MHz, $CDCl_3$, 25 ºC) δ 151.4 (dd, *J* = 253.3.1, 13.3 Hz), 149.4 (dd, *J* = 250.7, 13.3 Hz), 145.9, 144.0, 137.2, 135.9, 135.0, 129.9, 129.5, 128.0, 127.7, 126.9, 126.6, 125.8 (dd, *J* = 6.3, 1.3 Hz), 125.2, 120.9, 116.7, 116.6, 116.4, 73.6, 21.8; **^{19}F NMR** (376 MHz, $CDCl_3$, 25 ºC) δ -135.1 (s, 1F), -137.4 (s, 1F); **IR (ATR)**: 3309, 3063, 2241, 1600, 1506, 1377, 1177, 1076, 910, 571 cm^{-1}; **HRMS** (ESI, m/z): calcd for. $C_{40}H_{31}F_2N_2O_3S_2^+$ $(M+H)^+$: 689.1739; Found: 689.1733; **HPLC** analysis (AD-H column, *n*-hexane/^{i}PrOH = 70/30, 1.0 mL/min, 25 ºC, 220 nm) indicated 93% ee: t_R (minor) = 7.01 min, t_R (major) = 19.74 min.

(*S*)-2-(4-Chlorophenyl)-1-tosyl-*N*-trityl-1H-indole-3-sulfinamide (12b)

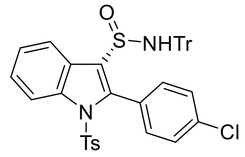

12b

White solid (45.6 mg, 66% yield, 93% ee); **R_f** = 0.5 (PE/EA = 4/1); **$[\alpha]^{25}_D$** = +44.1 (*c* = 0.9, CH_2Cl_2); NMR spectroscopy: **^{1}H NMR** (500 MHz, $CDCl_3$, 25 ºC) δ 8.42 (d, *J* = 8.5 Hz, 1H), 8.36 (d, *J* = 7.9 Hz, 1H), 7.51 (t, *J* = 8.0 Hz, 1H), 7.43 (t, *J* = 7.7 Hz, 1H), 7.34 (d, *J* = 8.0 Hz, 2H), 7.31 – 7.18 (m, 13H), 7.14 (d, *J* = 8.1 Hz, 2H), 7.06 (d, *J* = 7.5 Hz, 6H), 5.69 (s, 1H), 2.37 (s, 3H); **^{13}C NMR** (126 MHz, $CDCl_3$, 25 ºC) δ 145.8, 144.0, 137.2, 137.1, 136.1, 135.1, 129.8, 129.5, 128.4, 128.0, 127.6, 127.5, 127.0, 126.3, 125.4, 125.1, 120.8, 116.3, 73.6, 21.8; **IR (ATR)**: 3310, 3060, 2925, 1699, 1598, 1377, 1178, 1079, 833, 573 cm^{-1}; **HRMS** (ESI, m/z): calcd for. $C_{40}H_{32}ClN_2O_3S_2^+$ $(M+H)^+$: 687.1534; Found: 687.1532; **HPLC** analysis (AD-H column, *n*-hexane/^{i}PrOH = 70/30, 1.0 mL/min, 25 ºC, 220 nm) indicated 93% ee: t_R (minor) = 8.83 min, t_R (major) = 22.70 min.

(*S*)-2-(4-Bromophenyl)-1-tosyl-*N*-trityl-1H-indole-3-sulfinamide (13b)

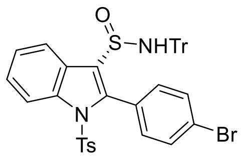

13b

White solid (41.7 mg, 57% yield, 90% ee); **R$_f$** = 0.5 (PE/EA = 4/1); **[α]^{25}$_D$** = +35.0 (*c* = 1.6, CH_2Cl_2); NMR spectroscopy: **^{1}H NMR** (500 MHz, $CDCl_3$, 25 ºC) δ 8.42 (d, *J* = 8.4 Hz, 1H), 8.36 (d, *J* = 7.8 Hz, 1H), 7.51 (ddd, *J* = 8.5, 7.2, 1.3 Hz, 1H), 7.44 (q, *J* = 7.2 Hz, 3H), 7.34 (d, J = 8.4 Hz, 2H), 7.29 – 7.21 (m, 9H), 7.14 (d, *J* = 8.2 Hz, 4H), 7.09 – 7.01 (m, 6H), 5.70 (s, 1H), 2.37 (s, 3H); **^{13}C NMR** (126 MHz, $CDCl_3$, 25 ºC) δ 145.8, 144.0, 137.2, 137.1, 135.0, 130.9, 129.8, 129.5, 128.3, 128.02, 128.97, 127.6, 127.0, 126.4, 125.4, 125.1, 124.4, 120.8, 116.3, 73.6, 21.8; **IR (ATR)**: 3311, 3060, 2854, 1596, 1489, 1377, 1178, 1075, 830, 572 cm^{-1}; **HRMS** (ESI, m/z): calcd for. $C_{40}H_{32}BrN_2O_3S_2^+$ $(M+H)^+$: 731.1032; Found: 731.1027; **HPLC** analysis(AD-H column, *n*-hexane/^{i}PrOH = 70/30, 1.0 mL/min, 25 ºC, 220 nm) indicated 90% ee: t_R (minor) = 9.33 min, t_R (major) = 23.18 min.

(*S*)-1-Tosyl-2-(3-(trifluoromethyl)phenyl)-*N*-trityl-1H-indole-3-sulfinamide (14b)

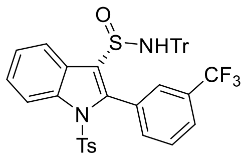

14b

White solid (40.8 mg, 57% yield, 89% ee); **R$_f$** = 0.45 (PE/EA = 4/1); **[α]^{25}$_D$** = +39.1 (*c* = 1.35, CH_2Cl_2); NMR spectroscopy: **^{1}H NMR** (500 MHz, $CDCl_3$, 25 ºC) δ 8.58 – 8.33 (m, 2H), 7.73 (d, *J* = 7.6 Hz, 1H), 7.57 – 7.50 (m, 2H), 7.49 – 7.35 (m, 2H), 7.31 – 7.19 (m, 12H), 7.15 (d, *J* = 8.1 Hz, 2H), 7.06 (s, 6H), 5.73 (s, 1H), 2.38 (s, 3H); **^{13}C NMR** (126 MHz, $CDCl_3$, 25 ºC) δ 146.0, 143.9, 137.3, 136.4, 135.9, 135.2, 134.7, 130.4 (q, *J* = 32.8 Hz), 129.9, 129.9, 129.5, 128.5 (q, *J* = 32.7 Hz), 127.9, 127.8 (q, *J* = 11.1 Hz), 127.6, 126.9, 126.5, 126.4, 125.1, 123.8 (q, *J* = 272.7 Hz). 121.0, 116.2, 73.6, 21.7; **^{19}F NMR** (471 MHz, $CDCl_3$, 25 ºC) δ -62.56 (s, 3F) **IR (ATR)**: 3310, 3065, 2239, 1597, 1445, 1335, 1176, 910, 810, 572 cm^{-1}; **HRMS** (ESI, m/z): calcd for. $C_{41}H_{32}F_3N_2O_3S_2^+$ $(M+H)^+$: 721.1801; Found: 721.1796; **HPLC** analysis (AD-H column, *n*-hexane/^{i}PrOH = 90/10, 0.7 mL/min, 25 ºC, 220 nm) indicated 89% ee: t_R (minor) = 15.61 min, t_R (major) = 25.52 min.

(*S*)-1-Tosyl-2-(4-(trimethylsilyl)phenyl)-*N*-trityl-1H-indole-3-sulfinamide (15b)

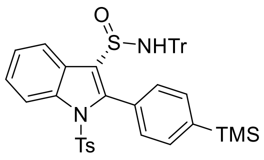

15b

White solid (61.3 mg, 84% yield, 95% ee); **R$_f$** = 0.5 (PE/EA = 4/1); **[α]$^{25}_D$** = +79.84 (*c* = 2.55, CH_2Cl_2); NMR spectroscopy: **^{1}H NMR** (500 MHz, $CDCl_3$, 25 ºC) 8.42 (t, *J* = 8.5 Hz, 2H), 7.50 (t, *J* = 7.9 Hz, 2H), 7.41 (dd, *J* = 23.0, 7.8 Hz, 4H), 7.25 (dt, *J* = 20.2, 8.4 Hz, 11H), 7.14 (d, *J* = 8.1 Hz, 2H), 7.05 (d, *J* = 7.5 Hz, 6H), 5.70 (s, 1H), 2.37 (s, 3H), 0.37 (s, 9H); **^{13}C NMR** (126 MHz, $CDCl_3$, 25 ºC) δ 145.4, 144.1, 142.3, 138.7, 137.0, 135.1, 132.4, 129.7, 129.6, 129.4, 127.8, 127.7, 127.4, 127.1, 125.9, 125.8, 124.8, 120.8, 116.2, 73.5, 21.7; **IR (ATR)**: 3312, 3060, 1916, 1597, 1376, 1177, 1076, 826, 571, 442 cm^{-1}; **HRMS** (ESI, m/z): calcd for. $C_{43}H_{41}N_2O_3S_2Si^+$ $(M+H)^+$: 725.2322; Found: 725.2314; **HPLC** analysis(AD-H column, *n*-hexane/^{i}PrOH = 70/30, 1.0 mL/min, 25 ºC, 220 nm) indicated 95% ee: t_R (minor) = 4.60 min, t_R (major) = 27.58 min.

(*S*)-2-(Benzofuran-5-yl)-1-tosyl-*N*-trityl-1H-indole-3-sulfinamide (16b)

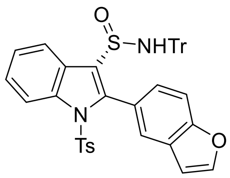

16b

White solid (53.4 mg, 77% yield, 90% ee); **R$_f$** = 0.4 (PE/EA = 4/1); **[α]$^{25}_D$** = +52.4 (*c* = 2.15, CH_2Cl_2); NMR spectroscopy: **^{1}H NMR** (500 MHz, $CDCl_3$, 25 ºC) δ 8.43 (d, *J* = 8.5 Hz, 1H), 8.36 (d, *J* = 7.9 Hz, 1H), 7.72 (s, 1H), 7.58 – 7.35 (m, 4H), 7.31 (d, *J* = 8.0 Hz, 2H), 7.19 (t, *J* = 7.5 Hz, 4H), 7.11 (dt, *J* = 12.7, 7.7 Hz, 8H), 7.00 (s, 6H), 6.85 – 6.48 (m, 1H), 5.69 (s, 1H), 2.34 (s, 3H); **^{13}C NMR** (126 MHz, $CDCl_3$, 25 ºC) δ 155.5, 146.0, 145.5, 144.1, 138.8, 137.1, 135.3, 129.7, 129.5, 128.0, 127.9, 127.8, 127.5, 127.4, 127.1, 126.9, 126.0, 125.5, 124.9, 123.6, 120.7, 116.3, 110.7, 106.9, 73.5, 21.7; **IR (ATR)**: 3312, 3062, 2925, 2239, 1744, 1597, 1376, 1178, 910, 572 cm^{-1}; **HRMS** (ESI, m/z): calcd for. $C_{42}H_{33}N_2O_4S_2^+$ $(M+H)^+$: 693.1876; Found: 693.1870; **HPLC** analysis (AD–H column, *n*-hexane/^{i}PrOH = 70/1, 1.0 mL/min, 25 ºC, 254 nm) indicated 90% ee: t_R (minor) = 13.68 min, t_R (major) = 31.82 min.

(*S*)-1'-methyl-1-tosyl-*N*-trityl-1H,1'H-[2,5'-biindole]-3-sulfinamide (17b)

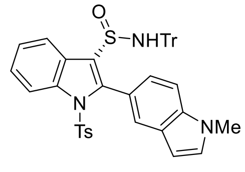

17b

White solid (60.8 mg, 86% yield, 94% ee); $\mathbf{R_f}$ = 0.5 (PE/EA = 2/1); $[\alpha]^{25}_{D}$ = +99.1 (*c* = 1.80, CH_2Cl_2); NMR spectroscopy: **^{1}H NMR** (400 MHz, $CDCl_3$, 25 ºC) δ 8.42 (d, *J* = 8.4 Hz, 1H), 8.38 – 8.21 (m, *J* = 20.2 Hz, 1H), 7.56 – 7.28 (m, 5H), 7.26 – 6.74 (m, 20H), 6.59 – 6.18 (m, 1H), 5.83 – 5.52 (m, 1H), 3.85 (s, 3H), 2.33 (s, 3H); **^{13}C NMR** (126 MHz, $CDCl_3$, 25 ºC) δ 145.2, 144.3, 140.6, 137.2, 137.1, 135.3, 129.60, 129.56, 127.8, 127.6, 127.3, 127.1, 125.9, 125.6, 125.2, 125.0, 124.7, 124.1, 120.5, 119.8, 116.4, 108.4, 102.0, 73.3, 33.1, 21.7; **IR (ATR)**: 3315, 3057, 2925, 2352, 1560, 1446, 1373, 1117, 1072, 907, 721 cm^{-1}; **HRMS** (ESI, m/z): calcd for. $C_{43}H_{35}N_3O_3S_2Na^+$ $(M+Na)^+$: 728.2012; Found: 728.2003; **HPLC** analysis (AD-H column, *n*-hexane/^{i}PrOH = 80/20, 1.0 mL/min, 25 ºC, 230 nm) indicated 94% ee: t_R (minor) = 16.20 min, t_R (major) = 44.30 min

(*S*)-2-(Cyclohex-1-en-1-yl)-1-tosyl-*N*-trityl-1H-indole-3-sulfinamide (18b)

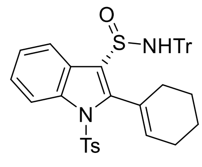

18b

White solid (57.1 mg, 87% yield, 87% ee); $\mathbf{R_f}$ = 0.5 (PE/EA = 4/1); $[\alpha]^{25}_{D}$ = +71.1(*c* = 2.3, CH_2Cl_2); NMR spectroscopy: **^{1}H NMR** (500 MHz, $CDCl_3$, 25 ºC) δ 8.28 (d, *J* = 8.4 Hz, 2H), 7.58 (d, *J* = 8.0 Hz, 2H), 7.40 (t, *J* = 7.8 Hz, 1H), 7.36 – 7.25 (m, 16H), 7.17 (s, 2H), 5.81 (s, 1H), 5.61 (s, 1H), 2.62 (s, 1H), 2.35 (s, 3H), 2.24 – 1.84 (m, 3H), 1.74 (s, 2H), 1.58 – 1.38 (m, 1H), 1.37 – 1.11 (m, 1H); **^{13}C NMR** (126 MHz, $CDCl_3$, 25 ºC) δ 145.4, 144.4, 141.9, 136.8, 135.4, 133.9, 133.0, 130.0, 129.8, 129.7, 127.9, 127.8, 127.6, 127.0, 125.7, 124.7, 120.5, 115.9, 73.5, 30.4, 25.6, 22.5, 21.7, 21.6; **IR (ATR)**: 3312, 3058, 2930, 1680, 1597, 1374, 1175, 1019, 756, 573 cm^{-1}; **HRMS** (ESI, m/z): calcd for. $C_{40}H_{37}N_2O_3S_2^+$ $(M+H)^+$: 657.2240; Found: 657.2235; **HPLC** analysis(AD-H column, *n*-hexane/^{i}PrOH = 70/30, 1.0 mL/min, 25 ºC, 254 nm) indicated 87% ee: t_R (minor) = 5.62 min, t_R (major) = 25.48 min.

(*S*)-2-Cyclohexyl-1-tosyl-*N*-trityl-1H-indole-3-sulfinamide (19b)

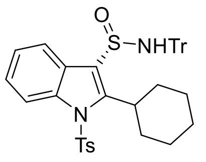

19b

White solid (51.1 mg, 77% yield, 84% ee); **R$_f$** = 0.4 (PE/EA = 4/1); **$[\alpha]^{25}_D$** = +48.6 (*c* = 1.3, CH_2Cl_2); NMR spectroscopy: **^{1}H NMR** (500 MHz, $CDCl_3$, 25 ºC) δ 8.46 (d, *J* = 7.0 Hz, 1H), 8.42 (d, *J* = 8.0 Hz, 1H), 7.68 (d, *J* = 8.5 Hz, 2H), 7.45 – 7.39 (m, 2H), 7.38 – 7.32 (m, 14H), 7.30 – 7.27 (m, 3H), 5.99 (s, 1H), 3.56 (t, *J* = 11.7 Hz, 1H), 2.43 (s, 3H), 1.65 – 1.56 (d, *J* = 20.7 Hz, 4H), 1.37 – 1.29 (m, 2H), 1.24 (q, *J* = 10.1 Hz, 2H), 1.13 (q, *J* = 12.6 Hz, 1H), 0.91 (dd, *J* = 16.8, 8.8 Hz, 1H); **^{13}C NMR** (126 MHz, $CDCl_3$, 25 ºC) δ 145.6, 144.3, 144.1, 137.0, 136.8, 130.2, 129.8, 128.0, 127.7, 126.6, 125.3, 124.7, 124.7, 124.4, 120.3, 115.9, 73.6, 37.0, 33.3, 32.0, 26.9, 26.8, 25.3, 21.8; **IR (ATR)**: 3327, 3059, 2928, 1738, 1596, 1446, 1173, 1071, 948, 566 cm^{-1}; **HRMS** (ESI, m/z): calcd for. $C_{40}H_{39}N_2O_3S_2^+$ $(M+H)^+$: 659.2400; Found: 659.2394; **HPLC** analysis (IA column, *n*-hexane/^{i}PrOH = 80/20, 1.0 mL/min, 25 ºC, 300 nm) indicated 84% ee: t_R (minor) = 12.04 min, t_R (major) = 49.30 min.

(*S*)-5-Methyl-2-phenyl-1-tosyl-N-trityl-1H-indole-3-sulfinamide (20b)

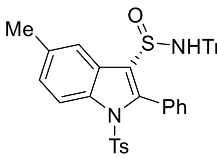

20b

White solid (57.6 mg, 86% yield, 89% ee); **R$_f$** = 0.4 (PE/EA = 4/1); **$[\alpha]^{25}_D$** = +25.9 (*c* = 2.0, CH_2Cl_2); NMR spectroscopy: **^{1}H NMR** (500 MHz, $CDCl_3$, 25 ºC) δ 8.27 (d, *J* = 8.6 Hz, 1H), 8.11 (s, 1H), 7.47 – 7.43 (m, 1H), 7.36 – 7.27 (m, 6H), 7.25 – 7.17 (m, 10H), 7.10 (d, *J* = 8.1 Hz, 2H), 7.06 – 7.01 (m, 6H), 5.69 (s, 1H), 2.47 (s, 3H), 2.34 (s, 3H); **^{13}C NMR** (126 MHz, $CDCl_3$, 25 ºC) δ 145.4, 144.2, 138.6, 135.4, 135.2, 134.8, 129.7, 129.7, 129.6, 129.2, 127.9, 127.7, 127.6, 127.49, 127.47, 127.0, 125.6, 120.4, 116.0, 73.5, 21.7, 21.6; **IR (ATR)**: 3310, 3058, 2855, 1736, 1597, 1375, 1177, 1073, 809, 583 cm^{-1}; **HRMS** (ESI, m/z): calcd for. $C_{41}H_{35}N_2O_3S_2^+$ $(M+H)^+$: 667.2084; Found: 667.2079; **HPLC** analysis (AD-H column, *n*-hexane/^{i}PrOH = 70/30, 1.0 mL/min, 25 ºC, 220 nm) indicated 89% ee: t_R (minor) = 6.62 min, t_R (major) = 37.35 min.

(*S*)-6-Methyl-2-phenyl-1-tosyl-*N*-trityl-1H-indole-3-sulfinamide (21b)

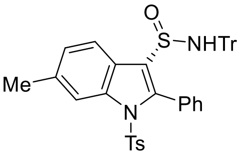

21b

White solid (48.1 mg, 72% yield, 95% ee); **R$_f$** = 0.45 (PE/EA = 4/1); **[α]^{25}$_D$** = +50.3 (*c* = 1.8, CH_2Cl_2); NMR spectroscopy: **^{1}H NMR** (500 MHz, $CDCl_3$, 25 ºC) δ 8.23 (s, 1H), 8.20 (d, *J* = 8.1 Hz, 1H), 7.47 – 7.41 (m, 1H), 7.35 – 7.27 (m, 4H), 7.25 – 7.14 (m, 12H), 7.11 (d, *J* = 8.1 Hz, 2H), 7.03 (d, *J* = 7.0 Hz, 6H), 5.67 (s, 1H), 2.57 (s, 3H), 2.34 (s, 3H); **^{13}C NMR** (126 MHz, $CDCl_3$, 25 ºC) δ 145.4, 144.2, 137.9, 137.6, 136.3, 135.3, 129.7, 129.6, 129.5, 129.2, 127.9, 127.9, 127.6, 127.4, 127.0, 126.4, 123.1, 120.2, 116.3, 73.4, 22.3, 21.7; **IR (ATR)**: 3312, 3059, 2855, 1680, 1598, 1376, 1179, 1077, 814, 578 cm^{-1}; **HRMS** (ESI, m/z): $C_{41}H_{35}N_2O_3S_2^+$ $(M+H)^+$: 667.2084; Found: 667.2081; **HPLC** analysis (AD-H column, *n*-hexane/^{i}PrOH = 70/30, 1.0 mL/min, 25 ºC, 220 nm) indicated 95% ee: t_R (minor) = 7.45 min, t_R (major) = 27.78 min.

(*S*)-5-Methoxy-2-phenyl-1-tosyl-*N*-trityl-1H-indole-3-sulfinamide (22b)

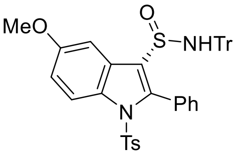

22b

White solid (42.4 mg, 62% yield, 91% ee); **R$_f$** = 0.3 (PE/EA = 4/1); **[α]^{25}$_D$** = +49.0 (*c* = 1.55, CH_2Cl_2); NMR spectroscopy: **^{1}H NMR** (500 MHz, $CDCl_3$, 25 ºC) δ 8.29 (d, *J* = 9.2 Hz, 1H), 7.80 (d, *J* = 2.6 Hz, 1H), 7.48 – 7.43 (m, 1H), 7.35 – 7.18 (m, 16H), 7.11 (d, *J* = 8.2 Hz, 2H), 7.07 – 7.04 (m, 6H), 5.69 (s, 1H), 3.86 (s, 3H), 2.36 (s, 3H). **^{13}C NMR** (126 MHz, $CDCl_3$, 25 ºC) δ 157.2, 145.4, 144.1, 139.2, 135.0, 131.6, 129.7, 129.7, 129.7, 129.1, 127.9, 127.6, 127.5, 127.0, 126.6, 117.4, 115.5, 102.4, 73.3, 55.8, 21.7; **IR (ATR)**: 3308, 3059, 2926, 1735, 1602, 1375, 1172, 1029, 809, 587 cm^{-1}; **HRMS** (ESI, m/z): calcd for. $C_{41}H_{35}N_2O_4S_2^+$ $(M+H)^+$: 683.2033; Found: 683.2026; **HPLC** analysis (AD-H column, *n*-hexane/^{i}PrOH = 70/30, 1.0 mL/min, 25 ºC, 220 nm) indicated 91% ee: t_R (minor) = 8.67 min, t_R (major) = 15.80 min

(*S*)-6-Methoxy-2-phenyl-1-tosyl-*N*-trityl-1H-indole-3-sulfinamide (23b)

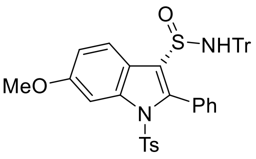

23b

White solid (57.4 mg, 84% yield, 99% ee); **R$_f$** = 0.4 (PE/EA = 3/1); **[α]^{25}$_D$** = +35.8 (*c* = 1.88, CH_2Cl_2); NMR spectroscopy: **^{1}H NMR** (500 MHz, $CDCl_3$, 25 ºC) 8.21 (d, *J* = 8.6 Hz, 1H), 7.95 (s,

1H), 7.43 (t, *J* = 7.4 Hz, 1H), 7.35 – 7.27 (m, 4H), 7.26 – 7.14 (m, 11H), 7.11 (d, *J* = 8.0 Hz, 2H), 7.03 (d, *J* = 7.3 Hz, 7H), 5.64 (s, 1H), 3.95 (s, 3H), 2.35 (s, 3H); **^{13}C NMR** (126 MHz, $CDCl_3$, 25 ºC) δ 158.8, 145.5, 144.2, 138.5, 137.2, 135.2, 129.7, 129.6, 129.5, 129.3, 128.0, 127.9, 127.6, 127.5, 127.0, 121.2, 119.2, 113.9, 100.6, 73.5, 56.0, 21.7; **IR (ATR)**: 3313, 3058, 2839, 1915, 1610, 1486, 1218, 1072, 810, 580 cm^{-1}; **HRMS** (ESI, m/z): calcd for. $C_{41}H_{35}N_2O_4S_2^+$ $(M+H)^+$: 683.2033; Found: 683.2025; **HPLC** analysis (AD-H column, *n*-hexane/^{i}PrOH = 70/30, 1.0 mL/min, 25 ºC, 254 nm) indicated 99% ee: t_R (minor) = 8.41 min, t_R (major) = 28.10 min.

(*S*)-6-Fluoro-2-phenyl-1-tosyl-*N*-trityl-1H-indole-3-sulfinamide (24b)

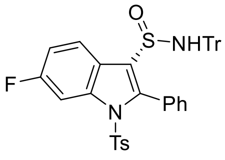

24b

White solid (53.4 mg, 80% yield, 95% ee); **R$_f$** = 0.45 (PE/EA = 4/1); **[α]$^{25}_D$** = +78.8 (*c* = 1.45, CH_2Cl_2); NMR spectroscopy: **^{1}H NMR** (500 MHz, $CDCl_3$, 25 ºC) δ 8.31 (dd, *J* = 8.7, 5.5 Hz, 1H), 8.15 (dd, *J* = 10.3, 2.3 Hz, 1H), 7.49 – 7.43 (m, 1H), 7.33 – 7.27 (m, 4H), 7.25 – 7.16 (m, 11H), 7.16 – 7.11 (m, 3H), 7.04 – 6.98 (m, 6H), 5.61 (s, 1H), 2.36 (s, 3H); **^{13}C NMR** (126 MHz, $CDCl_3$, 25 ºC) δ 161.5 (d, *J* = 243.8 Hz), 145.8, 144.0, 138.8 (d, *J* = 3.8 Hz), 137.4 (d, *J* = 12.6 Hz), 135.0, 129.8, 129.8, 129.5, 128.7, 127.9, 127.7, 127.5, 127.3, 127.1, 121.8, 121.8 (d, *J* = 9.5 Hz), 113.3 (d, *J* = 24.1 Hz), 103.6 (d, *J* = 29.1 Hz), 73.5, 21.8; **^{19}F NMR** (471 MHz, $CDCl_3$, 25 ºC) δ -113.7 (s, 1F); **IR (ATR)**: 3314, 3059, 2924, 1699, 1595, 1377, 1179, 1074, 813, 579 cm^{-1}; **HRMS** (ESI, m/z): calcd for. $C_{40}H_{32}FN_2O_3S_2^+$ $(M+H)^+$: 671.1833; Found: 671.1829; **HPLC** analysis (AD-H column, *n*-hexane/^{i}PrOH = 70/30, 1.0 mL/min, 25 ºC, 230 nm) indicated 95% ee: t_R (minor) = 6.01 min, t_R (major) = 26.58 min.

(*S*)-5,6-Difluoro-2-phenyl-1-tosyl-*N*-trityl-1H-indole-3-sulfinamide (25b)

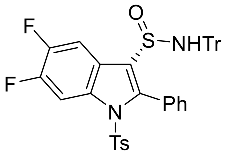

25b

White solid (52.5 mg, 76% yield, 88% ee); **R$_f$** = 0.5 (PE/EA = 4/1); **[α]$^{25}_D$** = +46.23 (*c* = 2.15, CH_2Cl_2); NMR spectroscopy: **^{1}H NMR** (500 MHz, $CDCl_3$, 25 ºC) δ 8.29 (dd, *J* = 11.3, 6.9 Hz, 1H), 8.21 (dd, *J* = 10.2, 7.9 Hz, 1H), 7.52 – 7.45 (m, 1H), 7.34 – 7.27 (m, 7H),

7.25 – 7.19 (m, 8H), 7.16 (d, *J* = 8.4 Hz, 2H), 7.07 – 6.96 (m, 6H), 5.54 (s, 1H), 2.39 (s, 3H); **^{13}C NMR** (126 MHz, $CDCl_3$, 25 ºC) δ 149.6 (dd, *J* = 246.8, 15.0 Hz), 148.6 (dd, *J* = 246.2, 14.4 Hz). 146.0, 143.9, 134.8, 132.3 (dd, *J* = 9.8 Hz), 130.0, 129.9, 129.6, 128.5, 128.3, 128.0, 127.9, 127.7, 127.6, 127.1, 126.7, 121.5 (dd, *J* = 9.0, 1.6 Hz), 108.4 (d, *J* = 21.2 Hz), 105.3 (d, *J* = 24.5 Hz), 73.6, 21.8; **^{19}F NMR** (471 MHz, $CDCl_3$, 25 ºC) δ -136.6 (d, *J* = 21.1 Hz, 1F), -139.1 (d, *J* = 21.0 Hz, 1F). **IR (ATR)**: 3314, 3060, 2924, 1743, 1594, 1378, 1176, 1017, 772, 579 cm^{-1}; **HRMS** (ESI, m/z): calcd for. $C_{40}H_{31}F_2N_2O_3S_2^+$ $(M+H)^+$: 689.1739; Found: 689.1741; **HPLC** analysis (AD–H column, *n*-hexane/^{i}PrOH = 70/1, 1.0 mL/min, 25 ºC, 220 nm) indicated 88% ee: t_R (minor) = 5.01 min, t_R (major) = 6.62 min.

(*S*)-6-Chloro-2-phenyl-1-tosyl-*N*-trityl-1H-indole-3-sulfinamide (26b)

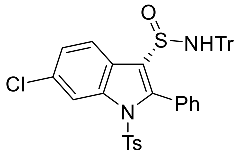

26b

White solid (55.8 mg, 81% yield, 94% ee); **R_f** = 0.45 (PE/EA = 4/1); **$[\alpha]^{25}_D$** = +14.67 (*c* = 1.05, CH_2Cl_2); NMR spectroscopy: **^{1}H NMR** (500 MHz, $CDCl_3$, 25 ºC) δ 8.46 (s, 1H), 8.29 (d, *J* = 8.4 Hz, 1H), 7.50 – 7.45 (m, 1H), 7.39 (d, *J* = 8.5 Hz, 1H), 7.33 – 7.28 (m, 5H), 7.27 – 7.18 (m, 10H), 7.15 (d, *J* = 8.0 Hz, 2H), 7.02 (d, *J* = 7.6 Hz, 6H), 5.58 (s, 1H), 2.38 (s, 3H); **^{13}C NMR** (126 MHz, $CDCl_3$, 25 ºC) δ 145.9, 144.0, 139.0, 137.5, 135.1, 132.1, 129.91, 129.89, 129.6, 128.6, 127.9, 127.7, 127.5, 127.4, 127.2, 125.5, 124.1, 121.6, 116.3, 73.6, 21.8; **IR (ATR)**: 3315, 3060, 2854, 1680, 1491, 1379, 1178, 959, 777 , 576 cm^{-1}; **HRMS** (ESI, m/z): calcd for. $C_{40}H_{32}ClN_2O_3S_2^+$ $(M+H)^+$: 687.1537; Found: 687.1531; **HPLC** analysis (AD-H column, *n*-hexane/^{i}PrOH = 75/25, 1.0 mL/min, 25 ºC, 254 nm) indicated 94% ee: t_R (minor) = 7.44 min, t_R (major) = 23.79 min.

(*S*)-6-Bromo-2-phenyl-1-tosyl-*N*-trityl-1H-indole-3-sulfinamide (27b)

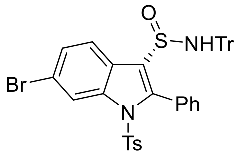

27b

White solid (56.2 mg, 77% yield, 96% ee); **R_f** = 0.5 (PE/EA = 4/1); **$[\alpha]^{25}_D$** = +24.5 (*c* = 2.0, CH_2Cl_2); NMR spectroscopy: **^{1}H NMR** (500 MHz, $CDCl_3$, 25 ºC) δ 8.61 (s, 1H), 8.23 (d, *J* = 8.5 Hz, 1H),

7.50 (s, 1H), 7.45 (d, *J* = 7.7 Hz, 1H), 7.31 – 7.17 (m, 15H), 7.13 (d, *J* = 8.1 Hz, 2H), 7.00 (d, *J* = 7.5 Hz, 6H), 5.57 (s, 1H), 2.36 (s, 3H); **^{13}C NMR** (126 MHz, $CDCl_3$, 25 ºC) δ 145.9, 144.0, 138.9, 137.7, 135.0, 129.9, 129.9, 129.7 129.5, 128.5, 128.2, 127.9, 127.7, 127.5, 127.4, 127.1, 124.5, 121.9, 119.8, 119.1, 73.5, 21.8; **IR (ATR)**: 3311, 3058, 2925, 1744, 1595, 1377, 1177, 1016, 775, 574 cm^{-1}; **HRMS** (ESI, m/z): calcd for. $C_{40}H_{32}BrN_2O_3S_2^+$ $(M+H)^+$: 731.1032; Found: 731.1027; **HPLC** analysis (AD-H column, *n*-hexane/^{i}PrOH = 70/30, 1.0 mL/min, 25 ºC, 220 nm) indicated 96% ee: t_R (minor) = 6.63 min, t_R (major) = 18.03 min.

(*S*)-5-Bromo-2-phenyl-1-tosyl-*N*-trityl-1H-indole-3-sulfinamide (28b)

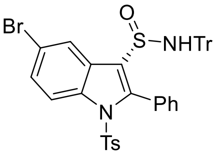

28b

White solid (59.8 mg, 82% yield, 95% ee); **R_f** = 0.4 (PE/EA = 4/1); **$[\alpha]^{25}_D$** = +36.4 (*c* = 1.9, CH_2Cl_2); NMR spectroscopy: **^{1}H NMR** (500 MHz, $CDCl_3$, 25 ºC) δ 8.51 (d, *J* = 2.1 Hz, 1H), 8.28 (d, *J* = 9.0 Hz, 1H), 7.56 (dd, *J* = 9.0, 2.0 Hz, 1H), 7.49 – 7.45 (m, 1H), 7.28 (dd, *J* = 7.4, 5.4 Hz, 4H), 7.26 – 7.19 (m, 11H), 7.12 (d, *J* = 8.1 Hz, 2H), 7.01 (dt, *J* = 6.7, 1.6 Hz, 6H), 5.58 (s, 1H), 2.36 (s, 3H); **^{13}C NMR** (126 MHz, $CDCl_3$, 25 ºC) δ 145.8, 144.0, 139.7, 135.7, 134.9, 129.9, 129.8, 129.6, 129.0, 128.6, 128.1, 127.9, 127.7, 127.5, 127.3, 127.0, 126.9, 123.5, 118.4, 117.5, 73.6, 21.8; **IR (ATR)**: 3311, 3059, 2854, 1744, 1595, 1378, 1179, 1017, 774, 578 cm^{-1}; **HRMS** (ESI, m/z): calcd for. $C_{40}H_{32}BrN_2O_3S_2^+$ $(M+H)^+$: 731.1032; Found: 731.1029; **HPLC** analysis (AD-H column, *n*-hexane/^{i}PrOH = 70/30, 1.0 mL/min, 25 ºC, 230 nm) indicated 95% ee: t_R (minor) = 6.89 min, t_R (major) = 14.69 min.

(*S*)-2-Phenyl-1-tosyl-6-(trifluoromethyl)-*N*-trityl-1H-indole-3-sulfinamide (29b)

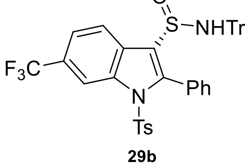

29b

White solid (54.2 mg, 75% yield, 93% ee); **R_f** = 0.4 (PE/EA = 4/1); **$[\alpha]^{25}_D$** = +18.8 (*c* = 1.0, CH_2Cl_2); NMR spectroscopy: **^{1}H NMR** (500 MHz, $CDCl_3$, 25 ºC) δ 8.73 (s, 1H), 8.50 (d, *J* = 8.4 Hz, 1H), 7.66 (d, *J* = 8.4 Hz, 1H), 7.53 – 7.48 (m, 1H), 7.33 – 7.19 (m, 15H), 7.15 (d, *J* = 8.0 Hz, 2H), 7.02 (d, *J* = 7.5 Hz, 6H), 5.58 (s, 1H), 2.38 (s, 3H); **^{13}C**

NMR (126 MHz, $CDCl_3$, 25 ºC) δ 146.1, 144.0, 140.9, 136.3, 134.9, 130.1, 129.9, 129.6, 128.4, 128.2, 128.0 (q, J = 32.4 Hz) 127.9, 127.8, 127.7, 127.6, 127.3, 127.2, 124.5 (q, J = 270.7 Hz) 121.5 (q, J = 3.5 Hz), 121.4, 113.6 (q, J = 4.6 Hz), 73.6, 21.8; **^{19}F NMR** (471 MHz, $CDCl_3$, 25 ºC) δ –61.24 (s, 3F); **IR (ATR)**: 3065, 2926, 1492, 1381, 1327, 1175, 1077, 913, 833, 573 cm^{-1}; **HRMS** (ESI, m/z): calcd for. $C_{41}H_{32}F_3N_2O_3S_2^+$ $(M+H)^+$:721.1801; Found: 721.1799; **HPLC** analysis (AD-H column, n-hexane/^{i}PrOH = 85/15, 1.0 mL/min, 25 ºC, 300 nm) indicated 93% ee: t_R (minor) = 8.90 min, t_R (major) = 19.17 min.

Methyl (*S*)-2-phenyl-1-tosyl-3-((tritylamino)sulfinyl)-1H-indole-6-carboxylate (30b)

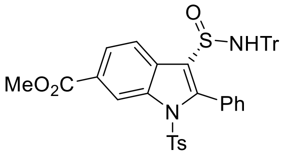

30b

White solid (31.1 mg, 44% yield, 96% ee); **R_f** = 0.3 (PE/EA = 2/1); **$[\alpha]^{25}_D$** = +34.3 (c = 0.6, CH_2Cl_2); NMR spectroscopy: **^{1}H NMR** (500 MHz, $CDCl_3$, 25 ºC) δ 9.00 (s, 1H), 8.44 (d, J = 8.9 Hz, 1H), 8.16 (dd, J = 8.9, 1.7 Hz, 1H), 7.49 – 7.44 (m, 1H), 7.30 (d, J = 8.1 Hz, 4H), 7.21 (q, J = 6.9, 6.3 Hz, 11H), 7.11 (d, J = 8.1 Hz, 2H), 7.04 (d, J = 7.7 Hz, 6H), 5.63 (s, 1H), 3.95 (s, 3H), 2.35 (s, 3H); **^{13}C NMR** (126 MHz, $CDCl_3$, 25 ºC) δ 166.9, 145.9, 144.1, 139.8, 139.6, 135.0, 130.0, 129.9, 129.6, 128.6, 128.3, 128.1, 128.0, 127.7, 127.5, 127.2, 127.1, 126.9, 125.4, 122.8, 116.0, 73.5, 52.4, 21.8; **IR (ATR)**: 3286, 3060, 2853, 1714, 1600, 1441, 1299, 1181, 773, 581 cm^{-1}; **HRMS** (ESI, m/z): calcd for. $C_{42}H_{35}N_2O_5S_2^+$ $(M+H)^+$: 711.1982; Found: 711.1976; **HPLC** analysis (IC column, n-hexane/^{i}PrOH = 70/30, 1.0 mL/min, 25 ºC, 220 nm) indicated 96% ee: t_R (minor) = 18.66 min, t_R (major) = 21.27 min.

(*S*)-2-Phenyl-1-tosyl-*N*-trityl-1H-benzo[f]indole-3-sulfinamide (31b)

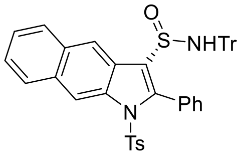

31b

White solid (51.2 mg, 73% yield, 95% ee); **R_f** = 0.4 (PE/EA = 4/1); **$[\alpha]^{25}_D$** = +2.07 (c = 1.55, CH_2Cl_2); NMR spectroscopy: **^{1}H NMR** (500 MHz, $CDCl_3$, 25 ºC) δ 8.88 (s, 1H), 8.80 (s, 1H), 8.08 (d, J = 8.0 Hz, 1H), 7.98 (d, J = 8.1 Hz, 1H), 7.59 – 7.46 (m, 3H), 7.40 –

7.30 (m, 6H), 7.22 (q, *J* = 7.7, 7.0 Hz, 9H), 7.06 (d, *J* = 7.5 Hz, 8H), 5.83 (s, 1H), 2.30 (s, 3H); **^{13}C NMR** (126 MHz, $CDCl_3$, 25 ºC) δ 145.5, 144.1, 141.6, 136.3, 134.8, 131.9, 131.1, 130.0, 129.7, 129.6, 129.2, 128.7, 128.5, 128.1, 127.9, 127.7, 127.5, 127.0, 126.0, 125.7, 125.3, 119.1, 114.1, 73.5, 21.7; **IR (ATR)**: 3309, 3057, 2925, 1699, 1558, 1374, 1179, 1075, 879, 583 cm^{-1}; **HRMS** (ESI, m/z): calcd for. $C_{44}H_{35}N_2O_3S_2^+$ (M+H)$^+$: 703.2084; Found: 703.2077; **HPLC** (AD-H column, *n*-hexane/^{i}PrOH = 85/15, 1.0 mL/min, 25 ºC, 300 nm) indicated 95% ee: t_R (minor) = 17.88 min, t_R (major) = 36.07 min.

(*Ra*, *S*)-2-(o-Tolyl)-1-tosyl-*N*-trityl-1H-indole-3-sulfinamide (32b)

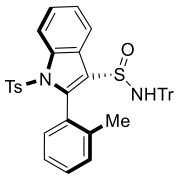

32b

White solid (54.4 mg, 82% yield, 12/1 dr, 99% ee; **R$_f$** = 0.5 (PE/EA = 4/1); **[α]$^{25}_D$** = +132.7 (*c* = 1.2, CH_2Cl_2); NMR spectroscopy: **^{1}H NMR** (500 MHz, $CDCl_3$, 25 ºC) δ 8.44 (d, *J* = 8.5 Hz, 1H), 8.35 (d, *J* = 7.9 Hz, 1H), 7.49 (t, *J* = 7.9 Hz, 1H), 7.40 (d, *J* = 8.4 Hz, 3H), 7.38 – 7.34 (m, 1H), 7.26 – 7.14 (m, 12H), 7.02 (d, *J* = 8.6 Hz, 6H), 6.98 – 6.91 (m, 2H), 5.66 (s, 1H), 2.38 (s, 3H), 1.92 (s, 3H). **^{13}C NMR** (126 MHz, $CDCl_3$, 25 ºC) δ 145.5, 144.2, 139.2, 137.3, 136.8, 135.8, 132.6, 130.0, 129.9, 129.7, 129.7, 128.6, 127.8, 127.5, 127.3, 126.9, 125.8, 125.3, 124.7, 124.5, 120.8, 115.6, 73.6, 21.8, 20.2.; **IR (ATR)**: 3321, 3059, 1915, 1743, 1597, 1445, 1178, 1073, 698, 570 cm^{-1}; **HRMS** (ESI, m/z): calcd for. $C_{41}H_{35}N_2O_3S_2^+$ (M+H)$^+$: 667.2084; Found: 667.2082; **HPLC** (IA-3 column, *n*-hexane/^{i}PrOH = 80/20, 0.5 mL/min, 25 ºC, 254 nm) indicated 99% ee: t_R (minor) = 19.37 min, t_R (major) = 68.51 min.

(*Ra, S*)-5-methyl-2-(o-tolyl)-1-tosyl-*N*-trityl-1H-indole-3-sulfinamide (33b)

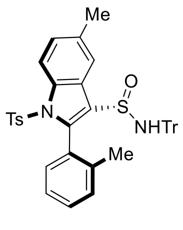

33b

White solid (68.4 mg, 83% yield, > 15/1 dr, 94% ee); **R$_f$** = 0.5 (PE/EA = 4/1); **[α]$^{25}_D$** = +118.4 (*c* = 1.75 CH_2Cl_2); NMR spectroscopy: **^{1}H NMR** (500 MHz, $CDCl_3$, 25 ºC) δ 8.31 (d, *J* = 8.7 Hz, 1H), 8.13 (s, 1H), 7.39 (d, *J* = 8.5 Hz, 2H), 7.35 (ddd, *J* = 7.7, 5.3, 3.6 Hz, 1H), 7.30 (dd, *J* = 8.7, 1.8 Hz, 1H), 7.26 – 7.18 (m, 10H), 7.16 (d, *J* = 8.1 Hz, 2H), 7.03 (dd, *J* = 6.9, 1.7 Hz, 6H), 6.94 (d, *J* = 4.1 Hz, 2H), 5.67 (s, 1H), 2.49 (s, 3H), 2.38

(s, 3H), 1.93 (s, 3H); **^{13}C NMR** (126 MHz, $CDCl_3$) δ 145.4, 144.2, 139.2, 137.3, 135.8, 135.1, 134.3, 132.5, 129.9, 129.9, 129.7, 128.7, 127.8, 127.5, 127.3, 127.3, 126.7, 125.4, 124.7, 120.5, 115.3, 73.6, 21.8, 21.5, 20.2; **IR (ATR)**: 3055, 2352, 1449, 1363, 1179, 1072, 727 cm^{-1}; **HRMS** (ESI, m/z): calcd for. $C_{42}H_{36}N_2O_3S_2Na^+$ $(M+Na)^+$: 703.2060; Found: 703.2060; **HPLC** analysis (IA-3 column, *n*-hexane/^{i}PrOH = 85/15, 0.7 mL/min, 25 ºC, 220 nm) indicated 94% ee: t_R (minor) = 17.10 min, t_R (major) = 53.46 min.

(*Ra, S*)-6-methyl-2-(o-tolyl)-1-tosyl-*N*-trityl-1H-indole-3-sulfinamide (34b)

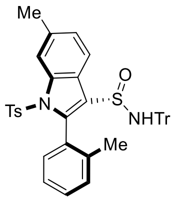

34b

White solid (59.4 mg, 90% yield, > 15/1 dr, 95% ee); **R_f** = 0.5 (PE/EA = 4/1); **$[\alpha]^{25}_D$** = +113.0 (*c* = 1.5, CH_2Cl_2); NMR spectroscopy: **^{1}H NMR** (500 MHz, $CDCl_3$, 25 ºC) δ 8.26 (s, 1H), 8.21 (d, *J* = 8.1 Hz, 1H), 7.39 (d, *J* = 8.4 Hz, 2H), 7.37 – 7.32 (m, 1H), 7.25 – 7.15 (m, 13H), 7.02 (d, *J* = 7.0 Hz, 6H), 6.93 (d, *J* = 3.9 Hz, 2H), 5.64 (s, 1H), 2.58 (s, 3H), 2.39 (s, 3H), 1.90 (s, 3H); **^{13}C NMR** (126 MHz, $CDCl_3$) δ 145.4, 144.2, 139.3, 137.3, 136.5, 136.1, 135.9, 132.6, 129.9, 129.8, 129.7, 129.7, 128.7, 127.8, 127.4, 127.3, 127.0, 126.0, 124.7, 122.9, 120.3, 115.6, 73.6, 22.3, 21.8, 20.2; **IR (ATR)**: 3320, 3054, 2925, 2352, 1599, 1445, 1375, 1264, 1073, 816, 731 cm^{-1}; **HRMS** (ESI, m/z): calcd for. $C_{42}H_{36}N_2O_3S_2Na^+$ $(M+Na)^+$: 703.2060; Found: 703.2052; **HPLC** analysis (IA-3 column, *n*-hexane/^{i}PrOH = 80/20, 1.0 mL/min, 25 ºC, 254 nm) indicated 95% ee: t_R (minor) = 11.44 min, t_R (major) = 27.73 min.

(*Ra, S*)-5,6-dimethyl-2-(o-tolyl)-1-tosyl-*N*-trityl-1H-indole-3-sulfinamide (35b)

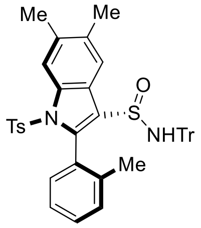

35b

White solid (60 mg, 90% yield, > 15/1 dr, 94% ee); **R_f** = 0.45 (PE/EA = 4/1); **$[\alpha]^{25}_D$** = +120.0 (*c* = 2.05, CH_2Cl_2); NMR spectroscopy: **^{1}H NMR** (500 MHz, $CDCl_3$, 25 ºC) δ 8.23 (s, 1H), 8.09 (s, 1H), 7.39 (d, *J* = 6.5 Hz, 2H), 7.34 (td, *J* = 7.3, 1.8 Hz, 1H), 7.25 – 7.18 (m, 10H), 7.16 (d, *J* = 8.1 Hz, 2H), 7.06 – 7.02 (m, 6H), 6.96 – 6.89 (m, 2H), 5.67 (s, 1H), 2.48 (s, 3H), 2.39 (s, 3H), 2.39 (s, 3H), 1.92 (s, 3H); **^{13}C NMR** (126 MHz, $CDCl_3$) δ 145.3, 144.3, 139.3, 136.4, 135.9, 135.7, 135.2, 133.6, 132.5, 129.8, 129.8,

129.7, 129.7, 128.8, 127.8, 127.4, 127.2, 126.8, 124.6, 123.3, 120.7, 116.0, 73.5, 21.8, 21.1, 20.2, 20.2; **IR (ATR)**: 3320, 3055, 2926, 2352, 1450, 1374, 1176, 1074, 698, 580 cm^{-1}; **HRMS** (ESI, m/z): calcd for. $C_{43}H_{38}N_2O_3S_2Na^+$ (M+Na)$^+$: 717.2216; Found: 717.2213; **HPLC** analysis (IA-3 column, *n*-hexane/^{i}PrOH = 80/20, 1.0 mL/min, 25 ºC, 254 nm) indicated 94% ee: t_R (minor) = 9.99 min, t_R (major) = 17.27 min.

(*Ra, S*)-5-methoxy-2-(o-tolyl)-1-tosyl-*N*-trityl-1H-indole-3-sulfinamide (36b)

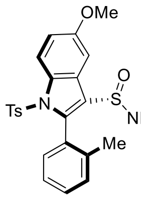

36b

White solid (58.7 mg, 87% yield, > 15/1 dr, 94% ee); **R$_f$** = 0.35 (PE/EA = 4/1); **[α]^{25}$_D$** = +113.1 (*c* = 1.35, CH_2Cl_2); NMR spectroscopy: **^{1}H NMR** (500 MHz, $CDCl_3$, 25 ºC) δ 8.33 (d, *J* = 9.2 Hz, 1H), 7.81 (d, *J* = 2.7 Hz, 1H), 7.42 – 7.35 (m, 3H), 7.28 – 7.16 (m, 12H), 7.10 (dd, *J* = 9.2, 2.7 Hz, 1H), 7.06 (dd, *J* = 7.0, 1.6 Hz, 6H), 6.98 – 6.93 (m, 2H), 5.67 (s, 1H), 3.89 (s, 3H), 2.41 (s, 3H), 1.98 (s, 3H); **^{13}C NMR** (126 MHz, $CDCl_3$, 25 ºC) δ 157.0, 145.4, 144.2, 139.2, 137.8, 135.7, 132.4, 131.3, 130.0, 129.9, 129.7, 128.7, 127.8, 127.5, 127.3, 126.6, 126.3, 124.7, 116.7, 115.4, 102.3, 73.5, 55.8, 21.8, 20.3; **IR (ATR)**: 3314, 3055, 2929, 2351 ,1601, 1452, 1375, 1177, 1075, 1029, 814, 700 cm^{-1}; **HRMS** (ESI, m/z): calcd for. $C_{42}H_{37}N_2O_4S_2^+$ (M+H)$^+$: 697.2189; Found: 697.2189; **HPLC** analysis (IA-3 column, *n*-hexane/^{i}PrOH = 90/10, 0.5 mL/min, 25 ºC, 220 nm) indicated 94% ee: t_R (minor) = 58.74 min, t_R (major) = 63.07 min.

(*Ra, S*)-6-methoxy-2-(o-tolyl)-1-tosyl-*N*-trityl-1H-indole-3-sulfinamide (37b)

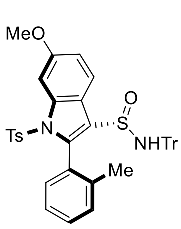

37b

White solid (47.1 mg, 69% yield, 13/1 dr, 95% ee); **R$_f$** = 0.35 (PE/EA = 4/1); **[α]^{25}$_D$** = +116.9 (*c* = 2.1, CH_2Cl_2); NMR spectroscopy: **^{1}H NMR** (500 MHz, $CDCl_3$, 25 ºC) δ 8.22 (d, *J* = 8.8 Hz, 1H), 7.99 (d, *J* = 2.4 Hz, 1H), 7.39 (d, *J* = 8.3 Hz, 2H), 7.34 (td, *J* = 7.4, 2.3 Hz, 1H), 7.24 – 7.16 (m, 12H), 7.05 – 7.00 (m, 7H), 6.97 – 6.91 (m, 2H), 5.63 (s, 1H), 3.96 (s, 3H), 2.39 (s, 3H), 1.92 (s, 3H); **^{13}C NMR** (126 MHz, $CDCl_3$, 25 ºC) δ 158.7, 145.5, 144.2, 139.3, 138.0, 135.9, 135.8, 132.7, 129.9, 129.8, 129.7, 129.7, 128.7, 127.8, 127.4, 127.3, 126.9, 124.7, 121.3, 119.0, 113.6, 100.0, 73.6, 56.0, 21.8, 20.2; **IR (ATR)**:

3313, 3055, 2932, 2352, 1606, 1483, 1373, 1274, 1171, 1073, 699, 577 cm^{-1}; **HRMS** (ESI, m/z): calcd for. $C_{42}H_{36}N_2O_4S_2Na^+$ $(M+Na)^+$: 719.2009; Found: 719.2004; **HPLC** analysis (IA-3 column, *n*-hexane/^{i}PrOH = 85/15, 0.7 mL/min, 25 ºC, 254 nm) indicated 95% ee: t_R (major) = 19.05 min, t_R (major) = 25.60 min.

(*Ra, S*)-6-fluoro-2-(o-tolyl)-1-tosyl-*N*-trityl-1H-indole-3-sulfinamide (38b)

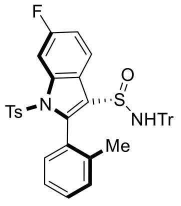

38b

White solid (41.1 mg, 62% yield, 12/1 dr, 93% ee); $\mathbf{R_f}$ = 0.55 (PE/EA = 4/1); $[\alpha]^{25}_D$ = +137.0 (*c* = 1.3, CH_2Cl_2); NMR spectroscopy: **^{1}H NMR** (500 MHz, $CDCl_3$, 25 ºC) δ 8.31 (dd, *J* = 8.7, 5.6 Hz, 1H), 8.22 – 8.13 (m, 1H), 7.39 – 7.32 (m, 3H), 7.25 – 7.14 (m, 13H), 6.99 (d, *J* = 6.9 Hz, 6H), 6.96 – 6.88 (m, 2H), 5.59 (s, 1H), 2.40 (s, 3H), 1.85 (s, 3H); **^{13}C NMR** (126 MHz, $CDCl_3$) δ 161.4 (d, *J* = 243.1 Hz), 145.8, 144.1, 139.2, 137.5 (d, *J* = 3.8 Hz), 137.0 (d, *J* = 12.3 Hz), 135.5, 132.8, 130.1, 129.8, 129.7, 128.2, 127.8, 127.5, 127.4, 126.4, 124.8, 121.9 (d, *J* = 9.5 Hz), 121.7, 113.0 (d, *J* = 24.1 Hz), 103.0 (d, *J* = 29.2 Hz), 73.7, 21.8, 20.1; **^{19}F NMR** (471 MHz, $CDCl_3$, 25 ºC) δ -114.2 (s, 1F); **IR (ATR)**: 3316, 3056, 2927, 2352, 1597, 1481, 1377, 1176, 1076, 820, 700, 577 cm^{-1}; **HRMS** (ESI, m/z): calcd for. $C_{41}H_{33}FN_2O_3S_2Na^+$ $(M+Na)^+$: 707.1809; Found: 707.1807; **HPLC** analysis (IA-3 column, *n*-hexane/^{i}PrOH = 85/15, 0.7 mL/min, 25 ºC, 220 nm) indicated 93% ee: t_R (minor) = 8.34 min, t_R (major) = 24.86 min.

(*Ra, S*)-5,6-difluoro-2-(o-tolyl)-1-tosyl-*N*-trityl-1H-indole-3-sulfinamide (39b)

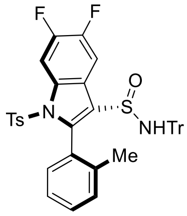

39b

According to the general procedure, react at 40 ºC for 3days. White solid (48.1 mg, 70% yield, 5/1 dr, 95% ee of major; $\mathbf{R_f}$ = 0.6 (PE/EA = 4/1); $[\alpha]^{25}_D$ = +134.2 (*c* = 1.55, CH_2Cl_2); NMR spectroscopy: **^{1}H NMR** (500 MHz, $CDCl_3$, 25 ºC) δ 8.30 (dd, *J* = 11.4, 6.8 Hz, 1H), 8.20 (dd, *J* = 10.2, 8.0 Hz, 1H), 7.34 (dd, *J* = 10.8, 7.9 Hz, 3H), 7.26 – 7.17 (m, 12H), 6.96 (d, *J* = 7.4 Hz, 6H), 6.91 (d, *J* = 6.2 Hz, 1H), 6.85 (t, *J* = 7.5 Hz, 1H), 5.53 (s, 1H), 2.41 (s, 3H), 1.80 (s, 3H); **^{13}C NMR** (126 MHz, $CDCl_3$) δ 149.51 (dd, *J* = 246.3, 15.0 Hz), 148.44 (dd, *J* = 245.5, 14.3 Hz), 146.0, 143.9, 139.1, 138.5 (d, *J* = 4.1 Hz),

135.3, 132.9, 131.9 (d, J = 10.2 Hz), 130.2, 129.9, 129.8, 129.7, 127.9, 127.6, 127.4, 125.8, 124.8, 121.2 (d, J = 9.2 Hz).108.4 (d, J = 21.0 Hz), 104.7 (d, J = 24.8 Hz), 73.8, 21.8, 20.0; **^{19}F NMR** (471 MHz, $CDCl_3$, 25 ºC) δ -137.1 (d, J = 21.0 Hz, 1F), -139.8 (d, J = 21.0 Hz, 1F). **IR (ATR)**: 3317, 3058, 2926, 2352, 1593, 1461, 1375, 1174, 1072, 858, 734, 697 cm^{-1}; **HRMS** (ESI, m/z): calcd for. $C_{41}H_{32}F_2N_2O_3S_2Na^+$ $(M+Na)^+$: 725.1715; Found: 725.1707; **HPLC** analysis (IA-3 column, *n*-hexane/^{i}PrOH = 88/12, 0.5 mL/min, 25 ºC, 254 nm) indicated 95% ee: t_R (major) = 19.77 min, t_R (minor) = 22.12 min.

(*Ra, S*)-6-chloro-2-(o-tolyl)-1-tosyl-*N*-trityl-1H-indole-3-sulfinamide (40b)

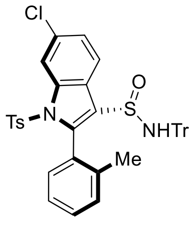

40b

White solid (55.2 mg, 81% yield, > 15/1 dr, 95% ee); **R_f** = 0.55 (PE/EA = 4/1); **$[\alpha]^{25}_D$** = +108.7 (c = 2.1, CH_2Cl_2); NMR spectroscopy: **^{1}H NMR** (500 MHz, $CDCl_3$, 25 ºC) δ 8.48 (d, J = 1.9 Hz, 1H), 8.29 (d, J = 8.5 Hz, 1H), 7.37 (dd, J = 9.4, 7.5 Hz, 4H), 7.25 – 7.16 (m, 12H), 6.98 (d, J = 5.5 Hz, 6H), 6.95 – 6.87 (m, 2H), 5.58 (s, 1H), 2.40 (s, 3H), 1.83 (s, 3H); **^{13}C NMR** (126 MHz, $CDCl_3$) δ 145.9, 144.0, 139.2, 137.8, 137.1, 135.5, 132.8, 131.9, 130.2, 129.9, 129.7, 128.0, 127.8, 127.5, 127.4, 126.4, 125.1, 124.8, 123.9, 121.7, 115.7, 73.7, 21.8, 20.1; **IR (ATR)**: 3317, 3056, 2352, 1596, 1378, 1266, 1179, 1076, 729 cm^{-1}; **HRMS** (ESI, m/z): calcd for. $C_{41}H_{33}ClN_2O_3S_2Na^+$ $(M+Na)^+$: 723.1513; Found: 723.1509; **HPLC** analysis (IA-3 column, *n*-hexane/^{i}PrOH = 80/20, 1.0 mL/min, 25 ºC, 254 nm) indicated 95% ee: t_R (minor) = 8.86 min, t_R (major) = 16.91 min.

(*Ra, S*)-5-chloro-2-(o-tolyl)-1-tosyl-*N*-trityl-1H-indole-3-sulfinamide (41b)

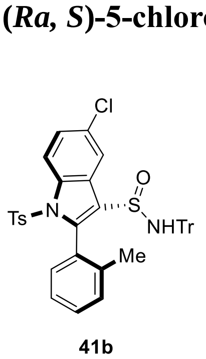

41b

White solid (46.9 mg, 69% yield, 8.5/1 dr, 95% ee); **R_f** = 0.55 (PE/EA = 4/1); **$[\alpha]^{25}_D$** = +48.1 (c = 2.35, CH_2Cl_2); NMR spectroscopy: **^{1}H NMR** (500 MHz, $CDCl_3$, 25 ºC) δ 8.35 (dd, J = 5.6, 3.3 Hz, 2H), 7.43 (dd, J = 9.1, 2.2 Hz, 1H), 7.37 – 7.32 (m, 3H), 7.26 – 7.14 (m, 12H), 6.98 (dd, J = 7.1, 1.6 Hz, 6H), 6.94 – 6.86 (m, 2H), 5.56 (s, 1H), 2.39

(s, 3H), 1.86 (s, 3H); **^{13}C NMR** (126 MHz, $CDCl_3$) δ 145.9, 144.0, 139.1, 138.6, 135.5, 135.0, 132.7, 130.3, 130.2, 129.9, 129.8, 129.7, 128.1, 127.9, 127.5, 127.4, 126.6, 126.1, 124.8, 120.6, 116.6, 73.8, 21.8, 20.1; **IR (ATR)**: 3316, 3057, 2925, 2352, 1595, 1442, 1378, 1179, 1078, 764, 581 cm^{-1}; **HRMS** (ESI, m/z): calcd for. $C_{41}H_{33}ClN_2O_3S_2Na^+$ $(M+Na)^+$: 723.1513; Found: 723.1510; **HPLC** analysis (IA-3 column, *n*-hexane/^{i}PrOH = 85/15, 0.7 mL/min, 25 ºC, 220 nm) indicated 95% ee: t_R (minor) = 15.55 min, t_R (major) = 19.93 min.

(*Ra, S*)-5-bromo-2-(o-tolyl)-1-tosyl-*N*-trityl-1H-indole-3-sulfinamide (42b)

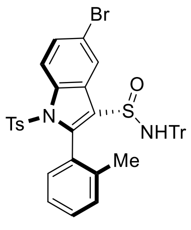

42b

White solid (59.9 mg, 83% yield, 11/1 dr, 94% ee); **R_f** = 0.55 (PE/EA = 4/1); **$[\alpha]^{25}_D$** = +93.1 (*c* = 2.25, CH_2Cl_2); NMR spectroscopy: **^{1}H NMR** (500 MHz, $CDCl_3$, 25 ºC) δ 8.52 (d, *J* = 2.2 Hz, 1H), 8.31 (d, *J* = 9.0 Hz, 1H), 7.57 (dd, *J* = 9.0, 2.0 Hz, 1H), 7.38 – 7.32 (m, 3H), 7.25 – 7.14 (m, 12H), 7.01 – 6.96 (m, 6H), 6.94 – 6.86 (m, 2H), 5.57 (s, 1H), 2.40 (s, 3H), 1.86 (s, 3H); **^{13}C NMR** (126 MHz, $CDCl_3$) δ 145.9, 144.0, 139.1, 138.4, 135.5, 135.4, 132.6, 130.2, 129.9, 129.8, 129.7, 128.8, 128.1, 127.9, 127.5, 127.4, 127.1, 126.0, 124.8, 123.6, 118.0, 116.9, 73.8, 21.8, 20.1; **IR (ATR)**: 3316, 3057, 2352, 1593, 1440, 1378, 1179, 1077, 730 cm^{-1}; **HRMS** (ESI, m/z): calcd for. $C_{41}H_{34}BrN_2O_3S_2^+$ $(M+Na)^+$: 745.1189; Found: 745.1182; **HPLC** analysis (IA-3 column, *n*-hexane/^{i}PrOH = 85/15, 0.7 mL/min, 25 ºC, 254 nm) indicated 94% ee: t_R (minor) = 15.75 min, t_R (major) = 21.47 min.

(*Ra, S*)- 2-(2,4-dimethylphenyl)-1-tosyl-*N*-trityl-1H-indole-3-sulfinamide (43b)

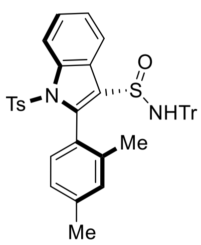

43b

White solid (58.3 mg, 88% yield, 12/1 dr, 95% ee); **R_f** = 0.4 (PE/EA = 4/1); **$[\alpha]^{25}_D$** = +112.4 (*c* = 2.0, CH_2Cl_2); NMR spectroscopy: **^{1}H NMR** (500 MHz, $CDCl_3$, 25 ºC) δ 8.43 (d, *J* = 6.0 Hz, 1H), 8.31 (d, *J* = 10.6 Hz, 1H), 7.48 (t, *J* = 7.2 Hz, 1H), 7.45 – 7.36 (m, 3H), 7.25 – 7.16 (m, 10H), 7.16 (s, 1H), 7.06 – 7.01 (m, 7H), 6.87 (d, *J* = 7.7 Hz, 1H), 6.78 (d, *J* = 7.7 Hz, 1H), 5.64 (s, 1H), 2.41 (s, 3H), 2.38 (s, 3H), 1.91 (s, 3H); **^{13}C**

NMR (126 MHz, $CDCl_3$, 25 ºC) δ 145.5, 144.2, 139.9, 138.9, 137.6, 136.7, 135.8, 132.4, 130.7, 129.7, 129.7, 127.8, 127.4, 127.3, 127.0, 125.7, 125.7, 125.5, 125.4, 124.5, 120.7, 115.6, 73.6, 21.8, 21.6, 20.2; **IR (ATR)**: 3317, 3053, 2924, 2352, 1599, 1445, 1375, 1177, 1074, 743, 698 cm^{-1}; **HRMS** (ESI, m/z): calcd for. $C_{42}H_{36}N_2O_3S_2Na^+$ $(M+Na)^+$: 703.2060; Found: 703.2059; **HPLC** analysis (IA-3 column, *n*-hexane/^{i}PrOH = 80/20, 1.0 mL/min, 25 ºC, 220 nm) indicated 95% ee: t_R (minor) = 9.32 min, t_R (major) = 47.48 min.

(*Ra, S*) -2-(2,5-dimethylphenyl)-1-tosyl-*N*-trityl-1H-indole-3-sulfinamide (44b)

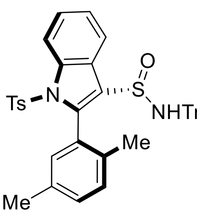

44b

White solid (56.4 mg, 85% yield, 9/1 dr, 93% ee); R_f = 0.4 (PE/EA = 4/1); $[\alpha]^{25}_D$ = +137.3 (*c* = 1.5, CH_2Cl_2); NMR spectroscopy: **^{1}H NMR** (500 MHz, $CDCl_3$, 25 ºC) δ 8.44 (d, *J* = 8.5 Hz, 1H), 8.34 (d, *J* = 7.9 Hz, 1H), 7.48 (t, *J* = 7.9 Hz, 1H), 7.39 (dd, *J* = 12.3, 7.7 Hz, 3H), 7.20 (dt, *J* = 22.6, 7.2 Hz, 10H), 7.14 (d, *J* = 8.1 Hz, 2H), 7.01 (d, *J* = 7.4 Hz, 6H), 6.89 – 6.81 (m, 2H), 5.65 (s, 1H), 2.38 (s, 3H), 2.26 (s, 3H), 1.74 (s, 3H); **^{13}C NMR** (126 MHz, $CDCl_3$, 25 ºC) δ 145.4, 144.2, 137.9, 137.7, 136.8, 136.7, 135.8, 131.4, 130.5, 129.7, 129.6, 128.5, 127.8, 127.5, 127.4, 126.7, 125.7, 125.2, 124.5, 124.4, 120.7, 115.6, 73.6, 21.8, 20.5, 17.3; **IR (ATR)**: 3317, 3055, 2926, 2352, 1595, 1445, 1375, 1177, 1074, 742 cm^{-1}; **HRMS** (ESI, m/z): calcd for. $C_{42}H_{36}N_2O_3S_2Na^+$ $(M+Na)^+$: 703.2060; Found: 703.2055; **HPLC** analysis (IA-3 column, *n*-hexane/^{i}PrOH = 87/13, 0.5 mL/min, 25 ºC, 220 nm) indicated 93% ee: t_R (minor) = 22.57 min, t_R (major) = 53.32 min.

(*Ra, S*) -2-(2-methyl-[1,1'-biphenyl]-3-yl)-1-tosyl-*N*-trityl-1H-indole-3-sulfinamide (45b)

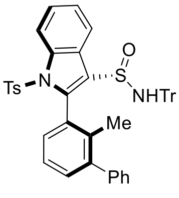

45b

White solid (58.2 mg, 80% yield, >15/1 dr, 92% ee); R_f = 0.4 (PE/EA = 4/1); $[\alpha]^{25}_D$ = +66.1 (*c* = 1.15, CH_2Cl_2); NMR spectroscopy: **^{1}H NMR** (500 MHz, $CDCl_3$, 25 ºC) δ 8.45 (d, *J* = 8.5 Hz, 1H), 8.35 (d, *J* = 7.8 Hz, 1H), 7.51 – 7.47 (m, 1H), 7.45 – 7.39 (m, 5H), 7.38 – 7.32

(m, 2H), 7.26 – 7.17 (m, 11H), 7.13 (d, *J* = 8.1 Hz, 2H), 7.09 – 7.00 (m, 8H), 5.73 (s, 1H), 2.33 (s, 3H), 1.73 (s, 3H); **^{13}C NMR** (126 MHz, $CDCl_3$, 25 ºC) δ 145.6, 144.2, 142.5, 141.5, 137.5, 136.8, 136.7, 135.8, 131.9, 131.5, 129.7, 129.4, 129.2, 128.2, 127.9, 127.5, 127.5, 127.2, 126.8, 125.9, 125.1, 124.7, 124.5, 120.8, 115.7, 73.7, 21.8, 18.7; **IR (ATR)**: 3321, 3056, 2923, 2352, 1444, 1375, 1179, 1076, 708 cm^{-1}; **HRMS** (ESI, m/z): calcd for. $C_{47}H_{38}N_2O_3S_2Na^+$ (M+Na)$^+$: 765.2216; Found: 765.2209; **HPLC** analysis (IA-3 column, *n*-hexane/^{i}PrOH = 85/15, 0.5 mL/min, 25 ºC, 220 nm) indicated 92% ee: t_R (minor) = 8.54 min, t_R (major) = 25.80 min.

(*Ra*, *S*)-2-(3-methoxy-2-methylphenyl)-1-tosyl-*N*-trityl-1H-indole-3-sulfinamide (46b)

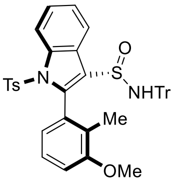

46b

White solid (51.9 mg, 77% yield, 13/1 dr, 95% ee); **R_f** = 0.4 (PE/EA = 4/1); **$[\alpha]^{25}_D$** = +127.4 (*c* = 1.8, CH_2Cl_2); NMR spectroscopy: **^{1}H NMR** (500 MHz, $CDCl_3$, 25 ºC) δ 8.42 (d, *J* = 8.5 Hz, 1H), 8.34 (d, *J* = 8.0 Hz, 1H), 7.50 – 7.45 (m, 1H), 7.43 (d, *J* = 8.4 Hz, 2H), 7.40 – 7.37 (m, 1H), 7.25 – 7.17 (m, 9H), 7.15 (d, *J* = 8.1 Hz, 2H), 7.01 (dd, *J* = 7.0, 1.7 Hz, 6H), 6.94 – 6.89 (m, 2H), 6.65 (dd, *J* = 5.5, 3.2 Hz, 1H), 5.64 (s, 1H), 3.90 (s, 3H), 2.37 (s, 3H), 1.78 (s, 3H); **^{13}C NMR** (126 MHz, $CDCl_3$, 25 ºC) δ 157.5, 145.5, 144.2, 137.2, 136.7, 135.7, 129.7, 128.1, 127.8, 127.8, 127.4, 127.4, 127.3, 126.9, 125.7, 125.4, 125.4, 124.9, 124.5, 120.8, 115.6, 111.2, 73.6, 55.6, 21.8, 13.9; **IR (ATR)**: 3318, 3056, 2932, 2351, 1585, 1446, 1375, 1258, 1176, 1077, 740, 702, 568 cm^{-1}; **HRMS** (ESI, m/z): calcd for. $C_{42}H_{37}N_2O_4S_2^+$ (M+H)$^+$: 697.2189; Found: 697.2180; **HPLC** analysis (IA-3 column, *n*-hexane/^{i}PrOH = 80/20, 1.0 mL/min, 25 ºC, 300 nm) indicated 95% ee: t_R (minor) = 8.47 min, t_R (major) = 78.47 min.

(*Ra*, *S*)-2-(2-methyl-4-(trifluoromethoxy)phenyl)-1-tosyl-*N*-trityl-1H-indole-3-sulfinamide (47b)

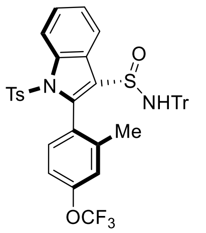

47b

White solid (48.9 mg, 67% yield, > 15/1 dr, 95% ee); **R$_f$** = 0.45 (PE/EA = 4/1); **[α]$^{25}_{D}$** = +98.5 (*c* = 1.6, CH_2Cl_2); NMR spectroscopy: **^{1}H NMR** (500 MHz, $CDCl_3$, 25 ºC) δ 8.44 (d, *J* = 8.5 Hz, 1H), 8.40 (d, *J* = 7.9 Hz, 1H), 7.51 (ddd, *J* = 8.5, 7.3, 1.3 Hz, 1H), 7.45 – 7.40 (m, 1H), 7.37 (d, *J* = 8.4 Hz, 2H), 7.26 – 7.15 (m, 11H), 7.05 (s, 1H), 7.02 (dd, *J* = 7.6, 2.2 Hz, 6H), 6.89 (d, *J* = 8.4 Hz, 1H), 6.71 (d, *J* = 8.3 Hz, 1H), 5.70 (s, 1H), 2.39 (s, 3H), 1.90 (s, 3H); **^{13}C NMR** (126 MHz, $CDCl_3$, 25 ºC) δ 150.3, 145.8, 144.0, 141.9, 136.9, 135.7, 135.6, 134.2, 129.9, 129.7, 127.9, 127.6, 127.3, 127.2, 126.2, 125.1, 124.7, 121.8, 121.0, 120.6 (d, *J* = 257.8 Hz), 116.8, 115.6, 73.8, 21.8, 20.3; **^{19}F NMR** (471 MHz, $CDCl_3$) δ -57.45 (s, 3F); **(ATR)**: 3315, 3057, 2930, 2352, 1598, 1491, 1377, 1251, 1171, 1078, 952, 822, 745, 698 cm^{-1}; **HRMS** (ESI, m/z): calcd for. $C_{42}H_{34}F_3N_2O_4S_2^+$ $(M+H)^+$: 751.1907; Found: 751.1904; **HPLC** analysis (IA-3 column, *n*-hexane/^{i}PrOH = 80/20, 1.0 mL/min, 25 ºC, 254 nm) indicated 95% ee: t_R (minor) = 9.14 min, t_R (major) = 23.49 min.

(*Ra*, *S*) -2-(4-fluoro-2-methylphenyl)-1-tosyl-*N*-trityl-1H-indole-3-sulfinamide (48b)

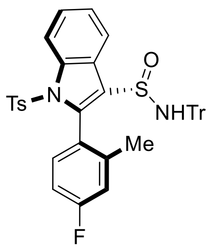

48b

White solid (48.8 mg, 73% yield, > 15/1 dr, 95% ee); **R$_f$** = 0.45 (PE/EA = 4/1); **[α]$^{25}_{D}$** = +145.5 (*c* = 1.5, CH_2Cl_2); NMR spectroscopy: **^{1}H NMR** (500 MHz, $CDCl_3$, 25 ºC) δ 8.44 (d, *J* = 8.5 Hz, 1H), 8.34 (d, *J* = 7.9 Hz, 1H), 7.53 – 7.47 (m, 1H), 7.40 (dd, *J* = 14.4, 7.8 Hz, 3H), 7.26 – 7.18 (m, 10H), 7.17 (s, 1H), 7.04 (d, *J* = 6.9 Hz, 6H), 6.95 – 6.86 (m, 2H), 6.64 (td, *J* = 8.3, 2.7 Hz, 1H), 5.67 (s, 1H), 2.39 (s, 3H), 1.90 (s, 3H); **^{13}C NMR** (126 MHz, $CDCl_3$) δ 163.7 (d, *J* = 249.7 Hz), 145.7, 144.1, 142.2 (d, *J* = 8.5 Hz), 136.9, 136.1, 135.8, 134.4 (d, *J* = 8.7 Hz), 129.8, 129.7, 127.9, 127.6, 127.4, 127.2, 126.0, 125.1, 124.6, 124.5 (d, *J* = 3.3 Hz), 120.8, 116.6 (d, *J* = 21.4 Hz), 115.7, 111.9 (d, *J* = 21.5 Hz), 73.7, 21.8, 20.4; **^{19}F NMR** (471 MHz, $CDCl_3$,) δ -111.3 (s, 1F); **IR (ATR)**: 3314, 3057, 2926, 2352, 1597, 1490, 1445, 1376, 1177, 1076, 746, 698, 569 cm^{-1}; **HRMS** (ESI, m/z): calcd for. $C_{41}H_{33}FN_2O_3S_2Na^+$ $(M+Na)^+$: 707.1809; Found:

707.1807; **HPLC** analysis (IA-3 column, *n*-hexane/^{i}PrOH = 80/20, 1.0 mL/min, 25 ºC, 254 nm) indicated 95% ee: t_R (minor) = 11.20 min, t_R (major) = 24.69 min.

(*Ra, S*) 2-(5-chloro-2-methylphenyl)-1-tosyl-*N*-trityl-1H-indole-3-sulfinamid (49b)

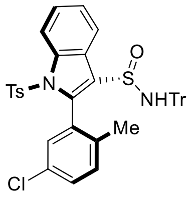

49b

White solid (57.3 mg, 84% yield, > 15/1 dr, 95% ee); **R_f** = 0.45 (PE/EA = 4/1); **[α]^{25}$_D$** = +75.5 (*c* = 2.25, CH_2Cl_2); NMR spectroscopy: **^{1}H NMR** (500 MHz, $CDCl_3$, 25 ºC) δ 8.45 (d, *J* = 8.4 Hz, 1H), 8.36 (d, *J* = 7.9 Hz, 1H), 7.51 (t, *J* = 7.9 Hz, 1H), 7.42 (dd, *J* = 15.4, 7.9 Hz, 3H), 7.33 (dd, *J* = 8.2, 2.3 Hz, 1H), 7.25 – 7.18 (m, 11H), 7.16 (d, *J* = 8.3 Hz, 1H), 7.13 – 7.07 (m, 6H), 6.71 (d, *J* = 2.2 Hz, 1H), 5.73 (s, 1H), 2.41 (s, 3H), 2.02 (s, 3H); **^{13}C NMR** (126 MHz, $CDCl_3$) δ 145.8, 144.1, 138.2, 137.1, 135.7, 135.6, 131.7, 131.2, 130.4, 130.2, 130.0, 129.9, 129.6, 127.9, 127.6, 127.4, 127.2, 126.2, 124.8, 124.8, 120.9, 115.8, 73.7, 21.8, 19.9. **IR (ATR)**: 3314, 3056, 2926, 2352, 1446, 1377, 1179, 1079 ,746, 699, 570 cm^{-1}; **HRMS** (ESI, m/z): calcd for. $C_{41}H_{34}ClN_2O_3S_2^+$ $(M+H)^+$: 701.1694; Found: 701.1686; **HPLC** analysis (IA-3 column, *n*-hexane/^{i}PrOH = 85/15, 1.0 mL/min, 25 ºC, 254 nm) indicated 95% ee: t_R (major) = 13.57 min, t_R (minor) = 14.55 min.

(*Ra, S*)-2-(2-Fluorophenyl)-1-tosyl-*N*-trityl-1H-indole-3-sulfinamide (50b)

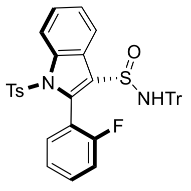

50b

According to the general procedure, react at 40 ºC for 5 days. White solid ((52.8 mg, 81% yield, 1.5/1 dr, 89% ee (major), 83% ee (minor)); **R_f** = 0.5 (PE/EA = 4/1); **[α]^{25}$_D$** = +47.7 (*c* = 0.75, CH_2Cl_2); NMR spectroscopy: **^{1}H NMR** (500 MHz, $CDCl_3$, 25 ºC) δ 8.43 (d, *J* = 7.9 Hz, 0.57H, major), 8.35 (d, *J* = 8.5 Hz, 0.57H, major), 8.32 (d, *J* = 8.4 Hz, 0.43H, minor), 8.23 (d, *J* = 7.9 Hz, 0.43H, minor), 7.51 – 7.34 (m, 5H, major + minor), 7.25 – 6.94 (m, 20H, major + minor), 5.71 (s, 0.43H, minor), 5.68 (s, 0.57H, (major), 2.35 (s, 1.29H, minor), 2.34 (s, 1.71H, major); **^{13}C NMR** (126 MHz, $CDCl_3$, 25 ºC) δ 161.3 (d, *J* = 251.5 Hz, major). 160.4 (d, *J* = 250.0 Hz, minor), 145.7 (major), 145.6 (minor), 144.1 (minor), 144.1 (major), 137.0 (major + minor), 135.3 (major), 135.2 (minor), 134.0

(major + minor), 132.3 (minor), 132.2 (major), 132.1 (minor), 132.1 (major). 132.0 (major), 131.6 (minor), 129.9 (major), 129.8 (minor), 129.7 (minor), 129.6. (major), 127.9 (minor), 127.9 (major), 127.6 (minor), 127.4 (major), 127.1 (minor), 127.1 (major), 126.2 (major)., 126.2 (minor). 125.7 (major), 125.5 (minor), 124.7 (major), 124.7 (minor). 123.5 (d, *J* = 3.6 Hz, major), 123.4 (d, *J* = 3.5 Hz, minor), 121.1(major), 120.8 (minor), 118.0 (d, *J* = 15.7 Hz, major), 117.6 (d, *J* = 16.0 Hz, minor), 115.8 (minor), 115.7 (major), 115.5 (major), 115.4 (minor), 73.6 (major), 73.6 (minor), 21.8 (minor), 21.7 (major); **^{19}F NMR** (471 MHz, $CDCl_3$, 25 ºC) δ -107.02 (s, 0.57F, major), -109.82 (s, 0.43F, minor); **IR (ATR)**: 3314, 3060, 2854, 1700, 1488, 1377, 1178, 1077, 760, 571 cm^{-1}; **HRMS** (ESI, m/z): calcd for. $C_{40}H_{32}FN_2O_3S_2^+$ $(M+H)^+$: 671.1833; Found: 671.1824; **HPLC** (IA-3 column, *n*-hexane/^{i}PrOH = 80/20, 1.0 mL/min, 25 ºC, 22 nm) indicated 89% ee of major: t_R (minor) = 11.41 min, t_R (major) = 28.38 min; 83% ee of major: t_R (major) = 9.66 min, t_R (minor) = 14.36 min.

(*Ra, S*)-2-(2-chlorophenyl)-1-tosyl-*N*-trityl-1H-indole-3-sulfinamide (51b)

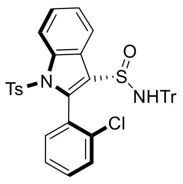

51b

According to the general procedure, react at 40 ºC for 5 days.White solid (56.1 mg, 84% yield, 14/1 dr, 94% ee); **R_f** = 0.5 (PE/EA = 4/1); **$[\alpha]^{25}_D$** = +92.2 (*c* = 1.0, CH_2Cl_2); NMR spectroscopy: **^{1}H NMR** (500 MHz, $CDCl_3$, 25 ºC) δ 8.35 (d, *J* = 8.5 Hz, 1H), 8.22 (d, *J* = 7.9 Hz, 1H), 7.54 – 7.33 (m, 6H), 7.20 (ddd, *J* = 21.8, 13.1, 7.6 Hz, 12H), 7.14 – 7.09 (m, 1H), 7.04 (d, *J* = 7.5 Hz, 6H), 5.73 (s, 1H), 2.36 (s, 3H); **^{13}C NMR** (126 MHz, $CDCl_3$) δ 145.7, 144.1, 136.5, 135.4, 135.3, 134.5, 134.4, 131.1, 129.8, 129.7, 129.3, 128.7, 127.9, 127.5, 127.4, 126.1, 125.8, 125.3, 124.5, 120.9, 115.4, 73.6, 21.8; **IR (ATR)**: 3316, 3056, 2325, 2352, 1443, 1377, 1263, 1178, 1076, 729 cm^{-1}; **HRMS** (ESI, m/z): calcd for. $C_{40}H_{32}ClN_2O_3S_2^+$ $(M+H)^+$: 687.1537; Found: 687.1530; **HPLC** analysis (IC column, *n*-hexane/^{i}PrOH = 85/15, 1.0 mL/min, 25 ºC, 220 nm) indicated 94% ee: t_R (minor) = 17.78 min, t_R (major) = 19.44 min.

(*Ra, S*)-2-(2-bromophenyl)-1-tosyl-*N*-trityl-1H-indole-3-sulfinamide (52b)

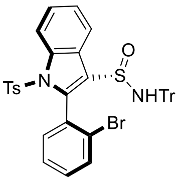

52b

According to the general procedure, react at 40 ºC for 5 days. White solid (56.3 mg, 79% yield, > 15/1 dr, 94% ee); **R$_f$** = 0.55 (PE/EA = 4/1); **$[\alpha]^{25}_D$** = +85.3 (*c* = 1.05, CH_2Cl_2); NMR spectroscopy: **^{1}H NMR** (500 MHz, $CDCl_3$, 25 ºC) δ 8.35 (d, *J* = 8.5 Hz, 1H), 8.21 (d, *J* = 7.9 Hz, 1H), 7.62 (d, *J* = 8.1 Hz, 1H), 7.57 – 7.43 (m, 3H), 7.35 (dt, *J* = 18.7, 7.7 Hz, 2H), 7.26 – 7.11 (m, 13H), 7.04 (d, *J* = 7.4 Hz, 6H), 5.76 (s, 1H), 2.36 (s, 3H); **^{13}C NMR** (126 MHz, $CDCl_3$, 25 ºC) δ 145.7, 144.1, 136.3, 136.2, 135.5, 134.4, 132.4, 131.2, 130.8, 129.9, 129.7, 127.9, 127.6, 127.5, 127.0, 126.4, 126.1, 125.8, 125.3, 124.5, 121.0, 115.3, 73.6, 21.8; **IR (ATR)**: 3313, 3057, 2924, 2352, 1443, 1376, 1178, 1079, 752, 701 cm^{-1}; **HRMS** (ESI, m/z): calcd for. $C_{40}H_{32}BrN_2O_3S_2^+$ $(M+H)^+$: 731.1032; Found: 731.1027; **HPLC** analysis (IC column, *n*-hexane/^{i}PrOH = 85/15, 1.0 mL/min, 25 ºC, 254 nm) indicated 94% ee: t_R (minor) = 18.73 min, t_R (major) = 21.43 min.

(*Ra*, *S*)-2-(2-bromo-5-chlorophenyl)-1-tosyl-*N*-trityl-1H-indole-3-sulfinamide (53b)

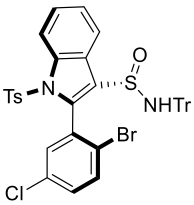

53b

According to the general procedure, react at 40 ºC for 5 days. White solid (50.4 mg, 66% yield, 6.5/1 dr, 94% ee); **R$_f$** = 0.5 (PE/EA = 4/1); **$[\alpha]^{25}_D$** = +32.9 (*c* = 2.35, CH_2Cl_2); NMR spectroscopy: **^{1}H NMR** (500 MHz, $CDCl_3$, 25 ºC) δ 8.39 (d, *J* = 8.5 Hz, 1H), 8.18 (d, *J* = 7.9 Hz, 1H), 7.57 (d, *J* = 8.6 Hz, 1H), 7.56 – 7.50 (m, 3H), 7.41 (t, *J* = 7.6 Hz, 1H), 7.33 (dd, *J* = 8.6, 2.5 Hz, 1H), 7.28 – 7.20 (m, 11H), 7.14 (dd, *J* = 7.9, 1.9 Hz, 6H), 7.05 (d, *J* = 2.5 Hz, 1H), 5.85 (s, 1H), 2.41 (s, 3H); **^{13}C NMR** (126 MHz, $CDCl_3$, 25 ºC) δ 145.9, 144.0, 136.5, 135.6, 134.7, 133.5, 133.5, 132.5, 132.5, 131.2, 130.0, 129.7, 128.0, 127.6, 127.4, 127.3, 126.5, 125.0, 124.7, 124.0, 121.0, 115.4, 73.6, 21.8; **IR (ATR)**: 3312, 3058, 2924, 2352 ,1446, 1376, 1178, 1083, 747, 697 cm^{-1}; **HRMS** (ESI, m/z): calcd for. $C_{40}H_{30}BrClN_2O_3S_2K^+$ $(M+K)^+$: 803.0201; Found: 803.0200; **HPLC** analysis (IA-3 column, *n*-hexane/^{i}PrOH = 85/15, 0.7 mL/min, 25 ºC, 220 nm) indicated 94% ee: t_R (minor) = 17.78 min, t_R (major) = 27.48 min.

(*Ra, S*)-2-(2-iodophenyl)-1-tosyl-*N*-trityl-1H-indole-3-sulfinamide (54b)

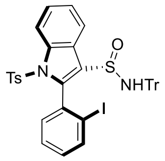

54b

According to the general procedure, react at 40 ºC for 5 days. According to the general procedure, react at 40 ºC for 5 days. White solid (36.3 mg, 48% yield, > 15/1 dr, 94% ee); **R**$_f$ = 0.55 (PE/EA = 4/1); **[α]$^{25}_{D}$** = +104.0 (*c* = 1.2 CH_2Cl_2); NMR spectroscopy: **^{1}H NMR** (500 MHz, $CDCl_3$, 25 ºC) δ 8.37 (d, *J* = 8.5 Hz, 1H), 8.24 (d, *J* = 8.0 Hz, 1H), 7.93 – 7.86 (m, 1H), 7.52 (d, *J* = 8.1 Hz, 2H), 7.48 (t, *J* = 7.9 Hz, 1H), 7.37 (t, *J* = 7.6 Hz, 1H), 7.24 – 7.17 (m, 11H), 7.13 (s, 3H), 7.03 (d, *J* = 7.0 Hz, 6H), 5.78 (s, 1H), 2.37 (s, 3H); **^{13}C NMR** (126 MHz, $CDCl_3$, 25 ºC) δ 145.7, 144.1, 139.1, 138.7, 136.1, 135.8, 134.9, 133.7, 131.0, 129.9, 129.8, 129.7, 127.9, 127.7, 127.5, 127.0, 126.1, 125.4, 124.4, 121.2, 115.2, 102.3, 73.7, 21.8; **IR (ATR)**: 3314, 3056, 2924, 2352, 1442, 1376, 1178, 1079, 755, 701 cm^{-1}; **HRMS** (ESI, m/z): calcd for. $C_{40}H_{31}IN_2O_3S_2Na^+$ $(M+Na)^+$: 801.0713; Found: 801.0707; **HPLC** analysis (IC column, *n*-hexane/^{i}PrOH = 85/15, 1.0 mL/min, 25 ºC, 254 nm) indicated 94% ee: t_R (minor) = 17.96 min, t_R (major) = 21.69 min.

(*Ra, S*)-2-(Naphthalen-1-yl)-1-tosyl-*N*-trityl-1H-indole-3-sulfinamide (55b)

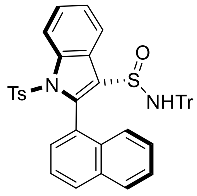

55b

According to the general procedure, react at 40 ºC for 5 days. White solid (44.5 mg, 65% yield, 7/1 dr, 97% ee (major), 48% ee (minor)); **R**$_f$ = 0.5 (PE/EA = 4/1); **[α]$^{25}_{D}$** = +96.0 (*c* = 1.0, CH_2Cl_2); NMR spectroscopy: **^{1}H NMR** (500 MHz, $CDCl_3$, 25 ºC) δ 8.66 – 8.23 (m, 2H, major + minor), 8.15 – 7.73 (m, 2H, major + minor), 7.56 (t, *J* = 7.7 Hz, 1H, major + minor), 7.51 – 7.41 (m, 2H, major + minor), 7.36 (d, *J* = 7.2 Hz, 1H, major + minor), 7.31 – 7.24 (m, 3H, major + minor), 7.23 – 7.16 (m, 5H, major + minor), 7.11 (t, *J* = 7.6 Hz, 5H, major + minor), 7.05 (t, *J* = 7.8 Hz, 1H, major + minor), 7.02 – 6.83 (m, 7H, major + minor), 6.82 (d, *J* = 7.5 Hz, 0.14H, minor), 6.78 (d, *J* = 7.9 Hz, 0.86H, major), 5.78 (s, 0.14H, minor) 5.68 (s, 0.86H, major), 2.33 (s, 0.42H, minor), 2.28 (s, 2.58H, major); **^{13}C NMR** (126 MHz, $CDCl_3$, 25 ºC) δ 145.5 (minor), 145.3 (major), 144.1 (major), 143.9 (minor), 137.2 (minor), 137.0 (major), 136.2 (minor), 135.9 (major), 135.4 (minor), 135.2 (major), 133.5 (minor), 133.1 (major), 133.0

(minor), 132.7 (major), 131.7 (major), 131.3 (minor), 130.5 (minor), 130.3 (major), 129.7 (minor), 129.6 (major), 129.5 (major + minor), 129.5 (major + minor), 128.2 (major), 128.0 (minor), 127.9 (minor), 127.7 (major), 127.7 (major + minor), 127.5 (minor), 127.4 (major) , 127.3 (major + minor), 127.0 (major), 126.8 (minor), 126.2 (minor), 126.2 (major), 126.0 (major + minor), 126.0 (major + minor), 125.5 (minor), 125.5 (major), 124.8 (minor), 124.7 (minor), 124.6 (major), 124.4 (major), 120.9 (major), 120.8 (minor), 115.8 (minor), 115.7 (major), 73.7 (minor), 73.6 (major), 21.7 (minor), 21.6 (major); **IR (ATR)**: 3317, 3058, 2854, 1679, 1494, 1377, 1177, 1074, 806 , 572 cm^{-1}; **HRMS** (ESI, m/z): calcd for. $C_{44}H_{35}N_2O_3S_2^+$ $(M+H)^+$: 703.2084; Found: 703.2078; **HPLC** analysis (two AD–H column connected in series, *n*-hexane/^{i}PrOH = 80/1, 1.0 mL/min, 25 ºC, 220 nm) indicated 97% ee of major: t_R (minor) = 17.84 min, t_R (major) = 78.83 min.

(*Ra*, *S*)-2-(4-methoxynaphthalen-1-yl)-1-tosyl-*N*-trityl-1H-indole-3-sulfinamide (56b)

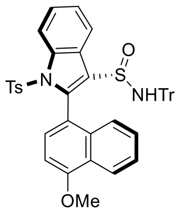

56b

According to the general procedure, react at 40 ºC for 5 days. White solid (53.6.8 mg, 75% yield, 7/1 dr, 97% ee); R_f = 0.35 (PE/EA = 4/1); $[\alpha]^{25}_D$ = +82.0 (*c* = 2.15, CH_2Cl_2); NMR spectroscopy: ^{1}**H NMR** (500 MHz, $CDCl_3$, 25 ºC) δ 8.49 (d, *J* = 8.5 Hz, 1H), 8.37 (d, *J* = 8.8 Hz, 1H), 8.29 (d, *J* = 8.3 Hz, 1H), 7.51 (ddd, *J* = 8.7, 7.3, 1.4 Hz, 1H), 7.44 – 7.37 (m, 2H), 7.27 (d, *J* = 7.9 Hz, 1H), 7.20 – 7.14 (m, 5H), 7.13 – 7.05 (m, 8H), 6.90 – 6.84 (m, 8H), 6.56 (d, *J* = 7.9 Hz, 1H), 5.64 (s, 1H), 4.03 (s, 3H), 2.23 (s, 3H); ^{13}**C NMR** (126 MHz, $CDCl_3$) δ 157.1, 145.2, 144.1, 136.9, 136.4, 135.3, 133.7, 132.5, 129.6, 129.4, 127.8, 127.7, 127.4, 127.3, 127.2, 125.8, 125.2, 125.1, 125.1, 124.4, 122.1, 120.8, 117.9, 115.6, 102.6, 73.5, 55.8, 21.6; **IR (ATR)**: 3318, 3055, 2928, 2352, 1584, 1446, 1375, 1174, 1077, 724, 566 cm^{-1}; **HRMS** (ESI, m/z): calcd for. $C_{45}H_{36}N_2O_4S_2Na^+$ $(M+Na)^+$: 755.2009; Found: 755.2000; **HPLC** analysis (IA-3 column, *n*-hexane/^{i}PrOH = 80/20, 1.0 mL/min, 25 ºC, 254 nm) indicated 97% ee: t_R (minor) = 10.12 min, t_R (major) = 12.17 min.

(*Ra*, *S*)-2-(4-bromonaphthalen-1-yl)-1-tosyl-*N*-trityl-1H-indole-3-sulfinamide (57b)

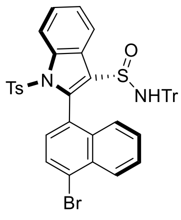

57b

According to the general procedure, react at 40 ºC for 5 days. White solid (54.5 mg, 72% yield, 4/1 dr, 89% ee); **R_f** = 0.4 (PE/EA = 4/1); **$[\alpha]^{25}_D$** = +25.9 (*c* = 1.9, CH_2Cl_2); NMR spectroscopy: **^{1}H NMR** (500 MHz, $CDCl_3$, 25 ºC) δ 8.51 – 8.45 (m, 1.2 H, major + minor + minor), 8.38 (d, *J* = 7.9 Hz, 0.8H, major), 8.28 (dd, *J* = 8.6, 4.9 Hz, 1 H, major + minor), 7.75 (d, *J* = 7.5 Hz, 0.2H, minor). 7.59 – 7.39 (m, 4H, major + minor), 7.35 (d, *J* = 8.5 Hz, 1H, minor), 7.24 – 7.02 (m, 14H, major + minor), 6.92 (d, *J* = 8.2 Hz, 1.6 H, major), 6.90 – 6.86 (m, 4.8 H, major), 6.71 (d, *J* = 7.2 Hz, 1H, major + minor), 5.72 (s, 0.2H, minor), 5.64 (s, 0.8H, major), 2.31 (s, 0.6H, minor), 2.26 (s, 2.4H, major); **^{13}C NMR** (126 MHz, $CDCl_3$) δ 145.8 (minor), 145.6 (major), 143.9 (major), 143.8 (minor), 137.2 (minor), 137.0 (major), 135.3 (minor), 135.2 (major), 135.1 (minor), 134.8 (major), 134.5 (minor), 133.8 (major), 131.7 (major), 131.6 (minor), 129.8 (minor), 129.6 (major), 129.5 (major + minor), 129.5 (major + minor), 128.7 (minor), 128.7 (major), 128.3 (major + minor), 127.8 (major + minor), 127.8 (major + minor), 127.8 (major), 127.7 (minor), 127.6 (minor), 127.5 (major), 127.4 (minor), 127.2 (major), 126.7 (major + minor), 126.4 (minor), 126.3 (minor), 126.2 (major), 126.0 (major), 125.6 (major + minor), 125.4 (major + minor), 124.9 (minor), 124.7 (major), 121.0 (minor), 120.9 (major), 115.8 (minor) , 115.7 (major), 73.8 (minor), 73.6 (major), 21.73 (minor), 21.68 (major); **IR (ATR)**: 3317, 3056, 2926, 2352, 1587, 1446, 1376, 1176, 1077, 728 cm^{-1}; **HRMS** (ESI, m/z): calcd for. $C_{44}H_{34}BrN_2O_3S_2^+$ $(M+H)^+$: 781.1189; Found: 781.1187; **HPLC** analysis (IA-3 column, *n*-hexane/^{i}PrOH = 85/15, 0.7 mL/min, 25 ºC, 220 nm) indicated 89% ee: t_R (minor) = 13.96 min, t_R (major) = 20.76 min.

(*Ra*, *S*)-2-(1-bromonaphthalen-2-yl)-1-tosyl-*N*-trityl-1H-indole-3-sulfinamide (58b)

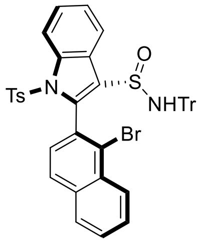

58b

According to the general procedure, react at 40 ºC for 5 days. White solid (54.1 mg, 71% yield, 10/1 dr, 87% ee); **R_f** = 0.35 (PE/EA = 4/1); **$[\alpha]^{25}_D$** = +72.0 (c = 2.4, CH_2Cl_2); NMR spectroscopy: **^{1}H NMR** (500 MHz, $CDCl_3$, 25 ºC) δ 8.40 (d, J = 8.5 Hz, 1H), 8.31 (d, J = 8.1 Hz, 1H), 8.28 (d, J = 8.2 Hz, 1H), 7.89 (d, J = 7.7 Hz, 1H), 7.73 – 7.64 (m, 2H), 7.57 (d, J = 8.4 Hz, 1H), 7.53 – 7.49 (m, 3H), 7.43 – 7.37 (m, 1H), 7.29 (d, J = 8.4 Hz, 1H), 7.11 (dd, J = 16.3, 7.8 Hz, 5H), 7.00 (t, J = 7.9 Hz, 6H), 6.90 (d, J = 7.1 Hz, 6H), 5.80 (s, 1H), 2.33 (s, 3H); **^{13}C NMR** (126 MHz, $CDCl_3$) δ 145.7, 144.0, 137.0, 136.3, 135.4, 134.7, 132.0, 130.3, 129.8, 129.6, 129.1, 128.5, 128.3, 128.0, 127.9, 127.7, 127.7, 127.4, 126.9, 126.5, 126.5, 126.1, 125.5, 124.5, 121.1, 115.2, 73.6, 21.7; **IR (ATR)**: 3313, 3056, 2352, 1445, 1374, 1176, 1078, 735 cm^{-1}; **HRMS** (ESI, m/z): calcd for. $C_{44}H_{33}BrN_2O_3S_2Na^+$ $(M+Na)^+$: 803.1008; Found: 803.1007; **HPLC** analysis (IA-3 column, n-hexane/^{i}PrOH = 80/20, 1.0 mL/min, 25 ºC, 254 nm) indicated 87% ee: t_R (minor) = 11.78 min, t_R (major) = 44.35 min

(*Ra, S*)-2-(3-bromonaphthalen-2-yl)-1-tosyl-*N*-trityl-1H-indole-3-sulfinamide (69b)

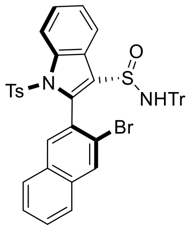

59b

According to the general procedure, react at 40 ºC for 3 days. White solid (55.3 mg, 73% yield, 12/1 dr, 91% ee); **R_f** = 0.45 (PE/EA = 4/1); **$[\alpha]^{25}_D$** = +50.6 (c = 1.9, CH_2Cl_2); NMR spectroscopy: **^{1}H NMR** (500 MHz, $CDCl_3$, 25 ºC) δ 8.43 (d, J = 8.5 Hz, 1H), 8.30 (d, J = 7.9 Hz, 1H), 8.16 (s, 1H), 7.88 (d, J = 8.2 Hz, 1H), 7.62 (t, J = 8.2 Hz, 1H), 7.57 (s, 1H), 7.56 – 7.51 (m, 1H), 7.49 (d, J = 8.4 Hz, 3H), 7.46 – 7.41 (m, 1H), 7.27 – 7.17 (m, 6H), 7.11 (t, J = 7.8 Hz, 6H), 7.03 (d, J = 7.1 Hz, 6H), 5.84 (s, 1H), 2.42 (s, 3H); **^{13}C NMR** (126 MHz, $CDCl_3$) δ 145.6, 144.1, 136.4, 136.1, 135.8, 134.8, 133.7, 131.0, 131.0, 129.8, 129.7, 128.3, 128.1, 127.8, 127.8, 127.5, 127.4, 127.0, 126.9, 126.2, 125.4, 124.6, 122.5, 121.1, 115.5, 73.6, 21.8; **IR (ATR)**: 3315, 3055, 2952, 1560, 1442, 1376, 1177, 1078, 739 cm^{-1}; **HRMS** (ESI, m/z): calcd for. $C_{44}H_{33}BrN_2O_3S_2K^+$ $(M+K)^+$: 819.0748; Found: 819.0730; **HPLC** analysis (IA-3 column, n-hexane/^{i}PrOH = 80/20, 1.0 mL/min, 25 ºC, 254 nm) indicated 91% ee: t_R (minor) = 10.20 min, t_R (major) =

14.61 min.

(*Ra*, *S*)-2-(8a,10a-Dihydrophenanthren-9-yl)-1-tosyl-*N*-trityl-1H-indole-3-sulfinamide (60b)

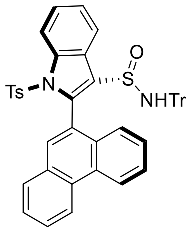

60b

According to the general procedure, react at 40 ºC for 5 days. White solid 35.1 mg, 48% yield, 12/1 dr, 97% ee; **Rf** = 0.5 (PE/EA = 4/1); **[α]^{25}D** = +26.7 (*c* = 0.75, CH_2Cl_2); NMR spectroscopy: **^{1}H NMR** (500 MHz, $CDCl_3$, 25 ºC) 8.75 (d, *J* = 8.4 Hz, 1H), 8.71 (d, *J* = 8.4 Hz, 1H), 8.51 (d, *J* = 8.5 Hz, 1H), 8.39 (d, *J* = 7.9 Hz, 1H), 7.75 (t, *J* = 7.7 Hz, 1H), 7.61 (t, *J* = 7.6 Hz, 1H), 7.55 (td, *J* = 7.7, 3.0 Hz, 2H), 7.50 – 7.42 (m, 3H), 7.34 (t, *J* = 7.6 Hz, 1H), 7.28 (d, *J* = 8.1 Hz, 2H), 7.23 (d, *J* = 7.8 Hz, 1H), 7.13 (t, *J* = 7.3 Hz, 3H), 7.03 – 6.95 (m, 8H), 6.91 (d, *J* = 7.9 Hz, 6H), 5.75 (s, 1H), 2.33 (s, 3H); **^{13}C NMR** (126 MHz, $CDCl_3$, 25 ºC) δ 145.4, 144.1, 137.1, 136.0, 135.4, 132.7, 131.6, 131.2, 130.3, 129.9, 129.7, 129.6, 129.4, 129.2, 128.6, 128.1, 127.7, 127.6, 127.4, 127.4, 127.3, 127.0, 126.9, 126.7, 126.1, 125.6, 125.6, 124.7, 122.8, 122.7, 121.0, 115.8, 73.6, 21.7; **R (ATR)**: 3342, 2969, 1449, 1376, 1310, 1159, 1127, 950, 816, 573 cm^{-1}; **HRMS** (ESI, m/z): calcd for. $C_{48}H_{37}N_2O_3S_2^+$ (M+H)$^+$: 753.2240; Found: 753.2236; **HPLC** analysis (two AD–H column connected in series, *n*-hexane/^{i}PrOH = 80/1, 0.5 mL/min, 25 ºC, 220 nm) indicated 97% ee; t_R (minor) = 37.80 min, t_R (major) = 50.09 min;

(*Ra*, *S*)-2-(o-tolyl)-1-tosyl-*N*-trityl-1H-benzo[f]indole-3-sulfinamide (61b)

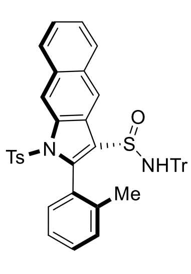

33b

According to the general procedure, react at 25 ºC for 40 h. White solid (58.4 mg, 84% yield, 3.5/1 dr, 86% ee); **Rf** = 0.45 (PE/EA = 4/1); **[α]^{25}D** = +71.2 (*c* = 1.45, CH_2Cl_2); NMR spectroscopy: **^{1}H NMR** (500 MHz, $CDCl_3$, 25 ºC) δ 8.91 (s, 1H), 8.80 (s, 1H), 8.08 (d, *J* = 8.3 Hz, 1H), 7.99 (d, *J* = 8.1 Hz, 1H), 7.58 – 7.49 (m, 2H), 7.43 (d, *J* = 8.4 Hz, 2H), 7.38 (ddd, *J* = 7.8, 6.1, 2.8 Hz, 1H), 7.26 – 7.19 (m, 10H), 7.13 (d, *J* = 8.2 Hz, 2H), 7.09 – 7.04 (d, *J* = 1.7 Hz, 6H), 7.01 – 6.97 (m, 2H), 5.80 (s, 1H), 2.36 (s, 3H), 2.01 (s, 3H); **^{13}C NMR** (126 MHz, $CDCl_3$) δ 145.5, 144.2, 140.4, 139.1, 136.0, 135.6, 132.2, 131.8,

130.9, 130.2, 130.0, 129.7, 129.7, 128.7, 128.6, 128.4, 127.9, 127.5, 127.3, 127.1, 126.0, 125.5, 125.1, 124.8, 119.0, 113.2, 73.7, 21.8, 20.3; **IR (ATR)**: 3313, 3054, 2926, 2352, 1442, 1375, 1175, 1078, 741, 699, 578 cm^{-1}; **HRMS** (ESI, m/z): calcd for. $C_{45}H_{36}N_2O_3S_2Na^+$ $(M+Na)^+$: 739.2060; Found: 739.2053; **HPLC** analysis (IA-3 column, *n*-hexane/^{i}PrOH = 85/15, 0.7 mL/min, 25 ºC, 220 nm) indicated 86% ee: t_R (minor) = 27.13 min, t_R (major) =37.53 min.

4 Synthetic Transformations

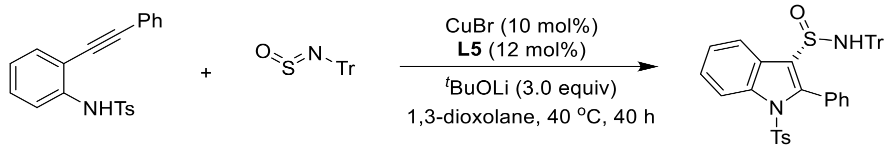

1a (1.0 equiv.) **I** (3.0 equiv.) **1b**

In a nitrogen-filled glove box, to an oven-dried 50 mL screw cap vial equipped with a magnetic stir bar was added CuBr (14.3 mg, 0.10 mmol, 10 mol%), **L5** (105 mg, 0.12 mmol, 12 mol%), **1a** (347 mg, 1.0 mmol, 1.0 equiv.), **I** (915 mg, 3.0 mmol, 3.0 equiv.), ^{t}BuOLi (240 mg, 3.0 mmol, 3.0 equiv.) and anhydrous 1,3-dioxolane (10 mL). The tube was sealed with a teflon-lined screw cap, removed from the glove box and the reaction was stirred at 40 °C for 40 hours. Afterwards, the mixture was cooled to room temperature. The solvent was evaporated under reduced pressure. The crude product was added DCM (~5 mL) and purified by column chromatography on silica gel (SiliaFlash® P60, particle size 40-63 µm, 230-400 mesh, Silicycle, PE/EtOAc with 1% Et_3N as eluent) to afford the corresponding product **1b** (580 mg, 89% yield, 95% ee).

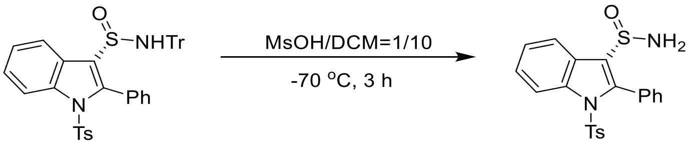

1b **62**

A solution of **1b** (65.3 mg, 0.1 mmol) in DCM (1 mL) was cooled to -70 ºC, and MsOH (100 uL) was then added. The reaction was stirred at -70 ºC for 3 h (Attention**:** The reaction time should ensure complete conversion of **1b**, otherwise it will result in

loss of enantioselectivity). The mixture was quickly diluted with DCM (5 mL) and quickly transferred to a stirring solution of sat. aq $NaHCO_3$ (10 mL) in a 50 mL breaker. After collecting the organic phase, the aqueous phase was extracted with DCM (2 × 10 mL). The combined organic phases were dried (Na_2SO_4), filtered and concentrated. Purification by flash column chromatography (EA with 1% Et_3N) afforded the product **62** as an off-white solid (23.3 mg, 57%, 92% ee). **R_f** = 0.6 (EA with 1% Et_3N); **$[\alpha]^{25}_D$** = +37.3 (c = 0.31, CH_2Cl_2); **^{1}H NMR** (500 MHz, $CDCl_3$, 25 ºC) δ 8.40 (d, J = 8.5 Hz, 1H), 8.23 (d, J = 7.9 Hz, 1H), 7.57 – 7.36 (m, 7H), 7.33 (d, J = 8.1 Hz, 2H), 7.11 (d, J = 8.0 Hz, 2H), 4.49 (s, 2H), 2.34 (s, 3H). **^{13}C NMR** (126 MHz, $CDCl_3$, 25 ºC) δ 145.6, 139.6, 137.0, 135.3, 131.7, 130.0, 129.8, 128.7, 127.7, 127.1, 127.0, 126.1, 124.8, 124.5, 120.4, 116.3, 21.7; **IR (ATR)**: 3324, 2926, 1446, 1377, 1312, 1179, 1060, 948, 776, 572 cm^{-1}; **HRMS** (ESI, m/z): calcd for. $C_{21}H_{19}N_2O_3S_2^+$ $(M+H)^+$: 411.0832; Found: 411.0826; **HPLC** (IB column, n-hexane/^{i}PrOH = 80/20, 1.0 mL/min, 25 ºC, 220 nm) indicated 92% ee: t_R (minor) = 12.48 min, t_R (major) = 14.42 min.

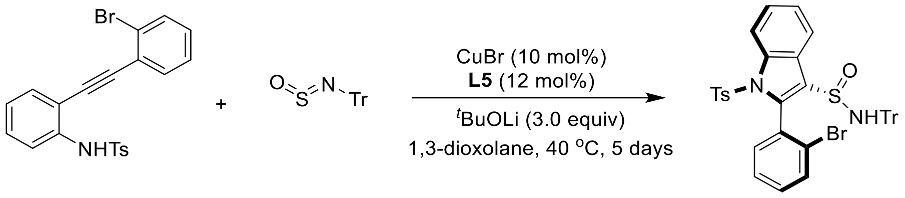

52a (1.0 equiv.) **I** (3.0 equiv.) **52b**

In a nitrogen-filled glove box, to an oven-dried 50 mL screw cap vial equipped with a magnetic stir bar was added CuBr (14.3 mg, 0.10 mmol, 10 mol%), **L5** (105 mg, 0.12 mmol, 12 mol%), **52a** (426 mg, 1.0 mmol, 1.0 equiv.), **I** (915 mg, 3.0 mmol, 3.0 equiv.), ^{t}BuOLi (240 mg, 3.0 mmol, 3.0 equiv.) and anhydrous 1,3-dioxolane (10 mL). The tube was sealed with a teflon-lined screw cap, removed from the glove box and the reaction was stirred at 40 °C for 5 days. Afterwards, the mixture was cooled to room temperature. The solvent was evaporated under reduced pressure. The crude product was added DCM (~5 mL) and purified by column chromatography on silica gel (SiliaFlash® P60, particle size 40-63 µm, 230-400 mesh, Silicycle, PE/EtOAc with 1% Et_3N as eluent) to afford the corresponding product **52b** (519 mg, 73% yield, >15/1 dr, 94% ee).

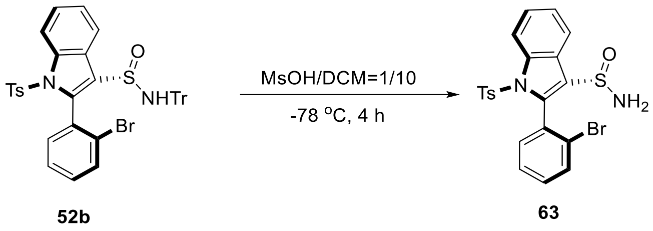

A solution of **52b** (35.5 mg, 0.05 mmol) in DCM (0.5 mL) was cooled to -78 °C, and MsOH (50 uL) was then added. The reaction was stirred at -78 °C for 4 h (Attention: The reaction time should ensure complete conversion of **53b**, otherwise it will result in loss of enantioselectivity). The mixture was quickly diluted with DCM (5 mL) and quickly transferred to a stirring solution of sat. aq $NaHCO_3$ (10 mL) in a 50 mL breaker. After collecting the organic phase, the aqueous phase was extracted with DCM (2 × 10 mL). The combined organic phases were dried (Na_2SO_4), filtered and concentrated. Purification by flash column chromatography (EA with 1% Et_3N) afforded the product **63** as an off-white solid (12.1 mg, 52%, >15/1 dr, 92% ee). $\mathbf{R_f}$ = 0.6 (EA with 1% Et_3N); $[\alpha]^{25}_D$ = +60.0 (*c* = 0.45, CH_2Cl_2). **^{1}H NMR** (500 MHz, $CDCl_3$, 25 °C) δ 8.35 (d, *J* = 8.5 Hz, 1H), 8.19 (d, *J* = 7.9 Hz, 1H), 7.69 – 7.63 (m, 1H), 7.52 (d, *J* = 8.4 Hz, 2H), 7.50 – 7.46 (m, 1H), 7.38 (tdd, *J* = 9.6, 7.8, 4.2 Hz, 4H), 7.18 (d, *J* = 8.1 Hz, 2H), 4.47 (s, 2H), 2.36 (s, 3H); **^{13}C NMR** (126 MHz, $CDCl_3$, 25 °C) 145.8, 137.0, 136.4, 135.5, 134.0, 132.6, 131.5, 130.5, 129.9, 127.5, 127.3, 126.4, 126.2, 125.8, 124.5, 124.5, 120.6, 115.5, 21.8; **IR (ATR)**: 3358, 3217, 2923, 2352, 1442, 1376, 1176, 1045, 754, 662, 576 cm^{-1}; **HRMS** (ESI, m/z): calcd for. $C_{21}H_{18}BrN_2O_3S_2^+$ $(M+H)^+$: 488.9937; Found: 488.9928; **HPLC** (OD-H column, *n*-hexane/^{i}PrOH = 85/15, 0.7 mL/min, 25 °C, 220 nm) indicated 92% ee: t_R (major) = 45.81 min, t_R (minor) = 55.92 min.

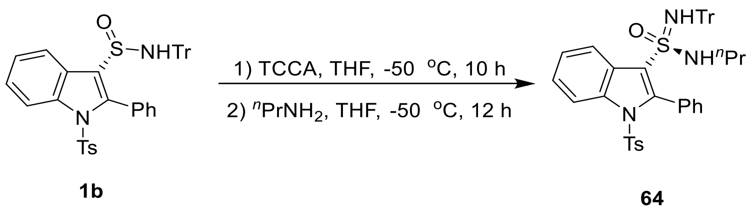

To a 10 mL Schlenk tube was added **1b** (32.7 mg, 0.05 mmol, 1.0 equiv) and trichloroisocyanuric acid (11.6 mg, 0.05 mmol, 1.0 equiv.) under Ar. THF (0.5 mL) was

added at -50 °C, and the mixture was stirred for 10 h at -50 °C. Then $^{n}PrNH_2$ (8.9 mg, 0.15 mmol, 3.0 equiv.) was added and the mixture was stirred at -50 °C for 12 h. The mixture was through a short plug of diatomite eluting with ethyl acetate (ca. 10 mL) and concentrated. The crude product was purified with flash column chromatography on silica gel (SiliaFlash® P60, particle size 40-63 μm, 230-400 mesh, Silicycle, PE/EtOAc with 1% Et_3N as eluent) to afford the corresponding product **64** as a colorless oil (25.7 mg, 67% yield, 92% ee). **R_f** = 0.5 (PE/EA = 5/1); **$[\alpha]^{25}_D$** = +34.7 (c = 0.37, CH_2Cl_2); **^{1}H NMR** (500 MHz, $CDCl_3$, 25 ºC) δ 8.37 (d, J = 8.5 Hz, 1H), 8.20 (d, J = 8.1 Hz, 1H), 7.47 – 7.41 (m, 3H), 7.41 – 7.31 (m, 10H), 7.29 (s, 1H), 7.25 – 7.23 (m, 1H), 7.16 (d, J = 8.2 Hz, 2H), 7.11 (q, J = 8.4, 7.5 Hz, 9H), 2.89 (s, 1H), 2.46 – 2.35 (m, 4H), 2.17 – 2.07 (m, 1H), 0.85 – 0.67 (m, 2H), 0.46 (t, J = 7.4 Hz, 3H). **^{13}C NMR** (126 MHz, $CDCl_3$, 25 ºC) δ 148.0, 145.5, 138.7, 135.9, 135.8, 131.9, 131.9, 129.9, 129.8, 129.3, 129.2, 128.1, 127.4, 127.3, 127.2, 127.1, 126.3, 125.8, 124.6, 122.5, 73.0, 44.7, 22.5, 21.8, 11.2; **IR (ATR)**: 3059, 2927, 2005, 1915, 1596, 1446, 1297, 1177, 949, 570 cm^{-1}; **HRMS** (ESI, m/z): calcd for. $C_{43}H_{40}N_3O_3S_2^+$ $(M+H)^+$: 710.2506; Found: 710.2502; **HPLC** (IC column, n-hexane/^{i}PrOH = 96/4, 0.6 mL/min, 25 ºC, 254 nm) indicated 92% ee: t_R (minor) = 17.10 min, t_R (major) = 19.81 min;

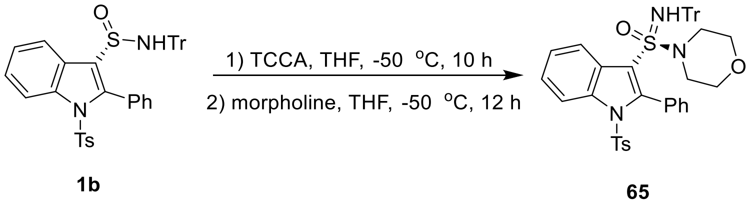

To a 10 mL Schlenk tube was added **1b** (32.7 mg, 0.05 mmol, 1.0 equiv) and trichloroisocyanuric acid (11.6 mg, 0.05 mmol, 1.0 equiv.) under Ar. THF (0.5 mL) was added at -50 °C, and the mixture was stirred for 10 h at -50 °C. Then morpholine (13.1 mg, 0.15 mmol, 3.0 equiv.) was added and the mixture was stirred at -50 °C for 12 h. The mixture was through a short plug of diatomite eluting with ethyl acetate (ca. 10 mL) and concentrated. The crude product was purified with flash column chromatography on silica gel (SiliaFlash® P60, particle size 40-63 μm, 230-400 mesh, Silicycle,

PE/EtOAc with 1% Et_3N as eluent) to afford the corresponding product **65** as a colorless oil (21.2 mg, 58% yield, 93% ee). **R_f** = 0.3 (PE/EA = 5/1); **$[\alpha]^{25}_D$** = +48.0 (c = 0.4, CH_2Cl_2); **^{1}H NMR** (500 MHz, $CDCl_3$, 25 ºC) δ 8.38 (d, J = 8.5 Hz, 1H), 8.20 (d, J = 8.1 Hz, 1H), 7.42 (tdd, J = 7.4, 3.4, 1.3 Hz, 2H), 7.35 (s, 1H), 7.34 – 7.30 (m, 7H), 7.29 – 7.27 (m, 2H), 7.24 (s, 1H), 7.17 (t, J = 8.3 Hz, 3H), 7.11 – 7.04 (m, 9H), 7.03 (d, J = 7.6 Hz, 1H), 3.25 (dddd, J = 17.5, 14.6, 11.4, 3.2 Hz, 4H), 2.87 – 2.72 (m, 4H), 2.40 (s, 3H). **^{13}C NMR** (126 MHz, $CDCl_3$, 25 ºC) δ 147.9, 145.5, 141.5, 135.9, 135.9, 132.6, 132.4, 129.7, 129.6, 129.4, 129.1, 127.3, 127.2, 126.7, 126.7, 126.2, 125.8, 124.1, 123.2, 115.2, 66.3, 45.6, 21.8; **IR (ATR)**: 3058, 2856, 1739, 1540, 1446, 1298, 1176, 1030, 775, 568 cm^{-1}; **HRMS** (ESI, m/z): calcd for. $C_{44}H_{40}N_3O_4S_2^+$ $(M+H)^+$: 760.2274; Found: 760.2269; **HPLC** (IA column, n-hexane/^{i}PrOH = 95/5, 1.0 mL/min, 25 ºC, 220 nm) indicated 93% ee: t_R (major) = 13.29 min, t_R (minor) = 16.50 min;

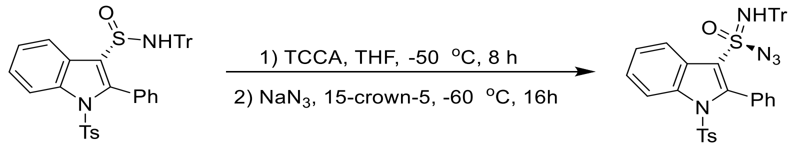

1b **66**

To a 10 mL Schlenk tube was added **1b** (32.7 mg, 0.05 mmol, 1.0 equiv) and trichloroisocyanuric acid (11.6 mg, 0.05 mmol, 1.0 equiv.) under Ar. THF (0.5 mL) was added at -50 °C, and the mixture was stirred for 8 h at -50 °C. Then NaN_3 (10.2 mg, 0.15 mmol, 3.0 equiv.) and 15-crown-5 (33 mg, 0.15 mmol, 3.0 equiv.) werw added and the mixture was stirred at -60 °C for 16 h. The mixture was through a short plug of diatomite eluting with ethyl acetate (ca. 10 mL) and concentrated. The crude product was purified with flash column chromatography on silica gel (SiliaFlash® P60, particle size 40-63 μm, 230-400 mesh, Silicycle, PE/EtOAc with 1% Et_3N as eluent) to afford the corresponding product **66** as a colorless oil (20.9 mg, 60% yield, 90% ee); **$[\alpha]^{25}_D$** = +62.4 (c = 0.35, CH_2Cl_2); **R_f** = 0.6 (PE/EA = 5/1); **^{1}H NMR** (500 MHz, $CDCl_3$, 25 ºC) δ 8.45 (d, J = 8.5 Hz, 1H), 8.20 (d, J = 8.1 Hz, 1H), 7.51 (t, J = 8.0 Hz, 1H), 7.46 (t, J = 7.5 Hz, 1H), 7.44 – 7.26 (m, 13H), 7.25 – 7.14 (m, 11H), 2.40 (s, 3H). **^{13}C NMR** (126 MHz, $CDCl_3$, 25 ºC) δ 146.1, 145.9, 142.1, 135.7, 135.7, 132.0, 130.2, 129.9, 129.1,

128.1, 127.9, 127.7, 127.5, 127.3, 126.9, 126.5, 125.0, 124.9, 122.0, 115.3, 74.7, 21.9; **IR (ATR)**: 3061, 2925, 2101, 1596, 1446, 1309, 1178, 1017, 777, 542 cm^{-1}; **HRMS** (ESI, m/z): calcd for. $C_{40}H_{31}N_5O_3S_2Na^+$ $(M+Na)^+$: 716.1761; Found: 716.1758; **HPLC** (IA column, *n*-hexane/^{i}PrOH = 95/5, 1.0 mL/min, 25 ºC, 254 nm) indicated 90% ee: t_R (major) = 8.20 min, t_R (minor) = 11.02 min.

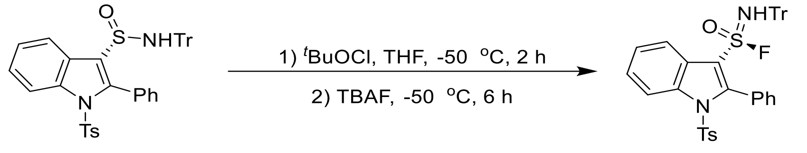

1b **67**

Under Ar, a solution of **1b** (32.7 mg, 0.05 mmol, 1.0 equiv) in THF (0.5 mL) was cooled to -50 ºC, and tert-butyl hypochlorite (5.4 mg, 0.05 mmol) was then slowly added. The reaction was stirred at -50ºC for 2 h. Then TBAF (100 uL, 0.1 mmol, 2.0 equiv.) was added and the reaction mixture was stirred at -50 ºC for 6 h. The mixture was through a short plug of diatomite eluting with ethyl acetate (ca. 10 mL) and concentrated. The crude product was purified with flash column chromatography on silica gel (SiliaFlash® P60, particle size 40-63 µm, 230-400 mesh, Silicycle, PE/EtOAc with 1% Et_3N as eluent) to afford the corresponding product **67** as an colorless as a colorless oil (18.9 mg, 56% yield, 85% ee); $\mathbf{R_f}$ = 0.6 (PE/EA = 5/1); $\mathbf{[\alpha]^{25}_D}$ = +38.1 (*c* = 0.2, CH_2Cl_2). ^{1}**H NMR** (500 MHz, $CDCl_3$, 25 ºC) δ 8.47 (d, *J* = 8.5 Hz, 1H), 8.10 (d, *J* = 8.0 Hz, 1H), 7.57 – 7.52 (m, 1H), 7.52 – 7.47 (m, 1H), 7.45 – 7.38 (m, 4H), 7.33 – 7.29 (m, 3H), 7.27 – 7.24 (m, 6H), 7.23 (q, *J* = 3.0 Hz, 9H), 7.19 (d, *J* = 8.2 Hz, 2H), 2.41 (s, 3H). ^{13}**C NMR** (126 MHz, $CDCl_3$, 25 ºC) 146.2, 146.2, 146.0, 142.5, 135.6, 132.2, 131.7, 130.2, 129.9, 129.0, 128.3, 128.1, 127.7, 127.4, 127.2, 127.0, 126.5, 125.1, 121.3, 115.4, 75.0, 21.8. ^{19}**F NMR** (377 MHz, $CDCl_3$, 25 ºC) δ 98.08 (s, 1F); **IR (ATR)**: 3062, 2924, 1596, 1447, 1381, 1181, 1136, 1030, 779, 570 cm^{-1}; **HRMS** (ESI, m/z): calcd for. $C_{40}H_{31}FN_2O_3S_2Na^+$ $(M+Na)^+$: 693.1652; Found: 692.1646; **HPLC** (IC column, *n*-hexane/^{i}PrOH = 93/7, 0.6 mL/min, 25 ºC, 254 nm) indicated 85% ee: t_R (major) = 11.69 min, t_R (minor) = 12.92 min.

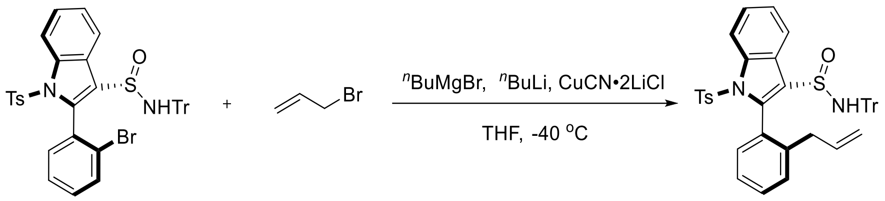

52b **68**

To a 5 mL Schlenk tube were added ^{n}BuMgBr (1 M in THF, 60 uL, 0.06 mmol, 1.2 equiv), THF (0.1 mL) and ^{n}BuLi (2.5 M in hexane, 48 uL, 0.12 mmol, 2.4 equiv.) at 0 °C under Ar. The mixture was stirred at 0 °C for 0.5 h. Then the solution of **52b** (35.5 mg, 0.05 mmol, 1 equiv.) in THF (0.1 mL) was added at -40 °C. The mixture was stirred -40 °C for 2 h. Afterwards, CuCN·2LiCl (1 M in THF, 50 uL, 0.05 mmol, 1.0 equiv.) and allyl bromide (18 uL, 0.2 mmol, 4.0 equiv.) were added at -40 °C. After stirring at -40 °C for 2 hours, the reaction was quenched by a short plug of diatomite eluting with ethyl acetate (ca. 10 mL) and concentrated. The crude product was purified with flash column chromatography on silica gel (SiliaFlash® P60, particle size 40-63 µm, 230-400 mesh, Silicycle, PE/EtOAc with 1% Et_3N as eluent) to afford the corresponding product **68** as a colorless oil (24.5 mg, 71%, > 15/1 dr, 93% ee). $\mathbf{R_f}$ = 0.55 (PE/EA = 4/1); $[\alpha]^{25}_D$ = +158.9 (c = 0.55, CH_2Cl_2). **^{1}H NMR** (500 MHz, $CDCl_3$, 25 °C) δ 8.41 (d, J = 8.5 Hz, 1H), 8.37 (d, J = 8.5 Hz, 1H), 7.48 (ddd, J = 8.5, 7.2, 1.3 Hz, 1H), 7.43 – 7.37 (m, 4H), 7.28 (d, J = 7.8 Hz, 1H), 7.25 – 7.15 (m, 11H), 7.01 (dd, J = 7.0, 1.7 Hz, 6H), 6.90 – 6.83 (m, 2H), 5.70 – 5.59 (m, 2H), 4.92 (dd, J = 10.0, 1.7 Hz, 1H), 4.84 (dd, J = 17.0, 1.7 Hz, 1H), 3.13 – 2.96 (m, 2H), 2.39 (s, 3H); **^{13}C NMR** (126 MHz, $CDCl_3$, 25 °C) 145.5, 144.2, 141.2, 136.9, 136.7, 135.9, 135.8, 132.6, 130.1, 129.8, 128.8, 128.4, 127.8, 127.5, 127.3, 127.2, 125.9, 125.4, 125.1, 124.5, 121.0, 117.0, 115.6, 73.7, 37.7, 21.8; **IR (ATR)**: 3316, 3059, 2924, 2352, 1444, 1376, 1179, 1077, 756 cm^{-1}; **HRMS** (ESI, m/z): calcd for. $C_{43}H_{36}N_2O_3S_2Na^+$ $(M+Na)^+$: 715.2060; Found: 715.2059; **HPLC** (IA-3 column, n-hexane/^{i}PrOH = 80/20, 1 mL/min, 25 °C, 220 nm) indicated 93% ee: t_R (minor) = 7.20 min, t_R (major) = 41.35 min.

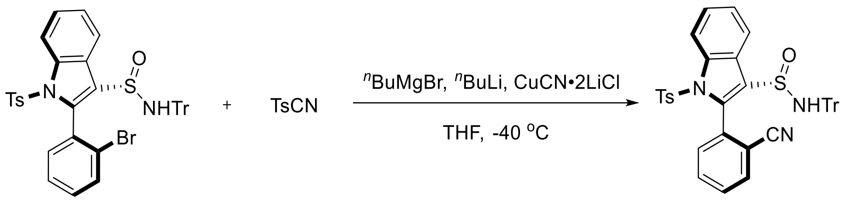

52b **69**

To a 5 mL Schlenk tube were added ^{n}BuMgBr (1 M in THF, 60 uL, 0.06 mmol, 1.2 equiv), THF (0.1 mL) and ^{n}BuLi (2.5 M in hexane, 48 uL, 0.12 mmol, 2.4 equiv.) at 0 ^{o}C under Ar. The mixture was stirred at 0 ^{o}C for 0.5 h. Then the solution of **52b** (35.5 mg, 0.05 mmol, 1 equiv.) in THF (0.1 mL) was added at -40 ^{o}C. The mixture was stirred -40 ^{o}C for 2 h. Afterwards, CuCN·2LiCl (1 M in THF, 50 uL, 0.05 mmol, 1.0 equiv.) and the solution of *p*-tosyl cyanide (36.2 mg, 0.2 mmol, 4.0 equiv.) in THF (0.1 Ml) were added at -40 ^{o}C. After stirring at -40 ^{o}C for 2 hour, the reaction was quenched by a short plug of diatomite eluting with ethyl acetate (ca. 10 mL) and concentrated. The crude product was purified with flash column chromatography on silica gel (SiliaFlash® P60, particle size 40-63 µm, 230-400 mesh, Silicycle, PE/EtOAc with 1% Et_3N as eluent) to afford the corresponding product **69** as a colorless oil (16.9 mg, 50%, >15/1 dr, 91% ee). $\mathbf{R_f}$ = 0.45 (PE/EA = 2/1); $[\alpha]^{25}_{D}$ = +36.3 (*c* = 0.65, CH_2Cl_2). **^{1}H NMR** (500 MHz, $CDCl_3$, 25 ºC) δ 8.36 (d, *J* = 7.9 Hz, 1H), 7.93 (s, 1H), 7.74 (s, 1H), 7.63 – 7.55 (m, 1H), 7.55 – 7.40 (m, 5H), 7.35 (s, 1H), 7.30 – 7.17 (m, 17H), 6.09 (s, 1H), 2.39 (s, 3H). **^{13}C NMR** (126 MHz, $CDCl_3$, 25 ºC) 146.0, 144.0, 136.9, 135.1, 134.5, 133.7, 133.3, 131.7, 131.4, 130.1, 129.8, 127.9, 127.6, 127.2, 126.6, 127.6, 125.8, 124.7, 121.2, 118.4, 115.8, 115.6, 73.7, 21.8; **IR (ATR)**: 3739, 3356, 2922, 2352, 1647, 1445, 1377, 1179, 1087, 743 cm^{-1}; **HRMS** (ESI, m/z): calcd for. $C_{41}H_{31}N_3O_3S_2Na^+$ $(M+Na)^+$: 700.1699; Found: 700.1689; **HPLC** (IA-3 column, *n*-hexane/^{i}PrOH = 80/20, 1.0 mL/min, 25 ºC, 220 nm) indicated 91% ee: t_R (minor) = 10.92 min, t_R (major) = 69.08 min.

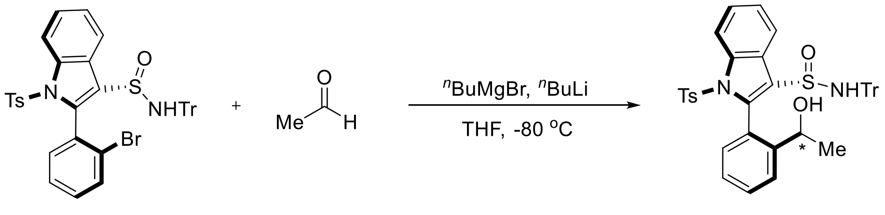

52b **70**

To a 5 mL Schlenk tube were added ^{n}BuMgBr (1 M in THF, 60 uL, 0.06 mmol, 1.2 equiv), THF (0.1 mL) and ^{n}BuLi (2.5 M in hexane, 48 uL, 0.12 mmol, 2.4 equiv.) at 0 ^{o}C under Ar. The mixture was stirred at 0 ^{o}C for 0.5 h. Then the solution of **52b** (35.5 mg, 0.05 mmol, 1 equiv.) in THF (0.1 mL) was added at -80 ^{o}C. The mixture was stirred -80 ^{o}C for 2 h. Afterwards, acetaldehyde (5 M in THF, 40 uL, 0.2 mmol, 4.0 equiv.) were added at -40 ^{o}C. After stirring at -80 ^{o}C for 2 hour, the reaction was quenched by a short plug of diatomite eluting with ethyl acetate (ca. 10 mL) and concentrated. The crude product was purified with flash column chromatography on silica gel (SiliaFlash® P60, particle size 40-63 µm, 230-400 mesh, Silicycle, PE/EtOAc with 1% Et_3N as eluent) to afford the corresponding product **70** as a colorless oil (22.3 mg, 64%, >15/1 dr, 90% ee). **R_f** = 0.5 (PE/EA = 2/1); **$[\alpha]^{25}_D$** = +46.4 (c = 0.5, CH_2Cl_2). **^{1}H NMR** (500 MHz, $CDCl_3$, 25 °C) δ 8.39 (d, J = 8.5 Hz, 1H), 7.94 (d, J = 8.0 Hz, 1H), 7.64 (d, J = 7.9 Hz, 1H), 7.51 – 7.42 (m, 4H), 7.29 (t, J = 7.6 Hz, 1H), 7.25 – 7.21 (m, 8H), 7.19 (d, J = 8.4 Hz, 3H), 7.16 – 7.10 (m, 6H), 6.96 (t, J = 7.5 Hz, 1H), 6.57 (d, J = 7.6 Hz, 1H), 5.99 (s, 1H), 4.81 (q, J = 6.5 Hz, 1H), 3.16 (s, 1H), 2.40 (s, 3H), 1.48 (s, 3H). **^{13}C NMR** (126 MHz, $CDCl_3$, 25 °C) 146.3, 145.7, 144.0, 138.8, 136.5, 136.2, 131.0, 130.6, 129.8, 129.8, 129.8, 128.0, 127.8, 127.6, 127.3, 126.3, 125.9, 125.5, 125.3, 124.2, 120.9, 115.2, 73.9, 67.0, 21.9, 21.8; **IR (ATR)**: 3739, 2257, 2922, 2352, 1653, 1446, 1378, 1180, 1076 ,743 cm^{-1}; **HRMS** (ESI, m/z): calcd for. $C_{42}H_{37}N_2O_4S_2^+$ $(M+H)^+$: 697.2189; Found: 697.2182; **HPLC** (IA-3 column, n-hexane/^{i}PrOH = 70/30, 1.0 mL/min, 25 °C, 220 nm) indicated 90% ee: t_R (minor) = 5.46 min, t_R (major) = 40.78 min.

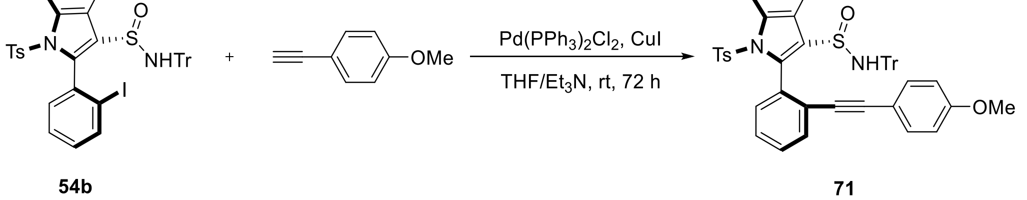

54b **71**

To a 5 mL Schlenk tube was added **54b** (37.9 mg, 0.05 mmol, 1.0 equiv), $Pd(PPh_3)_2Cl_2$ (2.8 mg, 0.004 mmol, 8 mol%.), CuI (1.6 mg, 0.008 mmol, 16 mol%) and THF (0.25 mL) under Ar. Then 4-ethynylanisole (13.2 mg, 0.1 mmol, 2.0 equiv.) and

Et_3N (0.25 mL) werw added. The mixture was stirred at room temperature for 72 h. Afterwards, the mixture was through a short plug of diatomite eluting with ethyl acetate (ca. 10 mL) and concentrated. The crude product was purified with flash column chromatography on silica gel (SiliaFlash® P60, particle size 40-63 μm, 230-400 mesh, Silicycle, PE/EtOAc with 1% Et_3N as eluent) to afford the corresponding product **71** as a light yellow oil (26.2 mg, 67%, >15/1 dr, 93% ee). $\mathbf{R_f}$ = 0.35 (PE/EA = 4/1); $[\alpha]^{25}_D$ = +32.7 (c = 0.6, CH_2Cl_2). **^{1}H NMR** (500 MHz, $CDCl_3$, 25 °C) δ 8.37 (d, J = 8.5 Hz, 1H), 8.07 (d, J = 7.9 Hz, 1H), 7.53 (d, J = 7.9 Hz, 1H), 7.50 – 7.44 (m, 4H), 7.42 (d, J = 7.7 Hz, 1H), 7.37 – 7.33 (m, 1H), 7.30 (dd, J = 7.6, 1.5 Hz, 1H), 7.18 (d, J = 7.3 Hz, 3H), 7.14 (t, J = 7.9 Hz, 6H), 7.02 (d, J = 7.6 Hz, 6H), 6.93 (d, J = 8.1 Hz, 2H), 6.70 (d, J = 8.8 Hz, 2H), 6.56 (d, J = 8.9 Hz, 2H), 5.84 (s, 1H), 3.71 (s, 3H), 2.12 (s, 3H); **^{13}C NMR** (126 MHz, $CDCl_3$, 25 °C) 159.7, 145.2, 144.2, 137.0, 136.5, 135.2, 133.1, 132.8, 132.0, 131.0, 129.7, 129.7, 129.6, 127.9, 127.8, 127.3, 127.3, 127.1, 125.8, 125.7, 125.3, 124.4, 120.7, 115.5, 114.7, 113.8, 94.5, 87.1, 73.4, 55.3, 21.6. **IR (ATR)**: 3739, 3308, 3056, 2925, 2351, 1601, 1508, 1446, 1376, 1250, 1177, 1081, 748 cm^{-1}; **HRMS** (ESI, m/z): calcd for. $C_{49}H_{38}N_2O_4S_2Na^+$ $(M+Na)^+$: 805.2165; Found: 805.2157; **HPLC** (IA-3 column, n-hexane/^{i}PrOH = 85/15, 0.7 mL/min, 25 ºC, 220 nm) indicated 93% ee: t_R (minor) = 16.01 min, t_R (major) = 27.06 min.

5 Mechanistic Studies

5.1 Synthesis of copper complex and testing its catalytic activity:

$$\text{CuBr} + \textbf{L5} \xrightarrow[\text{40 °C, 3 h}]{\text{1,3-dioxolane}} \text{CuBr}\bullet\textbf{L5}$$

In a nitrogen-filled glove box, to an oven-dried 8 mL screw cap vial equipped with a magnetic stir bar was added CuBr (5.7 mg, 0.05 mmol, 1.0 equiv.), **L5** (35.1 mg, 0.05 mmol, 1.0 equiv.), and anhydrous 1,3-dioxolane (1 mL). The tube was sealed with a teflon-lined screw cap, removed from the glove box and the reaction was stirred at 40 °C for 3 hours. Afterwards, the mixture was cooled to room temperature. The solvent

was evaporated under reduced pressure. Product **CuBr·L5** (yellow solid, 30.2 mg, 76% yield) can be obtained by column chromatography on silica gel (Et_2O/DCM = 1/20). **R_f** = 0.95 (Et_2O/DCM = 1/20); NMR spectroscopy: **^{1}H NMR** (600 MHz, $CDCl_3$, 25 ºC) δ 7.70 (d, *J* = 11.3 Hz, 2H), 7.34 (d, *J* = 10.2 Hz, 2H), 4.47 (s, 1H), 4.36 (s, 1H), 4.32 – 4.23 (m, 1H), 3.76 (s, 5H), 3.68 (s, 3H), 3.65 (s, 3H), 3.48 (d, *J* = 10.5 Hz, 1H), 1.88 (q, *J* = 12.1, 11.4 Hz, 4H), 1.78 (s, 2H), 1.68 (dd, *J* = 56.9, 11.5 Hz, 4H), 1.56 – 1.47 (m, 7H), 1.44 (s, 18H), 1.34 (s, 21H), 1.23 – 1.18 (m, 1H), 1.13 (q, *J* = 12.1 Hz, 1H), 1.02 (p, *J* = 12.6, 11.3 Hz, 2H), 0.81 (q, *J* = 12.8 Hz, 1H); **^{13}C NMR** (151 MHz, $CDCl_3$, 25 ºC) δ 161.5, 160.5, 143.8 (d, *J* = 10.3 Hz), 143.6 (d, *J* = 8.8 Hz), 133.6 (d, *J* = 19.3 Hz), 131.5 (d, *J* = 16.3 Hz), 130.1 (dd, *J* = 28.3, 8.6 Hz), 93.7 (dd, *J* = 22.0, 5.1 Hz), 74.2, 70.9, 70.2, 68.8, 64.7, 64.4, 36.2, 36.1, 32.6 (d, *J* = 7.1 Hz), 32.2, 32.2, 30.5, 28.8, 27.9 (d, *J* = 12.3 Hz), 27.6 (d, *J* = 9.3 Hz), 27.2 (d, *J* = 11.5 Hz), 27.0 (d, *J* = 11.9 Hz), 26.0, 16.1; **^{31}P NMR** (162 MHz, $CDCl_3$) δ 5.1 (d, *J* = 191.6 Hz), -22.1 (d, *J* = 190.6 Hz)

Note: The ^{31}P NMR and mass spectrometry indicate the possible presence of both monomeric and dimeric complex modes in the copper(I) phosphine complexes **CuBr·L5** , but it is not feasible to distinguish between the two complexes through NMR and HRMS. The appearance of this spectrum may come from the dynamic behavior in solution of copper(I) phosphine complexes. According to Feringa's work,[10] we speculate that copper(I) phosphine complexes initially exist in the form of dimers in 1,3-dioxolane (A type of ether solvent), which will be converted into monomers and participate in catalytic cycling processes in subsequent reactions.

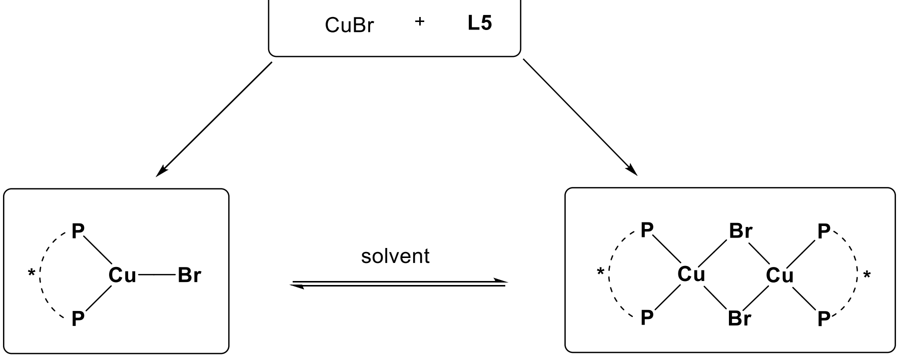

^{31}P NMR of CuBr·L5

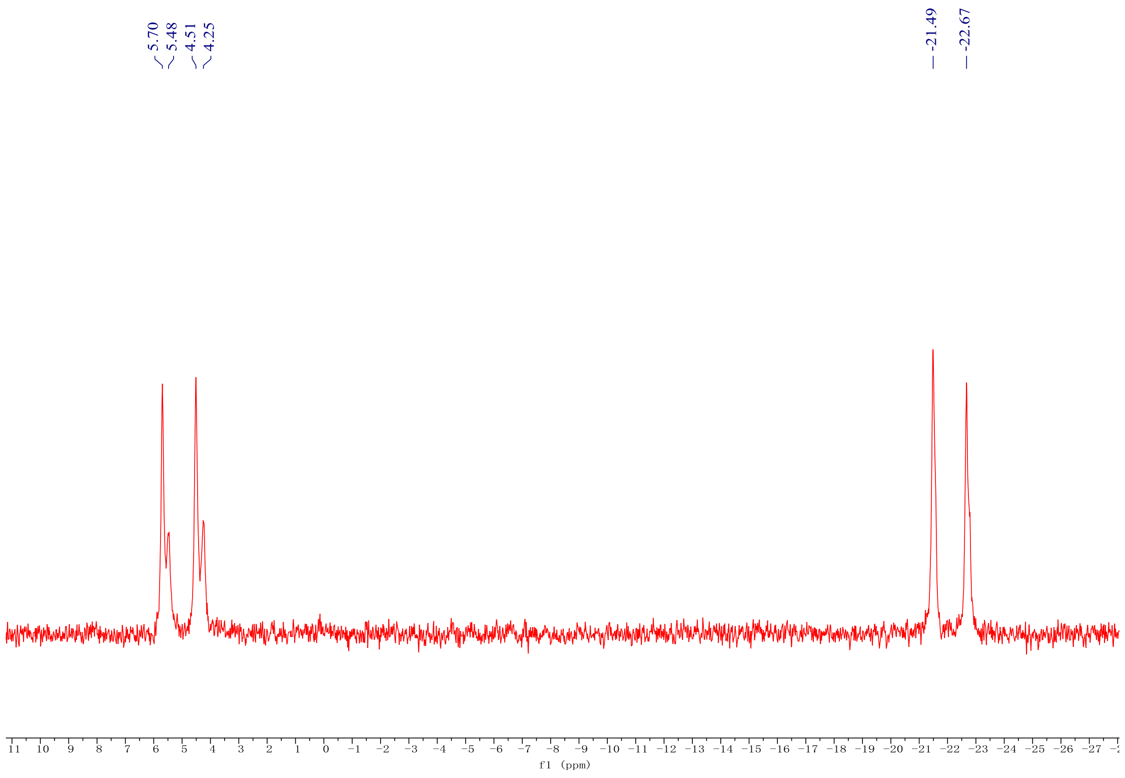

HRMS of CuBr·L5

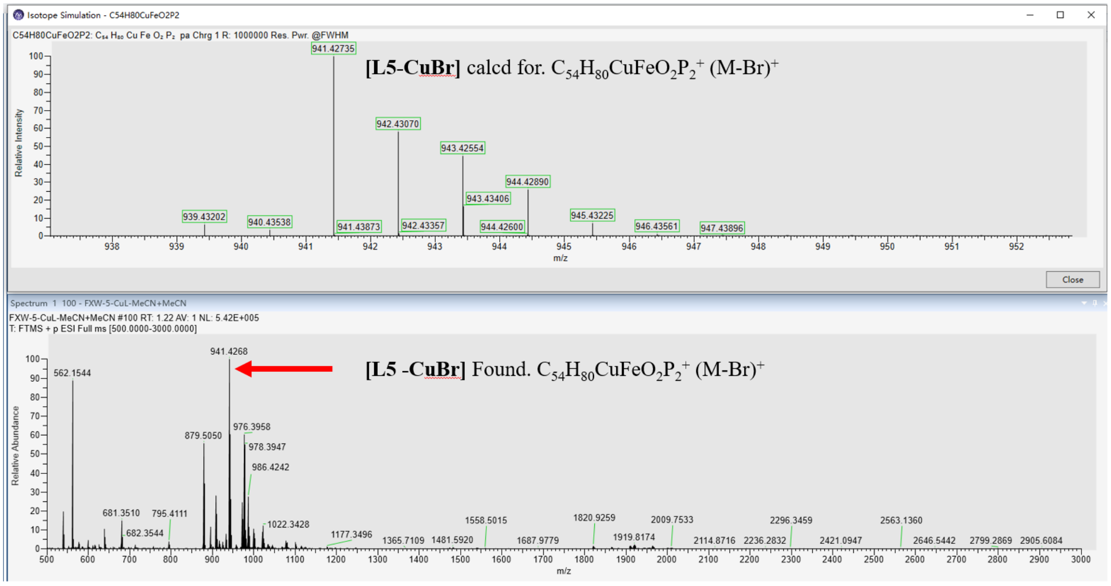

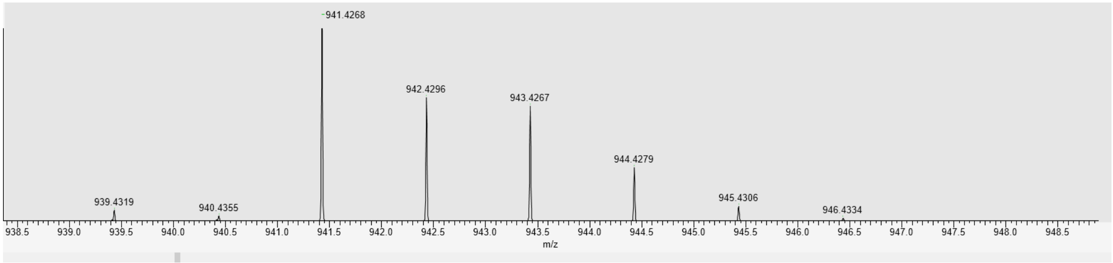

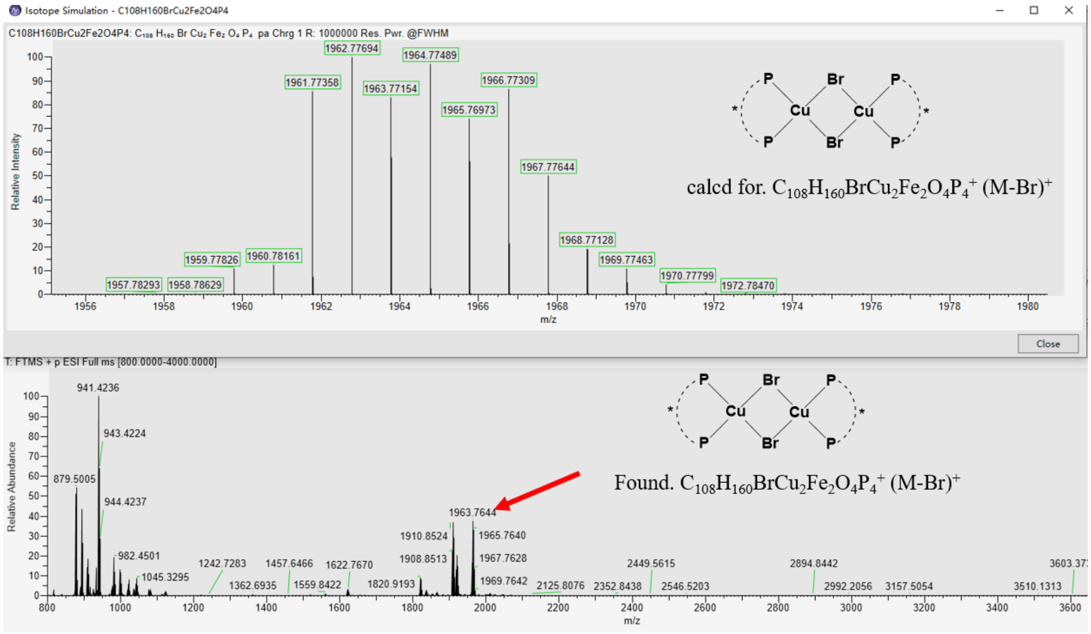

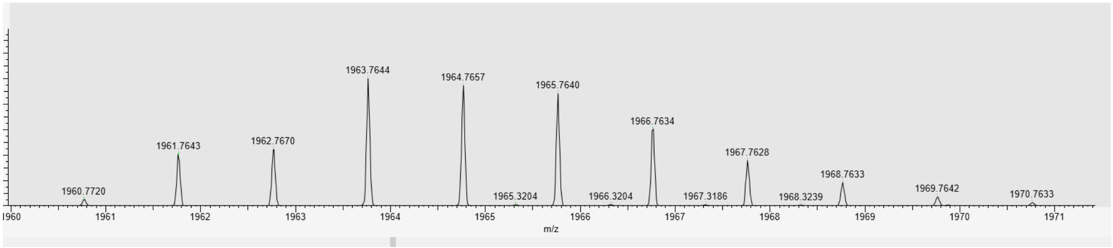

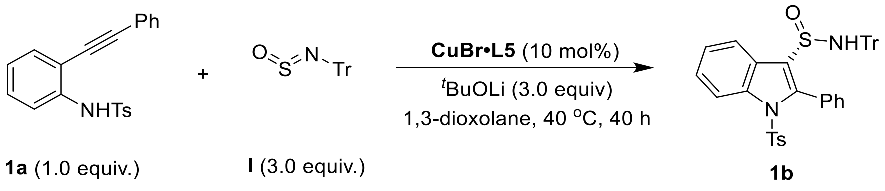

In a nitrogen-filled glove box, to an oven-dried 8 mL screw cap vial equipped with a magnetic stir bar was added **CuBr.L5** (10.2 mg, 0.01 mmol, 10 mol%), **1a** (34.7 mg, 0.1 mmol, 1.0 equiv.), **I** (91.5 mg, 0.3 mmol, 3.0 equiv.), ^{t}BuOLi (24 mg, 0.3 mmol, 3.0 equiv.) and anhydrous 1,3-dioxolane (1 mL). The tube was sealed with a teflon-lined screw cap, removed from the glove box and the reaction was stirred at 40 °C for 40 hours. Afterwards, the mixture was cooled to room temperature. The solvent was

evaporated under reduced pressure. The crude product was added DCM (~1 mL) and purified by column chromatography on silica gel (SiliaFlash® P60, particle size 40-63 µm, 230-400 mesh, Silicycle, PE/EtOAc with 1% Et_3N as eluent) to afford the corresponding product **1b** (88% yield, 94% ee).

5.2 Nonlinear effect

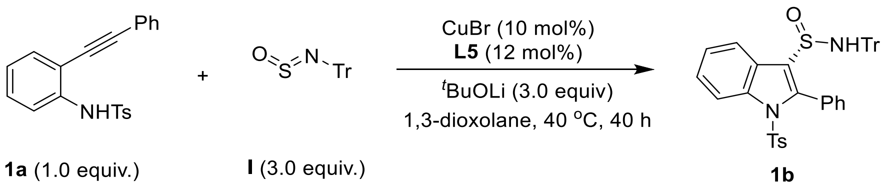

In a nitrogen-filled glove box, to an oven-dried 8 mL screw cap vial equipped with a magnetic stir bar was added CuBr (1.4 mg, 0.010 mmol, 10 mol%), **(*Sp, R*)-L5** + **(*Rp, S*)-L5** (10.5 mg, 0.012 mmol, 12 mol%), **1a** (34.7 mg, 0.1 mmol, 1.0 equiv.), **I** (91.5 mg, 0.3 mmol, 3.0 equiv.), ^{t}BuOLi (24 mg, 0.3 mmol, 3.0 equiv.) and anhydrous 1,3-dioxolane (1 mL). The tube was sealed with a teflon-lined screw cap, removed from the glove box and the reaction was stirred at 40 °C for 40 hours. Afterwards, the mixture was cooled to room temperature. The solvent was evaporated under reduced pressure. The crude product was added DCM (~1 mL) and purified by column chromatography on silica gel (SiliaFlash® P60, particle size 40-63 µm, 230-400 mesh, Silicycle, PE/EtOAc with 1% Et_3N as eluent) to afford the corresponding product **1b**. The enantiomeric excess (ee) was determined by chiral HPLC.

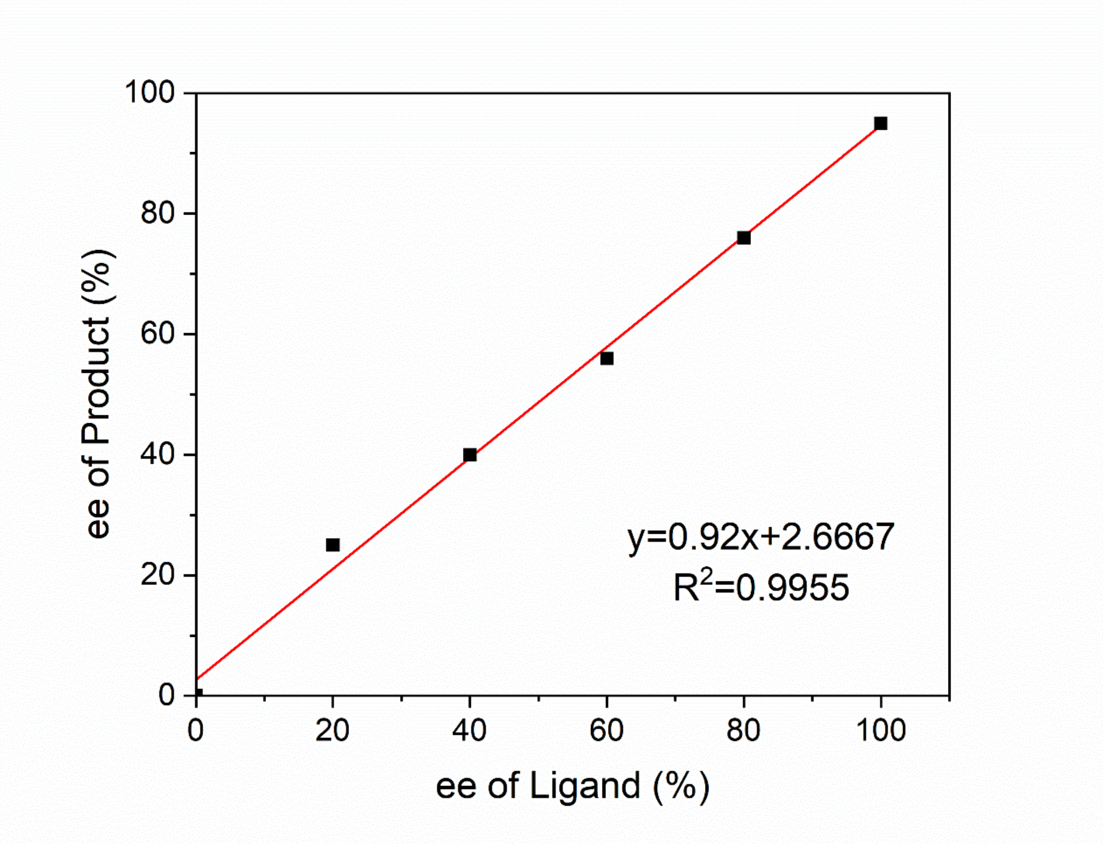

Figure S1. Nonlinear effect study

5.3 Direct sulfinamidation using indole 72:

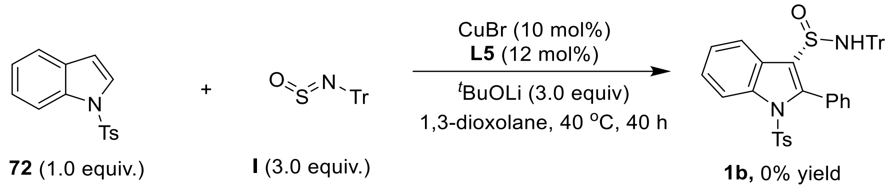

In a nitrogen-filled glove box, to an oven-dried 8 mL screw cap vial equipped with a magnetic stir bar was added CuBr (1.4 mg, 0.010 mmol, 10 mol%), **L5** (10.5 mg, 0.012 mmol, 12 mol%), **72** (0.1 mmol, 1.0 equiv.), **I** (91.5 mg, 0.3 mmol, 3.0 equiv.), ^{t}BuOLi (24 mg, 0.3 mmol, 3.0 equiv.) and anhydrous 1,3-dioxolane (1 mL). The tube was sealed with a teflon-lined screw cap, removed from the glove box and the reaction was stirred at 40 °C for 40 hours. No product was obtained through TLC detection.

5.4 Radical quenching experiments:

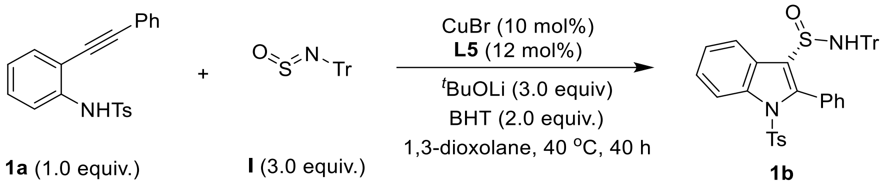

In a nitrogen-filled glove box, to an oven-dried 8 mL screw cap vial equipped with a magnetic stir bar was added CuBr (1.4 mg, 0.010 mmol, 10 mol%), **L5** (10.5 mg, 0.012 mmol, 12 mol%), **1a** (34.7 mg, 0.1 mmol, 1.0 equiv.), **I** (91.5 mg, 0.3 mmol, 3.0 equiv.), ^{t}BuOLi (24 mg, 0.3 mmol, 3.0 equiv.), BHT (44 mg, 0.2 mmol, 2.0 equiv.) and anhydrous 1,3-dioxolane (1 mL). The tube was sealed with a teflon-lined screw cap, removed from the glove box and the reaction was stirred at 40 °C for 40 hours. Afterwards, the mixture was cooled to room temperature. The solvent was evaporated under reduced pressure. The crude product was added DCM (~1 mL) and purified by column chromatography on silica gel (SiliaFlash® P60, particle size 40-63 µm, 230-400 mesh, Silicycle, PE/EtOAc with 1% Et_3N as eluent) to afford the corresponding product **1b** (87% yield, 95% ee).

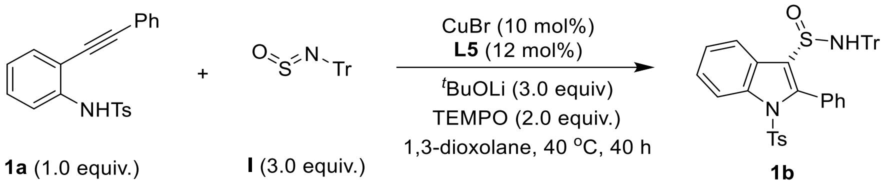

In a nitrogen-filled glove box, to an oven-dried 8 mL screw cap vial equipped with a magnetic stir bar was added CuBr (1.4 mg, 0.010 mmol, 10 mol%), **L5** (10.5 mg, 0.012 mmol, 12 mol%), **1a** (34.7 mg, 0.1 mmol, 1.0 equiv.), **I** (91.5 mg, 0.3 mmol, 3.0 equiv.), ^{t}BuOLi (24 mg, 0.3 mmol, 3.0 equiv.), TEMPO (31.2 mg, 0.2 mmol, 2.0 equiv.) and anhydrous 1,3-dioxolane (1 mL). The tube was sealed with a teflon-lined screw cap, removed from the glove box and the reaction was stirred at 40 °C for 40 hours. Afterwards, the mixture was cooled to room temperature. The solvent was evaporated under reduced pressure. The crude product was added DCM (~1 mL) and purified by column chromatography on silica gel (SiliaFlash® P60, particle size 40-63 µm, 230-400 mesh, Silicycle, PE/EtOAc with 1% Et_3N as eluent) to afford the corresponding product

1b (90% yield, 95% ee).

5.5 Testing different *N*-substituents in ortho-alkynylanilines:

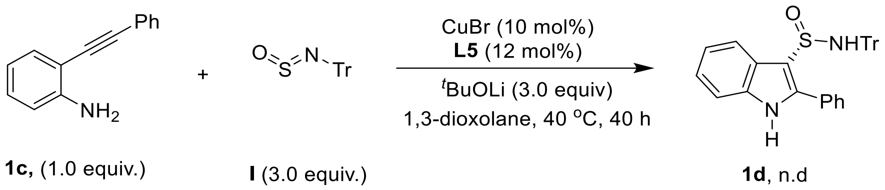

In a nitrogen-filled glove box, to an oven-dried 8 mL screw cap vial equipped with a magnetic stir bar was added CuBr (1.4 mg, 0.010 mmol, 10 mol%), **L5** (10.5 mg, 0.012 mmol, 12 mol%), **1c** (19.3 mg, 0.1 mmol, 1.0 equiv.), **I** (91.5 mg, 0.3 mmol, 3.0 equiv.), ^{t}BuOLi (24 mg, 0.3 mmol, 3.0 equiv.) and anhydrous 1,3-dioxolane (1 mL). The tube was sealed with a teflon-lined screw cap, removed from the glove box and the reaction was stirred at 40 °C for 40 hours. Afterwards, the mixture was cooled to room temperature. The solvent was evaporated under reduced pressure. Product **1d** cannot be obtained.

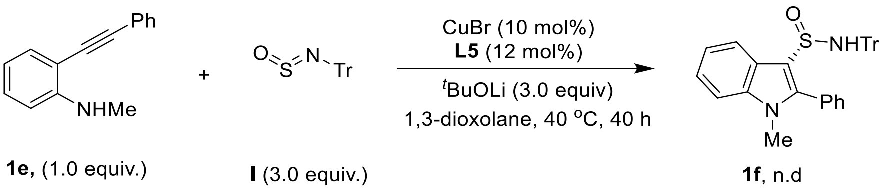

In a nitrogen-filled glove box, to an oven-dried 8 mL screw cap vial equipped with a magnetic stir bar was added CuBr (1.4 mg, 0.010 mmol, 10 mol%), **L5** (10.5 mg, 0.012 mmol, 12 mol%), **1e** (20.7 mg, 0.1 mmol, 1.0 equiv.), **I** (91.5 mg, 0.3 mmol, 3.0 equiv.), ^{t}BuOLi (24 mg, 0.3 mmol, 3.0 equiv.) and anhydrous 1,3-dioxolane (1 mL). The tube was sealed with a teflon-lined screw cap, removed from the glove box and the reaction was stirred at 40 °C for 40 hours. Afterwards, the mixture was cooled to room temperature. The solvent was evaporated under reduced pressure. Product **1f** cannot be obtained.

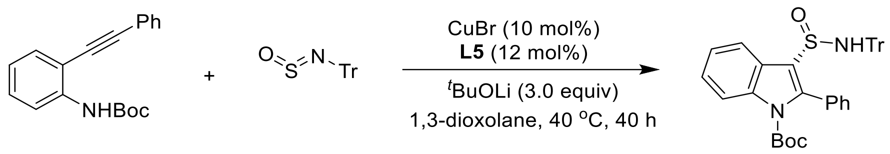

1g, (1.0 equiv.)   **I** (3.0 equiv.)   **1h**, n.d

In a nitrogen-filled glove box, to an oven-dried 8 mL screw cap vial equipped with a magnetic stir bar was added CuBr (1.4 mg, 0.010 mmol, 10 mol%), **L5** (10.5 mg, 0.012 mmol, 12 mol%), **1g** (29.3 mg, 0.1 mmol, 1.0 equiv.), **I** (91.5 mg, 0.3 mmol, 3.0 equiv.), ^{t}BuOLi (24 mg, 0.3 mmol, 3.0 equiv.) and anhydrous 1,3-dioxolane (1 mL). The tube was sealed with a teflon-lined screw cap, removed from the glove box and the reaction was stirred at 40 °C for 40 hours. Afterwards, the mixture was cooled to room temperature. The solvent was evaporated under reduced pressure. Product **1f** cannot be obtained.

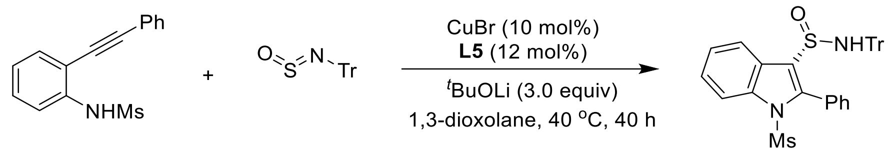

1i (1.0 equiv.)   **I** (3.0 equiv.)   **1j**, 8% yield

In a nitrogen-filled glove box, to an oven-dried 8 mL screw cap vial equipped with a magnetic stir bar was added CuBr (1.4 mg, 0.010 mmol, 10 mol%), **L5** (10.5 mg, 0.012 mmol, 12 mol%), **1i** (27.1 mg, 0.1 mmol, 1.0 equiv.), **I** (91.5 mg, 0.3 mmol, 3.0 equiv.), ^{t}BuOLi (24 mg, 0.3 mmol, 3.0 equiv.) and anhydrous 1,3-dioxolane (1 mL). The tube was sealed with a teflon-lined screw cap, removed from the glove box and the reaction was stirred at 40 °C for 40 hours. Afterwards, the mixture was cooled to room temperature. The solvent was evaporated under reduced pressure. Product **1j** can be obtained with 8% yield (determined by ^{1}HNMR with mesitylene as an internal standard).

5.6 Testing different *N*-substituents in sulfinylamines:

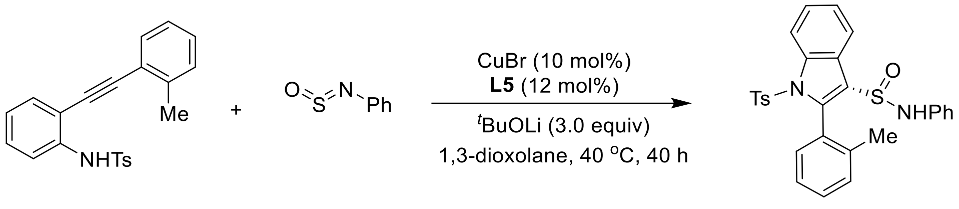

32a (1.0 equiv.) **III** (3.0 equiv.) **32c**: 68% yield, 1.2/1 dr

In a nitrogen-filled glove box, to an oven-dried 8 mL screw cap vial equipped with a magnetic stir bar was added CuBr (1.4 mg, 0.010 mmol, 10 mol%), **L5** (10.5 mg, 0.012 mmol, 12 mol%), **32a** (36.1 mg, 0.1 mmol, 1.0 equiv.), **II** (41.7 mg, 0.3 mmol, 3.0 equiv.), ^{t}BuOLi (24 mg, 0.3 mmol, 3.0 equiv.) and anhydrous 1,3-dioxolane (1 mL). The tube was sealed with a teflon-lined screw cap, removed from the glove box and the reaction was stirred at 40 °C for 40 hours. Afterwards, the mixture was cooled to room temperature. The solvent was evaporated under reduced pressure. Product **32c** White solid, 34.2 mg, 68% yield, 1.2/1 dr, 62% ee of major, 38% ee of minor) can be obtained by column chromatography on silica gel (SiliaFlash® P60, particle size 40-63 µm, 230-400 mesh, Silicycle, PE/EtOAc with 1% Et_3N as eluent). $\mathbf{R_f}$ = 0.5 (PE/EA = 2/1); $[\alpha]^{25}_D$ = 22.6 (*c* = 1.45, CH_2Cl_2); NMR spectroscopy: **^{1}H NMR** (500 MHz, $CDCl_3$, 25 ºC) δ 8.43 (d, *J* = 8.5 Hz, 1H, major + minor), 8.33 (d, *J* = 7.9 Hz, 0.47H, minor), 8.30 (d, *J* = 7.9 Hz, 0.53H, major), 7.53 – 7.47 (m, 1H, major + minor), 7.46 – 7.38 (m, 4H, major + minor), 7.31 (d, *J* = 7.7 Hz, 0.53H, major), 7.24 (t, *J* = 7.4 Hz, 1H, major + minor), 7.21 – 7.14 (m, 4H, major + minor), 7.14 (s, 0.47H, minor), 7.05 (d, *J* = 7.2 Hz, 0.47H, minor), 6.98 (q, *J* = 7.4 Hz, 1H, major + minor), 6.93 (d, *J* = 8.9 Hz, 0.53H, major), 6.75 (d, *J* = 7.8 Hz, 1H, major + minor), 6.70 (d, *J* = 7.9 Hz, 1H, major + minor), 6.36 (s, 1H, minor), 6.34 (s, 1H, major), 2.38 (s, 1.41H, minor), 2.36 (s, 1.59H, major), 2.15 (s, 1.59H, major), 2.06 (s, 1.41H, minor); **^{13}C NMR** (126 MHz, $CDCl_3$, 25 ºC) δ 145.9 (minor), 145.7 (major), 140.4 (major), 140.3 (minor), 140.3 (major), 139.6 (major), 139.5 (major), 138.9 (major), 136.7 (minor), 136.7 (major), 135.6 (major), 135.6 (minor), 131.9 (major), 130.7 (minor), 130.5 (major + minor), 130.1 (major), 130.0 (minor), 129.9 (minor), 129.8 (major), 129.5 (major), 129.5 (minor), 128.5 (minor), 128.3 (major), 127.3 (major), 127.3 (minor), 126.1 (minor), 126.1 (major), 125.1

(minor), 125.0 (major), 124.9 (major), 124.9 (major), 124.8 (minor), 124.7 (major), 124.6 (major + minor), 123.6 (major), 123.6 (major), 120.7 (major), 120.6 (minor), 119.0 (major), 118.8 (minor), 115.7 (major + minor), 21.8 (major + minor), 20.4 (major), 20.3 (minor); **IR (ATR)**: 3739, 3180, 3057, 2926, 2352, 1594, 1490, 1377, 1178, 1072, 751 cm^{-1}; **HRMS** (ESI, m/z): calcd for. $C_{28}H_{24}N_2O_3S_2Na^+$ $(M+Na)^+$: 523.1121; Found: 523.1118; **HPLC** (IA-3 column, *n*-hexane/^{i}PrOH = 70/30, 1.0 mL/min, 25 ºC, 220 nm) indicated 62% ee of major: t_R (minor) = 7.71 min, t_R (major) = 74.63 min; 38% ee of minor: t_R (major) = 10.81 min, t_R (minor) = 19.03 min.

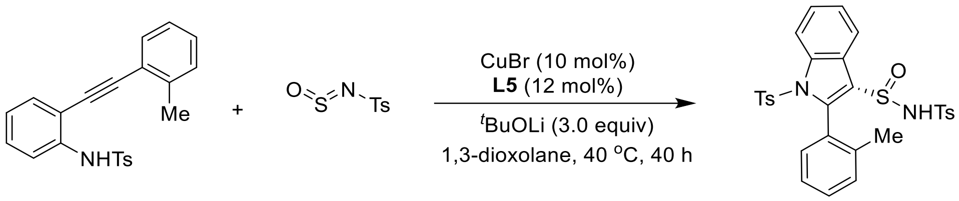

32a (1.0 equiv.) **III** (3.0 equiv.) **32d**: n.d.

In a nitrogen-filled glove box, to an oven-dried 8 mL screw cap vial equipped with a magnetic stir bar was added CuBr (1.4 mg, 0.010 mmol, 10 mol%), **L5** (10.5 mg, 0.012 mmol, 12 mol%), **32a** (36.1 mg, 0.1 mmol, 1.0 equiv.), **III** (65.1 mg, 0.3 mmol, 3.0 equiv.), ^{t}BuOLi (24 mg, 0.3 mmol, 3.0 equiv.) and anhydrous 1,3-dioxolane (1 mL). The tube was sealed with a teflon-lined screw cap, removed from the glove box and the reaction was stirred at 40 °C for 40 hours. Afterwards, the mixture was cooled to room temperature. The solvent was evaporated under reduced pressure. Product **32d** cannot be obtained.

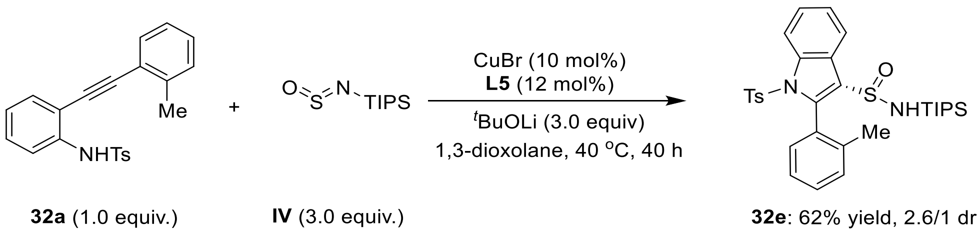

32a (1.0 equiv.) **IV** (3.0 equiv.) **32e**: 62% yield, 2.6/1 dr

In a nitrogen-filled glove box, to an oven-dried 8 mL screw cap vial equipped with a magnetic stir bar was added CuBr (1.4 mg, 0.010 mmol, 10 mol%), **L5** (10.5 mg, 0.012 mmol, 12 mol%), **32a** (36.1 mg, 0.1 mmol, 1.0 equiv.), **IV** (65.7 mg, 0.3 mmol, 3.0 equiv.), ^{t}BuOLi (24 mg, 0.3 mmol, 3.0 equiv.) and anhydrous 1,3-dioxolane (1 mL).

The tube was sealed with a teflon-lined screw cap, removed from the glove box and the reaction was stirred at 40 °C for 40 hours. Afterwards, the mixture was cooled to room temperature. The solvent was evaporated under reduced pressure. Product **32e** (White solid, 36.3 mg, 62% yield, 2.6/1 dr, 96% ee of major, 26% ee of minor) can be obtained by column chromatography on silica gel (SiliaFlash® P60, particle size 40-63 µm, 230-400 mesh, Silicycle, PE/EtOAc with 1% Et_3N as eluent). Using DCM/MeOH=100/1 as mobile phase can distinguish two isomers.

Minor: $\mathbf{R_f}$ = 0.6 (DCM/MeOH = 100/1); $[\alpha]^{25}_{D}$ = 2.29 (*c* = 0.7, CH_2Cl_2); NMR spectroscopy: **^{1}H NMR** (500 MHz, $CDCl_3$, 25 ºC) δ 8.39 (d, *J* = 8.5 Hz, 1H), 8.28 (d, *J* = 7.8 Hz, 1H), 7.49 – 7.45 (m, 1H), 7.43 – 7.36 (m, 4H), 7.28 (s, 1H), 7.22 (t, *J* = 7.5 Hz, 1H), 7.14 (d, *J* = 8.4 Hz, 3H), 4.39 (s, 1H), 2.35 (s, 3H), 2.25 (s, 3H), 1.02 – 0.95 (m, 3H), 0.93 (d, *J* = 7.0 Hz, 9H), 0.84 (d, *J* = 7.1 Hz, 9H); **^{13}C NMR** (126 MHz, $CDCl_3$, 25 ºC) δ 145.7, 140.5, 137.7, 137.0, 135.3, 130.8, 130.3, 130.0, 129.9, 129.7, 129.0, 127.2, 125.9, 125.2, 124.7, 124.3, 120.3, 116.1, 21.8, 20.9, 17.8, 17.8, 11.8; **IR (ATR)**: 3739, 3189, 2933, 2352, 1680, 1453, 1880, 1180, 1072, 862, 756, 570 cm^{-1}; **HRMS** (ESI, m/z): calcd for. $C_{31}H_{40}N_2O_3S_2SiNa^+$ $(M+Na)^+$: 603.2142; Found: 603.2135; **HPLC** analysis (IA-3 column, *n*-hexane/^{i}PrOH = 85/15, 0.7 mL/min, 25 ºC, 220 nm) indicated 26% ee: t_R (minor) = 8.03 min, t_R (major) = 10.19 min.

Major: $\mathbf{R_f}$ = 0.55 (DCM/MeOH = 100/1); $[\alpha]^{25}_{D}$ = 16.0 (*c* = 1.05, CH_2Cl_2); NMR spectroscopy: **^{1}H NMR** (500 MHz, $CDCl_3$, 25 ºC) δ 8.43 (d, *J* = 8.5 Hz, 1H), 8.21 (d, *J* = 7.8 Hz, 1H), 7.48 (t, *J* = 7.8 Hz, 1H), 7.43 – 7.34 (m, 4H), 7.25 – 7.18 (m, 3H), 7.13 (d, *J* = 8.0 Hz, 2H), 4.31 (s, 1H), 2.35 (s, 3H), 2.03 (s, 3H), 1.03 – 0.96 (m, 3H), 0.94 (d, *J* = 6.8 Hz, 9H), 0.87 (d, *J* = 7.0 Hz, 9H); **^{13}C NMR** (126 MHz, $CDCl_3$, 25 ºC) δ 145.6, 139.1, 137.0, 136.5, 135.8, 132.8, 130.3, 130.1, 129.8, 129.6, 128.5, 127.3, 125.9, 124.8, 124.5, 124.2, 120.1, 116.0, 21.8, 20.5, 17.9, 17.9, 11.8; **IR (ATR)**: 3739, 3318, 2865, 2352, 1452, 1379, 1180, 1074, 865, 757, 669, 570 cm^{-1}; **HRMS** (ESI, m/z): calcd for. $C_{31}H_{40}N_2O_3S_2SiNa^+$ $(M+Na)^+$: 603.2142; Found: 603.2136; **HPLC** analysis (IC-3 + IC column connected in series, *n*-hexane/^{i}PrOH = 95/5, 0.7 mL/min, 25 ºC, 220 nm) indicated 96% ee: t_R (minor) = 79.40 min, t_R (major) = 83.10 min.

6 Crystallographic Date

6.1 Crystallographic Date of 1b

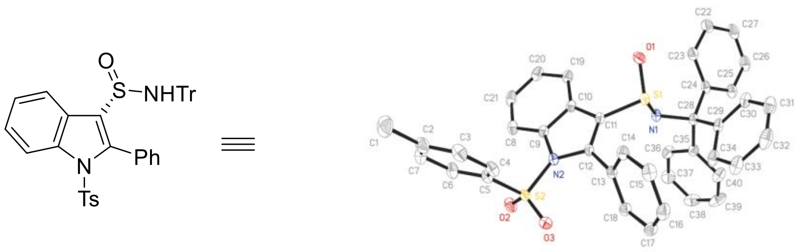

1b CCDC 2419186

Table S6. Crystal data and structure refinement for 1b

Identification code	**1b**
Empirical formula	$C_{40}H_{32}N_2O_3S_2$
Formula weight	652.79
Temperature/K	193.00
Crystal system	monoclinic
Space group	P21
a/Å	10.6159(2)
b/Å	9.77340(10)
c/Å	16.1304(2)
α/°	90
β/°	102.5580(10)
γ/°	90
Volume/Å^3	1633.55(4)
Z	2
ρ_{calc}g/cm^3	1.327
μ/mm^{-1}	1.814
F(000)	684.0
Crystal size/mm^3	0.15 × 0.13 × 0.12

Radiation	CuKα (λ = 1.54178)
2Θ range for data collection/°	5.612 to 144.682
Index ranges	-13 ≤ h ≤ 13, -12 ≤ k ≤ 10, -19 ≤ l ≤ 19
Reflections collected	33505
Independent reflections	6163 [R_{int} = 0.0275, R_{sigma} = 0.0274]
Data/restraints/parameters	6163/1/430
Goodness-of-fit on F^2	1.068
Final R indexes [I>=2σ (I)]	R_1 = 0.0246, wR_2 = 0.0634
Final R indexes [all data]	R_1 = 0.0249, wR_2 = 0.0635
Largest diff. peak/hole / e $Å^{-3}$	0.18/-0.37
Flack parameter	0.049(11)

Table S7. Fractional Atomic Coordinates (×10^4) and Equivalent Isotropic Displacement Parameters ($Å^2$×10^3) for 1b U_{eq} is defined as 1/3 of the trace of the orthogonalised U_{IJ} tensor.

**Atom**	***x***	***y***	***z***	**U(eq)**
S1	7520.4(4)	5755.8(4)	3563.4(2)	27.2(1)
C1	11344(3)	4468(4)	9397.0(18)	69.7(8)
O1	7773.2(13)	7248.9(15)	3669.4(8)	37.3(3)
N1	5988.9(14)	5430.7(16)	3061.3(9)	27.7(3)
N2	7232.9(14)	3752.2(16)	5642.8(9)	28.6(3)
O2	6257.6(14)	2261.2(17)	6571.4(9)	42.5(3)
S2	7469.1(4)	2441.7(5)	6334.6(3)	32.18(11)
C2	10374(2)	3955(3)	8641.8(13)	45.2(5)
O3	8028.0(16)	1351.0(16)	5956.6(9)	44.7(3)
C3	10760(2)	3561(3)	7900.6(14)	44.7(5)
C4	9880.3(19)	3099(2)	7194.2(13)	37.1(4)
C5	8583.7(18)	3036(2)	7229.3(12)	31.8(4)

C6	8176(2)	3389(3)	7961.7(13)	40.7(5)
C7	9078(2)	3838(3)	8660.4(13)	45.7(5)
C8	5933.8(18)	5283(2)	6430.9(12)	36.9(4)
C9	6548.5(16)	4942(2)	5773.7(11)	28.9(4)
C10	6588.0(15)	5853(2)	5110.5(10)	27.1(3)
C11	7296.0(15)	5174.5(18)	4563.2(10)	25.9(3)
C12	7712.3(16)	3936.1(19)	4894.9(10)	26.5(3)
C13	8531.3(17)	2964.0(19)	4537.5(10)	28.4(4)
C14	9741.3(18)	3423(2)	4456.0(12)	33.7(4)
C15	10512.5(19)	2598(2)	4069.7(13)	41.9(5)
C16	10081(2)	1326(3)	3765.1(13)	44.2(5)
C17	8882(2)	862(2)	3840.6(12)	40.1(4)
C18	8101.7(18)	1678(2)	4228.9(12)	34.0(4)
C19	6021.0(18)	7150(2)	5096.1(12)	33.4(4)
C20	5434.0(19)	7490(2)	5752.7(13)	40.7(5)
C21	5393.5(19)	6568(3)	6404.1(13)	41.8(5)
C22	5247(2)	9094(2)	1548.8(15)	45.8(5)
C23	5571.2(19)	7893(2)	2012.8(12)	36.0(4)
C24	5118.9(17)	6634(2)	1673.7(11)	30.7(4)
C25	4286.1(19)	6608(2)	869.2(12)	39.1(4)
C26	3965(2)	7805(3)	414.8(14)	47.5(6)
C27	4453(2)	9038(3)	744.0(14)	50.3(6)
C28	5589.7(17)	5276.7(19)	2118.1(11)	28.5(4)
C29	6770.1(17)	4744(2)	1803.5(11)	32.7(4)
C30	7368.0(18)	5525(2)	1281.4(12)	38.2(4)
C31	8464(2)	5032(3)	1029.1(15)	49.9(6)
C32	8963(2)	3771(3)	1298.7(15)	54.7(7)

C33	8382(2)	2986(3)	1831.5(14)	50.8(6)
C34	7294(2)	3470(3)	2080.5(12)	41.6(5)
C35	4459.2(17)	4248(2)	1948.0(11)	31.4(4)
C36	3467(2)	4389(2)	2379.2(14)	42.1(5)
C37	2403(2)	3528(3)	2224.9(16)	50.9(5)
C38	2315(2)	2498(3)	1643.9(17)	53.0(6)
C39	3262(2)	2351(3)	1203.4(18)	60.3(7)
C40	4337(2)	3216(3)	1349.3(15)	46.7(5)

Table S8. Anisotropic Displacement Parameters (Å^2×10^3) for 1b The Anisotropic displacement factor exponent takes the form: -2π^2[h^{2}a*^{2}U$_{11}$+2hka*b*U$_{12}$+…].

Atom	U_{11}	U_{22}	U_{33}	U_{23}	U_{13}	U_{12}
S1	25.77(19)	34.3(2)	22.14(19)	3.20(15)	6.53(14)	1.33(16)
C1	59.5(16)	91(2)	47.2(14)	3.3(14)	-13.1(12)	-13.1(15)
O1	39.4(7)	37.1(8)	34.4(7)	6.0(6)	5.8(5)	-8.5(6)
N1	26.3(7)	36.0(8)	21.2(7)	0.7(6)	6.4(5)	3.7(6)
N2	31.3(7)	32.8(8)	23.0(7)	2.5(6)	8.5(6)	0.0(6)
O2	39.6(7)	52.4(9)	35.3(7)	8.7(6)	7.5(6)	-14.9(7)
S2	38.3(2)	32.4(2)	26.4(2)	4.67(17)	8.24(17)	-4.51(17)
C2	43.4(11)	50.6(13)	35.9(11)	9.7(9)	-3.8(9)	-0.8(9)
O3	68.5(10)	31.3(7)	35.6(7)	3.5(6)	14.2(7)	2.4(7)
C3	30.5(9)	57.7(13)	44.5(11)	13.6(10)	4.7(8)	1.0(9)
C4	34.6(9)	43.9(11)	34.9(10)	10.8(8)	12.3(8)	6.1(8)
C5	33.0(9)	34.6(9)	28.0(9)	7.7(7)	7.4(7)	1.7(7)
C6	35.4(10)	56.7(13)	31.1(10)	2.7(9)	9.5(8)	3.5(9)
C7	45.9(11)	60.5(14)	29.9(10)	-1.1(9)	6.8(8)	3.5(10)
C8	33.2(9)	52.5(12)	27.0(9)	-0.7(8)	11.2(7)	1.7(8)
C9	26.1(8)	35.5(9)	25.0(8)	-2.4(7)	5.3(6)	-1.6(7)

C10	22.6(7)	34.7(9)	23.0(7)	-2.2(7)	2.8(6)	0.2(7)
C11	24.4(7)	31.3(9)	21.7(8)	-1.1(7)	4.7(6)	-1.2(7)
C12	26.3(8)	32.7(9)	20.4(7)	-0.1(7)	5.2(6)	-2.9(7)
C13	30.5(8)	32.3(9)	22.1(8)	3.2(7)	5.2(6)	5.6(7)
C14	31.1(9)	39.2(10)	29.6(9)	0.0(7)	4.0(7)	3.0(7)
C15	29.7(9)	55.5(13)	40.7(10)	3.9(9)	8.1(8)	8.7(9)
C16	44.3(11)	52.1(13)	36.7(11)	-1.3(9)	10.0(9)	21.2(10)
C17	49.6(11)	34.6(10)	34.6(10)	-1.4(8)	5.9(8)	10.4(9)
C18	35.1(9)	35.9(10)	30.7(9)	0.3(7)	6.7(7)	3.2(8)
C19	31.3(9)	39.0(10)	27.8(9)	-2.0(7)	1.6(7)	6.9(7)
C20	37.7(10)	46.6(12)	35.3(10)	-8.8(9)	2.5(8)	14.9(9)
C21	34.5(10)	61.8(14)	30.5(9)	-8.9(9)	10.3(8)	9.3(10)
C22	56.5(13)	39.0(11)	45.4(12)	7.7(9)	18.6(10)	12.8(10)
C23	38.9(10)	39.8(10)	30.3(9)	4.7(8)	9.6(7)	8.0(8)
C24	28.3(8)	40.4(10)	25.1(8)	4.6(7)	9.8(7)	7.7(7)
C25	38.0(10)	50.2(12)	28.5(9)	4.3(9)	5.9(8)	7.5(9)
C26	48.4(12)	63.6(15)	29.9(10)	12.3(10)	7.3(9)	17.5(10)
C27	64.2(14)	52.3(14)	38.1(11)	19.7(10)	19.2(10)	23.9(11)
C28	29.1(8)	35.4(9)	20.9(8)	0.5(7)	5.1(6)	5.3(7)
C29	29.6(9)	45.9(11)	21.6(8)	-4.3(7)	3.3(7)	8.1(8)
C30	34.2(9)	50.0(12)	31.3(9)	-7.1(8)	8.6(7)	3.5(8)
C31	36.7(10)	72.7(16)	43.8(12)	-11.4(11)	16.4(9)	0.5(11)
C32	34.1(10)	85.6(19)	43.2(12)	-18.0(12)	6.0(9)	18.5(11)
C33	47.6(12)	66.5(15)	33.8(10)	-6.6(10)	-1.1(9)	28.4(11)
C34	43.7(11)	52.4(12)	27.2(9)	-0.4(8)	4.7(8)	16.0(9)
C35	31.6(9)	33.0(10)	26.9(9)	0.7(7)	0.4(7)	6.1(7)
C36	36.2(10)	46.1(12)	44.6(11)	-12.6(9)	10.3(9)	-3.4(9)

C37	37.6(11)	59.8(14)	54.0(13)	-2.1(11)	7.2(9)	-6.1(10)
C38	39.9(11)	39.9(12)	69.6(15)	-0.3(11)	-9.0(10)	-1.8(9)
C39	51.6(13)	46.4(13)	71.8(17)	-26.0(13)	-11.1(12)	6.4(11)
C40	41.3(11)	53.3(14)	42.1(11)	-15.5(10)	2.0(9)	7.0(10)

Table S9. Bond Lengths for 1b

Atom	Atom	Length/Å	Atom	Atom	Length/Å
S1	O1	1.4869(16)	C15	C16	1.378(4)
S1	N1	1.6815(15)	C16	C17	1.381(3)
S1	C11	1.7741(17)	C17	C18	1.393(3)
C1	C2	1.500(3)	C19	C20	1.381(3)
N1	C28	1.496(2)	C20	C21	1.393(3)
N2	S2	1.6814(15)	C22	C23	1.395(3)
N2	C9	1.412(2)	C22	C27	1.387(3)
N2	C12	1.419(2)	C23	C24	1.389(3)
O2	S2	1.4300(14)	C24	C25	1.403(3)
S2	O3	1.4203(16)	C24	C28	1.538(2)
S2	C5	1.7544(19)	C25	C26	1.383(3)
C2	C3	1.399(3)	C26	C27	1.372(4)
C2	C7	1.387(3)	C28	C29	1.541(2)
C3	C4	1.382(3)	C28	C35	1.544(3)
C4	C5	1.391(3)	C29	C30	1.388(3)
C5	C6	1.387(3)	C29	C34	1.397(3)
C6	C7	1.383(3)	C30	C31	1.399(3)
C8	C9	1.400(3)	C31	C32	1.375(4)
C8	C21	1.377(3)	C32	C33	1.393(4)
C9	C10	1.399(3)	C33	C34	1.385(3)
C10	C11	1.439(2)	C35	C36	1.390(3)

C10	C19	1.401(3)	C35	C40	1.382(3)
C11	C12	1.358(3)	C36	C37	1.387(3)
C12	C13	1.486(2)	C37	C38	1.365(4)
C13	C14	1.393(3)	C38	C39	1.359(4)
C13	C18	1.391(3)	C39	C40	1.399(4)
C14	C15	1.390(3)			

Table S10. Bond Angles for 1b

Atom	Atom	Atom	Angle/°	Atom	Atom	Atom	Angle/°
O1	S1	N1	111.86(8)	C15	C14	C13	120.03(19)
O1	S1	C11	105.45(8)	C16	C15	C14	120.05(19)
N1	S1	C11	94.04(7)	C15	C16	C17	120.40(19)
C28	N1	S1	122.64(11)	C16	C17	C18	120.1(2)
C9	N2	S2	122.40(12)	C13	C18	C17	119.83(18)
C9	N2	C12	108.32(14)	C20	C19	C10	117.97(19)
C12	N2	S2	129.16(13)	C19	C20	C21	121.0(2)
N2	S2	C5	105.69(8)	C8	C21	C20	122.22(18)
O2	S2	N2	104.97(8)	C27	C22	C23	119.8(2)
O2	S2	C5	107.82(9)	C24	C23	C22	120.72(18)
O3	S2	N2	107.62(8)	C23	C24	C25	118.31(18)
O3	S2	O2	120.40(10)	C23	C24	C28	122.05(15)
O3	S2	C5	109.37(10)	C25	C24	C28	119.45(18)
C3	C2	C1	120.6(2)	C26	C25	C24	120.6(2)
C7	C2	C1	121.3(2)	C27	C26	C25	120.6(2)
C7	C2	C3	118.1(2)	C26	C27	C22	119.9(2)
C4	C3	C2	121.6(2)	N1	C28	C24	112.26(15)
C3	C4	C5	118.54(19)	N1	C28	C29	107.35(13)
C4	C5	S2	118.53(15)	N1	C28	C35	106.48(14)

C6	C5	S2	120.21(15)	C24	C28	C29	110.04(15)
C6	C5	C4	121.23(18)	C24	C28	C35	108.51(14)
C7	C6	C5	118.93(19)	C29	C28	C35	112.20(15)
C6	C7	C2	121.5(2)	C30	C29	C28	121.73(18)
C21	C8	C9	116.88(19)	C30	C29	C34	118.77(18)
C8	C9	N2	130.44(18)	C34	C29	C28	119.43(18)
C10	C9	N2	107.92(15)	C29	C30	C31	120.4(2)
C10	C9	C8	121.63(18)	C32	C31	C30	120.3(2)
C9	C10	C11	106.42(16)	C31	C32	C33	119.8(2)
C9	C10	C19	120.24(16)	C34	C33	C32	120.0(2)
C19	C10	C11	133.32(17)	C33	C34	C29	120.7(2)
C10	C11	S1	126.96(14)	C36	C35	C28	119.40(17)
C12	C11	S1	123.17(13)	C40	C35	C28	123.38(18)
C12	C11	C10	109.72(15)	C40	C35	C36	117.13(19)
N2	C12	C13	126.66(16)	C37	C36	C35	121.8(2)
C11	C12	N2	107.56(15)	C38	C37	C36	120.1(2)
C11	C12	C13	125.78(15)	C39	C38	C37	119.3(2)
C14	C13	C12	117.37(17)	C38	C39	C40	121.2(2)
C18	C13	C12	122.83(16)	C35	C40	C39	120.5(2)
C18	C13	C14	119.62(17)				

Table S11. Torsion Angles for 1b

A	B	C	D	Angle/°	A	B	C	D	Angle/°
S1	N1	C28	C24	-92.90(16)	C12	N2	S2	O2	-141.88(15)
S1	N1	C28	C29	28.1(2)	C12	N2	S2	O3	-12.49(18)
S1	N1	C28	C35	148.49(12)	C12	N2	S2	C5	104.29(16)
S1	C11	C12	N2	-173.03(12)	C12	N2	C9	C8	-177.76(19)
S1	C11	C12	C13	7.5(2)	C12	N2	C9	C10	0.63(19)

C1	C2	C3	C4	-179.3(2)	C12	C13	C14	C15	-175.34(17)
C1	C2	C7	C6	178.8(3)	C12	C13	C18	C17	174.87(16)
O1	S1	N1	C28	91.40(15)	C13	C14	C15	C16	0.1(3)
O1	S1	C11	C10	41.46(17)	C14	C13	C18	C17	-0.1(3)
O1	S1	C11	C12	-143.51(14)	C14	C15	C16	C17	0.0(3)
N1	S1	C11	C10	-72.55(16)	C15	C16	C17	C18	-0.2(3)
N1	S1	C11	C12	102.49(15)	C16	C17	C18	C13	0.2(3)
N1	C28	C29	C30	-114.70(18)	C18	C13	C14	C15	-0.1(3)
N1	C28	C29	C34	62.3(2)	C19	C10	C11	S1	-8.4(3)
N1	C28	C35	C36	46.0(2)	C19	C10	C11	C12	176.01(18)
N1	C28	C35	C40	-137.54(19)	C19	C20	C21	C8	0.2(3)
N2	S2	C5	C4	-77.43(17)	C21	C8	C9	N2	176.83(18)
N2	S2	C5	C6	104.65(17)	C21	C8	C9	C10	-1.4(3)
N2	C9	C10	C11	1.00(19)	C22	C23	C24	C25	-2.6(3)
N2	C9	C10	C19	-177.64(15)	C22	C23	C24	C28	172.23(18)
N2	C12	C13	C14	-119.87(19)	C23	C22	C27	C26	1.5(3)
N2	C12	C13	C18	65.1(2)	C23	C24	C25	C26	2.3(3)
O2	S2	C5	C4	170.72(16)	C23	C24	C28	N1	28.7(2)
O2	S2	C5	C6	-7.2(2)	C23	C24	C28	C29	-90.8(2)
S2	N2	C9	C8	-1.5(3)	C23	C24	C28	C35	146.12(17)
S2	N2	C9	C10	176.84(12)	C24	C25	C26	C27	-0.1(3)
S2	N2	C12	C11	-177.98(13)	C24	C28	C29	C30	7.7(2)
S2	N2	C12	C13	1.5(3)	C24	C28	C29	C34	-175.31(17)
S2	C5	C6	C7	179.21(18)	C24	C28	C35	C36	-75.1(2)
C2	C3	C4	C5	0.3(3)	C24	C28	C35	C40	101.4(2)
O3	S2	C5	C4	38.16(18)	C25	C24	C28	N1	-156.47(16)
O3	S2	C5	C6	-139.75(17)	C25	C24	C28	C29	84.04(19)

C3	C2	C7	C6	-2.0(4)	C25	C24	C28	C35	-39.1(2)
C3	C4	C5	S2	-179.69(16)	C25	C26	C27	C22	-1.8(3)
C3	C4	C5	C6	-1.8(3)	C27	C22	C23	C24	0.8(3)
C4	C5	C6	C7	1.4(3)	C28	C24	C25	C26	-172.66(18)
C5	C6	C7	C2	0.6(4)	C28	C29	C30	C31	177.88(18)
C7	C2	C3	C4	1.6(4)	C28	C29	C34	C33	-177.76(19)
C8	C9	C10	C11	179.56(16)	C28	C35	C36	C37	177.3(2)
C8	C9	C10	C19	0.9(3)	C28	C35	C40	C39	-177.5(2)
C9	N2	S2	O2	42.76(16)	C29	C28	C35	C36	163.14(18)
C9	N2	S2	O3	172.15(14)	C29	C28	C35	C40	-20.4(2)
C9	N2	S2	C5	-71.07(15)	C29	C30	C31	C32	-0.3(3)
C9	N2	C12	C11	-2.11(18)	C30	C29	C34	C33	-0.7(3)
C9	N2	C12	C13	177.39(16)	C30	C31	C32	C33	-0.5(3)
C9	C8	C21	C20	0.8(3)	C31	C32	C33	C34	0.7(4)
C9	C10	C11	S1	173.21(13)	C32	C33	C34	C29	-0.1(3)
C9	C10	C11	C12	-2.37(19)	C34	C29	C30	C31	0.9(3)
C9	C10	C19	C20	0.1(3)	C35	C28	C29	C30	128.65(18)
C10	C11	C12	N2	2.76(19)	C35	C28	C29	C34	-54.4(2)
C10	C11	C12	C13	-176.74(16)	C35	C36	C37	C38	0.8(4)
C10	C19	C20	C21	-0.7(3)	C36	C35	C40	C39	-0.9(3)
C11	S1	N1	C28	-160.18(14)	C36	C37	C38	C39	-1.8(4)
C11	C10	C19	C20	-178.06(19)	C37	C38	C39	C40	1.5(4)
C11	C12	C13	C14	59.5(2)	C38	C39	C40	C35	-0.1(4)
C11	C12	C13	C18	-115.5(2)	C40	C35	C36	C37	0.6(3)

Table S12. Hydrogen Atom Coordinates (Å×10^4) and Isotropic Displacement Parameters (Å^2×10^3) for 1b

Atom	*x*	*y*	*z*	U(eq)

H1A	12215.29	4363.77	9293.03	105
H1B	11272.79	3938.33	9900.65	105
H1C	11179.31	5435.81	9490.47	105
H1	5420(20)	5880(30)	3298(14)	34(5)
H3	11646.69	3613.17	7882.34	54
H4	10155.31	2831.14	6695.8	45
H6	7290.27	3323.36	7982.67	49
H7	8804.52	4072.51	9165.08	55
H8	5891.52	4656.33	6874.51	44
H14	10039.02	4300.68	4664.59	40
H15	11338.06	2910.54	4015.53	50
H16	10610.92	764.28	3501.64	53
H17	8588.91	-14.64	3627.41	48
H18	7278.43	1358.28	4282.88	41
H19	6039.92	7775.92	4648.48	40
H20	5051.77	8367.92	5759.91	49
H21	4979.17	6833.18	6844.97	50
H22	5569.86	9949.41	1783.51	55
H23	6107.05	7935.77	2566.61	43
H25	3939.67	5761.95	634.37	47
H26	3401.16	7773.25	-129.97	57
H27	4246.77	9851.43	421.03	60
H30	7030.96	6399.63	1094.31	46
H31	8864.23	5571.63	669.55	60
H32	9703.4	3435.76	1122.23	66
H33	8730.48	2118.51	2024.24	61
H34	6900.44	2929.17	2443.29	50

H36	3518.71	5093.62	2790.79	50
H37	1733.76	3655.92	2524.18	61
H38	1599.99	1889.42	1548.55	64
H39	3192.71	1648.84	788.82	72
H40	4988.76	3094.11	1034.74	56

6.2 Crystallographic Date of 32b

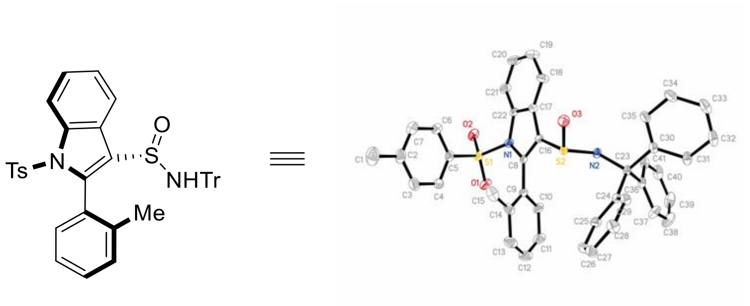

32b   CCDC 2436916

Table S13. Crystal data and structure refinement for 32b.

Identification code	**32b**
Empirical formula	$C_{42}H_{36}Cl_2N_2O_3S_2$
Formula weight	751.75
Temperature/K	193.00
Crystal system	monoclinic
Space group	$P2_1$
a/Å	9.9196(2)
b/Å	14.6757(3)
c/Å	12.9947(2)
α/°	90
β/°	97.6400(10)
γ/°	90
Volume/Å^3	1874.94(6)

Z	2
ρ_{calc}g/cm^3	1.332
μ/mm^{-1}	2.931
F(000)	784.0
Crystal size/mm^3	0.15 × 0.13 × 0.12
Radiation	CuKα (λ = 1.54178)
2Θ range for data collection/°	6.862 to 136.526
Index ranges	-11 ≤ h ≤ 11, -16 ≤ k ≤ 17, -15 ≤ l ≤ 14
Reflections collected	33005
Independent reflections	6691 [R_{int} = 0.0353, R_{sigma} = 0.0335]
Data/restraints/parameters	6691/58/491
Goodness-of-fit on F^2	1.072
Final R indexes [I>=2σ (I)]	R_1 = 0.0331, wR_2 = 0.0902
Final R indexes [all data]	R_1 = 0.0335, wR_2 = 0.0905
Largest diff. peak/hole / e Å^{-3}	0.75/-0.34
Flack parameter	0.073(14)

Table S14. Fractional Atomic Coordinates (×10^4) and Equivalent Isotropic Displacement Parameters (Å^2×10^3) for 32b. U_{eq} is defined as 1/3 of the trace of the orthogonalised U_{IJ} tensor.

Atom	*x*	*y*	*z*	U(eq)
C(1)	1225(6)	7683(3)	1403(4)	75.8(15)
N(1)	2020(2)	3977.7(17)	4226.7(16)	28.0(5)
O(1)	-514(2)	3928(2)	3755.0(16)	45.8(6)
S(1)	691.6(7)	3967.8(5)	3273.8(5)	31.41(17)
C(2)	1039(4)	6744(2)	1816(3)	49.8(9)
N(2)	4030(2)	4558.0(14)	7415.9(16)	24.6(5)
O(2)	980(2)	3262.6(16)	2582.0(17)	40.8(5)

S(2)	3984.4(6)	5400.8(4)	6555.6(5)	23.59(15)
C(3)	-11(4)	6553(3)	2390(3)	54.4(10)
O(3)	5356(2)	5716.4(15)	6381.2(17)	36.4(5)
C(4)	-150(3)	5707(3)	2827(2)	44.1(8)
C(5)	783(3)	5034(2)	2674(2)	33.1(6)
C(6)	1821(3)	5194(2)	2077(2)	37.1(7)
C(7)	1932(4)	6048(3)	1650(3)	47.0(8)
C(8)	2105(3)	4582.5(17)	5085.7(19)	23.4(5)
C(9)	908(3)	4948(2)	5515(2)	28.9(6)
C(10)	77(3)	4327(3)	5976(2)	40.3(7)
C(11)	-1043(3)	4636(3)	6404(3)	53.0(10)
C(12)	-1342(3)	5540(4)	6366(3)	57.3(11)
C(13)	-545(4)	6154(3)	5937(3)	52.7(10)
C(14)	625(3)	5874(2)	5499(2)	38.3(7)
C(15)	1478(4)	6571(2)	5076(3)	50.3(9)
C(16)	3436(3)	4705.0(19)	5455.0(19)	23.4(5)
C(17)	4264(3)	4215.3(19)	4810(2)	28.0(6)
C(18)	5672(3)	4124(2)	4831(3)	39.5(7)
C(19)	6136(4)	3585(3)	4075(3)	51.0(9)
C(20)	5249(4)	3141(3)	3336(3)	52.1(9)
C(21)	3851(4)	3216(2)	3304(3)	41.9(7)
C(22)	3377(3)	3775.0(19)	4043(2)	28.5(6)
C(23)	4095(3)	4733.3(18)	8557.4(19)	24.5(5)
C(24)	3367(3)	5643.8(17)	8672.9(19)	24.2(5)
C(25)	1982(3)	5728(2)	8307(2)	31.7(6)
C(26)	1324(3)	6550(2)	8356(2)	37.6(7)
C(27)	2030(3)	7315(2)	8761(3)	39.5(7)

C(28)	3390(3)	7239(2)	9126(3)	37.8(7)
C(29)	4058(3)	6411.8(18)	9079(2)	29.9(6)
C(30)	5573(3)	4758.3(17)	9118(2)	27.2(5)
C(31)	5800(3)	4677(2)	10200(2)	35.3(6)
C(32)	7116(4)	4737(2)	10730(3)	43.4(8)
C(33)	8212(4)	4859(2)	10193(3)	45.3(8)
C(34)	7995(3)	4934(2)	9129(3)	44.3(8)
C(35)	6678(3)	4885(2)	8589(2)	36.9(7)
C(36)	3375(3)	3928.6(19)	9007.9(19)	28.5(5)
C(37)	2478(4)	4035(2)	9735(2)	43.4(7)
C(38)	1935(5)	3285(3)	10180(3)	57.4(10)
C(39)	2246(5)	2423(3)	9910(3)	56.4(10)
C(40)	3125(4)	2295(2)	9180(3)	47.2(8)
C(41)	3701(3)	3036(2)	8747(2)	36.0(7)
Cl(1)	2794(2)	2292.0(13)	6110.8(17)	48.9(5)
Cl(2)	5444(3)	1487(3)	6567(2)	132.3(18)
C(42)	4031(10)	1647(9)	5658(7)	85(3)
Cl(1A)	3101(11)	2002(11)	6097(9)	149(4)
C(42A)	4670(20)	1780(20)	5782(18)	98(5)
Cl(2A)	5884(5)	2374(4)	6629(4)	92(2)

Table S15. Anisotropic Displacement Parameters (Å^2×10^3) for 32b. The Anisotropic displacement factor exponent takes the form: -2π^2[h^{2}a*^{2}U$_{11}$+2hka*b*U$_{12}$+…].

Atom	U_{11}	U_{22}	U_{33}	U_{23}	U_{13}	U_{12}
C(1)	125(5)	45(2)	57(3)	6.7(18)	12(3)	11(2)
N(1)	24.9(11)	37.7(12)	21.3(10)	-3.0(9)	3.1(8)	-2.9(10)

O(1)	26.4(10)	79.8(17)	31.1(10)	-4.7(11)	3.7(8)	-20.2(11)
S(1)	27.3(3)	45.7(4)	20.5(3)	-3.7(3)	0.8(2)	-13.0(3)
C(2)	74(3)	45.0(19)	28.5(16)	-1.8(14)	0.5(16)	5.4(17)
N(2)	33.1(12)	22.1(11)	17.9(10)	-2.4(8)	0.6(9)	1.3(9)
O(2)	49.0(13)	44.6(12)	28.3(10)	-7.2(9)	3.1(9)	-16.3(10)
S(2)	22.8(3)	25.9(3)	21.2(3)	0.7(2)	-0.3(2)	-2.0(2)
C(3)	69(3)	58(2)	33.8(17)	-0.4(16)	-2.8(16)	26.6(19)
O(3)	27.3(10)	46.0(12)	35.3(11)	2.1(9)	1.9(8)	-12.4(8)
C(4)	38.8(17)	66(2)	26.7(15)	-0.8(14)	0.7(12)	13.3(15)
C(5)	33.3(15)	44.4(16)	19.4(13)	-2.9(11)	-5.0(11)	-3.2(12)
C(6)	38.3(15)	40.8(17)	33.1(15)	-1.1(12)	8.1(12)	-5.9(12)
C(7)	59(2)	46.1(19)	36.6(18)	-0.2(14)	8.0(15)	-8.1(16)
C(8)	24.0(12)	29.2(13)	17.0(11)	4.0(9)	2.7(9)	-1.2(10)
C(9)	18.8(12)	47.3(17)	20.1(12)	-3.7(11)	0.9(10)	-0.7(11)
C(10)	28.5(14)	66(2)	26.8(14)	-2.0(14)	6.0(11)	-10.1(14)
C(11)	29.5(16)	93(3)	38.2(17)	-5.1(18)	10.3(13)	-10.8(18)
C(12)	24.4(15)	99(3)	48(2)	-17(2)	3.1(14)	6.1(18)
C(13)	38.2(19)	68(2)	48(2)	-21.3(18)	-10.4(15)	17.2(17)
C(14)	27.8(14)	47.5(18)	35.8(16)	-10.5(13)	-10.1(12)	5.8(12)
C(15)	51(2)	38.8(18)	56(2)	2.1(15)	-11.5(16)	0.6(15)
C(16)	21.6(12)	29.7(12)	19.1(11)	0.3(10)	3.5(9)	-1.5(10)
C(17)	27.3(13)	30.9(14)	26.5(13)	0.8(10)	5.9(10)	1.5(10)
C(18)	24.4(14)	46.5(18)	48.2(18)	-3.8(14)	7.0(12)	4.0(12)
C(19)	33.9(17)	61(2)	62(2)	-6.0(18)	18.5(15)	6.4(15)
C(20)	49(2)	60(2)	51(2)	-14.5(17)	23.0(17)	10.2(17)
C(21)	46.7(18)	47.2(18)	33.2(16)	-10.4(14)	10.3(14)	0.1(14)
C(22)	27.3(13)	35.2(15)	24.1(12)	1.5(11)	7.4(10)	-1.2(10)

C(23)	31.6(14)	22.8(12)	18.4(12)	-2.1(10)	0.7(10)	-0.5(11)
C(24)	29.9(13)	23.5(13)	19.1(11)	0.2(9)	2.9(10)	0.4(10)
C(25)	30.2(14)	34.0(14)	30.4(14)	-8.2(11)	2.2(11)	-1.0(11)
C(26)	33.1(16)	42.8(17)	35.3(15)	-5.6(13)	-1.0(12)	10.4(13)
C(27)	45.3(18)	33.2(15)	38.7(16)	-2.1(12)	0.8(13)	14.1(13)
C(28)	44.2(17)	23.1(14)	44.7(17)	-2.5(12)	0.7(14)	-0.5(12)
C(29)	33.4(15)	23.8(13)	31.5(14)	0.2(11)	0.4(11)	-0.9(11)
C(30)	35.3(14)	17.8(11)	26.0(13)	-2.7(10)	-4.8(10)	3.8(10)
C(31)	47.9(17)	26.9(13)	28.1(14)	-3.1(11)	-6.2(12)	-0.5(12)
C(32)	61(2)	27.4(14)	34.6(16)	-2.3(12)	-20.2(15)	3.3(14)
C(33)	42.7(18)	34.5(17)	51.5(19)	-11.0(14)	-19.9(15)	10.1(13)
C(34)	30.4(15)	49.8(19)	49.8(19)	-11.2(15)	-5.3(14)	8.9(13)
C(35)	33.0(15)	42.3(17)	33.2(15)	-6.3(12)	-4.2(12)	8.6(12)
C(36)	39.0(14)	25.4(13)	19.2(11)	0.0(10)	-2.4(10)	-4.1(11)
C(37)	66(2)	36.9(16)	30.2(14)	-5.5(13)	17.4(14)	-11.6(15)
C(38)	83(3)	53(2)	40.0(19)	0.7(16)	23.9(19)	-22.2(19)
C(39)	84(3)	43(2)	39.9(18)	13.9(15)	0.9(18)	-25.2(18)
C(40)	67(2)	25.9(15)	43.2(18)	3.3(13)	-12.6(17)	-6.5(14)
C(41)	47.6(18)	28.3(15)	29.7(14)	1.4(11)	-3.4(13)	1.0(12)
Cl(1)	49.7(9)	53.3(9)	45.0(8)	-1.1(6)	10.5(6)	6.0(6)
Cl(2)	95.2(19)	193(4)	99.0(18)	-44(2)	-23.6(14)	77(2)
C(42)	69(5)	132(7)	53(4)	-25(4)	1(4)	68(6)
Cl(1A)	117(6)	198(9)	127(6)	-51(6)	-7(5)	-4(6)
C(42A)	94(10)	131(9)	68(8)	-13(8)	13(9)	58(10)
Cl(2A)	73(3)	125(5)	81(3)	3(3)	23(2)	36(3)

Table S16. Bond Lengths for 32b.

Atom	Atom	Length/Å	Atom	Atom	Length/Å
C(1)	C(2)	1.499(6)	C(18)	C(19)	1.387(5)
N(1)	S(1)	1.684(2)	C(19)	C(20)	1.377(6)
N(1)	C(8)	1.420(3)	C(20)	C(21)	1.386(5)
N(1)	C(22)	1.429(3)	C(21)	C(22)	1.392(4)
O(1)	S(1)	1.423(2)	C(23)	C(24)	1.536(3)
S(1)	O(2)	1.425(2)	C(23)	C(30)	1.548(4)
S(1)	C(5)	1.756(3)	C(23)	C(36)	1.536(4)
C(2)	C(3)	1.389(6)	C(24)	C(25)	1.398(4)
C(2)	C(7)	1.388(5)	C(24)	C(29)	1.387(4)
N(2)	S(2)	1.664(2)	C(25)	C(26)	1.377(4)
N(2)	C(23)	1.498(3)	C(26)	C(27)	1.390(5)
S(2)	O(3)	1.483(2)	C(27)	C(28)	1.374(5)
S(2)	C(16)	1.783(3)	C(28)	C(29)	1.388(4)
C(3)	C(4)	1.379(6)	C(30)	C(31)	1.400(4)
C(4)	C(5)	1.386(5)	C(30)	C(35)	1.381(4)
C(5)	C(6)	1.389(4)	C(31)	C(32)	1.395(4)
C(6)	C(7)	1.381(5)	C(32)	C(33)	1.379(6)
C(8)	C(9)	1.478(4)	C(33)	C(34)	1.375(5)
C(8)	C(16)	1.355(4)	C(34)	C(35)	1.399(4)
C(9)	C(10)	1.415(4)	C(36)	C(37)	1.390(4)
C(9)	C(14)	1.387(4)	C(36)	C(41)	1.402(4)
C(10)	C(11)	1.383(5)	C(37)	C(38)	1.386(5)
C(11)	C(12)	1.358(7)	C(38)	C(39)	1.360(6)
C(12)	C(13)	1.366(7)	C(39)	C(40)	1.384(6)
C(13)	C(14)	1.419(5)	C(40)	C(41)	1.382(5)

C(14)	C(15)	1.479(5)	Cl(1)	C(42)	1.715(7)
C(16)	C(17)	1.441(4)	Cl(2)	C(42)	1.726(9)
C(17)	C(18)	1.400(4)	Cl(1A)	C(42A)	1.694(18)
C(17)	C(22)	1.397(4)	C(42A)	Cl(2A)	1.76(2)

Table S17. Bond Angles for 32b.

Atom	Atom	Atom	Angle/°	Atom	Atom	Atom	Angle/°
C(8)	N(1)	S(1)	122.82(19)	C(22)	C(17)	C(18)	120.1(3)
C(8)	N(1)	C(22)	107.6(2)	C(19)	C(18)	C(17)	117.7(3)
C(22)	N(1)	S(1)	122.53(18)	C(20)	C(19)	C(18)	121.5(3)
N(1)	S(1)	C(5)	103.72(12)	C(19)	C(20)	C(21)	121.9(3)
O(1)	S(1)	N(1)	107.38(12)	C(20)	C(21)	C(22)	117.0(3)
O(1)	S(1)	O(2)	119.94(14)	C(17)	C(22)	N(1)	107.6(2)
O(1)	S(1)	C(5)	109.12(16)	C(21)	C(22)	N(1)	130.5(3)
O(2)	S(1)	N(1)	105.49(13)	C(21)	C(22)	C(17)	121.8(3)
O(2)	S(1)	C(5)	109.90(13)	N(2)	C(23)	C(24)	106.7(2)
C(3)	C(2)	C(1)	121.0(4)	N(2)	C(23)	C(30)	112.5(2)
C(7)	C(2)	C(1)	120.6(4)	N(2)	C(23)	C(36)	106.5(2)
C(7)	C(2)	C(3)	118.3(3)	C(24)	C(23)	C(30)	111.2(2)
C(23)	N(2)	S(2)	122.09(17)	C(24)	C(23)	C(36)	112.5(2)
N(2)	S(2)	C(16)	95.24(11)	C(36)	C(23)	C(30)	107.4(2)
O(3)	S(2)	N(2)	113.04(12)	C(25)	C(24)	C(23)	119.8(2)
O(3)	S(2)	C(16)	104.17(12)	C(29)	C(24)	C(23)	121.9(2)
C(4)	C(3)	C(2)	121.8(3)	C(29)	C(24)	C(25)	118.1(2)
C(3)	C(4)	C(5)	118.5(3)	C(26)	C(25)	C(24)	120.8(3)
C(4)	C(5)	S(1)	119.7(3)	C(25)	C(26)	C(27)	120.5(3)

C(4)	C(5)	C(6)	121.3(3)	C(28)	C(27)	C(26)	119.1(3)
C(6)	C(5)	S(1)	119.0(2)	C(27)	C(28)	C(29)	120.6(3)
C(7)	C(6)	C(5)	118.8(3)	C(24)	C(29)	C(28)	120.8(3)
C(6)	C(7)	C(2)	121.3(3)	C(31)	C(30)	C(23)	119.1(3)
N(1)	C(8)	C(9)	123.9(2)	C(35)	C(30)	C(23)	122.3(2)
C(16)	C(8)	N(1)	108.3(2)	C(35)	C(30)	C(31)	118.6(3)
C(16)	C(8)	C(9)	127.7(2)	C(32)	C(31)	C(30)	120.3(3)
C(10)	C(9)	C(8)	117.9(3)	C(33)	C(32)	C(31)	120.5(3)
C(14)	C(9)	C(8)	121.4(3)	C(34)	C(33)	C(32)	119.3(3)
C(14)	C(9)	C(10)	120.6(3)	C(33)	C(34)	C(35)	120.7(3)
C(11)	C(10)	C(9)	120.2(4)	C(30)	C(35)	C(34)	120.5(3)
C(12)	C(11)	C(10)	119.3(4)	C(37)	C(36)	C(23)	123.0(3)
C(11)	C(12)	C(13)	121.6(3)	C(37)	C(36)	C(41)	117.3(3)
C(12)	C(13)	C(14)	121.4(4)	C(41)	C(36)	C(23)	119.5(3)
C(9)	C(14)	C(13)	116.9(3)	C(38)	C(37)	C(36)	120.9(3)
C(9)	C(14)	C(15)	123.9(3)	C(39)	C(38)	C(37)	121.1(4)
C(13)	C(14)	C(15)	119.2(3)	C(38)	C(39)	C(40)	119.2(3)
C(8)	C(16)	S(2)	122.6(2)	C(41)	C(40)	C(39)	120.3(3)
C(8)	C(16)	C(17)	109.4(2)	C(40)	C(41)	C(36)	121.0(3)
C(17)	C(16)	S(2)	128.0(2)	Cl(1)	C(42)	Cl(2)	113.1(5)
C(18)	C(17)	C(16)	132.9(3)	Cl(1A)	C(42A)	Cl(2A)	109.0(12)
C(22)	C(17)	C(16)	107.0(2)				

Table S18. Torsion Angles for 32b.

A	B	C	D	Angle/°	A	B	C	D	Angle/°
C(1)	C(2)	C(3)	C(4)	-176.2(4)	C(14)	C(9)	C(10)	C(11)	0.9(4)

C(1)	C(2)	C(7)	C(6)	176.2(4)	C(16)	C(8)	C(9)	C(10)	-110.5(3)
N(1)	S(1)	C(5)	C(4)	-106.7(2)	C(16)	C(8)	C(9)	C(14)	67.6(4)
N(1)	S(1)	C(5)	C(6)	71.3(2)	C(16)	C(17)	C(18)	C(19)	179.9(3)
N(1)	C(8)	C(9)	C(10)	65.2(3)	C(16)	C(17)	C(22)	N(1)	-1.1(3)
N(1)	C(8)	C(9)	C(14)	-116.8(3)	C(16)	C(17)	C(22)	C(21)	-178.0(3)
N(1)	C(8)	C(16)	S(2)	-178.59(18)	C(17)	C(18)	C(19)	C(20)	-1.1(6)
N(1)	C(8)	C(16)	C(17)	2.8(3)	C(18)	C(17)	C(22)	N(1)	179.0(3)
O(1)	S(1)	C(5)	C(4)	7.5(3)	C(18)	C(17)	C(22)	C(21)	2.1(4)
O(1)	S(1)	C(5)	C(6)	-174.4(2)	C(18)	C(19)	C(20)	C(21)	0.7(7)
S(1)	N(1)	C(8)	C(9)	28.9(3)	C(19)	C(20)	C(21)	C(22)	1.0(6)
S(1)	N(1)	C(8)	C(16)	-154.71(19)	C(20)	C(21)	C(22)	N(1)	-178.6(3)
S(1)	N(1)	C(22)	C(17)	154.1(2)	C(20)	C(21)	C(22)	C(17)	-2.4(5)
S(1)	N(1)	C(22)	C(21)	-29.3(4)	C(22)	N(1)	S(1)	O(1)	163.1(2)
S(1)	C(5)	C(6)	C(7)	-176.7(3)	C(22)	N(1)	S(1)	O(2)	34.1(3)
C(2)	C(3)	C(4)	C(5)	-0.7(5)	C(22)	N(1)	S(1)	C(5)	-81.4(2)
N(2)	S(2)	C(16)	C(8)	91.9(2)	C(22)	N(1)	C(8)	C(9)	-179.9(2)
N(2)	S(2)	C(16)	C(17)	-89.8(3)	C(22)	N(1)	C(8)	C(16)	-3.5(3)
N(2)	C(23)	C(24)	C(25)	63.1(3)	C(22)	C(17)	C(18)	C(19)	-0.3(5)
N(2)	C(23)	C(24)	C(29)	-113.0(3)	C(23)	N(2)	S(2)	O(3)	87.8(2)
N(2)	C(23)	C(30)	C(31)	-163.5(2)	C(23)	N(2)	S(2)	C(16)	-164.4(2)
N(2)	C(23)	C(30)	C(35)	18.4(3)	C(23)	C(24)	C(25)	C(26)	-176.8(3)
N(2)	C(23)	C(36)	C(37)	-137.2(3)	C(23)	C(24)	C(29)	C(28)	176.7(3)
N(2)	C(23)	C(36)	C(41)	47.9(3)	C(23)	C(30)	C(31)	C(32)	-177.2(3)
O(2)	S(1)	C(5)	C(4)	140.9(2)	C(23)	C(30)	C(35)	C(34)	177.8(3)
O(2)	S(1)	C(5)	C(6)	-41.0(3)	C(23)	C(36)	C(37)	C(38)	-174.9(3)
S(2)	N(2)	C(23)	C(24)	29.7(3)	C(23)	C(36)	C(41)	C(40)	176.6(3)
S(2)	N(2)	C(23)	C(30)	-92.5(2)	C(24)	C(23)	C(30)	C(31)	76.8(3)

S(2)	N(2)	C(23)	C(36)	150.11(19)	C(24)	C(23)	C(30)	C(35)	-101.2(3)
S(2)	C(16)	C(17)	C(18)	0.3(5)	C(24)	C(23)	C(36)	C(37)	-20.7(4)
S(2)	C(16)	C(17)	C(22)	-179.6(2)	C(24)	C(23)	C(36)	C(41)	164.5(2)
C(3)	C(2)	C(7)	C(6)	-2.6(5)	C(24)	C(25)	C(26)	C(27)	0.8(5)
C(3)	C(4)	C(5)	S(1)	176.7(3)	C(25)	C(24)	C(29)	C(28)	0.4(4)
C(3)	C(4)	C(5)	C(6)	-1.3(5)	C(25)	C(26)	C(27)	C(28)	-1.0(5)
O(3)	S(2)	C(16)	C(8)	-152.7(2)	C(26)	C(27)	C(28)	C(29)	0.9(5)
O(3)	S(2)	C(16)	C(17)	25.6(3)	C(27)	C(28)	C(29)	C(24)	-0.6(5)
C(4)	C(5)	C(6)	C(7)	1.3(4)	C(29)	C(24)	C(25)	C(26)	-0.5(4)
C(5)	C(6)	C(7)	C(2)	0.7(5)	C(30)	C(23)	C(24)	C(25)	-173.8(2)
C(7)	C(2)	C(3)	C(4)	2.6(5)	C(30)	C(23)	C(24)	C(29)	10.0(4)
C(8)	N(1)	S(1)	O(1)	-49.9(3)	C(30)	C(23)	C(36)	C(37)	102.0(3)
C(8)	N(1)	S(1)	O(2)	-178.9(2)	C(30)	C(23)	C(36)	C(41)	-72.8(3)
C(8)	N(1)	S(1)	C(5)	65.6(2)	C(30)	C(31)	C(32)	C(33)	-1.2(5)
C(8)	N(1)	C(22)	C(17)	2.8(3)	C(31)	C(30)	C(35)	C(34)	-0.2(4)
C(8)	N(1)	C(22)	C(21)	179.4(3)	C(31)	C(32)	C(33)	C(34)	0.8(5)
C(8)	C(9)	C(10)	C(11)	178.9(3)	C(32)	C(33)	C(34)	C(35)	-0.1(5)
C(8)	C(9)	C(14)	C(13)	-179.7(2)	C(33)	C(34)	C(35)	C(30)	-0.2(5)
C(8)	C(9)	C(14)	C(15)	-0.9(5)	C(35)	C(30)	C(31)	C(32)	0.9(4)
C(8)	C(16)	C(17)	C(18)	178.8(3)	C(36)	C(23)	C(24)	C(25)	-53.3(3)
C(8)	C(16)	C(17)	C(22)	-1.1(3)	C(36)	C(23)	C(24)	C(29)	130.5(3)
C(9)	C(8)	C(16)	S(2)	-2.4(4)	C(36)	C(23)	C(30)	C(31)	-46.7(3)
C(9)	C(8)	C(16)	C(17)	179.0(2)	C(36)	C(23)	C(30)	C(35)	135.3(3)
C(9)	C(10)	C(11)	C(12)	0.8(5)	C(36)	C(37)	C(38)	C(39)	-0.9(6)
C(10)	C(9)	C(14)	C(13)	-1.7(4)	C(37)	C(36)	C(41)	C(40)	1.5(4)
C(10)	C(9)	C(14)	C(15)	177.1(3)	C(37)	C(38)	C(39)	C(40)	0.2(7)
C(10)	C(11)	C(12)	C(13)	-1.6(5)	C(38)	C(39)	C(40)	C(41)	1.4(6)

C(11)	C(12)	C(13)	C(14)	0.8(5)	C(39)	C(40)	C(41)	C(36)	-2.3(5)
C(12)	C(13)	C(14)	C(9)	0.9(5)	C(41)	C(36)	C(37)	C(38)	0.1(5)
C(12)	C(13)	C(14)	C(15)	-178.0(3)					

Table S19. Hydrogen Atom Coordinates (Å×10^4^) and Isotropic Displacement Parameters (Å^2^×10^3^) for 32b

Atom	*x*	*y*	*z*	U(eq)
H(1A)	2083.4	7939.53	1740.26	114
H(1B)	468.15	8071.64	1545.54	114
H(1C)	1244.13	7651.3	651.34	114
H(2)	4015.78	3991.29	7193.44	30
H(3)	-651.82	7016.59	2484.52	65
H(4)	-868.46	5588.55	3224.85	53
H(6)	2443.99	4724.08	1964.25	44
H(7)	2633.47	6161.1	1235.28	56
H(10)	287.98	3695.28	5991.08	48
H(11)	-1595.85	4221.12	6722.05	64
H(12)	-2124.9	5749.24	6644.05	69
H(13)	-779.17	6781.91	5930.93	63
H(15A)	2172.4	6788.03	5629.03	75
H(15B)	906.4	7083.41	4802.03	75
H(15C)	1921.22	6303.12	4516.96	75
H(18)	6288.06	4421.24	5345.64	47
H(19)	7087.51	3521.45	4065.9	61
H(20)	5604.79	2772.14	2835.29	63
H(21)	3243.14	2899.56	2799.49	50

H(25)	1489.72	5212	8020.08	38
H(26)	379.75	6593.33	8112.33	45
H(27)	1577.88	7883.97	8785.78	47
H(28)	3877.6	7756.35	9412.3	45
H(29)	5001.31	6371.58	9327.52	36
H(31)	5053.57	4581.62	10576.34	42
H(32)	7258.28	4692.76	11465.69	52
H(33)	9108.19	4892.18	10555.25	54
H(34)	8746.33	5019.22	8756.33	53
H(35)	6544.04	4939.27	7854.48	44
H(37)	2234.41	4630.87	9929.39	52
H(38)	1334.68	3374.71	10683.22	69
H(39)	1865.18	1913.53	10218.69	68
H(40)	3333.42	1695.62	8974.65	57
H(41)	4327.83	2938.86	8265.22	43
H(42A)	4313.64	1948.29	5039.25	103
H(42B)	3644.1	1044.79	5437.27	103
H(42C)	4720.24	1964	5056.78	117
H(42D)	4852.97	1113.41	5839.86	117

Table S20. Atomic Occupancy for 32b.

**Atom**	***Occupancy***	**Atom**	***Occupancy***	**Atom**	***Occupancy***
Cl(1)	0.669(6)	Cl(2)	0.669(6)	C(42)	0.669(6)
H(42A)	0.669(6)	H(42B)	0.669(6)	Cl(1A)	0.331(6)
C(42A)	0.331(6)	H(42C)	0.331(6)	H(42D)	0.331(6)
Cl(2A)	0.331(6)				

6.3 Crystallographic Date of 55b

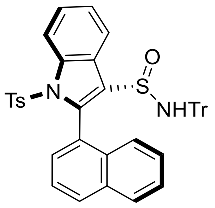

≡

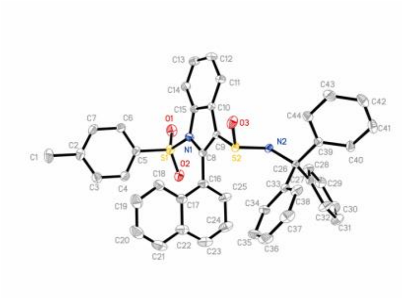

55b

CCDC 2436917

Table S21. Crystal data and structure refinement for 55b.

Identification code	**55b**
Empirical formula	$C_{45}H_{36}Cl_2N_2O_3S_2$
Formula weight	787.78
Temperature/K	193.00
Crystal system	orthorhombic
Space group	$P2_12_12_1$
a/Å	8.9414(2)
b/Å	15.1873(4)
c/Å	28.7842(7)
α/°	90
β/°	90
γ/°	90
Volume/Å^3	3908.77(17)
Z	4
ρ_{calc}g/cm^3	1.339
μ/mm^{-1}	2.839
F(000)	1640.0
Crystal size/mm^3	0.18 × 0.15 × 0.11
Radiation	CuKα (λ = 1.54178)

2Θ range for data collection/°	6.14 to 136.67
Index ranges	-10 ≤ h ≤ 10, -18 ≤ k ≤ 18, -34 ≤ l ≤ 34
Reflections collected	84232
Independent reflections	7163 [R_{int} = 0.0761, R_{sigma} = 0.0366]
Data/restraints/parameters	7163/0/489
Goodness-of-fit on F^2	1.031
Final R indexes [I>=2σ (I)]	R_1 = 0.0405, wR_2 = 0.1090
Final R indexes [all data]	R_1 = 0.0422, wR_2 = 0.1104
Largest diff. peak/hole / e Å^{-3}	0.48/-0.41
Flack parameter	0.099(18)

Table S22. Fractional Atomic Coordinates (×10^4) and Equivalent Isotropic Displacement Parameters (Å^2×10^3) for 55b. U_{eq} is defined as 1/3 of the trace of the orthogonalised U_{IJ} tensor.

**Atom**	***x***	***y***	***z***	**U(eq)**
C(1)	-542(6)	3609(3)	6597.8(16)	54.6(11)
N(1)	5261(3)	4745.3(18)	5228.0(9)	29.5(6)
O(1)	6164(3)	5076(2)	6018.8(9)	49.5(7)
S(1)	4909.4(10)	5266.7(6)	5732.7(3)	33.7(2)
C(2)	811(5)	4019(3)	6374.1(12)	40.3(9)
N(2)	5930(3)	4916.8(17)	3674.7(9)	24.0(6)
O(2)	4546(3)	6151.8(16)	5614.6(9)	44.1(7)
S(2)	4482.0(8)	4378.2(5)	3896.9(3)	26.21(18)
C(3)	724(5)	4828(3)	6156.1(14)	45.5(9)
O(3)	4447(3)	3437.5(16)	3754.9(9)	40.9(6)
C(4)	1965(5)	5210(3)	5953.6(14)	41.2(9)
C(5)	3308(4)	4769(2)	5968.9(11)	31.5(7)
C(6)	3432(5)	3955(2)	6184.5(13)	40.9(9)

C(7)	2173(5)	3590(3)	6383.9(14)	45.8(10)
C(8)	4653(4)	4966(2)	4790.3(11)	27.1(7)
C(9)	5147(3)	4367(2)	4478.0(10)	24.0(6)
C(10)	6080(4)	3731(2)	4705.9(11)	26.0(7)
C(11)	6878(4)	2990(2)	4553.9(13)	36.4(8)
C(12)	7678(5)	2516(3)	4871.7(15)	47.5(10)
C(13)	7719(5)	2761(3)	5342.3(16)	48.9(10)
C(14)	6947(4)	3481(3)	5500.8(13)	40.8(9)
C(15)	6134(4)	3969(2)	5179.3(11)	28.5(7)
C(16)	3701(4)	5746(2)	4693.5(12)	34.8(8)
C(17)	2128(4)	5664(3)	4723.4(13)	37.6(8)
C(18)	1421(4)	4882(3)	4838.7(14)	42.5(9)
C(19)	-121(5)	4831(4)	4867.3(17)	59.1(12)
C(20)	-973(5)	5587(4)	4793.4(19)	68.8(15)
C(21)	-321(6)	6356(4)	4678.1(19)	64.3(14)
C(22)	1230(5)	6436(3)	4636.2(14)	47.6(10)
C(23)	1958(6)	7203(3)	4499.1(18)	60.6(13)
C(24)	3481(6)	7272(3)	4452.6(18)	59.7(13)
C(25)	4382(5)	6525(2)	4550.8(14)	44.9(9)
C(26)	5895(3)	5327(2)	3197.8(10)	24.1(6)
C(27)	6623(4)	6245(2)	3236.9(11)	26.8(7)
C(28)	7774(4)	6416(2)	3547.3(14)	36.1(8)
C(29)	8414(5)	7240(3)	3572.3(15)	44.6(9)
C(30)	7945(5)	7914(3)	3287.3(16)	48.2(10)
C(31)	6826(6)	7753(3)	2971.1(16)	52.0(11)
C(32)	6161(5)	6927(2)	2950.0(14)	43.5(9)
C(33)	4255(4)	5442(2)	3049.1(11)	27.8(7)

C(34)	3325(4)	5966(3)	3324.4(14)	39.2(9)
C(35)	1868(5)	6133(3)	3190.9(18)	50.5(11)
C(36)	1325(5)	5803(3)	2776.0(19)	57.5(13)
C(37)	2226(5)	5296(3)	2501.8(16)	50.2(11)
C(38)	3694(4)	5107(2)	2636.9(13)	35.9(8)
C(39)	6741(4)	4737(2)	2855.2(11)	27.6(7)
C(40)	7547(4)	5095(3)	2487.5(12)	34.5(8)
C(41)	8218(5)	4560(3)	2156.0(13)	45.6(10)
C(42)	8105(5)	3663(3)	2185.6(14)	48.3(10)
C(43)	7307(5)	3287(3)	2549.0(15)	46.1(10)
C(44)	6634(4)	3819(2)	2881.2(13)	35.9(8)
Cl(1)	-97(3)	3712.4(16)	3779.6(6)	112.8(7)
Cl(2)	816.5(18)	2436.0(12)	3090.3(6)	86.5(5)
C(45)	844(7)	3542(4)	3251(2)	78.6(18)

Table S23. Anisotropic Displacement Parameters (Å^2×10^3) for 55b. The Anisotropic displacement factor exponent takes the form: -2π^2[h^{2}a*^{2}U$_{11}$+2hka*b*U$_{12}$+…].

**Atom**	**U$_{11}$**	**U$_{22}$**	**U$_{33}$**	**U$_{23}$**	**U$_{13}$**	**U$_{12}$**
C(1)	63(3)	55(3)	46(2)	-12.1(19)	20(2)	-18(2)
N(1)	32.6(14)	35.0(15)	20.9(12)	-1.0(11)	0.5(11)	7.3(12)
O(1)	47.5(15)	71(2)	29.7(13)	-6.1(13)	-8.4(11)	-7.5(14)
S(1)	41.4(5)	36.0(4)	23.6(4)	-5.2(3)	1.3(3)	-6.9(4)
C(2)	57(2)	38.2(19)	26.1(17)	-9.9(14)	9.2(16)	-11.6(18)
N(2)	24.9(13)	26.8(13)	20.1(12)	5.4(10)	-3.5(10)	-1.1(11)
O(2)	64.1(18)	30.9(12)	37.3(13)	-8.0(10)	10.8(13)	-11.6(13)
S(2)	27.7(4)	28.8(4)	22.1(3)	1.5(3)	-0.8(3)	-3.8(3)

C(3)	45(2)	48(2)	44(2)	-0.9(18)	6.1(18)	0.6(19)
O(3)	59.9(16)	29.4(12)	33.3(13)	-3.0(10)	4.0(12)	-15.7(12)
C(4)	48(2)	36.3(19)	39.1(19)	4.6(16)	4.7(16)	-0.5(18)
C(5)	43.3(19)	29.7(16)	21.5(15)	-4.3(13)	4.4(13)	-3.6(16)
C(6)	54(2)	35.5(19)	33.7(19)	3.3(15)	11.0(17)	8.1(18)
C(7)	71(3)	30.3(18)	36(2)	3.2(16)	16(2)	-1.3(19)
C(8)	26.6(16)	28.9(16)	25.7(15)	3.0(12)	-0.6(12)	2.9(13)
C(9)	26.7(15)	22.4(14)	22.8(14)	4.2(11)	2.8(12)	0.2(13)
C(10)	26.5(15)	25.1(16)	26.6(16)	4.3(12)	2.7(13)	0.7(13)
C(11)	40.2(19)	30.7(18)	38.4(19)	2.1(15)	4.7(16)	9.1(16)
C(12)	49(2)	36(2)	57(3)	7(2)	5(2)	16.0(18)
C(13)	46(2)	50(2)	51(2)	20(2)	-3.0(19)	15.3(19)
C(14)	40(2)	51(2)	31.5(19)	13.2(17)	-1.3(16)	10.5(18)
C(15)	25.6(15)	32.8(17)	27.0(16)	7.2(13)	-0.2(13)	3.9(13)
C(16)	40.9(19)	33.3(19)	30.2(17)	-3.4(14)	-4.5(15)	7.6(16)
C(17)	42(2)	39.0(19)	32.1(18)	-5.2(16)	-3.0(15)	8.0(17)
C(18)	38(2)	52(2)	37.1(19)	0.6(17)	0.3(16)	6.1(18)
C(19)	40(2)	77(3)	61(3)	-1(2)	0(2)	-8(2)
C(20)	38(2)	101(4)	68(3)	1(3)	-4(2)	14(3)
C(21)	55(3)	76(4)	62(3)	-7(3)	-10(2)	31(3)
C(22)	58(3)	46(2)	38(2)	-8.4(18)	-9.6(19)	15(2)
C(23)	69(3)	52(3)	61(3)	-4(2)	-19(2)	21(2)
C(24)	83(4)	33(2)	63(3)	4.3(19)	-12(3)	4(2)
C(25)	60(2)	27.6(18)	47(2)	1.6(16)	-12(2)	2.4(18)
C(26)	27.7(16)	24.7(15)	19.9(14)	3.6(12)	0.1(12)	2.5(13)
C(27)	29.5(16)	27.9(16)	23.0(15)	0.5(12)	2.6(13)	-0.2(13)
C(28)	35.7(19)	31.1(18)	42(2)	0.0(15)	-4.1(16)	-4.5(15)

C(29)	40(2)	43(2)	51(2)	-5.2(18)	-6.2(18)	-10.4(18)
C(30)	54(2)	27.9(18)	63(3)	-5.1(18)	11(2)	-8.8(18)
C(31)	67(3)	30(2)	59(3)	8.3(18)	-5(2)	-6(2)
C(32)	53(2)	31.0(19)	47(2)	8.8(16)	-13.0(19)	-4.9(17)
C(33)	27.1(16)	28.0(16)	28.4(15)	8.9(13)	-3.1(13)	-1.4(13)
C(34)	34.9(19)	44(2)	38.6(19)	6.3(16)	-1.8(16)	10.8(17)
C(35)	31(2)	49(2)	71(3)	14(2)	-2(2)	11.1(18)
C(36)	27(2)	58(3)	87(3)	28(3)	-17(2)	-1.5(19)
C(37)	40(2)	53(2)	57(3)	13(2)	-24(2)	-12(2)
C(38)	36.0(19)	31.6(18)	40.2(19)	5.4(15)	-10.0(16)	-7.5(15)
C(39)	26.6(16)	32.5(16)	23.6(15)	-3.1(13)	-2.0(12)	1.9(14)
C(40)	34.0(18)	44(2)	25.2(16)	2.8(15)	1.8(14)	-0.6(16)
C(41)	42(2)	67(3)	27.9(18)	-5.7(18)	8.0(16)	-4(2)
C(42)	46(2)	62(3)	37(2)	-18.8(19)	4.8(18)	9(2)
C(43)	52(2)	38(2)	48(2)	-11.8(18)	4.4(19)	6.4(18)
C(44)	41(2)	33.9(18)	32.9(18)	-4.4(14)	6.2(16)	1.8(16)
Cl(1)	118.6(15)	161.1(19)	58.8(8)	-10.3(10)	-10.7(9)	32.5(14)
Cl(2)	77.9(10)	91.5(10)	90.1(10)	10.5(8)	7.4(8)	7.6(8)
C(45)	73(4)	97(4)	65(3)	20(3)	-7(3)	-47(3)

Table S24. Bond Lengths for 55b.

**Atom**	**Atom**	**Length/Å**	**Atom**	**Atom**	**Length/Å**
C(1)	C(2)	1.506(6)	C(18)	C(19)	1.383(6)
N(1)	S(1)	1.684(3)	C(19)	C(20)	1.394(8)
N(1)	C(8)	1.413(4)	C(20)	C(21)	1.346(8)
N(1)	C(15)	1.420(4)	C(21)	C(22)	1.397(7)

O(1)	S(1)	1.422(3)	C(22)	C(23)	1.392(7)
S(1)	O(2)	1.424(3)	C(23)	C(24)	1.372(8)
S(1)	C(5)	1.756(4)	C(24)	C(25)	1.420(6)
C(2)	C(3)	1.382(6)	C(26)	C(27)	1.542(4)
C(2)	C(7)	1.381(6)	C(26)	C(33)	1.538(4)
N(2)	S(2)	1.659(3)	C(26)	C(39)	1.532(4)
N(2)	C(26)	1.508(4)	C(27)	C(28)	1.388(5)
S(2)	O(3)	1.486(2)	C(27)	C(32)	1.388(5)
S(2)	C(9)	1.775(3)	C(28)	C(29)	1.378(5)
C(3)	C(4)	1.382(6)	C(29)	C(30)	1.377(6)
C(4)	C(5)	1.376(6)	C(30)	C(31)	1.375(7)
C(5)	C(6)	1.387(5)	C(31)	C(32)	1.390(6)
C(6)	C(7)	1.380(6)	C(33)	C(34)	1.398(5)
C(8)	C(9)	1.353(4)	C(33)	C(38)	1.384(5)
C(8)	C(16)	1.485(5)	C(34)	C(35)	1.382(5)
C(9)	C(10)	1.434(4)	C(35)	C(36)	1.383(7)
C(10)	C(11)	1.403(5)	C(36)	C(37)	1.366(7)
C(10)	C(15)	1.411(5)	C(37)	C(38)	1.398(5)
C(11)	C(12)	1.366(5)	C(39)	C(40)	1.391(5)
C(12)	C(13)	1.405(7)	C(39)	C(44)	1.401(5)
C(13)	C(14)	1.371(6)	C(40)	C(41)	1.390(5)
C(14)	C(15)	1.391(5)	C(41)	C(42)	1.368(7)
C(16)	C(17)	1.415(5)	C(42)	C(43)	1.389(6)
C(16)	C(25)	1.392(5)	C(43)	C(44)	1.389(5)
C(17)	C(18)	1.386(6)	Cl(1)	C(45)	1.759(6)
C(17)	C(22)	1.444(6)	Cl(2)	C(45)	1.742(7)

Table S25. Bond Angles for 55b.

Atom	Atom	Atom	Angle/°	Atom	Atom	Atom	Angle/°
C(8)	N(1)	S(1)	125.9(2)	C(18)	C(17)	C(16)	123.0(4)
C(8)	N(1)	C(15)	108.7(2)	C(18)	C(17)	C(22)	119.0(4)
C(15)	N(1)	S(1)	125.3(2)	C(19)	C(18)	C(17)	121.1(4)
N(1)	S(1)	C(5)	106.47(15)	C(18)	C(19)	C(20)	119.3(5)
O(1)	S(1)	N(1)	104.86(16)	C(21)	C(20)	C(19)	121.0(4)
O(1)	S(1)	O(2)	120.70(18)	C(20)	C(21)	C(22)	121.8(5)
O(1)	S(1)	C(5)	109.36(17)	C(21)	C(22)	C(17)	117.7(5)
O(2)	S(1)	N(1)	106.29(15)	C(23)	C(22)	C(17)	118.0(4)
O(2)	S(1)	C(5)	108.25(18)	C(23)	C(22)	C(21)	124.2(4)
C(3)	C(2)	C(1)	121.1(4)	C(24)	C(23)	C(22)	123.8(4)
C(7)	C(2)	C(1)	120.3(4)	C(23)	C(24)	C(25)	118.9(5)
C(7)	C(2)	C(3)	118.6(4)	C(16)	C(25)	C(24)	119.3(4)
C(26)	N(2)	S(2)	122.6(2)	N(2)	C(26)	C(27)	107.4(2)
N(2)	S(2)	C(9)	96.11(13)	N(2)	C(26)	C(33)	108.6(2)
O(3)	S(2)	N(2)	112.61(15)	N(2)	C(26)	C(39)	109.5(2)
O(3)	S(2)	C(9)	104.88(15)	C(33)	C(26)	C(27)	108.7(3)
C(4)	C(3)	C(2)	121.3(4)	C(39)	C(26)	C(27)	111.5(3)
C(5)	C(4)	C(3)	118.9(4)	C(39)	C(26)	C(33)	111.0(3)
C(4)	C(5)	S(1)	119.3(3)	C(28)	C(27)	C(26)	122.0(3)
C(4)	C(5)	C(6)	121.2(4)	C(28)	C(27)	C(32)	117.6(3)
C(6)	C(5)	S(1)	119.5(3)	C(32)	C(27)	C(26)	120.4(3)
C(7)	C(6)	C(5)	118.7(4)	C(29)	C(28)	C(27)	120.8(4)
C(6)	C(7)	C(2)	121.4(4)	C(30)	C(29)	C(28)	121.1(4)
N(1)	C(8)	C(16)	125.2(3)	C(31)	C(30)	C(29)	119.0(4)
C(9)	C(8)	N(1)	107.9(3)	C(30)	C(31)	C(32)	120.0(4)

C(9)	C(8)	C(16)	126.8(3)	C(27)	C(32)	C(31)	121.4(4)
C(8)	C(9)	S(2)	120.7(2)	C(34)	C(33)	C(26)	118.3(3)
C(8)	C(9)	C(10)	109.8(3)	C(38)	C(33)	C(26)	122.8(3)
C(10)	C(9)	S(2)	129.2(2)	C(38)	C(33)	C(34)	118.7(3)
C(11)	C(10)	C(9)	133.9(3)	C(35)	C(34)	C(33)	120.5(4)
C(11)	C(10)	C(15)	119.3(3)	C(34)	C(35)	C(36)	120.3(4)
C(15)	C(10)	C(9)	106.8(3)	C(37)	C(36)	C(35)	119.7(4)
C(12)	C(11)	C(10)	118.7(4)	C(36)	C(37)	C(38)	120.6(4)
C(11)	C(12)	C(13)	121.3(4)	C(33)	C(38)	C(37)	120.2(4)
C(14)	C(13)	C(12)	121.3(4)	C(40)	C(39)	C(26)	121.1(3)
C(13)	C(14)	C(15)	117.8(4)	C(40)	C(39)	C(44)	117.7(3)
C(10)	C(15)	N(1)	106.8(3)	C(44)	C(39)	C(26)	120.9(3)
C(14)	C(15)	N(1)	131.6(3)	C(41)	C(40)	C(39)	121.2(4)
C(14)	C(15)	C(10)	121.6(3)	C(42)	C(41)	C(40)	120.5(4)
C(17)	C(16)	C(8)	119.2(3)	C(41)	C(42)	C(43)	119.6(4)
C(25)	C(16)	C(8)	118.9(3)	C(44)	C(43)	C(42)	120.2(4)
C(25)	C(16)	C(17)	121.9(4)	C(43)	C(44)	C(39)	120.8(4)
C(16)	C(17)	C(22)	118.1(4)	Cl(2)	C(45)	Cl(1)	111.4(3)

Table S26.Torsion Angles for 55b.

A	B	C	D	Angle/˚	A	B	C	D	Angle/˚
C(1)	C(2)	C(3)	C(4)	179.5(4)	C(15)	N(1)	S(1)	C(5)	-87.4(3)
C(1)	C(2)	C(7)	C(6)	-179.5(4)	C(15)	N(1)	C(8)	C(9)	-1.3(4)
N(1)	S(1)	C(5)	C(4)	-105.7(3)	C(15)	N(1)	C(8)	C(16)	-178.5(3)
N(1)	S(1)	C(5)	C(6)	77.0(3)	C(15)	C(10)	C(11)	C(12)	0.5(5)
N(1)	C(8)	C(9)	S(2)	174.6(2)	C(16)	C(8)	C(9)	S(2)	-8.2(5)

N(1)	C(8)	C(9)	C(10)	0.7(4)	C(16)	C(8)	C(9)	C(10)	177.9(3)
N(1)	C(8)	C(16)	C(17)	-92.1(4)	C(16)	C(17)	C(18)	C(19)	179.5(4)
N(1)	C(8)	C(16)	C(25)	91.4(4)	C(16)	C(17)	C(22)	C(21)	-178.4(4)
O(1)	S(1)	C(5)	C(4)	141.5(3)	C(16)	C(17)	C(22)	C(23)	4.0(6)
O(1)	S(1)	C(5)	C(6)	-35.7(3)	C(17)	C(16)	C(25)	C(24)	3.0(6)
S(1)	N(1)	C(8)	C(9)	-177.0(2)	C(17)	C(18)	C(19)	C(20)	-2.1(7)
S(1)	N(1)	C(8)	C(16)	5.8(5)	C(17)	C(22)	C(23)	C(24)	-1.6(7)
S(1)	N(1)	C(15)	C(10)	177.1(2)	C(18)	C(17)	C(22)	C(21)	0.7(6)
S(1)	N(1)	C(15)	C(14)	-3.6(6)	C(18)	C(17)	C(22)	C(23)	-176.9(4)
S(1)	C(5)	C(6)	C(7)	177.5(3)	C(18)	C(19)	C(20)	C(21)	2.5(8)
C(2)	C(3)	C(4)	C(5)	0.4(6)	C(19)	C(20)	C(21)	C(22)	-1.3(8)
N(2)	S(2)	C(9)	C(8)	98.4(3)	C(20)	C(21)	C(22)	C(17)	-0.3(8)
N(2)	S(2)	C(9)	C(10)	-89.0(3)	C(20)	C(21)	C(22)	C(23)	177.1(5)
N(2)	C(26)	C(27)	C(28)	32.6(4)	C(21)	C(22)	C(23)	C(24)	-179.0(5)
N(2)	C(26)	C(27)	C(32)	-148.9(3)	C(22)	C(17)	C(18)	C(19)	0.5(6)
N(2)	C(26)	C(33)	C(34)	59.8(4)	C(22)	C(23)	C(24)	C(25)	-0.3(8)
N(2)	C(26)	C(33)	C(38)	-125.5(3)	C(23)	C(24)	C(25)	C(16)	-0.3(7)
N(2)	C(26)	C(39)	C(40)	-146.3(3)	C(25)	C(16)	C(17)	C(18)	176.2(4)
N(2)	C(26)	C(39)	C(44)	38.7(4)	C(25)	C(16)	C(17)	C(22)	-4.8(5)
O(2)	S(1)	C(5)	C(4)	8.3(3)	C(26)	N(2)	S(2)	O(3)	85.5(3)
O(2)	S(1)	C(5)	C(6)	-169.0(3)	C(26)	N(2)	S(2)	C(9)	-165.5(2)
S(2)	N(2)	C(26)	C(27)	136.8(2)	C(26)	C(27)	C(28)	C(29)	179.4(3)
S(2)	N(2)	C(26)	C(33)	19.4(3)	C(26)	C(27)	C(32)	C(31)	-178.4(4)
S(2)	N(2)	C(26)	C(39)	-102.0(3)	C(26)	C(33)	C(34)	C(35)	176.1(3)
S(2)	C(9)	C(10)	C(11)	7.4(6)	C(26)	C(33)	C(38)	C(37)	-174.6(3)
S(2)	C(9)	C(10)	C(15)	-173.1(3)	C(26)	C(39)	C(40)	C(41)	-174.9(3)
C(3)	C(2)	C(7)	C(6)	0.4(6)	C(26)	C(39)	C(44)	C(43)	175.0(3)

C(3)	C(4)	C(5)	S(1)	-177.5(3)	C(27)	C(26)	C(33)	C(34)	-56.8(4)
C(3)	C(4)	C(5)	C(6)	-0.3(6)	C(27)	C(26)	C(33)	C(38)	117.9(3)
O(3)	S(2)	C(9)	C(8)	-146.2(3)	C(27)	C(26)	C(39)	C(40)	-27.6(4)
O(3)	S(2)	C(9)	C(10)	26.3(3)	C(27)	C(26)	C(39)	C(44)	157.4(3)
C(4)	C(5)	C(6)	C(7)	0.3(6)	C(27)	C(28)	C(29)	C(30)	-0.7(6)
C(5)	C(6)	C(7)	C(2)	-0.3(6)	C(28)	C(27)	C(32)	C(31)	0.1(6)
C(7)	C(2)	C(3)	C(4)	-0.4(6)	C(28)	C(29)	C(30)	C(31)	-0.5(7)
C(8)	N(1)	S(1)	O(1)	-156.6(3)	C(29)	C(30)	C(31)	C(32)	1.5(7)
C(8)	N(1)	S(1)	O(2)	-27.7(3)	C(30)	C(31)	C(32)	C(27)	-1.3(7)
C(8)	N(1)	S(1)	C(5)	87.6(3)	C(32)	C(27)	C(28)	C(29)	0.9(6)
C(8)	N(1)	C(15)	C(10)	1.4(4)	C(33)	C(26)	C(27)	C(28)	150.0(3)
C(8)	N(1)	C(15)	C(14)	-179.3(4)	C(33)	C(26)	C(27)	C(32)	-31.6(4)
C(8)	C(9)	C(10)	C(11)	-179.4(4)	C(33)	C(26)	C(39)	C(40)	93.8(4)
C(8)	C(9)	C(10)	C(15)	0.1(4)	C(33)	C(26)	C(39)	C(44)	-81.2(4)
C(8)	C(16)	C(17)	C(18)	-0.2(5)	C(33)	C(34)	C(35)	C(36)	-1.9(6)
C(8)	C(16)	C(17)	C(22)	178.8(3)	C(34)	C(33)	C(38)	C(37)	0.1(5)
C(8)	C(16)	C(25)	C(24)	179.3(4)	C(34)	C(35)	C(36)	C(37)	1.3(7)
C(9)	C(8)	C(16)	C(17)	91.2(4)	C(35)	C(36)	C(37)	C(38)	-0.1(6)
C(9)	C(8)	C(16)	C(25)	-85.3(5)	C(36)	C(37)	C(38)	C(33)	-0.7(6)
C(9)	C(10)	C(11)	C(12)	180.0(4)	C(38)	C(33)	C(34)	C(35)	1.2(5)
C(9)	C(10)	C(15)	N(1)	-0.9(4)	C(39)	C(26)	C(27)	C(28)	-87.4(4)
C(9)	C(10)	C(15)	C(14)	179.7(3)	C(39)	C(26)	C(27)	C(32)	91.1(4)
C(10)	C(11)	C(12)	C(13)	-0.5(6)	C(39)	C(26)	C(33)	C(34)	-179.8(3)
C(11)	C(10)	C(15)	N(1)	178.7(3)	C(39)	C(26)	C(33)	C(38)	-5.1(4)
C(11)	C(10)	C(15)	C(14)	-0.7(5)	C(39)	C(40)	C(41)	C(42)	-0.3(6)
C(11)	C(12)	C(13)	C(14)	0.6(7)	C(40)	C(39)	C(44)	C(43)	-0.2(6)
C(12)	C(13)	C(14)	C(15)	-0.8(6)	C(40)	C(41)	C(42)	C(43)	0.3(7)

C(13)	C(14)	C(15)	N(1)	-178.4(4)	C(41)	C(42)	C(43)	C(44)	-0.3(7)
C(13)	C(14)	C(15)	C(10)	0.9(6)	C(42)	C(43)	C(44)	C(39)	0.2(6)
C(15)	N(1)	S(1)	O(1)	28.4(3)	C(44)	C(39)	C(40)	C(41)	0.3(6)
C(15)	N(1)	S(1)	O(2)	157.3(3)					

Table S27. Hydrogen Atom Coordinates (Å×10^4) and Isotropic Displacement Parameters (Å^2×10^3) for 55b.

Atom	*x*	*y*	*z*	U(eq)
H(1A)	-1370.24	4031.97	6595.33	82
H(1B)	-305.87	3448.26	6919.27	82
H(1C)	-832.21	3080.52	6424.57	82
H(2)	6749.67	4964.84	3841.83	29
H(3)	-208.73	5127.23	6145.2	55
H(4)	1893.17	5768.07	5806.35	49
H(6)	4364.19	3655.91	6194.68	49
H(7)	2245.15	3031.61	6531.1	55
H(11)	6863.35	2821.72	4236.14	44
H(12)	8215.95	2010.68	4772.59	57
H(13)	8292.42	2421.75	5554.57	59
H(14)	6967.5	3641.16	5819.83	49
H(18)	2004.45	4371.61	4899.08	51
H(19)	-594.47	4286.76	4936.64	71
H(20)	-2029.21	5559.41	4824.9	83
H(21)	-931.59	6856.93	4623.69	77
H(23)	1367.29	7708.1	4433.89	73
H(24)	3924.5	7810.16	4356.34	72

H(25)	5438.43	6556.06	4519.4	54
H(28)	8125.41	5960.32	3745.01	43
H(29)	9193.01	7345.31	3789.86	54
H(30)	8387.21	8481.6	3308.96	58
H(31)	6505.97	8207.32	2766.83	62
H(32)	5374.23	6827.26	2734.65	52
H(34)	3697.87	6209.86	3605.47	47
H(35)	1235.55	6475.08	3385.2	61
H(36)	328.97	5929.48	2681.96	69
H(37)	1853.01	5069.47	2216.84	60
H(38)	4308.41	4748.81	2445.3	43
H(40)	7640.83	5716.41	2462.54	41
H(41)	8759.15	4818.24	1906.88	55
H(42)	8568.89	3299.53	1958.94	58
H(43)	7221.59	2664.88	2570.39	55
H(44)	6093.3	3555.84	3129.18	43
H(45A)	362.95	3898.19	3004.52	94
H(45B)	1893.67	3740.76	3281.35	94

7 Computational Details

Density functional theory (DFT) calculations were performed to elucidate the detailed mechanism of asymmetric copper-catalyzed cyclizative sulfinamidation using the Gaussian 09 package.[11] The geometric optimizations of all intermediates and transition states were carried out at the B3LYP[12-13] level of theory with Grimme D3 correction[14] and Becke-Johnson damping[15] in the gas phase. The SDD[16-17] basis set is used for Cu and Fe atoms, and 6-31G(d)[18-20] basis set is employed for the other atoms. Vibrational frequency calculations were calculated at the same level for all stationary

points to confirm whether each optimized structure represents a local minimum (no imaginary frequencies) or a transition state (with only one imaginary frequency corresponding to the expected bond breaking or forming process), while the thermal corrections to the free energy (298.15 K, 1.0 atm) were also obtained. To obtain higher accuracy in the energy calculations, solvation single-point energy were calculated in 1,3-dioxolane with IEFPCM[21-22] continuum solvent model at the M06[23] /6-311+G(d,p)[24-25]-SDD level of theory, in which the key word int=ultrafine was used to enhance the integration grid. The calculated 3D optimized structures are visualized using CYLview program.[26]

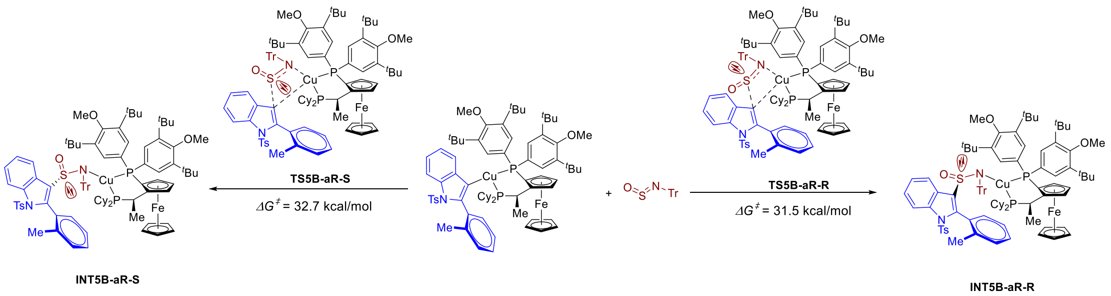

Figure S2. The migratory insertion of the S=N bond of reagent I into INT4A-aR.

Table S28. The calculated energies of stationary points (in Hartree/Particle)

Structure	ZPE	H_{corr}	G_{corr}	E_{ele}	H_{sol}	G_{sol}
**32a**	0.356387	0.380566	0.302835	-1452.580259	-1452.199693	-1452.277424
**INT1A**	1.240463	1.308618	1.138053	-5833.397968	-5832.089350	-5832.259915
**INT2A**	1.600102	1.692792	1.473314	-7286.009429	-7284.316637	-7284.536115
[LiO^{t}Bu]$_8$	1.031449	1.097688	0.937196	-1924.914928	-1923.817240	-1923.977732
1,3-dioxolane	0.093030	0.097341	0.066496	-268.257140	-268.159799	-268.190644
**INT3A**	1.584784	1.675466	1.457073	-4711.304889	-4709.629423	-4709.847816
**complex**	0.732482	0.783360	0.646372	-6648.351813	-6647.568453	-6647.705441
**TS4A-aR**	1.584511	1.674383	1.458690	-4711.301180	-4709.626797	-4709.842490
**INT4A-aR**	1.587263	1.675841	1.464402	-4711.342980	-4709.667139	-4709.878578
**TS4A-aS**	1.584416	1.674291	1.457791	-4711.297440	-4709.623149	-4709.839649

**INT4A-aS**	1.588132	1.677148	1.464896	-4711.341404	-4709.664256	-4709.876508
**I**	0.290433	0.309463	0.242502	-1260.722378	-1260.412915	-1260.479876
**TS5A-aR-R**	1.881137	1.988805	1.737840	-5972.067989	-5970.079184	-5970.330149
**INT5A-aR-R**	1.882174	1.990118	1.737648	-5972.097422	-5970.107304	-5970.359774
**TS5A-aR-S**	1.881536	1.989083	1.737718	-5972.070992	-5970.081909	-5970.333274
**INT5A-aR-S**	1.881158	1.989145	1.736436	-5972.095126	-5970.105981	-5970.358690
**TS5B-aR-R**	1.881523	1.988906	1.740053	-5972.048329	-5970.059423	-5970.308276
**INT5B-aR-R**	1.882944	1.991086	1.738262	-5972.101755	-5970.110669	-5970.363493
**TS5B-aR-S**	1.882190	1.989363	1.740740	-5972.047815	-5970.058452	-5970.307075
**INT5B-aR-S**	1.882665	1.990385	1.738796	-5972.111618	-5970.121233	-5970.372822
R-**32b-Li**	0.737516	0.786660	0.651206	-2988.642796	-2987.856136	-2987.991590
$[LiO^tBu\text{-}^tBuOH]_2$	0.533160	0.565789	0.470217	-948.385765	-947.819976	-947.915548

Note: ZPE = zero-point vibrational energy; H_{corr} = thermal correction to enthalpy; G_{corr} = thermal correction to Gibbs free energy; E_{ele} = the electronic energies in solvent; H_{sol} = ethalpies in solvent; G_{sol} = Gibbs free energies in solvent.

Cartesian coordinates (unit: angstrom)

32a

C	-4.349170	2.020203	0.118517
C	-3.943383	0.801330	-0.419902
C	-2.615945	0.616726	-0.809468
C	-1.677196	1.665848	-0.650597
C	-2.114532	2.899674	-0.136301
C	-3.438710	3.073302	0.249291
H	-5.383240	2.153606	0.421523
H	-4.637255	-0.021563	-0.540824
H	-1.394380	3.704129	-0.028102
H	-3.762179	4.027703	0.653591
C	-0.307903	1.429721	-0.946192
C	0.850390	1.106092	-1.128127
C	2.215586	0.717110	-1.196637
C	3.160996	1.339521	-0.345150
C	2.612287	-0.333045	-2.042203
C	4.476440	0.873762	-0.366353
C	3.929687	-0.780037	-2.042948
H	1.871775	-0.799959	-2.684117
C	4.864595	-0.177101	-1.200405
H	5.209835	1.342838	0.284807
H	4.224402	-1.595724	-2.696336
H	5.895024	-0.520432	-1.193172
S	-2.043776	-1.977426	-0.330715
O	-1.599632	-3.060660	-1.206901
O	-3.271688	-2.052402	0.458560
C	-0.690792	-1.540270	0.747034
C	0.596355	-1.969571	0.420094
C	-0.915889	-0.679874	1.821629
C	1.671207	-1.523376	1.179348
H	0.742887	-2.635089	-0.422902
C	0.175482	-0.239177	2.567291
H	-1.924102	-0.363069	2.062942
C	1.479059	-0.648990	2.258026
H	2.677503	-1.837092	0.916679
H	0.012399	0.435374	3.403638
C	2.660582	-0.177958	3.067664
H	2.402682	0.680774	3.694630
H	3.026400	-0.974040	3.728424
H	3.490521	0.109179	2.413272
N	-2.214992	-0.624711	-1.370132
H	-1.359050	-0.546874	-1.918968
C	2.734407	2.449814	0.576641
H	3.577781	2.818426	1.167453
H	2.311211	3.291655	0.016312
H	1.952383	2.104222	1.262728

INT1A

C	-0.973741	-3.649859	0.940255
C	0.025472	-3.967401	1.907170
H	1.091041	-3.985369	1.726762
C	-0.626229	-4.195692	3.157092
H	-0.140197	-4.423582	4.096697
C	-2.029097	-4.007648	2.963984
H	-2.790116	-4.075272	3.730434
C	-2.240517	-3.667091	1.593995
H	-3.188723	-3.441133	1.126739

C	-0.407509	-0.428225	1.835580	C	4.986169	0.524357	2.435448
C	0.398931	-0.835937	2.952772	C	5.990301	-1.748936	2.295758
C	-0.447279	-1.039473	4.074359	H	7.075243	-0.756959	0.019737
H	-0.140278	-1.399377	5.047580	H	6.139463	0.746480	-0.132799
C	-1.781610	-0.762529	3.665490	H	7.274813	0.528498	1.210157
H	-2.665576	-0.857066	4.283440	H	4.195281	0.186565	3.114072
C	-1.788974	-0.383907	2.282551	H	5.845306	0.815922	3.048858
Fe	-1.000027	-2.275517	2.507518	H	4.632153	1.422055	1.915288
C	1.849928	-1.025070	0.100585	H	6.835825	-1.399154	2.900729
C	2.968810	-0.520835	0.772734	H	5.225913	-2.140056	2.977397
C	1.979230	-2.166235	-0.683027	H	6.334881	-2.563991	1.657248
C	4.228449	-1.098811	0.623117	C	3.187841	-4.137403	-1.663695
H	2.844609	0.354953	1.395574	C	1.999444	-5.000580	-1.164032
C	3.211498	-2.818607	-0.853250	C	2.942299	-3.823158	-3.157272
H	1.104694	-2.533398	-1.206795	C	4.443199	-5.021212	-1.508954
C	4.337983	-2.202220	-0.268829	H	2.090320	-5.212957	-0.093124
C	0.705712	1.558930	0.094683	H	1.029529	-4.533234	-1.346407
C	1.321631	2.028510	-1.071162	H	1.999209	-5.957146	-1.698086
C	0.465486	2.446949	1.135999	H	3.735591	-3.195090	-3.577317
C	1.807842	3.331017	-1.171457	H	2.910036	-4.755206	-3.734077
H	1.434614	1.342347	-1.900943	H	1.986838	-3.307254	-3.298373
C	0.885466	3.787868	1.084570	H	4.229815	-6.001058	-1.951217
H	-0.049904	2.082742	2.016344	H	5.323183	-4.625023	-2.014822
C	1.656305	4.169932	-0.033526	H	4.700024	-5.173711	-0.456571
H	-0.817040	-3.424784	-0.105472	C	0.455701	4.705904	2.253882
P	0.220132	-0.204484	0.149665	C	-1.034864	4.430259	2.578568
H	1.463070	-1.015946	2.912050	C	1.284769	4.371620	3.513719
C	5.422206	-0.583194	1.454884	C	0.541779	6.217344	1.954021
C	6.543060	0.013726	0.575972	H	-1.663204	4.583366	1.693967

H	-1.207397	3.416707	2.947649	H	7.140953	-2.565525	-1.882681
H	-1.374107	5.119487	3.359490	H	5.531427	-2.651493	-2.646212
H	2.351765	4.560574	3.360522	C	-3.091869	0.073232	1.639568
H	0.951982	4.982358	4.361499	H	-3.826968	-0.710026	1.864528
H	1.170966	3.317398	3.789065	C	-3.567217	1.370010	2.325288
H	0.035338	6.761301	2.759733	H	-4.580156	1.638759	2.019918
H	1.561860	6.593558	1.898551	H	-3.565956	1.244326	3.412851
H	0.042957	6.464420	1.012218	H	-2.903187	2.205769	2.091563
C	2.437605	3.826560	-2.491351	P	-3.115620	0.140135	-0.242334
C	3.949269	4.102084	-2.331924	C	-2.804193	1.921340	-0.740357
C	2.282839	2.778091	-3.611824	C	-2.191207	1.931184	-2.157950
C	1.725436	5.113446	-2.967462	C	-3.971895	2.922159	-0.674329
H	4.139145	4.986645	-1.724066	H	-2.019888	2.254628	-0.047217
H	4.459333	3.244712	-1.877016	C	-1.737570	3.338853	-2.559621
H	4.398182	4.275825	-3.317010	H	-2.933151	1.557639	-2.878240
H	1.234494	2.509629	-3.780098	H	-1.341337	1.241133	-2.206885
H	2.672378	3.196415	-4.545728	C	-3.515250	4.333852	-1.077013
H	2.844505	1.860622	-3.404101	H	-4.766709	2.594918	-1.358324
H	2.181512	5.463748	-3.901276	H	-4.408180	2.955172	0.326432
H	0.666205	4.918403	-3.164558	C	-2.885044	4.349934	-2.473689
H	1.801542	5.909078	-2.225309	H	-1.317925	3.317101	-3.573034
O	5.613749	-2.661031	-0.552618	H	-0.926571	3.649487	-1.888441
O	2.293084	5.399663	-0.061646	H	-4.366045	5.024904	-1.028705
C	3.506346	5.434753	0.688153	H	-2.776347	4.691493	-0.345053
H	4.020955	6.355245	0.401859	H	-2.526554	5.357422	-2.718256
H	3.315801	5.451122	1.766137	H	-3.652383	4.096625	-3.219763
H	4.140472	4.572352	0.455270	C	-4.866674	-0.298059	-0.728671
C	6.105276	-2.223056	-1.819446	C	-4.963906	-1.832766	-0.864024
H	6.074013	-1.130688	-1.899066	C	-6.022224	0.240032	0.133530

H	-4.959112	0.129018	-1.739193
C	-6.327985	-2.265370	-1.416002
H	-4.819939	-2.284737	0.129541
H	-4.154111	-2.205697	-1.499784
C	-7.384784	-0.192080	-0.431182
H	-5.924089	-0.152957	1.154924
H	-5.981969	1.329945	0.206443
C	-7.481021	-1.715992	-0.567849
H	-6.375624	-3.359586	-1.469040
H	-6.426575	-1.896701	-2.447261
H	-8.188670	0.188499	0.211306
H	-7.525670	0.270589	-1.418901
H	-8.447070	-1.998617	-1.003639
H	-7.441918	-2.170311	0.433432
Cu	-1.424099	-1.014364	-1.165327
Br	-1.188602	-2.642694	-2.813800

INT2A

C	-1.469985	-0.307395	3.974759
C	-1.214820	-1.684234	4.264275
H	-1.462158	-2.521517	3.626509
C	-0.518013	-1.751930	5.507949
H	-0.146206	-2.651600	5.980717
C	-0.333381	-0.418653	5.983848
H	0.197600	-0.132336	6.882278
C	-0.926093	0.470792	5.036020
H	-0.927635	1.551521	5.085905
C	1.424425	-0.577043	2.268537
C	1.900440	-1.710025	3.016548
C	2.543114	-1.236700	4.191871
H	2.969179	-1.846062	4.978005
C	2.454054	0.186190	4.194512
H	2.801364	0.828222	4.990841
C	1.746346	0.618359	3.027056
Fe	0.560067	-0.648191	4.131816
C	-0.317789	-2.110843	0.497635
C	0.158646	-3.377915	0.163508
C	-1.682726	-1.934677	0.732973
C	-0.699269	-4.465153	-0.007437
H	1.218107	-3.504307	0.003003
C	-2.594196	-2.998188	0.644520
H	-2.033204	-0.940431	0.978262
C	-2.085787	-4.240081	0.184774
C	2.258511	-1.245569	-0.384928
C	2.303473	-0.946749	-1.753662
C	3.283651	-2.022404	0.147672
C	3.219918	-1.552332	-2.616387
H	1.593796	-0.231365	-2.143150
C	4.276230	-2.614944	-0.649483
H	3.315190	-2.179743	1.216046
C	4.138720	-2.470654	-2.045854
H	-1.928639	0.098761	3.084655
P	0.790184	-0.654900	0.568617
H	1.750856	-2.743237	2.733702
C	-0.110341	-5.842377	-0.382662
C	-0.536562	-6.252406	-1.808826
C	1.432608	-5.819263	-0.371351
C	-0.552058	-6.915696	0.636832

H	-1.606035	-6.443483	-1.879679	H	4.468727	-5.323360	0.065901
H	-0.275305	-5.469013	-2.530356	H	5.673585	-5.109650	1.348366
H	-0.015396	-7.170709	-2.105460	H	4.074503	-4.359319	1.493349
H	1.830681	-5.529467	0.606546	H	7.447868	-4.050118	-0.110221
H	1.804692	-6.824769	-0.595998	H	6.439263	-4.554045	-1.460288
H	1.847469	-5.137883	-1.122583	H	6.998175	-2.881148	-1.364478
H	-0.119532	-7.887422	0.368370	C	3.253867	-1.178724	-4.113671
H	-0.197105	-6.655419	1.640938	C	2.936834	-2.385791	-5.023987
H	-1.637172	-7.012000	0.670561	C	2.222102	-0.085076	-4.446988
C	-4.067830	-2.748120	1.052477	C	4.654380	-0.624660	-4.460065
C	-4.160766	-1.615162	2.100706	H	3.749320	-3.111190	-5.030974
C	-4.896579	-2.282856	-0.162324	H	2.014005	-2.885877	-4.707614
C	-4.713823	-3.981829	1.722213	H	2.791188	-2.039413	-6.053672
H	-3.590155	-1.850423	3.002319	H	2.371503	0.815594	-3.847182
H	-3.827612	-0.647868	1.717805	H	2.330754	0.199951	-5.498506
H	-5.209871	-1.495042	2.390157	H	1.190193	-0.420295	-4.301969
H	-4.839539	-2.970482	-1.006133	H	4.697888	-0.353503	-5.521740
H	-5.948392	-2.172933	0.120798	H	4.868821	0.274780	-3.872218
H	-4.542622	-1.310919	-0.512372	H	5.433023	-1.363327	-4.259906
H	-5.691887	-3.693420	2.124910	O	-2.924258	-5.317860	-0.042344
H	-4.861938	-4.823288	1.049135	O	4.922776	-3.220490	-2.903978
H	-4.098036	-4.333643	2.557705	C	4.461061	-4.564173	-3.047941
C	5.423079	-3.344407	0.092203	H	5.129775	-5.045334	-3.765245
C	5.977801	-2.384636	1.175456	H	4.494597	-5.106459	-2.097583
C	4.874913	-4.608604	0.788789	H	3.434044	-4.588247	-3.429081
C	6.638552	-3.729488	-0.776581	C	-3.712923	-5.309079	-1.229727
H	6.396909	-1.486739	0.710259	H	-3.293173	-4.632412	-1.979393
H	5.219976	-2.069411	1.897303	H	-3.723612	-6.330184	-1.622805
H	6.778354	-2.882523	1.734013	H	-4.743000	-5.010153	-1.011646

C	1.351518	2.058906	2.739389	H	0.232838	4.750970	2.268570
H	0.257398	2.092450	2.691459	H	-0.415746	4.328877	0.704226
C	1.794270	3.009110	3.860177	C	2.707713	6.714466	0.993237
H	1.428548	4.020065	3.680563	H	2.981550	5.101320	2.410539
H	1.373725	2.672584	4.814684	H	3.860593	4.892322	0.900339
H	2.880450	3.060923	3.967796	C	1.353047	7.225994	1.495398
P	1.799848	2.525561	0.968354	H	-0.759496	6.745414	1.271439
C	3.531684	1.869830	0.742855	H	0.198602	6.549704	-0.193963
C	4.022830	2.069358	-0.699074	H	3.528396	7.302143	1.423665
C	4.604971	2.260098	1.774682	H	2.759498	6.844687	-0.097407
H	3.372576	0.791385	0.860197	H	1.231474	8.287809	1.247627
C	5.330087	1.304426	-0.942344	H	1.321191	7.152222	2.592818
H	4.165370	3.138768	-0.907921	Cu	0.182219	1.394931	-0.199758
H	3.256386	1.707427	-1.389295	Br	-2.054204	1.914217	0.993079
C	5.907012	1.488706	1.515762	C	-0.673806	1.093534	-2.164425
H	4.808193	3.334602	1.734806	C	-0.090864	2.183693	-2.211801
H	4.249999	2.035871	2.783887	C	-3.277273	-2.018333	-3.340262
C	6.408449	1.684730	0.079578	C	-3.790278	-0.760707	-3.032371
H	5.688541	1.492277	-1.962212	C	-2.947576	0.248001	-2.553921
H	5.121966	0.228287	-0.876604	C	-1.552526	-0.003763	-2.455485
H	6.673983	1.803793	2.234487	C	-1.071204	-1.297347	-2.713794
H	5.726017	0.421613	1.695604	C	-1.924361	-2.302853	-3.152266
H	7.317437	1.093786	-0.089925	H	-3.952510	-2.785642	-3.708078
H	6.687592	2.739023	-0.064069	H	-4.845086	-0.563072	-3.161323
C	1.757776	4.380283	0.754961	H	-0.018726	-1.506090	-2.580652
C	0.372358	4.907017	1.192371	H	-1.530033	-3.292923	-3.358285
C	2.909081	5.227055	1.323744	S	-4.946289	2.134833	-2.337550
H	1.800973	4.477654	-0.338092	O	-5.435288	1.747176	-3.661658
C	0.210522	6.401811	0.891990	O	-4.824634	3.535273	-1.936120

C	-5.949641	1.303302	-1.115110
C	-7.029641	0.525672	-1.526664
C	-5.662765	1.490964	0.239199
C	-7.829244	-0.084802	-0.560633
H	-7.240440	0.417021	-2.584726
C	-6.471303	0.872335	1.185558
H	-4.810575	2.087635	0.547157
C	-7.559153	0.072631	0.803648
H	-8.673693	-0.693798	-0.872436
H	-6.247686	1.005041	2.240370
C	-8.387226	-0.635427	1.846016
H	-7.866711	-1.531501	2.210203
H	-8.571718	0.005666	2.714555
H	-9.353302	-0.955634	1.443963
N	-3.389141	1.525246	-2.168267
H	-2.912750	1.913388	-1.345358
C	0.468513	3.467880	-2.521860
C	1.800471	3.570596	-2.961367
C	-0.322025	4.635902	-2.385246
C	2.364978	4.807933	-3.250541
H	2.389497	2.669435	-3.065915
C	0.266945	5.864820	-2.699585
C	1.594299	5.964116	-3.117303
H	3.397042	4.866252	-3.583631
H	-0.335836	6.764300	-2.608127
H	2.020407	6.937342	-3.343241
C	-1.750367	4.557400	-1.928457
H	-2.379157	4.041760	-2.660716
H	-1.844979	3.999240	-0.991066
H	-2.170889	5.554823	-1.775003

[LiO^{t}Bu]$_8$

Li	-2.350224	-0.994567	-0.541051
Li	-2.322007	1.046598	0.670410
Li	-0.716893	-1.342878	1.261131
Li	-0.754249	1.338080	-1.200371
O	-0.782720	-2.022477	-0.561510
O	-0.727328	2.014361	0.623530
O	-2.560083	0.743075	-1.214681
O	-2.496079	-0.713900	1.348627
C	-1.182185	-3.369040	-0.750697
C	-2.642927	-3.415171	-1.248650
H	-2.987191	-4.442171	-1.415286
H	-3.329142	-2.968976	-0.517665
H	-2.731112	-2.876390	-2.200380
C	-1.072140	-4.128745	0.583211
H	-1.347732	-5.185678	0.485874
H	-0.044051	-4.077003	0.959365
H	-1.736716	-3.676266	1.329987
C	-0.297571	-4.037991	-1.813613
H	0.745026	-4.059697	-1.486261
H	-0.607697	-5.070462	-2.014866
H	-0.353524	-3.475479	-2.753401
C	-0.945659	3.408691	0.761830
C	-0.078883	3.952982	1.908598
H	-0.235488	5.025790	2.073134
H	-0.320432	3.424345	2.838825
H	0.982397	3.800454	1.691430

C	-2.424797	3.678650	1.102361	H	-2.338892	-0.847604	4.036177
H	-2.639259	4.751436	1.170901	C	-4.674935	-1.729686	1.641362
H	-3.083619	3.262861	0.330401	H	-5.016747	-1.320148	0.685329
H	-2.682060	3.226664	2.068048	H	-4.284316	-2.738210	1.465932
C	-0.587627	4.117467	-0.557021	H	-5.544629	-1.815275	2.303979
H	-0.667972	5.208356	-0.477942	Li	2.415681	0.535121	-1.072631
H	0.436967	3.866902	-0.848935	Li	0.767213	-1.279405	-1.241205
H	-1.263613	3.795340	-1.358181	Li	0.844497	1.266339	1.263514
C	-3.590909	1.000845	-2.144462	Li	2.475990	-0.531018	1.015248
C	-3.421418	0.097255	-3.379575	O	2.586247	-1.312666	-0.690618
H	-4.206343	0.271193	-4.125471	O	0.847407	-0.557426	1.925140
H	-2.451929	0.272810	-3.854002	O	2.635341	1.321089	0.629112
H	-3.463180	-0.959183	-3.087668	O	0.755980	0.539154	-1.933861
C	-3.558872	2.474592	-2.587043	C	3.505644	-2.377325	-0.793846
H	-4.339598	2.692403	-3.325619	C	3.126660	-3.484366	0.208632
H	-3.711690	3.135761	-1.727731	H	3.790901	-4.353730	0.135130
H	-2.591915	2.723556	-3.037456	H	3.183575	-3.104253	1.236808
C	-4.958896	0.709700	-1.502023	H	2.098416	-3.815891	0.042083
H	-5.787879	0.917987	-2.188839	C	4.924023	-1.878053	-0.467188
H	-5.024943	-0.343467	-1.205313	H	5.666370	-2.682454	-0.533063
H	-5.097546	1.323958	-0.604605	H	5.216619	-1.080453	-1.158274
C	-3.577733	-0.837359	2.247595	H	4.962055	-1.468456	0.550232
C	-4.162066	0.559150	2.541239	C	3.497358	-2.941551	-2.225414
H	-5.008276	0.519702	3.236781	H	3.794252	-2.163963	-2.938116
H	-3.392369	1.200834	2.988601	H	4.187218	-3.786720	-2.336664
H	-4.523046	1.030294	1.617007	H	2.494595	-3.284834	-2.500098
C	-3.112969	-1.464665	3.570396	C	1.126738	-0.758368	3.300811
H	-3.940642	-1.571021	4.282056	C	0.487555	0.375915	4.120114
H	-2.696061	-2.461789	3.386831	H	0.600720	0.227054	5.200736

H	0.953662	1.336946	3.865384
H	-0.578786	0.446826	3.883483
C	2.653021	-0.754224	3.531681
H	2.908897	-0.871669	4.590656
H	3.127076	-1.589657	2.994959
H	3.092086	0.186888	3.184175
C	0.577114	-2.124646	3.743294
H	0.796286	-2.335615	4.796988
H	-0.508436	-2.167046	3.618358
H	1.025397	-2.917609	3.133047
C	3.653483	2.296936	0.702672
C	3.310149	3.486401	-0.213116
H	4.107297	4.239288	-0.218932
H	3.160289	3.143655	-1.244798
H	2.385718	3.970087	0.111395
C	4.991799	1.691041	0.242384
H	5.805443	2.425216	0.278511
H	5.267081	0.842946	0.878018
H	4.917602	1.324220	-0.789737
C	3.810017	2.796893	2.149966
H	4.550883	3.601950	2.224044
H	2.856145	3.176886	2.531310
H	4.133795	1.980571	2.804445
C	1.000920	0.630604	-3.327437
C	0.542899	-0.666737	-4.017119
H	0.645958	-0.617027	-5.107806
H	1.144557	-1.515884	-3.668673
H	-0.505934	-0.865835	-3.774991
C	2.511157	0.833564	-3.577268
H	2.746902	0.902222	-4.645227
H	2.853105	1.766663	-3.107619
H	3.087087	-0.006777	-3.169265
C	0.242132	1.834849	-3.905699
H	-0.836110	1.704875	-3.770482
H	0.547255	2.751369	-3.386254
H	0.430617	1.968238	-4.977696

1,3-dioxolane

C	-0.966693	0.773007	0.000000
O	0.404224	1.150225	0.000000
C	1.218575	-0.001652	0.000000
C	-0.969080	-0.770099	0.000000
H	-1.458265	1.186359	0.889326
H	-1.458265	1.186358	-0.889327
H	1.854971	-0.002923	-0.898310
H	1.854972	-0.002922	0.898308
H	-1.462447	-1.181432	-0.889352
H	-1.462448	-1.181432	0.889352
O	0.400110	-1.151668	0.000001

INT3A

C	0.869774	0.390994	-4.460428
C	2.055081	1.184838	-4.370280
H	2.198344	2.029763	-3.712381
C	3.024019	0.623862	-5.251952
H	4.040061	0.972557	-5.381885
C	2.444725	-0.518049	-5.882352
H	2.941354	-1.181217	-6.578293

C	1.109620	-0.658722	-5.395102	C	4.254148	4.392975	-0.204277
H	0.417612	-1.449036	-5.655526	C	4.008633	5.306831	1.016107
C	2.351248	-1.092063	-1.804624	C	5.354464	3.391146	0.204234
C	3.709538	-0.858211	-2.213018	C	4.805689	5.218582	-1.387890
C	4.077806	-1.865621	-3.143008	H	3.381745	6.161194	0.766979
H	5.022915	-1.940015	-3.664237	H	3.526549	4.749458	1.827430
C	2.953759	-2.719994	-3.330926	H	4.964772	5.692478	1.390023
H	2.917150	-3.548386	-4.022592	H	5.592072	2.689601	-0.603900
C	1.864216	-2.251710	-2.526424	H	6.269881	3.943671	0.440687
Fe	2.450839	-0.771229	-3.825768	H	5.081135	2.811475	1.093199
C	1.708362	1.598178	-0.846250	H	5.729689	5.729040	-1.090125
C	2.867522	2.282595	-0.486275	H	5.040742	4.562926	-2.234966
C	0.649926	2.293897	-1.427047	H	4.084902	5.966115	-1.720044
C	2.958957	3.668044	-0.622178	C	-0.469709	4.328514	-2.440766
H	3.683602	1.728105	-0.044660	C	-1.059317	3.322102	-3.459490
C	0.696294	3.674195	-1.657182	C	-1.624988	4.719603	-1.493292
H	-0.243314	1.742294	-1.690523	C	-0.002071	5.533473	-3.287576
C	1.829814	4.355560	-1.144902	H	-0.296233	2.951472	-4.151492
C	2.390735	-0.535946	1.019122	H	-1.546864	2.469796	-2.977170
C	1.643071	-0.595755	2.202011	H	-1.832481	3.828620	-4.045655
C	3.767460	-0.726105	1.099629	H	-1.304110	5.359786	-0.671724
C	2.239575	-0.644870	3.460934	H	-2.409193	5.241452	-2.052242
H	0.565722	-0.560454	2.128158	H	-2.085905	3.833529	-1.052311
C	4.430953	-0.858328	2.328978	H	-0.832855	5.861421	-3.921686
H	4.344927	-0.784680	0.187567	H	0.325150	6.386094	-2.697597
C	3.657010	-0.670424	3.497346	H	0.829565	5.247920	-3.942794
H	-0.038002	0.546156	-3.895005	C	5.932821	-1.227218	2.270995
P	1.469611	-0.170334	-0.524529	C	6.083742	-2.449117	1.326868
H	4.318193	-0.021805	-1.898770	C	6.741822	-0.048581	1.686994

C	6.559120	-1.660954	3.612640	C	1.077685	6.435419	-0.227634
H	5.517360	-3.304178	1.712040	H	0.780101	5.785025	0.598504
H	5.738334	-2.248534	0.309067	H	1.666414	7.275275	0.152235
H	7.138421	-2.739933	1.266370	H	0.183714	6.827165	-0.719835
H	6.670089	0.841512	2.320632	C	0.454613	-2.833612	-2.566280
H	7.801007	-0.318797	1.603321	H	-0.195711	-2.075053	-3.017253
H	6.384976	0.228693	0.689282	C	0.388114	-4.070439	-3.474158
H	7.551074	-2.080357	3.408795	H	-0.634986	-4.428876	-3.580303
H	6.693890	-0.838552	4.314517	H	0.745725	-3.808562	-4.475956
H	5.959595	-2.430581	4.105851	H	0.998673	-4.894970	-3.098493
C	1.357753	-0.662793	4.726901	P	-0.299748	-2.996783	-0.841360
C	1.471822	0.672560	5.495708	C	1.021874	-3.747107	0.231015
C	-0.132531	-0.845415	4.368482	C	0.475317	-4.029907	1.642340
C	1.765011	-1.836645	5.643804	C	1.833788	-4.933489	-0.315970
H	2.455449	0.796943	5.949710	H	1.729596	-2.916503	0.337637
H	1.269177	1.522310	4.834001	C	1.623681	-4.377843	2.598756
H	0.730657	0.694702	6.302967	H	-0.240568	-4.862360	1.608126
H	-0.305499	-1.760598	3.790894	H	-0.072656	-3.155972	2.013790
H	-0.710421	-0.926739	5.294865	C	2.977332	-5.271861	0.653725
H	-0.535506	0.002545	3.805495	H	1.193952	-5.812863	-0.451140
H	1.134470	-1.840641	6.540687	H	2.251525	-4.680844	-1.294625
H	1.624858	-2.794101	5.130333	C	2.456788	-5.551971	2.069170
H	2.807883	-1.759404	5.953962	H	1.223073	-4.611328	3.592594
O	1.903833	5.731292	-1.163997	H	2.264982	-3.494182	2.714639
O	4.274297	-0.512980	4.722575	H	3.543639	-6.131859	0.275848
C	4.823476	0.793087	4.910933	H	3.673883	-4.422456	0.685221
H	5.189059	0.826672	5.939445	H	3.294017	-5.756968	2.747306
H	5.653607	0.984055	4.223012	H	1.835528	-6.459437	2.052497
H	4.060159	1.564809	4.764774	C	-1.779862	-4.127372	-0.913522

C	-2.792466	-3.582286	-1.943521	S	-4.838032	3.379966	0.711612
C	-1.540330	-5.640235	-1.069349	O	-4.956098	3.892791	-0.673748
H	-2.233717	-3.964931	0.075410	O	-5.059615	4.356933	1.804625
C	-4.096434	-4.389790	-1.923345	C	-6.094755	2.105074	0.890183
H	-2.364831	-3.622346	-2.953021	C	-7.075708	1.962955	-0.083936
H	-3.004767	-2.531771	-1.726711	C	-6.101976	1.296515	2.029386
C	-2.869115	-6.411719	-1.053979	C	-8.074397	0.999736	0.084641
H	-1.013525	-5.850337	-2.006034	H	-7.039599	2.597966	-0.962416
H	-0.898009	-6.001729	-0.260805	C	-7.100651	0.344121	2.186291
C	-3.835732	-5.886705	-2.121462	H	-5.316862	1.408973	2.769162
H	-4.774679	-4.007159	-2.695495	C	-8.103917	0.182197	1.217678
H	-4.596219	-4.229186	-0.961732	H	-8.841864	0.883791	-0.677378
H	-2.674071	-7.481454	-1.198356	H	-7.097207	-0.297791	3.063924
H	-3.335542	-6.305123	-0.064004	C	-9.175567	-0.864120	1.399415
H	-4.779301	-6.444398	-2.085773	H	-8.735318	-1.858432	1.544223
H	-3.404995	-6.059830	-3.119188	H	-9.793882	-0.655532	2.281515
Cu	-0.630853	-0.855117	-0.115967	H	-9.840034	-0.912125	0.530750
C	-1.589949	0.604146	0.966660	N	-3.524358	2.449150	0.882573
C	-2.386552	-0.331830	0.716598	C	-3.569931	-1.147943	0.722803
C	-0.802464	4.496485	2.395711	C	-3.758938	-2.114463	1.724198
C	-2.063584	4.170321	1.934106	C	-4.552334	-0.953016	-0.278585
C	-2.350863	2.877949	1.396012	C	-4.922846	-2.879305	1.757398
C	-1.261237	1.925862	1.405120	H	-2.989963	-2.240889	2.481139
C	0.013307	2.304546	1.861227	C	-5.712060	-1.727390	-0.216242
C	0.261192	3.576027	2.350929	C	-5.904991	-2.679515	0.786519
H	-0.635200	5.493148	2.799859	H	-5.065286	-3.616709	2.542347
H	-2.868641	4.892263	1.978668	H	-6.484253	-1.569503	-0.962755
H	0.819098	1.587012	1.833246	H	-6.819494	-3.265521	0.805321
H	1.253233	3.846061	2.700239	C	-4.336872	0.053664	-1.375623

H	-4.071824	1.028287	-0.956993
H	-3.507207	-0.254662	-2.029715
H	-5.235095	0.161175	-1.989137

complex

Li	-1.262431	-1.261857	-0.376707
Li	-0.526573	0.759806	1.335079
Li	1.031999	-1.455515	0.471650
Li	0.664665	0.567814	-1.348038
O	0.424464	-1.285896	-1.275295
O	-0.622338	-1.111041	1.360006
C	0.549901	-1.997343	-2.493638
C	-0.325193	-1.335557	-3.575536
H	-0.237041	-1.857642	-4.536014
H	-1.376036	-1.345359	-3.272604
H	-0.022049	-0.292759	-3.728809
C	0.092148	-3.449353	-2.275647
H	0.198847	-4.055484	-3.183372
H	0.685775	-3.915477	-1.479342
H	-0.961944	-3.470252	-1.974591
C	2.017538	-1.978347	-2.950175
H	2.651339	-2.501510	-2.227486
H	2.149753	-2.457796	-3.927390
H	2.373057	-0.943854	-3.027104
C	-0.877684	-1.715653	2.613990
C	0.058183	-1.115911	3.681426
H	-0.111671	-1.570661	4.664810
H	1.103568	-1.277693	3.403090
H	-0.107657	-0.036187	3.775337
C	-0.628221	-3.229475	2.502719
H	-0.838226	-3.752750	3.443419
H	-1.265475	-3.660427	1.720433
H	0.418534	-3.418442	2.236015
C	-2.338113	-1.456341	3.017241
H	-2.542323	-0.378990	3.012225
H	-3.021071	-1.933296	2.307246
H	-2.563298	-1.843085	4.018278
Br	-1.668323	1.403150	-0.814082
Br	1.854946	1.182695	0.773936
O	-1.102815	2.156530	2.601954
C	-0.306178	3.346830	2.593858
C	-2.446415	2.632344	2.394462
H	0.721506	3.075938	2.359556
H	-0.395771	3.835347	3.578705
H	-2.926496	2.794633	3.366224
H	-2.987318	1.871492	1.831241
O	1.466383	1.786218	-2.661298
C	0.816283	3.073049	-2.771561
C	1.637754	3.905038	-1.799729
C	2.858779	2.078258	-2.495049
H	-0.224461	2.940680	-2.480297
H	0.896112	3.433141	-3.804695
H	1.655679	4.970602	-2.043124
H	1.291661	3.760030	-0.770540
H	3.365402	2.037188	-3.469951
H	3.268724	1.333475	-1.807091
C	-4.277199	-1.872587	-0.973919
C	-4.116064	-3.254581	-0.345084

H	-3.493596	-3.201972	0.553522
H	-5.090891	-3.667614	-0.066350
H	-3.639771	-3.939206	-1.054863
C	-5.126725	-1.942213	-2.245807
H	-6.147323	-2.269518	-2.018208
H	-5.183582	-0.957407	-2.723431
H	-4.684547	-2.644834	-2.958953
C	-4.852735	-0.858645	0.019766
H	-4.191083	-0.747021	0.882622
H	-4.962462	0.124857	-0.451683
H	-5.839412	-1.179072	0.371393
O	-2.937969	-1.463491	-1.347466
H	-2.933421	-0.496648	-1.519202
O	2.662845	-1.812738	1.485660
H	2.746207	-0.845883	1.637937
C	3.952498	-2.317366	1.050089
C	4.873139	-2.386864	2.271096
H	5.856683	-2.785047	1.997883
H	5.018225	-1.387764	2.697777
H	4.436179	-3.030288	3.041150
C	4.525625	-1.380404	-0.018352
H	5.468409	-1.776282	-0.411536
H	3.821503	-1.263281	-0.845840
H	4.718754	-0.386622	0.399808
C	3.669617	-3.709718	0.492044
H	4.597306	-4.190513	0.165415
H	3.204753	-4.337986	1.259243
H	2.990585	-3.652357	-0.364552
O	2.962310	3.383018	-1.973484
O	-0.833010	4.142901	1.571871
C	-2.250995	3.952090	1.607532
H	-2.741205	4.795227	2.111485
H	-2.599849	3.875530	0.577417

TS4A-aR

C	1.813800	0.053590	2.741679
C	1.383086	0.706225	3.934373
H	1.002706	1.714779	4.000787
C	1.500425	-0.224941	5.009585
H	1.219343	-0.045557	6.038917
C	1.998025	-1.454074	4.478537
H	2.164298	-2.365949	5.036632
C	2.190146	-1.281545	3.075645
H	2.546288	-2.024698	2.378852
C	-1.381988	-0.954734	2.267879
C	-1.755884	-0.316820	3.498518
C	-1.597960	-1.252016	4.556077
H	-1.753441	-1.055921	5.608682
C	-1.123819	-2.470188	3.992173
H	-0.875813	-3.370385	4.540568
C	-0.972498	-2.308972	2.575914
Fe	0.204718	-0.954686	3.577899
C	-1.452082	1.662711	1.047975
C	-2.724611	2.215005	1.229474
C	-0.341599	2.501776	1.058747
C	-2.921410	3.592099	1.296639
H	-3.574302	1.549130	1.270150
C	-0.460768	3.895625	1.180810

H	0.639841	2.068756	0.916507	C	0.660129	6.149431	1.848051
C	-1.773197	4.418618	1.171229	H	1.264755	3.931712	3.320456
C	-2.877422	-0.451885	-0.140648	H	2.056531	3.000717	2.033198
C	-3.086723	0.072360	-1.422004	H	2.679377	4.585180	2.475908
C	-3.914487	-1.127236	0.491162	H	1.040365	5.284490	-0.807634
C	-4.345238	0.066047	-2.020684	H	2.565056	5.256691	0.089315
H	-2.252347	0.524852	-1.938758	H	1.782832	3.748047	-0.421221
C	-5.192959	-1.232105	-0.084647	H	1.647855	6.558073	2.089913
H	-3.729898	-1.562880	1.465900	H	0.199029	6.824371	1.128713
C	-5.410862	-0.518672	-1.283407	H	0.054749	6.163724	2.759679
H	1.838927	0.478640	1.748387	C	-6.225769	-2.119560	0.650615
P	-1.270863	-0.117209	0.669055	C	-5.530861	-3.445910	1.052027
H	-2.053325	0.718231	3.588210	C	-6.697310	-1.414016	1.941395
C	-4.337560	4.158729	1.528459	C	-7.447603	-2.533241	-0.196363
C	-4.846861	4.955503	0.307755	H	-5.123138	-3.954290	0.170657
C	-5.358145	3.030283	1.785278	H	-4.718144	-3.299239	1.766756
C	-4.324569	5.069545	2.776558	H	-6.259730	-4.114587	1.522549
H	-4.306294	5.893042	0.183212	H	-7.208328	-0.470552	1.725054
H	-4.749931	4.367034	-0.612685	H	-7.394170	-2.056136	2.493052
H	-5.907667	5.199169	0.440991	H	-5.851013	-1.188010	2.599343
H	-5.076154	2.405397	2.639836	H	-7.996747	-3.311631	0.345744
H	-6.333151	3.476304	2.008164	H	-8.143738	-1.718011	-0.386404
H	-5.483556	2.379223	0.912387	H	-7.141535	-2.944771	-1.162363
H	-5.328583	5.470888	2.960223	C	-4.531710	0.644794	-3.439828
H	-4.019593	4.502078	3.663525	C	-5.450490	1.887455	-3.435740
H	-3.635060	5.904999	2.645546	C	-3.186557	1.087785	-4.051000
C	0.847955	4.714195	1.312346	C	-5.133706	-0.439009	-4.363354
C	1.759965	4.004387	2.345220	H	-6.492618	1.625867	-3.254321
C	1.599297	4.753171	-0.034582	H	-5.128917	2.612268	-2.678292

H	-5.399571	2.381720	-4.412493	H	0.261695	-2.955608	-2.954238
H	-2.459289	0.272270	-4.097791	H	-0.416741	-1.491369	-2.264578
H	-3.360302	1.430426	-5.076197	C	-2.613928	-4.719369	-2.790905
H	-2.728574	1.917919	-3.503823	H	-0.619757	-5.259458	-2.167005
H	-5.266466	-0.033463	-5.373306	H	-1.822391	-5.356832	-0.884390
H	-4.463711	-1.302698	-4.435681	C	-2.188830	-3.917282	-4.024551
H	-6.103189	-0.780055	-3.996439	H	-1.397234	-1.945469	-4.494293
O	-1.985041	5.776597	1.030504	H	-2.619201	-1.964606	-3.224196
O	-6.688936	-0.378487	-1.791847	H	-2.891023	-5.742949	-3.071211
C	-7.462120	0.623802	-1.130827	H	-3.508608	-4.258016	-2.346571
H	-8.373824	0.747166	-1.719749	H	-3.008922	-3.868511	-4.751270
H	-7.728116	0.323448	-0.112688	H	-1.354892	-4.433073	-4.522308
H	-6.921423	1.575326	-1.087288	C	1.455586	-4.476447	-0.171753
C	-1.812816	6.239560	-0.312380	C	2.753218	-4.110981	0.573814
H	-2.462554	5.690011	-1.002614	C	1.020855	-5.904682	0.211244
H	-2.088995	7.296344	-0.308754	H	1.671169	-4.450678	-1.250657
H	-0.778400	6.131388	-0.647079	C	3.868674	-5.127275	0.298311
C	-0.614503	-3.497793	1.705790	H	2.546485	-4.092123	1.654626
H	0.178828	-4.029772	2.245293	H	3.084070	-3.110974	0.291006
C	-1.831142	-4.440505	1.618927	C	2.135813	-6.921063	-0.082170
H	-1.562283	-5.408350	1.192126	H	0.792953	-5.934417	1.285492
H	-2.235690	-4.614482	2.621613	H	0.110281	-6.197043	-0.313718
H	-2.628857	-4.003905	1.013804	C	3.434567	-6.554781	0.644000
P	0.192739	-3.111434	0.044057	H	4.765067	-4.843067	0.859978
C	-1.102852	-3.334323	-1.302389	H	4.141434	-5.069165	-0.763974
C	-0.662348	-2.523796	-2.543522	H	1.801774	-7.925719	0.205050
C	-1.496383	-4.757817	-1.736483	H	2.319359	-6.947698	-1.165963
H	-1.994186	-2.841119	-0.892668	H	4.227424	-7.269014	0.391087
C	-1.752515	-2.506392	-3.621739	H	3.274113	-6.633627	1.729639

Cu	0.652657	-0.902242	-0.265518
C	2.737086	-0.188816	-1.103079
C	1.670843	0.449679	-1.305138
C	2.479152	2.205074	-2.757766
C	2.244423	3.418558	-3.458362
C	0.979171	3.988188	-3.498695
C	-0.118247	3.396687	-2.853376
C	0.082782	2.221034	-2.141198
C	1.349238	1.632023	-2.064076
H	3.080363	3.881948	-3.966293
H	0.840366	4.913220	-4.054242
H	-1.103604	3.851989	-2.897623
H	-0.736562	1.765299	-1.601318
C	3.894362	-0.960891	-0.816584
C	4.497684	-0.871690	0.451544
C	4.459818	-1.785805	-1.822200
C	5.626617	-1.623936	0.751171
H	4.086748	-0.187230	1.182048
C	5.601462	-2.521070	-1.497155
C	6.177826	-2.452988	-0.227109
H	6.087655	-1.540247	1.730218
H	6.052915	-3.150399	-2.259336
H	7.067565	-3.037305	-0.008827
S	5.128832	2.073203	-2.804248
O	5.221325	3.486549	-3.234744
O	5.968411	1.076352	-3.496329
C	5.646095	2.030518	-1.075800
C	4.845721	2.633200	-0.100848
C	6.823726	1.386353	-0.716923
C	5.225468	2.573342	1.234949
H	3.925475	3.127273	-0.390902
C	7.199658	1.339179	0.628227
H	7.417598	0.907786	-1.487495
C	6.408902	1.921639	1.621604
H	4.596715	3.033770	1.993503
H	8.114947	0.823689	0.909592
C	6.796652	1.844768	3.078596
H	6.013136	1.359683	3.675079
H	7.722144	1.276401	3.215206
H	6.952130	2.843120	3.506573
N	3.633459	1.493279	-2.700668
C	3.871741	-1.817384	-3.202852
H	2.782165	-1.937341	-3.169653
H	4.073649	-0.860444	-3.697511
H	4.304128	-2.628345	-3.796966

INT4A-aR

C	-1.593905	0.522934	2.821621
C	-1.255766	-0.276170	3.953730
H	-1.092860	-1.344369	3.945821
C	-1.115892	0.593942	5.077578
H	-0.829296	0.299186	6.078570
C	-1.362729	1.930190	4.635485
H	-1.300358	2.821864	5.245383
C	-1.657561	1.885407	3.240157
H	-1.879831	2.725367	2.598646
C	1.687651	0.768731	2.198567
C	1.990148	0.059423	3.409747

C	2.109173	1.002012	4.466659	H	2.688140	-6.634774	0.231437
H	2.282482	0.771669	5.509646	H	3.522849	-5.295485	-0.585883
C	1.882058	2.297501	3.920379	H	4.409422	-6.368628	0.511031
H	1.874664	3.228463	4.473500	H	4.303396	-3.420987	2.647709
C	1.615249	2.178224	2.516865	H	5.241161	-4.793894	2.047486
Fe	0.230976	1.109628	3.601857	H	4.725196	-3.526172	0.926419
C	1.027868	-1.768103	0.974380	H	3.714964	-6.434684	3.013927
C	2.103110	-2.633788	1.204762	H	2.710749	-5.129614	3.673972
C	-0.260078	-2.289619	0.921260	H	1.972325	-6.395295	2.670850
C	1.932660	-4.014864	1.264592	C	-1.996258	-4.102722	1.113185
H	3.093943	-2.210798	1.285913	C	-2.717756	-3.188490	2.136059
C	-0.513431	-3.666065	1.044159	C	-2.672597	-3.912147	-0.260418
H	-1.084784	-1.613613	0.731735	C	-2.230403	-5.543551	1.614283
C	0.613528	-4.513791	1.092942	H	-2.269925	-3.286972	3.131543
C	2.956093	-0.123736	-0.192995	H	-2.697228	-2.134355	1.853061
C	3.038941	-0.798722	-1.416454	H	-3.770428	-3.477108	2.204257
C	4.122850	0.368776	0.379624	H	-2.187475	-4.501074	-1.044164
C	4.260450	-1.104262	-2.011503	H	-3.722345	-4.214761	-0.205563
H	2.122083	-1.117050	-1.890675	H	-2.641592	-2.868488	-0.581173
C	5.388530	0.146293	-0.193877	H	-3.300687	-5.664069	1.820978
H	4.050350	0.920073	1.309524	H	-1.957433	-6.308185	0.887886
C	5.435640	-0.691301	-1.327673	H	-1.681936	-5.745126	2.539071
H	-1.742268	0.179898	1.807505	C	6.596006	0.845836	0.475471
P	1.304186	0.005220	0.602038	C	6.212508	2.321566	0.753841
H	2.055357	-1.016018	3.492688	C	6.906828	0.167504	1.828251
C	3.142897	-4.931490	1.541056	C	7.877295	0.906181	-0.382908
C	3.449235	-5.864310	0.348921	H	5.911240	2.827884	-0.170532
C	4.421706	-4.109290	1.803798	H	5.398205	2.416508	1.474884
C	2.862386	-5.777565	2.802819	H	7.075399	2.854532	1.167823

H	7.196370	-0.880455	1.699434	C	1.479090	3.415358	1.649297
H	7.730942	0.685579	2.333235	H	0.811157	4.092560	2.195351
H	6.034673	0.190702	2.490823	C	2.857284	4.098735	1.547912
H	8.586626	1.586448	0.102701	H	2.784360	5.093969	1.107205
H	8.375183	-0.056026	-0.491836	H	3.298083	4.202182	2.545229
H	7.668599	1.292086	-1.384615	H	3.546443	3.504784	0.944084
C	4.300001	-1.811446	-3.383619	P	0.591237	3.185046	-0.003630
C	4.975498	-3.198588	-3.304160	C	1.925744	3.069596	-1.328918
C	2.879612	-2.036561	-3.938928	C	1.357143	2.298774	-2.541251
C	5.065061	-0.924341	-4.392169	C	2.603998	4.362348	-1.814916
H	6.051224	-3.117689	-3.152756	H	2.695003	2.432450	-0.873015
H	4.547685	-3.801039	-2.493658	C	2.444962	2.005127	-3.581517
H	4.813854	-3.740320	-4.243500	H	0.550391	2.886608	-3.001853
H	2.315307	-1.104260	-4.038624	H	0.897661	1.359943	-2.210937
H	2.953376	-2.485058	-4.934962	C	3.705782	4.055320	-2.840808
H	2.299447	-2.721952	-3.311338	H	1.851005	5.012895	-2.280242
H	5.105965	-1.421137	-5.369038	H	3.035839	4.919864	-0.981797
H	4.557323	0.037008	-4.525020	C	3.156014	3.281179	-4.042916
H	6.086675	-0.736708	-4.056597	H	2.005264	1.476340	-4.436328
O	0.464212	-5.884416	0.962307	H	3.180628	1.326588	-3.132311
O	6.650200	-1.137343	-1.821253	H	4.178433	4.990260	-3.166795
C	7.196479	-2.227915	-1.079985	H	4.490186	3.457393	-2.352696
H	8.067025	-2.577229	-1.640281	H	3.964831	3.035676	-4.742415
H	7.511705	-1.918253	-0.078689	H	2.446918	3.919437	-4.590113
H	6.471341	-3.043341	-0.982796	C	-0.293667	4.822115	-0.241141
C	0.238657	-6.297714	-0.386392	C	-1.646063	4.769327	0.498844
H	1.034807	-5.936421	-1.046975	C	0.459179	6.113995	0.131679
H	0.236998	-7.390323	-0.377212	H	-0.505491	4.841470	-1.321294
H	-0.720920	-5.934731	-0.764982	C	-2.487203	6.025672	0.238089

H	-1.455628	4.687450	1.580114	H	-3.809418	0.945801	1.344417
H	-2.203323	3.875549	0.205901	C	-5.080904	4.017504	-0.599668
C	-0.386951	7.361601	-0.164689	C	-5.270304	3.953613	0.782468
H	0.689527	6.099774	1.205659	H	-4.914187	2.796643	2.572484
H	1.411247	6.184165	-0.396772	H	-5.428835	4.889215	-1.148510
C	-1.726167	7.310536	0.575902	H	-5.775498	4.766108	1.298031
H	-3.416520	5.963959	0.811658	S	-5.699201	-0.380784	-2.266824
H	-2.779109	6.039995	-0.821453	O	-5.998404	-0.978416	-3.569159
H	0.175610	8.262487	0.111218	O	-6.365823	0.843180	-1.835137
H	-0.570073	7.423471	-1.247368	C	-5.953069	-1.623689	-1.011945
H	-2.333651	8.190841	0.331832	C	-6.158403	-2.949604	-1.388886
H	-1.538614	7.348213	1.659615	C	-5.969105	-1.239602	0.330335
Cu	-0.509034	1.204137	-0.266999	C	-6.384835	-3.904081	-0.398991
C	-3.328805	0.727567	-1.255134	H	-6.162189	-3.216135	-2.439023
C	-2.014715	0.345429	-1.145733	C	-6.184698	-2.210243	1.303936
C	-3.101892	-1.050012	-2.707216	H	-5.837842	-0.197599	0.599272
C	-3.258377	-2.099911	-3.614268	C	-6.394928	-3.553147	0.956959
C	-2.144664	-2.900544	-3.872301	H	-6.558212	-4.938335	-0.684528
C	-0.908347	-2.653543	-3.253994	H	-6.202018	-1.919622	2.351055
C	-0.767340	-1.598397	-2.358747	C	-6.600541	-4.600917	2.022016
C	-1.870010	-0.786962	-2.058161	H	-7.102834	-4.185641	2.901580
H	-4.202348	-2.274970	-4.112608	H	-7.197740	-5.438881	1.648960
H	-2.239515	-3.723041	-4.575959	H	-5.638396	-5.009585	2.359027
H	-0.057467	-3.291047	-3.478840	N	-4.019442	-0.077374	-2.245696
H	0.176511	-1.411309	-1.861905	C	-4.228320	3.099224	-2.787345
C	-4.003042	1.857007	-0.584051	H	-3.248016	2.701781	-3.071487
C	-4.169685	1.814142	0.805566	H	-4.983987	2.520847	-3.329240
C	-4.441244	2.992692	-1.300176	H	-4.296388	4.140702	-3.118750
C	-4.794723	2.854449	1.494082				

TS4A-aS

C	1.101983	-0.283652	3.602124
C	0.315472	0.269833	4.656162
H	-0.126517	1.255495	4.663669
C	0.174243	-0.729059	5.665662
H	-0.395984	-0.631722	6.579851
C	0.868310	-1.899459	5.232095
H	0.917657	-2.841237	5.762326
C	1.441530	-1.623252	3.955068
H	2.011521	-2.309109	3.346810
C	-1.783897	-1.275196	2.199535
C	-2.522966	-0.838588	3.350520
C	-2.583268	-1.911340	4.279804
H	-3.030136	-1.876943	5.264616
C	-1.882542	-3.014565	3.715196
H	-1.722361	-3.974813	4.189071
C	-1.374487	-2.644505	2.426395
Fe	-0.616270	-1.401281	3.879836
C	-1.639471	1.496082	1.370852
C	-2.910258	2.073348	1.290212
C	-0.554918	2.296887	1.715997
C	-3.097610	3.448317	1.426085
H	-3.752307	1.435095	1.062309
C	-0.672042	3.682081	1.899634
H	0.428051	1.846024	1.758042
C	-1.939776	4.246323	1.632742
C	-2.711785	-0.456981	-0.385289
C	-2.661035	0.219738	-1.611711
C	-3.809724	-1.259633	-0.096566
C	-3.749629	0.224598	-2.484814
H	-1.767304	0.775295	-1.864191
C	-4.916999	-1.342438	-0.956898
H	-3.811061	-1.821567	0.829543
C	-4.911392	-0.486617	-2.078640
H	1.375956	0.209043	2.680839
P	-1.344760	-0.215396	0.804401
H	-2.910188	0.160740	3.489204
C	-4.522765	4.038448	1.378194
C	-4.734844	4.960963	0.157874
C	-5.587030	2.925442	1.284646
C	-4.785744	4.830218	2.678778
H	-4.194830	5.901316	0.261886
H	-4.414939	4.468459	-0.768208
H	-5.799918	5.201446	0.058768
H	-5.515389	2.213957	2.114609
H	-6.581820	3.381420	1.323822
H	-5.516934	2.365319	0.344575
H	-5.799336	5.248898	2.665323
H	-4.702586	4.174185	3.553060
H	-4.074012	5.650049	2.790739
C	0.607364	4.437739	2.336635
C	1.232898	3.679736	3.535551
C	1.637013	4.443471	1.183536
C	0.379001	5.880300	2.834929
H	0.535408	3.638580	4.380115
H	1.528468	2.658132	3.287169
H	2.135147	4.204737	3.867301
H	1.252443	4.939613	0.288017

H	2.545462	4.967755	1.502822	H	-3.323970	-0.870916	-5.016787
H	1.920980	3.430142	0.884437	H	-5.036015	-0.421137	-4.870350
H	1.304343	6.230465	3.306409	O	-2.089134	5.617619	1.555948
H	0.140967	6.582290	2.036950	O	-6.058478	-0.320111	-2.832998
H	-0.420962	5.928533	3.578989	C	-7.005465	0.563511	-2.233353
C	-6.015815	-2.369302	-0.593357	H	-7.792411	0.720826	-2.974474
C	-5.324373	-3.713321	-0.248046	H	-7.442765	0.132545	-1.326831
C	-6.789406	-1.890162	0.654827	H	-6.543578	1.524004	-1.978833
C	-7.010086	-2.690566	-1.728210	C	-1.636710	6.167995	0.315062
H	-4.721061	-4.068715	-1.091457	H	-2.210238	5.762546	-0.525171
H	-4.675936	-3.641628	0.628422	H	-1.796362	7.246287	0.382602
H	-6.082918	-4.472786	-0.029138	H	-0.577339	5.961636	0.143454
H	-7.304784	-0.941722	0.471936	C	-0.706417	-3.683998	1.543617
H	-7.542532	-2.632925	0.943440	H	0.012838	-4.204846	2.187192
H	-6.115421	-1.742669	1.505836	C	-1.763257	-4.718827	1.106577
H	-7.605685	-3.561390	-1.430467	H	-1.302135	-5.597262	0.651688
H	-7.705505	-1.879305	-1.939163	H	-2.336974	-5.053244	1.976987
H	-6.489898	-2.936872	-2.657943	H	-2.470606	-4.289022	0.393443
C	-3.657157	0.948367	-3.846131	P	0.377930	-3.045757	0.133376
C	-4.595237	2.175079	-3.897582	C	-0.610063	-3.224488	-1.461148
C	-2.228724	1.459898	-4.113704	C	-0.059228	-2.233296	-2.510656
C	-4.024458	-0.029422	-4.986333	C	-0.739172	-4.619809	-2.098180
H	-5.644665	1.885237	-3.953073	H	-1.613866	-2.875894	-1.182280
H	-4.450239	2.817521	-3.020678	C	-0.934469	-2.204161	-3.768822
H	-4.369171	2.771479	-4.788891	H	0.971550	-2.508595	-2.773541
H	-1.483651	0.660313	-4.066195	H	-0.003322	-1.222467	-2.094748
H	-2.188174	1.886200	-5.121360	C	-1.646681	-4.578025	-3.337235
H	-1.922389	2.243836	-3.416656	H	0.257004	-4.968328	-2.401189
H	-3.960987	0.490616	-5.949411	H	-1.131272	-5.348331	-1.387048

C	-1.113626	-3.594782	-4.383366	C	0.884419	4.088784	-3.207258
H	-0.500588	-1.510845	-4.498550	C	-0.042135	3.714386	-2.223309
H	-1.918124	-1.802767	-3.493683	C	0.192848	2.563575	-1.479484
H	-1.735800	-5.585664	-3.761481	C	1.342285	1.793385	-1.685531
H	-2.658142	-4.272319	-3.030357	H	2.720351	3.610864	-4.230394
H	-1.790382	-3.546263	-5.245112	H	0.710969	4.984668	-3.799216
H	-0.146273	-3.957390	-4.759823	H	-0.936078	4.305060	-2.046324
C	1.801287	-4.258368	0.086138	H	-0.503737	2.272598	-0.707956
C	2.817184	-3.874579	1.180196	C	3.892852	-0.940875	-0.881551
C	1.473216	-5.760613	0.193755	C	4.340220	-1.658127	-2.007561
H	2.273337	-4.063018	-0.887977	C	4.644094	-0.963527	0.320997
C	4.089026	-4.726708	1.098672	C	5.505433	-2.412993	-1.942541
H	2.348792	-4.026400	2.164303	H	3.795857	-1.547060	-2.937003
H	3.072049	-2.815855	1.098483	C	5.800154	-1.743861	0.363593
C	2.747865	-6.614927	0.105284	C	6.233586	-2.462490	-0.751966
H	0.985486	-5.953606	1.159465	H	5.859122	-2.934478	-2.826211
H	0.772818	-6.071815	-0.582511	H	6.384379	-1.765469	1.279896
C	3.768437	-6.222449	1.178895	H	7.148871	-3.045226	-0.695428
H	4.779571	-4.432865	1.898185	S	4.831003	1.788255	-3.248513
H	4.599261	-4.505722	0.153522	O	4.903693	3.025795	-4.056162
H	2.484157	-7.676169	0.192846	O	5.596372	0.611804	-3.696889
H	3.197757	-6.483007	-0.889152	C	5.458341	2.216754	-1.615319
H	4.683489	-6.817016	1.070508	C	4.914783	3.309347	-0.930176
H	3.355439	-6.456151	2.171678	C	6.422812	1.416083	-1.011912
Cu	0.726437	-0.785235	0.079581	C	5.330792	3.576368	0.370383
C	2.740592	-0.116536	-1.001488	H	4.174488	3.938185	-1.413277
C	1.717328	0.621881	-0.927495	C	6.841202	1.705065	0.288241
C	2.288429	2.148658	-2.714718	H	6.822551	0.571056	-1.560446
C	2.015916	3.325326	-3.459705	C	6.295456	2.775505	1.002874

H	4.906658	4.422989	0.905401
H	7.593515	1.076377	0.759067
C	6.701791	3.046510	2.431021
H	5.958269	2.650644	3.136680
H	7.661784	2.577159	2.669780
H	6.791785	4.120828	2.627585
N	3.331806	1.289907	-2.906006
C	4.215051	-0.151841	1.511103
H	4.015274	0.884648	1.221875
H	3.296361	-0.550623	1.955622
H	4.988579	-0.151785	2.284181

INT4A-aS

C	1.476244	0.781315	3.160759
C	0.635743	1.487752	4.070810
H	0.052662	2.366835	3.837564
C	0.663304	0.799181	5.321341
H	0.101705	1.065248	6.207282
C	1.511327	-0.341022	5.177708
H	1.708405	-1.085519	5.937939
C	2.009510	-0.351442	3.841207
H	2.669250	-1.086881	3.408727
C	-1.382017	-0.872099	2.327018
C	-2.081096	-0.301888	3.442618
C	-1.877504	-1.137133	4.574687
H	-2.232520	-0.947077	5.579092
C	-1.053451	-2.226311	4.169551
H	-0.692619	-3.023505	4.807568
C	-0.730223	-2.081752	2.781408
Fe	-0.068591	-0.442449	3.848338
C	-1.784878	1.589473	0.851006
C	-3.143046	1.909972	0.961720
C	-0.848230	2.614795	0.784249
C	-3.592381	3.226321	0.892616
H	-3.855207	1.102650	1.057581
C	-1.227554	3.967889	0.772520
H	0.199691	2.350040	0.700061
C	-2.611137	4.237925	0.708536
C	-2.696007	-0.836172	-0.193139
C	-2.989372	-0.346933	-1.471590
C	-3.544189	-1.783762	0.369341
C	-4.152617	-0.701043	-2.151054
H	-2.308661	0.363337	-1.917758
C	-4.724633	-2.207726	-0.269706
H	-3.302058	-2.180258	1.347221
C	-5.055932	-1.571355	-1.483321
H	1.671182	1.040121	2.128444
P	-1.230620	-0.145619	0.679492
H	-2.617033	0.636291	3.419265
C	-5.093915	3.540208	1.060013
C	-5.706340	4.164792	-0.212975
C	-5.904070	2.263332	1.365106
C	-5.278116	4.507308	2.250762
H	-5.350803	5.181617	-0.375302
H	-5.471192	3.561737	-1.098408
H	-6.797837	4.206173	-0.115746
H	-5.544734	1.753148	2.265233
H	-6.950806	2.537031	1.535238

H	-5.878445	1.549492	0.533198	H	-6.075242	-4.394695	-1.405030
H	-6.341083	4.742335	2.385529	C	-4.388981	-0.209795	-3.595189
H	-4.912391	4.049881	3.177397	C	-5.638309	0.689757	-3.718557
H	-4.734976	5.439583	2.086271	C	-3.193189	0.615347	-4.110064
C	-0.085841	5.010675	0.840500	C	-4.540003	-1.439136	-4.520813
C	0.864378	4.597653	1.994103	H	-6.561641	0.122568	-3.608151
C	0.714575	4.993985	-0.479783	H	-5.621747	1.490677	-2.969675
C	-0.526129	6.453162	1.165342	H	-5.655820	1.159373	-4.708944
H	0.326817	4.579087	2.948999	H	-2.256376	0.051058	-4.080057
H	1.322960	3.618870	1.839551	H	-3.376710	0.893182	-5.153344
H	1.678362	5.325001	2.079791	H	-3.056524	1.541916	-3.541405
H	0.092283	5.266964	-1.338474	H	-4.698602	-1.111740	-5.555468
H	1.547128	5.705177	-0.427280	H	-3.632355	-2.053368	-4.497776
H	1.128418	4.004000	-0.681801	H	-5.386858	-2.058802	-4.220114
H	0.371604	7.042938	1.384138	O	-3.061126	5.522369	0.452686
H	-1.041978	6.947975	0.343077	O	-6.288166	-1.784741	-2.079120
H	-1.177588	6.488427	2.043092	C	-7.342194	-1.012080	-1.505228
C	-5.547894	-3.316939	0.429344	H	-8.198303	-1.108106	-2.177388
C	-4.580144	-4.411614	0.944533	H	-7.620619	-1.382432	-0.513750
C	-6.283317	-2.722970	1.651845	H	-7.058848	0.042639	-1.420885
C	-6.553435	-4.052256	-0.482672	C	-2.953788	5.889748	-0.923491
H	-3.968995	-4.811756	0.127553	H	-3.516550	5.198358	-1.560714
H	-3.910058	-4.050724	1.727011	H	-3.376520	6.893650	-1.008039
H	-5.158199	-5.237966	1.372044	H	-1.912527	5.903152	-1.257561
H	-7.005166	-1.953892	1.359879	C	-0.001353	-3.180006	2.032039
H	-6.827532	-3.509834	2.187807	H	0.783684	-3.533036	2.711564
H	-5.574904	-2.263234	2.349751	C	-0.965484	-4.356780	1.787563
H	-6.927665	-4.932117	0.053190	H	-0.433367	-5.248538	1.449489
H	-7.416412	-3.449083	-0.759299	H	-1.491703	-4.607201	2.715200

H	-1.718536	-4.102175	1.039540	C	4.649011	-5.412611	1.748803
P	0.964154	-2.635444	0.502357	H	5.445426	-3.501845	2.413272
C	-0.041264	-3.128495	-1.013475	H	5.288656	-3.649555	0.665969
C	0.405289	-2.244450	-2.199181	H	3.527109	-7.041593	0.847081
C	-0.115039	-4.591822	-1.483912	H	4.102005	-5.836297	-0.300465
H	-1.059551	-2.816638	-0.746113	H	5.625211	-5.903917	1.656223
C	-0.544255	-2.381051	-3.394086	H	4.270656	-5.650492	2.753824
H	1.425753	-2.520603	-2.496515	Cu	1.041135	-0.369320	0.159488
H	0.461232	-1.194984	-1.894617	C	3.231842	1.661994	-0.507766
C	-1.102724	-4.726419	-2.655066	C	2.014128	1.069242	-0.724754
H	0.878373	-4.915662	-1.822066	C	2.498326	2.547472	-2.497042
H	-0.413889	-5.262425	-0.675852	C	2.299641	3.286246	-3.661113
C	-0.695501	-3.839459	-3.837646	C	1.091445	3.106754	-4.337661
H	-0.188062	-1.754733	-4.221535	C	0.112908	2.225731	-3.849917
H	-1.527755	-1.992211	-3.100497	C	0.324245	1.506068	-2.675418
H	-1.167501	-5.776306	-2.966879	C	1.532570	1.653247	-1.981203
H	-2.105654	-4.434518	-2.309949	H	3.060049	3.963678	-4.027178
H	-1.431916	-3.919999	-4.646963	H	0.904002	3.668261	-5.248539
H	0.261309	-4.199644	-4.244367	H	-0.821739	2.113363	-4.391000
C	2.487821	-3.726150	0.570270	H	-0.436329	0.836749	-2.291591
C	3.433036	-3.194389	1.664236	C	4.112012	1.522433	0.663860
C	2.313180	-5.244991	0.757734	C	4.413233	0.225383	1.100575
H	2.966738	-3.542418	-0.402795	C	4.605503	2.627846	1.392836
C	4.797409	-3.892228	1.618812	C	5.173702	-0.002868	2.243398
H	2.976732	-3.370966	2.649353	H	4.041480	-0.594662	0.499844
H	3.549028	-2.116532	1.565683	C	5.353470	2.380195	2.549234
C	3.671794	-5.963572	0.703855	C	5.641526	1.085321	2.979603
H	1.850585	-5.434192	1.736332	H	5.402691	-1.018301	2.555541
H	1.646682	-5.668372	0.005965	H	5.721519	3.228376	3.121457

H	6.230225	0.930207	3.879610
S	5.139170	2.212189	-2.322886
O	6.165944	2.411725	-1.302780
O	5.196752	2.939256	-3.591818
C	4.980112	0.457441	-2.638223
C	5.645690	-0.442540	-1.809847
C	4.087203	0.015488	-3.619390
C	5.404087	-1.807629	-1.962723
H	6.318596	-0.070124	-1.046998
C	3.850467	-1.349644	-3.746974
H	3.580365	0.727874	-4.259083
C	4.498201	-2.280276	-2.919434
H	5.925012	-2.515158	-1.322969
H	3.148713	-1.699204	-4.500109
C	4.232788	-3.757847	-3.076715
H	4.587216	-4.124037	-4.048160
H	4.735528	-4.339493	-2.297824
H	3.159641	-3.978761	-3.023299
N	3.601065	2.568003	-1.598074
C	4.366899	4.049349	0.953294
H	5.077563	4.326983	0.167489
H	3.366099	4.184168	0.539330
H	4.495403	4.740499	1.792986

I

C	-0.063212	0.020054	0.266788
C	-1.431907	-0.679891	0.167531
C	-2.316247	-0.674874	1.250491
C	-1.835742	-1.269633	-1.036180
C	-3.578329	-1.257602	1.133219
H	-2.005486	-0.218534	2.182516
C	-3.100571	-1.844821	-1.154718
H	-1.159766	-1.278022	-1.884653
C	-3.976117	-1.843976	-0.068324
H	-4.251397	-1.251988	1.986055
H	-3.397643	-2.297855	-2.096439
H	-4.958965	-2.298123	-0.157496
C	0.967463	-0.732756	-0.601351
C	1.179555	-2.097071	-0.340273
C	1.682995	-0.131884	-1.638114
C	2.099865	-2.831384	-1.079872
H	0.605112	-2.583598	0.442684
C	2.604929	-0.871038	-2.386628
H	1.524200	0.913964	-1.871126
C	2.820659	-2.217671	-2.108583
H	2.250562	-3.884301	-0.859436
H	3.151536	-0.384947	-3.189788
H	3.538263	-2.789607	-2.689710
C	-0.183677	1.508759	-0.137494
C	0.861585	2.394927	0.153402
C	-1.299277	1.997290	-0.823626
C	0.802909	3.728860	-0.241257
H	1.738202	2.043400	0.686716
C	-1.362939	3.335933	-1.215850
H	-2.124411	1.335558	-1.055034
C	-0.314651	4.206980	-0.927650
H	1.627511	4.395356	-0.005221
H	-2.240595	3.694363	-1.746292

H	-0.367373	5.249014	-1.229615
N	0.291160	0.027375	1.706714
S	1.732254	-0.249552	2.187394
O	1.871538	-0.160586	3.659060

TS5A-aR-R

C	-0.622791	-3.940295	2.012324
C	0.208565	-5.097079	1.978802
H	0.616407	-5.556983	1.088698
C	0.467601	-5.491483	3.327530
H	1.097610	-6.313504	3.641663
C	-0.204854	-4.573362	4.192104
H	-0.173671	-4.584165	5.273858
C	-0.877937	-3.614637	3.376258
H	-1.457256	-2.767291	3.714767
C	2.152653	-2.044340	2.065836
C	2.950033	-3.240675	2.061674
C	3.180835	-3.638250	3.404476
H	3.702449	-4.529700	3.727201
C	2.529142	-2.696840	4.250710
H	2.481541	-2.752364	5.330092
C	1.888674	-1.694492	3.446681
Fe	1.156435	-3.575676	2.999028
C	1.815038	-2.338464	-0.780956
C	3.005350	-2.807648	-1.340839
C	0.601416	-2.819058	-1.262734
C	3.005151	-3.697149	-2.416331
H	3.940382	-2.450042	-0.934103
C	0.524020	-3.750104	-2.308897
H	-0.308745	-2.439815	-0.816607
C	1.743059	-4.101256	-2.930864
C	3.378433	-0.215193	0.358203
C	3.672665	0.471253	-0.826851
C	4.318115	-0.182293	1.385935
C	4.902345	1.096741	-1.036136
H	2.933484	0.497176	-1.610698
C	5.570339	0.440762	1.252293
H	4.081618	-0.677389	2.316117
C	5.876093	0.988667	-0.009052
H	-0.969519	-3.361512	1.175268
P	1.764892	-1.097068	0.561904
H	3.272108	-3.760238	1.171113
C	4.340931	-4.267536	-2.940798
C	4.601333	-3.908484	-4.420174
C	5.539253	-3.726754	-2.133658
C	4.325285	-5.804518	-2.778563
H	3.945822	-4.461249	-5.091675
H	4.465226	-2.834811	-4.596192
H	5.634585	-4.161748	-4.685813
H	5.461704	-3.967760	-1.068044
H	6.458504	-4.187960	-2.509905
H	5.647408	-2.639965	-2.232743
H	5.269385	-6.231839	-3.138563
H	4.210721	-6.078410	-1.723245
H	3.504604	-6.249902	-3.343713
C	-0.886224	-4.287688	-2.661689
C	-1.639248	-4.607734	-1.345321
C	-1.692034	-3.201059	-3.405710

C	-0.898228	-5.600978	-3.472042	H	6.269694	1.885345	-4.163127
H	-1.074358	-5.307558	-0.721271	H	3.014742	2.405311	-2.618850
H	-1.853436	-3.717620	-0.752699	H	4.055614	2.691694	-4.017586
H	-2.604162	-5.064233	-1.585222	H	3.593742	1.045337	-3.592391
H	-1.258217	-2.962626	-4.379466	H	5.667920	4.002694	-2.773618
H	-2.724334	-3.533477	-3.558962	H	4.694464	3.824550	-1.300328
H	-1.721352	-2.269552	-2.832409	H	6.419328	3.399020	-1.283522
H	-1.924677	-5.985489	-3.488180	O	1.746255	-4.887450	-4.071008
H	-0.583761	-5.480890	-4.507784	O	7.147534	1.464116	-0.287238
H	-0.259338	-6.360970	-3.012208	C	8.087232	0.445885	-0.626956
C	6.490911	0.455255	2.496229	H	8.939595	0.950110	-1.089023
C	5.636020	0.731561	3.757888	H	8.431495	-0.096999	0.258962
C	7.144911	-0.933428	2.675617	H	7.656762	-0.269855	-1.335899
C	7.571428	1.557043	2.477318	C	1.423288	-4.178983	-5.266166
H	5.057979	1.655918	3.646980	H	2.127075	-3.357980	-5.439638
H	4.943701	-0.082053	3.983280	H	1.498050	-4.902703	-6.081413
H	6.292016	0.843662	4.627795	H	0.411100	-3.768284	-5.233056
H	7.787647	-1.197638	1.830628	C	1.178360	-0.520032	4.108159
H	7.759130	-0.949526	3.584097	H	0.351410	-0.946991	4.687113
H	6.379571	-1.712142	2.767545	C	2.134262	0.160276	5.109980
H	8.036456	1.608267	3.468511	H	1.624890	0.926219	5.694433
H	8.364141	1.381291	1.752647	H	2.542376	-0.573808	5.811460
H	7.131715	2.535203	2.258663	H	2.978365	0.626624	4.599437
C	5.142378	1.939748	-2.307140	P	0.294063	0.710480	2.977635
C	6.260665	1.360647	-3.200407	C	1.550251	2.086395	2.689269
C	3.872205	2.016839	-3.174062	C	1.365171	2.687010	1.280701
C	5.508142	3.381146	-1.883469	C	1.649425	3.223184	3.720132
H	7.246020	1.480295	-2.751902	H	2.507547	1.546025	2.675715
H	6.090000	0.296602	-3.402941	C	2.525805	3.620274	0.925092

H	0.413520	3.232740	1.233338	C	-1.309548	1.692243	-1.763711
H	1.291043	1.890276	0.536521	C	-2.429481	3.446360	-0.756693
C	2.786618	4.197369	3.366549	C	-3.146938	4.599527	-0.434343
H	0.696132	3.769127	3.740882	C	-3.713696	4.670846	0.838910
H	1.815622	2.833529	4.726586	C	-3.602718	3.606134	1.745902
C	2.650279	4.755341	1.945176	C	-2.875182	2.468706	1.410298
H	2.377301	4.020466	-0.082072	C	-2.237389	2.395423	0.167283
H	3.457631	3.037606	0.901777	H	-3.305153	5.386443	-1.159603
H	2.815809	5.012580	4.100648	H	-4.290862	5.550661	1.108352
H	3.745648	3.664738	3.450988	H	-4.107552	3.663047	2.706738
H	3.506819	5.399600	1.709445	H	-2.834559	1.615166	2.068513
H	1.753128	5.388453	1.886532	C	-0.744761	0.886095	-2.862209
C	-1.110026	1.294057	4.074948	C	0.542858	0.370129	-2.676018
C	-2.202191	0.197210	4.040964	C	-1.454564	0.552650	-4.041044
C	-0.862326	1.710576	5.536034	C	1.167511	-0.417475	-3.639921
H	-1.489915	2.172816	3.539094	H	1.035683	0.611359	-1.744626
C	-3.505882	0.671256	4.696590	C	-0.795978	-0.197867	-5.018068
H	-1.832782	-0.678428	4.594984	C	0.503167	-0.672945	-4.835853
H	-2.388567	-0.150434	3.020466	H	2.153715	-0.830861	-3.452334
C	-2.164410	2.220940	6.176804	H	-1.327846	-0.432938	-5.936700
H	-0.504095	0.846173	6.110838	H	0.981896	-1.254421	-5.617875
H	-0.095008	2.484915	5.604556	S	-1.375742	4.126699	-3.175964
C	-3.270627	1.160382	6.130103	O	-2.440503	5.130295	-3.238312
H	-4.239723	-0.143459	4.681999	O	-0.986152	3.378104	-4.365706
H	-3.934408	1.487947	4.098548	C	0.091260	4.896413	-2.509467
H	-1.971989	2.527614	7.212650	C	-0.002755	5.811455	-1.458114
H	-2.497054	3.120046	5.638017	C	1.314758	4.617686	-3.118379
H	-4.199391	1.557806	6.557868	C	1.154514	6.446368	-1.014828
H	-2.974787	0.306689	6.757657	H	-0.960104	6.029434	-1.001338

C	2.456002	5.278957	-2.672558
H	1.355161	3.908939	-3.937281
C	2.396432	6.195991	-1.614965
H	1.091324	7.156341	-0.194987
H	3.408741	5.076265	-3.150703
C	3.650738	6.865885	-1.115090
H	4.299603	7.168774	-1.943654
H	3.421797	7.751774	-0.515365
H	4.229537	6.178649	-0.485179
N	-1.880469	3.003009	-1.988195
C	-5.518515	-0.561133	-0.101948
C	-6.493808	-0.963408	1.024105
C	-6.274340	-0.483874	2.320704
C	-7.638942	-1.723510	0.770017
C	-7.174279	-0.768683	3.344950
H	-5.388531	0.107906	2.513781
C	-8.542412	-2.009926	1.796659
H	-7.831187	-2.092071	-0.231679
C	-8.312224	-1.537436	3.088088
H	-6.985729	-0.389138	4.345903
H	-9.425513	-2.605620	1.582171
H	-9.012596	-1.764650	3.887088
C	-5.558925	-1.598727	-1.247298
C	-5.273418	-2.940382	-0.938881
C	-5.860313	-1.269597	-2.570662
C	-5.281624	-3.918979	-1.927489
H	-5.054949	-3.213806	0.088825
C	-5.856758	-2.251459	-3.567959
H	-6.098826	-0.246386	-2.832734
C	-5.569159	-3.576776	-3.252557
H	-5.067235	-4.950600	-1.663784
H	-6.087055	-1.970728	-4.591837
H	-5.573011	-4.339181	-4.026473
C	-5.881453	0.844639	-0.617653
C	-4.967727	1.556727	-1.400769
C	-7.156668	1.386516	-0.419330
C	-5.321805	2.768238	-1.992031
H	-3.974148	1.160048	-1.561979
C	-7.509075	2.606881	-0.998097
H	-7.883002	0.855658	0.183479
C	-6.595973	3.299140	-1.791612
H	-4.596934	3.303511	-2.595449
H	-8.503363	3.010958	-0.827707
H	-6.868555	4.247662	-2.245602
N	-4.187867	-0.564382	0.561449
O	-1.908839	-1.459005	0.890743
Cu	-0.201686	0.018829	0.831348
S	-2.875912	-0.950670	-0.162776
C	-1.475956	1.317006	-0.445916
C	-2.907316	0.900044	-4.244716
H	-3.165949	0.868049	-5.307413
H	-3.547350	0.173960	-3.727279
H	-3.162606	1.888417	-3.867333

INT5A-aR-R

C	-1.405291	-3.753577	1.402123
C	-0.780698	-4.999547	1.095404
H	-0.344901	-5.266003	0.143775

C	-0.773883	-5.793734	2.281641	C	5.717527	0.432708	0.502967
H	-0.336108	-6.777729	2.386290	H	-1.567703	-2.930586	0.722377
C	-1.389946	-5.036130	3.324221	P	1.552372	-1.571239	0.603274
H	-1.504291	-5.347847	4.354153	H	2.642584	-4.436158	1.161033
C	-1.775580	-3.774386	2.779522	C	3.926042	-4.863559	-2.948145
H	-2.249851	-2.962896	3.313738	C	4.448355	-4.398194	-4.324940
C	1.664104	-2.643645	2.066727	C	5.085083	-4.684649	-1.946699
C	2.243565	-3.957939	2.044009	C	3.591934	-6.371592	-3.003363
C	2.128853	-4.530953	3.336900	H	3.785144	-4.703118	-5.133103
H	2.427008	-5.531621	3.620983	H	4.563772	-3.308309	-4.354189
C	1.480641	-3.580453	4.174298	H	5.431530	-4.844006	-4.517708
H	1.209205	-3.736367	5.210380	H	4.811794	-5.014342	-0.938353
C	1.175160	-2.400275	3.416150	H	5.935405	-5.292608	-2.273262
Fe	0.261081	-4.033491	2.586444	H	5.424666	-3.644037	-1.888707
C	1.557312	-2.669900	-0.860063	H	4.475129	-6.941376	-3.317517
C	2.702245	-3.317218	-1.335319	H	3.290177	-6.734061	-2.013647
C	0.347538	-2.843734	-1.531046	H	2.782431	-6.569933	-3.707562
C	2.669645	-4.084807	-2.500782	C	-1.204500	-3.852075	-3.243247
H	3.628748	-3.195807	-0.790039	C	-2.189757	-4.070098	-2.065055
C	0.229193	-3.640880	-2.680297	C	-1.692050	-2.589339	-3.986430
H	-0.521871	-2.306843	-1.178106	C	-1.350261	-5.093041	-4.148218
C	1.431012	-4.171797	-3.198288	H	-1.901902	-4.927147	-1.449339
C	3.212384	-0.791772	0.581793	H	-2.280092	-3.192598	-1.423537
C	3.582204	0.000510	-0.511990	H	-3.188953	-4.271005	-2.466494
C	4.073705	-0.864191	1.672657	H	-1.116142	-2.381588	-4.889980
C	4.818918	0.639749	-0.577502	H	-2.740808	-2.720919	-4.280600
H	2.888749	0.105809	-1.332619	H	-1.627758	-1.705831	-3.345606
C	5.327546	-0.228969	1.685447	H	-2.417447	-5.269784	-4.324254
H	3.764612	-1.441297	2.534065	H	-0.873917	-4.986274	-5.122115

H	-0.935511	-5.985485	-3.668654	C	7.959616	-0.068387	-0.005545
C	6.147422	-0.313689	2.996019	H	8.876898	0.464729	-0.267093
C	5.198876	-0.067358	4.196302	H	8.166250	-0.754720	0.822144
C	6.742662	-1.731313	3.146101	H	7.614894	-0.649818	-0.867651
C	7.266636	0.741212	3.127866	C	1.401335	-3.936270	-5.542940
H	4.685977	0.895807	4.098920	H	2.219690	-3.207895	-5.504832
H	4.441999	-0.847518	4.304373	H	1.512081	-4.562442	-6.431533
H	5.778688	-0.051366	5.125413	H	0.453386	-3.395805	-5.593930
H	7.440701	-1.967679	2.336751	C	0.519753	-1.215055	4.119361
H	7.285485	-1.817348	4.095121	H	-0.397292	-1.605175	4.577646
H	5.952584	-2.490178	3.135723	C	1.432994	-0.723831	5.260960
H	7.644794	0.720244	4.156534	H	0.932200	0.023382	5.878081
H	8.112914	0.563087	2.466517	H	1.714720	-1.559949	5.908554
H	6.889187	1.747829	2.925211	H	2.354958	-0.286567	4.871297
C	5.131869	1.605337	-1.741230	P	-0.117315	0.175279	3.020620
C	6.296093	1.109800	-2.625984	C	1.270387	1.435357	2.952510
C	3.910317	1.781258	-2.663540	C	1.203146	2.162490	1.597351
C	5.484306	2.993132	-1.155862	C	1.353606	2.475399	4.084113
H	7.254898	1.169991	-2.111334	H	2.185333	0.826455	2.957209
H	6.130747	0.075047	-2.947791	C	2.400590	3.090841	1.389665
H	6.363286	1.732207	-3.525944	H	0.281623	2.747531	1.560361
H	3.011770	2.089906	-2.122230	H	1.150164	1.437925	0.777751
H	4.122948	2.552022	-3.410848	C	2.547483	3.424492	3.883210
H	3.662440	0.864398	-3.206978	H	0.426959	3.064697	4.091300
H	5.649358	3.710631	-1.968978	H	1.434683	1.994304	5.062091
H	4.663960	3.362890	-0.531915	C	2.500985	4.120643	2.518167
H	6.385402	2.947281	-0.542298	H	2.299087	3.596658	0.424787
O	1.446529	-4.818654	-4.422762	H	3.320799	2.492035	1.345043
O	7.004631	0.925800	0.361653	H	2.568027	4.164212	4.693500

H	3.480090	2.846700	3.962103	H	-2.779128	4.276277	2.901610
H	3.384380	4.757904	2.383290	H	-3.184088	2.224613	1.516744
H	1.621500	4.778173	2.475823	C	-0.457545	1.262689	-3.111764
C	-1.584646	0.909752	3.914417	C	0.618933	0.396351	-2.905775
C	-2.855884	0.152093	3.470583	C	-1.107894	1.307720	-4.359814
C	-1.534831	1.032000	5.446443	C	1.127320	-0.376786	-3.945434
H	-1.641733	1.919997	3.487907	H	1.039547	0.333888	-1.910172
C	-4.128879	0.798882	4.028556	C	-0.572820	0.539735	-5.399182
H	-2.793679	-0.888439	3.825941	C	0.544571	-0.274181	-5.207510
H	-2.919420	0.113710	2.378955	H	1.953041	-1.058251	-3.763256
C	-2.812367	1.696058	5.986721	H	-1.046723	0.580976	-6.376429
H	-1.439196	0.031044	5.890664	H	0.937182	-0.853271	-6.038995
H	-0.660317	1.607127	5.763983	S	0.228760	4.410447	-2.937649
C	-4.073353	0.939230	5.552580	O	-0.938681	5.125738	-3.458764
H	-5.003753	0.218939	3.716327	O	1.061000	3.583477	-3.805755
H	-4.253635	1.791467	3.575556	C	1.272048	5.591961	-2.115741
H	-2.758214	1.764511	7.080681	C	0.838298	6.913581	-1.996719
H	-2.862086	2.728026	5.608563	C	2.519773	5.189972	-1.646815
H	-4.970339	1.450983	5.922270	C	1.663594	7.832493	-1.356822
H	-4.068135	-0.060385	6.012821	H	-0.133461	7.195457	-2.385473
C	-0.891176	2.098328	-1.969514	C	3.327326	6.121719	-0.999297
C	-0.912195	3.831879	-0.454990	H	2.854642	4.171460	-1.790736
C	-0.660028	4.971952	0.313072	C	2.910609	7.449257	-0.837158
C	-1.348843	5.114840	1.516432	H	1.335395	8.863359	-1.253659
C	-2.258980	4.140489	1.957239	H	4.296133	5.811210	-0.619585
C	-2.498994	2.997735	1.202387	C	3.779811	8.453995	-0.123963
C	-1.828059	2.840431	-0.018243	H	4.736036	8.015396	0.174667
H	0.061576	5.716931	0.016664	H	3.987730	9.320522	-0.762449
H	-1.165202	5.996752	2.123401	H	3.284620	8.830080	0.779433

N	-0.293169	3.358925	-1.643487
C	-5.253553	0.095905	-0.318135
C	-5.434771	-1.422697	-0.130524
C	-4.535195	-2.172941	0.630596
C	-6.550280	-2.075190	-0.678685
C	-4.736649	-3.542123	0.827234
H	-3.667674	-1.676980	1.046980
C	-6.753480	-3.440293	-0.485674
H	-7.261611	-1.505863	-1.268545
C	-5.841876	-4.181884	0.268172
H	-4.021577	-4.109532	1.412559
H	-7.621184	-3.923464	-0.927466
H	-5.990588	-5.248172	0.416601
C	-5.643394	0.472594	-1.770810
C	-5.200724	-0.340689	-2.831077
C	-6.336838	1.642758	-2.098664
C	-5.418918	0.015925	-4.159492
H	-4.686071	-1.269367	-2.613765
C	-6.560604	2.002340	-3.430320
H	-6.718309	2.282584	-1.312775
C	-6.098530	1.195858	-4.468660
H	-5.053929	-0.631031	-4.952958
H	-7.104693	2.917068	-3.650715
H	-6.270326	1.477475	-5.503964
C	-6.114801	0.846698	0.726817
C	-5.853863	2.206839	0.961720
C	-7.109535	0.230437	1.491517
C	-6.560290	2.924339	1.923606
H	-5.074689	2.691088	0.387371
C	-7.815543	0.945431	2.464989
H	-7.330343	-0.820174	1.348191
C	-7.544682	2.293189	2.688806
H	-6.334106	3.976052	2.081076
H	-8.575700	0.436836	3.052397
H	-8.091134	2.846116	3.448267
N	-3.880396	0.575743	-0.002260
O	-1.677637	-0.800135	-0.511519
Cu	-0.440236	-0.488600	0.937576
S	-2.738626	0.254639	-1.075122
C	-1.790986	1.773646	-0.994399
C	-2.348163	2.139668	-4.562481
H	-2.690531	2.078459	-5.599730
H	-3.163174	1.783232	-3.922945
H	-2.176611	3.193821	-4.322887

TS5A-aR-S

C	1.052938	3.174677	3.021232
C	0.477891	4.363112	3.553210
H	0.263362	5.267452	2.999238
C	0.175048	4.129048	4.929841
H	-0.304828	4.826092	5.604523
C	0.566482	2.790461	5.244514
H	0.434534	2.301386	6.201277
C	1.106838	2.202089	4.061794
H	1.458603	1.189508	3.937404
C	-1.993068	1.793100	2.347031
C	-2.487727	3.129353	2.537071
C	-2.854595	3.289788	3.898662

H	-3.212347	4.200611	4.360580	H	-3.450106	3.793306	-4.109443
C	-2.589127	2.060606	4.564613	H	-4.482390	5.229333	-4.236403
H	-2.719566	1.876768	5.623154	H	-4.823305	4.530868	-0.659227
C	-2.055720	1.116043	3.624853	H	-5.624572	4.987411	-2.168151
Fe	-0.852939	2.787599	3.746835	H	-4.886416	3.387627	-2.015554
C	-1.289336	2.613979	-0.302216	H	-4.367121	7.033081	-2.389043
C	-2.380342	3.272609	-0.871769	H	-3.499784	6.691113	-0.880611
C	-0.004236	3.101485	-0.529437	H	-2.589508	7.056926	-2.357382
C	-2.218771	4.365478	-1.721937	C	1.683200	4.756391	-1.440505
H	-3.371937	2.906430	-0.654500	C	2.495833	4.380484	-0.178120
C	0.229807	4.234680	-1.326223	C	2.395797	4.083629	-2.633549
H	0.828499	2.567865	-0.090682	C	1.756304	6.295244	-1.542993
C	-0.891693	4.801372	-1.980441	H	2.015030	4.759243	0.730102
C	-3.178374	0.555201	0.120665	H	2.651398	3.304635	-0.074238
C	-3.305620	0.129489	-1.207028	H	3.489346	4.836952	-0.245725
C	-4.318253	0.588552	0.916970	H	1.930467	4.310116	-3.593398
C	-4.546711	-0.136581	-1.782866	H	3.439300	4.418769	-2.678324
H	-2.416535	0.041959	-1.812143	H	2.385541	2.997504	-2.517181
C	-5.598017	0.290480	0.415550	H	2.807076	6.604350	-1.507145
H	-4.216942	0.882134	1.952875	H	1.313852	6.698224	-2.451227
C	-5.696433	0.037981	-0.967695	H	1.240819	6.758865	-0.693951
H	1.361603	2.994530	2.007105	C	-6.766770	0.286558	1.429440
P	-1.527679	1.112665	0.725936	C	-6.308556	-0.443162	2.716871
H	-2.533368	3.882631	1.764544	C	-7.124724	1.739811	1.813644
C	-3.468064	5.049652	-2.318043	C	-8.031746	-0.461049	0.957119
C	-3.526598	4.856001	-3.849073	H	-5.954838	-1.455304	2.489397
C	-4.770013	4.444543	-1.749941	H	-5.511724	0.088413	3.240924
C	-3.474732	6.553787	-1.968029	H	-7.151358	-0.525605	3.411630
H	-2.731999	5.394039	-4.363118	H	-7.476972	2.314073	0.951231

H	-7.917263	1.748062	2.571561	H	-1.026836	-0.220494	4.885717
H	-6.253373	2.260577	2.225704	C	-3.030475	-0.935621	4.666985
H	-8.712756	-0.564496	1.809757	H	-2.824468	-1.892408	5.148150
H	-8.575081	0.054061	0.167061	H	-3.461518	-0.271347	5.422207
H	-7.787084	-1.464144	0.594983	H	-3.792457	-1.094760	3.901947
C	-4.629780	-0.662048	-3.231553	P	-0.856715	-1.423506	2.838237
C	-5.361707	0.318660	-4.173645	C	-2.217782	-2.439179	2.024378
C	-3.224984	-0.886210	-3.822944	C	-1.833813	-2.788540	0.572084
C	-5.362937	-2.023407	-3.228785	C	-2.735619	-3.703822	2.731342
H	-6.432002	0.356913	-3.973363	H	-3.047436	-1.721479	1.958516
H	-4.948079	1.330535	-4.086923	C	-3.038833	-3.355163	-0.183866
H	-5.231625	-0.005864	-5.212780	H	-1.011224	-3.516437	0.567974
H	-2.624608	-1.565515	-3.212718	H	-1.456589	-1.901505	0.055191
H	-3.325036	-1.333937	-4.817877	C	-3.930994	-4.306079	1.973865
H	-2.664782	0.046834	-3.940819	H	-1.925101	-4.444266	2.779709
H	-5.420933	-2.418534	-4.250944	H	-3.036144	-3.491810	3.759062
H	-4.819274	-2.748214	-2.613847	C	-3.590314	-4.603768	0.509832
H	-6.377845	-1.925539	-2.839852	H	-2.753164	-3.580035	-1.214761
O	-0.751397	5.856024	-2.867257	H	-3.821873	-2.586176	-0.230675
O	-6.933842	-0.063628	-1.582006	H	-4.271908	-5.215438	2.484835
C	-7.539247	1.196910	-1.867816	H	-4.766857	-3.591722	2.010963
H	-8.390258	0.990150	-2.521371	H	-4.475492	-4.981000	-0.017477
H	-7.896774	1.689977	-0.958356	H	-2.834187	-5.400236	0.468968
H	-6.838887	1.866228	-2.378421	C	0.177133	-2.486872	3.982581
C	-0.270454	5.564311	-4.176227	C	1.399721	-1.645009	4.417083
H	-0.409761	4.507366	-4.424432	C	-0.451771	-3.140057	5.226672
H	-0.838046	6.179971	-4.880516	H	0.532515	-3.289466	3.325091
H	0.789406	5.820635	-4.268044	C	2.437884	-2.487647	5.169123
C	-1.753933	-0.306434	4.069805	H	1.056091	-0.835695	5.077069

H	1.857241	-1.153566	3.551864	H	-0.933724	1.943364	-3.150598
C	0.581834	-4.008957	5.962283	H	2.651828	0.950756	-5.309733
H	-0.806194	-2.359994	5.913571	H	0.635572	2.358709	-5.059079
H	-1.316187	-3.749961	4.957223	S	1.206243	-3.809074	-3.481046
C	1.811677	-3.193420	6.377551	O	2.121595	-4.936509	-3.657329
H	3.273618	-1.851125	5.484213	O	1.061390	-2.785392	-4.508440
H	2.857385	-3.238888	4.485748	C	-0.429079	-4.464919	-3.166438
H	0.116919	-4.473662	6.840754	C	-0.625899	-5.548722	-2.307712
H	0.894970	-4.829301	5.300200	C	-1.481075	-3.949441	-3.925389
H	2.550283	-3.837945	6.869528	C	-1.898834	-6.104661	-2.205697
H	1.508465	-2.438779	7.118265	H	0.195504	-5.956548	-1.733417
C	1.465473	-1.677838	-1.626639	C	-2.742127	-4.528195	-3.817912
C	2.006880	-3.793533	-0.869618	H	-1.297226	-3.120853	-4.598030
C	2.380670	-5.130911	-0.714678	C	-2.972699	-5.608846	-2.956726
C	2.676625	-5.573528	0.575721	H	-2.059838	-6.943211	-1.534322
C	2.649154	-4.693343	1.668427	H	-3.561508	-4.128856	-4.407432
C	2.294611	-3.360199	1.490934	C	-4.351742	-6.206284	-2.838594
C	1.927363	-2.899308	0.222608	H	-4.692156	-6.611667	-3.798969
H	2.477924	-5.790371	-1.567349	H	-4.376520	-7.014096	-2.101890
H	2.973152	-6.608142	0.722605	H	-5.080321	-5.446174	-2.532305
H	2.937131	-5.049043	2.653971	N	1.718607	-3.036158	-2.031728
H	2.344991	-2.659444	2.310440	C	5.313041	0.509493	-0.278380
C	1.297808	-0.591103	-2.607057	C	5.617388	-0.901211	0.257772
C	0.191138	0.253677	-2.441262	C	5.205756	-2.017792	-0.485265
C	2.214092	-0.330326	-3.652829	C	6.188549	-1.113415	1.517248
C	-0.067417	1.308673	-3.309130	C	5.378025	-3.308335	0.003773
H	-0.462165	0.050088	-1.605250	H	4.742865	-1.867261	-1.452398
C	1.946457	0.743806	-4.508957	C	6.350609	-2.408550	2.016341
C	0.814142	1.542865	-4.363576	H	6.502878	-0.265736	2.114771

C	5.949916	-3.509167	1.261291
H	5.039502	-4.154300	-0.584319
H	6.792992	-2.550890	2.998785
H	6.070040	-4.516458	1.649530
C	6.085452	1.586799	0.525201
C	5.436341	2.459028	1.404073
C	7.477485	1.682320	0.390613
C	6.163370	3.404144	2.133658
H	4.360871	2.412485	1.513899
C	8.203010	2.618572	1.122957
H	7.995772	1.019288	-0.294076
C	7.547412	3.487859	1.998942
H	5.636971	4.075163	2.807228
H	9.281967	2.672980	1.005101
H	8.111874	4.223161	2.565778
C	5.715791	0.683674	-1.755028
C	5.117772	1.714672	-2.487284
C	6.715948	-0.080364	-2.363695
C	5.494538	1.967854	-3.803280
H	4.348393	2.305349	-2.011802
C	7.097432	0.171980	-3.684270
H	7.192241	-0.884853	-1.814181
C	6.486828	1.193926	-4.411016
H	5.006758	2.767058	-4.355035
H	7.869221	-0.439150	-4.144168
H	6.779603	1.384352	-5.439886
N	3.876821	0.846507	-0.175222
O	1.856922	0.988557	1.219726
Cu	0.158645	-0.408362	1.041867
C	1.542298	-1.569831	-0.244399
C	3.458314	-1.143721	-3.902377
H	4.329232	-0.491468	-3.991536
H	3.649367	-1.864336	-3.108428
H	3.358114	-1.709816	-4.833700
S	2.971967	0.032430	0.797756

INT5A-aR-S

C	1.315573	2.852232	2.397008
C	0.921353	3.928171	3.240105
H	0.543936	4.888343	2.914599
C	1.064035	3.497717	4.595170
H	0.807793	4.072856	5.475587
C	1.539598	2.150865	4.580577
H	1.728095	1.534509	5.449896
C	1.692946	1.759836	3.219484
H	2.049313	0.816794	2.847980
C	-1.862002	1.427404	2.572546
C	-2.207352	2.741486	3.023495
C	-2.112158	2.767226	4.443573
H	-2.263747	3.631193	5.077387
C	-1.699847	1.473374	4.875551
H	-1.504820	1.177065	5.898869
C	-1.524723	0.628233	3.732774
Fe	-0.282080	2.279780	3.620844
C	-2.121068	2.223923	-0.237230
C	-3.360155	2.560802	-0.791720
C	-0.991345	2.943215	-0.620830
C	-3.477662	3.527520	-1.791072

H	-4.239225	2.031294	-0.458837	C	0.602783	5.357619	-0.306187
C	-1.054216	3.985356	-1.559335	C	1.388041	3.981924	-2.259899
H	-0.032582	2.658349	-0.210023	C	0.095443	6.112244	-2.589101
C	-2.288872	4.173827	-2.216350	H	-0.200229	5.980942	0.104769
C	-3.561428	0.004860	0.857561	H	0.790840	4.544157	0.392210
C	-3.790822	-1.172104	0.140607	H	1.513996	5.963588	-0.363204
C	-4.616641	0.572591	1.567439	H	1.188664	3.650180	-3.284630
C	-5.075149	-1.710991	0.009396	H	2.312117	4.571104	-2.272813
H	-2.946437	-1.661072	-0.325086	H	1.569114	3.089668	-1.653861
C	-5.921596	0.067816	1.507684	H	1.002898	6.710691	-2.448462
H	-4.411741	1.435239	2.188784	H	0.019050	5.901495	-3.656164
C	-6.143544	-0.999583	0.611205	H	-0.763334	6.722526	-2.298465
H	1.337338	2.816101	1.321065	C	-6.959060	0.703412	2.463370
P	-1.903870	0.786956	0.876506	C	-6.330825	0.743726	3.882023
H	-2.458005	3.568369	2.372838	C	-7.266367	2.149719	2.019177
C	-4.869928	3.901757	-2.341840	C	-8.282020	-0.075238	2.611267
C	-5.005450	3.588873	-3.847396	H	-6.072909	-0.266601	4.218274
C	-5.990709	3.127975	-1.617582	H	-5.426025	1.355018	3.931458
C	-5.118220	5.408613	-2.103951	H	-7.052217	1.165394	4.590742
H	-4.397675	4.259685	-4.453193	H	-7.725672	2.176614	1.025262
H	-4.712285	2.553870	-4.061304	H	-7.959652	2.623899	2.723890
H	-6.049198	3.712817	-4.160454	H	-6.356216	2.757593	1.980573
H	-5.982458	3.307704	-0.538054	H	-8.848897	0.363030	3.440727
H	-6.960411	3.462435	-2.002126	H	-8.913983	-0.025413	1.724833
H	-5.922910	2.046283	-1.783436	H	-8.104623	-1.129394	2.840696
H	-6.106930	5.693944	-2.484067	C	-5.293535	-3.057407	-0.709247
H	-5.088251	5.637753	-1.032536	C	-6.046890	-2.881838	-2.044529
H	-4.364301	6.014914	-2.609149	C	-3.946928	-3.732644	-1.020125
C	0.226276	4.843694	-1.720459	C	-6.074498	-4.019926	0.214565

H	-7.084582	-2.586480	-1.888046	H	-1.780134	-2.888322	1.516178
H	-5.557095	-2.130539	-2.674494	C	-0.421649	-4.795534	-0.058768
H	-6.052308	-3.830633	-2.594824	H	1.109308	-3.816760	1.089289
H	-3.361588	-3.885705	-0.109323	H	0.221467	-2.729140	0.052524
H	-4.129441	-4.717939	-1.463096	C	-1.580538	-5.608044	2.035928
H	-3.339862	-3.162374	-1.729260	H	-0.035073	-4.723545	3.244156
H	-6.197637	-4.991011	-0.279727	H	-1.652823	-4.188443	3.671729
H	-5.522887	-4.184467	1.147654	C	-0.672471	-5.993603	0.862541
H	-7.061849	-3.627716	0.460180	H	0.300491	-5.055390	-0.839291
O	-2.369933	5.020918	-3.310784	H	-1.354337	-4.523248	-0.567804
O	-7.433631	-1.392521	0.301025	H	-1.720974	-6.462905	2.709008
C	-8.065445	-0.523824	-0.639533	H	-2.577516	-5.339851	1.656998
H	-9.044769	-0.958617	-0.851889	H	-1.109908	-6.826205	0.298102
H	-8.194252	0.484907	-0.233941	H	0.290648	-6.349997	1.255606
H	-7.482723	-0.456161	-1.565811	C	1.349533	-2.348610	3.656201
C	-1.820118	4.465552	-4.503813	C	2.639686	-1.515449	3.644035
H	-2.439485	3.642200	-4.878751	C	1.016103	-2.766947	5.098172
H	-1.801813	5.270541	-5.242082	H	1.577692	-3.261603	3.090005
H	-0.805259	4.091963	-4.342800	C	3.803042	-2.340659	4.209231
C	-1.300026	-0.867258	3.881232	H	2.508125	-0.618979	4.260767
H	-0.843006	-1.002301	4.865822	H	2.869162	-1.177181	2.629667
C	-2.674484	-1.569858	3.911829	C	2.168923	-3.591455	5.693774
H	-2.598297	-2.589601	4.294872	H	0.872078	-1.866445	5.711315
H	-3.347862	-1.010656	4.570218	H	0.081067	-3.335058	5.144216
H	-3.139277	-1.600907	2.924376	C	3.498776	-2.826981	5.632460
P	-0.067035	-1.628054	2.646289	H	4.723839	-1.744759	4.198054
C	-0.804145	-3.196659	1.912596	H	3.977887	-3.203903	3.551827
C	0.101817	-3.584302	0.721450	H	1.934973	-3.868091	6.729317
C	-1.002737	-4.420883	2.824189	H	2.261439	-4.530992	5.129712

H	4.314848	-3.457119	6.006402	C	-2.403577	-3.705643	-4.244790
H	3.441248	-1.956320	6.302218	H	-0.897075	-4.822877	-3.168486
C	1.630945	-1.602930	-1.926202	C	-1.591907	-1.894969	-5.615234
C	2.704338	-3.572187	-1.358848	H	0.549463	-1.606957	-5.621459
C	3.140360	-4.891103	-1.207451	C	-2.668631	-2.636552	-5.110128
C	4.073554	-5.146424	-0.203412	H	-3.227037	-4.305834	-3.871075
C	4.563161	-4.123249	0.626655	H	-1.781311	-1.068141	-6.293378
C	4.126321	-2.813788	0.469387	C	-4.084001	-2.259536	-5.464336
C	3.190602	-2.534007	-0.534753	H	-4.193426	-2.066303	-6.537074
H	2.773103	-5.680313	-1.847301	H	-4.793676	-3.042142	-5.181069
H	4.431184	-6.162894	-0.067333	H	-4.374597	-1.340270	-4.938470
H	5.301380	-4.354996	1.388682	N	1.709379	-3.008906	-2.211121
H	4.513014	-2.016431	1.092107	C	5.133551	1.346236	-0.459604
C	0.576530	-0.759164	-2.529218	C	5.717482	2.479406	-1.332478
C	-0.757708	-1.012813	-2.177898	C	4.963285	3.081754	-2.341187
C	0.884429	0.293481	-3.412091	C	7.025305	2.931713	-1.113449
C	-1.792354	-0.223589	-2.668594	C	5.501692	4.113238	-3.115222
H	-0.974895	-1.845930	-1.517816	H	3.952340	2.736298	-2.503245
C	-0.173729	1.070123	-3.901472	C	7.564198	3.960505	-1.882106
C	-1.494643	0.829750	-3.531996	H	7.626778	2.478564	-0.332177
H	-2.817591	-0.417213	-2.370163	C	6.803299	4.558185	-2.890631
H	0.053290	1.879403	-4.589667	H	4.895886	4.567921	-3.895380
H	-2.290983	1.464227	-3.907603	H	8.580574	4.296520	-1.693625
S	1.607221	-3.595412	-3.850556	H	7.223448	5.360050	-3.491962
O	1.731378	-5.049552	-3.740415	C	6.021403	0.102352	-0.711162
O	2.525916	-2.818697	-4.685106	C	5.693678	-0.744019	-1.779068
C	-0.059045	-3.222322	-4.341099	C	7.170831	-0.194195	0.033545
C	-1.102285	-4.003420	-3.847683	C	6.454359	-1.875219	-2.064349
C	-0.284681	-2.184734	-5.244511	H	4.817323	-0.514090	-2.371658

C	7.934118	-1.330249	-0.246262
H	7.468127	0.452805	0.851802
C	7.574099	-2.182460	-1.289918
H	6.159465	-2.523516	-2.885009
H	8.810718	-1.547451	0.358989
H	8.161199	-3.072391	-1.499772
C	5.106044	1.801649	1.015478
C	5.222746	0.906686	2.089134
C	4.814702	3.141460	1.312376
C	5.099881	1.343184	3.410031
H	5.430431	-0.139351	1.897128
C	4.686709	3.579867	2.628385
H	4.687595	3.845214	0.497149
C	4.840492	2.683808	3.687548
H	5.207479	0.628121	4.221836
H	4.462577	4.624231	2.827154
H	4.741357	3.024942	4.713934
N	3.761184	1.100127	-0.949365
O	1.395098	0.883572	-0.059733
Cu	0.130271	-0.189394	0.958169
C	2.506282	-1.318154	-0.913256
S	2.834159	0.215679	-0.003982
C	2.302206	0.562737	-3.837453
H	2.911322	0.866289	-2.976717
H	2.759530	-0.338251	-4.261790
H	2.338117	1.356603	-4.590430

TS5B-aR-R

C	-0.048403	0.765916	3.639466
C	-1.262046	1.192238	4.260037
H	-1.962274	1.909034	3.858472
C	-1.430097	0.442172	5.462575
H	-2.280063	0.498223	6.130558
C	-0.321789	-0.449413	5.584890
H	-0.173906	-1.182243	6.367160
C	0.528567	-0.243490	4.462283
H	1.430703	-0.782149	4.246990
C	-2.296735	-1.288781	2.053384
C	-3.254384	-1.124548	3.111957
C	-2.973055	-2.077072	4.127537
H	-3.478435	-2.165328	5.080506
C	-1.839179	-2.829279	3.708480
H	-1.360435	-3.616413	4.276886
C	-1.422727	-2.382180	2.408205
Fe	-1.370579	-0.823165	3.835978
C	-2.854761	1.376282	1.071075
C	-4.232442	1.601418	1.166719
C	-1.992688	2.453648	1.269154
C	-4.768412	2.877188	1.330610
H	-4.896262	0.756431	1.067402
C	-2.459629	3.764951	1.469308
H	-0.924521	2.279919	1.254449
C	-3.855233	3.962118	1.383518
C	-3.677577	-0.876492	-0.351451
C	-4.106649	-0.175354	-1.485392
C	-4.362294	-2.030355	0.011601
C	-5.255140	-0.537470	-2.187784
H	-3.528651	0.679646	-1.808153

C	-5.504747	-2.480553	-0.673106	H	-1.107594	6.779278	2.676064
H	-4.008010	-2.590116	0.867039	H	-2.519460	6.772312	1.626347
C	-5.990978	-1.654652	-1.706657	H	-2.596947	5.974663	3.198702
H	0.366592	1.106164	2.701906	C	-6.111628	-3.828979	-0.214793
P	-2.185316	-0.269827	0.558116	C	-4.965174	-4.843183	0.034335
H	-4.011778	-0.354206	3.143837	C	-6.860675	-3.629995	1.122011
C	-6.294142	3.056909	1.483021	C	-7.039984	-4.503048	-1.247015
C	-6.910004	3.852880	0.311170	H	-4.357382	-4.978408	-0.866350
C	-7.018495	1.694963	1.523679	H	-4.304681	-4.544648	0.851803
C	-6.589004	3.783012	2.814388	H	-5.392129	-5.814867	0.305815
H	-6.629836	4.905208	0.343570	H	-7.697677	-2.932376	1.020215
H	-6.600243	3.433941	-0.653893	H	-7.259961	-4.586104	1.481621
H	-8.004134	3.800613	0.362997	H	-6.187927	-3.230947	1.889243
H	-6.652997	1.058005	2.336514	H	-7.254502	-5.523527	-0.908843
H	-8.087449	1.865226	1.691447	H	-7.994250	-3.994979	-1.374361
H	-6.913694	1.142510	0.582488	H	-6.560555	-4.564738	-2.228445
H	-7.670847	3.912093	2.942092	C	-5.638783	0.202646	-3.486976
H	-6.215574	3.196897	3.662165	C	-7.006704	0.911788	-3.382455
H	-6.116447	4.766479	2.837993	C	-4.599713	1.282953	-3.845722
C	-1.398009	4.860314	1.738718	C	-5.668049	-0.814935	-4.650857
C	-0.369352	4.334227	2.772489	H	-7.833451	0.202584	-3.373250
C	-0.631059	5.158380	0.431201	H	-7.061723	1.531331	-2.479110
C	-1.953501	6.170445	2.336023	H	-7.146494	1.570596	-4.247678
H	-0.855239	4.078453	3.719678	H	-3.592380	0.872447	-3.956488
H	0.183403	3.463845	2.418876	H	-4.878623	1.739980	-4.801288
H	0.366346	5.121860	2.973921	H	-4.563012	2.081867	-3.096027
H	-1.298185	5.500898	-0.367189	H	-5.931046	-0.306685	-5.586584
H	0.114035	5.943337	0.609499	H	-4.682560	-1.275436	-4.782633
H	-0.106748	4.268043	0.073624	H	-6.399301	-1.604313	-4.468065

O	-4.388276	5.241390	1.347318	H	-2.701174	-1.352523	-3.168305
O	-7.211356	-1.920329	-2.308490	H	-2.655074	-5.111265	-3.416019
C	-8.333673	-1.466272	-1.554482	H	-3.357612	-3.749722	-2.538290
H	-9.202820	-1.548576	-2.211866	H	-2.865353	-3.100250	-4.896967
H	-8.498378	-2.081029	-0.663840	H	-1.170004	-3.533498	-4.672891
H	-8.207517	-0.423258	-1.243617	C	1.384694	-4.201815	-0.457633
C	-4.301768	5.857112	0.063257	C	2.176519	-3.961604	-1.757817
H	-4.704883	5.202371	-0.717516	C	2.342619	-4.677256	0.646387
H	-4.898584	6.771012	0.117578	H	0.687911	-5.023369	-0.654314
H	-3.270080	6.116365	-0.193715	C	2.893382	-5.247436	-2.199369
C	-0.451108	-3.254168	1.613503	H	2.900148	-3.156224	-1.600344
H	0.438970	-3.366693	2.241660	H	1.518402	-3.624532	-2.562750
C	-1.118139	-4.643700	1.471218	C	3.015738	-5.992860	0.226125
H	-0.474186	-5.375504	0.979283	H	3.112334	-3.919577	0.823425
H	-1.374440	-5.039868	2.457168	H	1.814647	-4.829618	1.593468
H	-2.046590	-4.561612	0.901289	C	3.780603	-5.836992	-1.094047
P	0.315350	-2.711826	-0.039397	H	3.488885	-5.045558	-3.098664
C	-1.020243	-2.816605	-1.373159	H	2.133710	-5.988913	-2.487645
C	-0.710122	-1.788342	-2.479444	H	3.695657	-6.329406	1.018100
C	-1.314081	-4.205151	-1.977036	H	2.245074	-6.770569	0.118169
H	-1.938078	-2.496005	-0.875375	H	4.185748	-6.804679	-1.414791
C	-1.773503	-1.758218	-3.584316	H	4.637620	-5.176684	-0.922417
H	0.269312	-2.004545	-2.924324	C	2.832294	0.501020	-2.642829
H	-0.628529	-0.798273	-2.034238	C	0.989633	1.514341	-3.569867
C	-2.439099	-4.110274	-3.021538	C	0.034124	2.086670	-4.411171
H	-0.422263	-4.605718	-2.473625	C	-1.157693	2.521629	-3.828744
H	-1.593135	-4.922480	-1.200129	C	-1.385473	2.373174	-2.450471
C	-2.060457	-3.149588	-4.153019	C	-0.449460	1.738820	-1.640472
H	-1.448914	-1.066661	-4.371462	C	0.746969	1.286073	-2.200822

H	0.231835	2.222712	-5.466002	H	2.663388	6.346925	0.535002
H	-1.915174	2.985413	-4.453614	H	2.683398	4.704695	1.193461
H	-2.302855	2.748501	-2.005685	N	2.310377	1.071429	-3.869979
H	-0.629094	1.597768	-0.591146	C	4.004116	-0.255317	2.128046
C	4.206882	-0.034380	-2.654386	C	3.676790	-1.081060	3.389599
C	5.159893	0.489657	-1.769717	C	2.925417	-2.251802	3.218583
C	4.594046	-1.018100	-3.590331	C	4.160354	-0.779836	4.663500
C	6.496782	0.112485	-1.845129	C	2.644897	-3.090541	4.291006
H	4.848440	1.221629	-1.035938	H	2.546193	-2.481918	2.233806
C	5.937116	-1.403878	-3.633272	C	3.871047	-1.615279	5.748375
C	6.889734	-0.832823	-2.789382	H	4.757593	0.110702	4.822083
H	7.221243	0.541055	-1.158659	C	3.112746	-2.770442	5.569787
H	6.240953	-2.158998	-4.354276	H	2.054495	-3.989542	4.133514
H	7.928527	-1.143257	-2.855985	H	4.244748	-1.356292	6.735053
S	3.305864	2.320380	-4.637833	H	2.892304	-3.418006	6.413557
O	2.496792	2.862566	-5.727987	C	4.643025	1.108145	2.448339
O	4.617256	1.736062	-4.896600	C	3.933511	1.997575	3.276308
C	3.413417	3.489662	-3.297654	C	5.814422	1.561651	1.834471
C	2.282874	4.240655	-2.961786	C	4.398293	3.290530	3.498577
C	4.529585	3.455086	-2.462506	H	2.998802	1.674284	3.723087
C	2.257840	4.906501	-1.741802	C	6.279386	2.861988	2.053850
H	1.431344	4.280019	-3.629111	H	6.363225	0.903499	1.171819
C	4.478111	4.118239	-1.238143	C	5.578008	3.728751	2.889369
H	5.400753	2.884845	-2.758221	H	3.833392	3.960095	4.140846
C	3.329466	4.816110	-0.841783	H	7.192749	3.192166	1.566145
H	1.375850	5.475214	-1.467708	H	5.940689	4.738147	3.061965
H	5.325588	4.058700	-0.561125	C	4.955116	-1.181507	1.345720
C	3.220117	5.404065	0.540357	C	4.598422	-1.779317	0.142334
H	4.204140	5.579389	0.983434	C	6.191820	-1.524837	1.917679

C	5.452837	-2.681763	-0.494113
H	3.651728	-1.522949	-0.320075
C	7.049776	-2.419921	1.286624
H	6.474881	-1.092505	2.872360
C	6.680969	-3.005578	0.071804
H	5.170395	-3.092998	-1.455165
H	8.003388	-2.665722	1.745718
H	7.349569	-3.699179	-0.429993
N	2.691421	0.027698	1.464241
Cu	1.522165	-0.823952	-0.054101
S	2.627340	1.312614	0.482533
O	1.403669	2.124600	0.800394
C	1.886460	0.563214	-1.639000
C	3.601108	-1.634130	-4.543779
H	2.633842	-1.807723	-4.061438
H	3.418630	-0.971693	-5.396486
H	3.974939	-2.589068	-4.927735

INT5B-aR-R

C	4.029640	-2.800803	1.626256
C	5.438806	-2.587593	1.564395
H	6.015212	-2.408228	0.670138
C	5.944068	-2.584105	2.899986
H	6.971787	-2.408989	3.189779
C	4.845334	-2.795665	3.788125
H	4.895144	-2.810136	4.869031
C	3.665038	-2.930191	2.998065
H	2.659741	-3.079276	3.361200
C	3.480590	0.476660	1.874243
C	4.891469	0.706477	1.718432
C	5.497368	0.713136	3.000696
H	6.554822	0.813690	3.206924
C	4.473045	0.492862	3.961925
H	4.626508	0.403027	5.028672
C	3.211264	0.344372	3.290600
Fe	4.539464	-1.091181	2.662401
C	3.519651	0.324990	-0.975819
C	3.868491	1.544132	-1.566431
C	4.031692	-0.845419	-1.534793
C	4.576004	1.622117	-2.763192
H	3.546230	2.453838	-1.088401
C	4.792992	-0.843578	-2.718943
H	3.807259	-1.791231	-1.058837
C	4.951604	0.399582	-3.374011
C	1.679865	2.019005	0.293191
C	0.920065	2.277021	-0.847246
C	1.915744	3.046397	1.198658
C	0.427119	3.546444	-1.134637
H	0.710772	1.456491	-1.512594
C	1.420266	4.346792	0.995912
H	2.505767	2.828948	2.078161
C	0.714384	4.585564	-0.209628
H	3.338966	-2.843352	0.797643
P	2.344829	0.315936	0.464098
H	5.396103	0.809087	0.769353
C	4.935814	3.005948	-3.346182
C	4.215237	3.267704	-4.687016
C	4.518702	4.144344	-2.390322

C	6.464279	3.100822	-3.546368	H	2.068328	5.545447	4.213907
H	4.612166	2.648622	-5.490671	H	2.975428	6.668329	0.769986
H	3.138267	3.082546	-4.595867	H	3.247159	6.909806	2.501888
H	4.351394	4.315355	-4.980790	H	3.857631	5.452711	1.693141
H	4.983800	4.046728	-1.403479	H	0.771181	7.061322	3.128852
H	4.842718	5.099942	-2.816648	H	0.319164	7.006907	1.418315
H	3.432162	4.191206	-2.253502	H	-0.374365	5.844616	2.538929
H	6.728619	4.081692	-3.960085	C	-0.481682	3.719564	-2.371032
H	6.984662	2.987250	-2.588290	C	-0.081724	4.908856	-3.269317
H	6.821081	2.327843	-4.228864	C	-0.452961	2.460514	-3.263455
C	5.367846	-2.192882	-3.218341	C	-1.927637	3.909834	-1.865047
C	5.887075	-3.005774	-2.007767	H	-0.356058	5.865368	-2.828760
C	4.256830	-3.025463	-3.892515	H	0.995391	4.909278	-3.475723
C	6.577300	-2.054391	-4.169090	H	-0.601571	4.829223	-4.231106
H	6.649292	-2.441929	-1.458382	H	-0.824734	1.575220	-2.744761
H	5.095611	-3.290629	-1.311255	H	-1.091792	2.632068	-4.136043
H	6.347598	-3.933454	-2.364831	H	0.557840	2.248302	-3.631933
H	3.830406	-2.525110	-4.765262	H	-2.615807	4.002387	-2.714913
H	4.654792	-3.995411	-4.214756	H	-2.232744	3.046724	-1.265613
H	3.435696	-3.202869	-3.195582	H	-2.011363	4.812140	-1.252736
H	7.034426	-3.042450	-4.296309	O	5.506351	0.472749	-4.640666
H	6.315789	-1.685631	-5.158879	O	0.208182	5.842722	-0.505978
H	7.333773	-1.382084	-3.752427	C	1.118304	6.809562	-1.026145
C	1.712824	5.412552	2.080559	H	0.594376	7.378906	-1.798386
C	1.942346	4.757732	3.463490	H	1.438895	7.503623	-0.242685
C	3.021487	6.160056	1.733505	H	1.997222	6.330946	-1.470041
C	0.539392	6.395741	2.289415	C	4.609113	0.145818	-5.701468
H	1.084629	4.147805	3.761963	H	3.642369	0.643697	-5.571643
H	2.840973	4.135490	3.500408	H	5.079721	0.498244	-6.622636

H	4.443196	-0.932571	-5.774054
C	1.942022	0.143939	4.109113
H	2.058470	-0.819473	4.619484
C	1.874630	1.231123	5.201112
H	1.063836	1.045439	5.903075
H	2.804246	1.253582	5.775904
H	1.737656	2.223198	4.765857
P	0.300401	-0.089634	3.185939
C	-0.600676	1.564845	3.279535
C	-1.593818	1.657695	2.109639
C	-1.330335	1.938230	4.584601
H	0.191584	2.299074	3.092570
C	-2.221065	3.049366	1.997987
H	-2.392447	0.917895	2.255167
H	-1.117218	1.396115	1.168879
C	-1.960037	3.339154	4.485509
H	-2.126735	1.204625	4.769091
H	-0.665359	1.914714	5.448762
C	-2.920644	3.455234	3.297351
H	-2.927567	3.051077	1.161615
H	-1.436457	3.778374	1.751640
H	-2.476444	3.573091	5.425339
H	-1.158522	4.083618	4.375773
H	-3.317975	4.476801	3.229298
H	-3.779897	2.789481	3.464319
C	-0.610553	-1.264110	4.341741
C	-0.239929	-2.704103	3.943444
C	-0.482986	-1.102134	5.867453
H	-1.660540	-1.094360	4.082050
C	-1.068956	-3.760929	4.681212
H	0.822895	-2.867309	4.170360
H	-0.344281	-2.819507	2.863956
C	-1.348147	-2.142204	6.599859
H	0.563352	-1.241723	6.170607
H	-0.778870	-0.100831	6.184851
C	-0.979682	-3.575669	6.200122
H	-0.727677	-4.763608	4.394288
H	-2.116136	-3.686310	4.360119
H	-1.243032	-2.008838	7.683760
H	-2.406325	-1.957218	6.363962
H	-1.628287	-4.294977	6.714594
H	0.048621	-3.785782	6.529857
C	-4.469217	-0.776551	-1.244938
C	-5.348142	-1.173748	0.838984
C	-6.165358	-1.409196	1.946070
C	-5.536574	-1.705470	3.154520
C	-4.138036	-1.798498	3.249981
C	-3.329088	-1.574778	2.139719
C	-3.936447	-1.226897	0.929242
H	-7.242789	-1.370658	1.861786
H	-6.146406	-1.892715	4.033436
H	-3.689725	-2.061569	4.202776
H	-2.252607	-1.675968	2.171823
C	-4.390805	-0.574944	-2.704254
C	-3.728301	0.560419	-3.188268
C	-4.854662	-1.563822	-3.597989
C	-3.540805	0.744552	-4.556505
H	-3.352214	1.290329	-2.480279

C	-4.650888	-1.360714	-4.965361	C	2.744274	-5.231205	-0.629038
C	-4.008300	-0.219030	-5.447662	H	1.890278	-4.143936	-2.277199
H	-3.024180	1.627901	-4.917732	C	2.606602	-5.607698	0.706080
H	-4.998329	-2.118269	-5.663224	H	1.373094	-5.450993	2.470465
H	-3.866304	-0.090421	-6.517390	H	3.591571	-5.582506	-1.211853
S	-7.047523	0.080128	-0.910003	H	3.349200	-6.245087	1.176719
O	-8.086776	-0.265119	0.057112	C	-0.067733	-2.537126	-2.522488
O	-7.268245	-0.052709	-2.344279	C	1.023575	-1.658927	-2.541940
C	-6.418213	1.706436	-0.556673	C	-0.864534	-2.614608	-3.671035
C	-6.332369	2.127789	0.772969	C	1.315662	-0.879804	-3.657633
C	-5.952149	2.499304	-1.604482	H	1.616361	-1.552866	-1.645975
C	-5.760046	3.365495	1.046613	C	-0.577126	-1.838377	-4.796351
H	-6.710947	1.499903	1.571096	H	-1.748668	-3.239973	-3.676898
C	-5.385373	3.736739	-1.306057	C	0.511508	-0.967347	-4.796187
H	-6.029841	2.145727	-2.625504	H	2.159547	-0.198675	-3.624233
C	-5.268754	4.182530	0.016563	H	-1.234423	-1.893019	-5.659131
H	-5.686732	3.703192	2.076015	H	0.721681	-0.350506	-5.666186
H	-5.019243	4.363200	-2.113623	C	-1.582723	-4.210374	-1.312200
C	-4.600925	5.495541	0.335765	C	-2.707000	-4.190385	-0.485214
H	-4.586846	6.159776	-0.533322	C	-1.450828	-5.263664	-2.229629
H	-5.106792	6.012268	1.158188	C	-3.699321	-5.168182	-0.597192
H	-3.560420	5.331120	0.641771	H	-2.793836	-3.409670	0.255663
N	-5.686562	-0.922815	-0.516652	C	-2.440039	-6.236803	-2.349861
C	-0.461166	-3.155249	-1.160337	H	-0.566331	-5.318784	-2.857193
C	0.690092	-3.934105	-0.508951	C	-3.576592	-6.189572	-1.537465
C	0.554799	-4.333290	0.827557	H	-4.567998	-5.122702	0.054570
C	1.791288	-4.403713	-1.230813	H	-2.322031	-7.036545	-3.076301
C	1.502026	-5.154700	1.432724	H	-4.349905	-6.946876	-1.632128
H	-0.332351	-4.028581	1.366198	N	-0.863735	-2.050953	-0.226116

O	-1.186668	0.441619	-0.597348
Cu	0.395815	-0.888699	0.941865
S	-1.748536	-0.910743	-1.065206
C	-3.414319	-0.957442	-0.388931
C	-5.526496	-2.816536	-3.099386
H	-5.628356	-3.548933	-3.905756
H	-4.954022	-3.281113	-2.289651
H	-6.526003	-2.592475	-2.712598

TS5B-aR-S

C	-0.008426	-3.740545	-0.831565
C	-1.043003	-4.699236	-0.594495
H	-1.677474	-4.751885	0.277070
C	-1.144485	-5.525733	-1.754157
H	-1.861486	-6.322514	-1.903208
C	-0.180382	-5.073781	-2.705405
H	-0.031019	-5.471594	-3.700240
C	0.516845	-3.970502	-2.135748
H	1.283215	-3.385115	-2.613704
C	-2.443341	-1.769677	-1.905066
C	-3.401859	-2.836332	-1.984231
C	-3.289160	-3.455316	-3.257292
H	-3.837298	-4.323569	-3.599233
C	-2.262650	-2.777999	-3.975161
H	-1.909057	-3.037557	-4.964671
C	-1.727324	-1.721207	-3.160882
Fe	-1.494133	-3.545996	-2.233969
C	-2.818155	-1.703966	0.974036
C	-4.160616	-1.651728	1.367323
C	-1.924291	-2.371433	1.812207
C	-4.588240	-2.071585	2.626582
H	-4.875460	-1.214104	0.688300
C	-2.287397	-2.854278	3.081148
H	-0.895970	-2.488116	1.493769
C	-3.600614	-2.567563	3.513201
C	-3.657910	0.416900	-0.668855
C	-4.002816	1.303037	0.360896
C	-4.410811	0.435104	-1.837790
C	-5.144063	2.104437	0.289910
H	-3.368220	1.342344	1.237068
C	-5.541235	1.253533	-2.000580
H	-4.123569	-0.222969	-2.646392
C	-5.949406	1.998633	-0.876855
H	0.303234	-2.937146	-0.180086
P	-2.198363	-0.700261	-0.458205
H	-4.052102	-3.134724	-1.173820
C	-6.084690	-2.005122	2.996598
C	-6.357071	-0.994528	4.132525
C	-6.939498	-1.564876	1.790417
C	-6.563838	-3.409391	3.426255
H	-5.983897	-1.353888	5.091250
H	-5.896558	-0.024038	3.913027
H	-7.437127	-0.836109	4.236027
H	-6.812492	-2.232144	0.930996
H	-7.996644	-1.588137	2.076113
H	-6.703085	-0.544202	1.469675
H	-7.629016	-3.381094	3.686449
H	-6.434186	-4.127139	2.608020

H	-6.005090	-3.766573	4.293382	C	-4.362582	3.111898	2.488652
C	-1.215396	-3.646662	3.871468	C	-5.468490	4.548617	0.784176
C	-0.693276	-4.778086	2.952129	H	-7.648311	3.090344	1.472000
C	-0.030139	-2.724658	4.235793	H	-6.869453	1.817125	2.438051
C	-1.714186	-4.351332	5.150722	H	-6.872643	3.494340	3.002092
H	-1.514051	-5.428928	2.630952	H	-3.367889	3.317510	2.085535
H	-0.189614	-4.400779	2.060039	H	-4.584830	3.882921	3.234311
H	0.026978	-5.394546	3.501208	H	-4.322535	2.148521	3.009218
H	-0.349511	-1.889093	4.867327	H	-5.674712	5.292874	1.563046
H	0.734971	-3.291207	4.779139	H	-4.495612	4.786293	0.340401
H	0.447079	-2.301165	3.346959	H	-6.235339	4.634640	0.012137
H	-0.920188	-5.020781	5.501744	O	-3.958550	-2.757663	4.837908
H	-1.935135	-3.663234	5.966073	O	-7.155752	2.680770	-0.882910
H	-2.607345	-4.952317	4.962765	C	-8.285711	1.850241	-0.623084
C	-6.221377	1.241330	-3.390984	H	-9.134354	2.521169	-0.468434
C	-5.127212	1.285466	-4.488349	H	-8.502600	1.186025	-1.465735
C	-7.012280	-0.073704	-3.571489	H	-8.132515	1.241268	0.274441
C	-7.137541	2.451282	-3.671178	C	-3.499593	-1.706907	5.688812
H	-4.488881	2.167690	-4.371494	H	-3.916523	-0.740711	5.382727
H	-4.488229	0.399256	-4.486885	H	-3.843309	-1.951334	6.696607
H	-5.601412	1.338110	-5.474490	H	-2.407255	-1.631973	5.684371
H	-7.822439	-0.167068	-2.841730	C	-0.706314	-0.765399	-3.765225
H	-7.455203	-0.116918	-4.573910	H	0.114695	-1.395563	-4.122858
H	-6.355801	-0.942546	-3.451256	C	-1.353314	-0.083707	-4.992164
H	-7.404877	2.447623	-4.734346	H	-0.649220	0.558207	-5.526894
H	-8.065664	2.437196	-3.102206	H	-1.717996	-0.837388	-5.696235
H	-6.626620	3.393665	-3.452971	H	-2.210001	0.523614	-4.690714
C	-5.451134	3.131221	1.402053	P	0.205582	0.510449	-2.704309
C	-6.796577	2.860742	2.110647	C	-1.050036	1.920298	-2.493078

C	-1.031187	3.003138	-3.592691	H	4.789952	0.756221	-4.051357
H	-2.030289	1.430473	-2.527153	C	2.144964	2.781518	0.777676
C	-1.921446	3.696038	-0.873007	C	0.405429	2.780611	2.287033
C	-2.117172	4.061531	-3.345962	C	-0.584988	3.080828	3.224137
H	-0.056567	3.508172	-3.591484	C	-1.403976	2.034099	3.651603
H	-1.163197	2.567313	-4.586382	C	-1.263892	0.736891	3.133501
C	-1.923588	4.743431	-1.989043	C	-0.295061	0.466694	2.170623
H	-1.717713	4.161423	0.099639	C	0.547274	1.491749	1.733999
H	-2.919800	3.251936	-0.806988	H	-0.693365	4.079761	3.622910
H	-2.099858	4.798047	-4.159151	H	-2.173778	2.237809	4.389535
H	-3.105745	3.581569	-3.371123	H	-1.922957	-0.058079	3.466857
H	-2.714138	5.484287	-1.815640	H	-0.191794	-0.522611	1.750329
H	-0.970699	5.290965	-1.993423	C	3.277219	3.372434	0.039871
C	1.459410	1.204293	-3.930564	C	4.526359	2.742314	0.143679
C	2.490522	2.019739	-3.124795	C	3.158321	4.561019	-0.717702
C	2.142565	0.118541	-4.775845	C	5.657650	3.282958	-0.464032
H	0.944278	1.884689	-4.615239	H	4.621255	1.853891	0.755799
C	3.621061	2.572004	-3.997587	C	4.299829	5.064083	-1.349755
H	2.931256	1.376723	-2.350241	C	5.544159	4.448941	-1.219451
H	1.994236	2.832113	-2.585341	H	6.618530	2.795903	-0.335069
C	3.300119	0.674369	-5.618605	H	4.208701	5.972672	-1.940135
H	2.516165	-0.663537	-4.113914	H	6.416628	4.880335	-1.702883
H	1.418434	-0.359765	-5.443613	S	2.307853	4.619842	2.826422
C	4.314440	1.442999	-4.766058	O	1.326268	5.142800	3.775741
H	4.332278	3.109382	-3.363272	O	3.137148	5.530034	2.043548
H	3.212406	3.302678	-4.711677	C	3.346690	3.406360	3.607220
H	3.788887	-0.150891	-6.152270	C	2.766261	2.442536	4.438033
H	2.893376	1.347721	-6.387522	C	4.699407	3.359795	3.273098
H	5.116005	1.843102	-5.399078	C	3.557392	1.396337	4.898635

H	1.718473	2.505181	4.705942	H	6.115697	-2.304697	1.667185
C	5.473838	2.310136	3.758670	C	4.153053	-4.349394	3.538993
H	5.118271	4.117509	2.622359	H	2.179012	-5.008970	2.969250
C	4.910761	1.298935	4.545463	H	6.116821	-3.505189	3.816427
H	3.111942	0.627719	5.524527	H	4.147914	-4.872626	4.491135
H	6.523644	2.252355	3.485236	C	5.402684	-1.538186	-0.676625
C	5.715817	0.089834	4.938972	C	5.453767	-0.222570	-1.135223
H	6.786675	0.312665	4.982725	C	6.545364	-2.339642	-0.814106
H	5.403754	-0.307237	5.910708	C	6.614107	0.283516	-1.724623
H	5.565246	-0.698071	4.190184	H	4.576428	0.404403	-1.050552
N	1.400629	3.613320	1.703232	C	7.714625	-1.828707	-1.372337
C	4.069923	-2.184518	-0.241744	H	6.509940	-3.379389	-0.504383
C	3.735226	-3.070939	-1.461766	C	7.751964	-0.513107	-1.839813
C	3.584588	-2.437864	-2.702590	H	6.617661	1.303534	-2.094438
C	3.762165	-4.467260	-1.432788	H	8.590084	-2.466176	-1.461569
C	3.451797	-3.170108	-3.878407	H	8.655523	-0.118143	-2.295747
H	3.619595	-1.357324	-2.734004	N	2.970974	-1.182433	-0.049288
C	3.622892	-5.208595	-2.610612	Cu	1.430193	0.049356	-0.854011
H	3.904895	-4.990277	-0.495704	C	1.615111	1.500597	0.735060
C	3.469404	-4.567603	-3.837148	S	2.959388	-0.377428	1.348083
H	3.348795	-2.653216	-4.828350	O	4.327706	-0.053812	1.856034
H	3.642449	-6.293559	-2.560698	C	-0.895650	2.583237	-1.116183
H	3.374093	-5.145411	-4.752085	H	0.114611	2.992067	-1.038101
C	4.171624	-2.999111	1.064062	H	-0.974492	1.830505	-0.332188
C	3.055744	-3.749239	1.469439	C	1.869640	5.335244	-0.839835
C	5.264688	-2.918304	1.932175	H	1.860076	6.157328	-0.116053
C	3.048604	-4.425290	2.687147	H	1.769802	5.764986	-1.843000
H	2.188300	-3.801106	0.820318	H	0.993501	4.721930	-0.635325
C	5.256827	-3.589428	3.157609				

INT5B-aR-S

C	0.552259	1.235491	3.550761
C	-0.293788	2.294025	4.006685
H	-0.717478	3.072458	3.391118
C	-0.545699	2.096921	5.396504
H	-1.184569	2.708874	6.019815
C	0.138290	0.913154	5.805437
H	0.112359	0.471307	6.793016
C	0.816652	0.384862	4.666125
H	1.399997	-0.525133	4.647181
C	-2.377736	-0.222332	2.759334
C	-3.164707	0.588911	3.648458
C	-3.130787	0.015495	4.944188
H	-3.583185	0.425733	5.837507
C	-2.322794	-1.152454	4.874335
H	-2.061037	-1.781624	5.714531
C	-1.846137	-1.331181	3.529766
Fe	-1.217707	0.476533	4.301520
C	-2.232294	2.031393	0.947611
C	-3.442818	2.725341	1.028040
C	-1.051613	2.752324	0.775494
C	-3.509567	4.109022	0.861363
H	-4.350198	2.161798	1.188167
C	-1.038286	4.149246	0.620507
H	-0.121136	2.199286	0.721856
C	-2.296267	4.793134	0.576471
C	-3.755652	-0.314598	0.293937
C	-4.263836	0.312578	-0.850462
C	-4.415367	-1.434804	0.790739

C	-5.478459	-0.070291	-1.420626
H	-3.697032	1.118832	-1.294241
C	-5.617528	-1.904770	0.236677
H	-3.988897	-1.950371	1.643471
C	-6.188187	-1.127277	-0.793300
H	0.892505	1.074899	2.536521
P	-2.143239	0.196826	1.005039
H	-3.650302	1.512844	3.371205
C	-4.858301	4.838727	1.041656
C	-5.334464	5.530778	-0.254669
C	-5.976250	3.858838	1.454823
C	-4.717459	5.887275	2.167742
H	-4.739946	6.413870	-0.485734
H	-5.293095	4.841981	-1.106834
H	-6.375102	5.855790	-0.138094
H	-5.740039	3.329959	2.384780
H	-6.899700	4.423179	1.622147
H	-6.180350	3.113261	0.677175
H	-5.669233	6.412338	2.314565
H	-4.449199	5.401793	3.113378
H	-3.948501	6.622562	1.924778
C	0.350112	4.829403	0.487543
C	1.323761	4.208023	1.520051
C	0.942957	4.556552	-0.914211
C	0.355714	6.346145	0.776376
H	0.968086	4.355636	2.544314
H	1.488524	3.142127	1.357690
H	2.296442	4.699794	1.425075
H	0.316331	4.953288	-1.718241

H	1.936278	5.012835	-0.993888	H	-5.307532	-1.018599	-4.029340
H	1.058017	3.484335	-1.092042	H	-6.985601	-1.195770	-3.467813
H	1.397661	6.673556	0.869783	O	-2.390093	6.138130	0.257762
H	-0.100398	6.946334	-0.009738	O	-7.475745	-1.389284	-1.234053
H	-0.156455	6.576739	1.715486	C	-8.481271	-0.860236	-0.372368
C	-6.171078	-3.239104	0.792104	H	-9.438338	-1.035727	-0.869632
C	-5.008085	-4.263997	0.830223	H	-8.483077	-1.360093	0.602466
C	-6.686834	-3.034135	2.233041	H	-8.341257	0.215035	-0.214409
C	-7.275316	-3.898932	-0.059778	C	-2.282118	6.417115	-1.138123
H	-4.577032	-4.402895	-0.167209	H	-3.052151	5.887014	-1.709096
H	-4.204124	-3.960043	1.504271	H	-2.426625	7.494668	-1.246479
H	-5.378324	-5.233936	1.181012	H	-1.301624	6.140004	-1.532846
H	-7.523038	-2.327779	2.262427	C	-0.977631	-2.551653	3.224726
H	-7.034206	-3.986005	2.652710	H	-0.101881	-2.439699	3.872346
H	-5.898917	-2.642041	2.885126	C	-1.711438	-3.827138	3.688356
H	-7.444344	-4.913745	0.319319	H	-1.020730	-4.666540	3.775043
H	-8.228315	-3.373184	-0.014912	H	-2.166286	-3.674536	4.669942
H	-6.980695	-3.972299	-1.110136	H	-2.513458	-4.109271	3.000110
C	-5.969736	0.600900	-2.721884	P	-0.185060	-2.724587	1.504053
C	-7.253805	1.427289	-2.492251	C	-0.987638	-4.199952	0.645982
C	-4.903869	1.556951	-3.287817	C	-0.671353	-4.127373	-0.862155
C	-6.231171	-0.479202	-3.796718	C	-0.673942	-5.631022	1.125696
H	-8.118855	0.792078	-2.300320	H	-2.064640	-4.017512	0.761097
H	-7.131562	2.121445	-1.651544	C	-1.494929	-5.142166	-1.662700
H	-7.472544	2.022430	-3.386920	H	0.393781	-4.345006	-1.008143
H	-3.950317	1.053235	-3.469130	H	-0.842506	-3.122247	-1.247094
H	-5.254077	1.957631	-4.245017	C	-1.505036	-6.665170	0.348097
H	-4.717595	2.409411	-2.625903	H	0.392883	-5.831034	0.957712
H	-6.584553	-0.002059	-4.719009	H	-0.855562	-5.759437	2.192138

C	-1.261509	-6.570944	-1.161588	C	2.654770	-2.769114	-1.321182
H	-1.250776	-5.053148	-2.727150	C	3.492814	-1.648624	-1.314011
H	-2.559460	-4.887830	-1.570797	H	6.534086	-3.199336	-1.738554
H	-1.271645	-7.673443	0.713033	H	5.038183	-5.173895	-1.699100
H	-2.571076	-6.493696	0.557501	H	2.585162	-4.910160	-1.461938
H	-1.904863	-7.279379	-1.698109	H	1.588283	-2.614337	-1.247607
H	-0.222848	-6.864100	-1.375696	C	4.652601	1.887911	-1.098931
C	1.570250	-3.254410	1.892424	C	4.920486	2.680322	-2.222296
C	2.460785	-2.008460	2.064864	C	4.557993	2.474811	0.182264
C	1.809033	-4.236029	3.053397	C	5.101726	4.054199	-2.096943
H	1.884087	-3.747838	0.963963	H	4.992631	2.199472	-3.190301
C	3.942716	-2.388868	2.155363	C	4.760779	3.856537	0.286344
H	2.166794	-1.484342	2.984224	C	5.025491	4.643050	-0.833602
H	2.296125	-1.299339	1.252716	H	5.308162	4.657872	-2.975574
C	3.295111	-4.626284	3.133834	H	4.709343	4.317260	1.269073
H	1.517135	-3.766198	4.003268	H	5.174630	5.713445	-0.719264
H	1.199388	-5.136183	2.944636	S	7.142703	-0.157332	-1.401148
C	4.196807	-3.394562	3.283809	O	7.372114	1.015867	-2.234661
H	4.545662	-1.485074	2.302186	O	7.827207	-1.419816	-1.666096
H	4.256475	-2.825398	1.198486	C	7.369148	0.282466	0.304619
H	3.451193	-5.321942	3.968106	C	7.600847	1.611555	0.647998
H	3.570558	-5.164944	2.215658	C	7.259976	-0.718257	1.274065
H	5.251713	-3.698185	3.300956	C	7.717006	1.941476	1.997138
H	3.990881	-2.914711	4.252796	H	7.667763	2.365855	-0.126840
C	4.428723	0.434538	-1.262946	C	7.373192	-0.366111	2.613818
C	4.892672	-1.813074	-1.463531	H	7.081705	-1.747359	0.981146
C	5.468448	-3.079940	-1.608971	C	7.594030	0.967417	2.995006
C	4.613569	-4.179874	-1.591761	H	7.889120	2.976714	2.277786
C	3.222178	-4.029414	-1.453356	H	7.281317	-1.133732	3.377431

C	7.657696	1.342737	4.453846
H	8.148297	0.563928	5.046831
H	8.199558	2.281424	4.603467
H	6.647307	1.475763	4.862217
N	5.461450	-0.518819	-1.455770
C	0.166300	-0.227235	-3.120328
C	-0.915356	-1.272844	-3.459308
C	-1.982527	-1.440056	-2.568787
C	-0.971833	-1.946059	-4.684548
C	-3.055595	-2.275813	-2.865026
H	-1.976744	-0.896669	-1.634219
C	-2.044860	-2.788816	-4.988888
H	-0.180525	-1.811658	-5.413072
C	-3.089042	-2.962351	-4.080924
H	-3.865780	-2.375606	-2.149628
H	-2.062286	-3.304664	-5.945298
H	-3.923699	-3.615483	-4.320197
C	1.455074	-0.382789	-3.952847
C	2.026040	-1.648985	-4.138897
C	2.192298	0.735570	-4.365164
C	3.290205	-1.797460	-4.704033
H	1.491219	-2.527038	-3.798422
C	3.456601	0.590940	-4.939547
H	1.788741	1.730380	-4.215100
C	4.017001	-0.675177	-5.104771
H	3.718368	-2.790927	-4.801564
H	4.005100	1.475202	-5.253034
H	5.008476	-0.786109	-5.534149
C	-0.552298	1.110831	-3.429217

C	-1.088790	1.877035	-2.397713
C	-0.788247	1.517598	-4.750702
C	-1.827181	3.031071	-2.661813
H	-0.935863	1.558375	-1.378899
C	-1.514194	2.676094	-5.022233
H	-0.399178	0.923428	-5.571827
C	-2.036720	3.442302	-3.976096
H	-2.240259	3.592552	-1.831922
H	-1.677817	2.977358	-6.053557
H	-2.611828	4.339932	-4.187794
O	1.337073	0.676183	0.389644
C	3.236010	-0.234844	-1.200602
S	1.670598	0.658643	-1.134287
N	0.537945	-0.404580	-1.693566
Cu	-0.333215	-0.880580	0.178841
C	4.257172	1.659094	1.414042
H	3.224583	1.297580	1.393201
H	4.906511	0.782179	1.483492
H	4.400564	2.260168	2.316848

***R*-32b-Li**

C	1.715558	0.420065	-0.292654
C	0.957290	0.868090	0.753987
C	2.568263	2.464244	0.347427
C	3.272967	3.662553	0.488379
C	2.840709	4.544248	1.476864
C	1.730598	4.257952	2.291056
C	1.028393	3.068708	2.143507
C	1.458435	2.154334	1.172159

H	4.113551	3.896780	-0.149270	H	7.310329	-3.060479	2.745811
H	3.370139	5.483456	1.607384	H	6.281089	-4.270481	1.965226
H	1.415338	4.980113	3.038370	H	5.668860	-3.325874	3.334913
H	0.159585	2.844553	2.754497	N	2.704369	1.402327	-0.581311
C	1.531380	-0.824795	-1.068424	N	-1.457605	0.056260	-0.014542
C	1.845918	-2.047367	-0.457791	C	-2.789698	0.688077	-0.097207
C	0.946002	-0.801015	-2.353328	C	-3.615313	-0.271044	-0.983939
C	1.584602	-3.255643	-1.105806	C	-2.997484	-0.829152	-2.115659
H	2.270623	-2.037072	0.538706	C	-4.955339	-0.586351	-0.740210
C	0.685317	-2.024480	-2.981126	C	-3.686815	-1.703484	-2.953435
C	0.997351	-3.241516	-2.371568	H	-1.968239	-0.567686	-2.331322
H	1.830695	-4.196054	-0.621298	C	-5.651125	-1.460298	-1.581944
H	0.224693	-2.018419	-3.965443	H	-5.459206	-0.160882	0.120136
H	0.777279	-4.174225	-2.883168	C	-5.020040	-2.029396	-2.687031
S	-0.590615	0.170469	1.372005	H	-3.182995	-2.125783	-3.819081
S	4.188761	0.981318	-1.357250	H	-6.689128	-1.697190	-1.364530
O	3.846889	0.352886	-2.628604	H	-5.560040	-2.711935	-3.337348
O	5.021551	2.179461	-1.306035	C	-3.409473	0.865976	1.302361
C	4.840376	-0.261042	-0.261853	C	-3.710969	-0.274002	2.065088
C	5.024154	-1.556080	-0.736412	C	-3.571353	2.121500	1.895036
C	5.113562	0.079842	1.065485	C	-4.164152	-0.158774	3.376036
C	5.498452	-2.529488	0.141799	H	-3.594945	-1.260766	1.631587
H	4.778329	-1.791735	-1.764952	C	-4.019737	2.238396	3.213871
C	5.573394	-0.908302	1.926424	H	-3.338110	3.016728	1.330832
H	4.957304	1.094698	1.416228	C	-4.318345	1.101056	3.960215
C	5.774623	-2.224747	1.478952	H	-4.397711	-1.057267	3.941409
H	5.644356	-3.544524	-0.216968	H	-4.133321	3.225674	3.653899
H	5.779470	-0.658804	2.963762	H	-4.668132	1.192175	4.984954
C	6.283427	-3.279718	2.428581	C	-2.723663	2.047260	-0.830986

C	-1.531703	2.772344	-0.891630
C	-3.871664	2.606609	-1.408187
C	-1.480361	4.022474	-1.511584
H	-0.635141	2.354926	-0.456522
C	-3.825698	3.853235	-2.029652
H	-4.809325	2.060849	-1.373303
C	-2.627195	4.568389	-2.085540
H	-0.538457	4.563642	-1.542732
H	-4.727866	4.265651	-2.473913
H	-2.589968	5.538699	-2.573115
O	-0.219193	-1.323938	1.653913
O	-1.715797	-3.641931	0.079131
C	-3.132110	-3.813584	0.135119
C	-1.255219	-4.203700	1.327246
H	-3.372957	-4.854151	-0.134559
H	-3.603310	-3.107711	-0.546887
C	-2.376187	-3.817669	2.305578
H	-0.297410	-3.745188	1.568943
H	-1.151305	-5.289128	1.211351
H	-2.125408	-2.906831	2.854292
H	-2.621482	-4.627060	3.001534
O	-3.506673	-3.533343	1.461090
Li	-0.995518	-1.852896	-0.043180
C	0.579387	0.499632	-3.012690
H	0.067603	0.325506	-3.963846
H	1.474273	1.097619	-3.209832
H	-0.083469	1.083079	-2.366359

$[LiO^tBu\text{-}^tBuOH]_2$

Li	-1.229925	0.497965	1.068355
Li	1.035935	-0.067379	0.753754
O	-0.418473	-1.210730	0.769578
O	0.228963	1.533471	0.992377
C	-0.443193	-2.400602	1.522161
C	-1.683333	-2.415306	2.436420
H	-1.744856	-3.331453	3.036298
H	-1.648855	-1.561498	3.126164
H	-2.598443	-2.333211	1.839335
C	0.824141	-2.485116	2.394778
H	0.850840	-3.396219	3.004522
H	1.724680	-2.475980	1.767715
H	0.869518	-1.623335	3.073656
C	-0.494978	-3.603270	0.563692
H	0.370914	-3.584894	-0.108156
H	-0.499303	-4.561830	1.097046
H	-1.399404	-3.550771	-0.054180
C	0.484539	2.902637	1.025605
C	0.517203	3.385957	2.487631
H	0.732397	4.458784	2.571728
H	-0.451322	3.190822	2.963857
H	1.282084	2.831483	3.043412
C	-0.617315	3.659788	0.257982
H	-0.463074	4.745970	0.263479
H	-0.649872	3.320603	-0.784056
H	-1.595057	3.453708	0.711985
C	1.847271	3.203126	0.364864
H	2.652885	2.712268	0.928163
H	1.846665	2.832160	-0.669698

H	2.075516	4.275104	0.333438	O	2.814657	-0.010186	-0.026957
C	-3.071799	0.143311	-1.347267	H	2.826643	0.950380	-0.182369
C	-3.902582	1.417555	-1.207557	C	3.020174	-0.688551	-1.305033
H	-3.294244	2.225589	-0.787482	C	4.185098	-0.013318	-2.028491
H	-4.283677	1.742238	-2.181605	H	4.390627	-0.514697	-2.979899
H	-4.754235	1.242570	-0.541870	H	3.951747	1.036021	-2.249569
C	-3.909095	-1.002100	-1.925408	H	5.091227	-0.046680	-1.415115
H	-4.252282	-0.768835	-2.939854	C	1.724139	-0.606456	-2.116072
H	-3.316436	-1.923327	-1.969919	H	1.829386	-1.131113	-3.072156
H	-4.783788	-1.187556	-1.294142	H	0.899475	-1.060624	-1.557121
C	-1.823443	0.392187	-2.201461	H	1.462276	0.436526	-2.328587
H	-1.182429	1.146021	-1.732667	C	3.350262	-2.132995	-0.942048
H	-1.241024	-0.528852	-2.308945	H	3.521419	-2.722045	-1.848847
H	-2.099107	0.738313	-3.203837	H	4.249395	-2.177019	-0.319704
O	-2.676346	-0.213370	-0.006554	H	2.521825	-2.587245	-0.390605
H	-1.943108	-0.897710	-0.013086				

8 Copies of ^{1}H NMR, ^{13}C NMR , ^{19}F NMR and ^{31}P NMR Spectras

8.1 *ortho*-Alkynylanilines

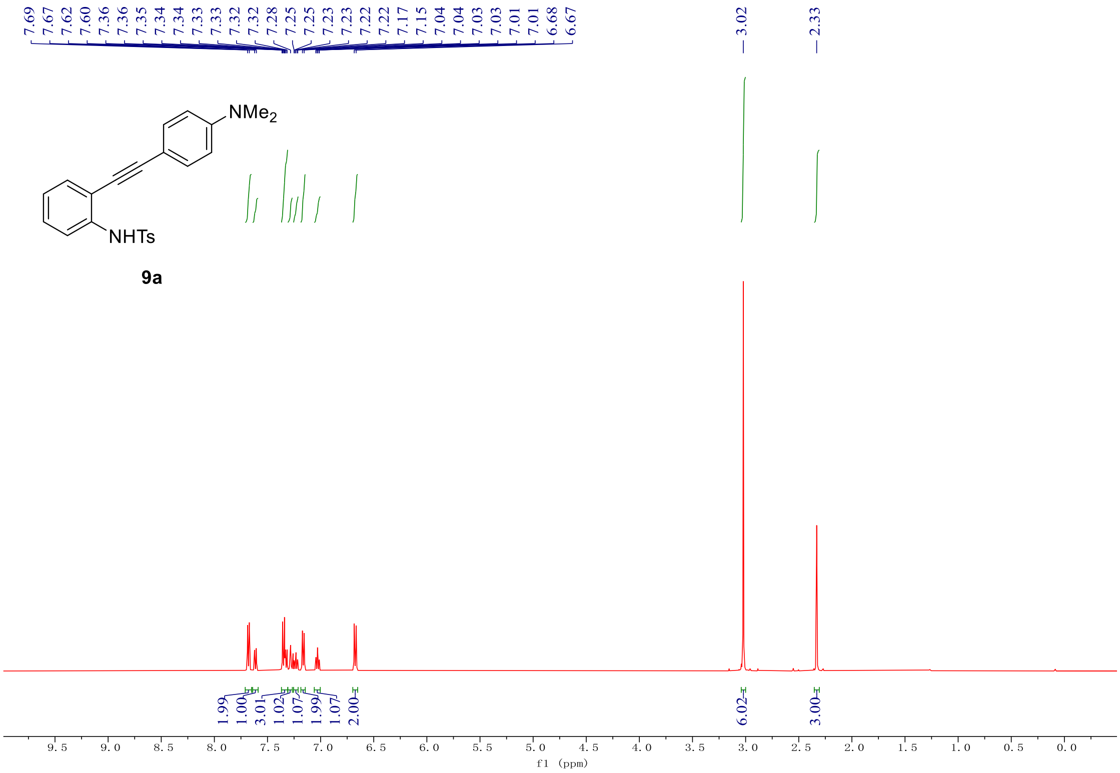

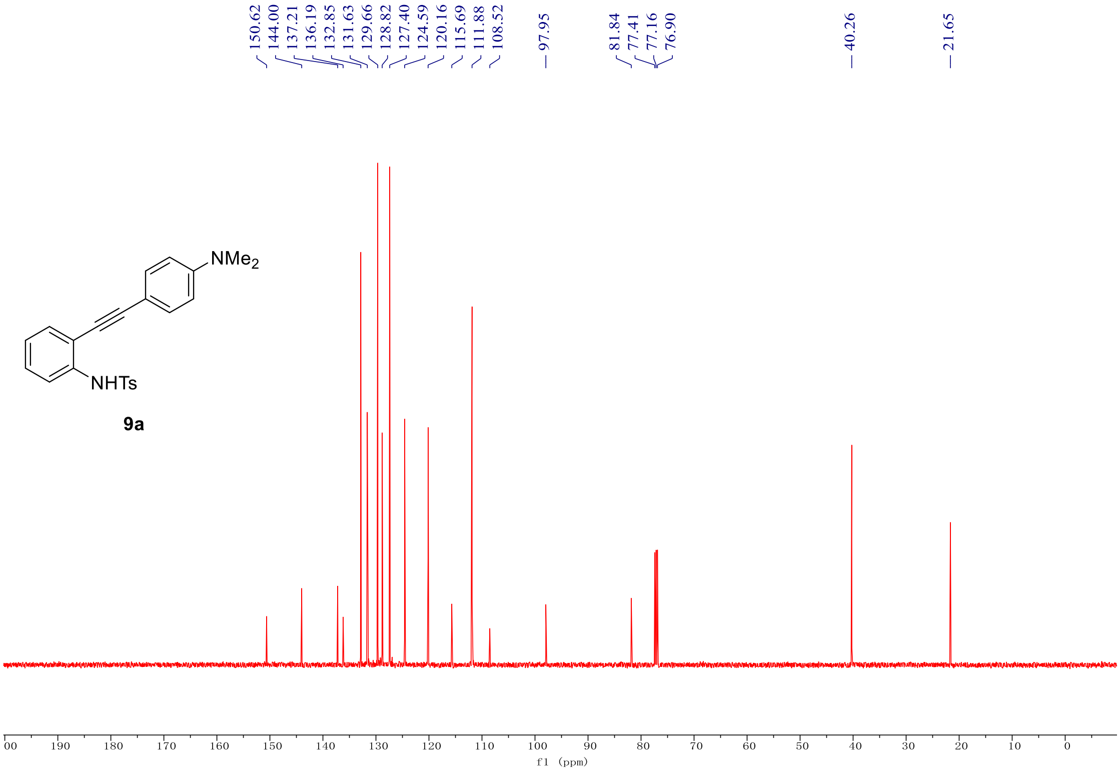

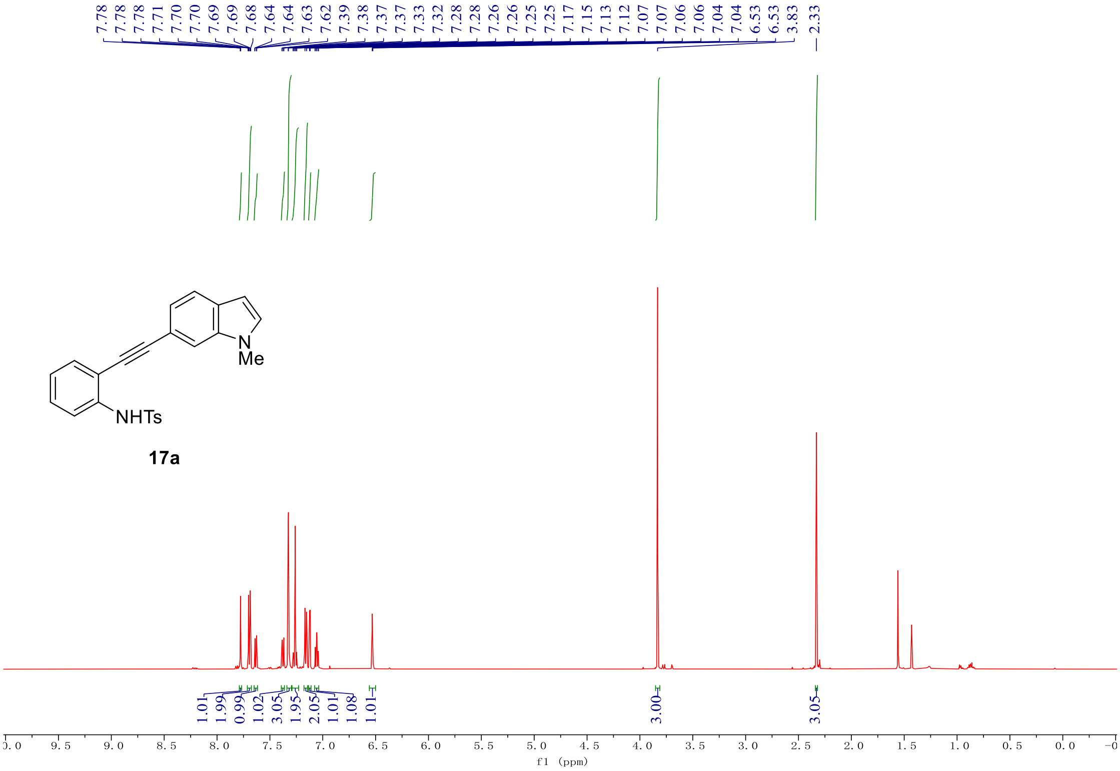
NHTs
N
Me
17a
f1 (ppm)

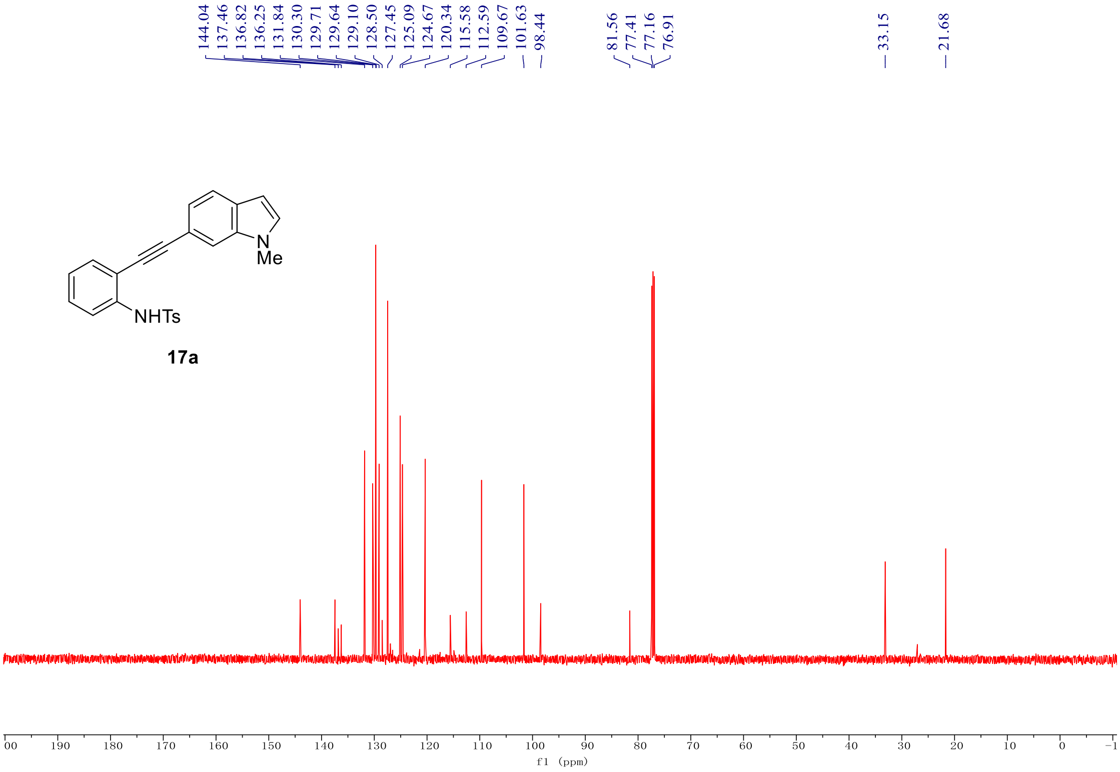
NHTs
N
Me
17a
f1 (ppm)

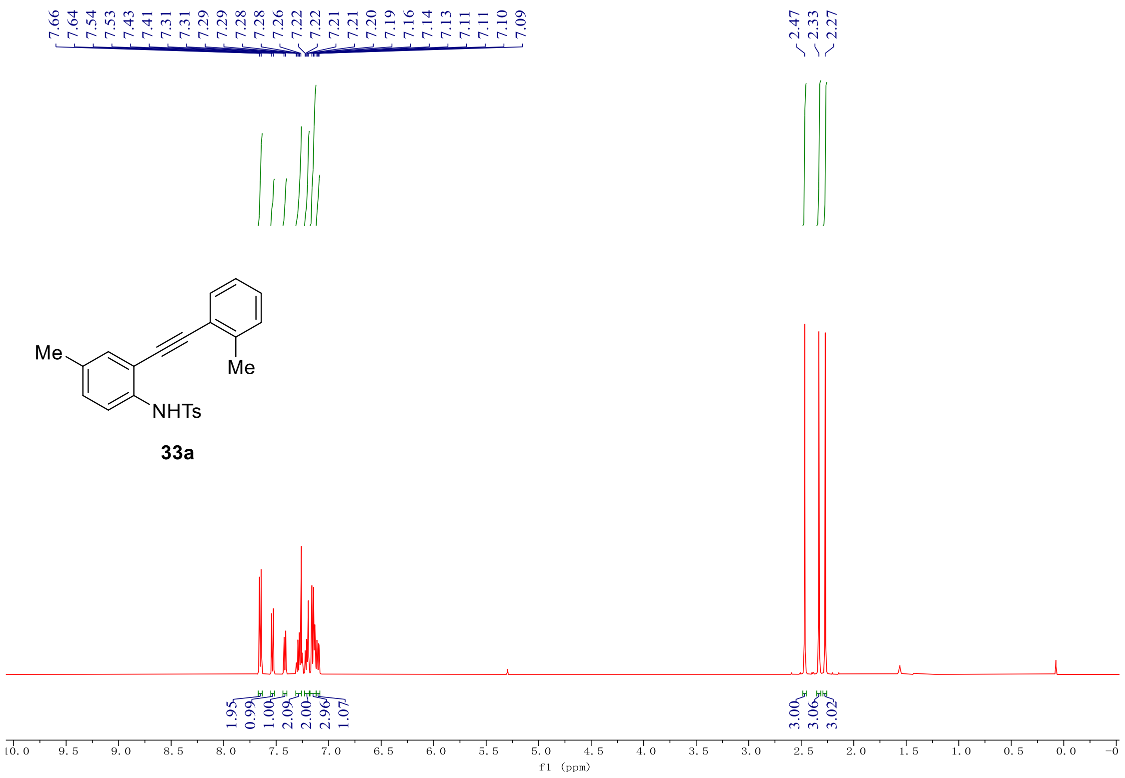

7.66
7.64
7.54
7.53
7.43
7.41
7.31
7.29
7.29
7.28
7.28
7.26
7.22
7.22
7.21
7.21
7.20
7.19
7.16
7.14
7.13
7.11
7.11
7.10
7.09
2.47
2.33
2.27
Me
Me
NHTs
33a
1.95
0.99
1.00
2.09
2.00
2.96
1.07
3.00
3.06
3.02
f1 (ppm)

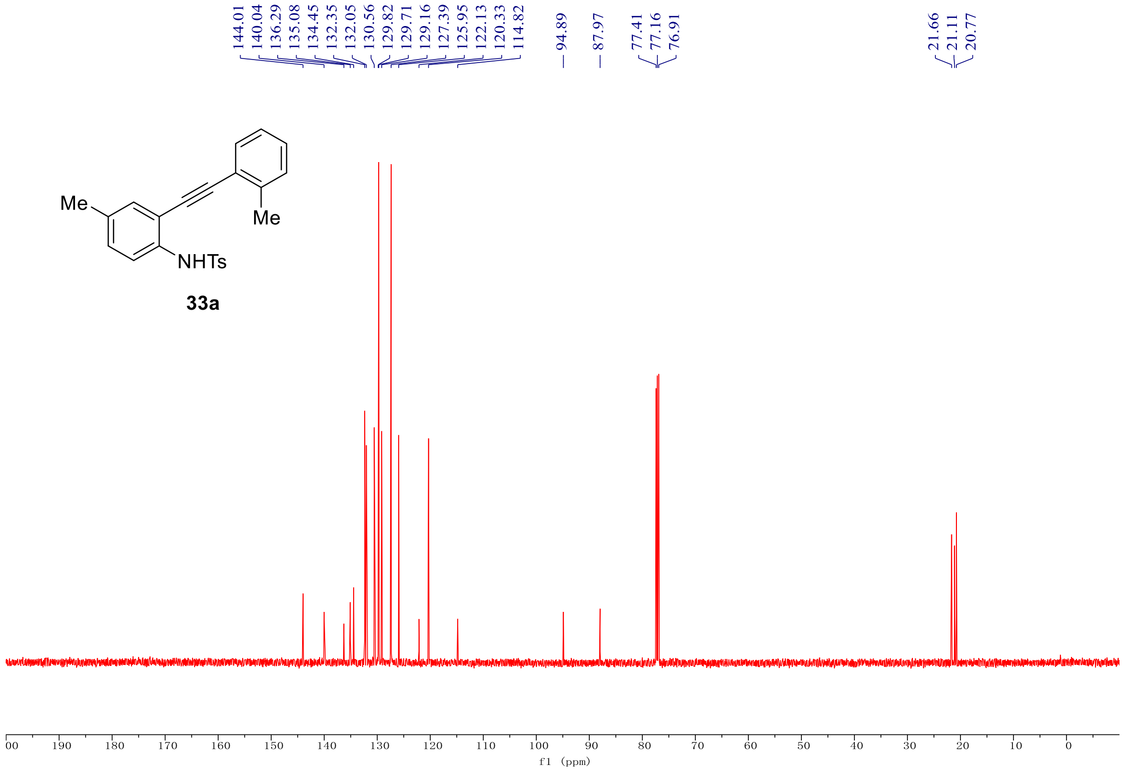

144.01
140.04
136.29
135.08
134.45
132.35
132.05
130.56
129.82
129.71
129.16
127.39
125.95
122.13
120.33
114.82
94.89
87.97
77.41
77.16
76.91
21.66
21.11
20.77
Me
Me
NHTs
33a
f1 (ppm)

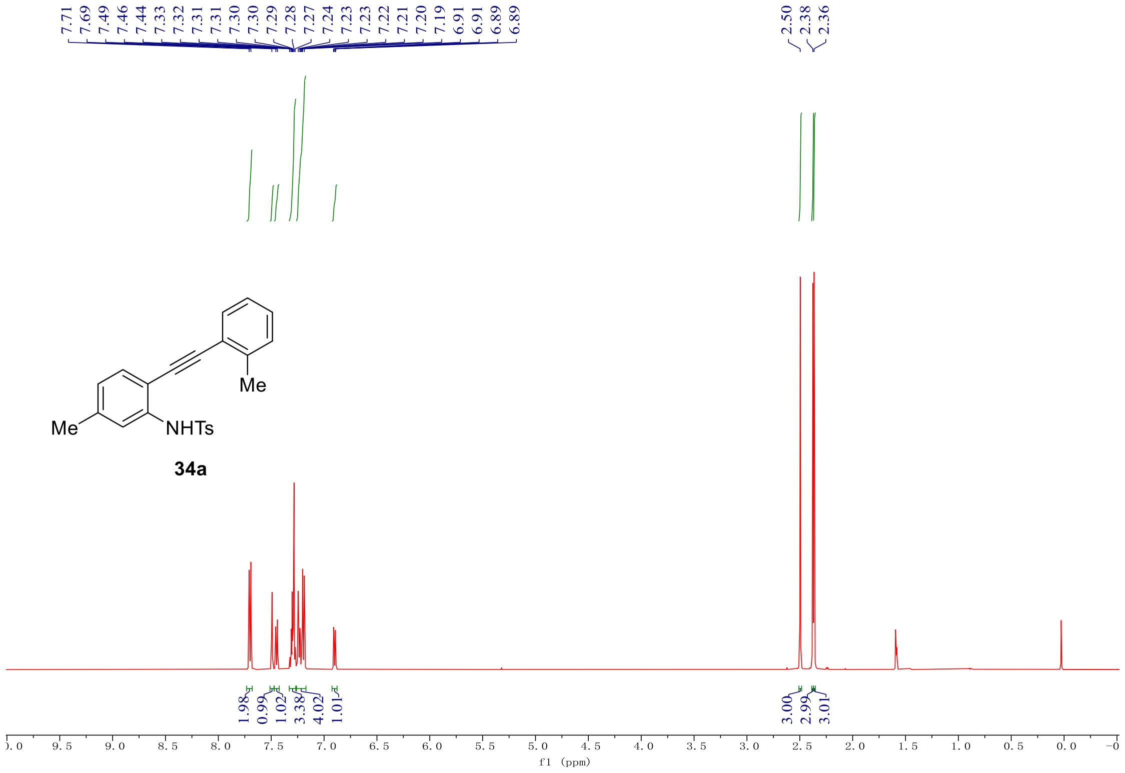
7.71
7.69
7.49
7.46
7.44
7.33
7.32
7.31
7.31
7.30
7.30
7.29
7.28
7.27
7.24
7.23
7.23
7.22
7.21
7.20
7.19
6.91
6.91
6.89
6.89
2.50
2.38
2.36
Me
Me
NHTs
34a
1.98
0.99
1.02
3.38
4.02
1.01
3.00
2.99
3.01
f1 (ppm)

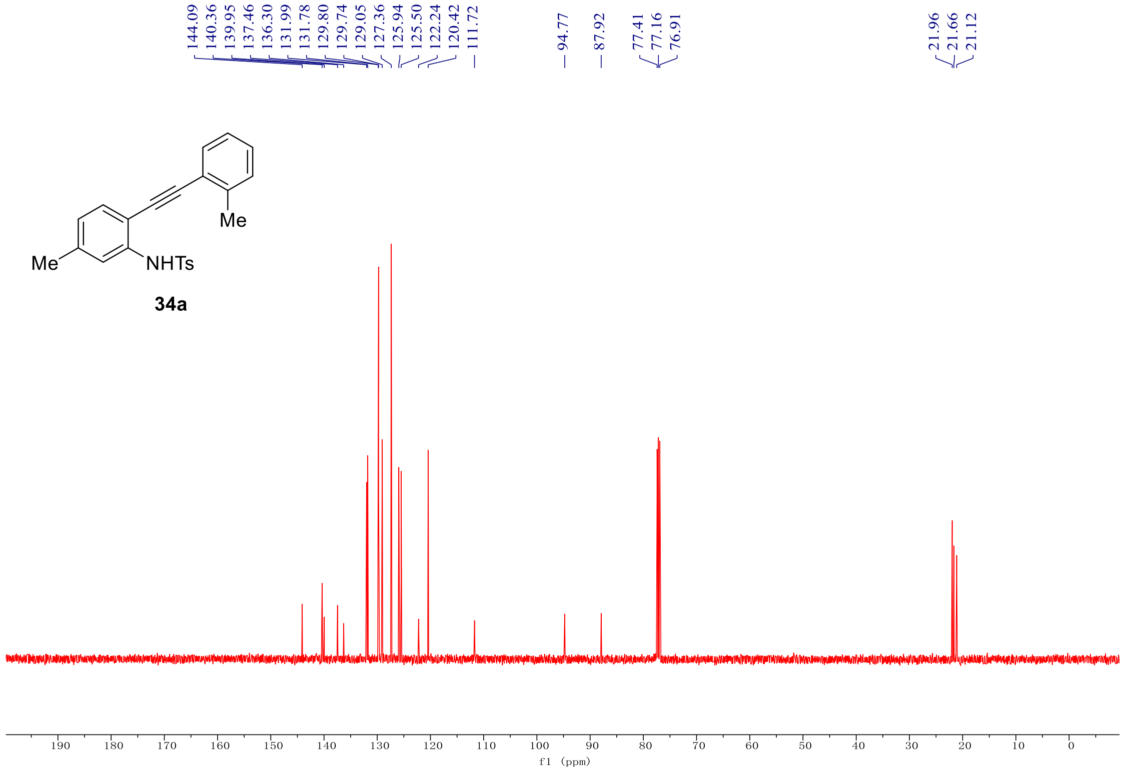
144.09
140.36
139.95
137.46
136.30
131.99
131.78
129.80
129.74
129.05
127.36
125.94
125.50
122.24
120.42
111.72
94.77
87.92
77.41
77.16
76.91
21.96
21.66
21.12
Me
Me
NHTs
34a
f1 (ppm)

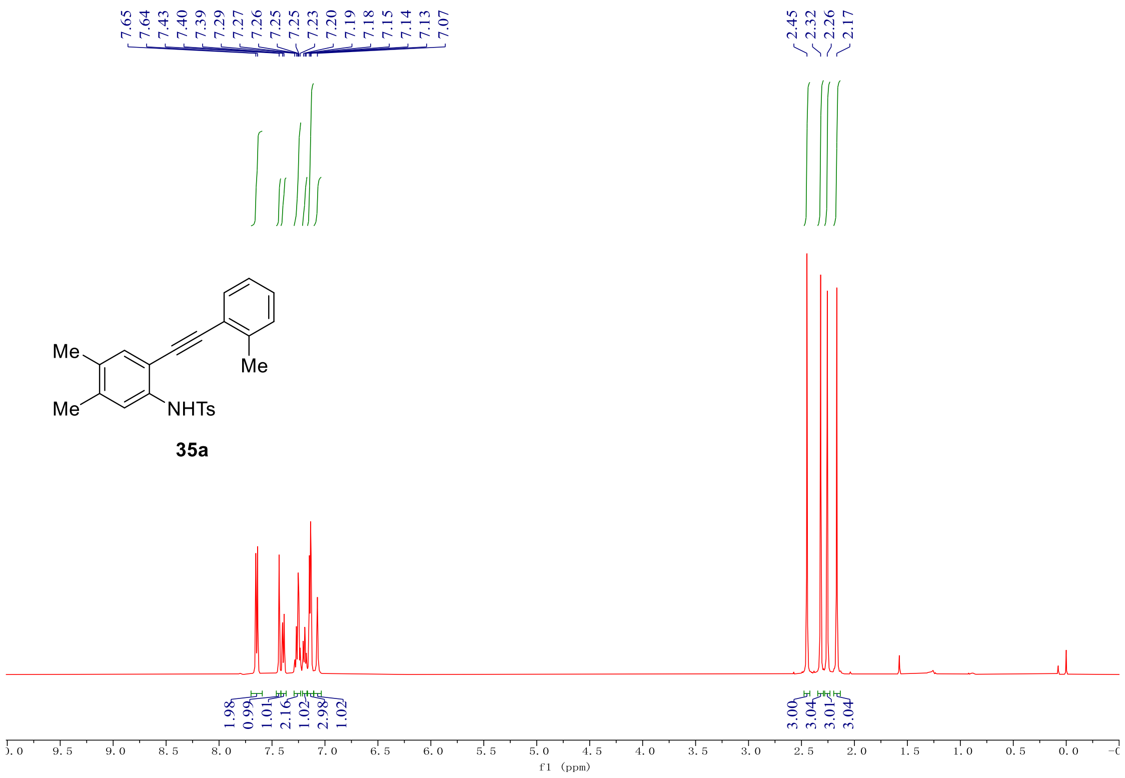

7.65
7.64
7.43
7.40
7.39
7.29
7.27
7.26
7.25
7.25
7.23
7.20
7.19
7.18
7.15
7.14
7.13
7.07
2.45
2.32
2.26
2.17
Me
Me
Me
NHTs
35a
1.98
0.99
1.01
2.16
1.02
2.98
1.02
3.00
3.04
3.01
3.04
f1 (ppm)

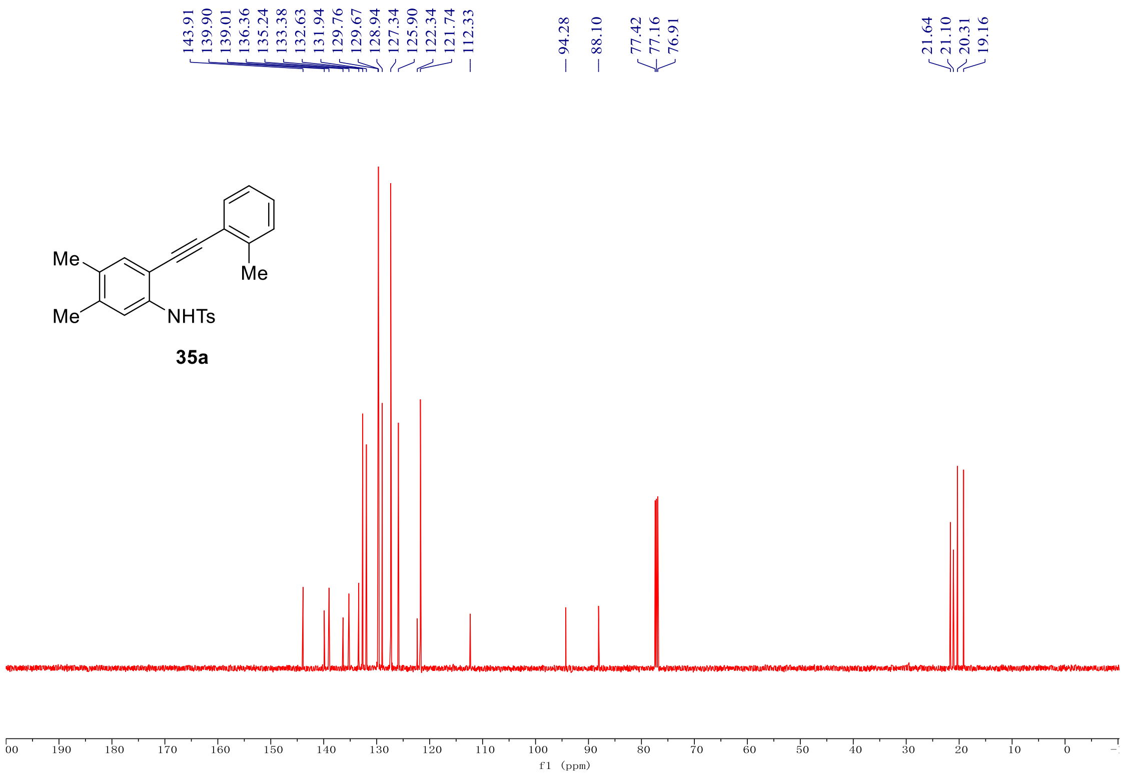

143.91
139.90
139.01
136.36
135.24
133.38
132.63
131.94
129.76
129.67
128.94
127.34
125.90
122.34
121.74
112.33
94.28
88.10
77.42
77.16
76.91
21.64
21.10
20.31
19.16
Me
Me
Me
NHTs
35a
f1 (ppm)

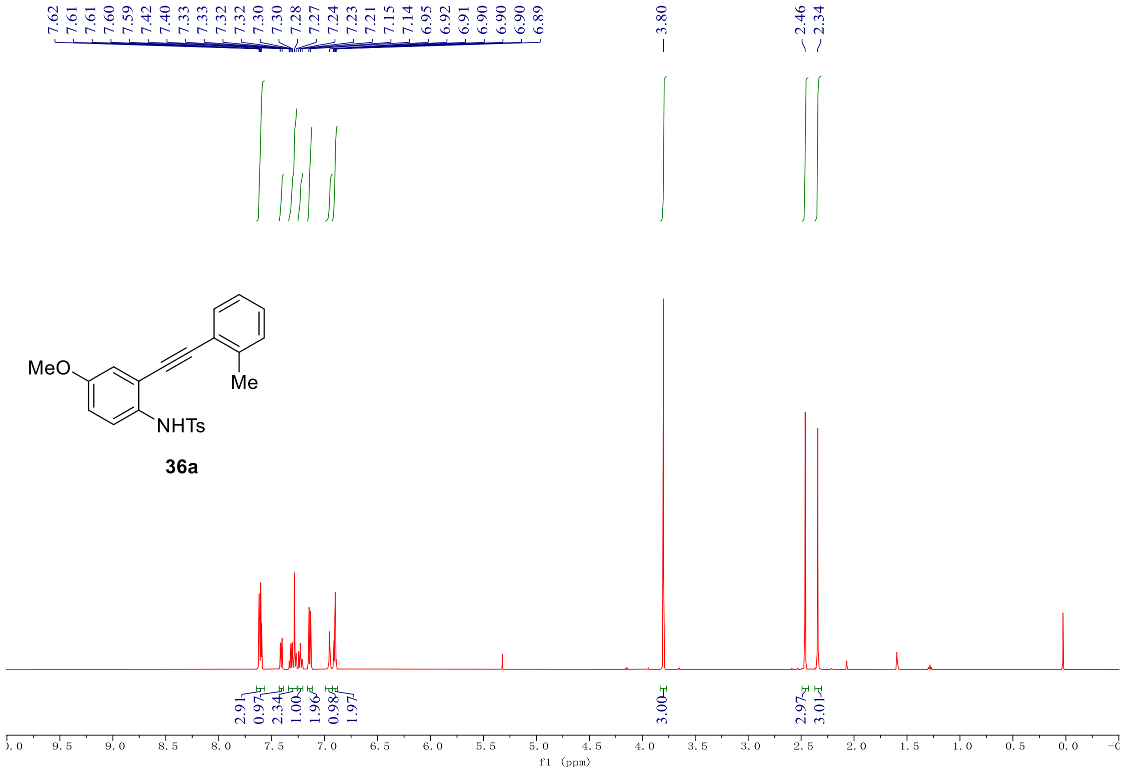
7.62 7.61 7.61 7.60 7.59 7.42 7.40 7.33 7.33 7.32 7.32 7.30 7.30 7.28 7.27 7.24 7.23 7.21 7.15 7.14 6.95 6.92 6.91 6.90 6.90 6.90 6.89
3.80
2.46 2.34
MeO
Me
NHTs
36a
2.91 0.97 2.34 1.00 1.96 0.98 1.97
3.00
2.97 3.01
f1 (ppm)

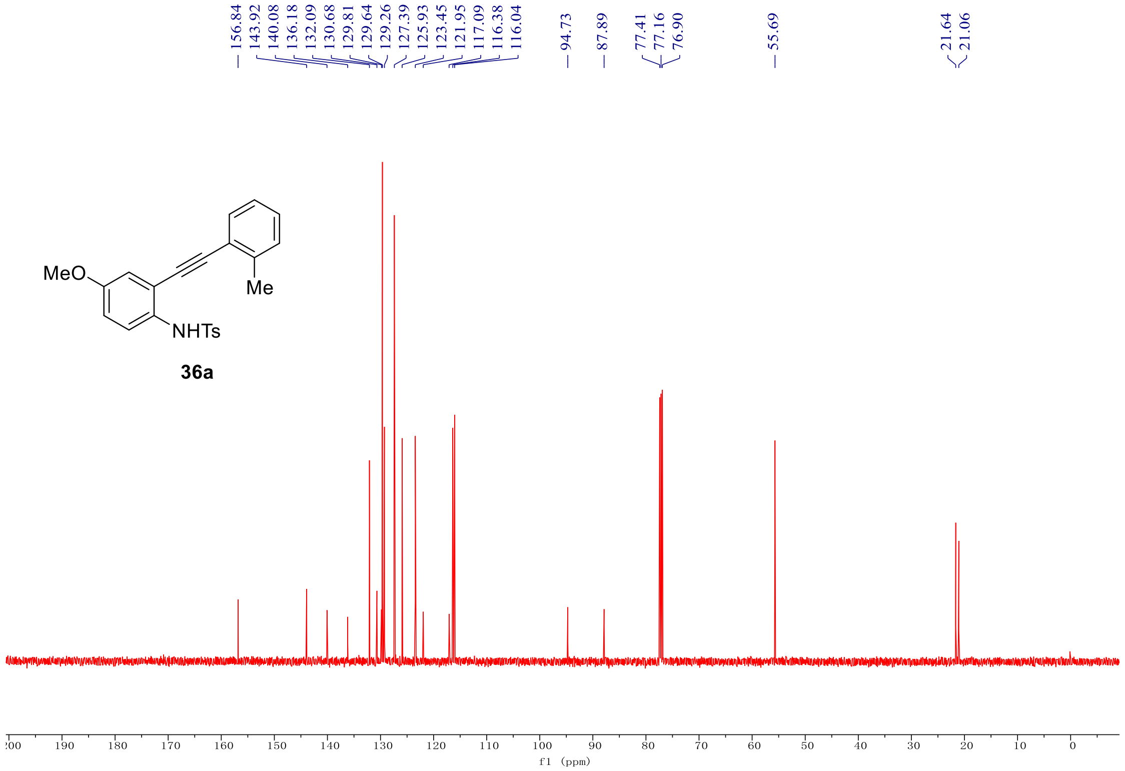
156.84 143.92 140.08 136.18 132.09 130.68 129.81 129.64 129.26 127.39 125.93 123.45 121.95 117.09 116.38 116.04
94.73
87.89
77.41 77.16 76.90
55.69
21.64 21.06
MeO
Me
NHTs
36a
f1 (ppm)

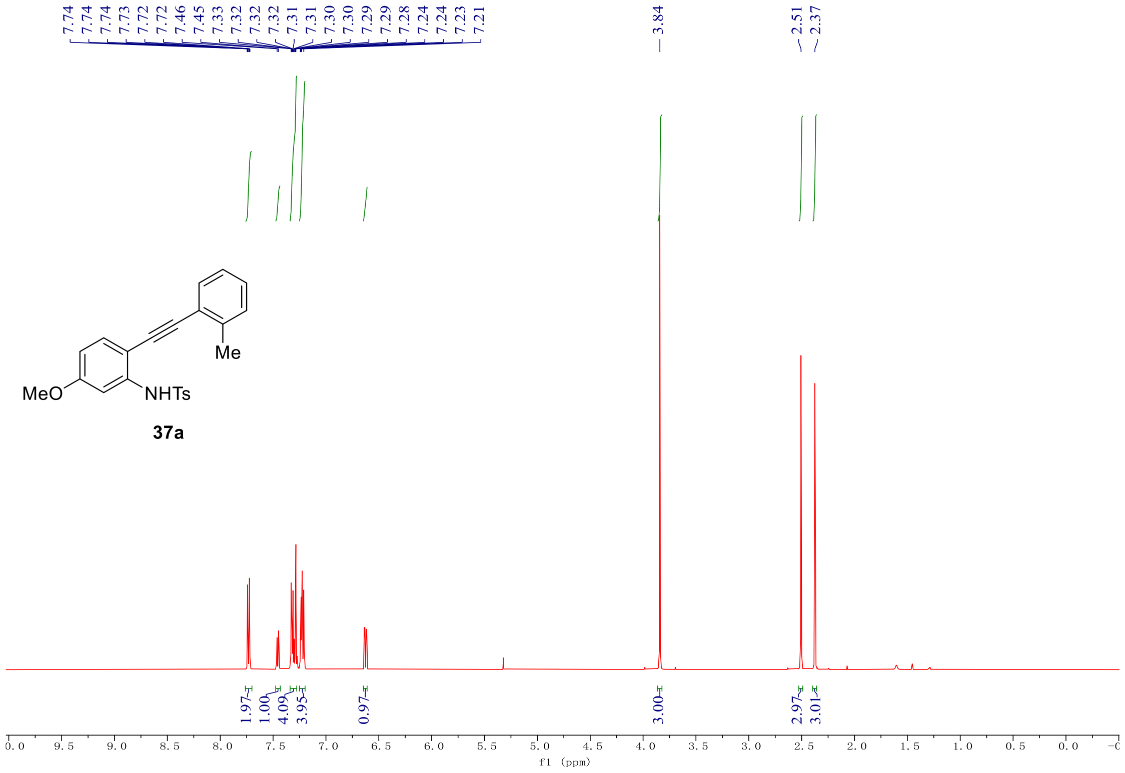
7.74 7.74 7.74 7.73 7.72 7.72 7.46 7.45 7.33 7.32 7.32 7.32 7.31 7.31 7.30 7.29 7.29 7.28 7.24 7.24 7.23 7.21
3.84
2.51 2.37
Me
MeO
NHTs
37a
1.97 1.00 4.09 3.95 0.97 3.00 2.97 3.01
f1 (ppm)

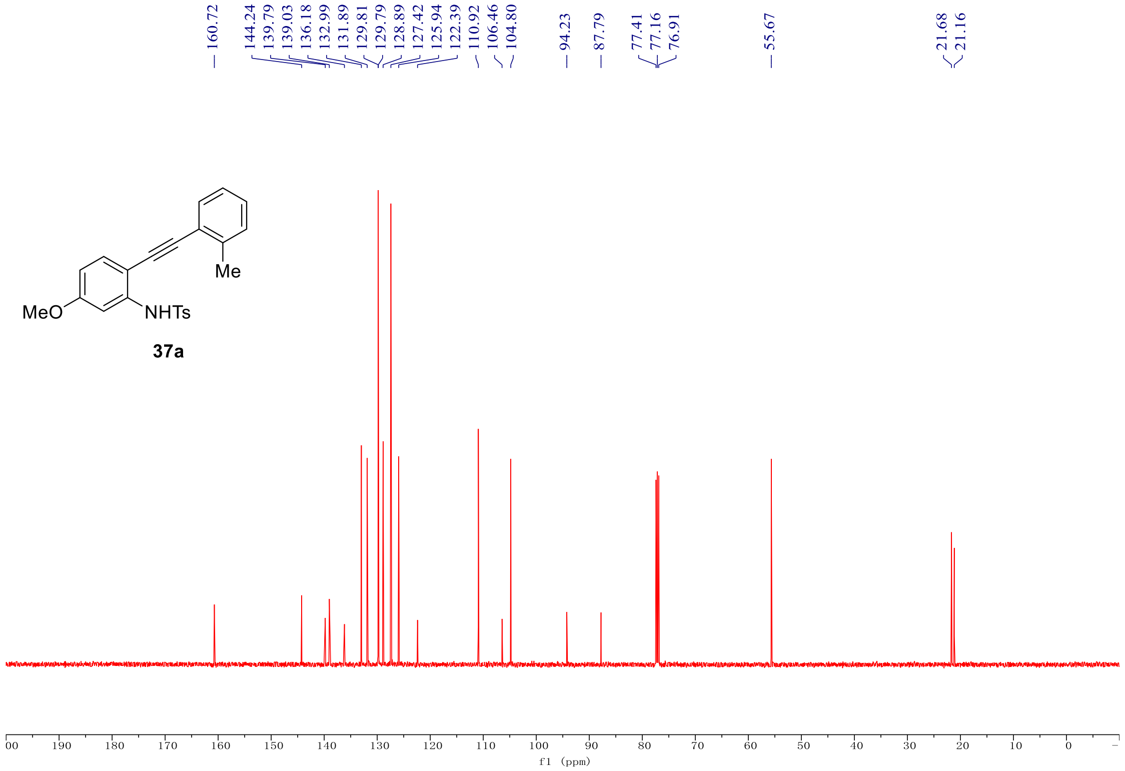
160.72
144.24 139.79 139.03 136.18 132.99 131.89 129.81 129.79 128.89 127.42 125.94 122.39 110.92 106.46 104.80
94.23 87.79
77.41 77.16 76.91
55.67
21.68 21.16
Me
MeO
NHTs
37a
f1 (ppm)

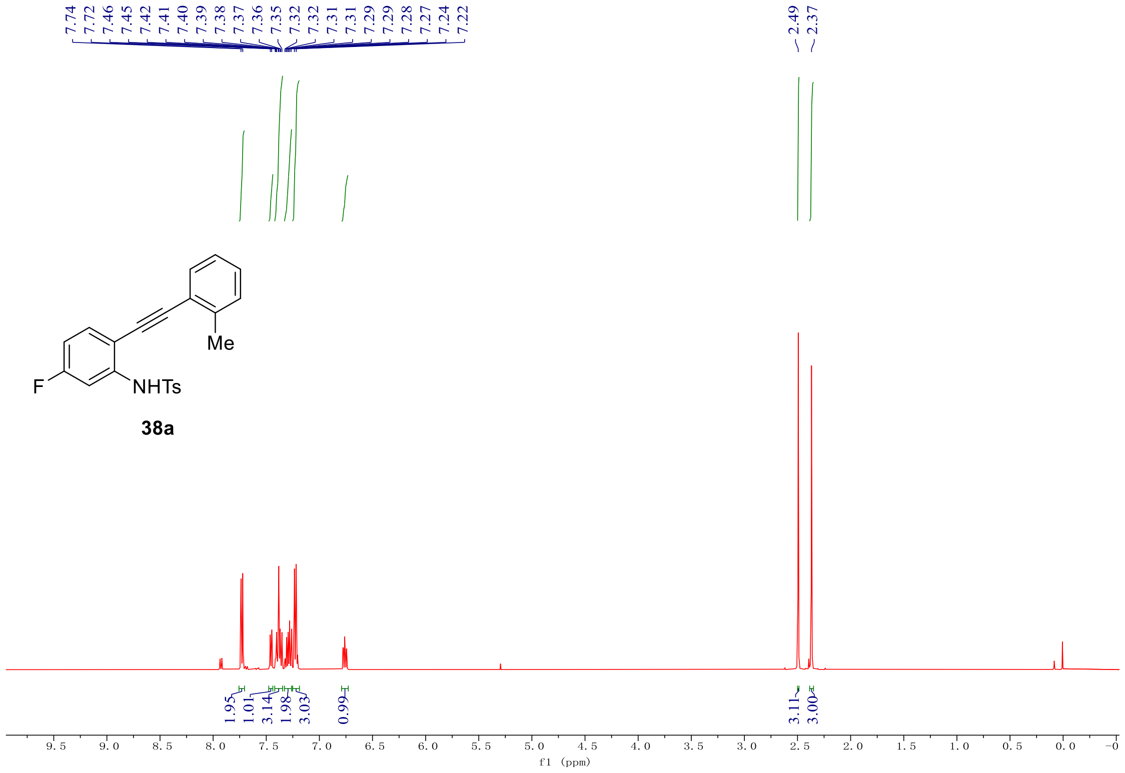
7.74
7.72
7.46
7.45
7.42
7.41
7.40
7.39
7.38
7.37
7.36
7.35
7.32
7.32
7.31
7.31
7.29
7.29
7.28
7.27
7.24
7.22
2.49
2.37
F
NHTs
Me
1.95
1.01
3.14
1.98
3.03
0.99
3.11
3.00
f1 (ppm)

38a

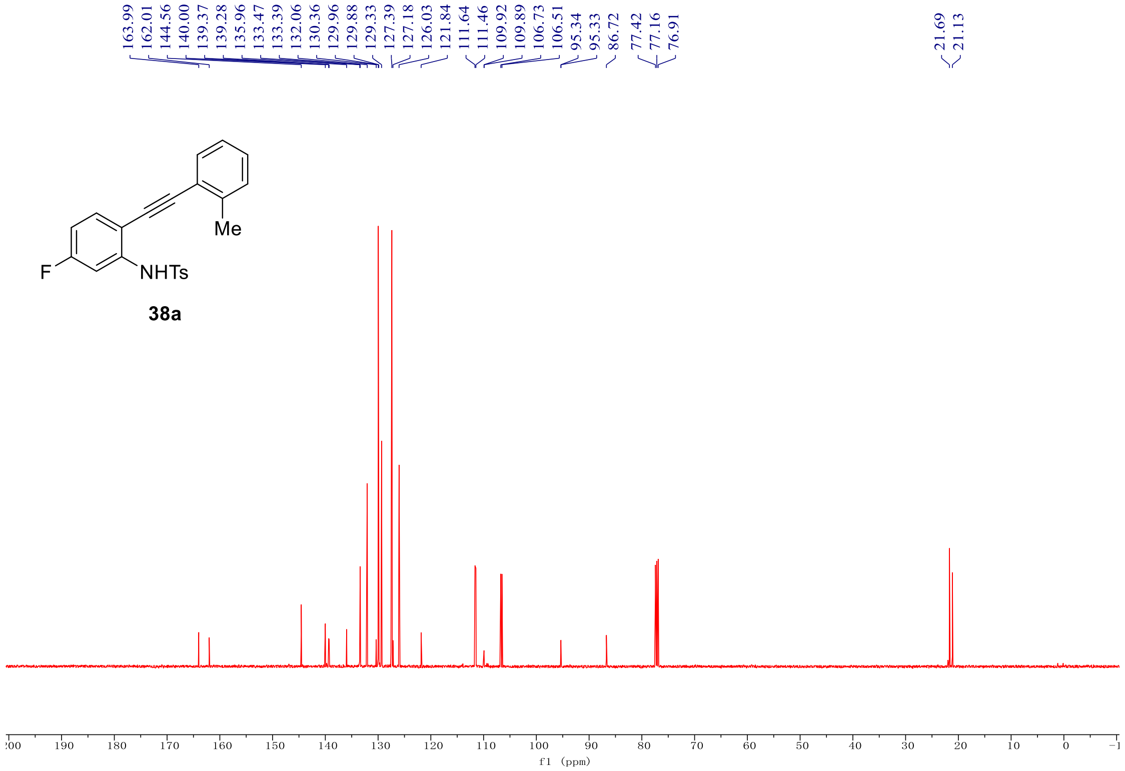
163.99
162.01
144.56
140.00
139.37
139.28
135.96
133.47
133.39
132.06
130.36
129.96
129.88
129.33
127.39
127.18
126.03
121.84
111.64
111.46
109.92
109.89
106.73
106.51
95.34
95.33
86.72
77.42
77.16
76.91
21.69
21.13
F
NHTs
Me
f1 (ppm)

38a

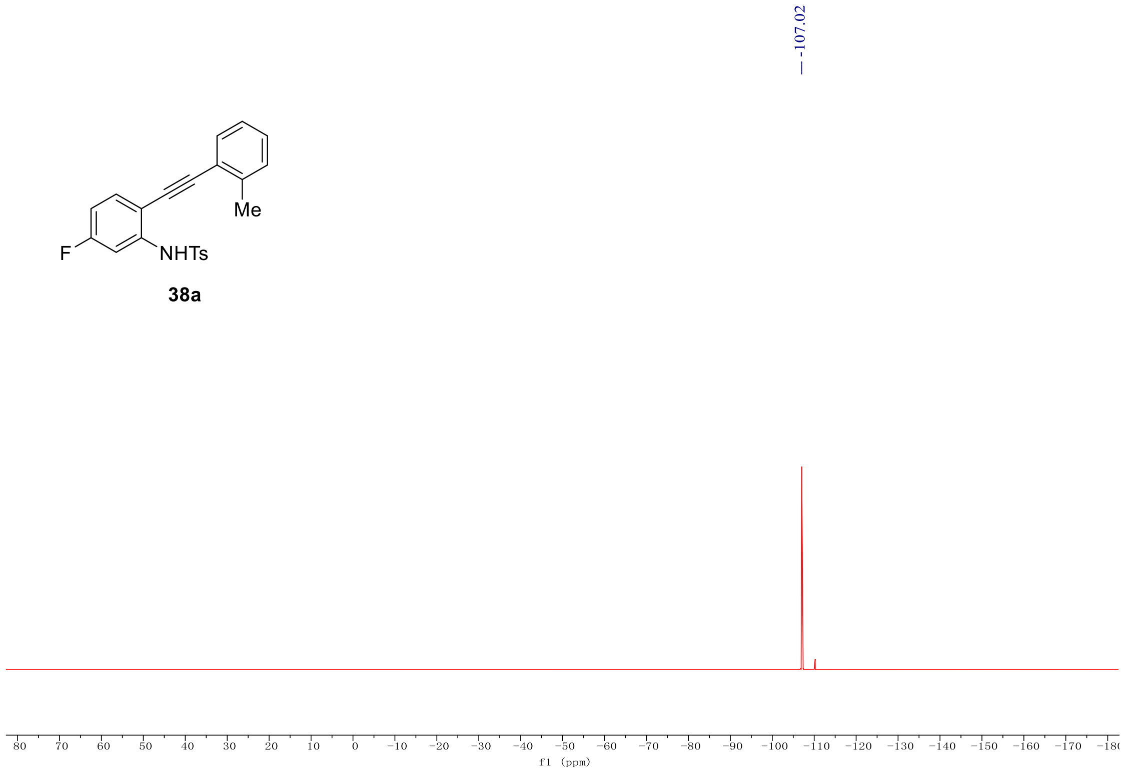
-107.02
F
NHTs
Me
f1 (ppm)

38a

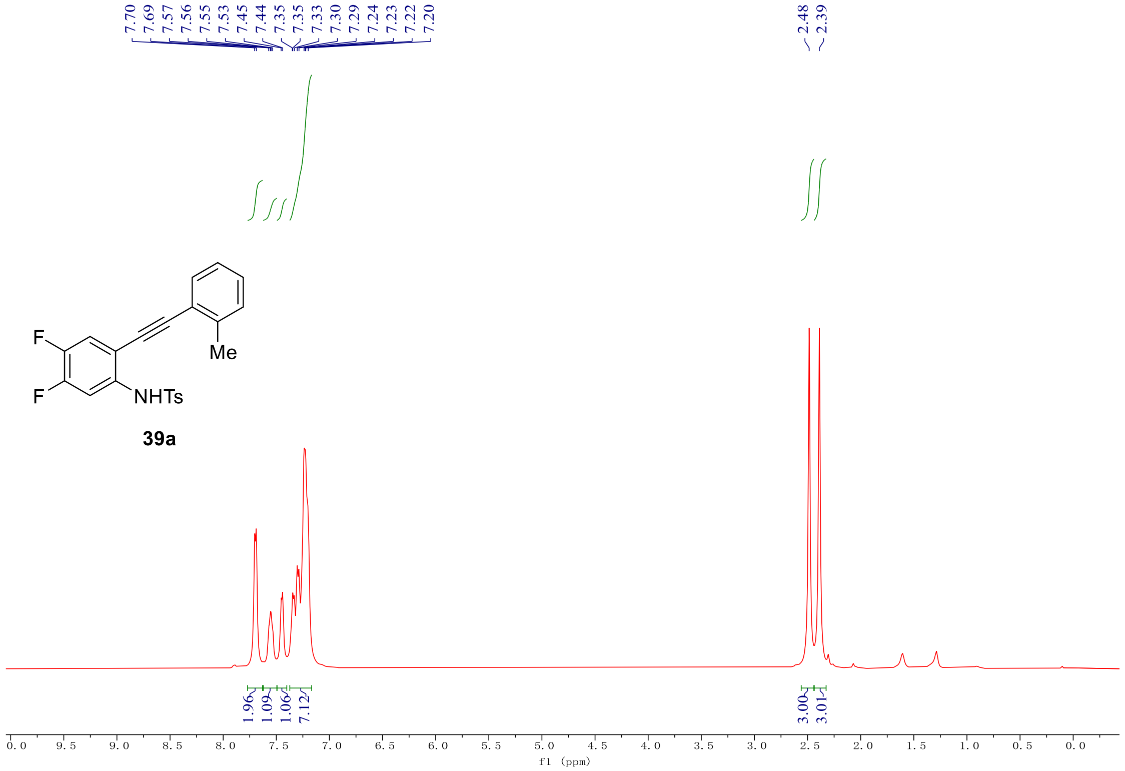
7.70
7.69
7.57
7.56
7.55
7.53
7.45
7.44
7.35
7.35
7.33
7.30
7.29
7.24
7.23
7.22
7.20
2.48
2.39
1.96
1.09
1.06
7.12
3.00
3.01
F
F
NHTs
Me
f1 (ppm)

39a

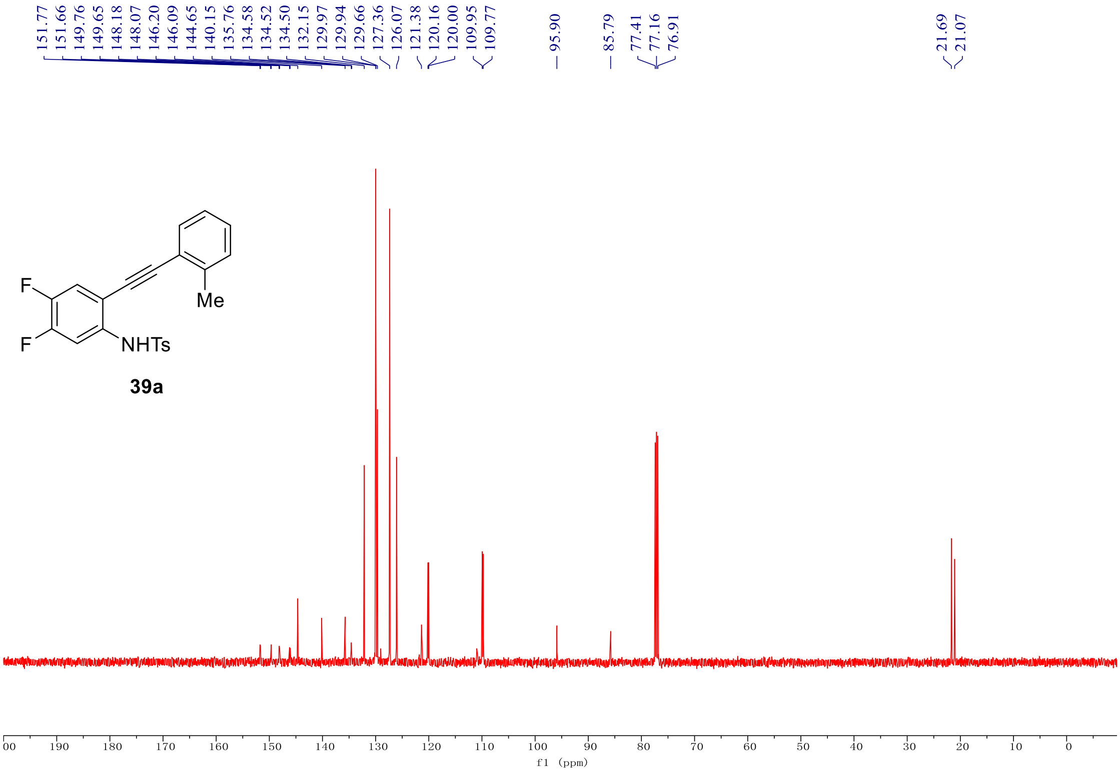
151.77
151.66
149.76
149.65
148.18
148.07
146.20
146.09
144.65
140.15
135.76
134.58
134.52
134.50
132.15
129.97
129.94
129.66
127.36
126.07
121.38
120.16
120.00
109.95
109.77
95.90
85.79
77.41
77.16
76.91
21.69
21.07
F
F
Me
NHTs
f1 (ppm)

39a

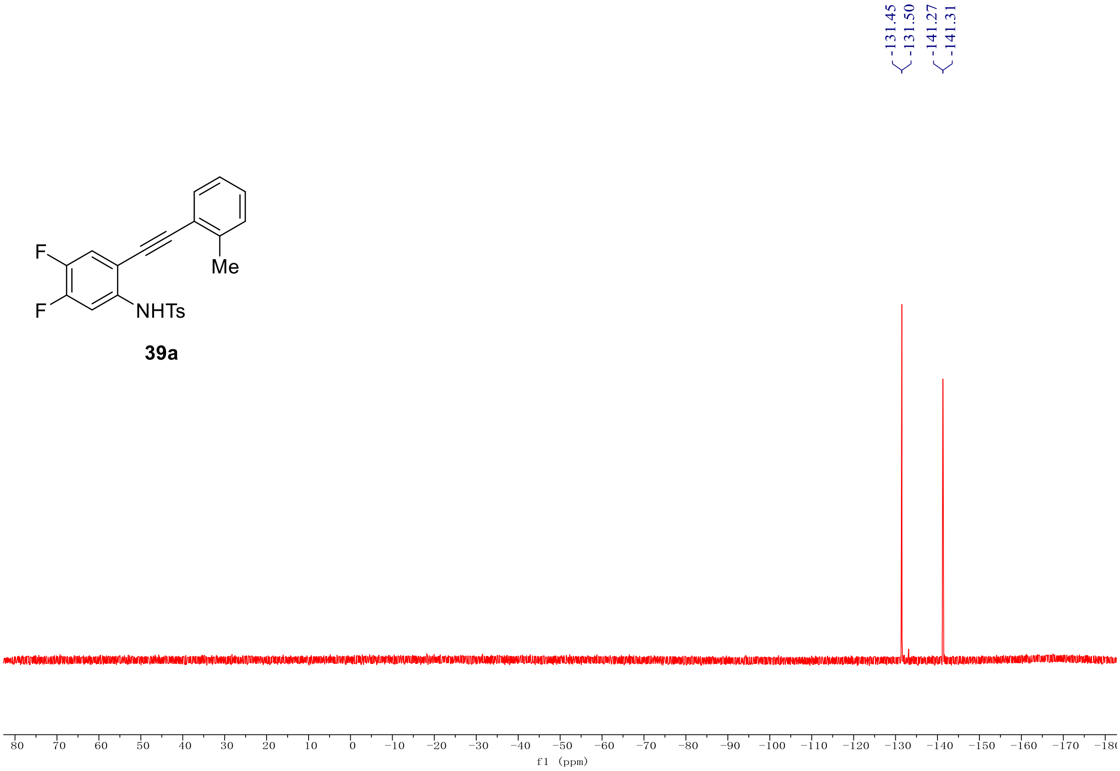
-131.45
-131.50
-141.27
-141.31
F
F
Me
NHTs
f1 (ppm)

39a

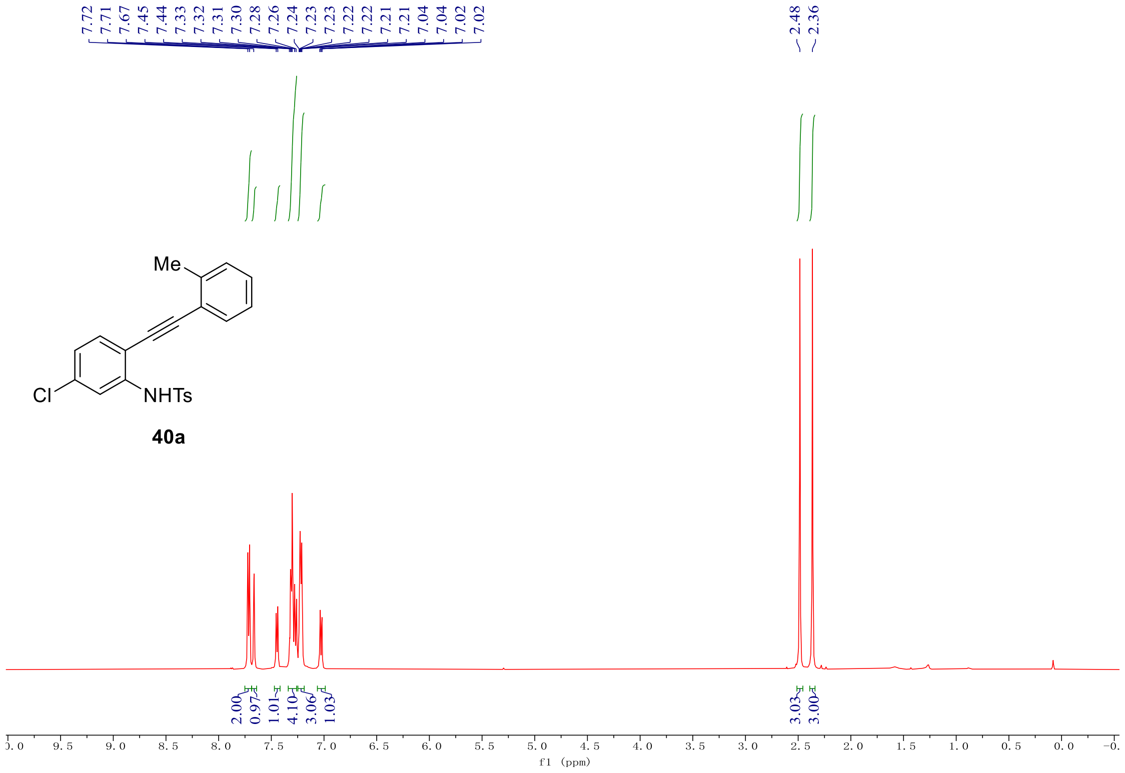
7.72
7.71
7.67
7.45
7.44
7.33
7.32
7.31
7.30
7.28
7.26
7.24
7.23
7.23
7.22
7.22
7.21
7.21
7.04
7.04
7.02
7.02
2.48
2.36
Me
Cl
NHTs
40a
2.00
0.97
1.01
4.10
3.06
1.03
3.03
3.00
f1 (ppm)

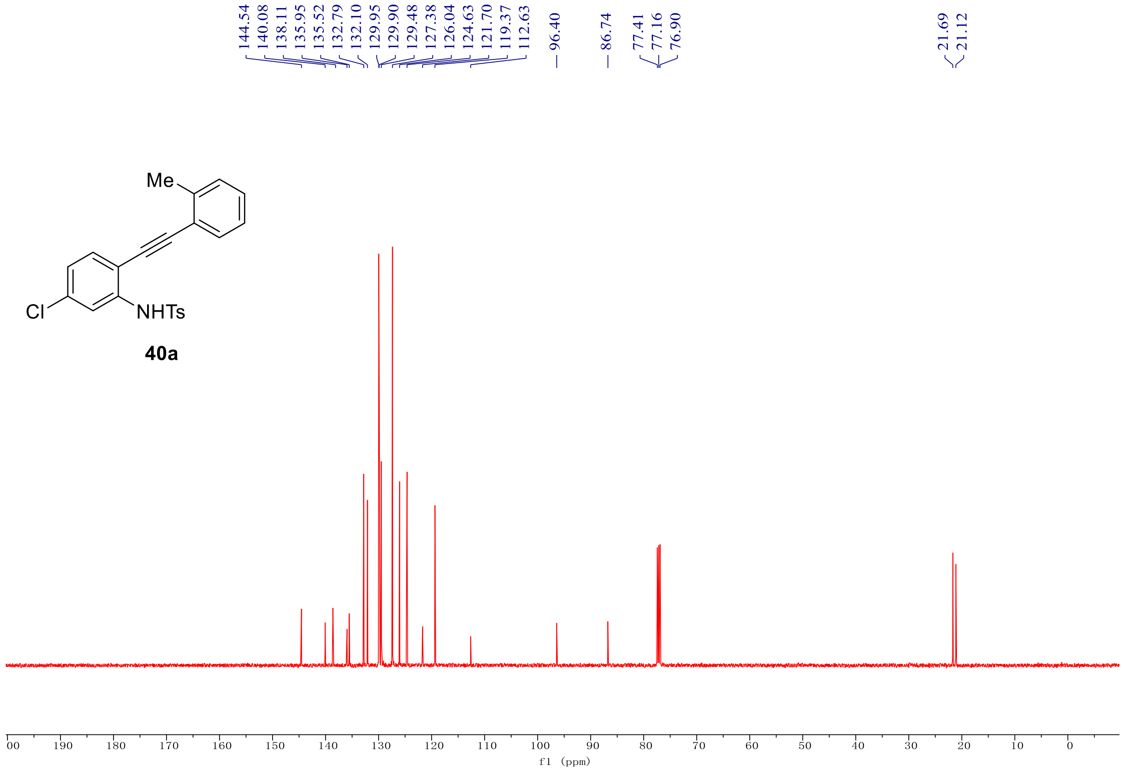
144.54
140.08
138.11
135.95
135.52
132.79
132.10
129.95
129.90
129.48
127.38
126.04
124.63
121.70
119.37
112.63
96.40
86.74
77.41
77.16
76.90
21.69
21.12
Me
Cl
NHTs
40a
f1 (ppm)

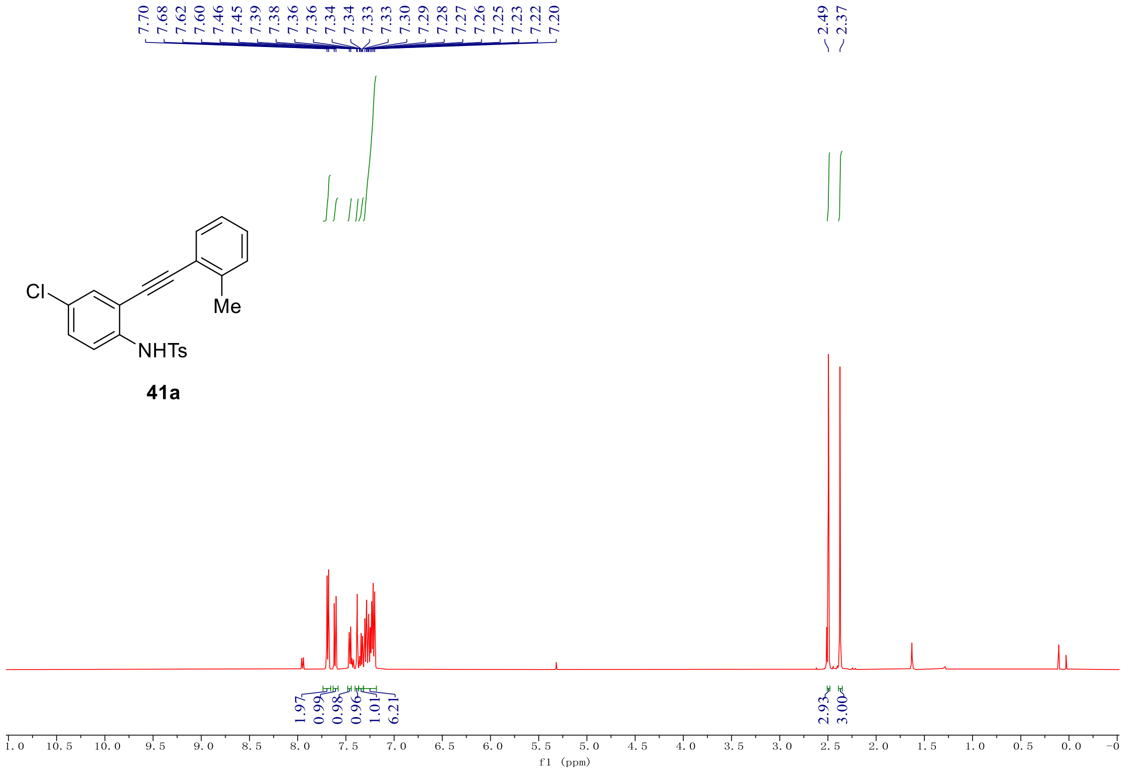

7.70
7.68
7.62
7.60
7.46
7.45
7.39
7.38
7.36
7.36
7.34
7.34
7.33
7.33
7.30
7.29
7.28
7.27
7.26
7.25
7.23
7.22
7.20
2.49
2.37
Cl
Me
NHTs
41a
1.97
0.99
0.98
0.96
1.01
6.21
2.93
3.00
f1 (ppm)

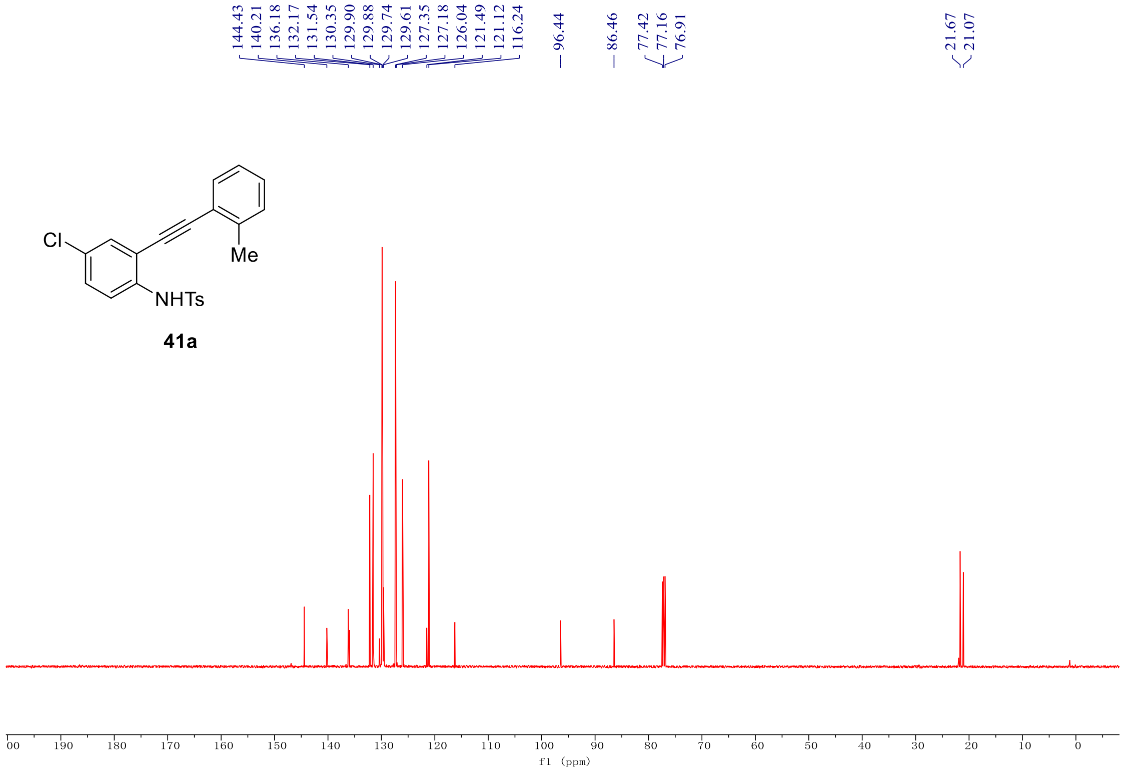

144.43
140.21
136.18
132.17
131.54
130.35
129.90
129.88
129.74
129.61
127.35
127.18
126.04
121.49
121.12
116.24
96.44
86.46
77.42
77.16
76.91
21.67
21.07
Cl
Me
NHTs
41a
f1 (ppm)

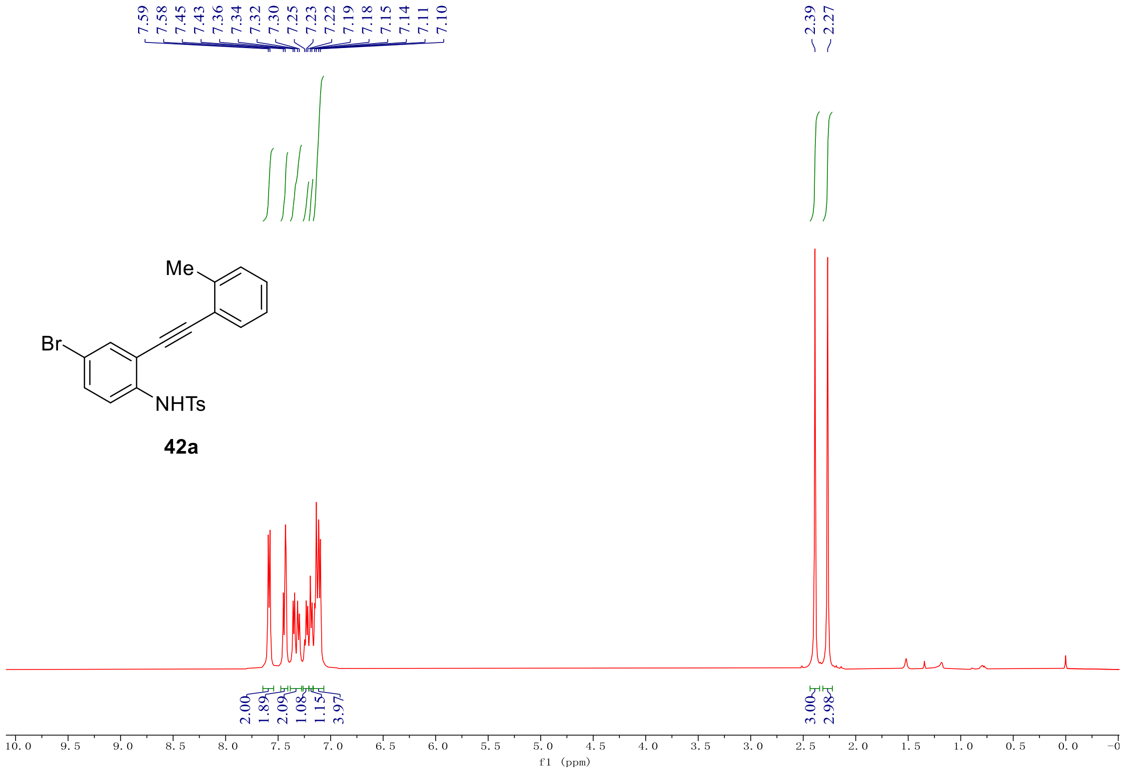
7.59
7.58
7.45
7.43
7.36
7.34
7.32
7.30
7.25
7.23
7.22
7.19
7.18
7.15
7.14
7.11
7.10
2.39
2.27
Me
Br
NHTs
42a
2.00
1.89
2.09
1.08
1.15
3.97
3.00
2.98
f1 (ppm)

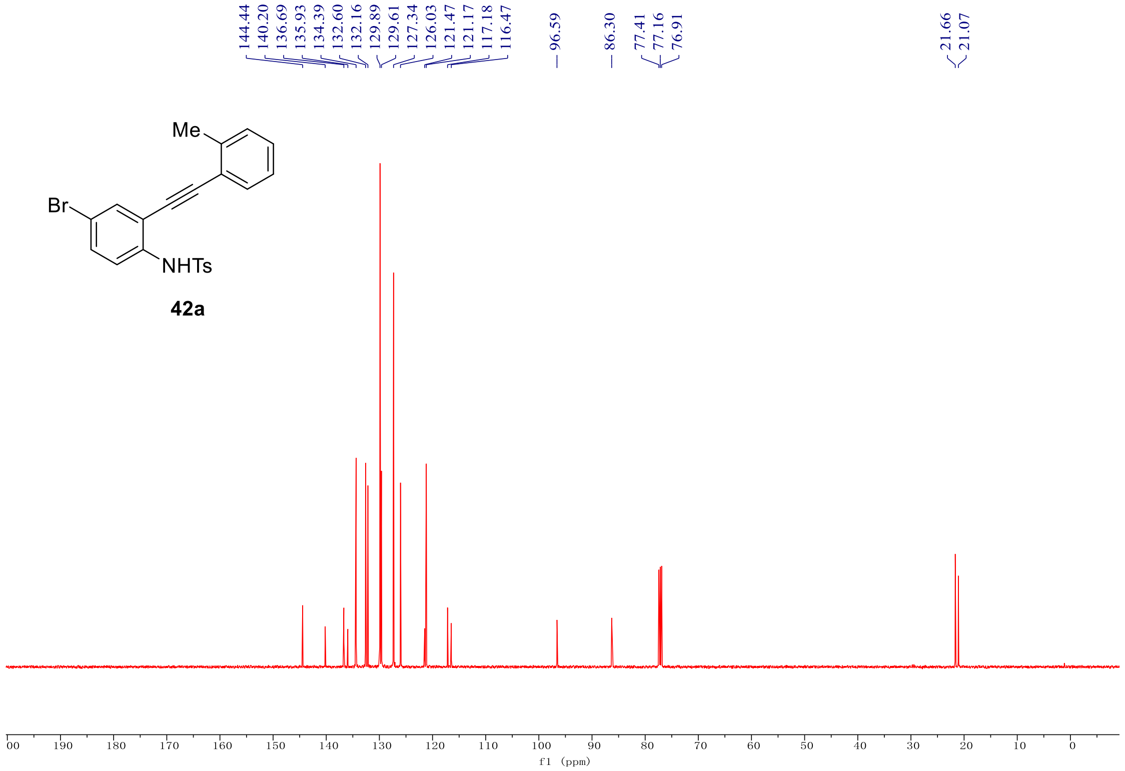
144.44
140.20
136.69
135.93
134.39
132.60
132.16
129.89
129.61
127.34
126.03
121.47
121.17
117.18
116.47
96.59
86.30
77.41
77.16
76.91
21.66
21.07
Me
Br
NHTs
42a
f1 (ppm)

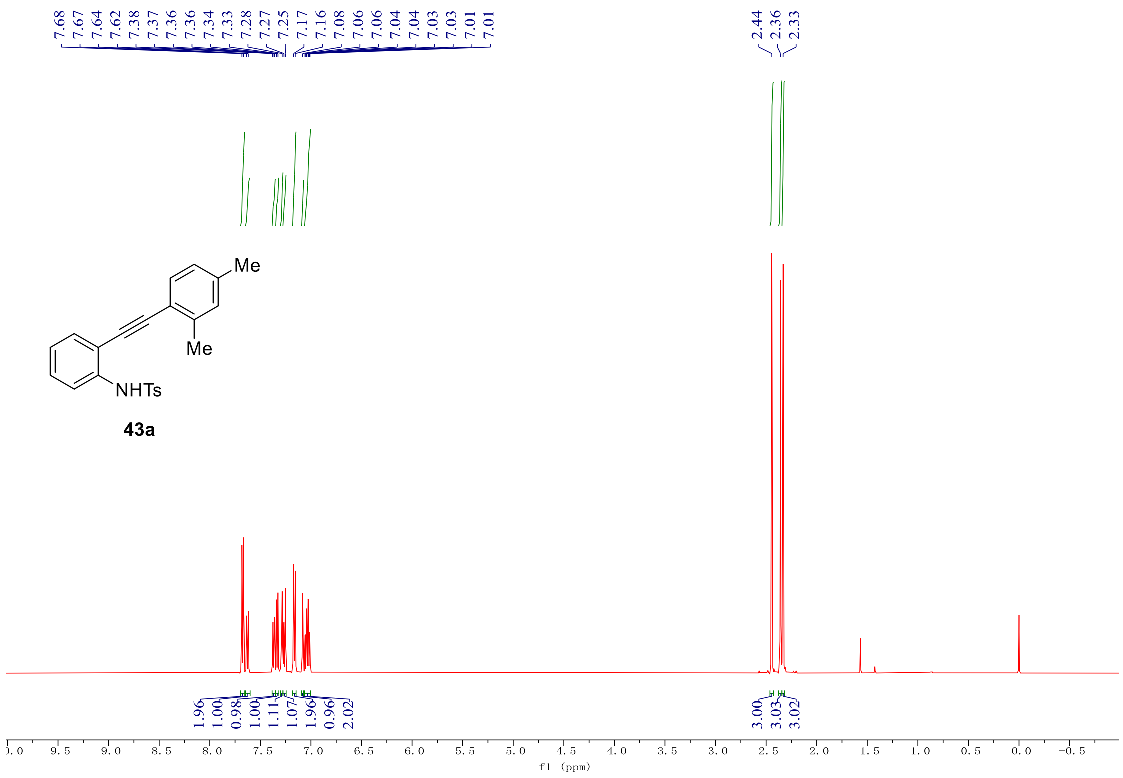
7.68
7.67
7.64
7.62
7.38
7.37
7.36
7.36
7.34
7.33
7.28
7.27
7.25
7.17
7.16
7.08
7.06
7.06
7.04
7.04
7.03
7.03
7.01
7.01
2.44
2.36
2.33
Me
Me
NHTs
43a
1.96
1.00
0.98
1.00
1.11
1.07
1.96
0.96
2.02
3.00
3.03
3.02
f1 (ppm)

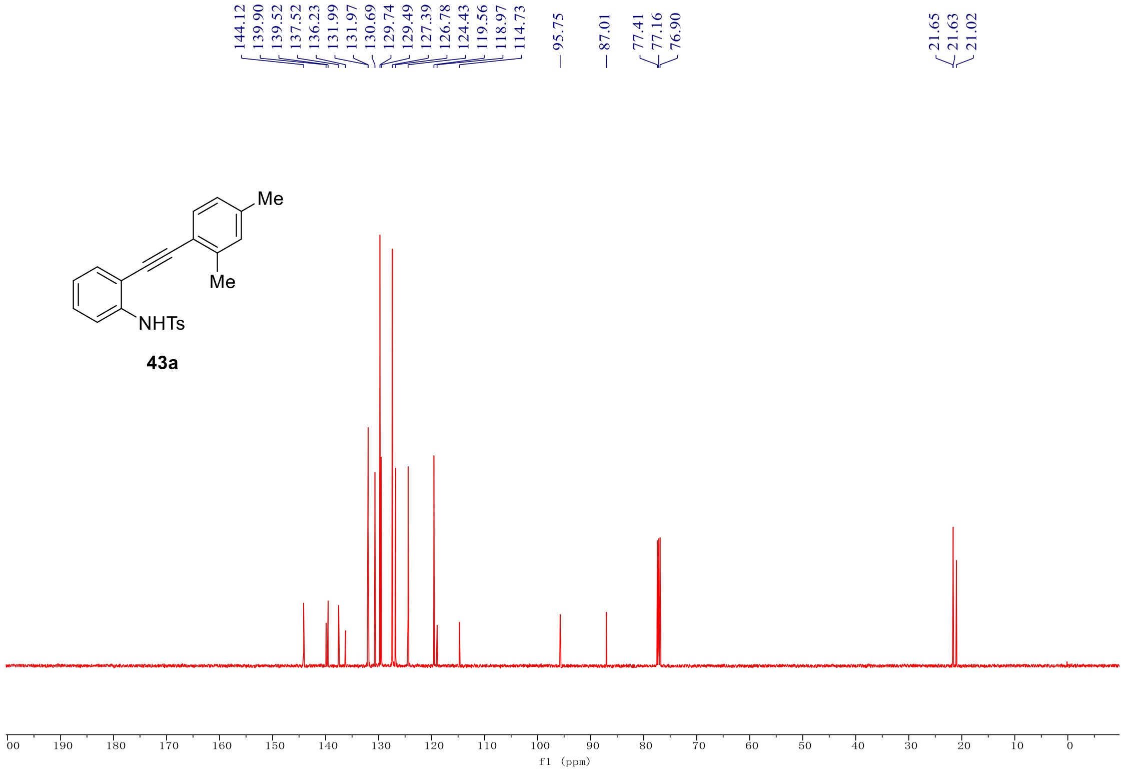
144.12
139.90
139.52
137.52
136.23
131.99
131.97
130.69
129.74
129.49
127.39
126.78
124.43
119.56
118.97
114.73
95.75
87.01
77.41
77.16
76.90
21.65
21.63
21.02
Me
Me
NHTs
43a
f1 (ppm)

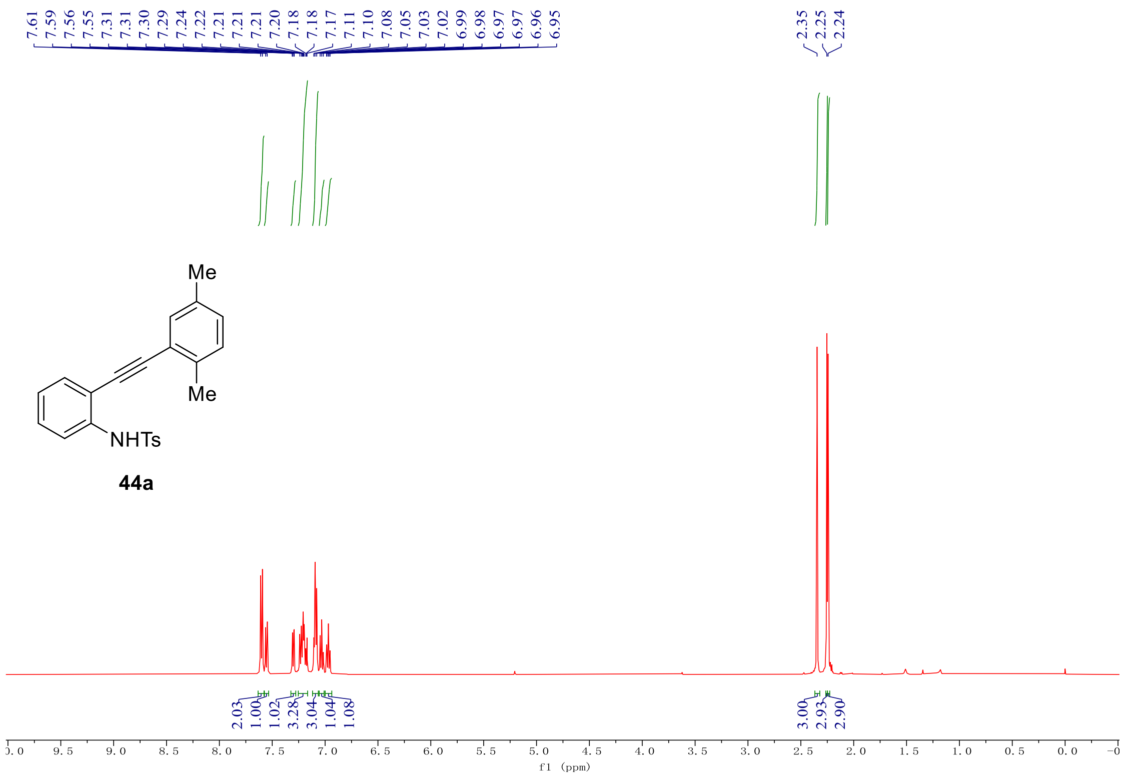
7.61 7.59 7.56 7.55 7.31 7.31 7.30 7.29 7.24 7.22 7.21 7.21 7.21 7.20 7.18 7.18 7.17 7.11 7.10 7.08 7.05 7.03 7.02 6.99 6.98 6.97 6.97 6.96 6.95
2.35 2.25 2.24
Me
Me
NHTs
44a
2.03 1.00 1.02 3.28 3.04 1.04 1.08
3.00 2.93 2.90
f1 (ppm)

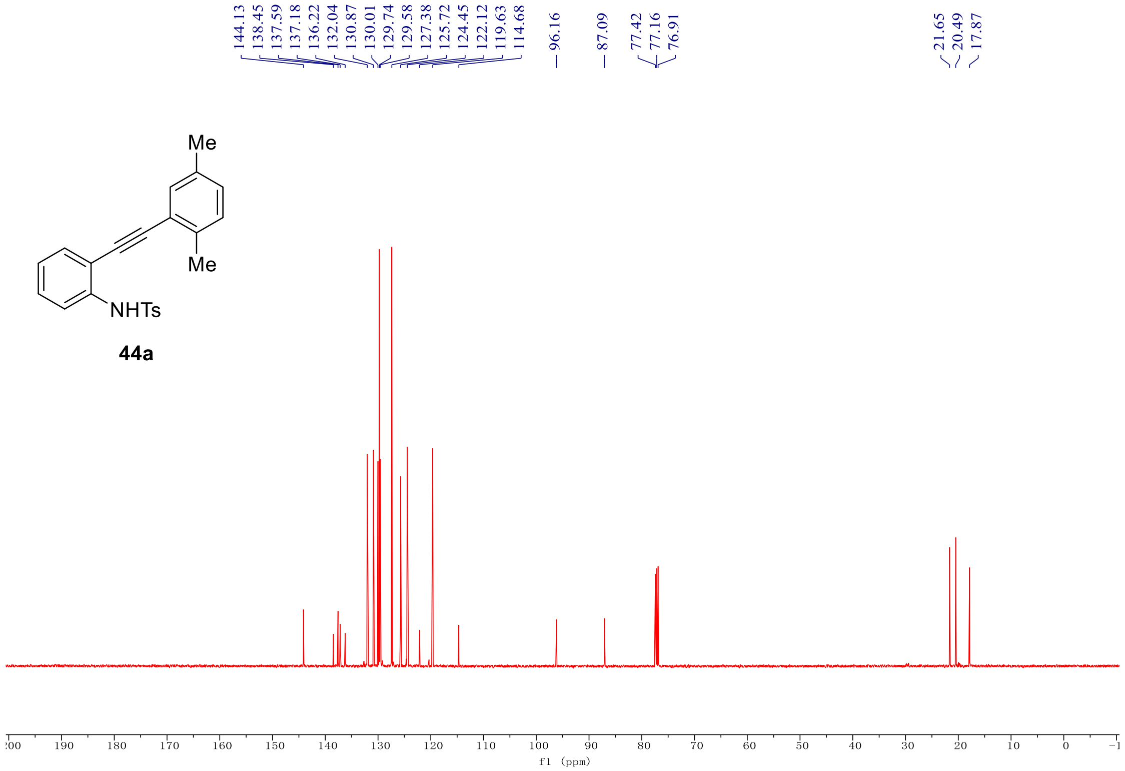
144.13 138.45 137.59 137.18 136.22 132.04 130.87 130.01 129.74 129.58 127.38 125.72 124.45 122.12 119.63 114.68
96.16
87.09
77.42 77.16 76.91
21.65 20.49 17.87
Me
Me
NHTs
44a
f1 (ppm)

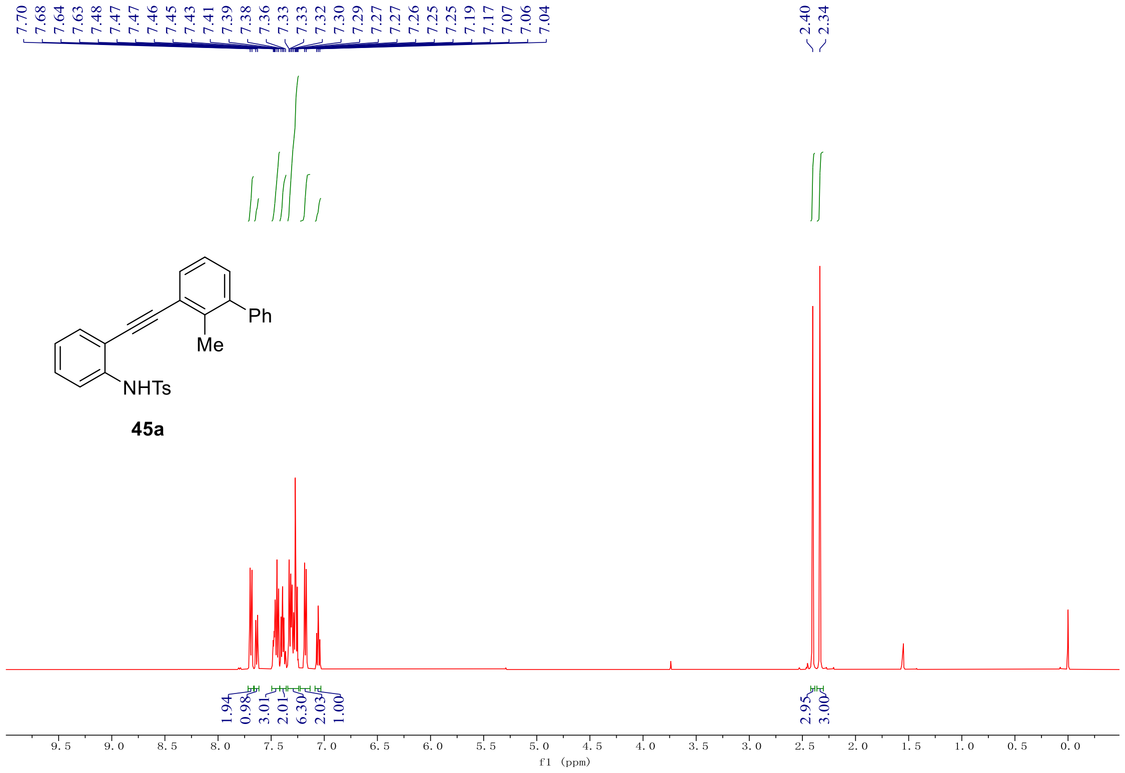

7.70
7.68
7.64
7.63
7.48
7.47
7.47
7.46
7.45
7.43
7.41
7.39
7.38
7.36
7.33
7.33
7.33
7.32
7.30
7.29
7.27
7.27
7.26
7.25
7.25
7.19
7.17
7.07
7.06
7.04
2.40
2.34
Ph
Me
NHTs
45a
1.94
0.98
3.01
2.01
6.30
2.03
1.00
2.95
3.00
9.5
9.0
8.5
8.0
7.5
7.0
6.5
6.0
5.5
5.0
4.5
4.0
3.5
3.0
2.5
2.0
1.5
1.0
0.5
0.0
f1 (ppm)

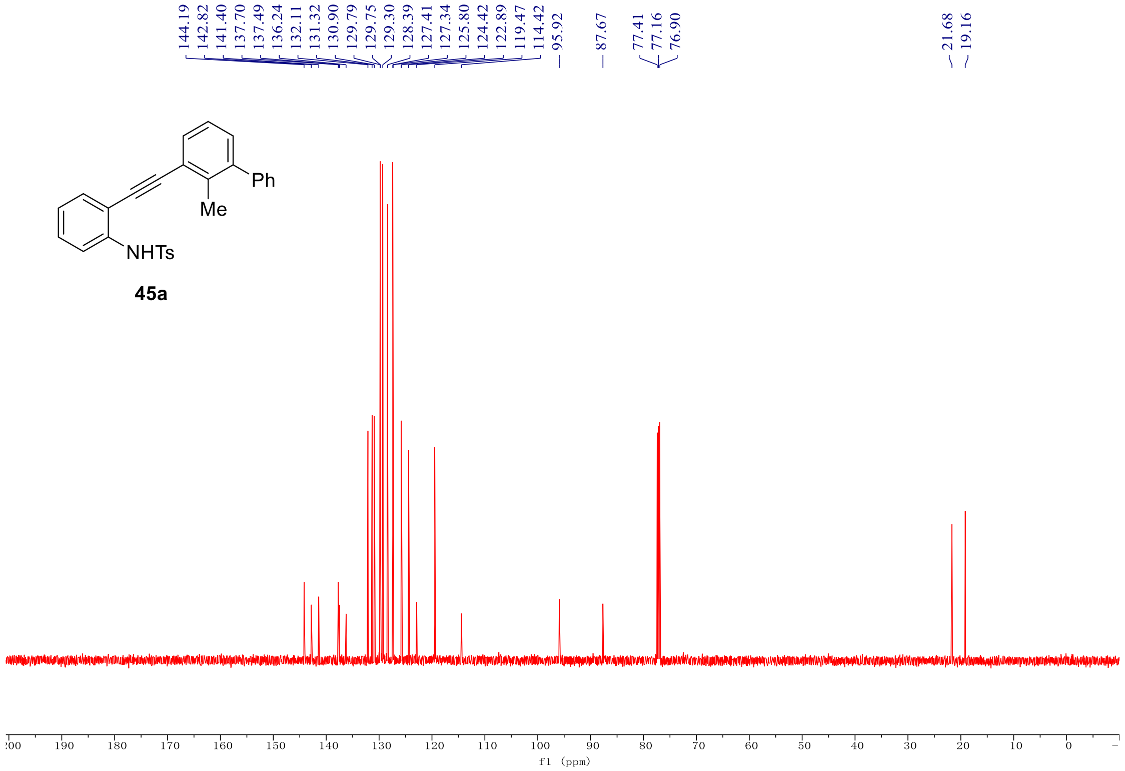

144.19
142.82
141.40
137.70
137.49
136.24
132.11
131.32
130.90
129.79
129.75
129.30
128.39
127.41
127.34
125.80
124.42
122.89
119.47
114.42
95.92
87.67
77.41
77.16
76.90
21.68
19.16
Ph
Me
NHTs
45a
190
180
170
160
150
140
130
120
110
100
90
80
70
60
50
40
30
20
10
0
f1 (ppm)

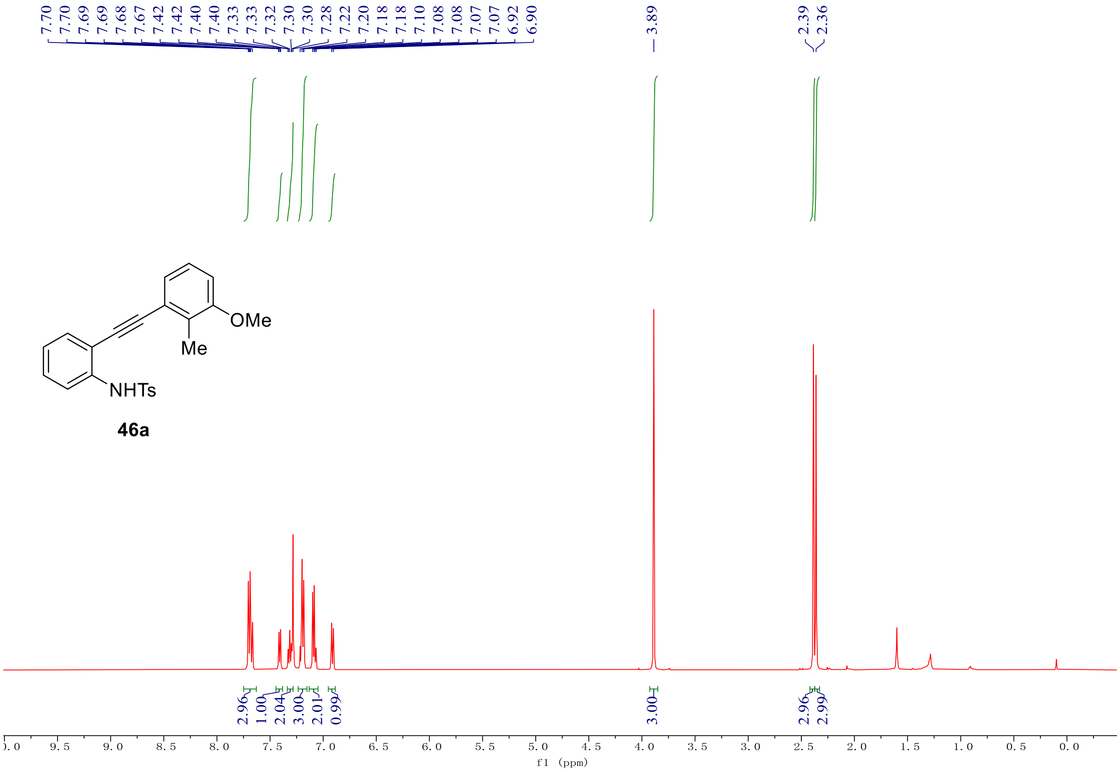
7.70
7.70
7.69
7.69
7.68
7.67
7.42
7.42
7.40
7.40
7.33
7.33
7.32
7.30
7.30
7.28
7.22
7.20
7.18
7.18
7.10
7.08
7.08
7.07
7.07
6.92
6.90
3.89
2.39
2.36
OMe
Me
NHTs
46a
2.96
1.00
2.04
3.00
2.01
0.99
3.00
2.96
2.99
f1 (ppm)

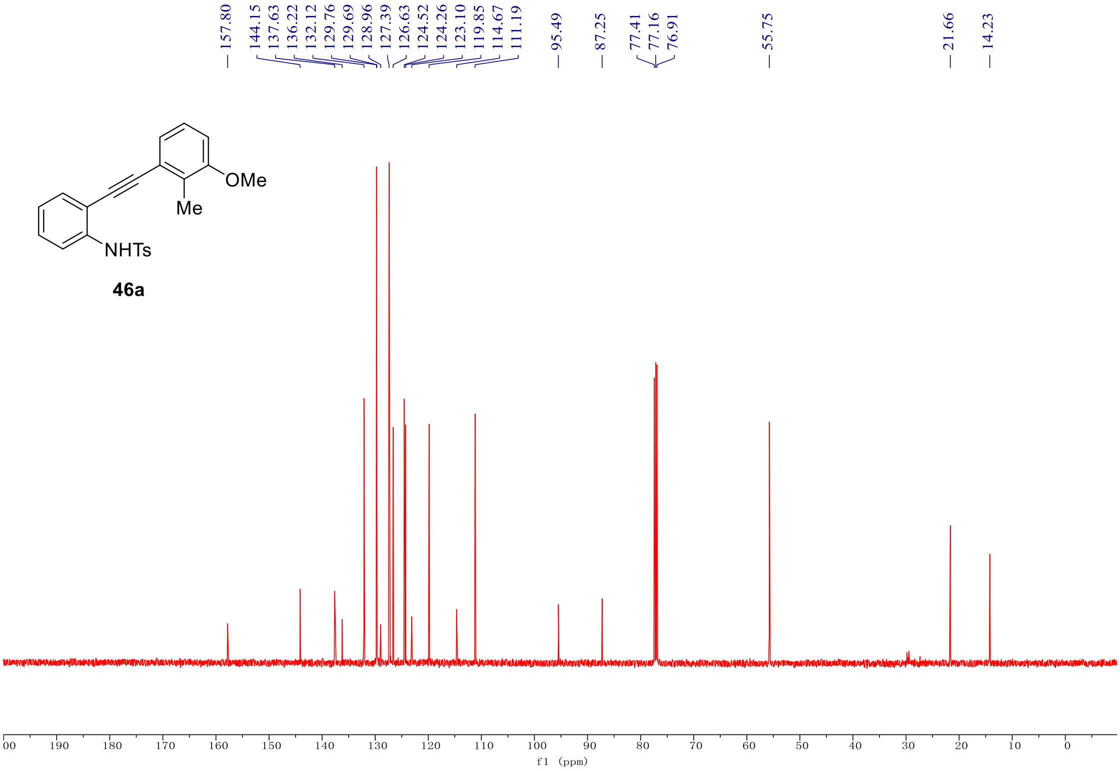
157.80
144.15
137.63
136.22
132.12
129.76
129.69
128.96
127.39
126.63
124.52
124.26
123.10
119.85
114.67
111.19
95.49
87.25
77.41
77.16
76.91
55.75
21.66
14.23
OMe
Me
NHTs
46a
f1 (ppm)

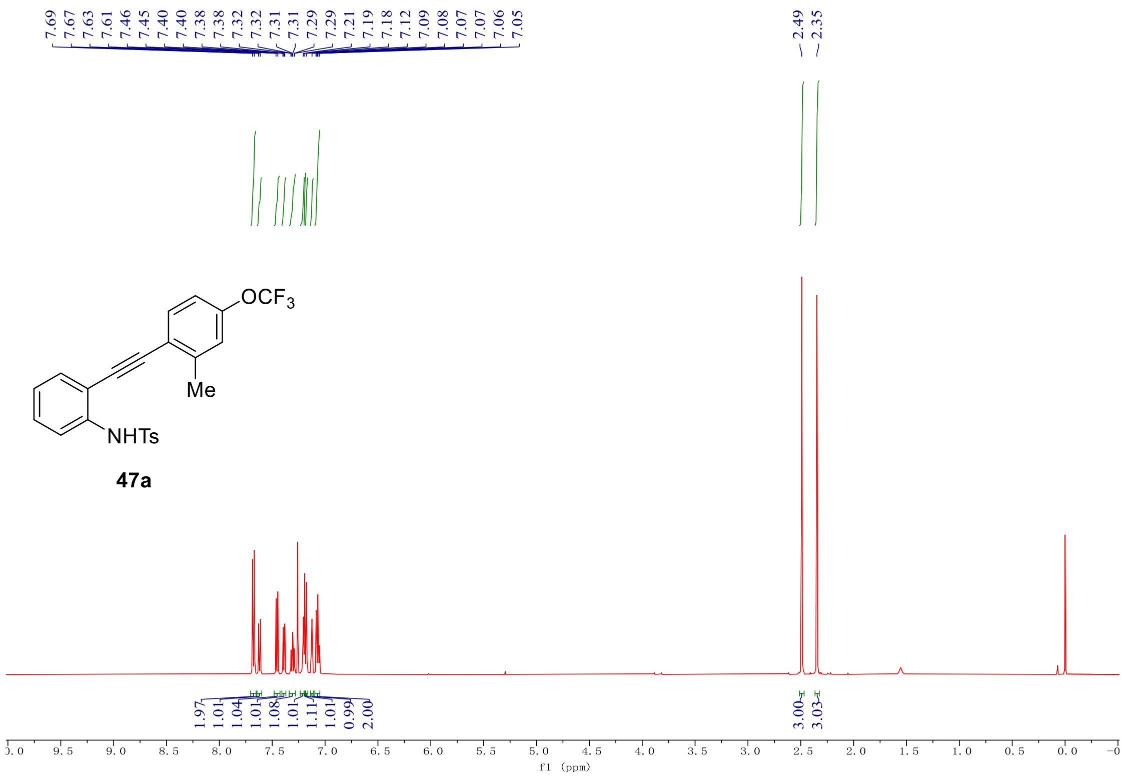
7.69
7.67
7.63
7.61
7.46
7.45
7.40
7.40
7.38
7.38
7.32
7.32
7.31
7.31
7.29
7.29
7.21
7.19
7.18
7.12
7.09
7.08
7.07
7.07
7.06
7.05
2.49
2.35
OCF3
Me
NHTs
47a
1.97
1.01
1.04
1.01
1.08
1.01
1.11
1.01
0.99
2.00
3.00
3.03
f1 (ppm)

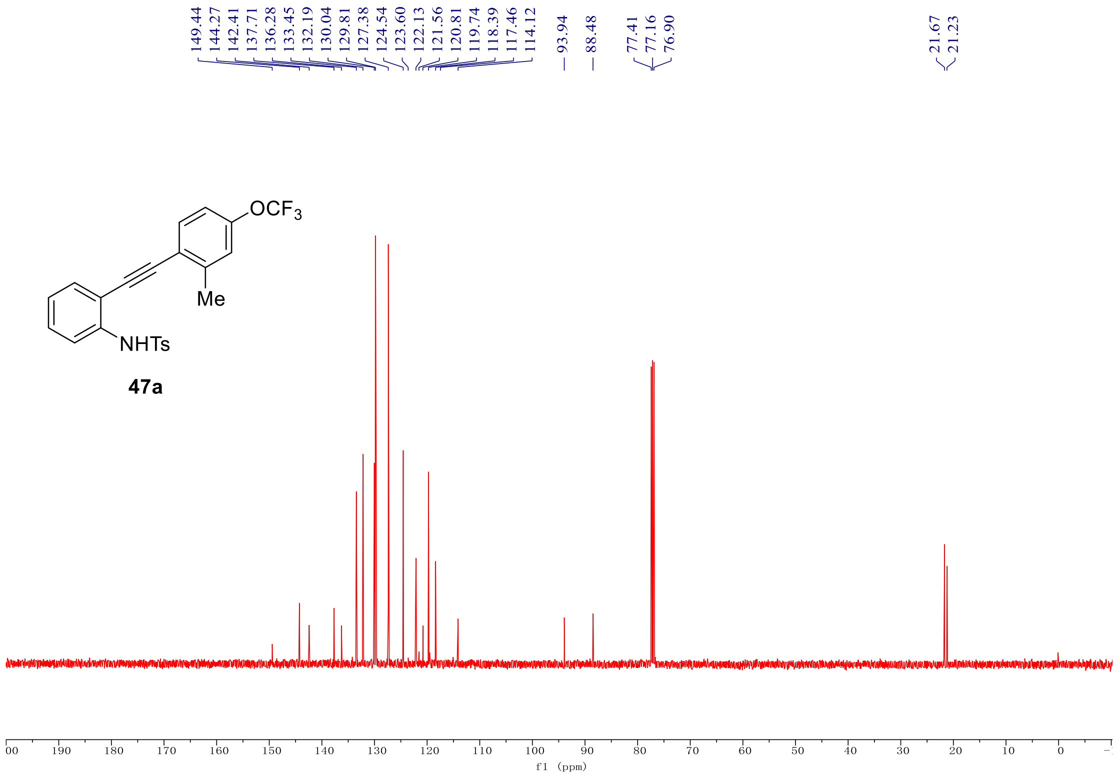
149.44
144.27
142.41
137.71
136.28
133.45
132.19
130.04
129.81
127.38
124.54
123.60
122.13
121.56
120.81
119.74
118.39
117.46
114.12
93.94
88.48
77.41
77.16
76.90
21.67
21.23
OCF3
Me
NHTs
47a
f1 (ppm)

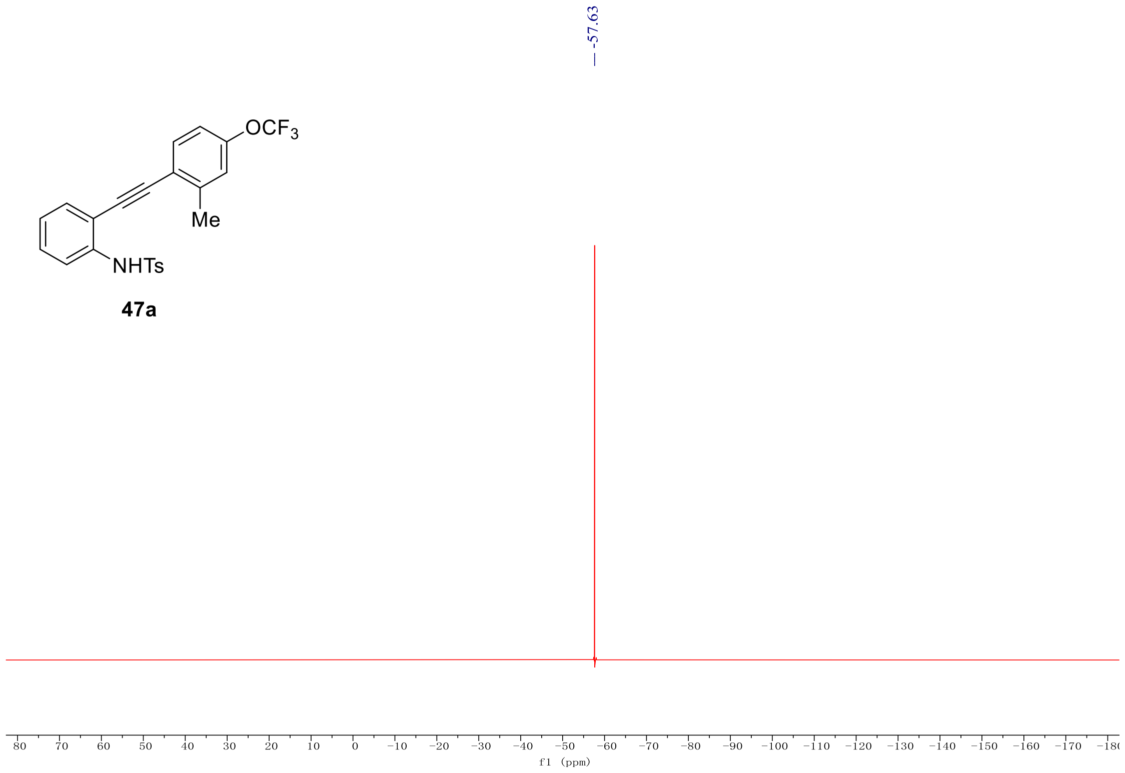
-57.63
OCF3
Me
NHTs
47a
80 70 60 50 40 30 20 10 0 -10 -20 -30 -40 -50 -60 -70 -80 -90 -100 -110 -120 -130 -140 -150 -160 -170 -180
f1 (ppm)

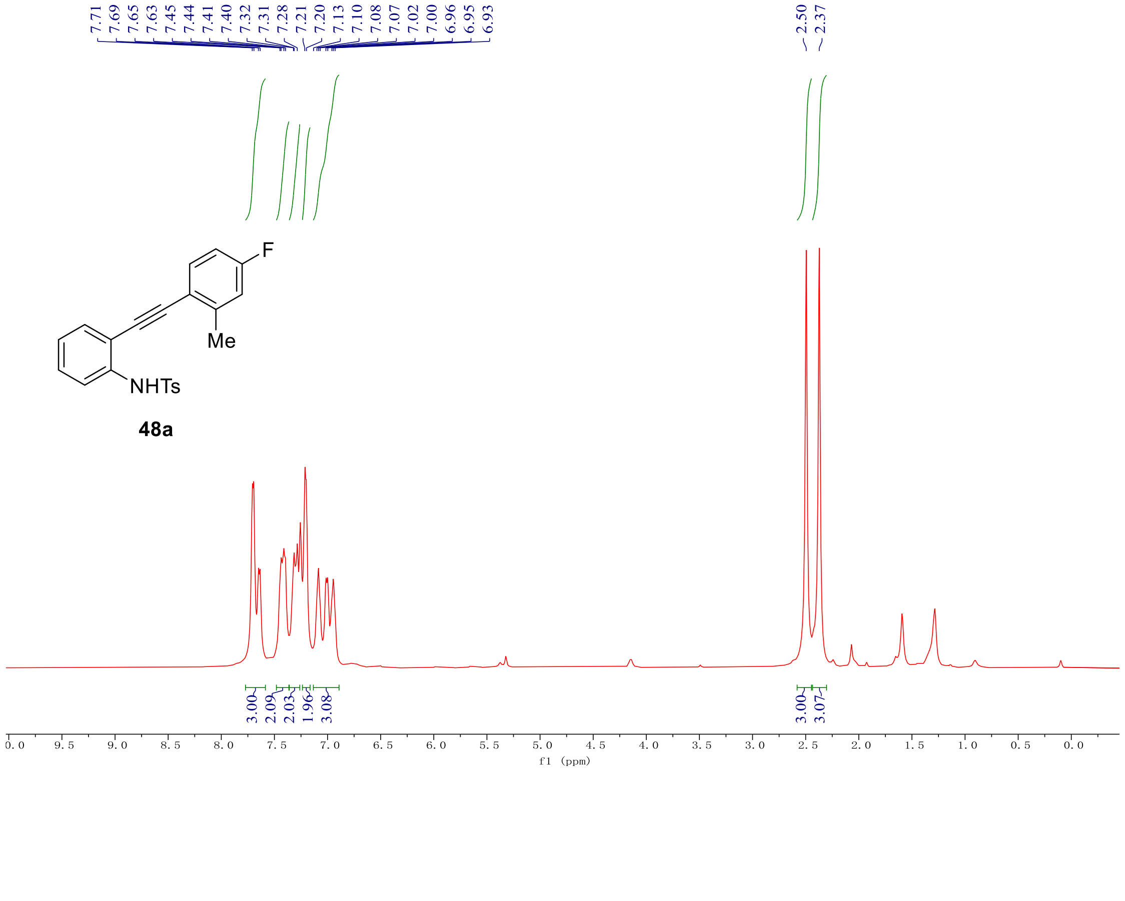
7.71 7.69 7.65 7.63 7.45 7.44 7.41 7.40 7.32 7.31 7.28 7.21 7.20 7.13 7.10 7.08 7.07 7.02 7.00 6.96 6.95 6.93
2.50 2.37
F
Me
NHTs
48a
3.00 2.09 2.03 1.96 3.08
3.00 3.07
0.0 9.5 9.0 8.5 8.0 7.5 7.0 6.5 6.0 5.5 5.0 4.5 4.0 3.5 3.0 2.5 2.0 1.5 1.0 0.5 0.0
f1 (ppm)

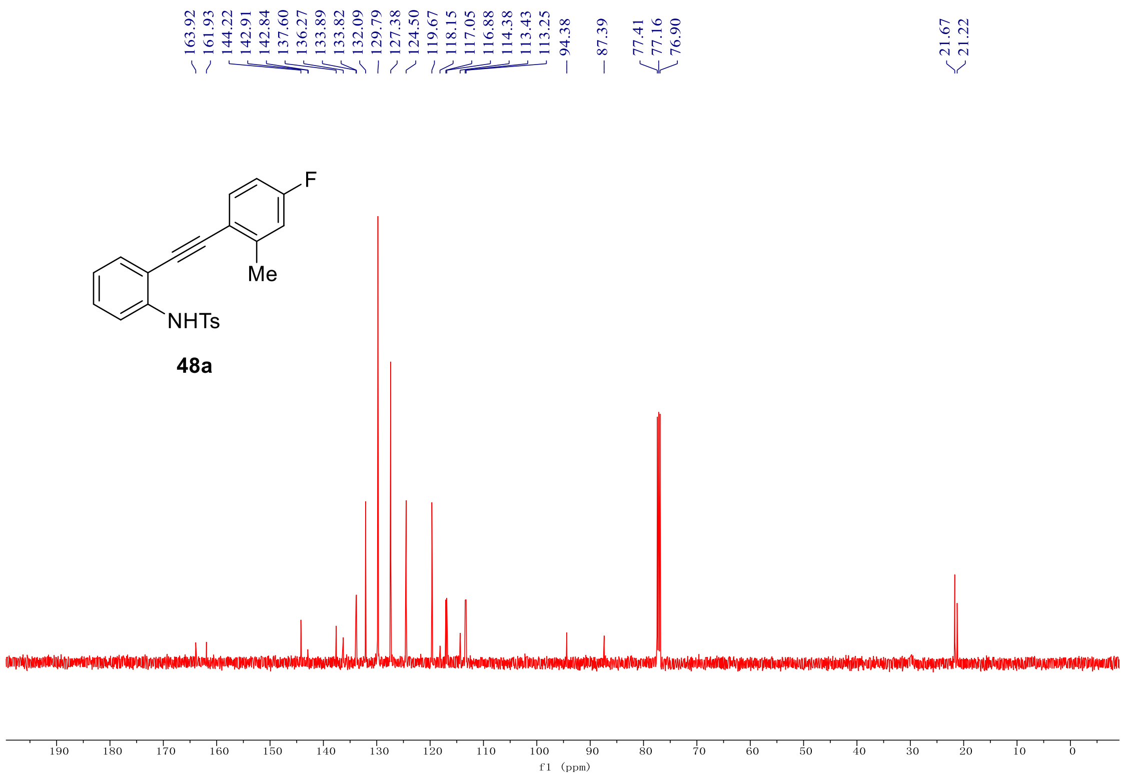
163.92
161.93
144.22
142.91
142.84
137.60
136.27
133.89
133.82
132.09
129.79
127.38
124.50
119.67
118.15
117.05
116.88
114.38
113.43
113.25
94.38
87.39
77.41
77.16
76.90
21.67
21.22
F
Me
NHTs
48a
190
180
170
160
150
140
130
120
110
100
90
80
70
60
50
40
30
20
10
0
f1 (ppm)

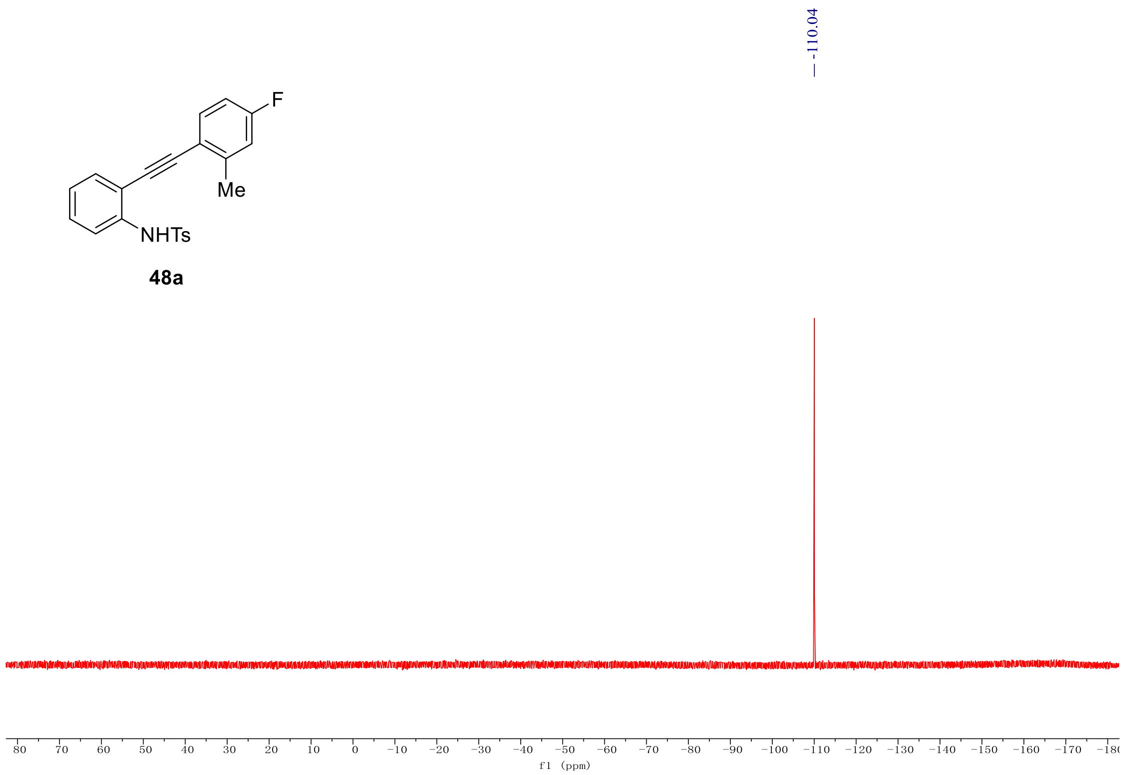
-110.04
F
Me
NHTs
48a
80
70
60
50
40
30
20
10
0
-10
-20
-30
-40
-50
-60
-70
-80
-90
-100
-110
-120
-130
-140
-150
-160
-170
f1 (ppm)

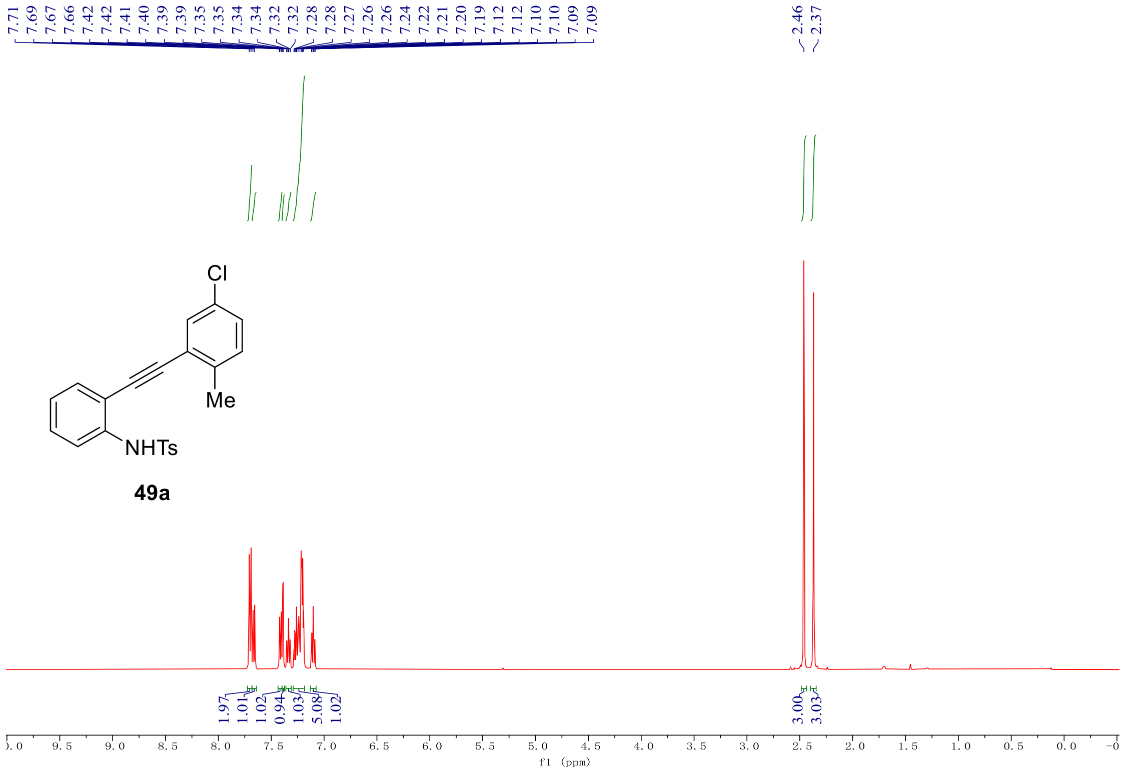

7.71
7.69
7.67
7.66
7.42
7.42
7.41
7.40
7.39
7.39
7.35
7.35
7.34
7.34
7.32
7.32
7.28
7.27
7.26
7.26
7.24
7.22
7.21
7.20
7.19
7.12
7.12
7.10
7.10
7.09
7.09
2.46
2.37
Cl
Me
NHTs
49a
1.97
1.01
1.02
0.94
1.03
5.08
1.02
3.00
3.03
f1 (ppm)

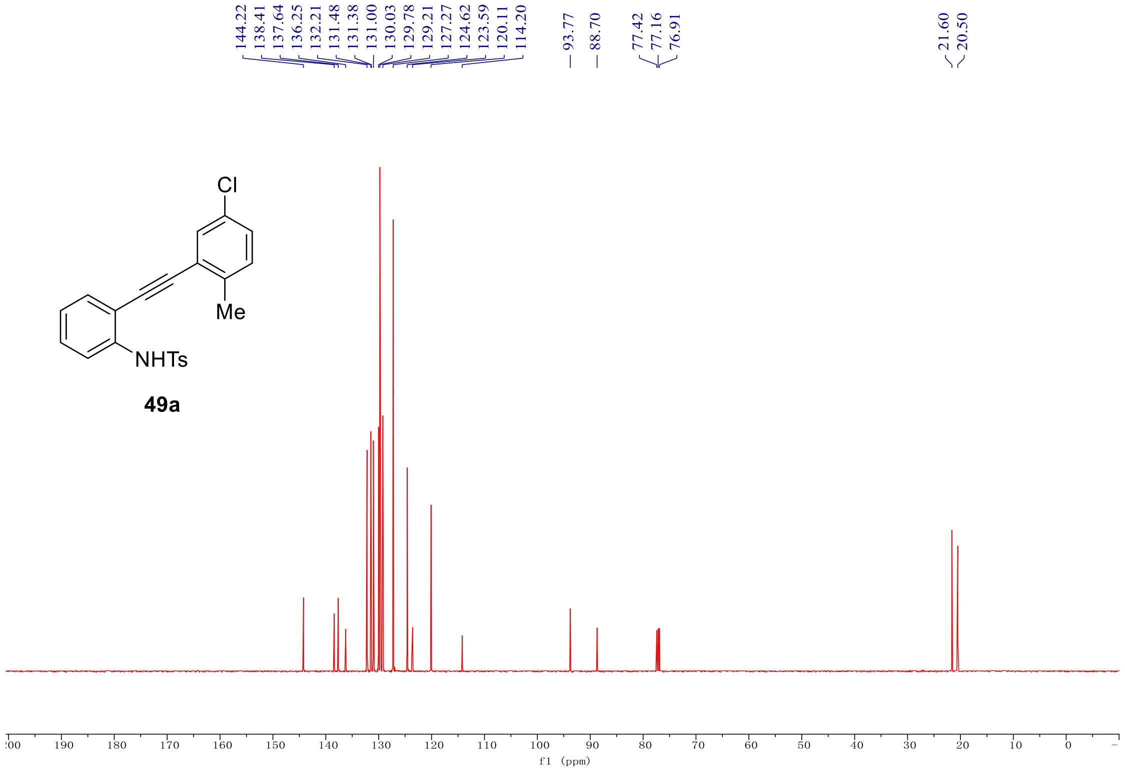

144.22
138.41
137.64
136.25
132.21
131.48
131.38
131.00
130.03
129.78
129.21
127.27
124.62
123.59
120.11
114.20
93.77
88.70
77.42
77.16
76.91
21.60
20.50
Cl
Me
NHTs
49a
f1 (ppm)

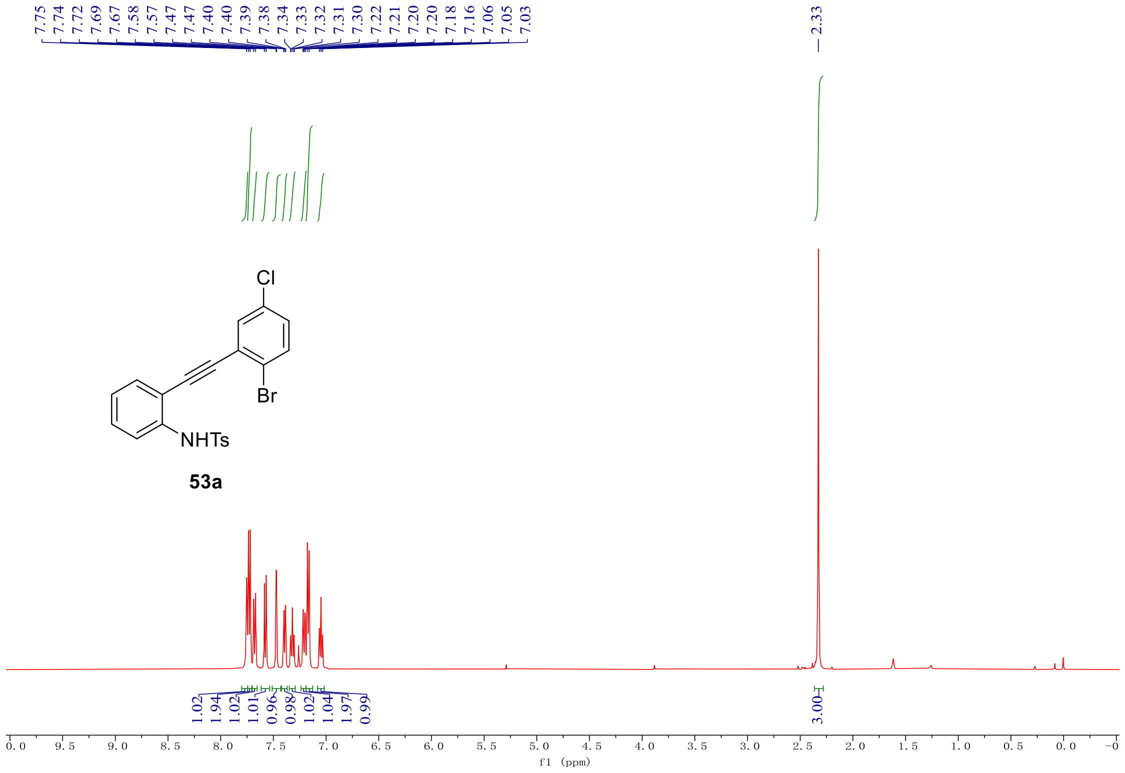
7.75 7.74 7.72 7.69 7.67 7.58 7.57 7.47 7.47 7.40 7.40 7.39 7.38 7.34 7.33 7.32 7.31 7.30 7.22 7.21 7.20 7.20 7.18 7.16 7.06 7.05 7.03
2.33
Cl
Br
NHTs
53a
1.02 1.94 1.02 1.01 0.96 0.98 1.02 1.04 1.97 0.99
3.00
f1 (ppm)

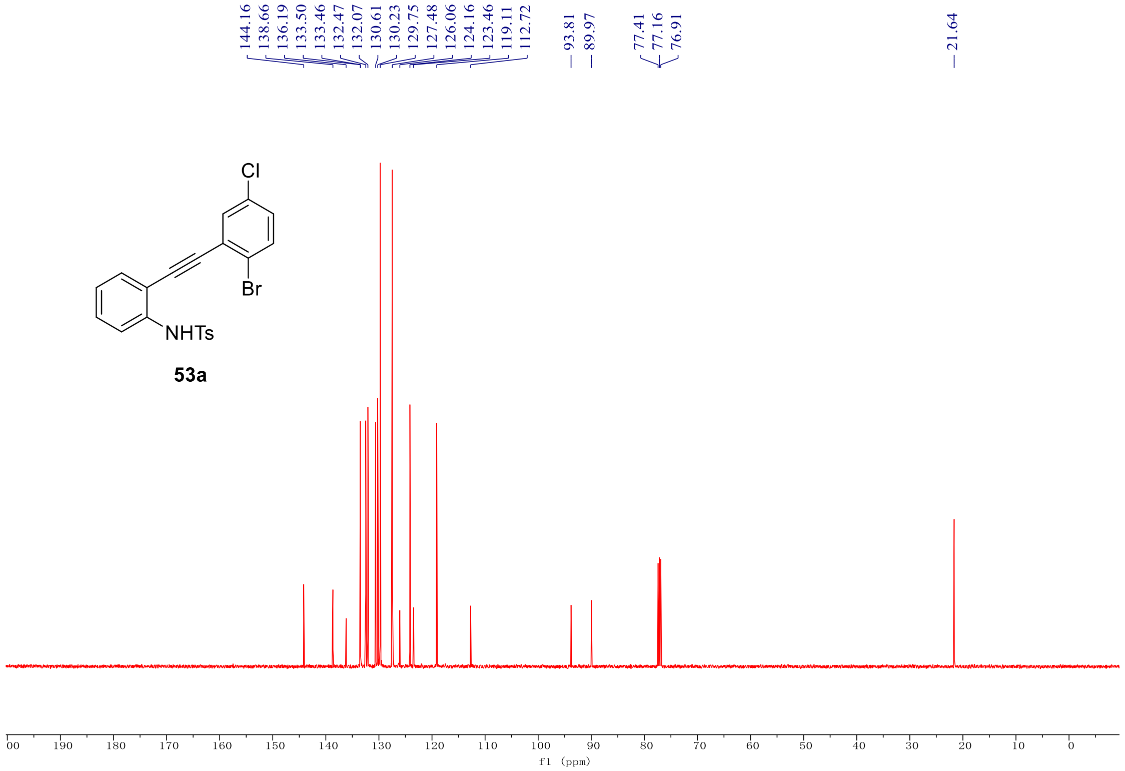
144.16 138.66 136.19 133.50 133.46 132.47 132.07 130.61 130.23 129.75 127.48 126.06 124.16 123.46 119.11 112.72
93.81 89.97
77.41 77.16 76.91
21.64
Cl
Br
NHTs
53a
f1 (ppm)

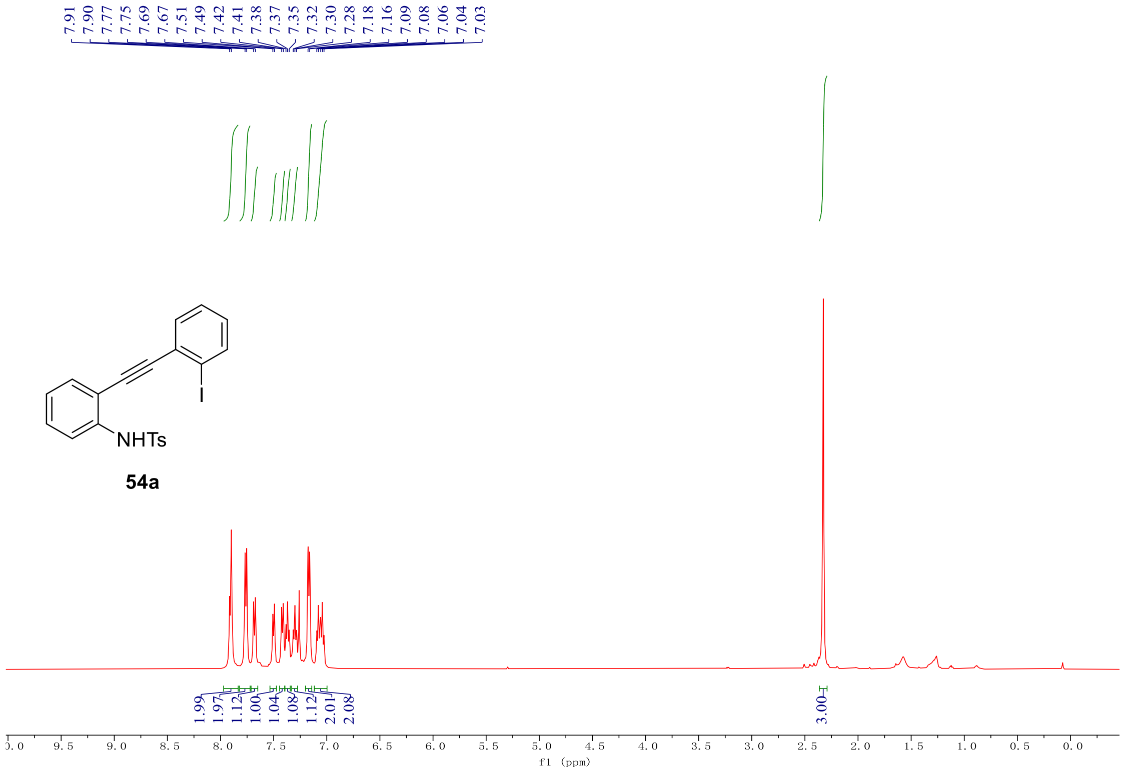
7.91
7.90
7.77
7.75
7.69
7.67
7.51
7.49
7.42
7.41
7.38
7.37
7.35
7.32
7.30
7.28
7.18
7.16
7.09
7.08
7.06
7.04
7.03
NHTs
54a
1.99
1.97
1.12
1.00
1.04
1.08
1.12
2.01
2.08
3.00
f1 (ppm)

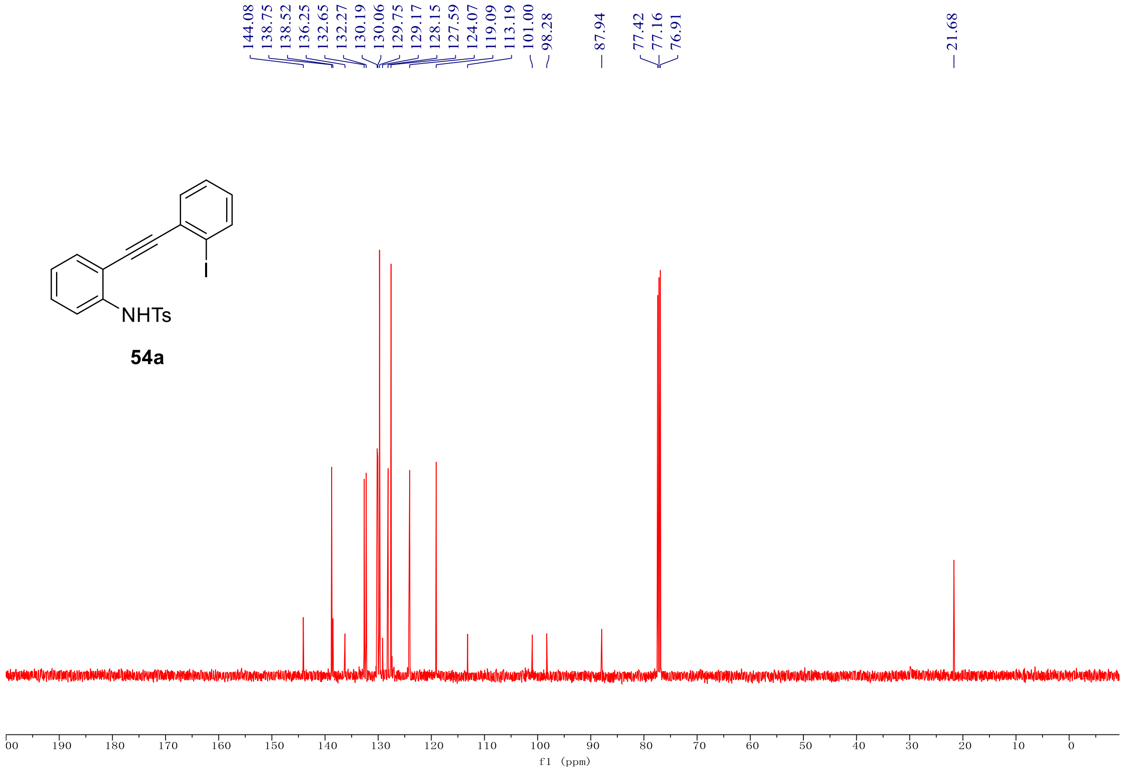
144.08
138.75
138.52
136.25
132.65
132.27
130.19
130.06
129.75
129.17
128.15
127.59
124.07
119.09
113.19
101.00
98.28
87.94
77.42
77.16
76.91
21.68
NHTs
54a
f1 (ppm)

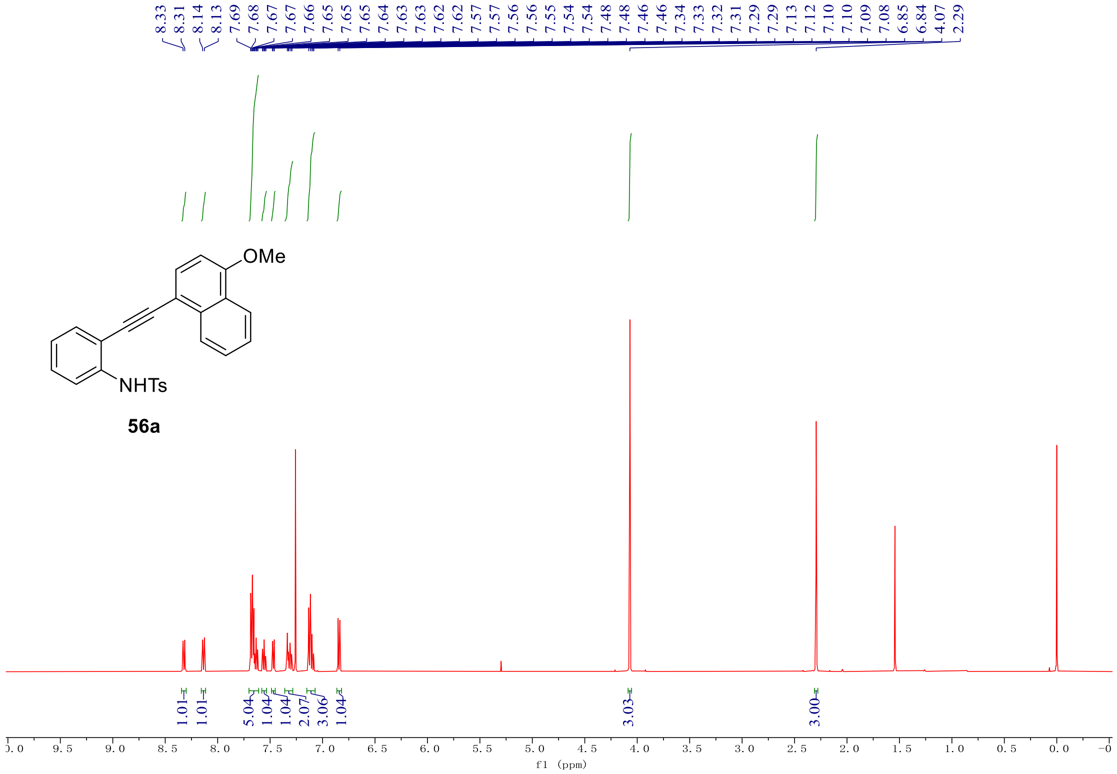

8.33
8.31
8.14
8.13
7.69
7.68
7.67
7.67
7.66
7.65
7.65
7.64
7.63
7.63
7.62
7.62
7.57
7.56
7.56
7.55
7.54
7.54
7.48
7.48
7.46
7.34
7.33
7.32
7.31
7.29
7.29
7.13
7.12
7.10
7.10
7.09
7.08
6.85
6.84
4.07
2.29
OMe
NHTs
56a
1.01
1.01
5.04
1.04
1.04
2.07
3.06
1.04
3.03
3.00
f1 (ppm)

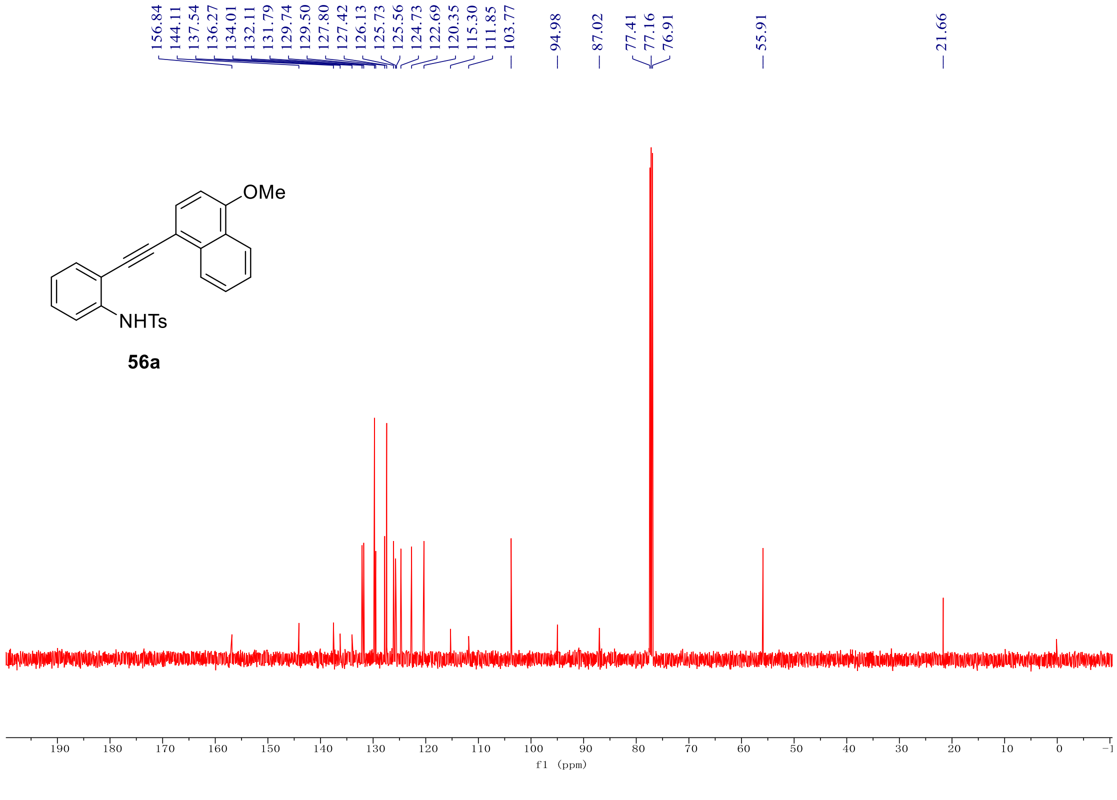

156.84
144.11
137.54
136.27
134.01
132.11
131.79
129.74
129.50
127.80
127.42
126.13
125.73
125.56
124.73
122.69
120.35
115.30
111.85
103.77
94.98
87.02
77.41
77.16
76.91
55.91
21.66
OMe
NHTs
56a
f1 (ppm)

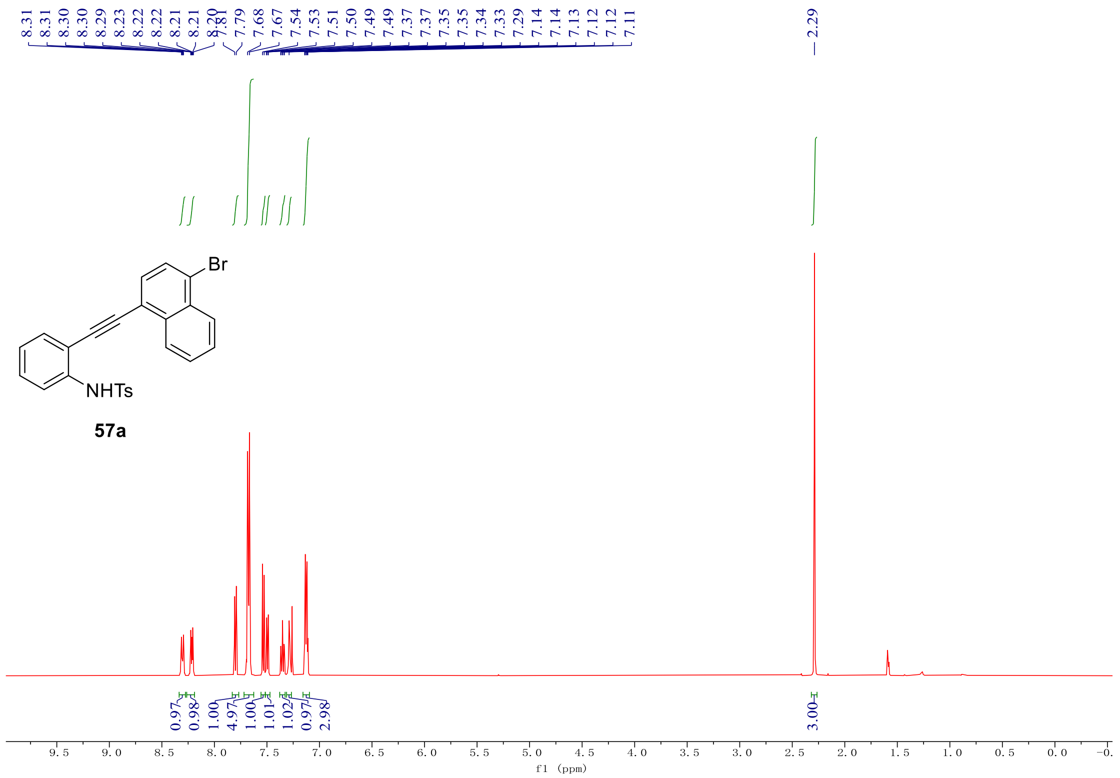
8.31
8.31
8.30
8.30
8.29
8.23
8.22
8.22
8.21
8.21
8.20
7.81
7.79
7.68
7.67
7.54
7.53
7.51
7.50
7.49
7.49
7.37
7.37
7.35
7.35
7.34
7.33
7.29
7.14
7.14
7.13
7.12
7.12
7.11
2.29
Br
NHTs
57a
0.97
0.98
1.00
4.97
1.00
1.01
1.02
0.97
2.98
3.00
f1 (ppm)

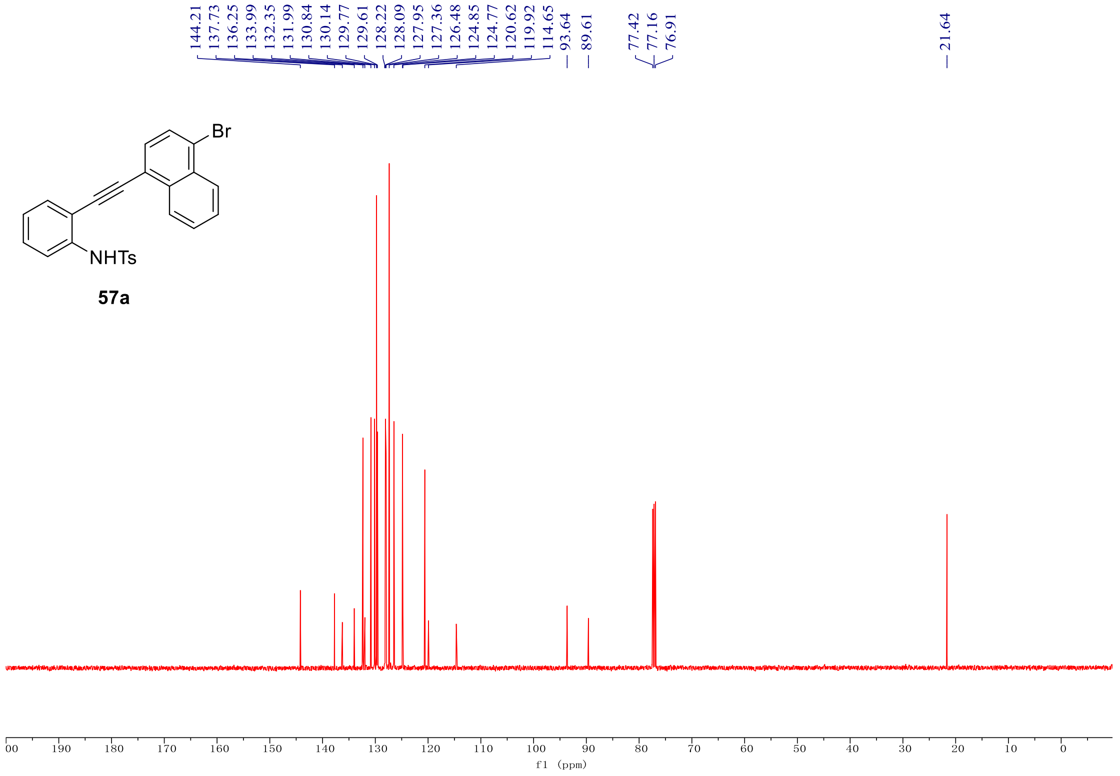
144.21
137.73
136.25
133.99
132.35
131.99
130.84
130.14
129.77
129.61
128.22
128.09
127.95
127.36
126.48
124.85
124.77
120.62
119.92
114.65
93.64
89.61
77.42
77.16
76.91
21.64
Br
NHTs
57a
f1 (ppm)

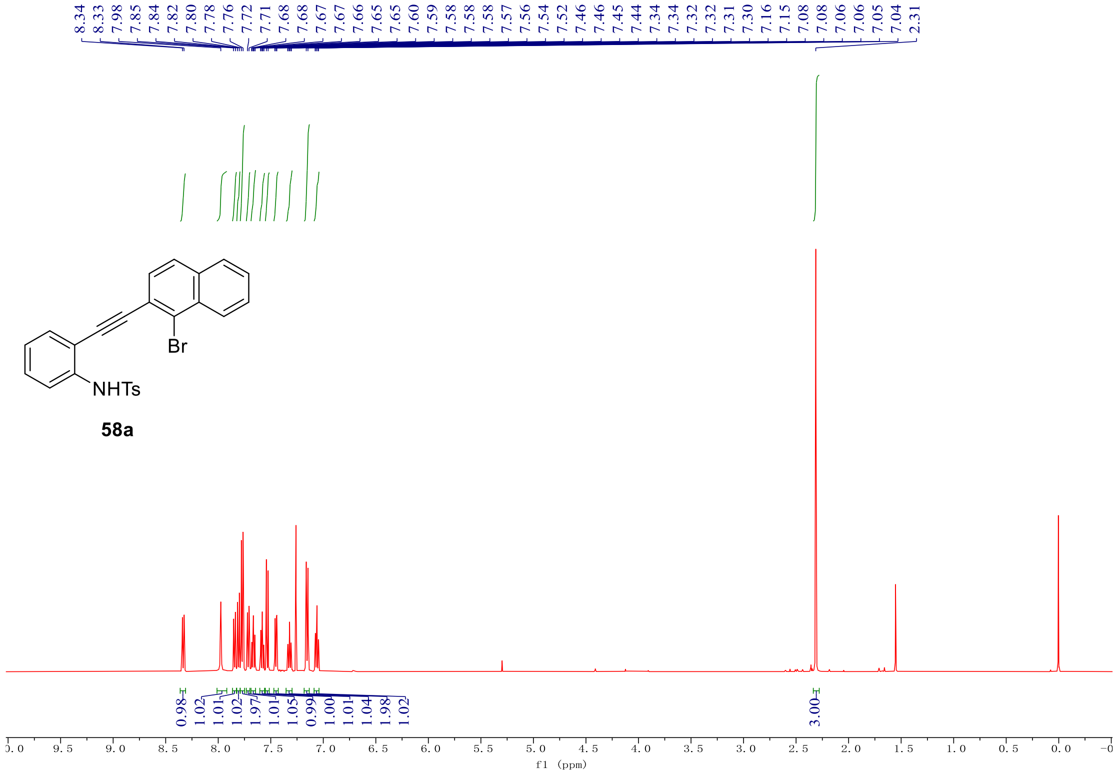
8.34
8.33
7.98
7.85
7.84
7.82
7.80
7.78
7.76
7.72
7.71
7.68
7.68
7.67
7.67
7.66
7.65
7.65
7.60
7.59
7.58
7.58
7.58
7.57
7.56
7.54
7.52
7.46
7.45
7.44
7.34
7.34
7.32
7.32
7.31
7.30
7.16
7.15
7.08
7.08
7.06
7.06
7.05
7.04
2.31
Br
NHTs
58a
0.98
1.02
1.01
1.02
1.97
1.01
1.01
1.05
0.99
1.00
1.01
1.04
1.98
1.02
3.00
f1 (ppm)
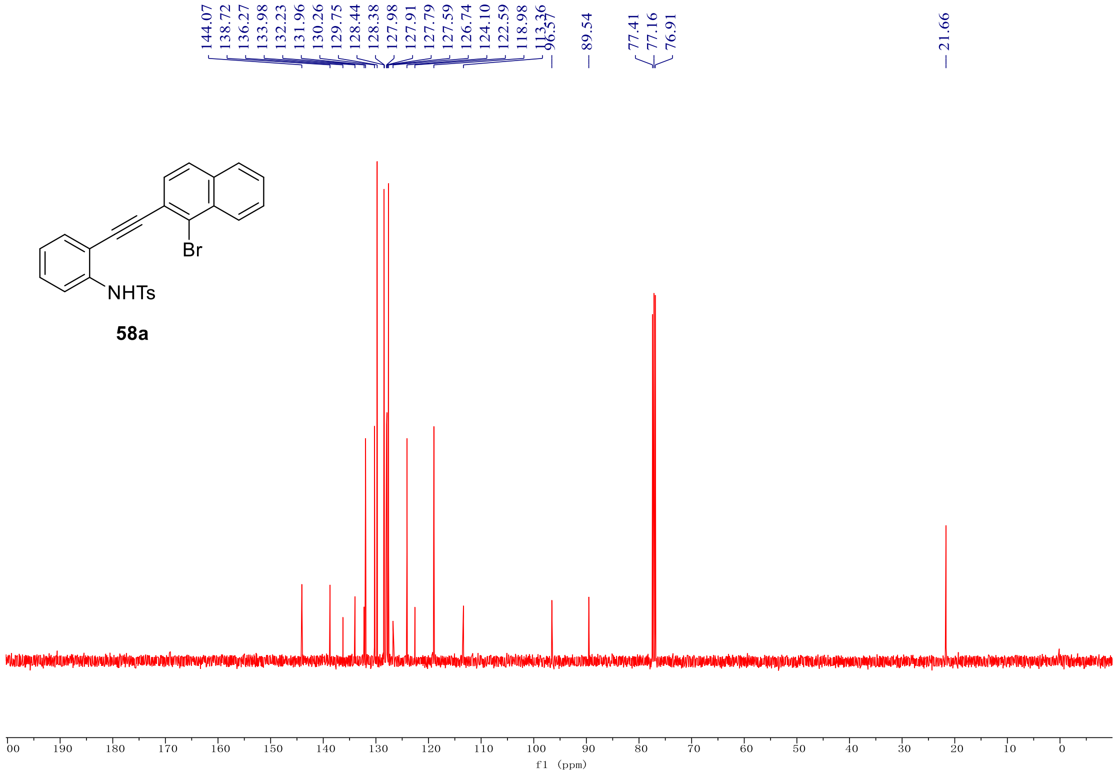
144.07
138.72
136.27
133.98
132.23
131.96
130.26
129.75
128.44
128.38
127.98
127.91
127.79
127.59
126.74
124.10
122.59
118.98
113.36
96.57
89.54
77.41
77.16
76.91
21.66
Br
NHTs
58a
f1 (ppm)

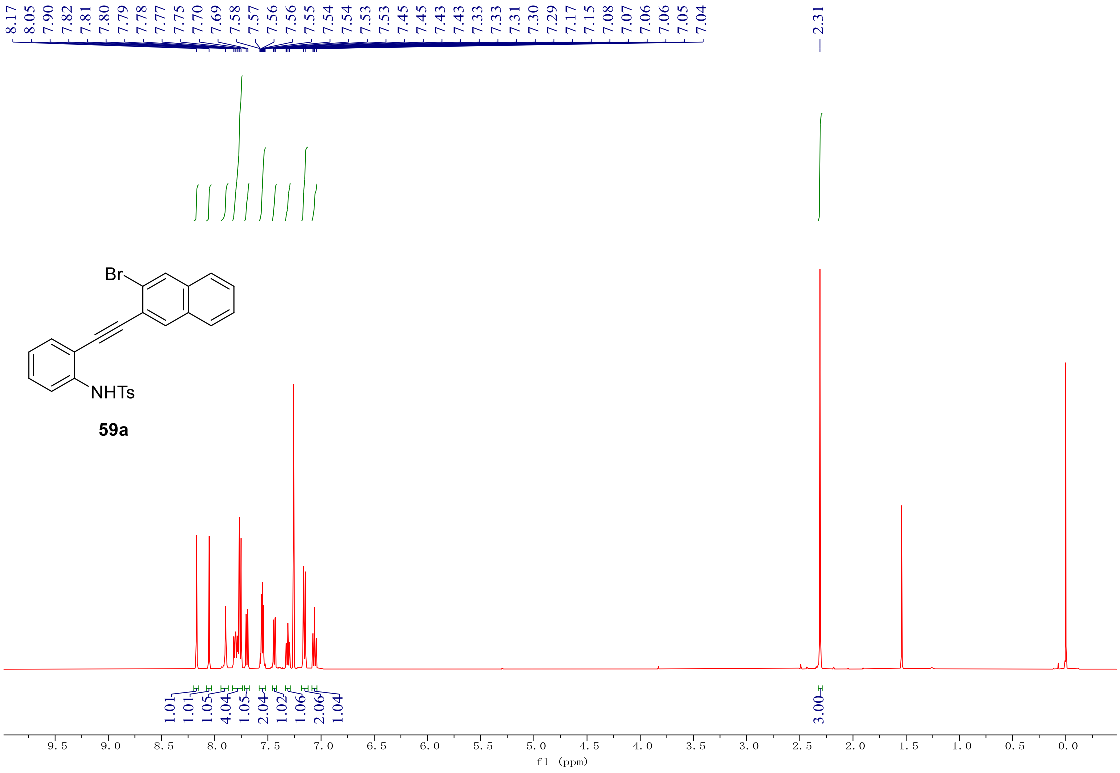
8.17
8.05
7.90
7.82
7.81
7.80
7.79
7.78
7.77
7.75
7.70
7.69
7.58
7.57
7.56
7.56
7.55
7.54
7.54
7.53
7.53
7.45
7.45
7.43
7.43
7.33
7.33
7.31
7.30
7.29
7.17
7.15
7.08
7.07
7.06
7.06
7.05
7.04
2.31
Br
NHTs
1.01
1.01
1.05
4.04
1.05
2.04
1.02
1.06
2.06
1.04
3.00
f1 (ppm)

59a

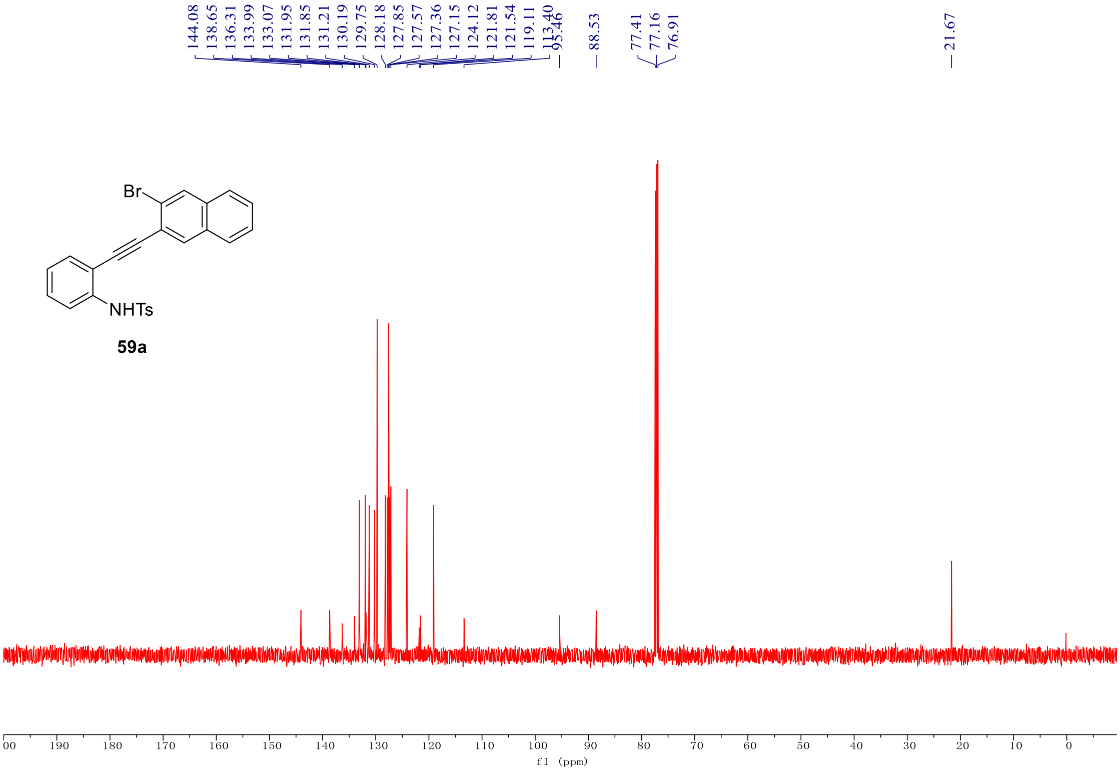
144.08
138.65
136.31
133.99
133.07
131.95
131.85
131.21
130.19
129.75
128.18
127.85
127.57
127.36
127.15
124.12
121.81
121.54
119.11
113.40
95.46
88.53
77.41
77.16
76.91
21.67
Br
NHTs
f1 (ppm)

59a

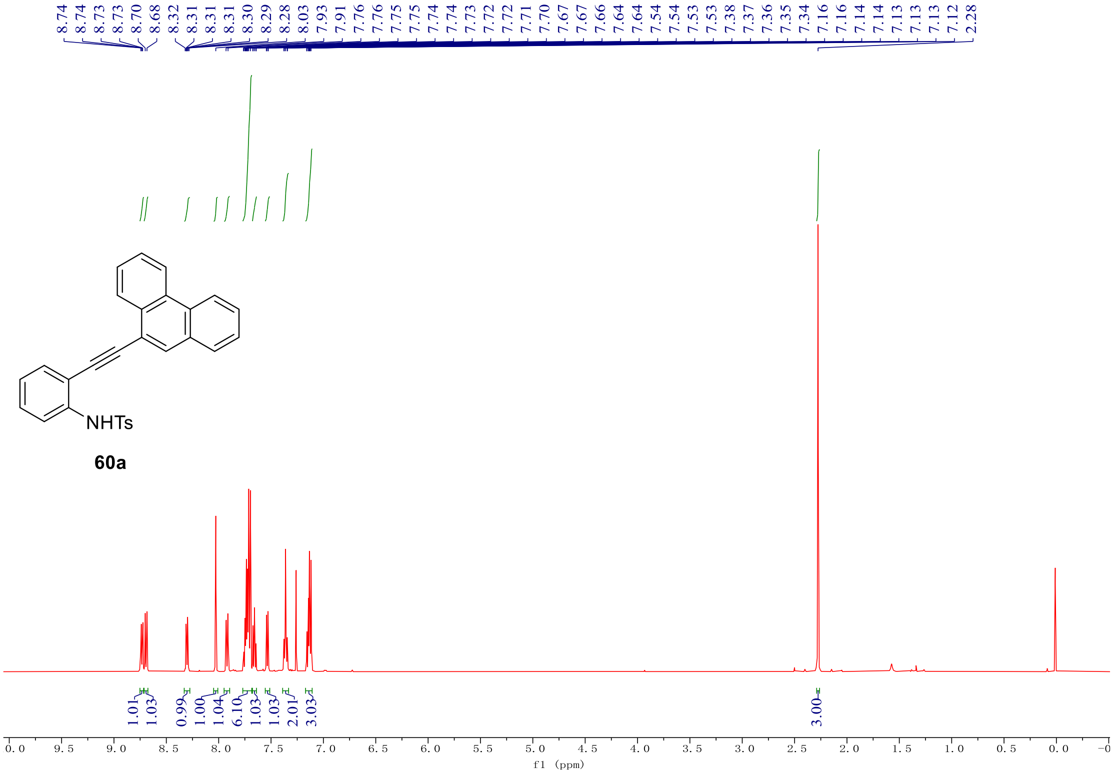
8.74 8.74 8.73 8.73 8.70 8.68 8.32 8.31 8.31 8.31 8.30 8.29 8.28 8.03 7.93 7.91 7.76 7.75 7.75 7.74 7.74 7.73 7.72 7.72 7.71 7.70 7.67 7.67 7.66 7.64 7.64 7.54 7.54 7.53 7.53 7.38 7.37 7.36 7.35 7.34 7.16 7.16 7.14 7.14 7.13 7.13 7.12 2.28
NHTs
60a
1.01 1.03 0.99 1.00 1.04 6.10 1.03 1.03 2.01 3.03 3.00
f1 (ppm)

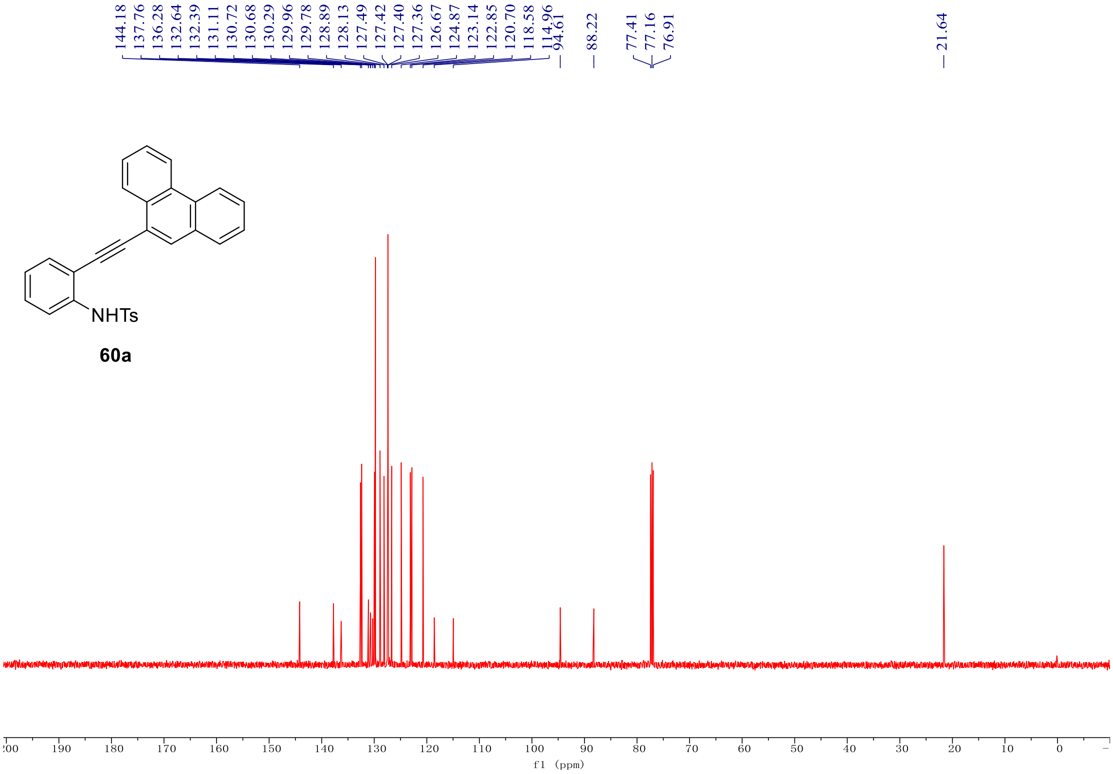
144.18 137.76 136.28 132.64 132.39 131.11 130.72 130.68 130.29 129.96 129.78 128.89 128.13 127.49 127.42 127.40 127.36 126.67 124.87 123.14 122.85 120.70 118.58 114.96 94.61 88.22 77.41 77.16 76.91 21.64
NHTs
60a
f1 (ppm)

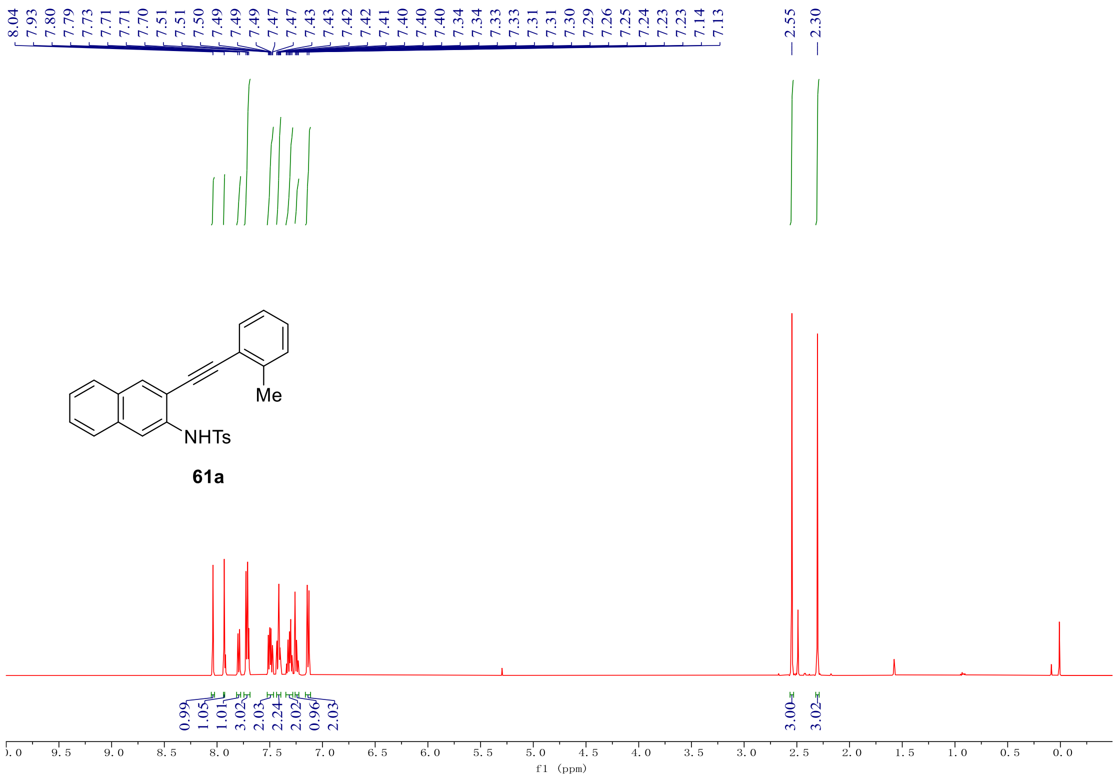
8.04
7.93
7.80
7.79
7.73
7.71
7.71
7.70
7.51
7.51
7.50
7.49
7.49
7.49
7.47
7.47
7.43
7.43
7.42
7.42
7.41
7.40
7.40
7.40
7.34
7.34
7.33
7.33
7.31
7.31
7.30
7.29
7.26
7.25
7.24
7.23
7.23
7.14
7.13
2.55
2.30
Me
NHTs
61a
0.99
1.05
1.01
3.02
2.03
2.24
2.02
0.96
2.03
3.00
3.02
f1 (ppm)

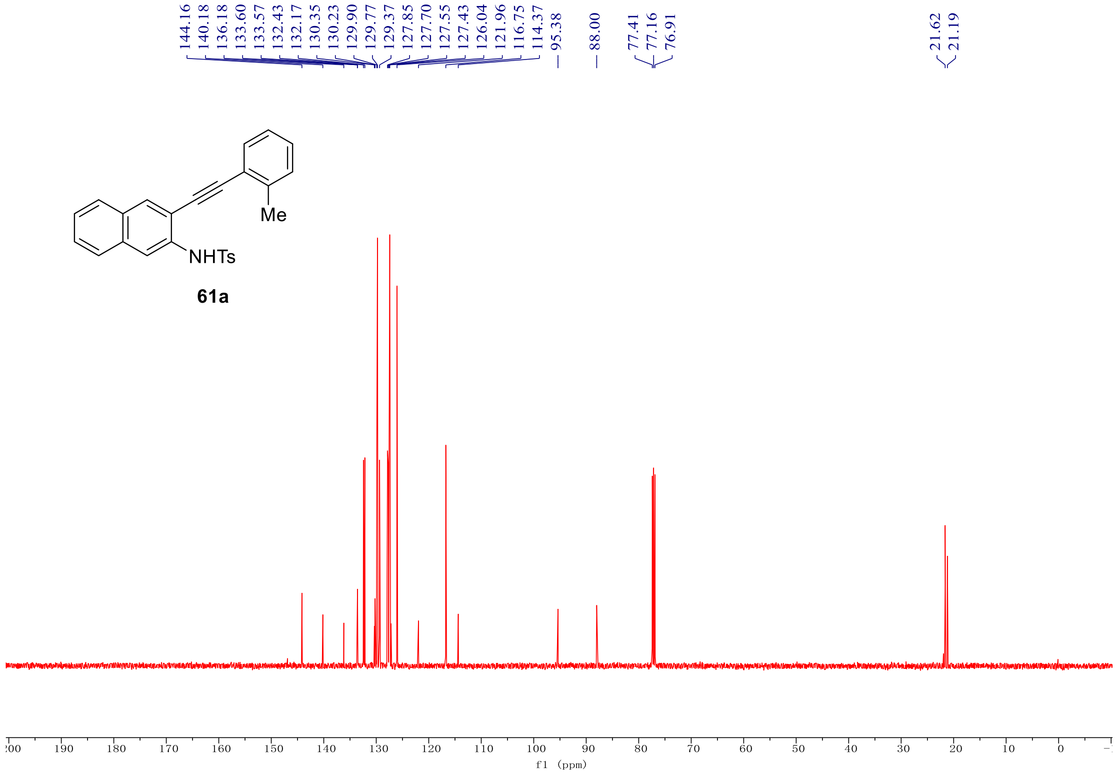
144.16
140.18
136.18
133.60
133.57
132.43
132.17
130.35
130.23
129.90
129.77
129.37
127.85
127.70
127.55
127.43
126.04
121.96
116.75
114.37
95.38
88.00
77.41
77.16
76.91
21.62
21.19
Me
NHTs
61a
f1 (ppm)

8.2 Sulfinamides

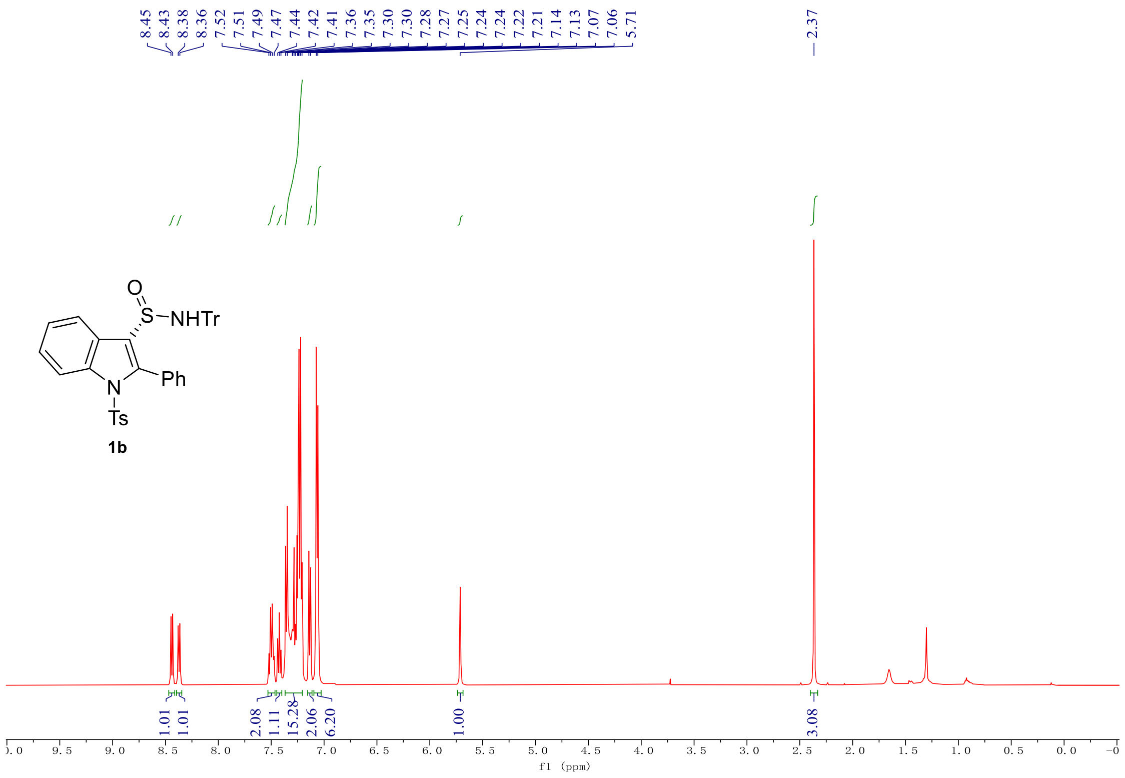

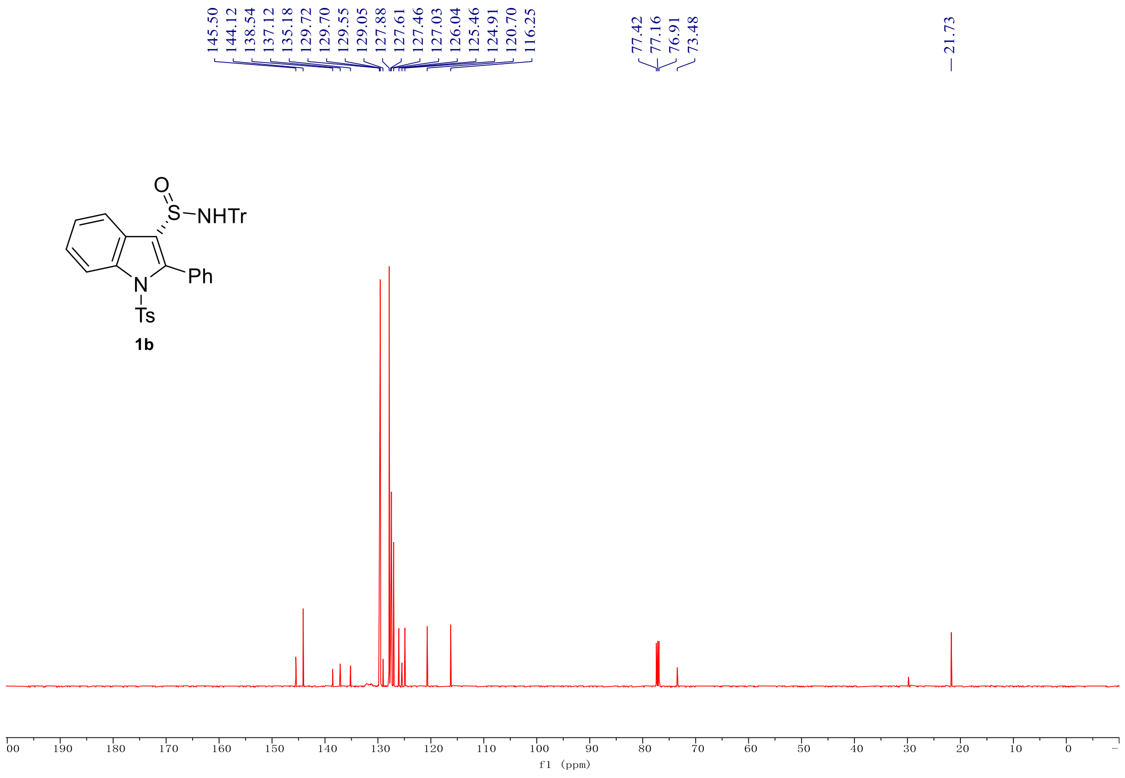

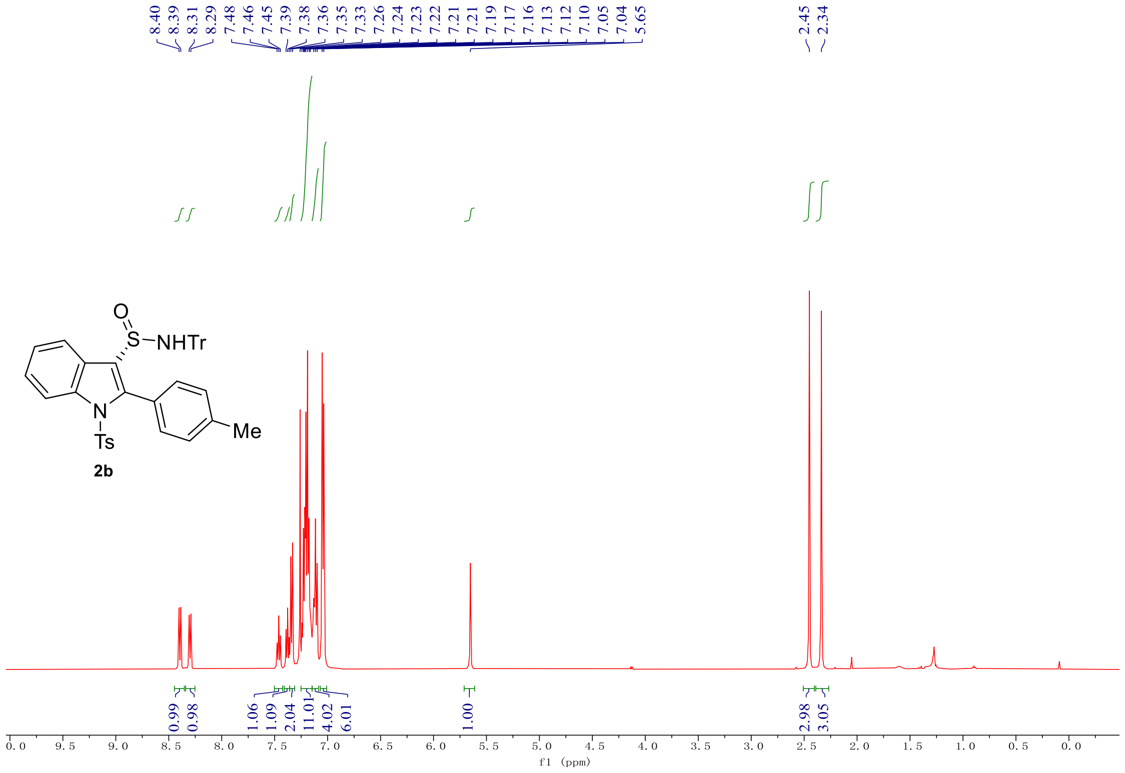
8.40
8.39
8.31
8.29
7.48
7.46
7.45
7.39
7.38
7.36
7.35
7.33
7.26
7.24
7.23
7.22
7.21
7.21
7.19
7.17
7.16
7.13
7.12
7.10
7.05
7.04
5.65
2.45
2.34
O
S
NHTr
N
Ts
Me
2b
0.99
0.98
1.06
1.09
2.04
11.01
4.02
6.01
1.00
2.98
3.05
f1 (ppm)

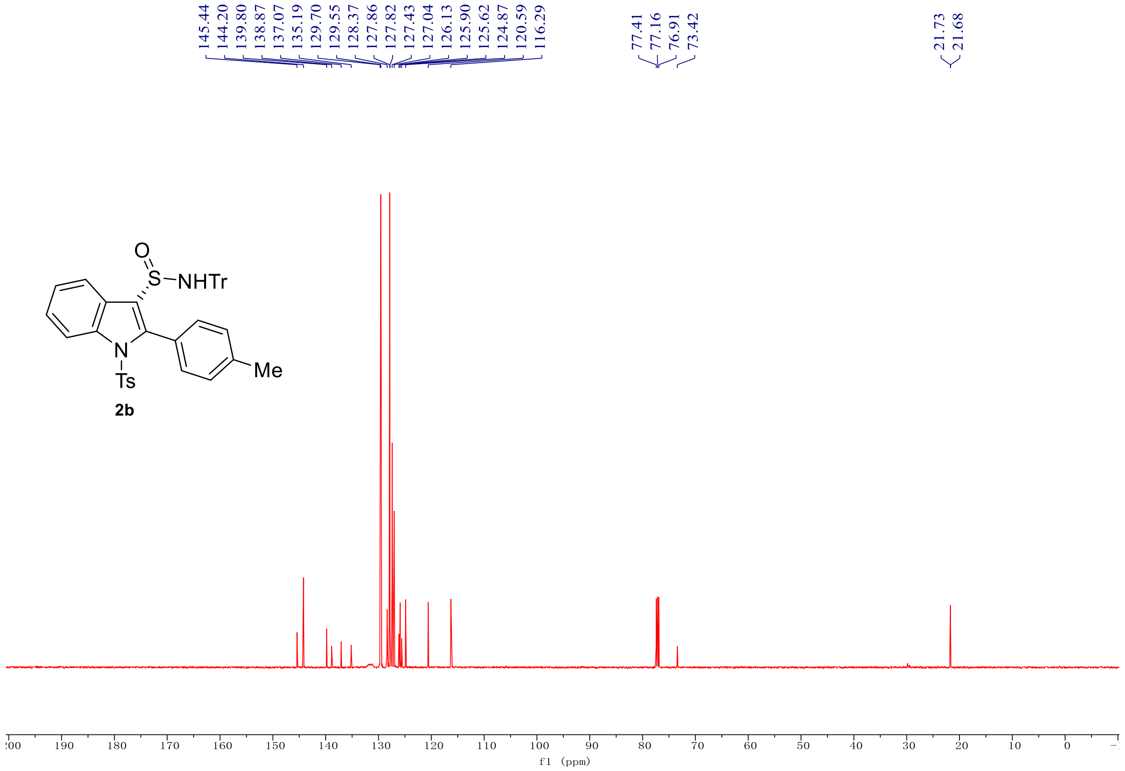
145.44
144.20
139.80
138.87
137.07
135.19
129.70
129.55
128.37
127.86
127.82
127.43
127.04
126.13
125.90
125.62
124.87
120.59
116.29
77.41
77.16
76.91
73.42
21.73
21.68
O
S
NHTr
N
Ts
Me
2b
f1 (ppm)

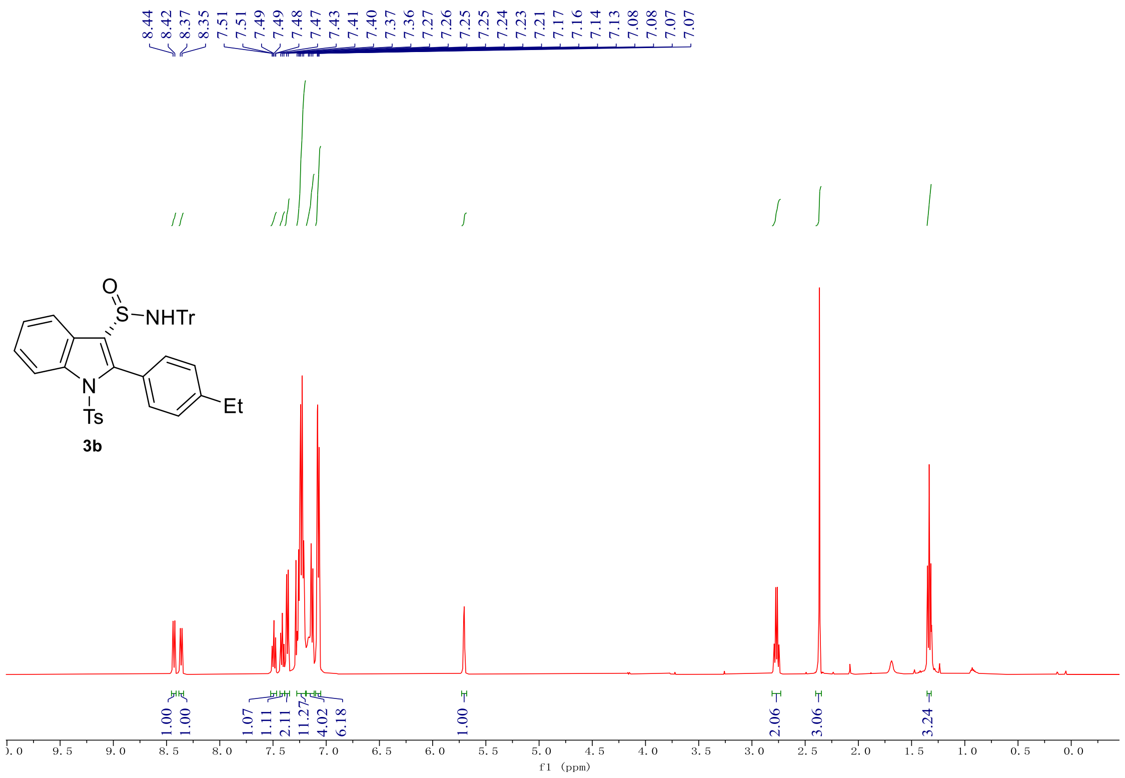

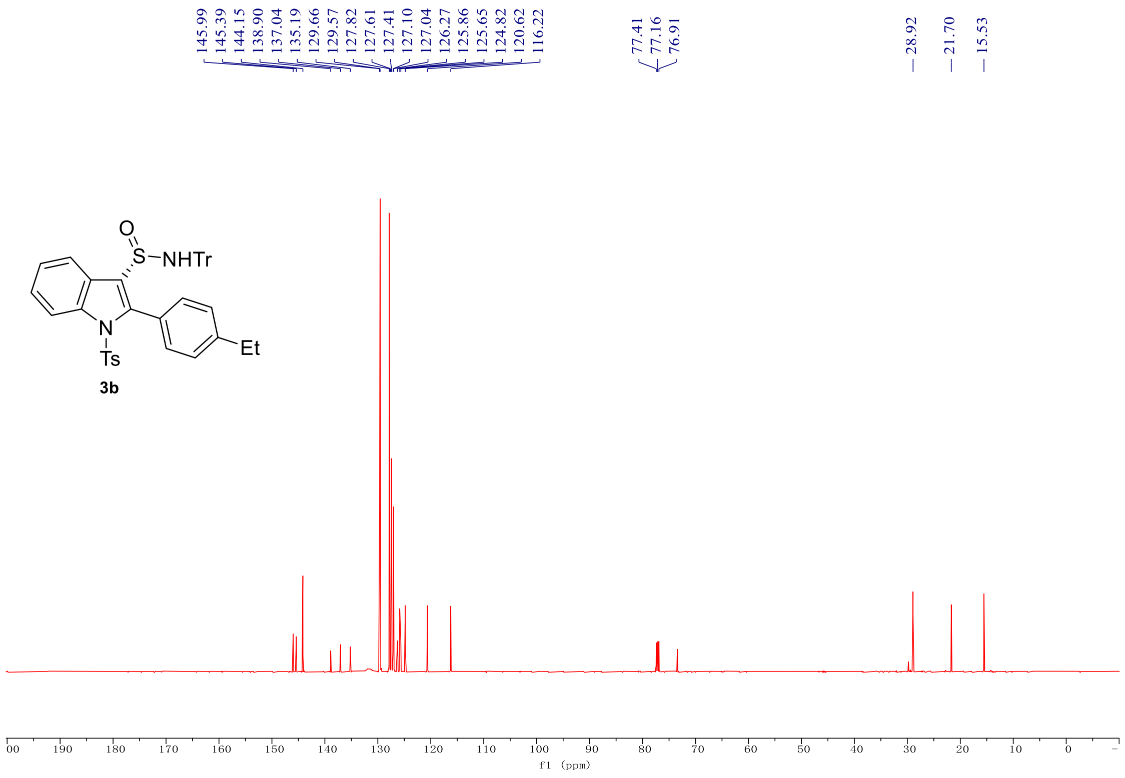

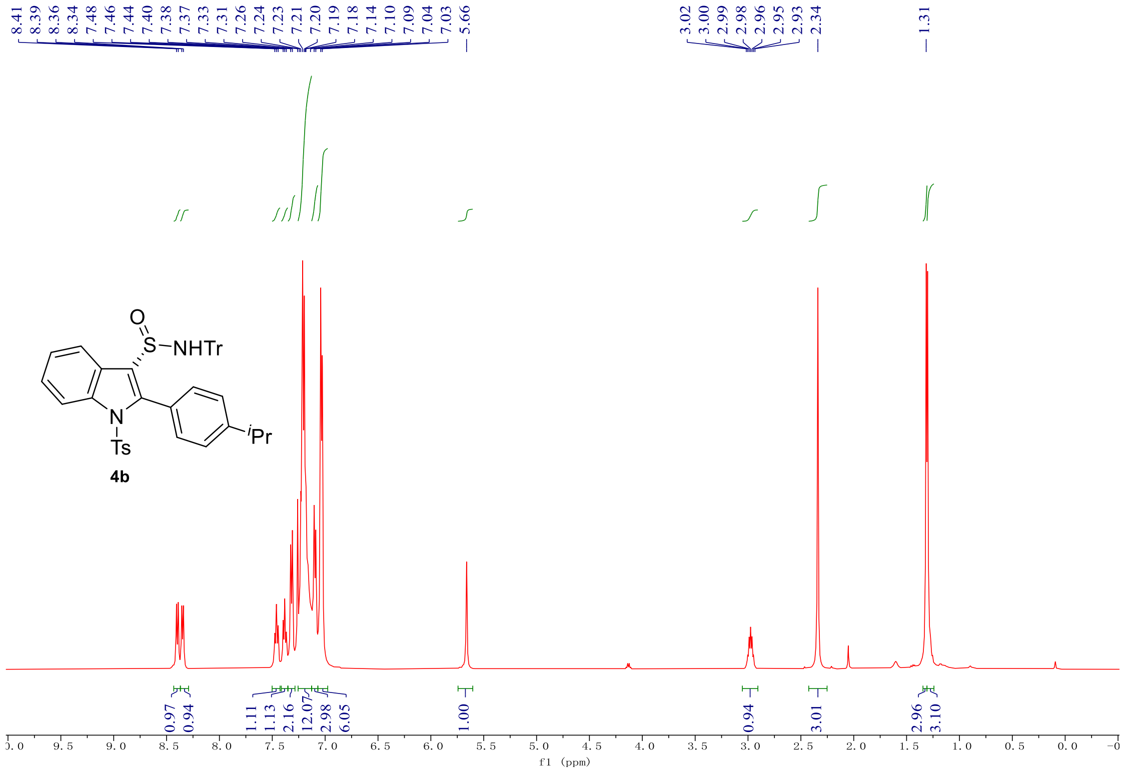
8.41 8.39 8.36 8.34 7.48 7.46 7.44 7.40 7.38 7.37 7.33 7.31 7.26 7.24 7.23 7.21 7.20 7.19 7.18 7.14 7.10 7.09 7.04 7.03 5.66
3.02 3.00 2.99 2.98 2.96 2.95 2.93 2.34
1.31
S–NHTr
Ts
4b
0.97 0.94 1.11 1.13 2.16 12.07 2.98 6.05 1.00 0.94 3.01 2.96 3.10
f1 (ppm)

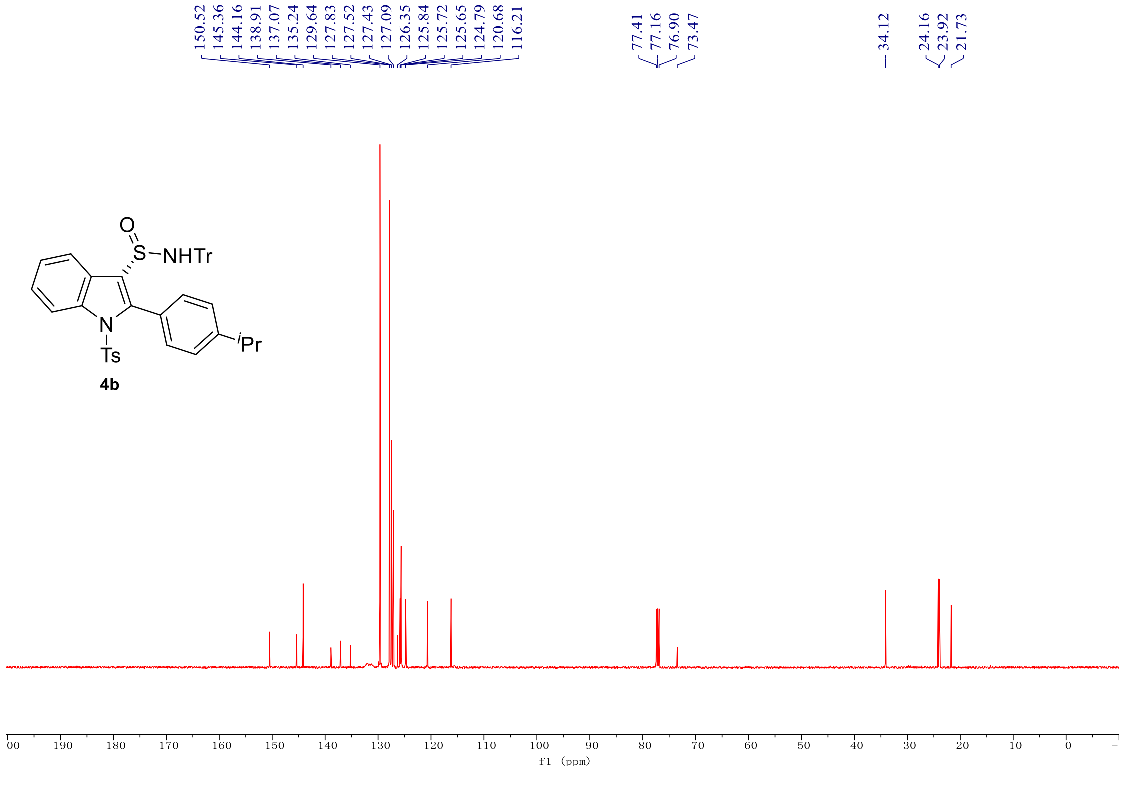
150.52 145.36 144.16 138.91 137.07 135.24 129.64 127.83 127.52 127.43 127.09 126.35 125.84 125.72 125.65 124.79 120.68 116.21
77.41 77.16 76.90 73.47
34.12
24.16 23.92 21.73
S–NHTr
Ts
4b
f1 (ppm)

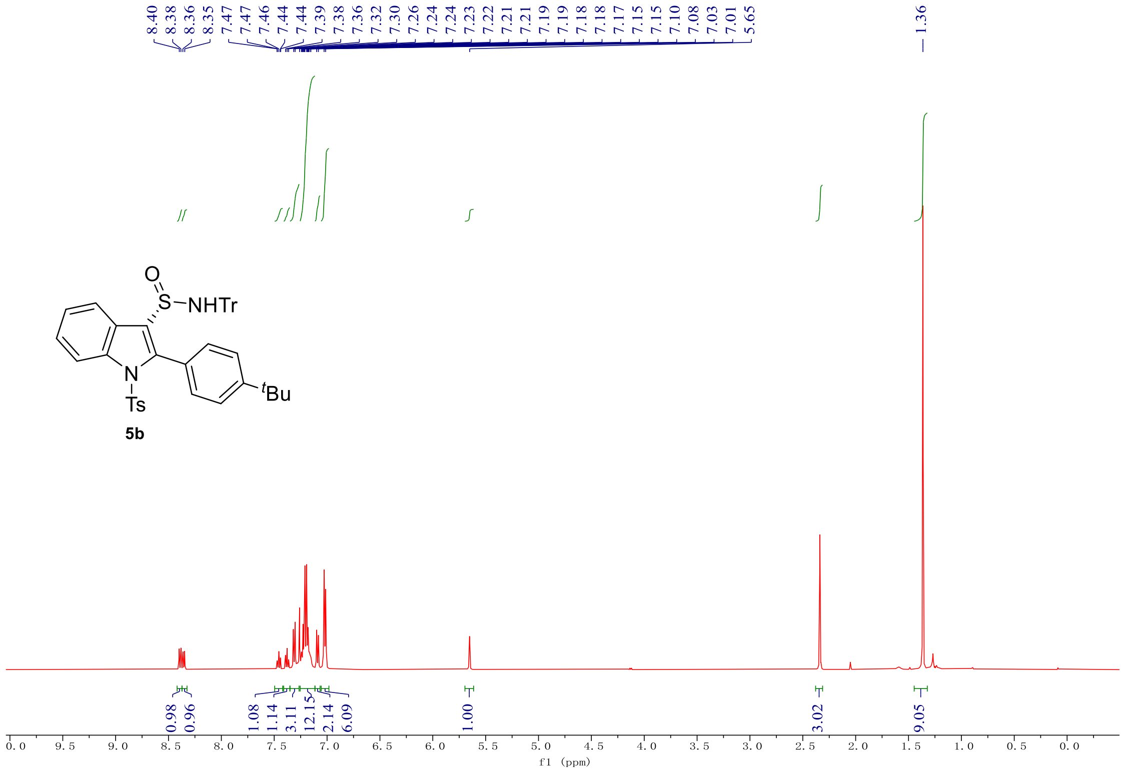

8.40 8.38 8.36 8.35 7.47 7.47 7.46 7.44 7.44 7.39 7.38 7.36 7.32 7.30 7.26 7.24 7.24 7.23 7.22 7.21 7.21 7.19 7.19 7.18 7.18 7.17 7.15 7.15 7.10 7.08 7.03 7.01 5.65
1.36
S–NHTr
O
N
Ts
tBu
5b
0.98 0.96 1.08 1.14 3.11 12.15 2.14 6.09 1.00 3.02 9.05
f1 (ppm)

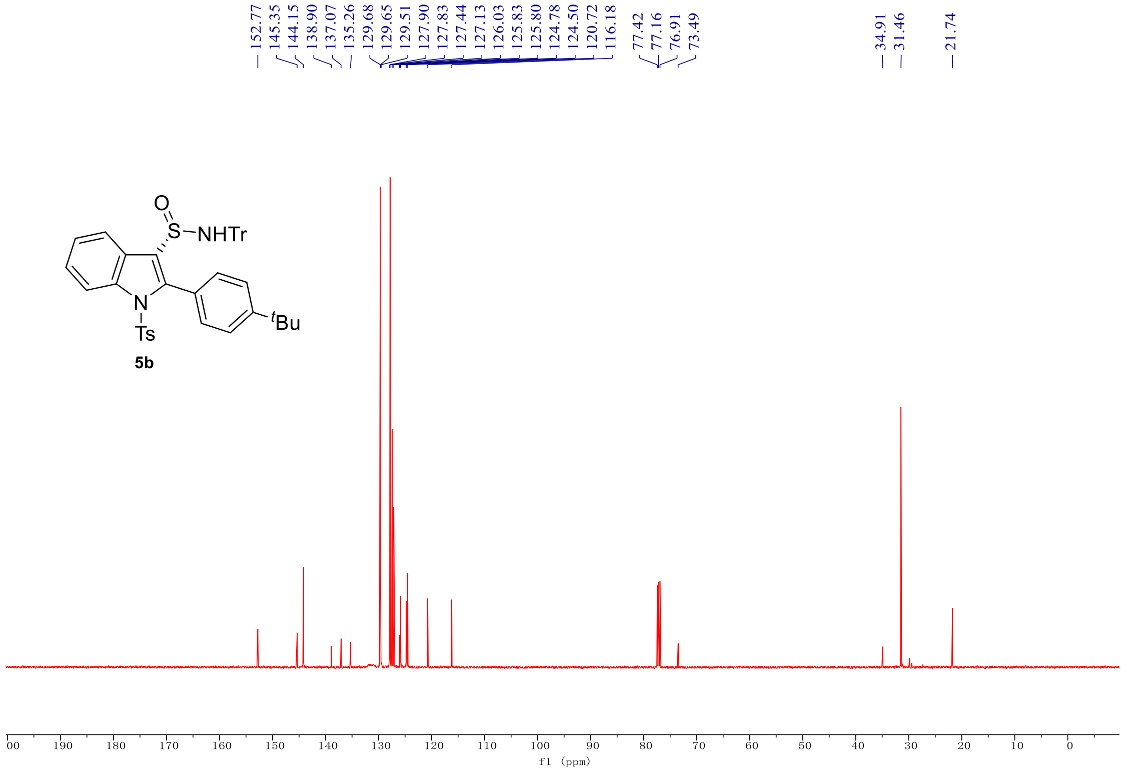

152.77 145.35 144.15 138.90 137.07 135.26 129.68 129.65 129.51 127.90 127.83 127.44 127.13 126.03 125.83 125.80 124.78 124.50 120.72 116.18
77.42 77.16 76.91 73.49
34.91 31.46 21.74
S–NHTr
O
N
Ts
tBu
5b
f1 (ppm)

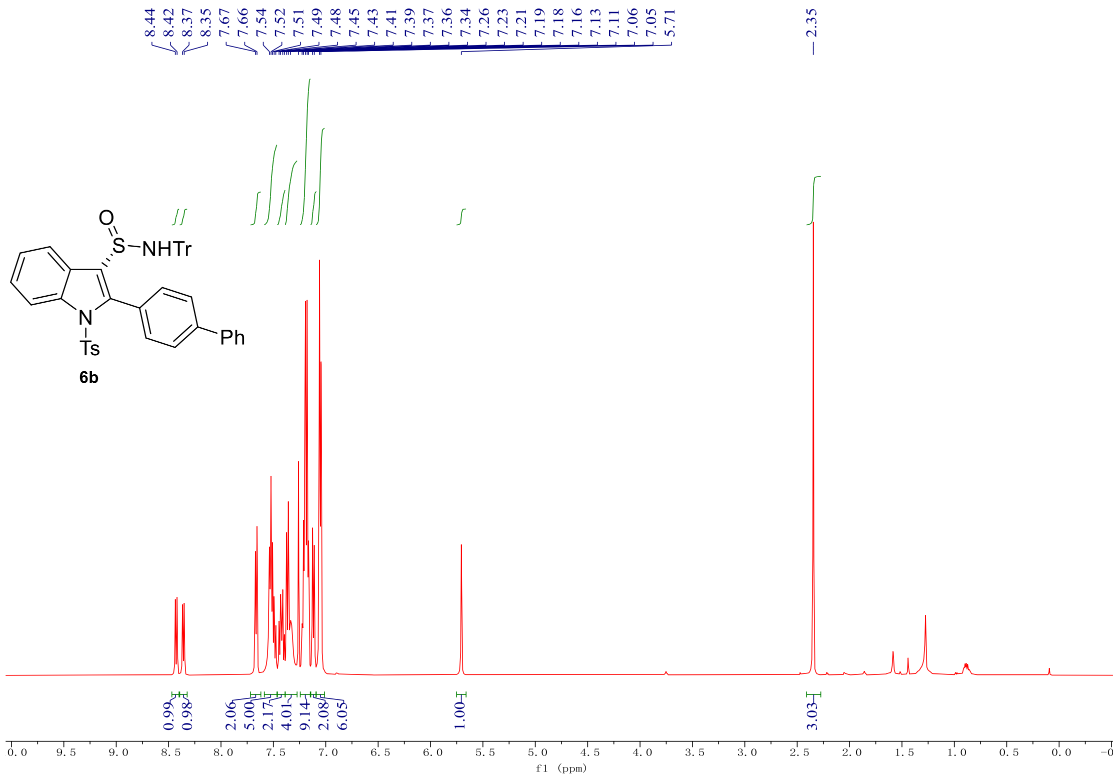
8.44
8.42
8.37
8.35
7.67
7.66
7.54
7.52
7.51
7.49
7.48
7.45
7.43
7.41
7.39
7.37
7.36
7.34
7.26
7.23
7.21
7.19
7.18
7.16
7.13
7.11
7.06
7.05
5.71
2.35
S
O
NHTr
N
Ts
Ph
6b
0.99
0.98
2.06
5.00
2.17
4.01
9.14
2.08
6.05
1.00
3.03
f1 (ppm)

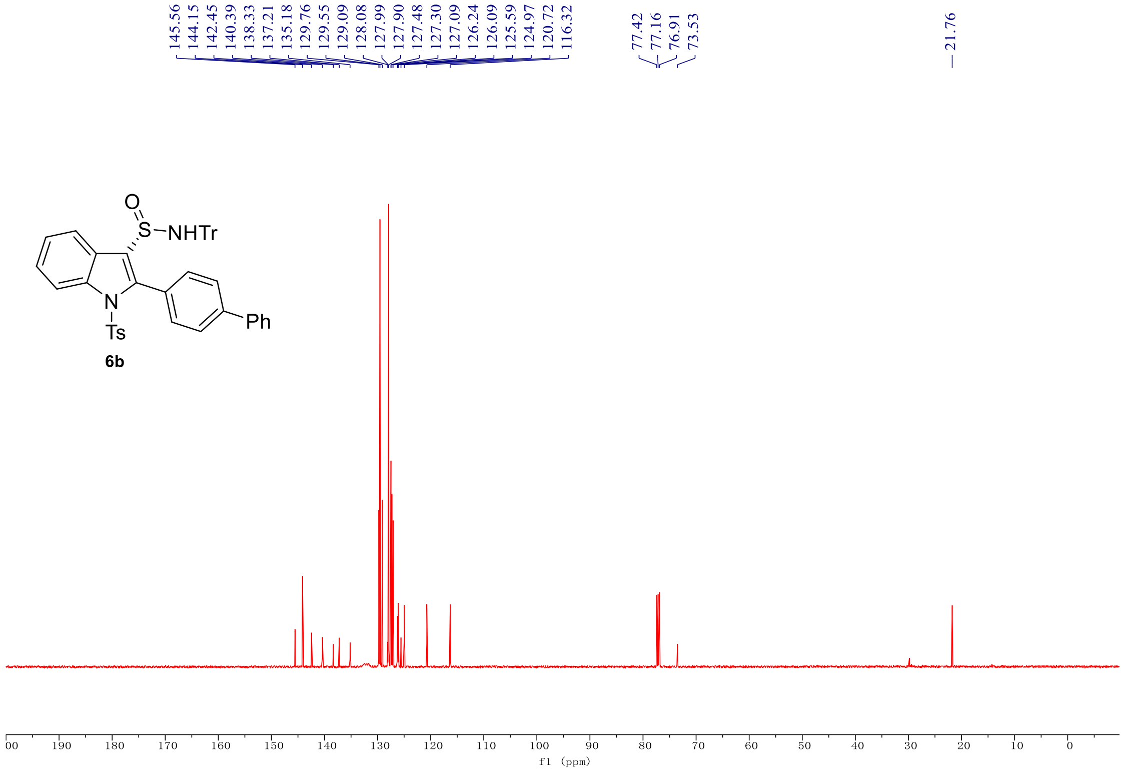
145.56
144.15
142.45
140.39
138.33
137.21
135.18
129.76
129.55
129.09
128.08
127.99
127.90
127.48
127.30
127.09
126.24
126.09
125.59
124.97
120.72
116.32
77.42
77.16
76.91
73.53
21.76
S
O
NHTr
N
Ts
Ph
6b
f1 (ppm)

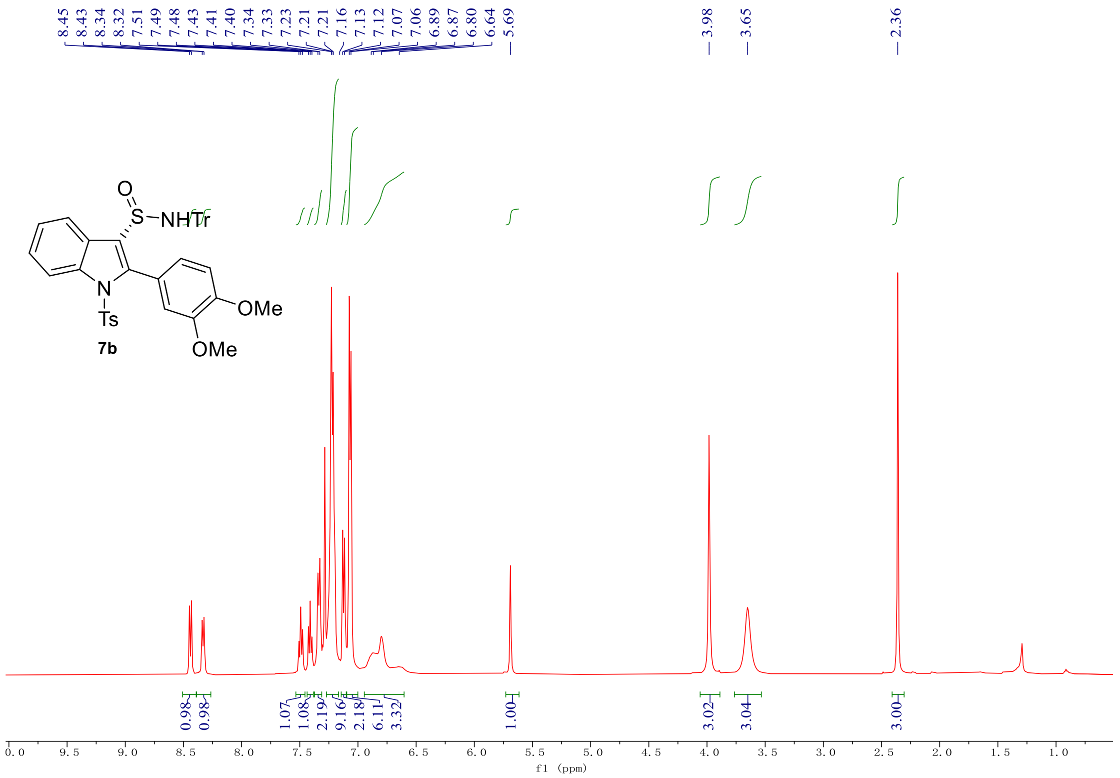

8.45 8.43 8.34 8.32 7.51 7.49 7.48 7.43 7.41 7.40 7.34 7.33 7.23 7.21 7.16 7.13 7.12 7.07 7.06 6.89 6.87 6.80 6.64 5.69
3.98
3.65
2.36
NHTr
OMe
OMe
7b
0.98 0.98 1.07 1.08 2.19 9.16 2.18 6.11 3.32 1.00 3.02 3.04 3.00
f1 (ppm)

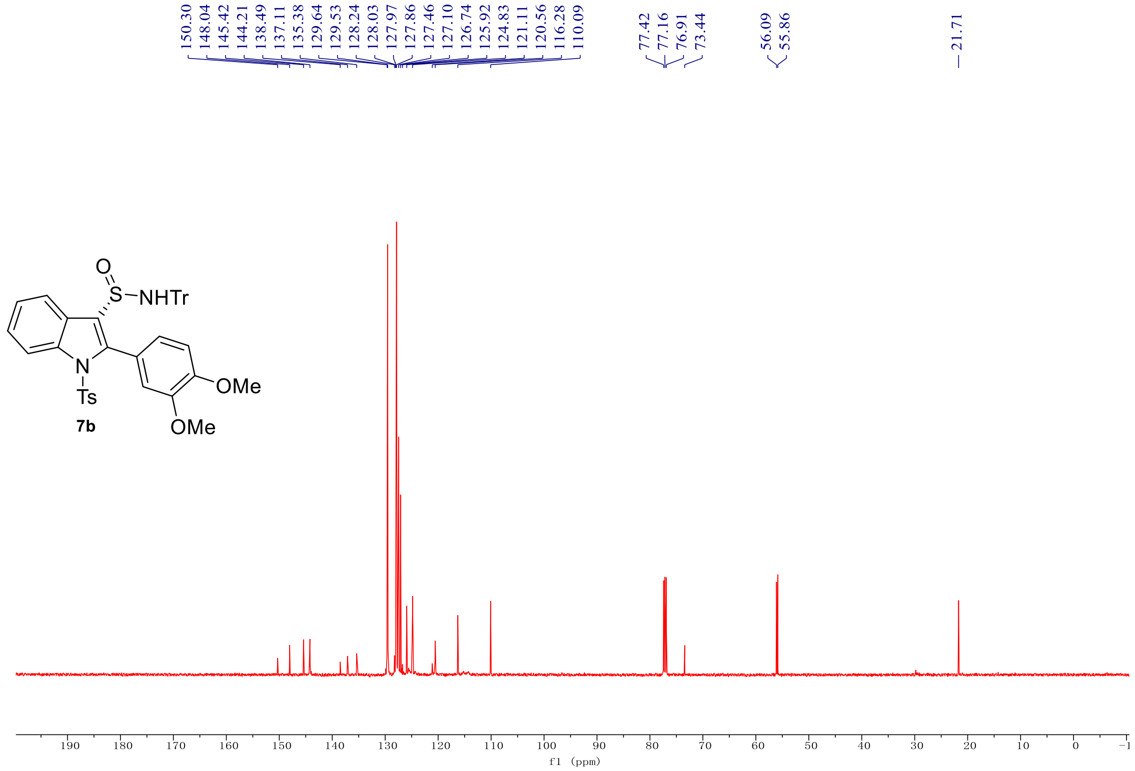

150.30 148.04 145.42 144.21 138.49 137.11 135.38 129.64 129.53 128.24 128.03 127.97 127.86 127.46 127.10 126.74 125.92 124.83 121.11 120.56 116.28 110.09
77.42 77.16 76.91 73.44
56.09 55.86
21.71
NHTr
OMe
OMe
7b
f1 (ppm)

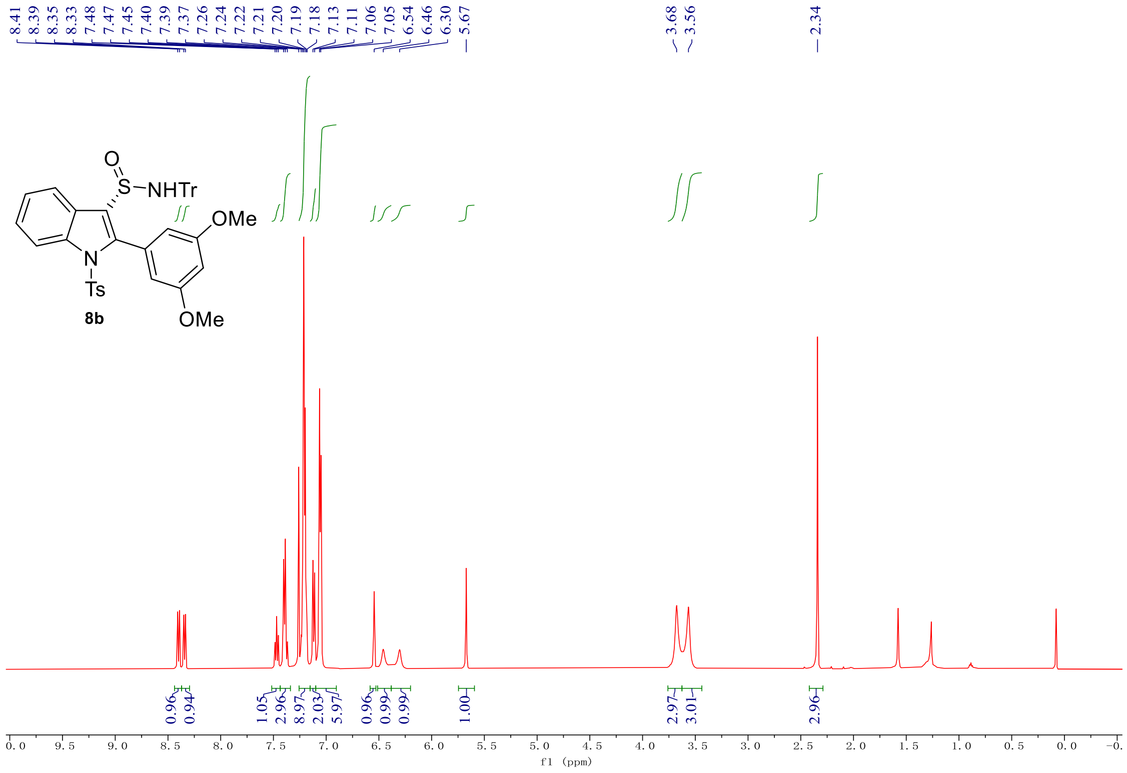

8.41
8.39
8.35
8.33
7.48
7.47
7.45
7.40
7.39
7.37
7.26
7.24
7.22
7.21
7.20
7.19
7.18
7.13
7.11
7.06
7.05
6.54
6.46
6.30
5.67
3.68
3.56
2.34
O
S
NHTr
OMe
N
Ts
8b
OMe
0.96
0.94
1.05
2.96
8.97
2.03
5.97
0.96
0.99
0.99
1.00
2.97
3.01
2.96
f1 (ppm)

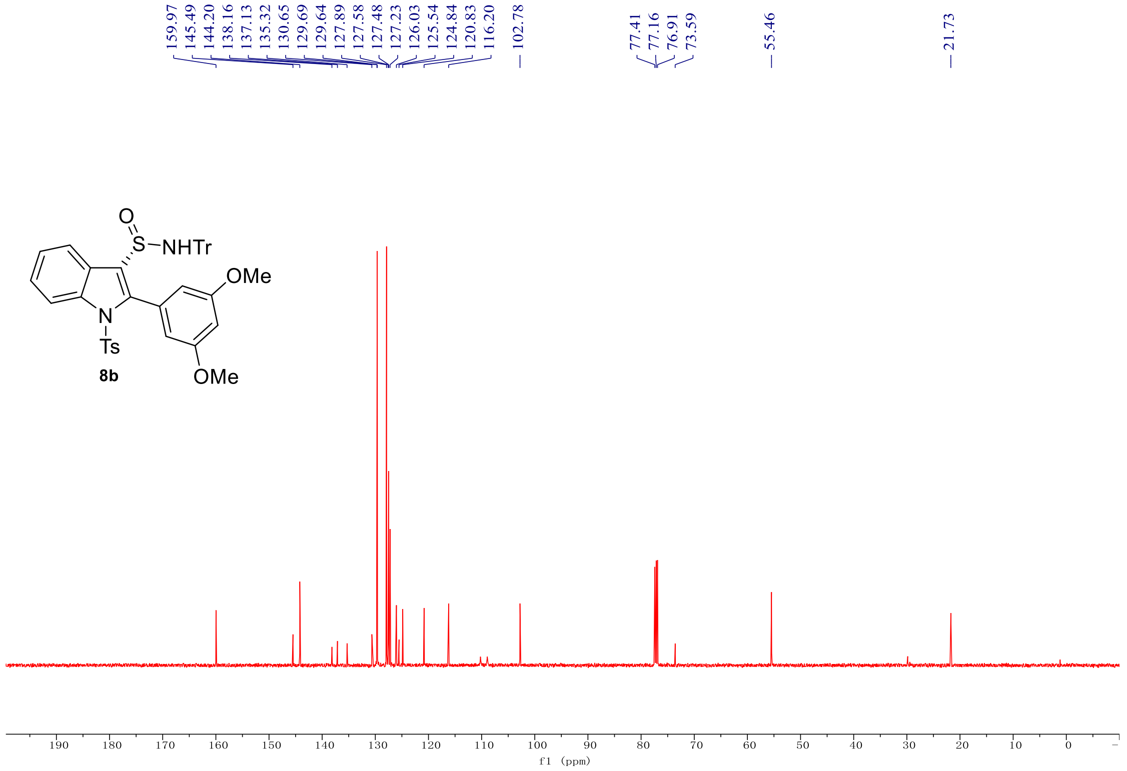

159.97
145.49
144.20
138.16
137.13
135.32
130.65
129.69
129.64
127.89
127.58
127.48
127.23
126.03
125.54
124.84
120.83
116.20
102.78
77.41
77.16
76.91
73.59
55.46
21.73
O
S
NHTr
OMe
N
Ts
8b
OMe
f1 (ppm)

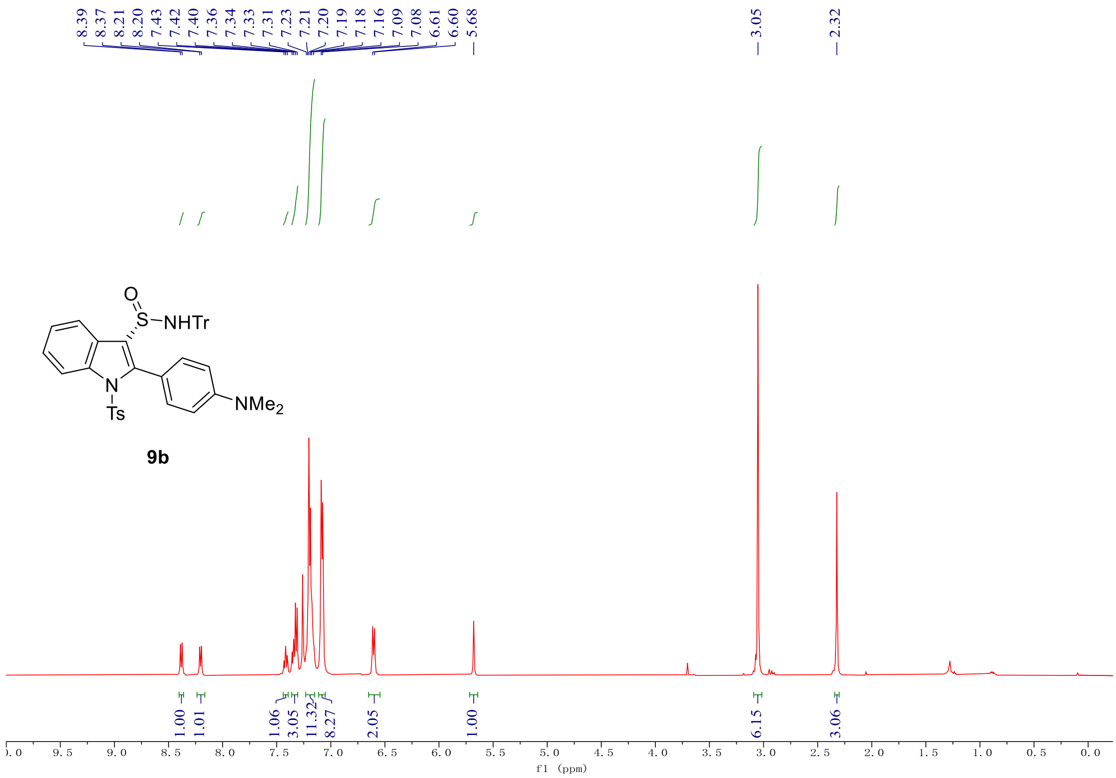
8.39
8.37
8.21
8.20
7.43
7.42
7.40
7.36
7.34
7.33
7.31
7.23
7.21
7.20
7.19
7.18
7.16
7.09
7.08
6.61
6.60
5.68
3.05
2.32
O
S
NHTr
N
Ts
NMe2
9b
1.00
1.01
1.06
3.05
11.32
8.27
2.05
1.00
6.15
3.06
f1 (ppm)

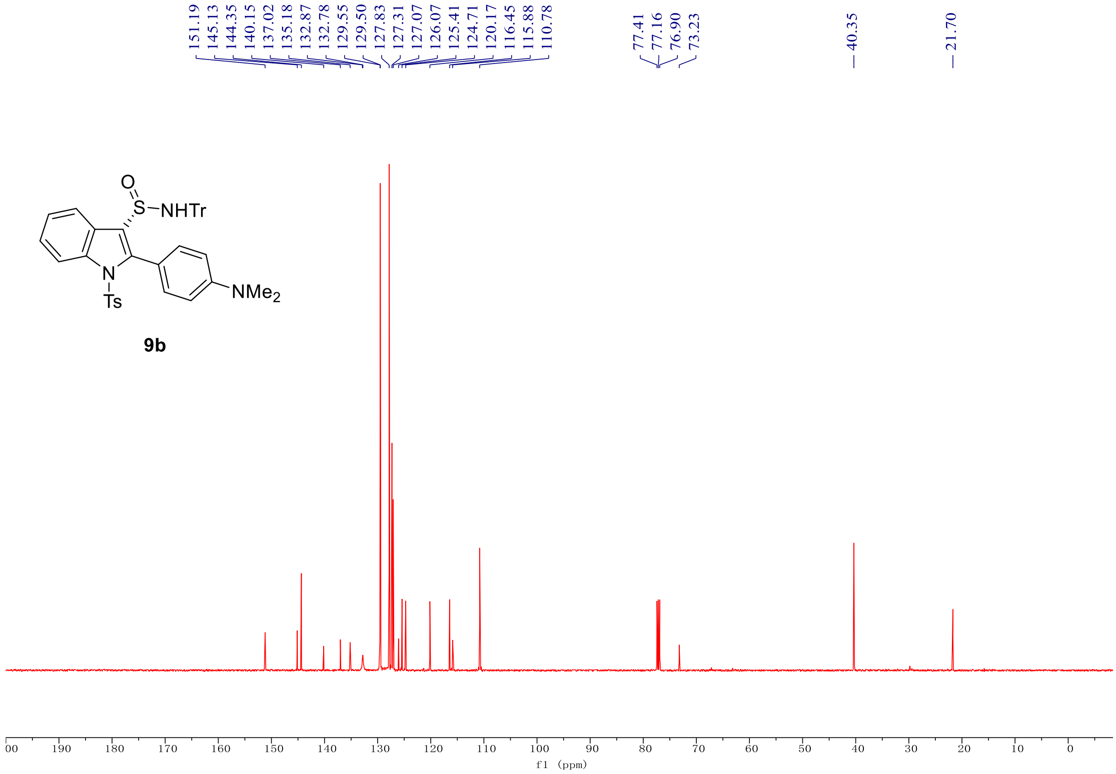
151.19
145.13
144.35
140.15
137.02
135.18
132.87
132.78
129.55
129.50
127.83
127.31
127.07
126.07
125.41
124.71
120.17
116.45
115.88
110.78
77.41
77.16
76.90
73.23
40.35
21.70
O
S
NHTr
N
Ts
NMe2
9b
f1 (ppm)

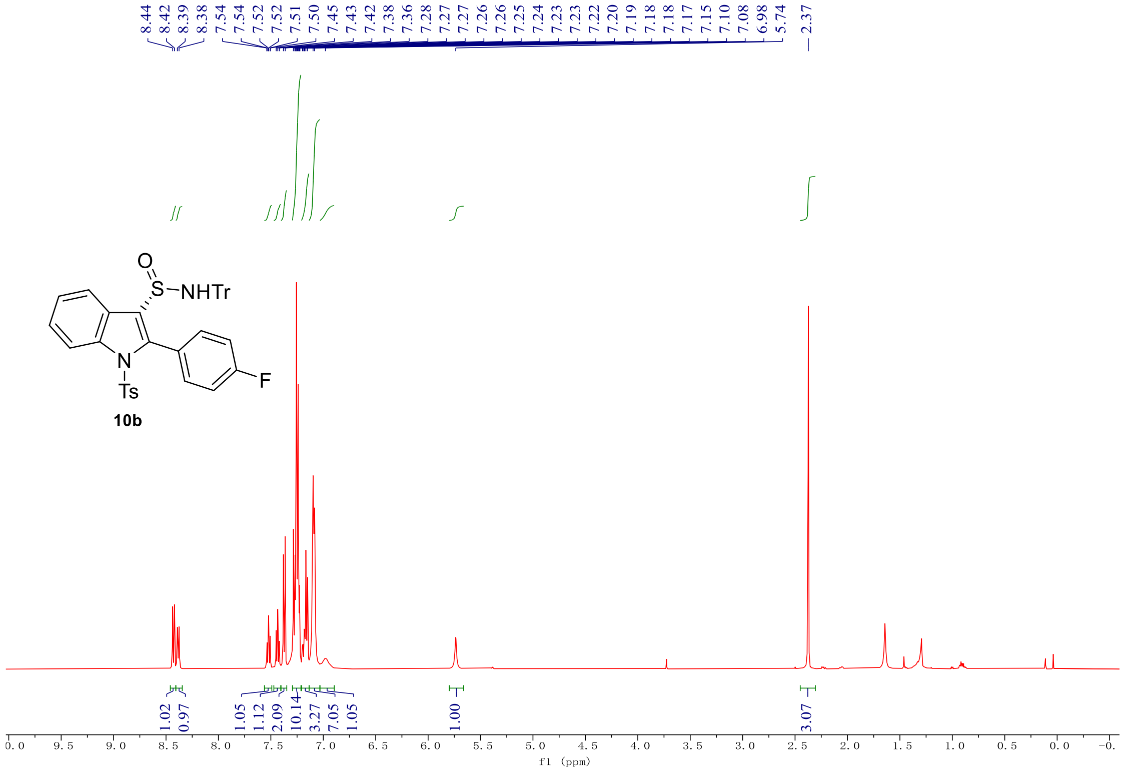
8.44
8.42
8.39
8.38
7.54
7.54
7.52
7.52
7.51
7.50
7.45
7.43
7.42
7.38
7.36
7.28
7.27
7.27
7.26
7.25
7.24
7.23
7.23
7.22
7.20
7.19
7.18
7.18
7.17
7.15
7.10
7.08
6.98
5.74
2.37
O
S
NHTr
N
Ts
F
10b
1.02
0.97
1.05
1.12
2.09
10.14
3.27
7.05
1.05
1.00
3.07
f1 (ppm)

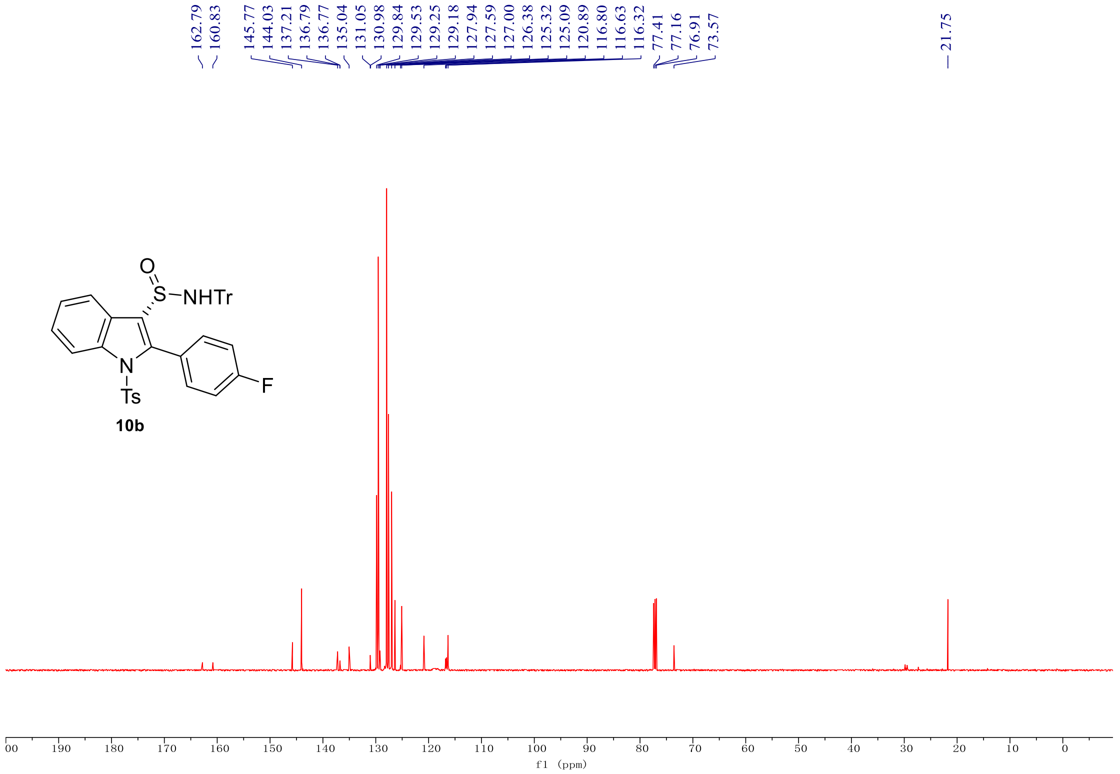
162.79
160.83
145.77
144.03
137.21
136.79
136.77
135.04
131.05
130.98
129.84
129.53
129.25
129.18
127.94
127.59
127.00
126.38
125.32
125.09
120.89
116.80
116.63
116.32
77.41
77.16
76.91
73.57
21.75
O
S
NHTr
N
Ts
F
10b
f1 (ppm)

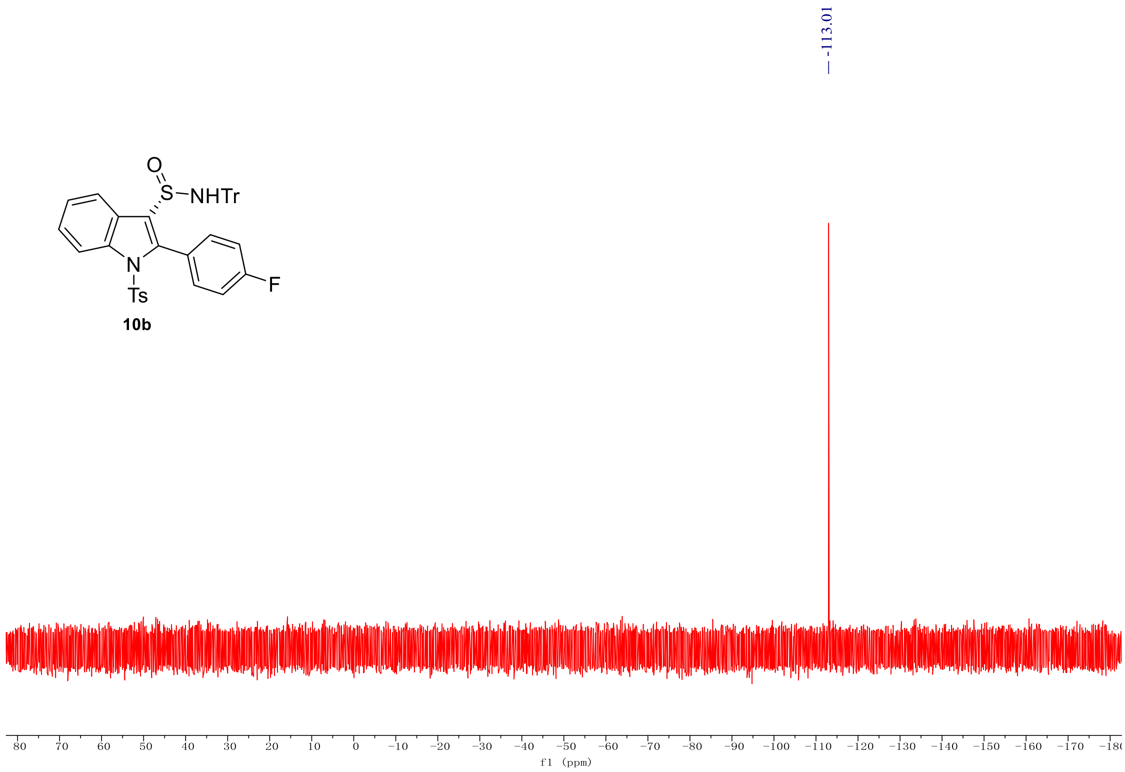
O
S
NHTr
N
Ts
F
-113.01
80 70 60 50 40 30 20 10 0 -10 -20 -30 -40 -50 -60 -70 -80 -90 -100 -110 -120 -130 -140 -150 -160 -170
f1 (ppm)

10b

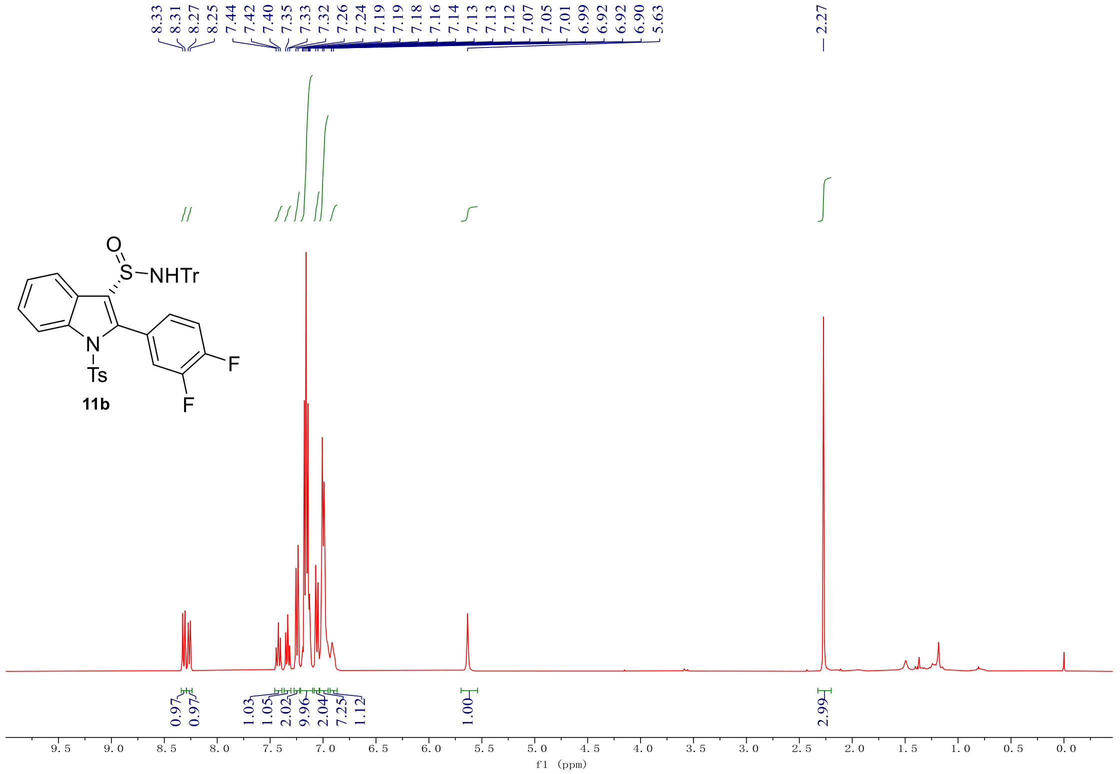
O
S
NHTr
N
Ts
F
F
8.33
8.31
8.27
8.25
7.44
7.42
7.40
7.35
7.33
7.32
7.26
7.24
7.19
7.19
7.18
7.16
7.14
7.13
7.13
7.12
7.07
7.05
7.01
6.99
6.92
6.92
6.90
5.63
2.27
0.97
0.97
1.03
1.05
2.02
9.96
2.04
7.25
1.12
1.00
2.99
9.5 9.0 8.5 8.0 7.5 7.0 6.5 6.0 5.5 5.0 4.5 4.0 3.5 3.0 2.5 2.0 1.5 1.0 0.5 0.0
f1 (ppm)

11b

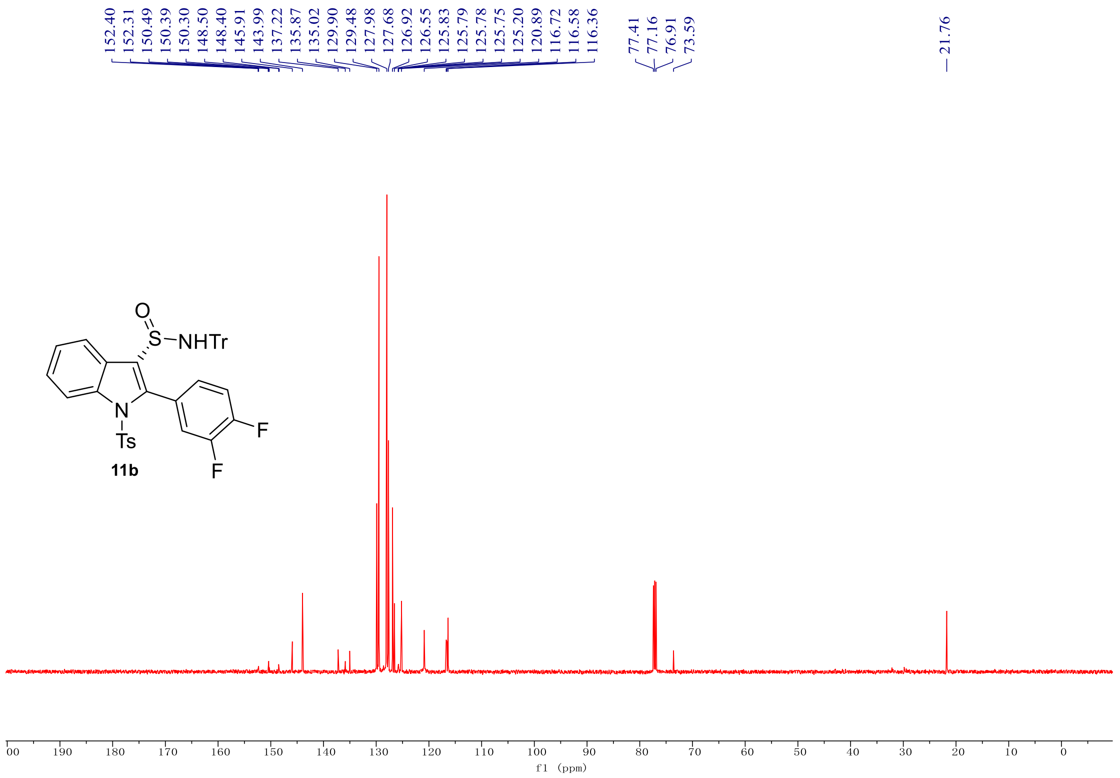
152.40
152.31
150.49
150.39
150.30
148.50
148.40
145.91
143.99
137.22
135.87
135.02
129.90
129.48
127.98
127.68
126.92
126.55
125.83
125.79
125.78
125.75
125.20
120.89
116.72
116.58
116.36
77.41
77.16
76.91
73.59
21.76
S
NHTr
N
Ts
F
F
11b
f1 (ppm)

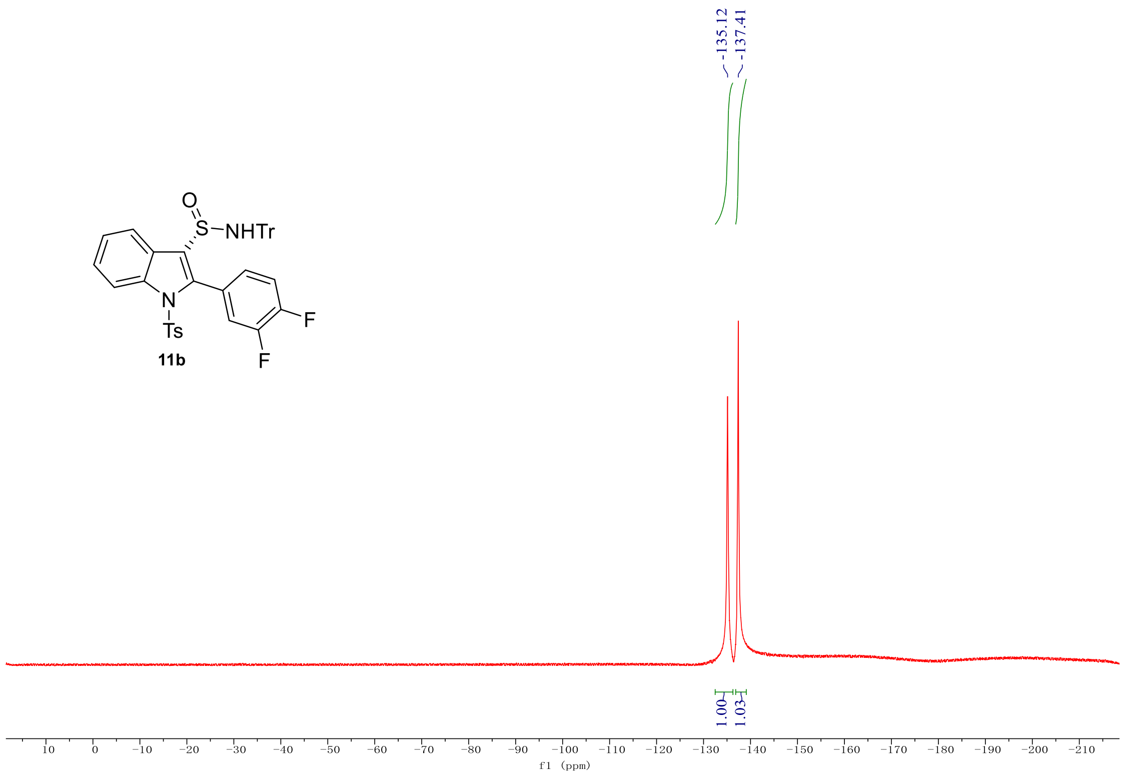
-135.12
-137.41
S
NHTr
N
Ts
F
F
11b
1.00
1.03
f1 (ppm)

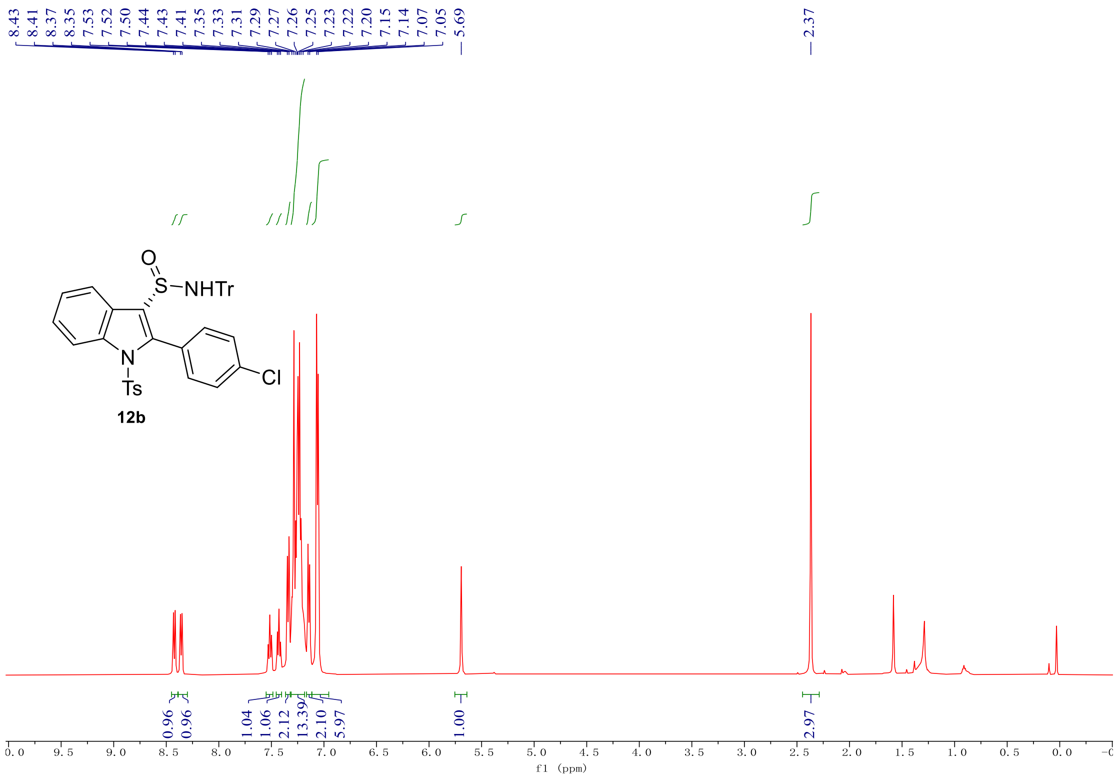
8.43
8.41
8.37
8.35
7.53
7.52
7.50
7.44
7.43
7.41
7.35
7.33
7.31
7.29
7.27
7.26
7.25
7.23
7.22
7.20
7.15
7.14
7.07
7.05
5.69
2.37
O
S
NHTr
N
Ts
Cl
12b
0.96
0.96
1.04
1.06
2.12
13.39
2.10
5.97
1.00
2.97
f1 (ppm)

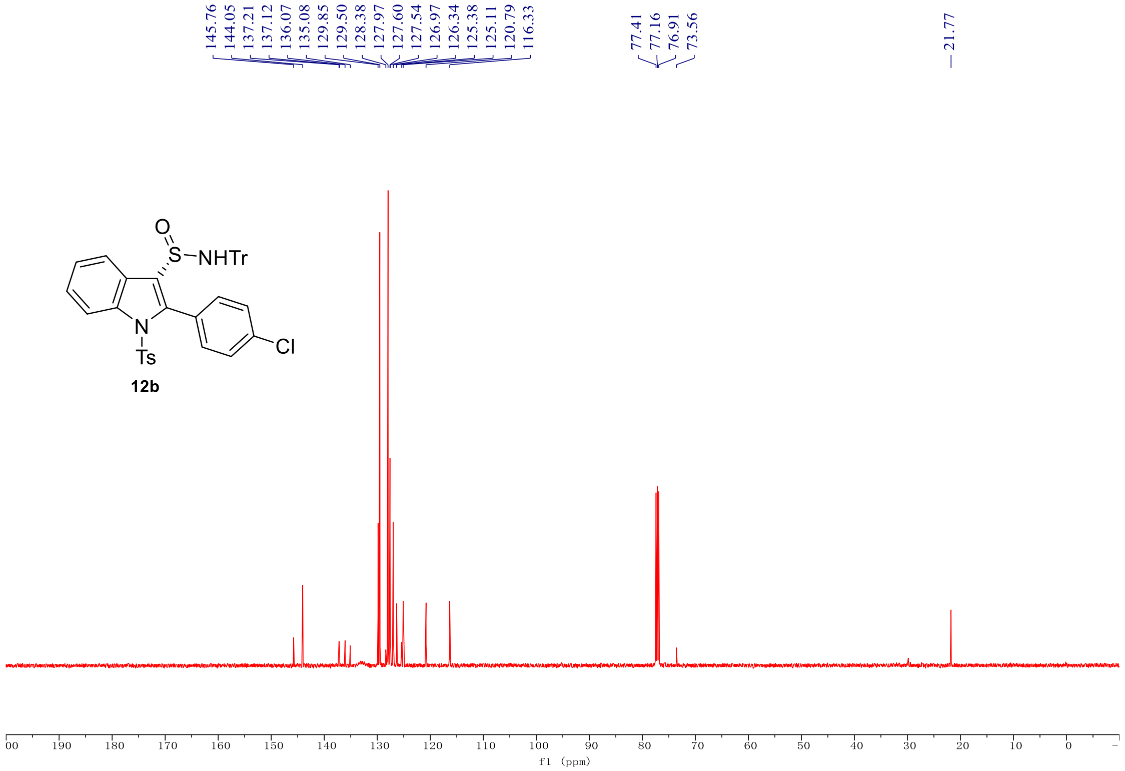
145.76
144.05
137.21
137.12
136.07
135.08
129.85
129.50
128.38
127.97
127.60
127.54
126.97
126.34
125.38
125.11
120.79
116.33
77.41
77.16
76.91
73.56
21.77
O
S
NHTr
N
Ts
Cl
12b
f1 (ppm)

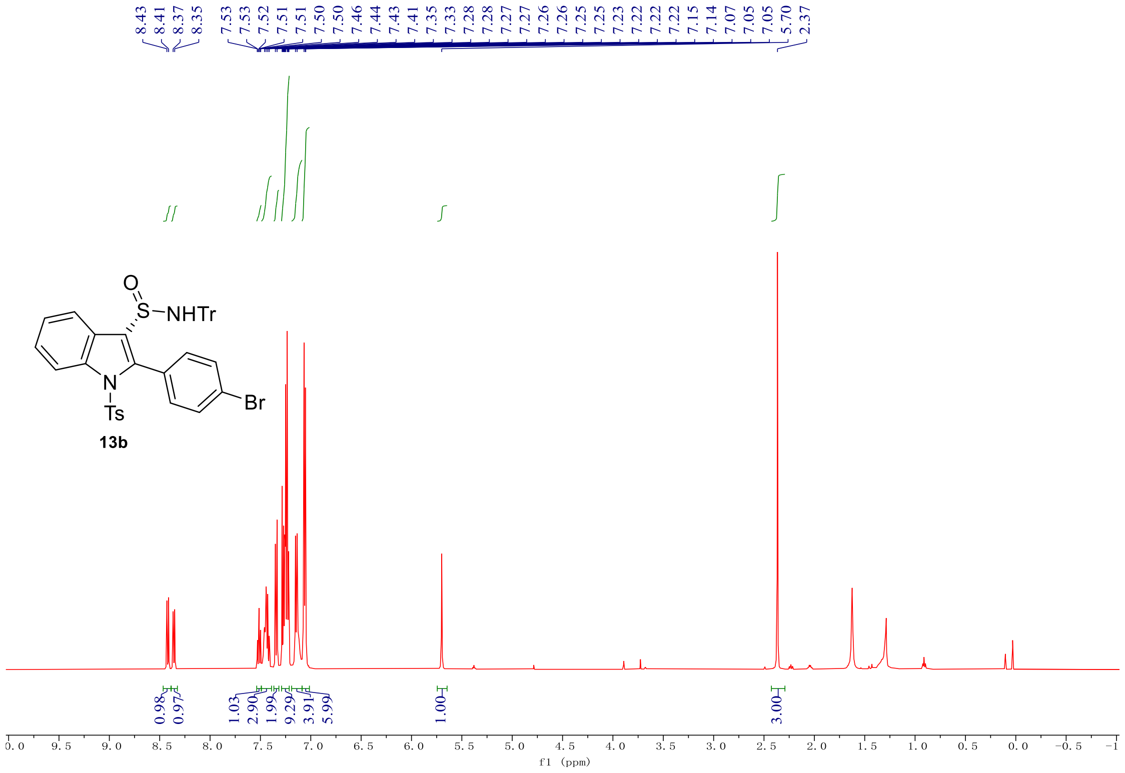
8.43
8.41
8.37
8.35
7.53
7.53
7.52
7.51
7.51
7.50
7.50
7.46
7.44
7.43
7.41
7.35
7.33
7.28
7.28
7.27
7.27
7.26
7.26
7.25
7.25
7.23
7.22
7.22
7.22
7.15
7.14
7.07
7.05
7.05
5.70
2.37
S–NHTr
O
N
Ts
Br
13b
0.98
0.97
1.03
2.90
1.99
9.29
3.91
5.99
1.00
3.00
f1 (ppm)

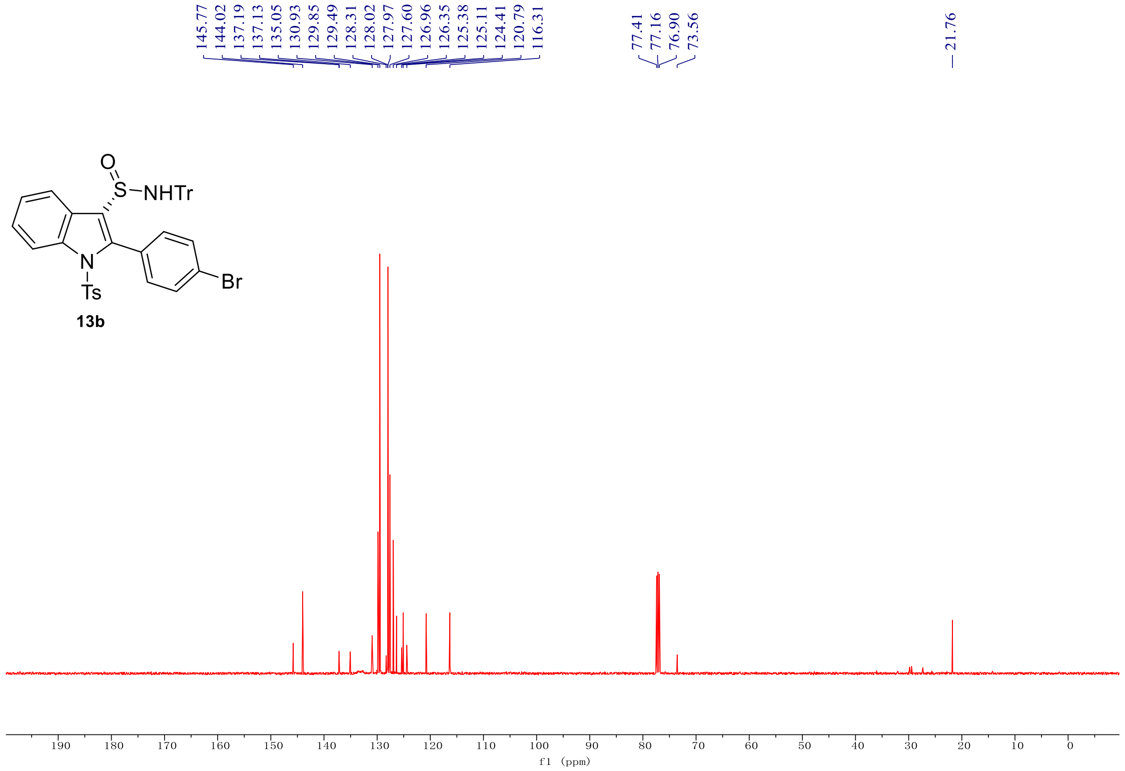
145.77
144.02
137.19
137.13
135.05
130.93
129.85
129.49
128.31
128.02
127.97
127.60
126.96
126.35
125.38
125.11
124.41
120.79
116.31
77.41
77.16
76.90
73.56
21.76
S–NHTr
O
N
Ts
Br
13b
f1 (ppm)

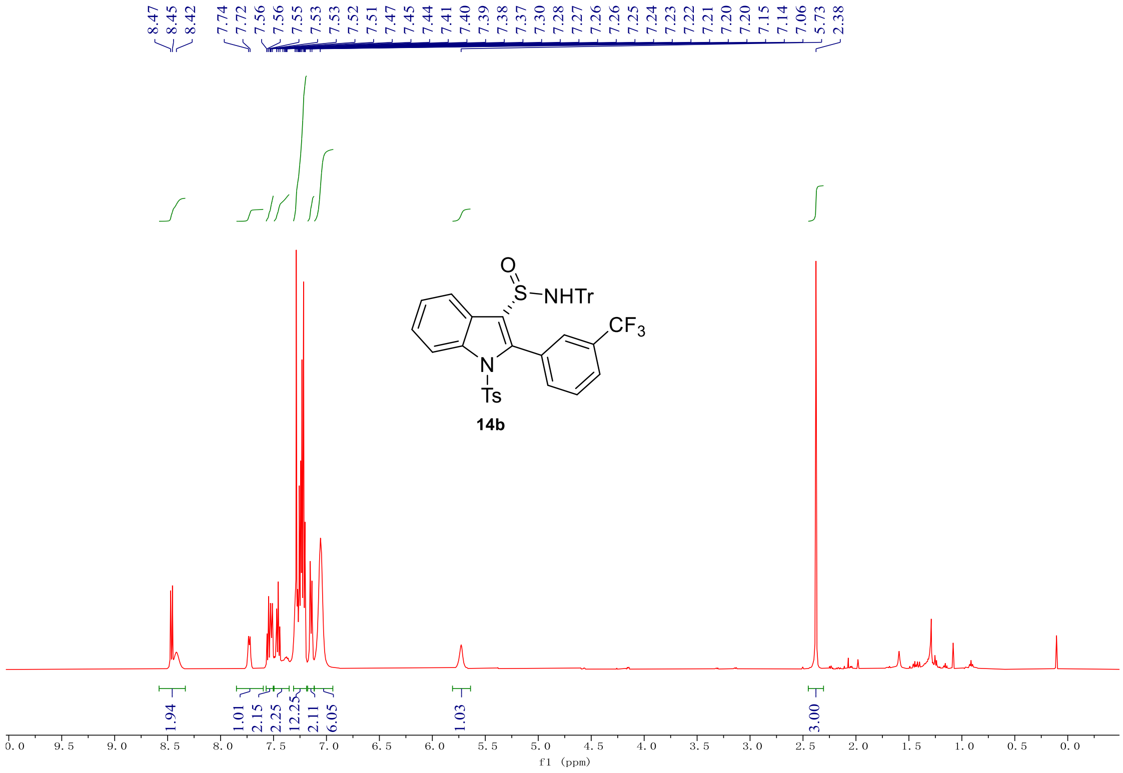

8.47
8.45
8.42
7.74
7.72
7.56
7.56
7.55
7.53
7.52
7.51
7.47
7.45
7.44
7.41
7.40
7.39
7.38
7.37
7.30
7.28
7.27
7.26
7.26
7.25
7.24
7.23
7.22
7.21
7.20
7.20
7.15
7.14
7.06
5.73
2.38
O
S
NHTr
CF3
N
Ts
14b
1.94
1.01
2.15
2.25
12.25
2.11
6.05
1.03
3.00
f1 (ppm)

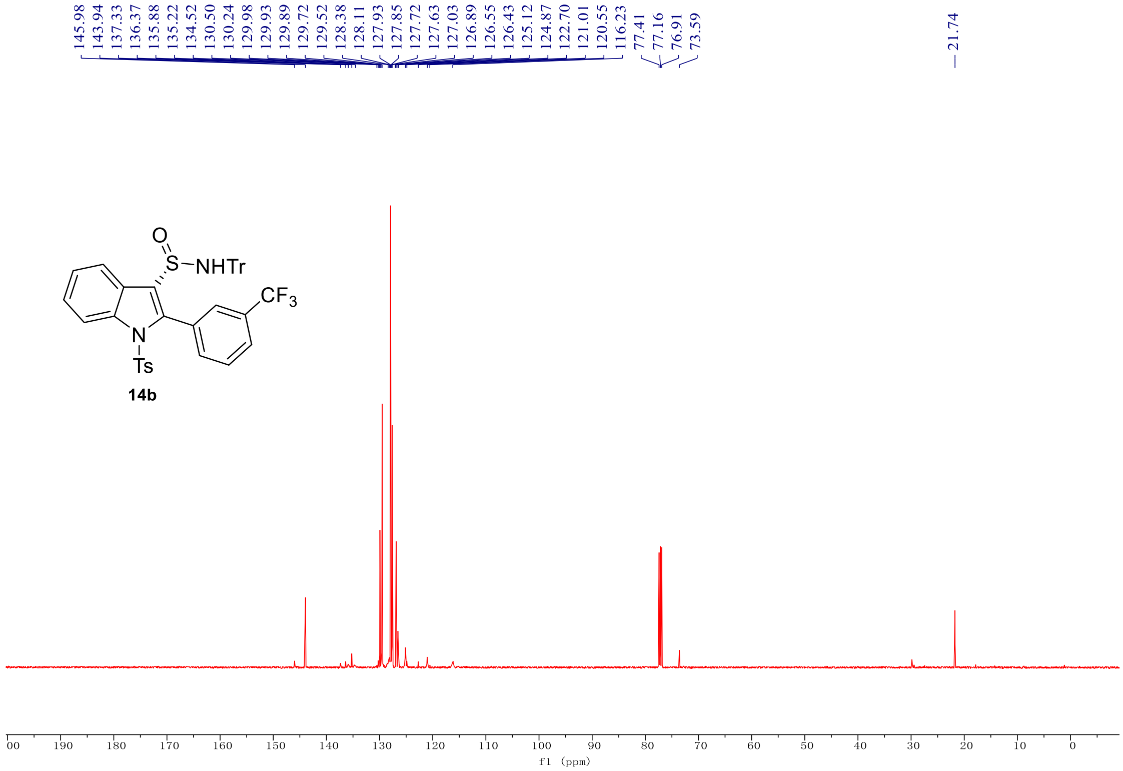

145.98
143.94
137.33
136.37
135.88
135.22
134.52
130.50
130.24
129.98
129.93
129.89
129.72
129.52
128.38
128.11
127.93
127.85
127.72
127.63
127.03
126.89
126.55
126.43
125.12
124.87
122.70
121.01
120.55
116.23
77.41
77.16
76.91
73.59
21.74
O
S
NHTr
CF3
N
Ts
14b
f1 (ppm)

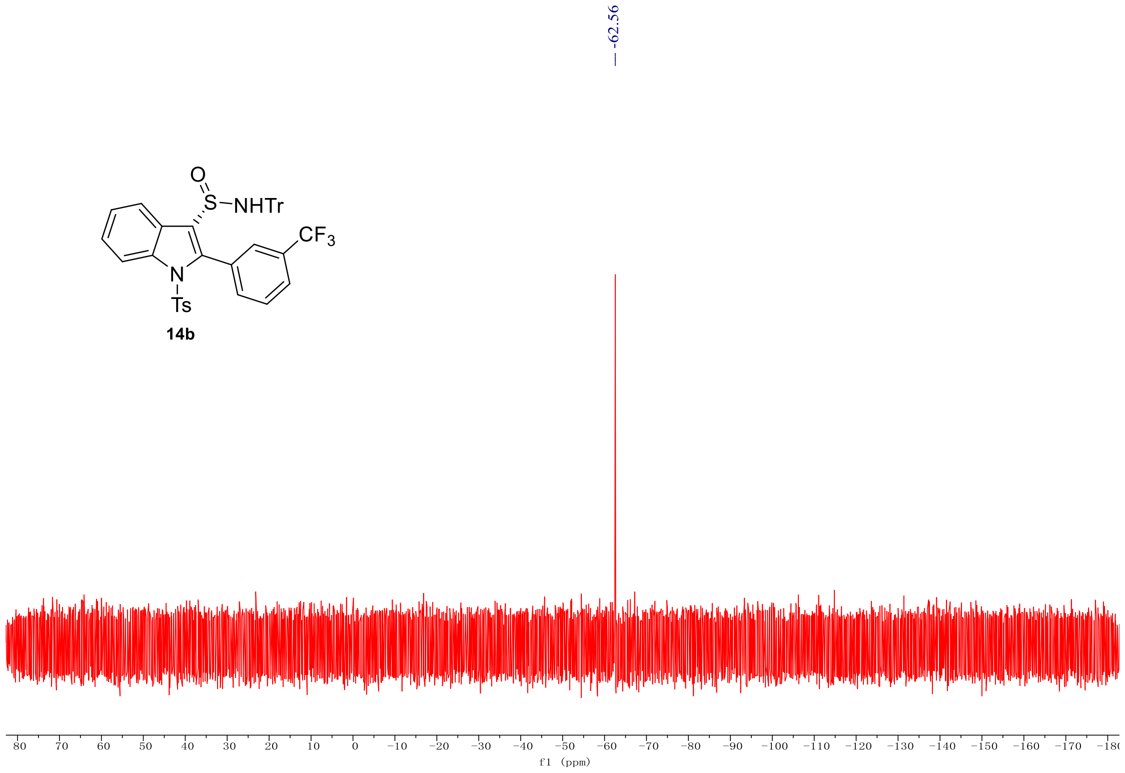

-62.56
O
S
NHTr
CF3
N
Ts
14b
80 70 60 50 40 30 20 10 0 -10 -20 -30 -40 -50 -60 -70 -80 -90 -100 -110 -120 -130 -140 -150 -160 -170
f1 (ppm)

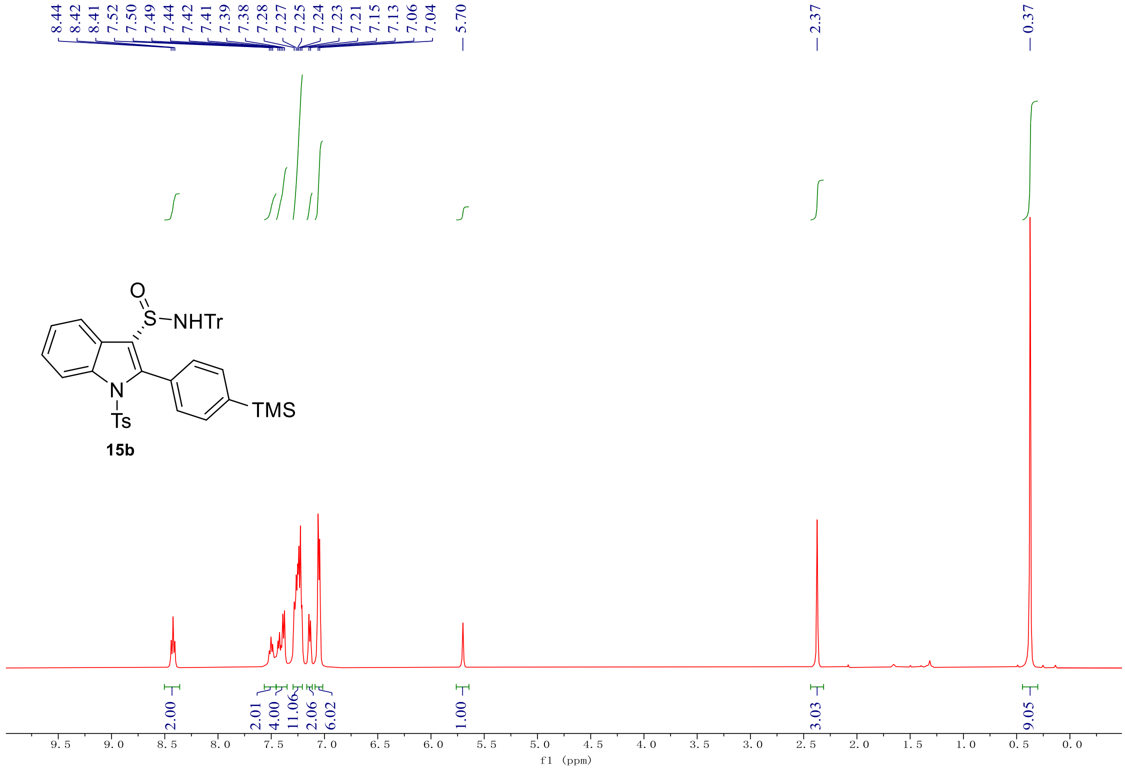

8.44 8.42 8.41 7.52 7.50 7.49 7.44 7.42 7.41 7.39 7.38 7.28 7.27 7.25 7.24 7.23 7.21 7.15 7.13 7.06 7.04
5.70
2.37
0.37
O
S
NHTr
N
Ts
TMS
15b
2.00
2.01
4.00
11.06
2.06
6.02
1.00
3.03
9.05
9.5 9.0 8.5 8.0 7.5 7.0 6.5 6.0 5.5 5.0 4.5 4.0 3.5 3.0 2.5 2.0 1.5 1.0 0.5 0.0
f1 (ppm)

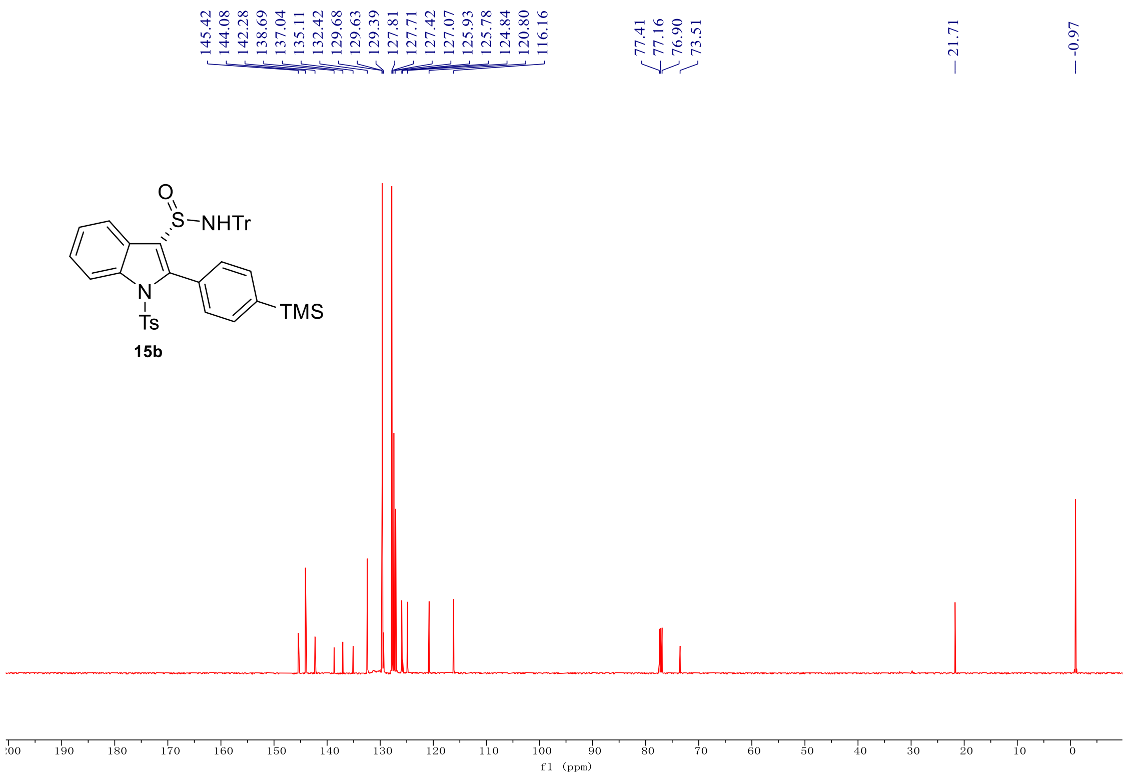
145.42
144.08
142.28
138.69
137.04
135.11
132.42
129.68
129.63
129.39
127.81
127.71
127.42
127.07
125.93
125.78
124.84
120.80
116.16
77.41
77.16
76.90
73.51
21.71
-0.97
S-NHTr
O
N
Ts
TMS
15b
f1 (ppm)

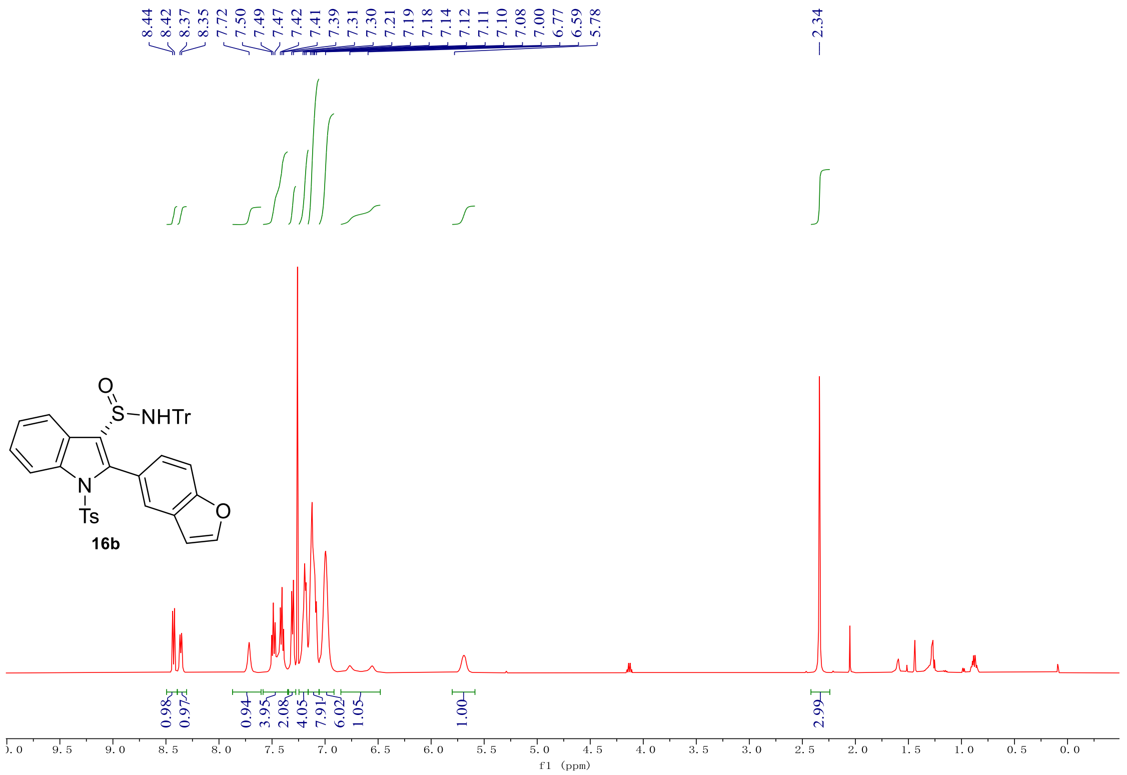
8.44
8.42
8.37
8.35
7.72
7.50
7.49
7.47
7.42
7.41
7.39
7.31
7.30
7.21
7.19
7.18
7.14
7.12
7.11
7.10
7.08
7.00
6.77
6.59
5.78
2.34
S-NHTr
O
N
Ts
O
16b
0.98
0.97
0.94
3.95
2.08
4.05
7.91
6.02
1.05
1.00
2.99
f1 (ppm)

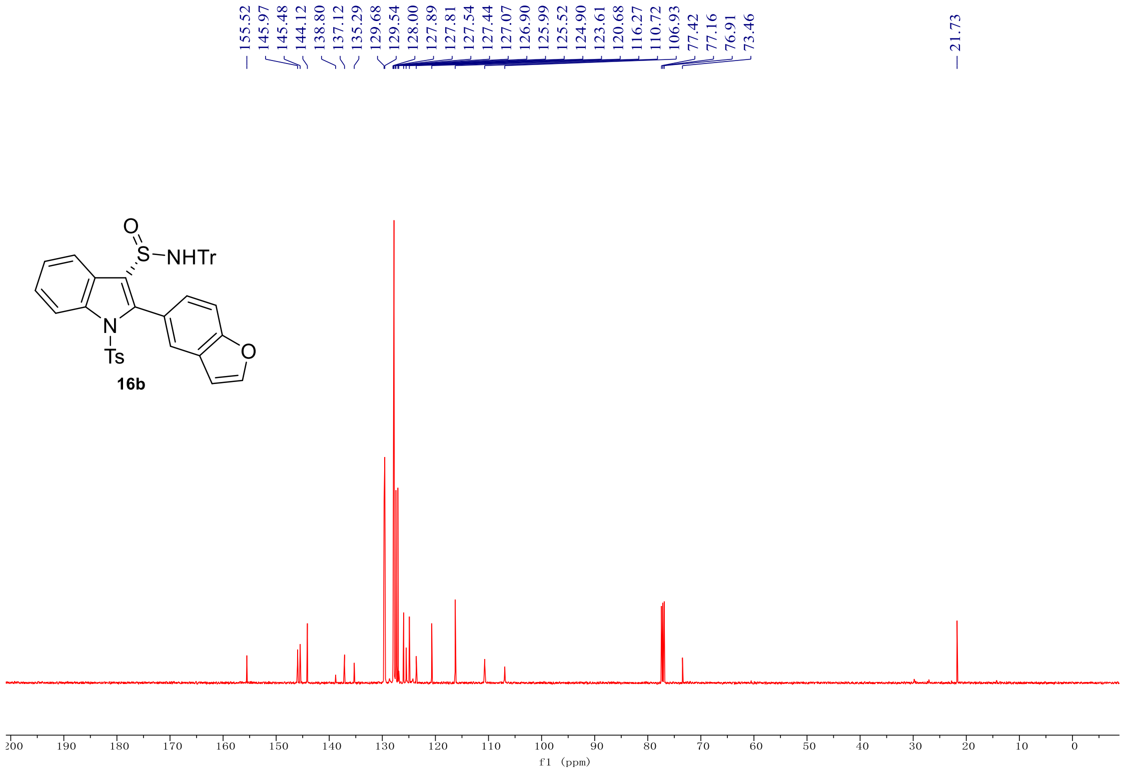
155.52
145.97
145.48
144.12
138.80
137.12
135.29
129.68
129.54
128.00
127.89
127.81
127.54
127.44
127.07
126.90
125.99
125.52
124.90
123.61
120.68
116.27
110.72
106.93
77.42
77.16
76.91
73.46
21.73
O
S
NHTr
N
Ts
O
16b
200 190 180 170 160 150 140 130 120 110 100 90 80 70 60 50 40 30 20 10 0
f1 (ppm)

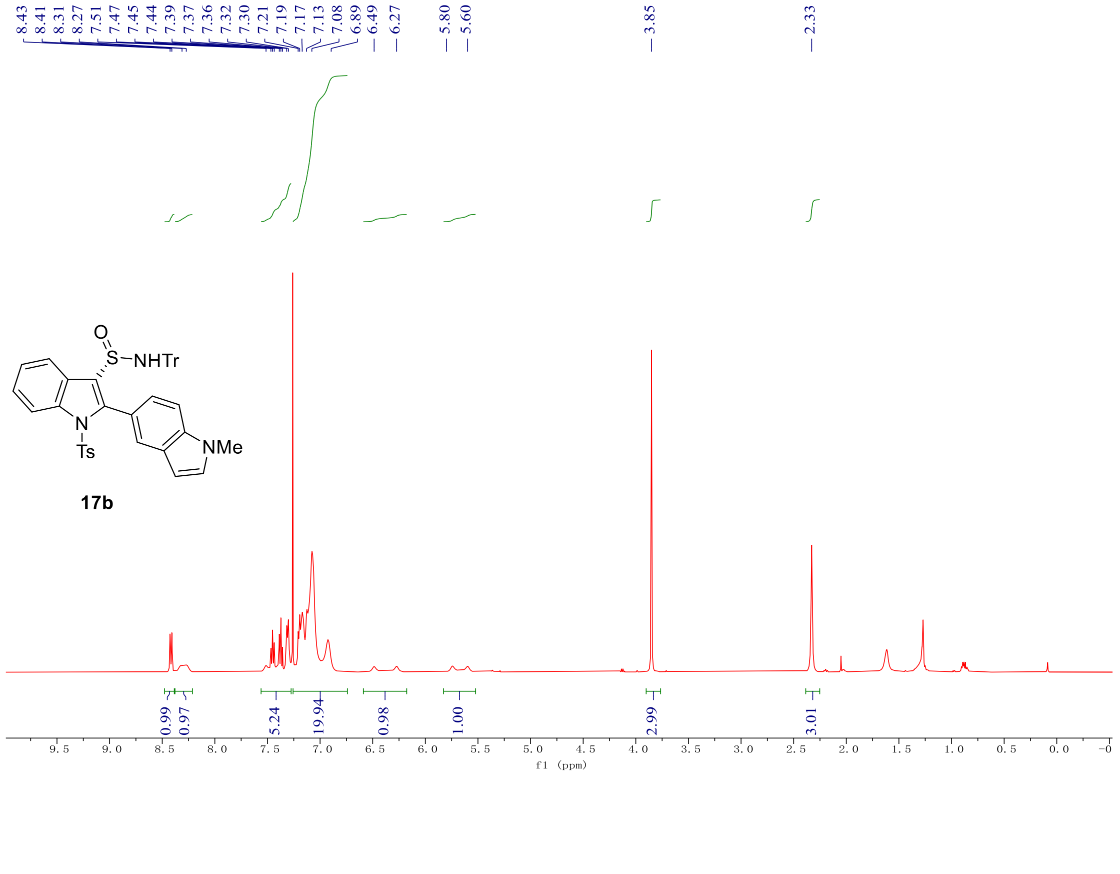
8.43
8.41
8.31
8.27
7.51
7.47
7.45
7.44
7.39
7.37
7.36
7.32
7.30
7.21
7.19
7.17
7.13
7.08
6.89
6.49
6.27
5.80
5.60
3.85
2.33
O
S
NHTr
N
Ts
NMe
17b
0.99
0.97
5.24
19.94
0.98
1.00
2.99
3.01
9.5 9.0 8.5 8.0 7.5 7.0 6.5 6.0 5.5 5.0 4.5 4.0 3.5 3.0 2.5 2.0 1.5 1.0 0.5 0.0 -0
f1 (ppm)

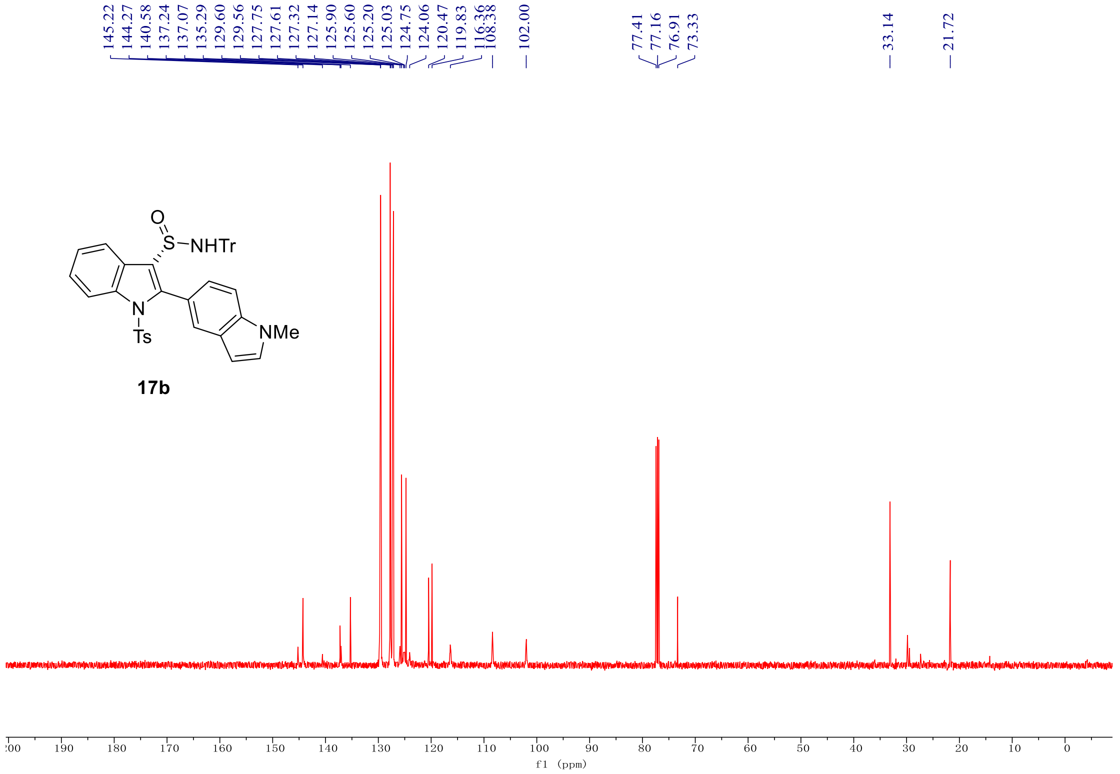
145.22
144.27
140.58
137.24
137.07
135.29
129.60
129.56
127.75
127.61
127.32
127.14
125.90
125.60
125.20
125.03
124.75
124.06
120.47
119.83
116.36
108.38
102.00
77.41
77.16
76.91
73.33
33.14
21.72
S
O
NHTr
N
Ts
NMe
17b
f1 (ppm)

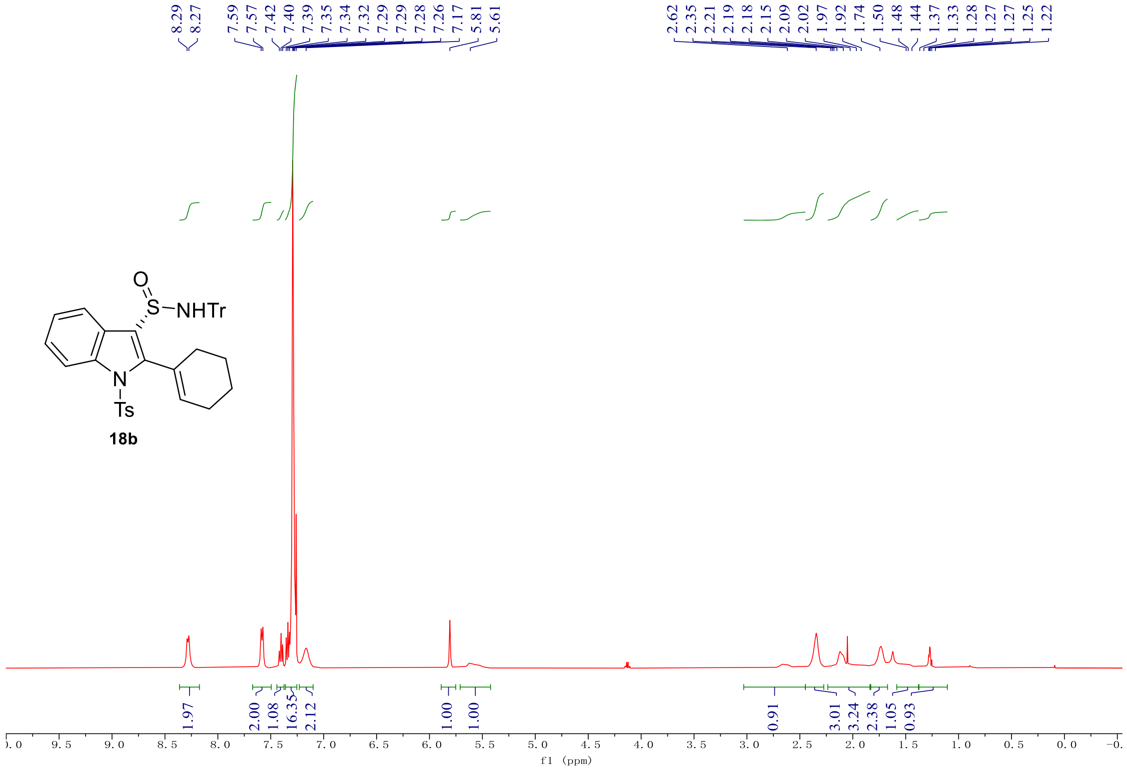
8.29
8.27
7.59
7.57
7.42
7.40
7.39
7.35
7.34
7.32
7.29
7.29
7.28
7.26
7.17
5.81
5.61
2.62
2.35
2.21
2.19
2.18
2.15
2.09
2.02
1.97
1.92
1.74
1.50
1.48
1.44
1.37
1.33
1.28
1.27
1.27
1.25
1.22
S
O
NHTr
N
Ts
18b
1.97
2.00
1.08
16.35
2.12
1.00
1.00
0.91
3.01
3.24
2.38
1.05
0.93
f1 (ppm)

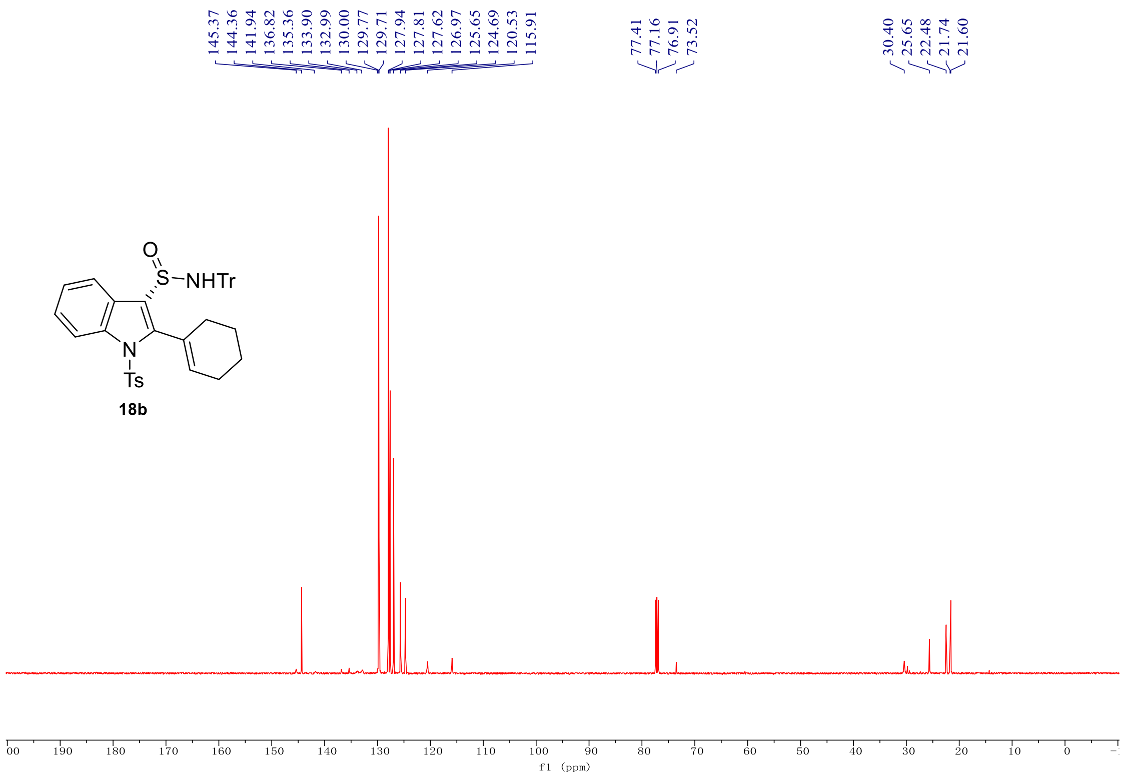
145.37 144.36 141.94 136.82 135.36 133.90 132.99 130.00 129.77 129.71 127.94 127.81 127.62 126.97 125.65 124.69 120.53 115.91
77.41 77.16 76.91 73.52
30.40 25.65 22.48 21.74 21.60
O
S
NHTr
N
Ts
18b
f1 (ppm)

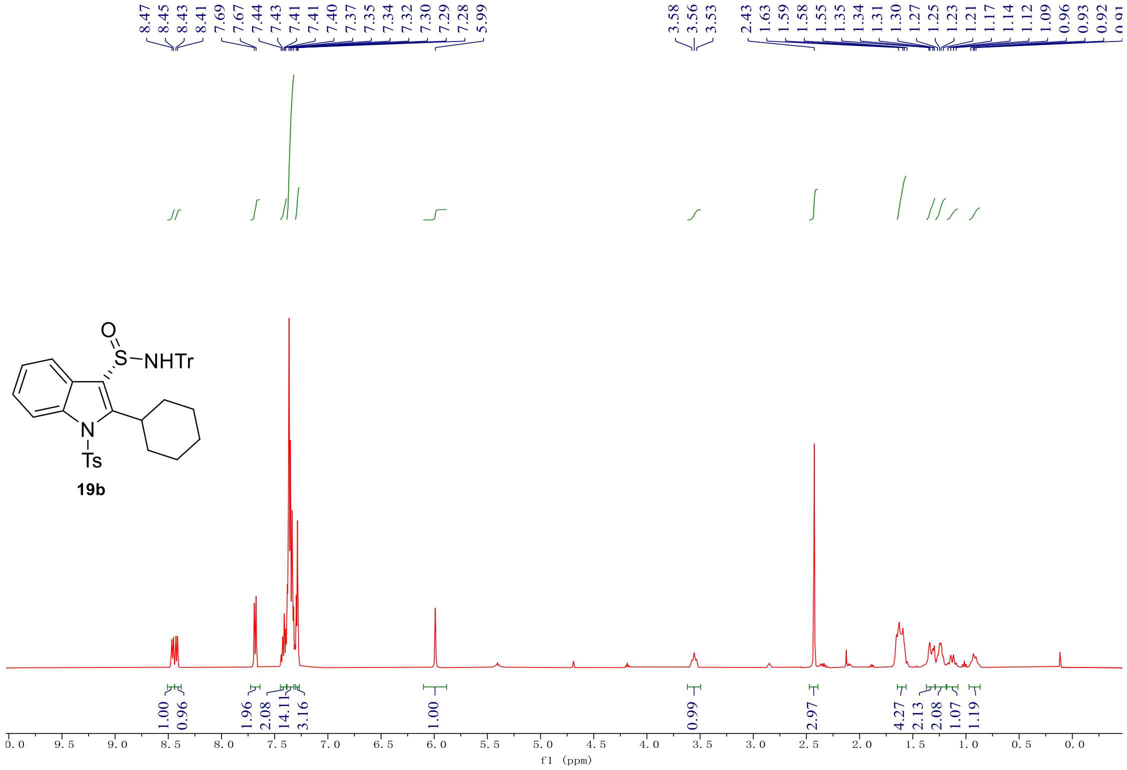
8.47 8.45 8.43 8.41 7.69 7.67 7.44 7.43 7.41 7.41 7.40 7.37 7.35 7.34 7.32 7.30 7.29 7.28 5.99
3.58 3.56 3.53
2.43 1.63 1.59 1.58 1.55 1.35 1.34 1.31 1.30 1.27 1.25 1.23 1.21 1.17 1.14 1.12 1.09 0.96 0.93 0.92
O
S
NHTr
N
Ts
19b
1.00 0.96 1.96 2.08 14.11 3.16 1.00 0.99 2.97 4.27 2.13 2.08 1.07 1.19
f1 (ppm)

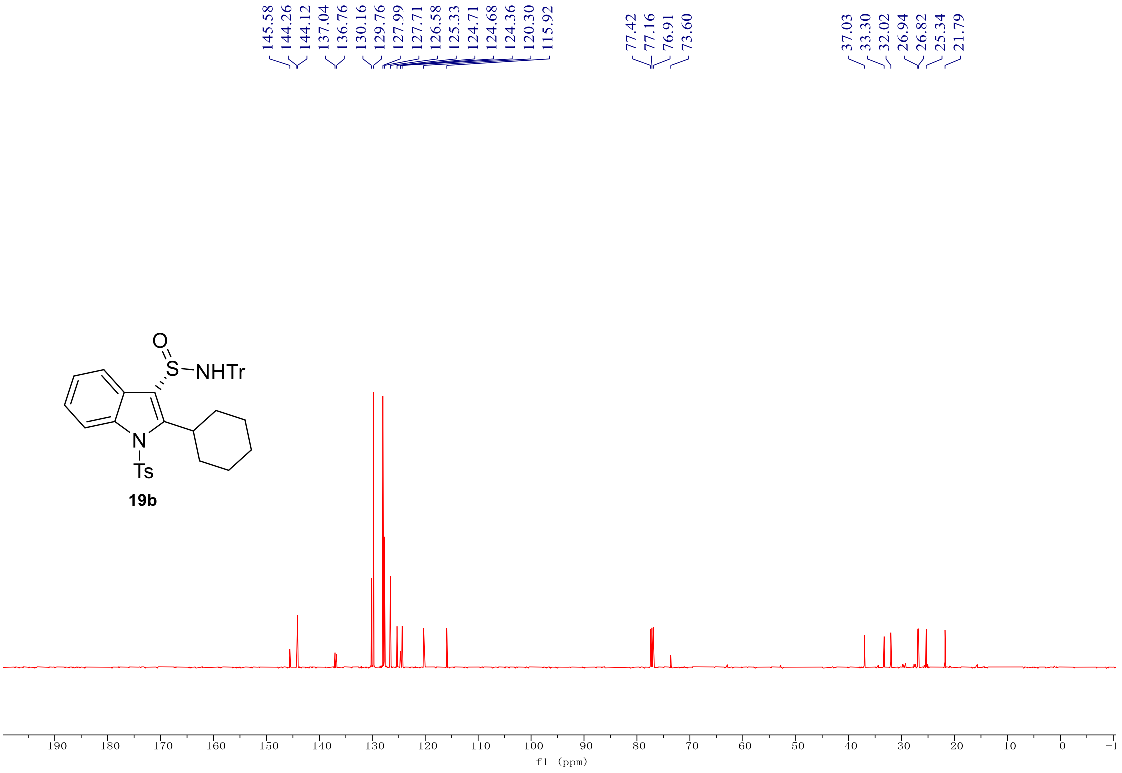

145.58
144.26
144.12
137.04
136.76
130.16
129.76
127.99
127.71
126.58
125.33
124.71
124.68
124.36
120.30
115.92
77.42
77.16
76.91
73.60
37.03
33.30
32.02
26.94
26.82
25.34
21.79
O
S
NHTr
N
Ts
19b
f1 (ppm)

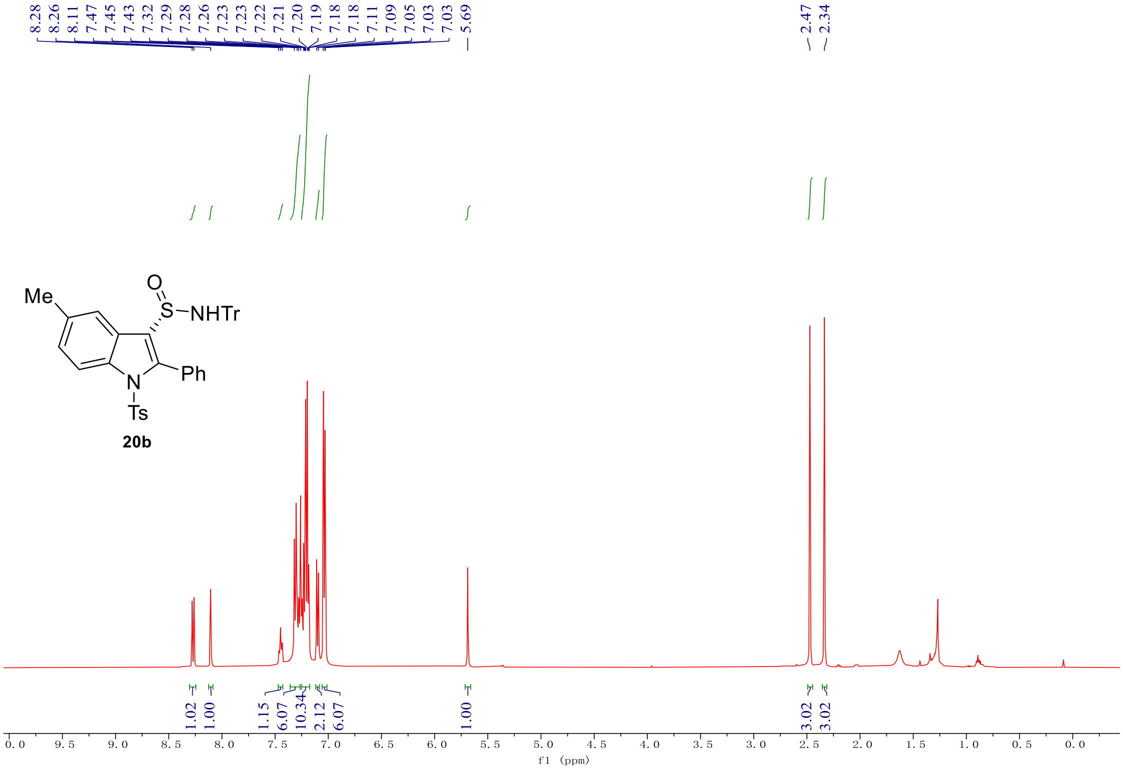

8.28
8.26
8.11
7.47
7.45
7.43
7.32
7.29
7.28
7.26
7.23
7.23
7.22
7.21
7.20
7.19
7.18
7.18
7.11
7.09
7.05
7.03
7.03
5.69
2.47
2.34
Me
O
S
NHTr
Ph
N
Ts
20b
1.02
1.00
1.15
6.07
10.34
2.12
6.07
1.00
3.02
3.02
f1 (ppm)

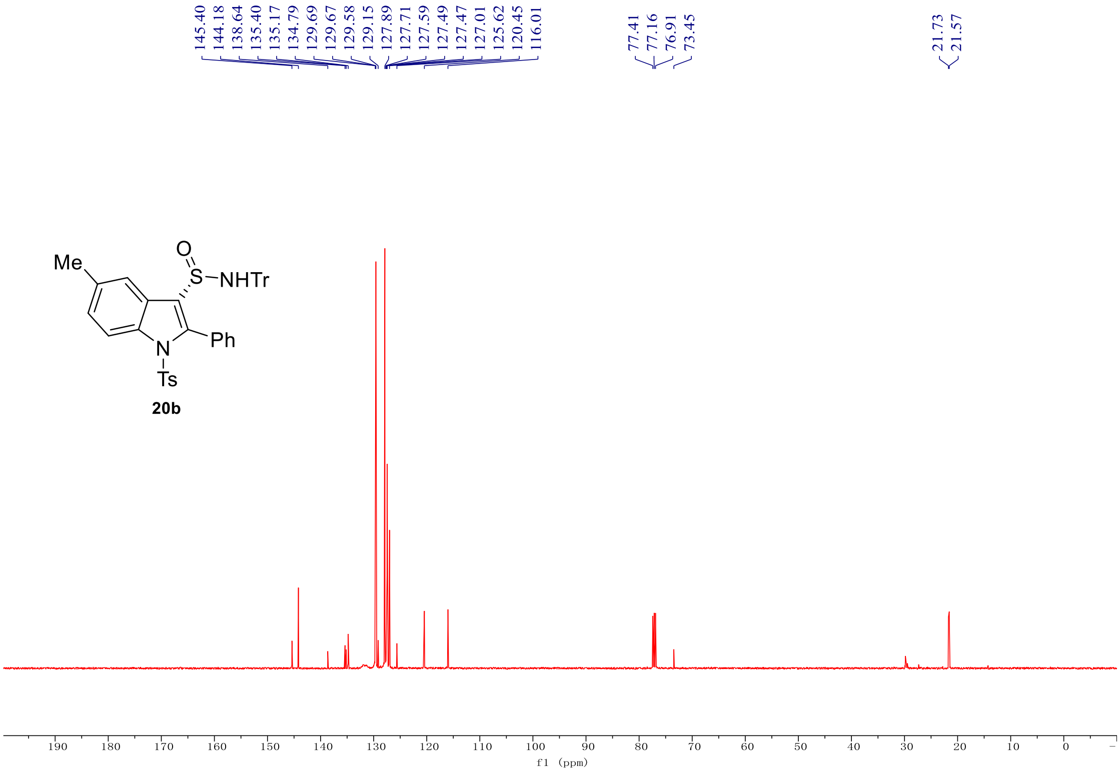
145.40
144.18
138.64
135.40
135.17
134.79
129.69
129.67
129.58
129.15
127.89
127.71
127.59
127.49
127.47
127.01
125.62
120.45
116.01
77.41
77.16
76.91
73.45
21.73
21.57
Me
O
S
NHTr
Ph
N
Ts
20b
190 180 170 160 150 140 130 120 110 100 90 80 70 60 50 40 30 20 10 0
f1 (ppm)

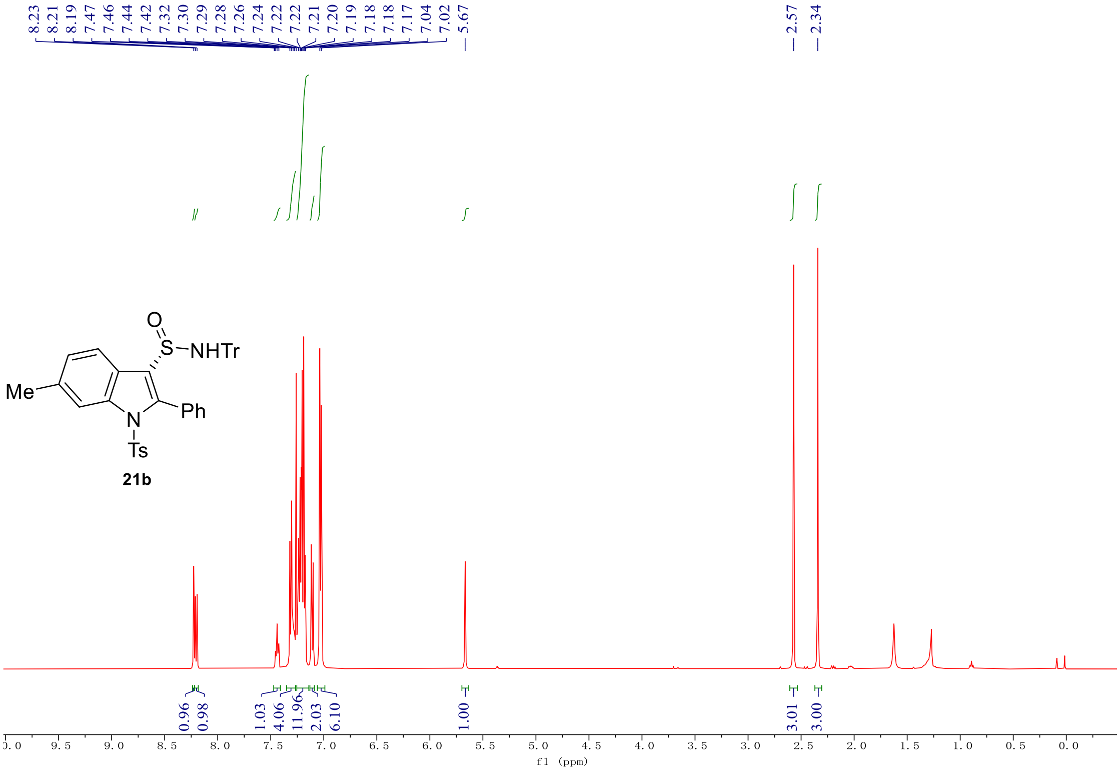
8.23
8.21
8.19
7.47
7.46
7.44
7.42
7.32
7.30
7.29
7.28
7.26
7.24
7.22
7.22
7.21
7.20
7.19
7.18
7.17
7.04
7.02
5.67
2.57
2.34
O
S
NHTr
Me
Ph
N
Ts
21b
0.96
0.98
1.03
4.06
11.96
2.03
6.10
1.00
3.01
3.00
9.5 9.0 8.5 8.0 7.5 7.0 6.5 6.0 5.5 5.0 4.5 4.0 3.5 3.0 2.5 2.0 1.5 1.0 0.5 0.0
f1 (ppm)

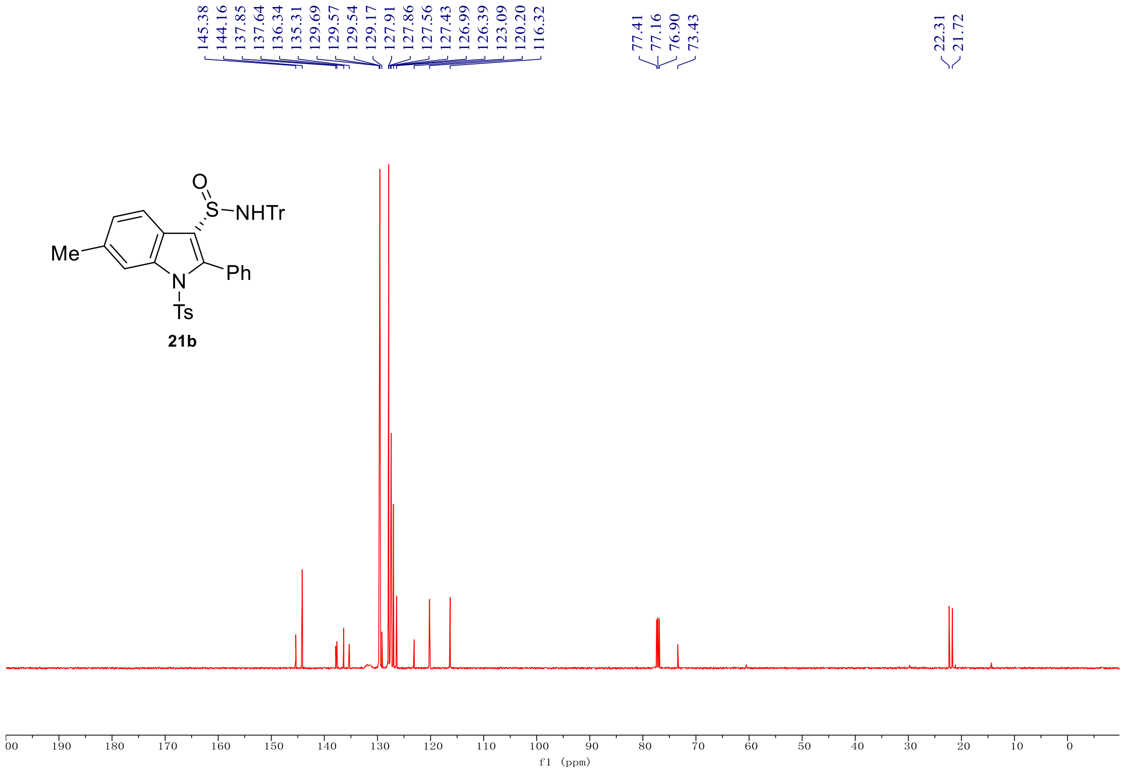
145.38
144.16
137.85
137.64
136.34
135.31
129.69
129.57
129.54
129.17
127.91
127.86
127.56
127.43
126.99
126.39
123.09
120.20
116.32
77.41
77.16
76.90
73.43
22.31
21.72
O
S
NHTr
Me
N
Ph
Ts
21b
f1 (ppm)

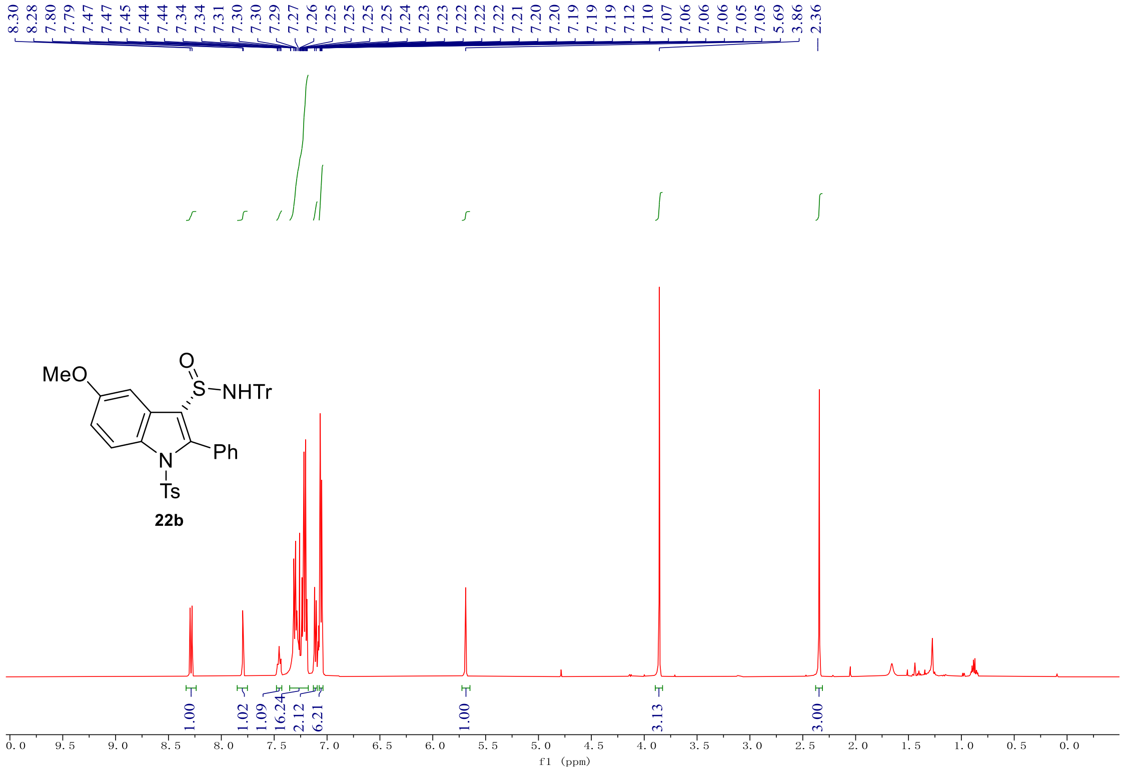
8.30
8.28
7.80
7.79
7.47
7.45
7.44
7.34
7.31
7.30
7.29
7.27
7.26
7.25
7.25
7.25
7.24
7.23
7.23
7.22
7.22
7.22
7.21
7.20
7.19
7.19
7.12
7.10
7.07
7.06
7.06
7.05
7.05
5.69
3.86
2.36
O
S
NHTr
MeO
N
Ph
Ts
22b
1.00
1.02
1.09
16.24
2.12
6.21
1.00
3.13
3.00
f1 (ppm)

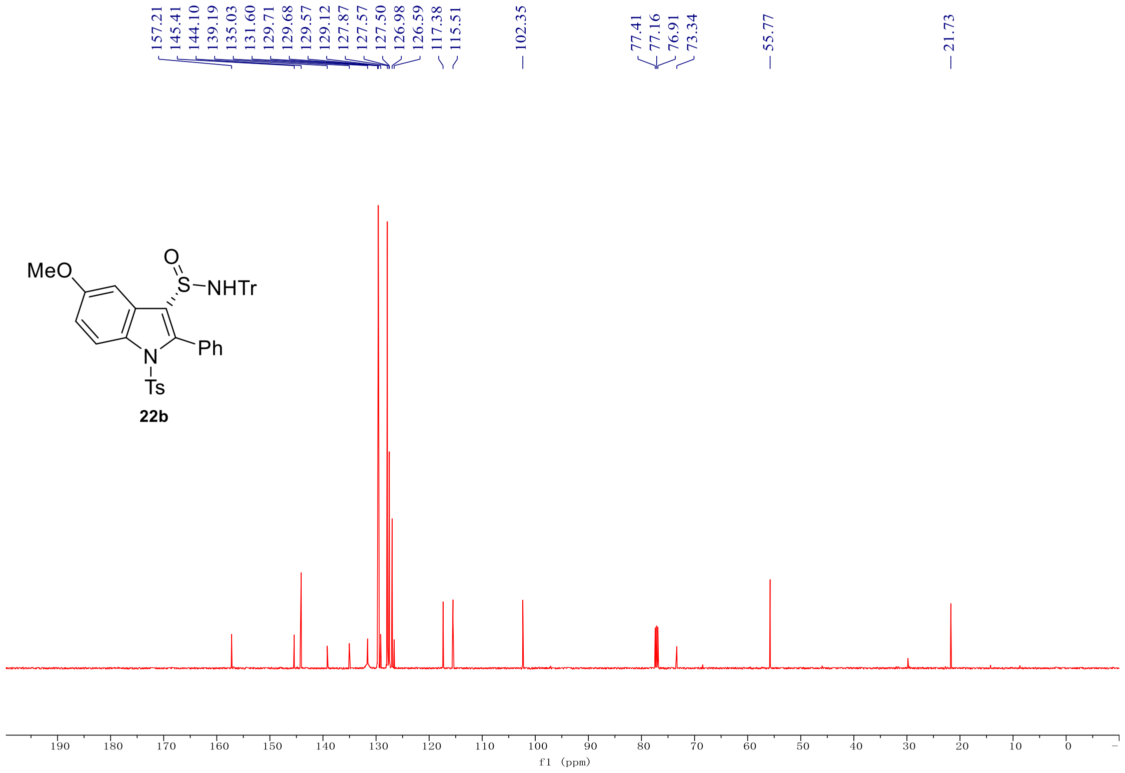
157.21
145.41
144.10
139.19
135.03
131.60
129.71
129.68
129.57
129.12
127.87
127.57
127.50
126.98
126.59
117.38
115.51
102.35
77.41
77.16
76.91
73.34
55.77
21.73
f1 (ppm)
MeO
O
S
NHTr
N
Ph
Ts

22b

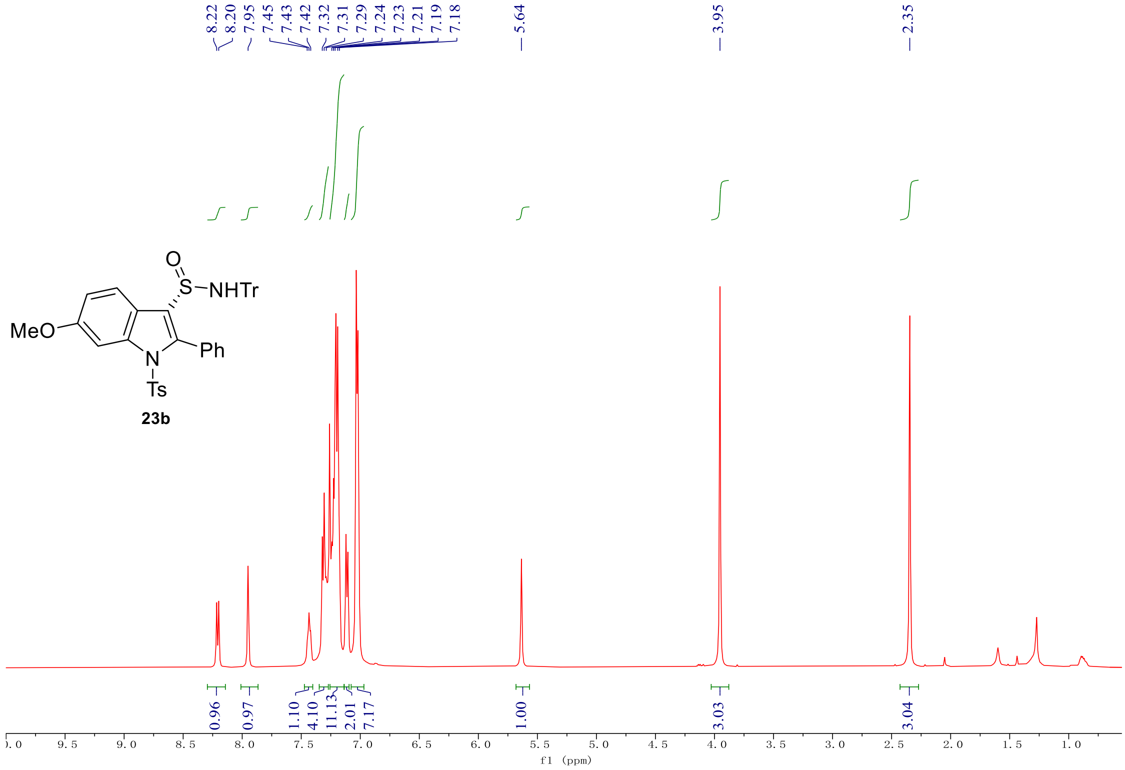
8.22
8.20
7.95
7.45
7.43
7.42
7.32
7.31
7.29
7.24
7.23
7.21
7.19
7.18
5.64
3.95
2.35
0.96
0.97
1.10
4.10
11.13
2.01
7.17
1.00
3.03
3.04
f1 (ppm)
O
S
NHTr
MeO
N
Ph
Ts

23b

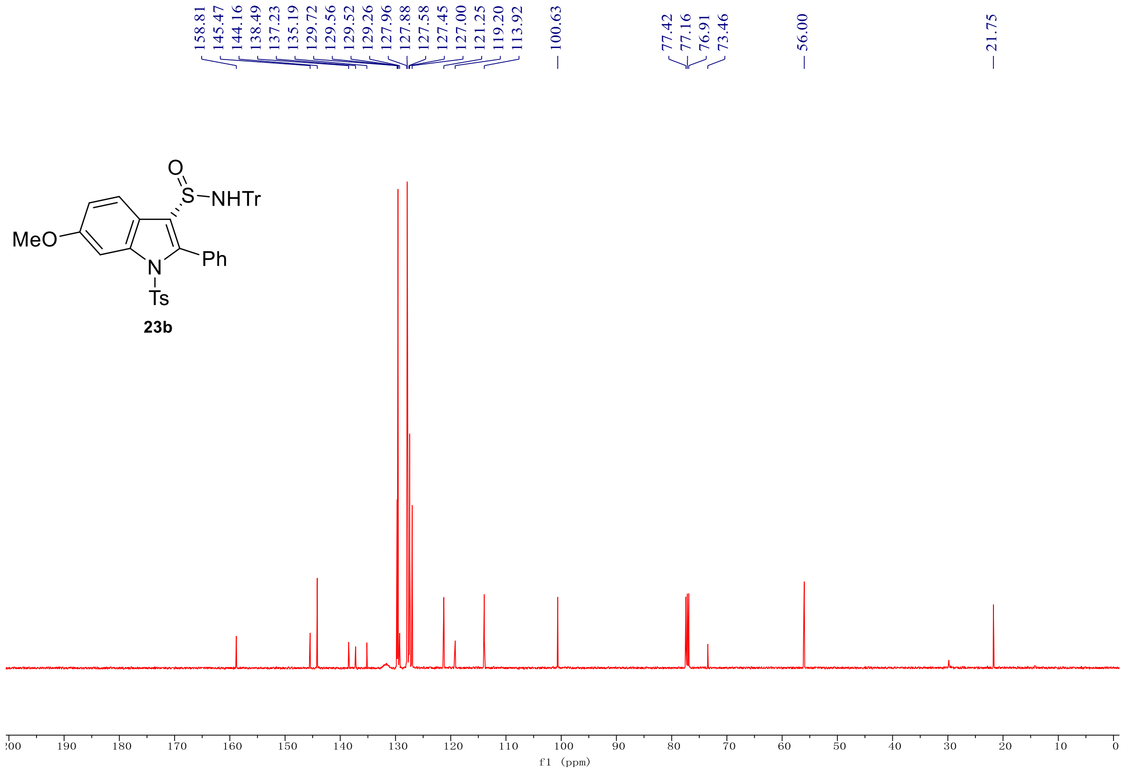
158.81
145.47
144.16
138.49
137.23
135.19
129.72
129.56
129.52
129.26
127.96
127.88
127.58
127.45
127.00
121.25
119.20
113.92
100.63
77.42
77.16
76.91
73.46
56.00
21.75
O
S
NHTr
MeO
N
Ph
Ts
23b
f1 (ppm)

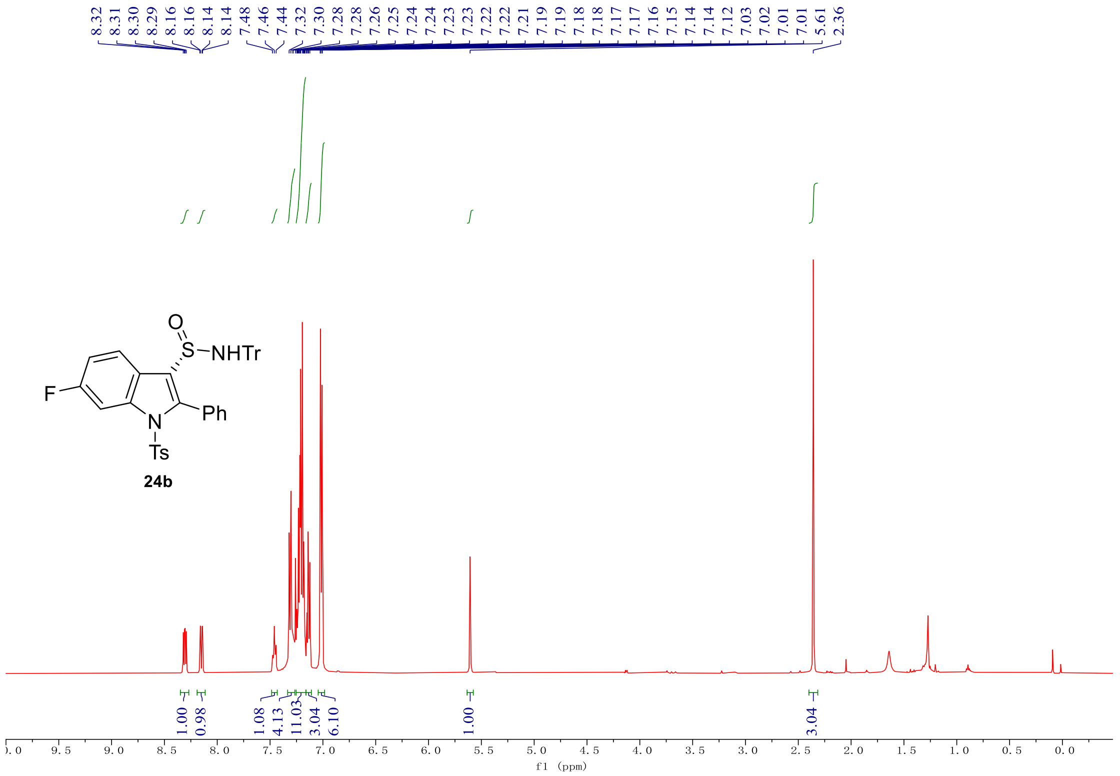
8.32
8.31
8.30
8.29
8.16
8.16
8.14
8.14
7.48
7.46
7.44
7.32
7.30
7.28
7.28
7.26
7.25
7.24
7.24
7.23
7.23
7.22
7.22
7.21
7.19
7.19
7.18
7.18
7.17
7.17
7.16
7.15
7.14
7.14
7.12
7.03
7.02
7.01
7.01
5.61
2.36
O
S
NHTr
F
N
Ph
Ts
24b
1.00
0.98
1.08
4.13
11.03
3.04
6.10
1.00
3.04
f1 (ppm)

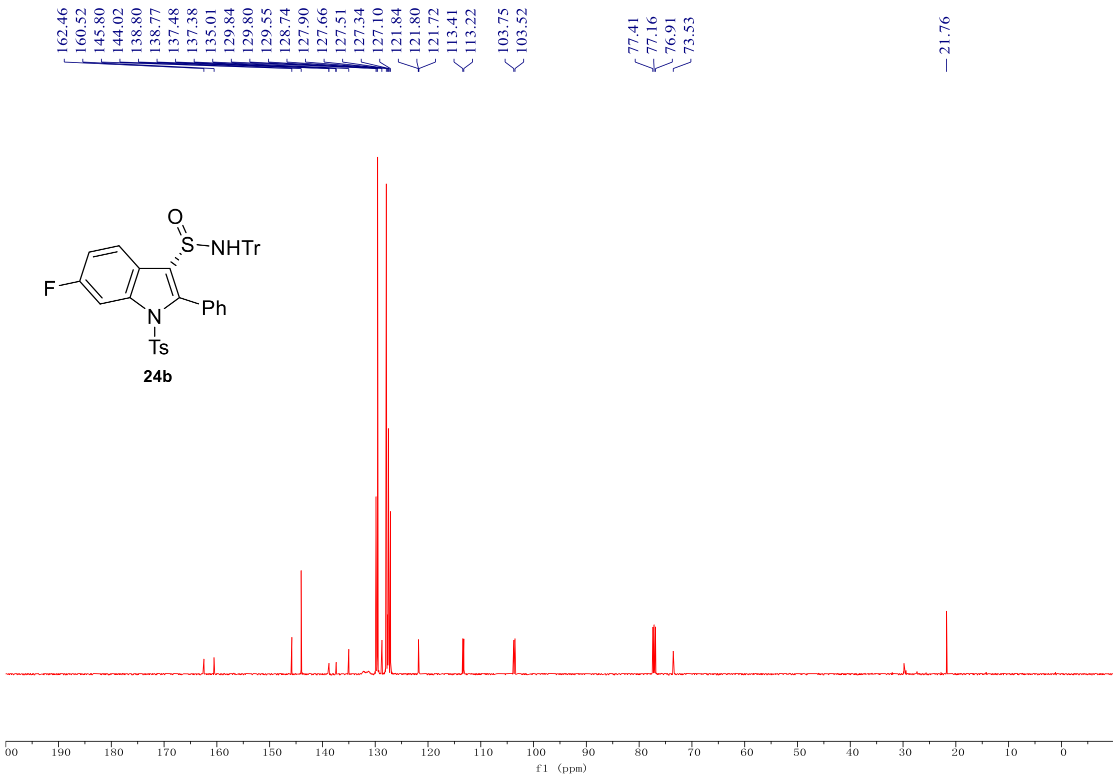
162.46
160.52
145.80
144.02
138.80
138.77
137.48
137.38
135.01
129.84
129.80
129.55
128.74
127.90
127.66
127.51
127.34
127.10
121.84
121.80
121.72
113.41
113.22
103.75
103.52
77.41
77.16
76.91
73.53
21.76
O
S
NHTr
F
N
Ph
Ts
24b
f1 (ppm)

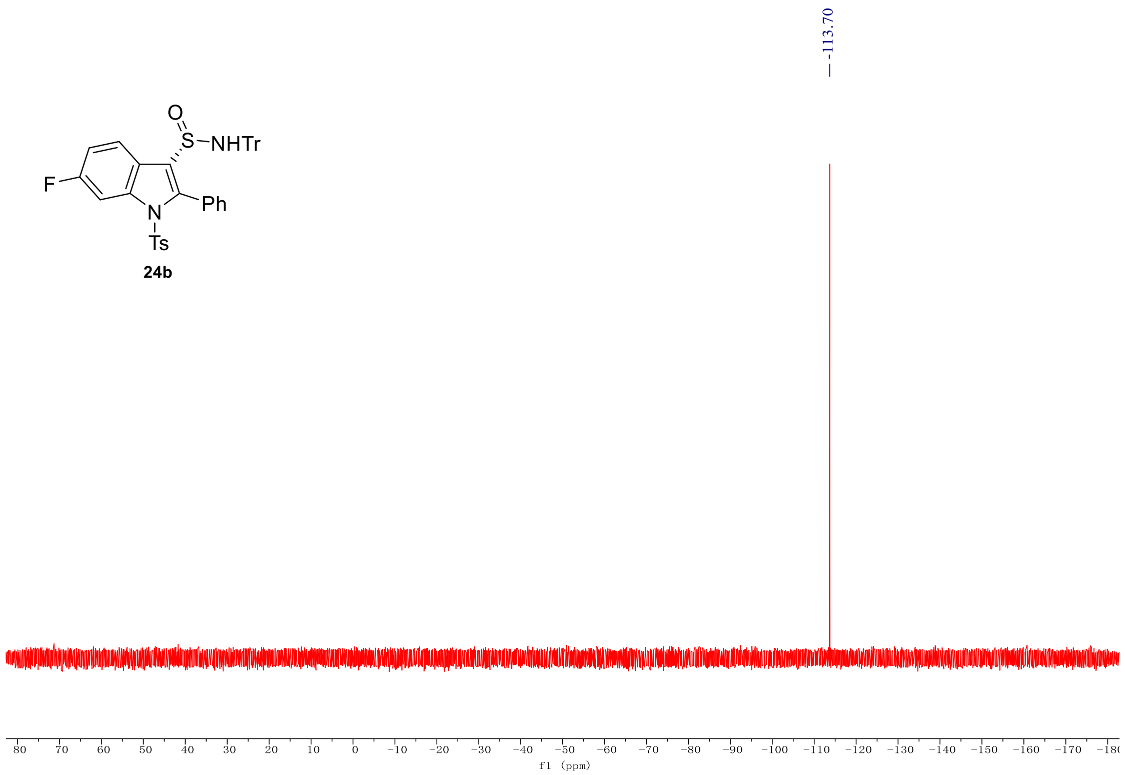
-113.70
O
S
NHTr
F
N
Ph
Ts
24b
f1 (ppm)

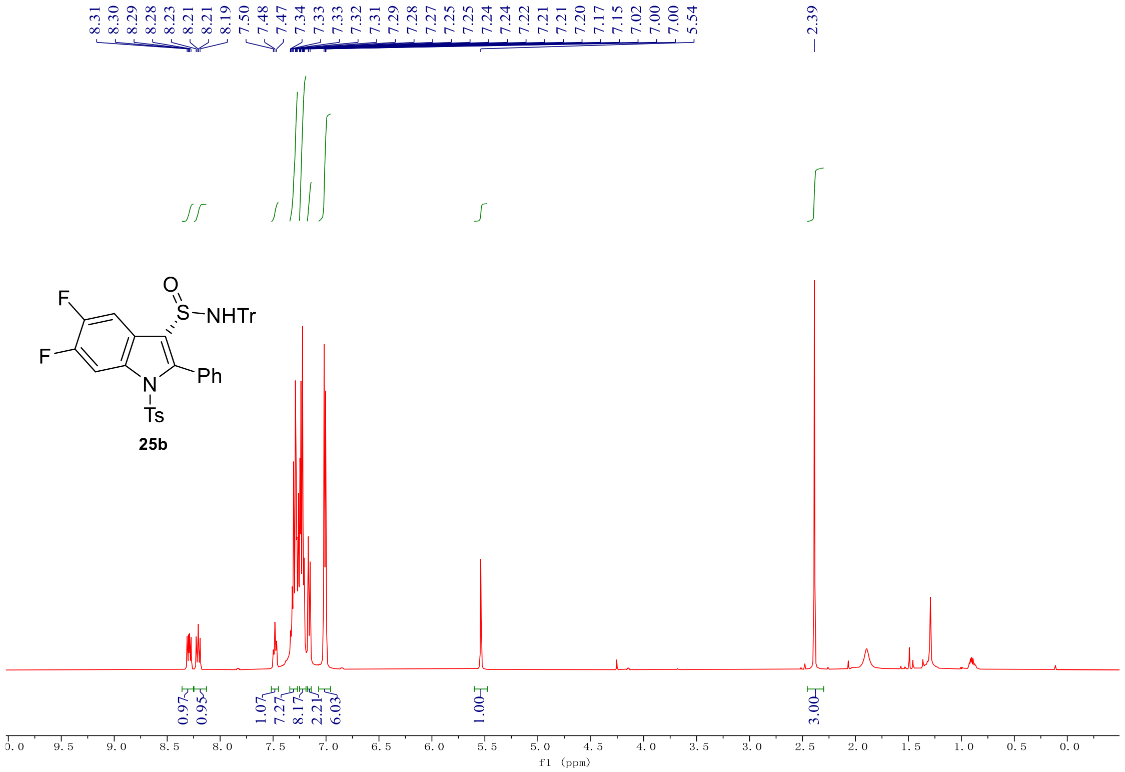
8.31 8.30 8.29 8.28 8.23 8.21 8.21 8.19 7.50 7.48 7.47 7.34 7.33 7.33 7.32 7.31 7.29 7.28 7.27 7.25 7.25 7.24 7.24 7.22 7.21 7.21 7.20 7.17 7.15 7.02 7.00 7.00 5.54
2.39
F
F
O
S
NHTr
Ph
N
Ts
25b
0.97 0.95 1.07 7.27 8.17 2.21 6.03 1.00 3.00
f1 (ppm)

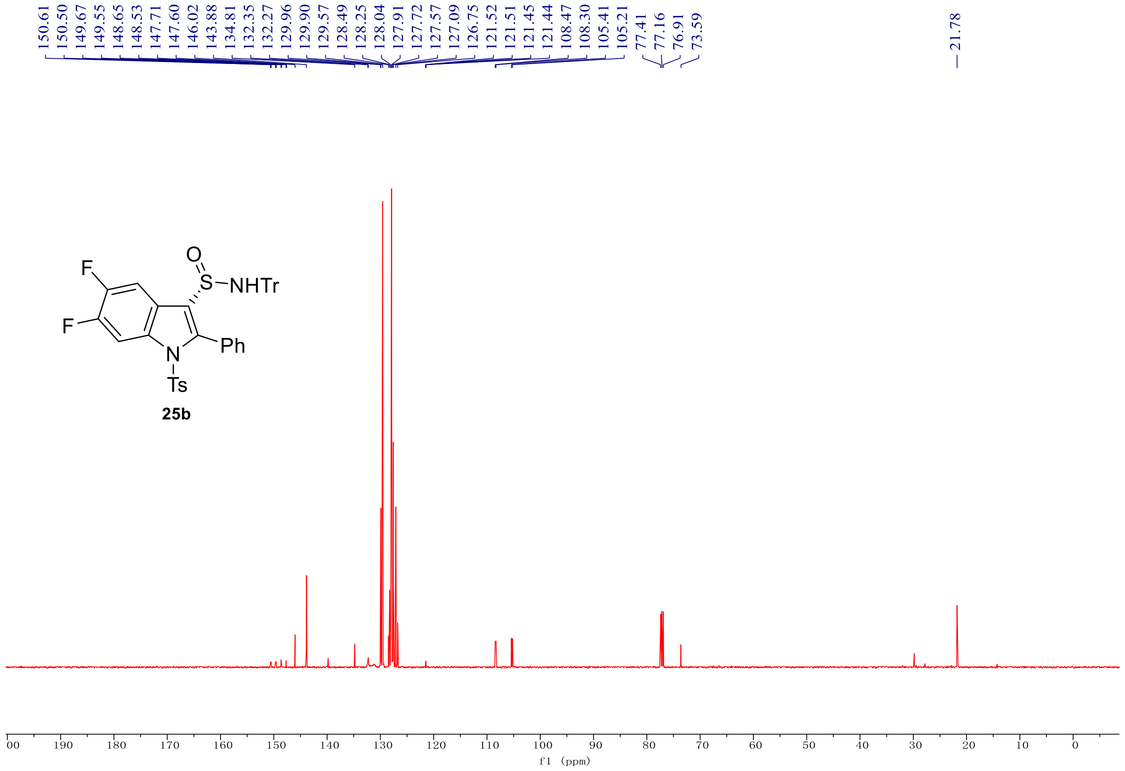
150.61 150.50 149.67 149.55 148.65 148.53 147.71 147.60 146.02 143.88 134.81 132.35 132.27 129.96 129.90 129.57 128.49 128.25 128.04 127.91 127.72 127.57 127.09 126.75 121.52 121.51 121.45 121.44 108.47 108.30 105.41 105.21 77.41 77.16 76.91 73.59
21.78
F
F
O
S
NHTr
Ph
N
Ts
25b
f1 (ppm)

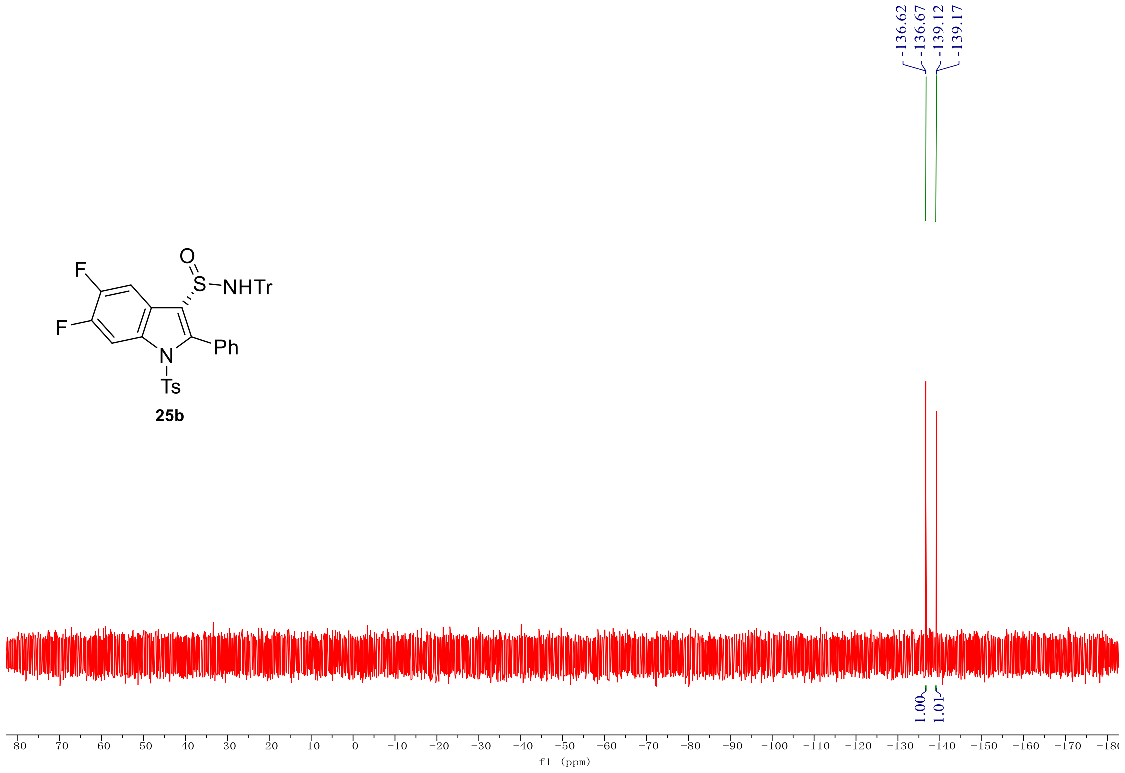

-136.62
-136.67
-139.12
-139.17
F
F
O
S
NHTr
Ph
N
Ts
25b
1.00
1.01
80 70 60 50 40 30 20 10 0 −10 −20 −30 −40 −50 −60 −70 −80 −90 −100 −110 −120 −130 −140 −150 −160 −170
f1 (ppm)

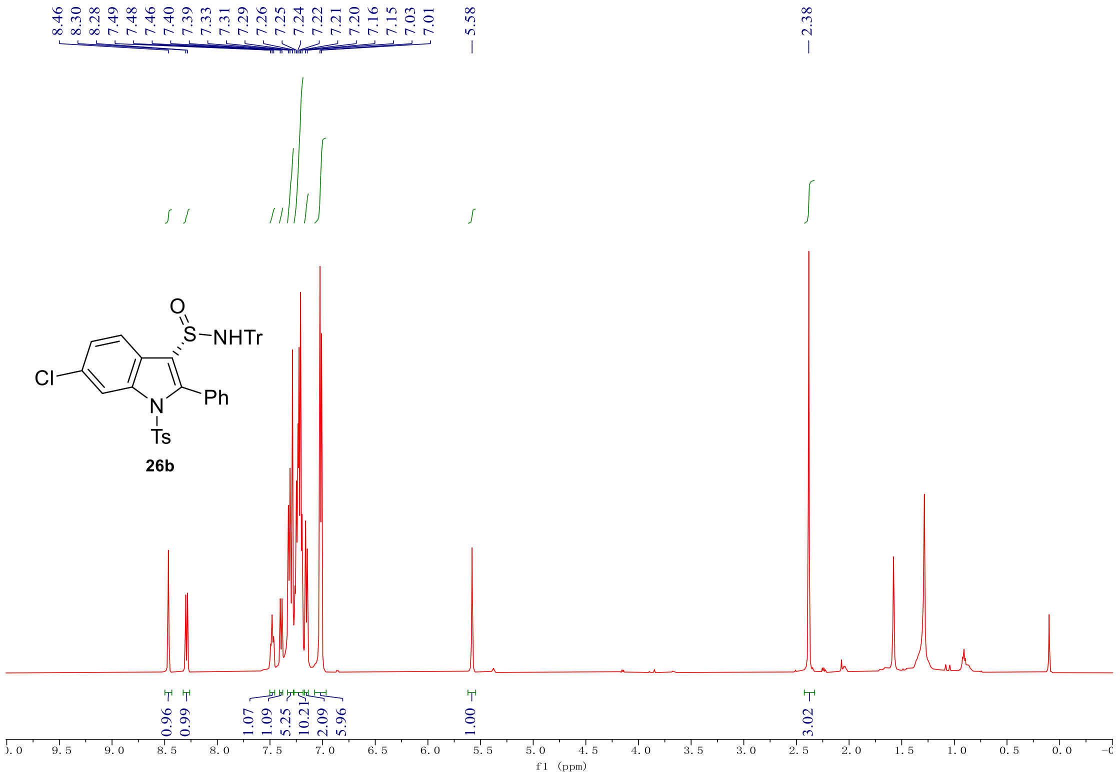

8.46
8.30
8.28
7.49
7.48
7.46
7.40
7.39
7.33
7.31
7.29
7.26
7.25
7.24
7.22
7.21
7.20
7.16
7.15
7.03
7.01
5.58
2.38
O
S
NHTr
Cl
Ph
N
Ts
26b
0.96
0.99
1.07
1.09
5.25
10.21
2.09
5.96
1.00
3.02
9.5 9.0 8.5 8.0 7.5 7.0 6.5 6.0 5.5 5.0 4.5 4.0 3.5 3.0 2.5 2.0 1.5 1.0 0.5 0.0
f1 (ppm)

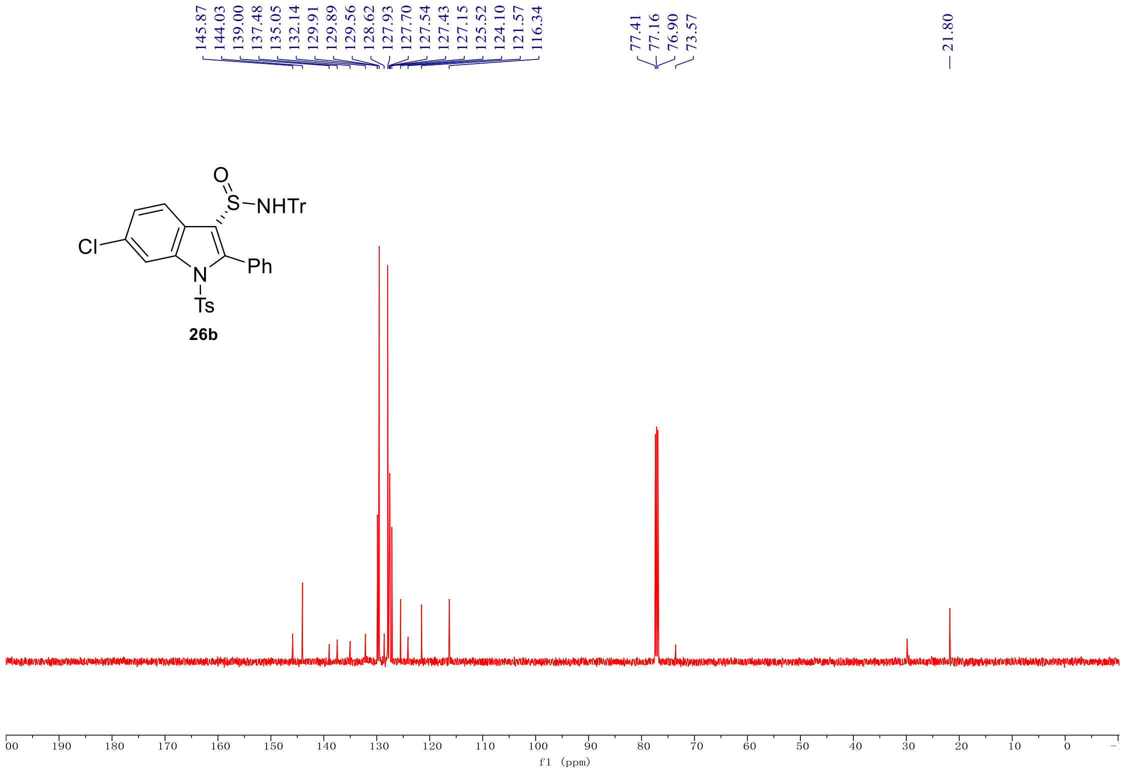

145.87
144.03
139.00
137.48
135.05
132.14
129.91
129.89
129.56
128.62
127.93
127.70
127.54
127.43
127.15
125.52
124.10
121.57
116.34
77.41
77.16
76.90
73.57
21.80
O
S
NHTr
Cl
N
Ph
Ts
26b
f1 (ppm)

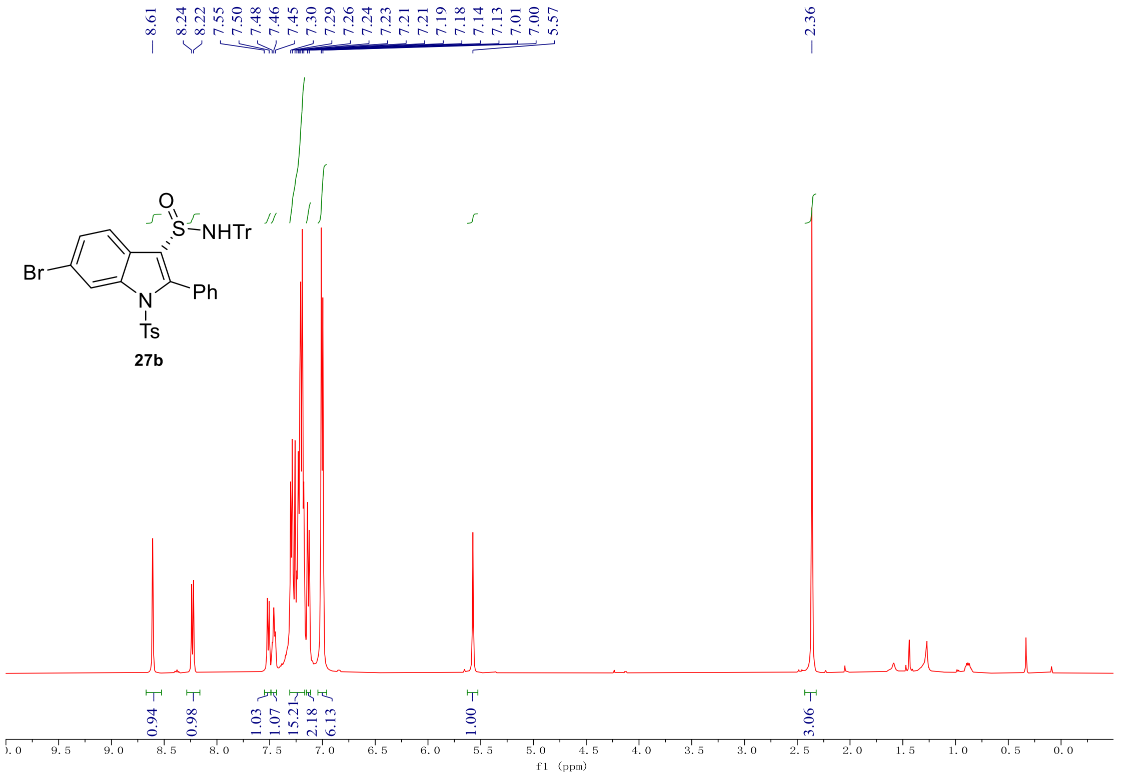

8.61
8.24
8.22
7.55
7.50
7.48
7.46
7.45
7.30
7.29
7.26
7.24
7.23
7.21
7.21
7.19
7.18
7.14
7.13
7.01
7.00
5.57
2.36
O
S
NHTr
Br
N
Ph
Ts
27b
0.94
0.98
1.03
1.07
15.21
2.18
6.13
1.00
3.06
f1 (ppm)

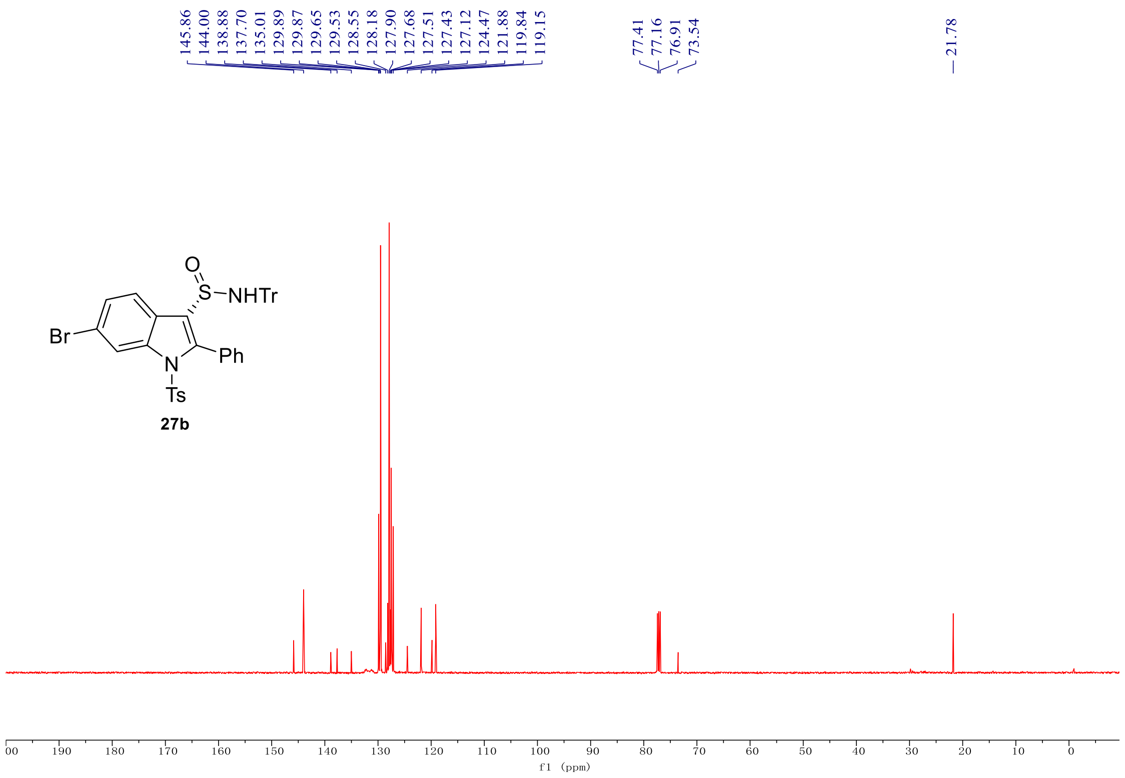

145.86
144.00
138.88
137.70
135.01
129.89
129.87
129.65
129.53
128.55
128.18
127.90
127.68
127.51
127.43
127.12
124.47
121.88
119.84
119.15
77.41
77.16
76.91
73.54
21.78
O
S
NHTr
Br
N
Ph
Ts
27b
f1 (ppm)

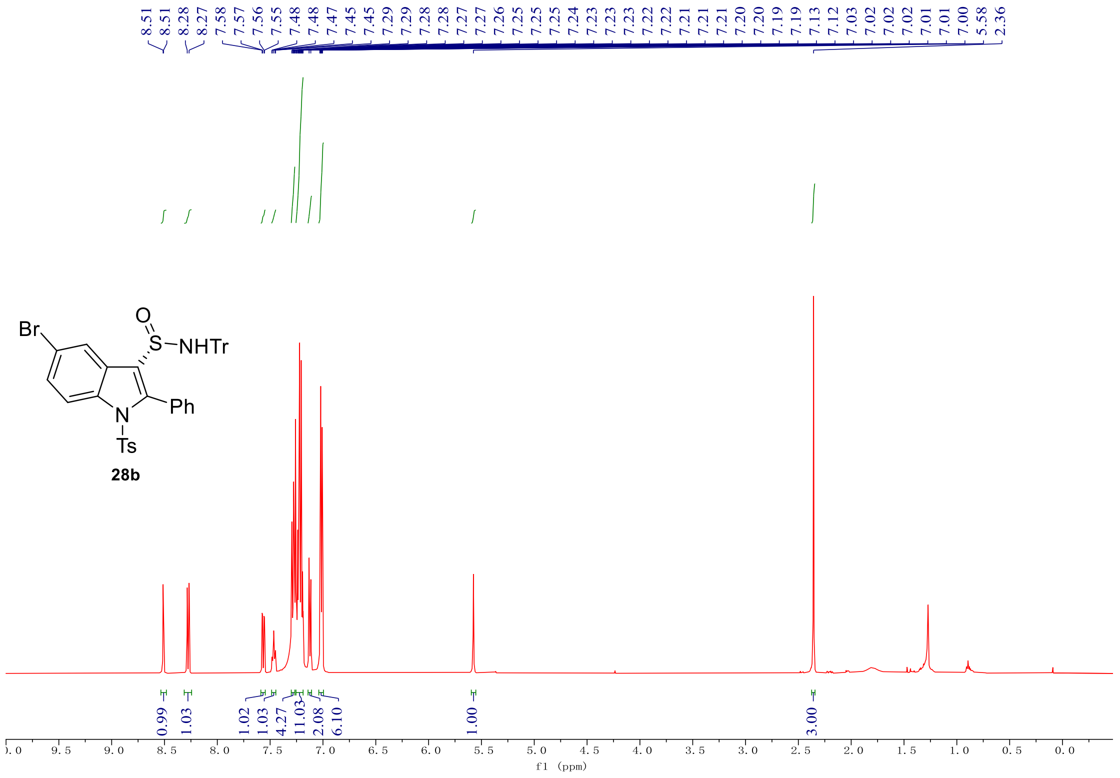

8.51
8.51
8.28
8.27
7.58
7.57
7.56
7.55
7.48
7.48
7.47
7.45
7.45
7.29
7.29
7.28
7.28
7.27
7.27
7.26
7.25
7.25
7.25
7.24
7.23
7.23
7.23
7.22
7.22
7.21
7.21
7.21
7.20
7.19
7.19
7.13
7.12
7.03
7.02
7.02
7.02
7.01
7.01
7.00
5.58
2.36
Br
O
S
NHTr
N
Ph
Ts
28b
0.99
1.03
1.02
1.03
4.27
11.03
2.08
6.10
1.00
3.00
f1 (ppm)

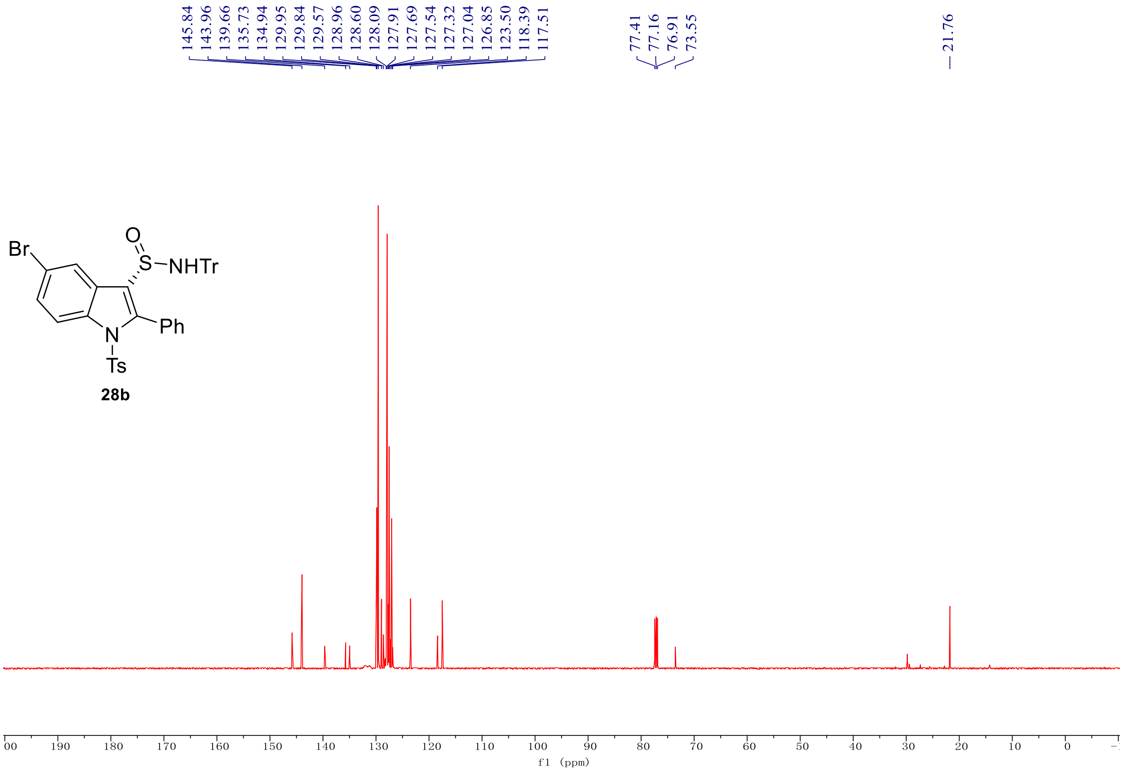
145.84
143.96
139.66
135.73
134.94
129.95
129.84
129.57
128.96
128.60
128.09
127.91
127.69
127.54
127.32
127.04
126.85
123.50
118.39
117.51
77.41
77.16
76.91
73.55
21.76
Br
O
S
NHTr
N
Ph
Ts
28b
f1 (ppm)

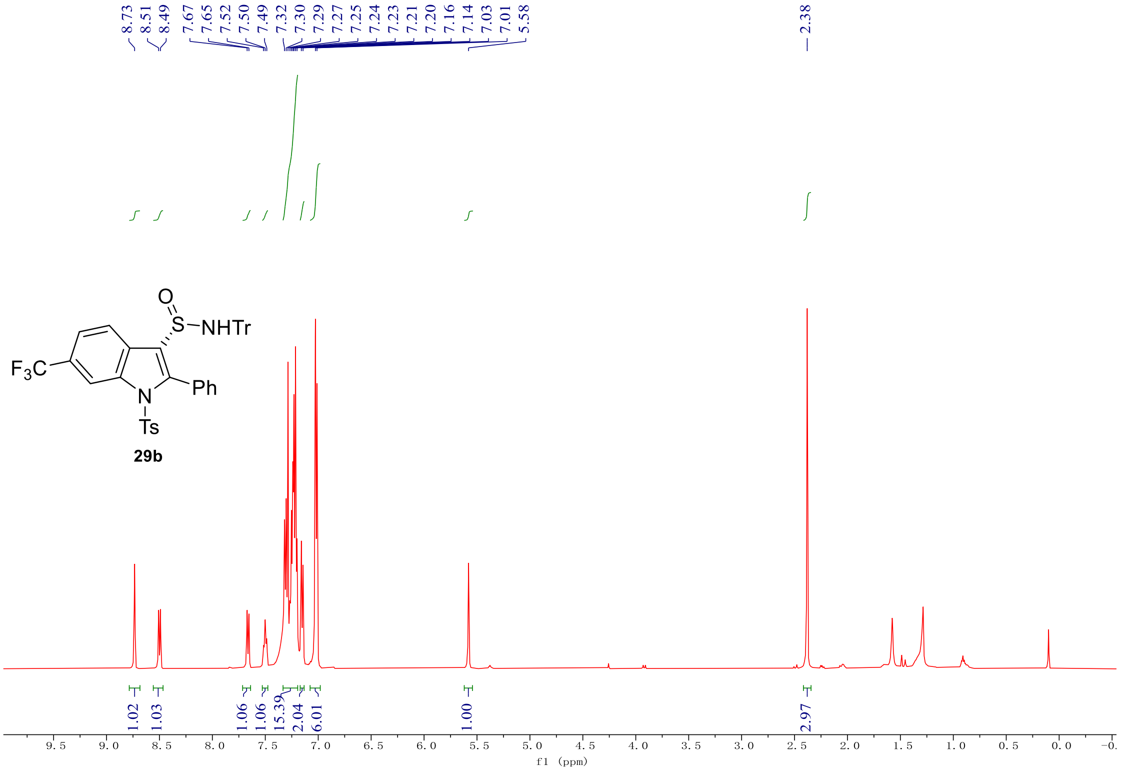
8.73
8.51
8.49
7.67
7.65
7.52
7.50
7.49
7.32
7.30
7.29
7.27
7.25
7.24
7.23
7.21
7.20
7.16
7.14
7.03
7.01
5.58
2.38
O
S
NHTr
F_3C
N
Ph
Ts
29b
1.02
1.03
1.06
1.06
15.39
2.04
6.01
1.00
2.97
f1 (ppm)

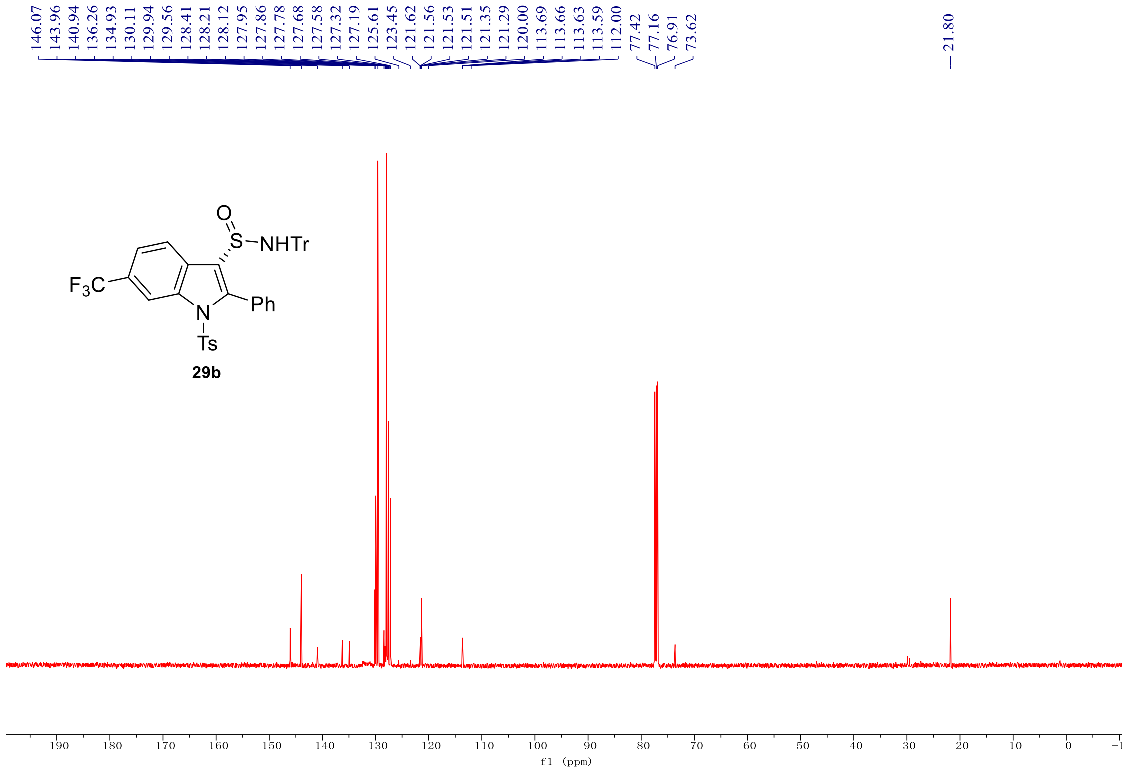
146.07
143.96
140.94
136.26
134.93
130.11
129.94
129.56
128.41
128.21
128.12
127.95
127.86
127.78
127.68
127.58
127.32
127.19
125.61
123.45
121.62
121.56
121.53
121.51
121.35
121.29
120.00
113.69
113.66
113.63
113.59
112.00
77.42
77.16
76.91
73.62
21.80
S–NHTr
F3C
Ph
N
Ts
29b
f1 (ppm)

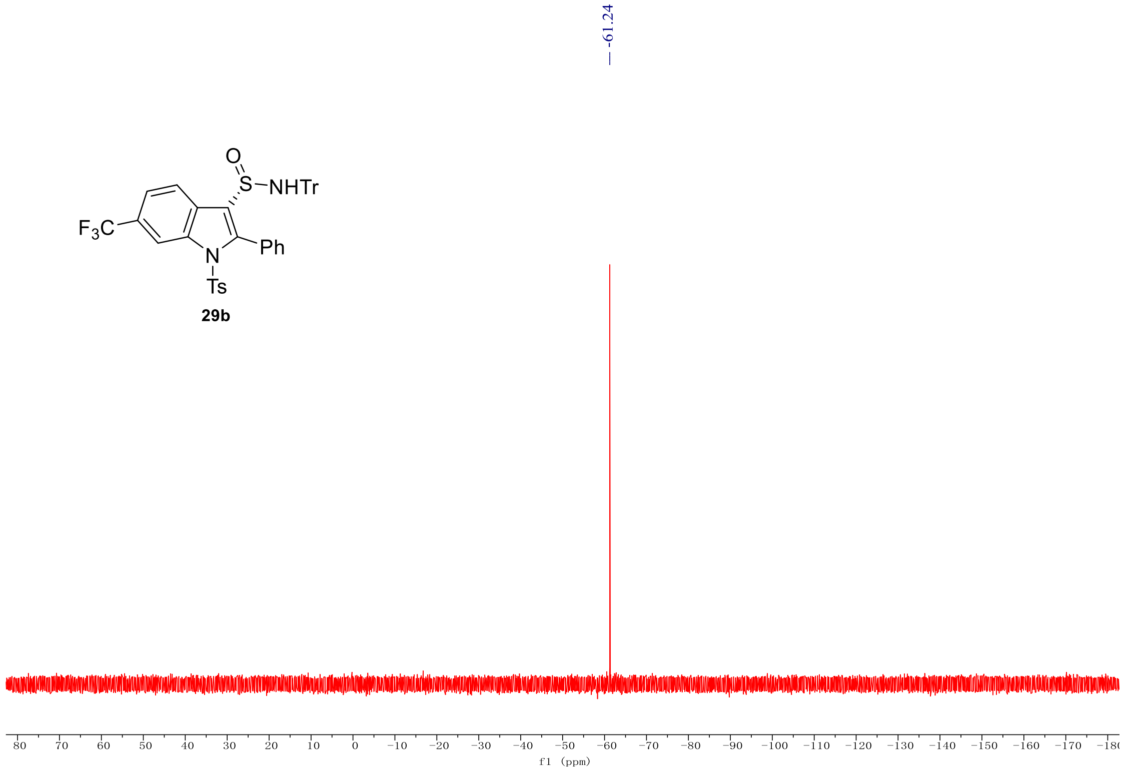
-61.24
S–NHTr
F3C
Ph
N
Ts
29b
f1 (ppm)

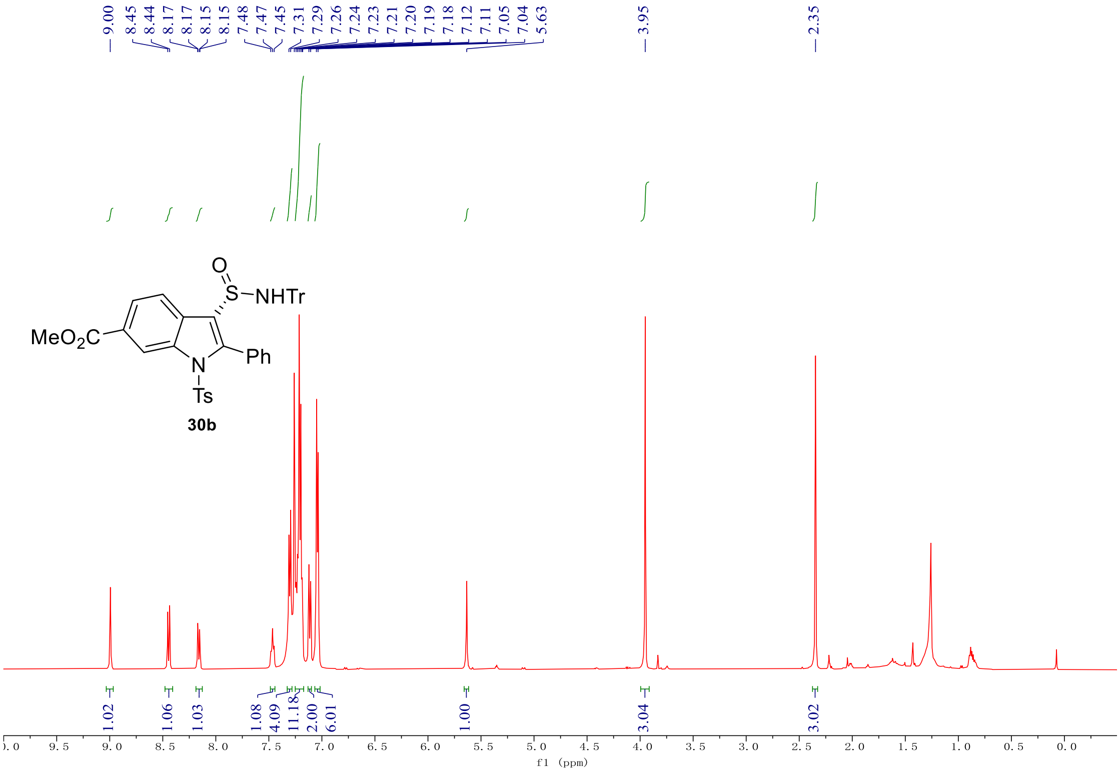

9.00
8.45
8.44
8.17
8.17
8.15
8.15
7.48
7.47
7.45
7.31
7.29
7.26
7.24
7.23
7.21
7.20
7.19
7.18
7.12
7.11
7.05
7.04
5.63
3.95
2.35
O
S
NHTr
MeO2C
Ph
N
Ts
30b
1.02
1.06
1.03
1.08
4.09
11.18
2.00
6.01
1.00
3.04
3.02
f1 (ppm)

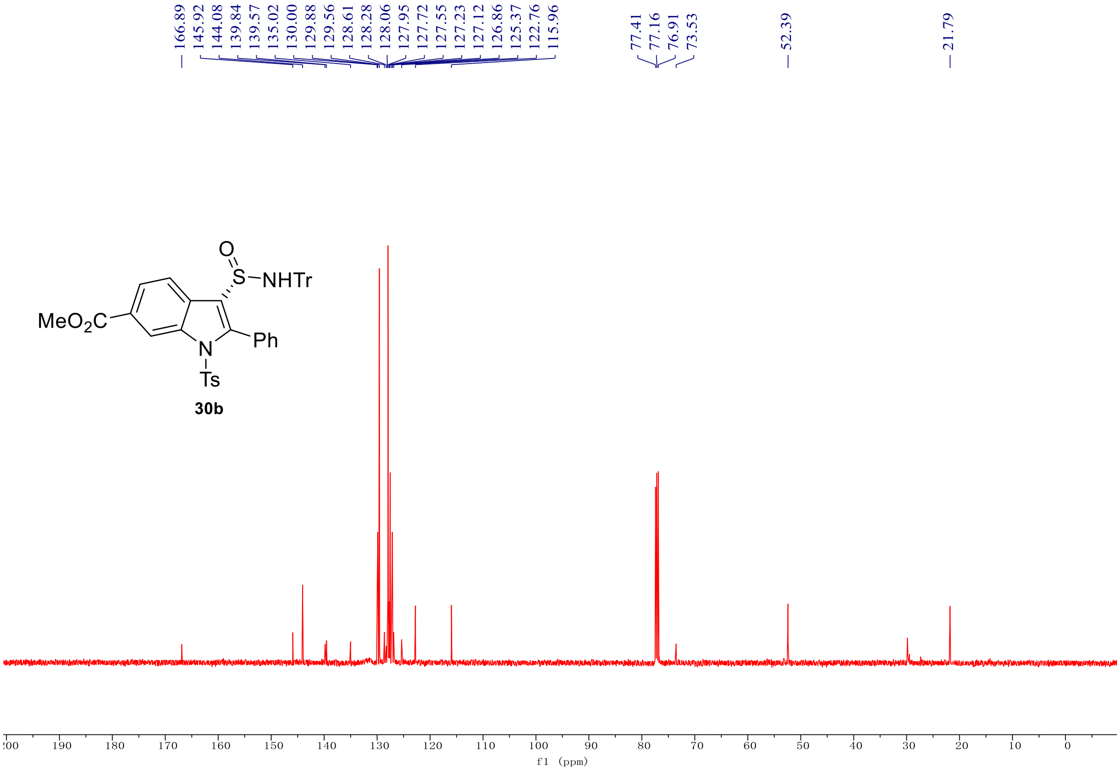

166.89
145.92
144.08
139.84
139.57
135.02
130.00
129.88
129.56
128.61
128.28
128.06
127.95
127.72
127.55
127.23
127.12
126.86
125.37
122.76
115.96
77.41
77.16
76.91
73.53
52.39
21.79
O
S
NHTr
MeO2C
Ph
N
Ts
30b
f1 (ppm)

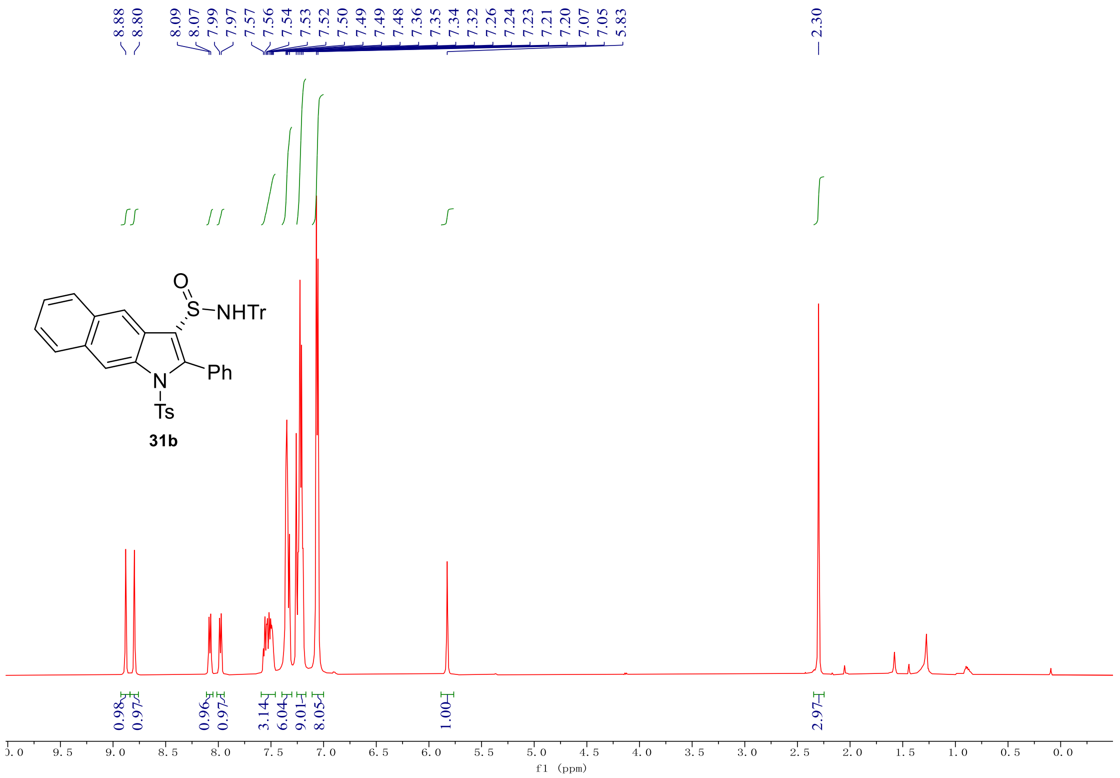
8.88
8.80
8.09
8.07
7.99
7.97
7.57
7.56
7.54
7.53
7.52
7.50
7.49
7.49
7.48
7.36
7.35
7.34
7.32
7.26
7.24
7.23
7.21
7.20
7.07
7.05
5.83
2.30
O
S
NHTr
Ph
N
Ts
31b
0.98
0.97
0.96
0.97
3.14
6.04
9.01
8.05
1.00
2.97
f1 (ppm)

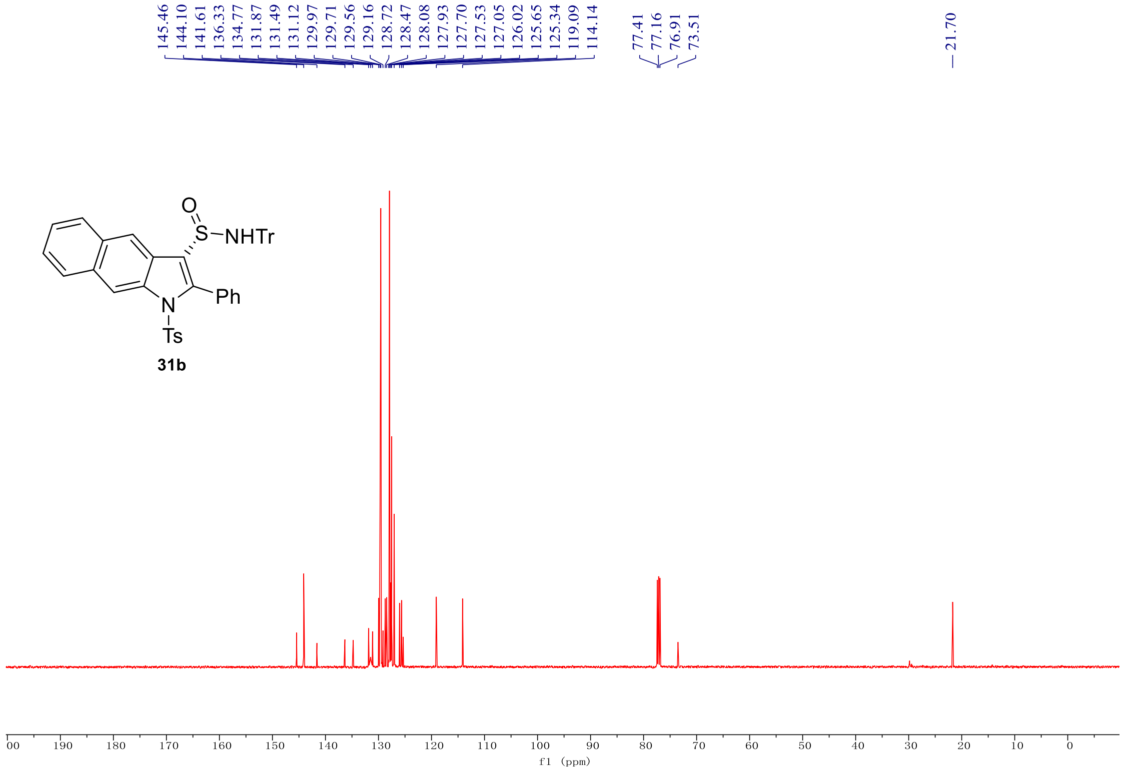
145.46
144.10
141.61
136.33
134.77
131.87
131.49
131.12
129.97
129.71
129.56
129.16
128.72
128.47
128.08
127.93
127.70
127.53
127.05
126.02
125.65
125.34
119.09
114.14
77.41
77.16
76.91
73.51
21.70
O
S
NHTr
Ph
N
Ts
31b
f1 (ppm)

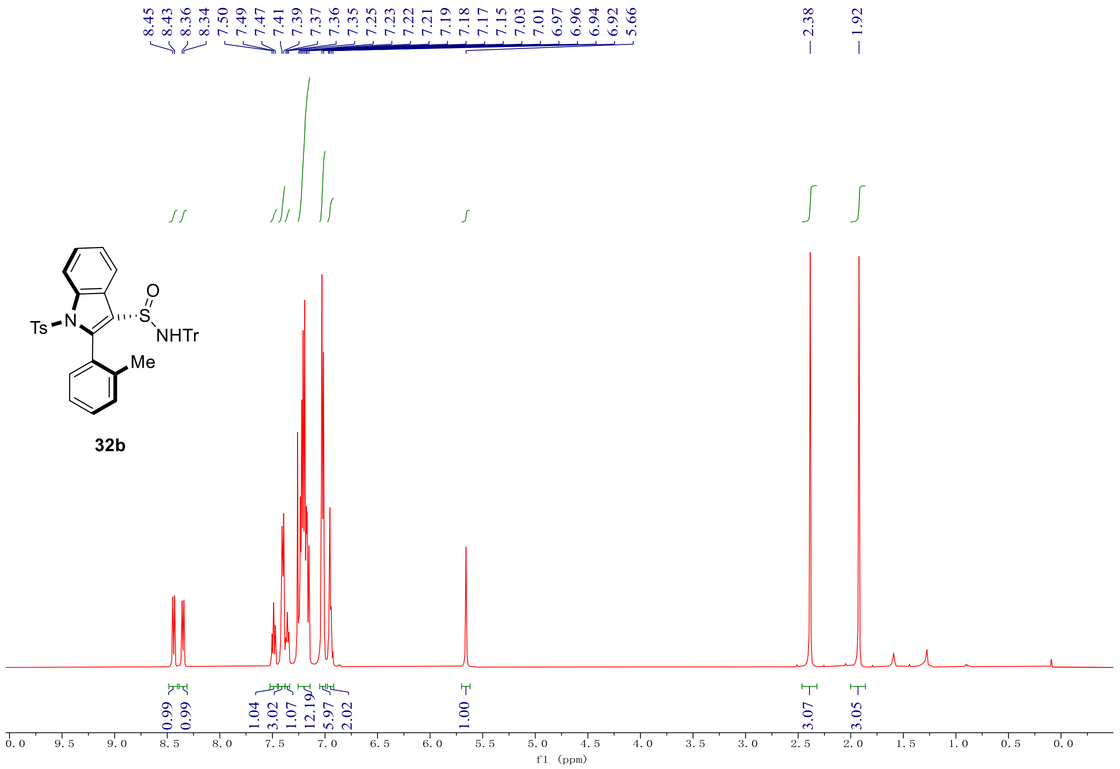
8.45
8.43
8.36
8.34
7.50
7.49
7.47
7.41
7.39
7.37
7.36
7.35
7.25
7.23
7.22
7.21
7.19
7.18
7.17
7.15
7.03
7.01
6.97
6.96
6.94
6.92
5.66
2.38
1.92
Ts
N
S
O
NHTr
Me
32b
0.99
0.99
1.04
3.02
1.07
12.19
5.97
2.02
1.00
3.07
3.05
f1 (ppm)

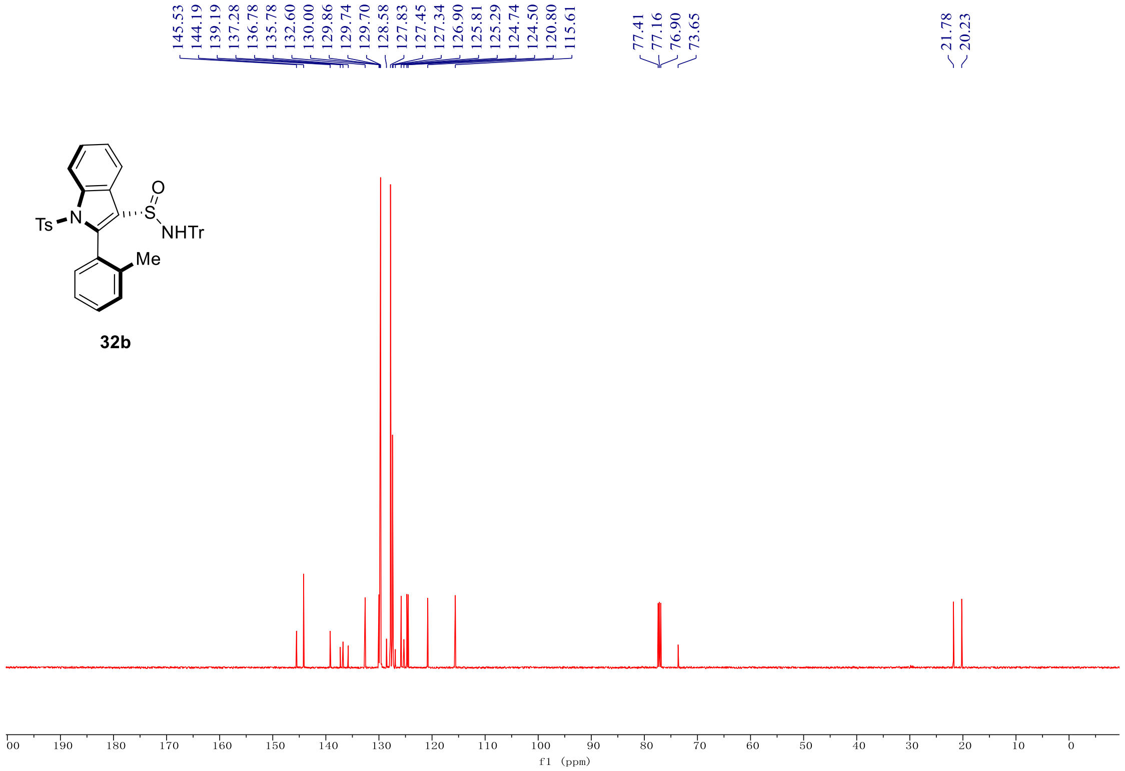
145.53
144.19
139.19
137.28
136.78
135.78
132.60
130.00
129.86
129.74
129.70
128.58
127.83
127.45
127.34
126.90
125.81
125.29
124.74
124.50
120.80
115.61
77.41
77.16
76.90
73.65
21.78
20.23
Ts
N
S
O
NHTr
Me
32b
f1 (ppm)

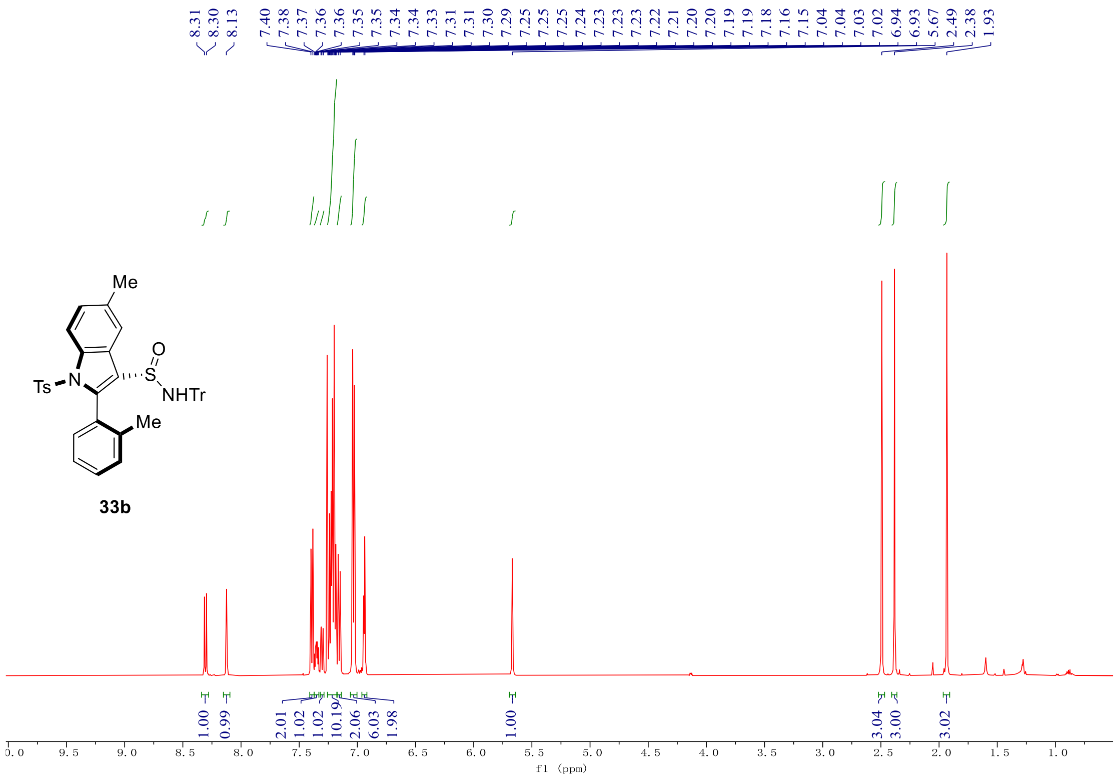
8.31
8.30
8.13
7.40
7.38
7.37
7.36
7.36
7.35
7.35
7.34
7.34
7.33
7.31
7.31
7.30
7.29
7.25
7.25
7.25
7.24
7.23
7.23
7.23
7.22
7.21
7.20
7.20
7.19
7.19
7.18
7.16
7.15
7.04
7.04
7.03
7.02
6.94
6.93
5.67
2.49
2.38
1.93
Me
O
S
Ts
N
NHTr
Me
1.00
0.99
2.01
1.02
1.02
10.19
2.06
6.03
1.98
1.00
3.04
3.00
3.02
9.5
9.0
8.5
8.0
7.5
7.0
6.5
6.0
5.5
5.0
4.5
4.0
3.5
3.0
2.5
2.0
1.5
1.0
f1 (ppm)

33b

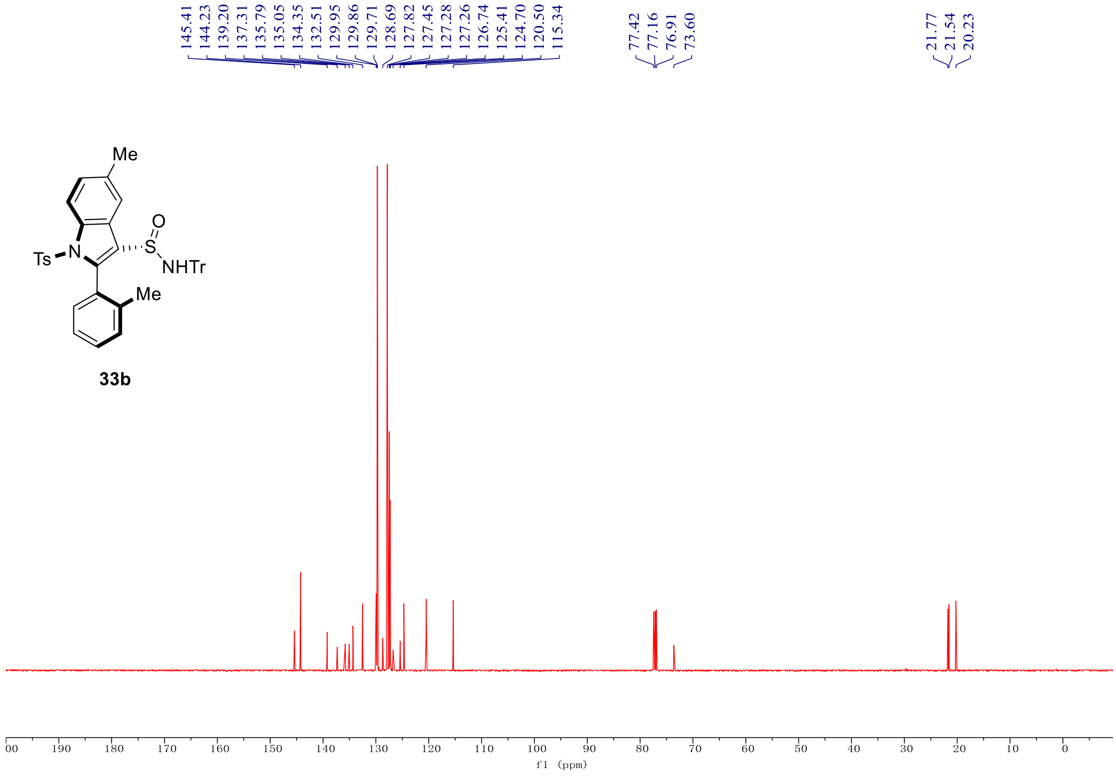
145.41
144.23
139.20
137.31
135.79
135.05
134.35
132.51
129.95
129.86
129.71
128.69
127.82
127.45
127.28
127.26
126.74
125.41
124.70
120.50
115.34
77.42
77.16
76.91
73.60
21.77
21.54
20.23
Me
O
S
Ts
N
NHTr
Me
190
180
170
160
150
140
130
120
110
100
90
80
70
60
50
40
30
20
10
0
f1 (ppm)

33b

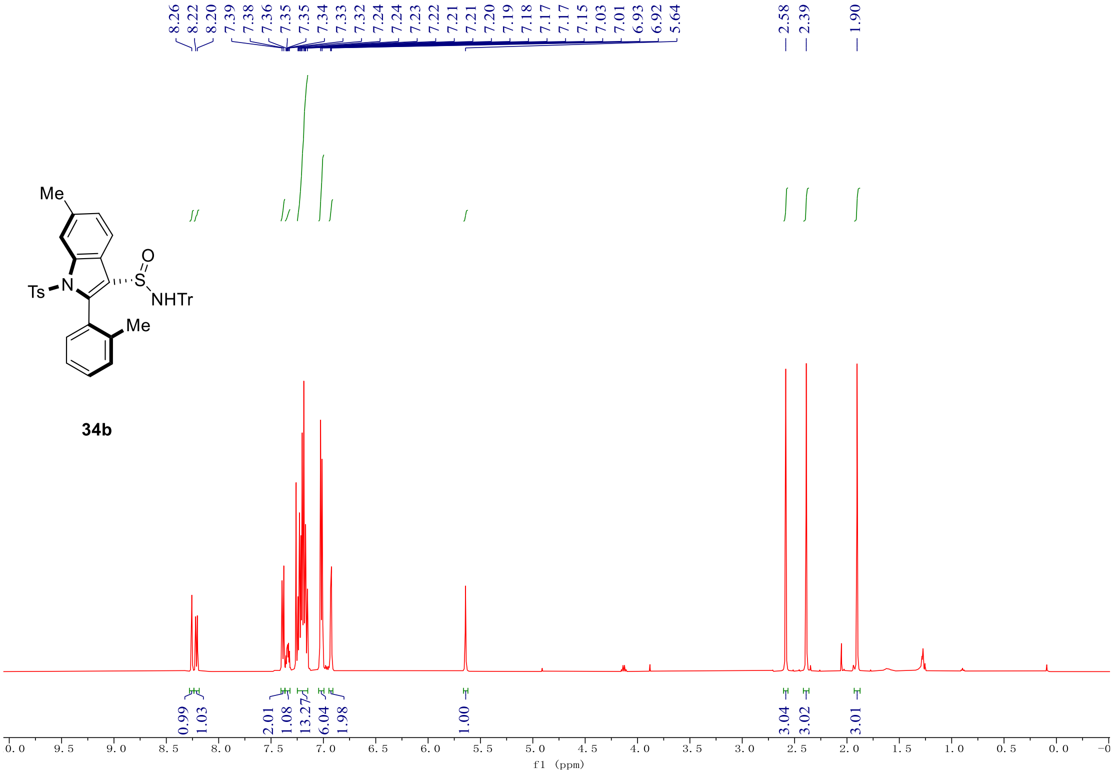
8.26
8.22
8.20
7.39
7.38
7.36
7.35
7.35
7.34
7.33
7.32
7.24
7.24
7.23
7.22
7.21
7.21
7.20
7.19
7.18
7.17
7.17
7.15
7.03
7.01
6.93
6.92
5.64
2.58
2.39
1.90
Me
O
S
N
Ts
NHTr
Me
0.99
1.03
2.01
1.08
13.27
6.04
1.98
1.00
3.04
3.02
3.01
f1 (ppm)

34b

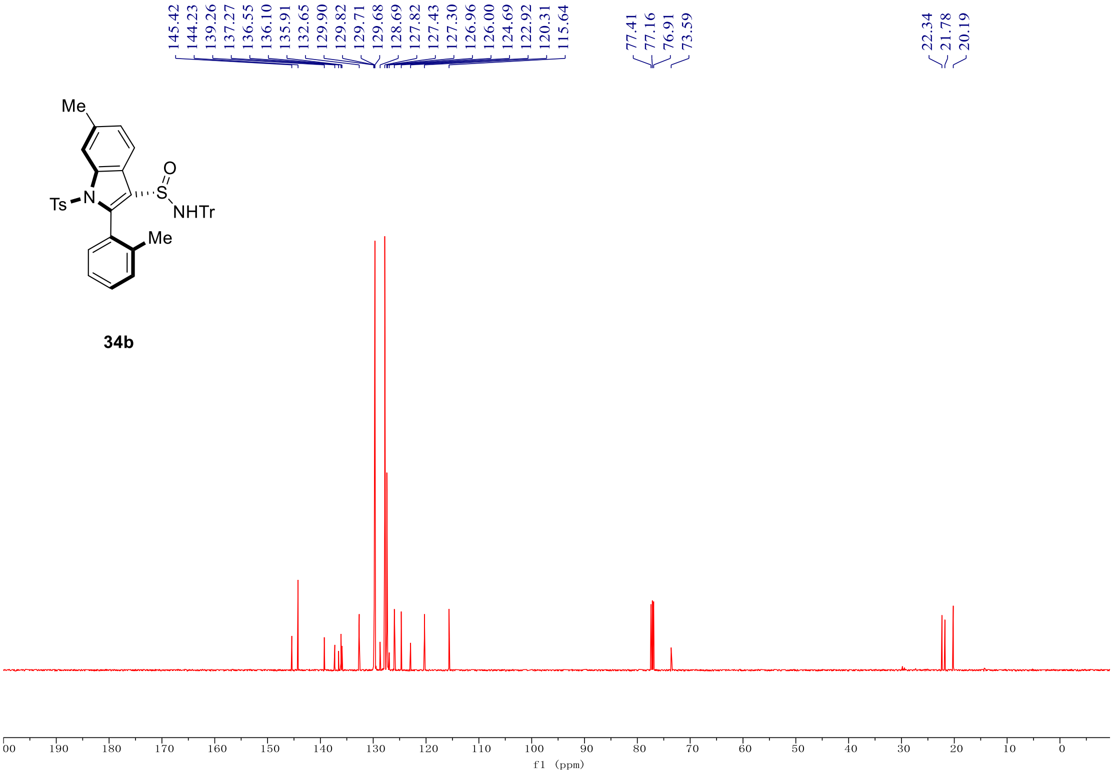
145.42
144.23
139.26
137.27
136.55
136.10
135.91
132.65
129.90
129.82
129.71
129.68
128.69
127.82
127.43
127.30
126.96
126.00
124.69
122.92
120.31
115.64
77.41
77.16
76.91
73.59
22.34
21.78
20.19
Me
O
S
N
Ts
NHTr
Me
f1 (ppm)

34b

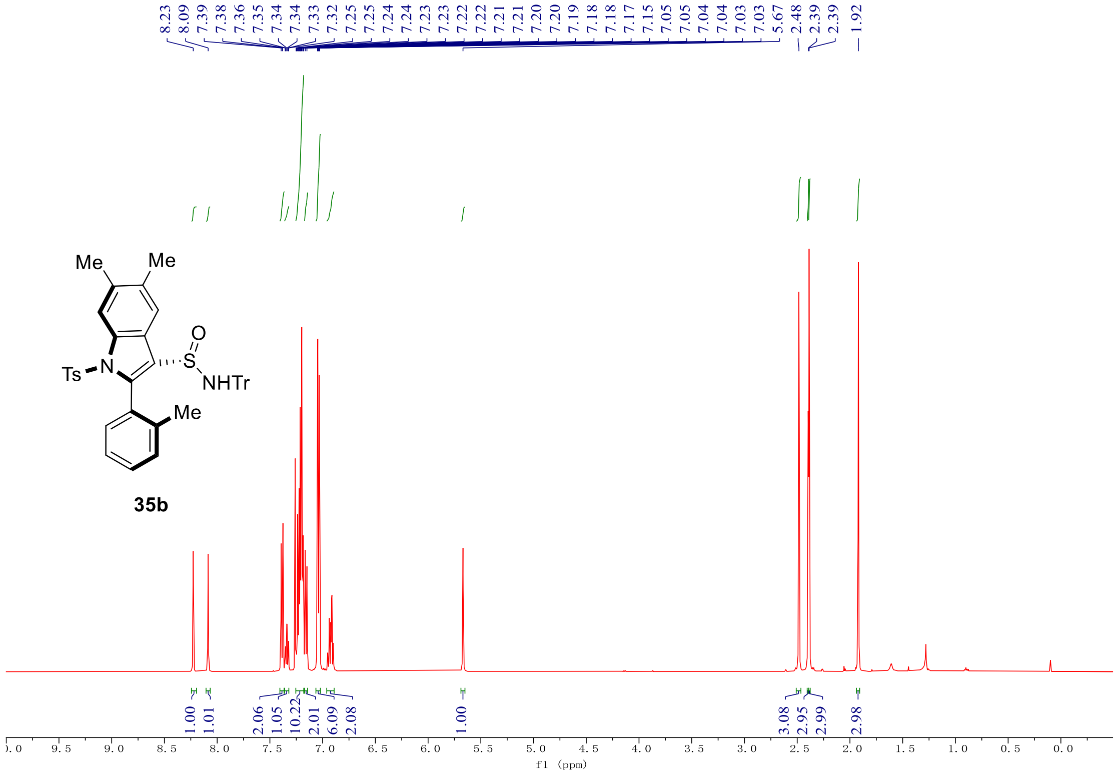

8.23
8.09
7.39
7.38
7.36
7.35
7.34
7.34
7.33
7.32
7.25
7.25
7.24
7.24
7.23
7.23
7.22
7.22
7.21
7.21
7.20
7.20
7.19
7.18
7.18
7.17
7.15
7.05
7.05
7.04
7.04
7.03
7.03
5.67
2.48
2.39
2.39
1.92
Me
Me
O
S
Ts
N
NHTr
Me
35b
1.00
1.01
2.06
1.05
10.22
2.01
6.09
2.08
1.00
3.08
2.95
2.99
2.98
f1 (ppm)

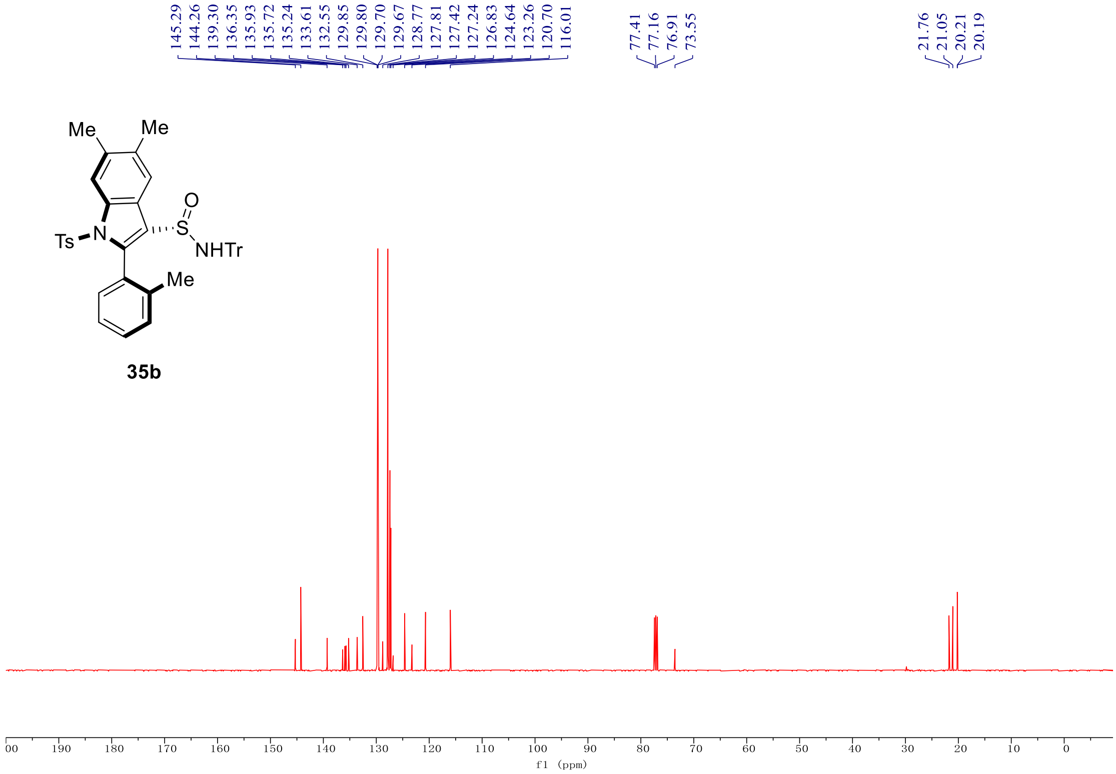

145.29
144.26
139.30
136.35
135.93
135.72
135.24
133.61
132.55
129.85
129.80
129.70
129.67
128.77
127.81
127.42
127.24
126.83
124.64
123.26
120.70
116.01
77.41
77.16
76.91
73.55
21.76
21.05
20.21
20.19
Me
Me
O
S
Ts
N
NHTr
Me
35b
f1 (ppm)

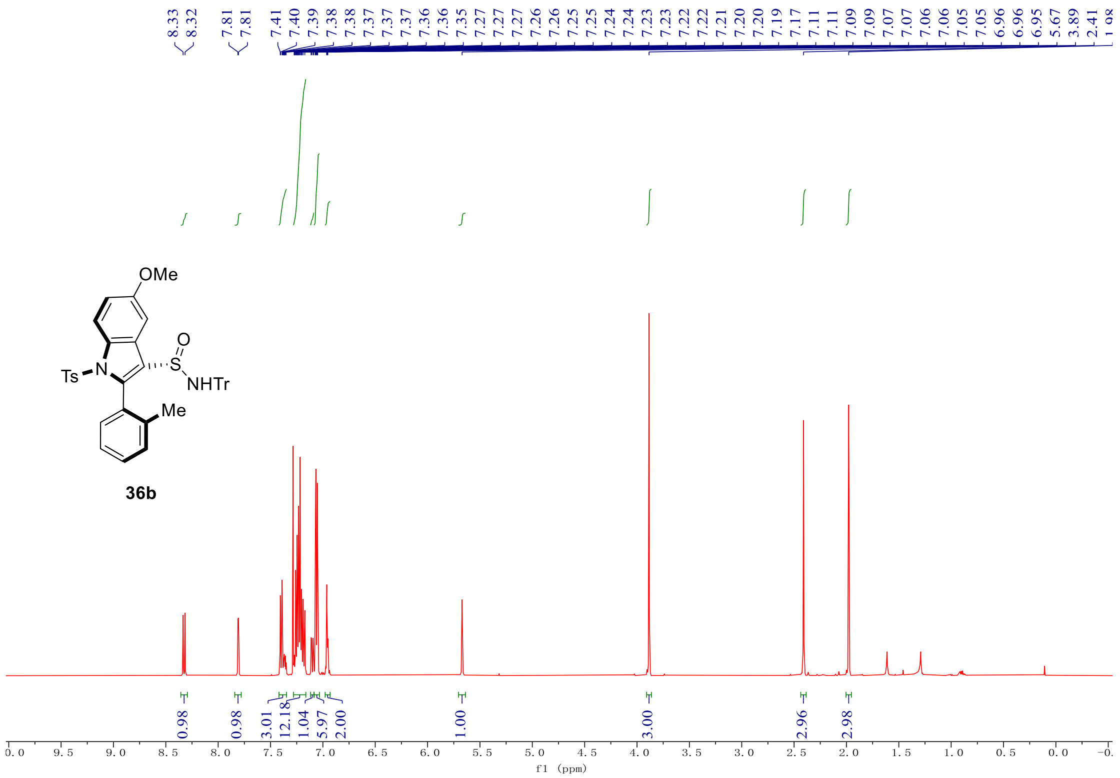

8.33
8.32
7.81
7.81
7.41
7.40
7.39
7.38
7.38
7.37
7.37
7.37
7.36
7.35
7.27
7.27
7.27
7.26
7.26
7.25
7.25
7.24
7.24
7.23
7.23
7.22
7.22
7.21
7.20
7.20
7.19
7.17
7.11
7.11
7.09
7.09
7.07
7.07
7.06
7.06
7.05
7.05
6.96
6.96
6.95
5.67
3.89
2.41
OMe
O
S
N
Ts
NHTr
Me
36b
0.98
0.98
3.01
12.18
1.04
5.97
2.00
1.00
3.00
2.96
2.98
f1 (ppm)

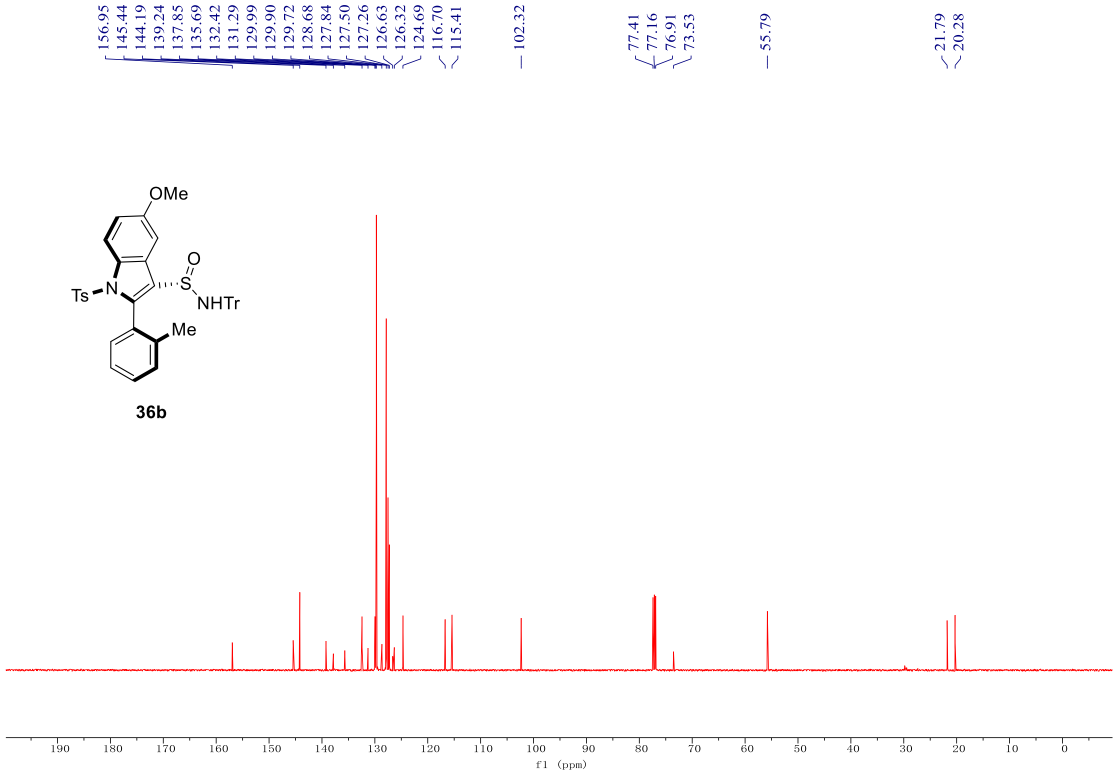

156.95
145.44
144.19
139.24
137.85
135.69
132.42
131.29
129.99
129.90
129.72
128.68
127.84
127.50
127.26
126.63
126.32
124.69
116.70
115.41
102.32
77.41
77.16
76.91
73.53
55.79
21.79
20.28
OMe
O
S
N
Ts
NHTr
Me
36b
f1 (ppm)

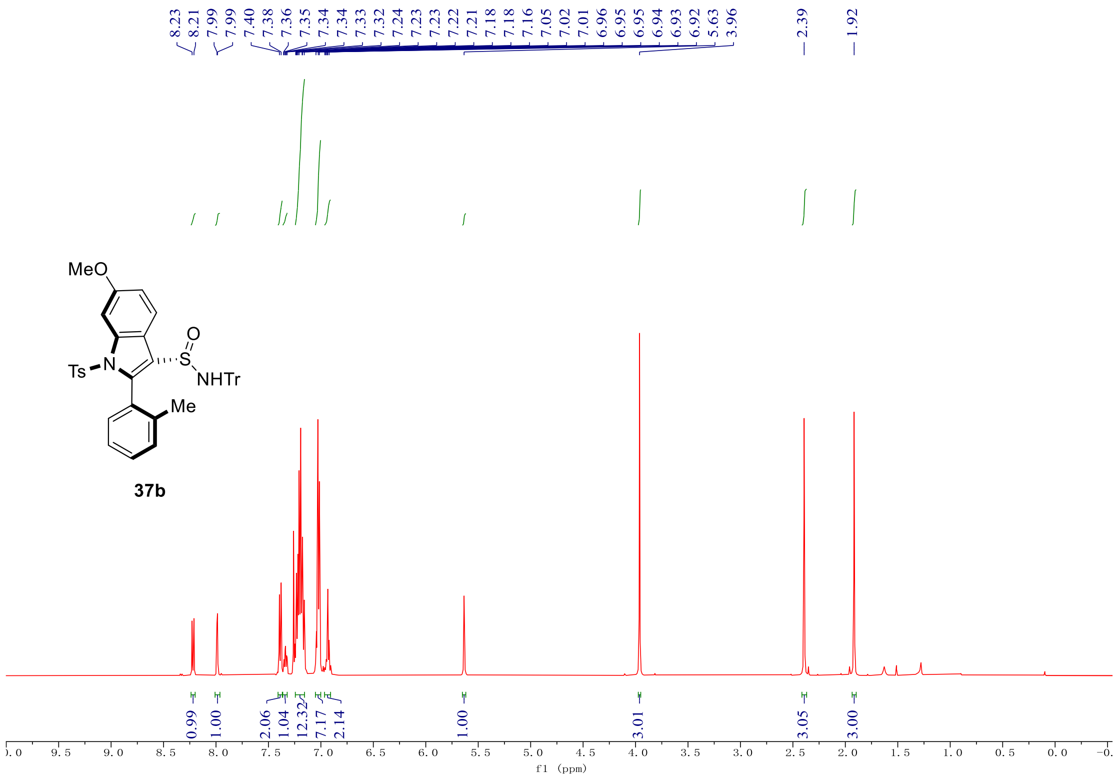
MeO
Ts
N
S
O
NHTr
Me
8.23
8.21
7.99
7.99
7.40
7.38
7.36
7.35
7.34
7.34
7.33
7.32
7.24
7.23
7.23
7.22
7.21
7.18
7.18
7.16
7.05
7.02
7.01
6.96
6.95
6.95
6.94
6.93
6.92
5.63
3.96
2.39
1.92
0.99
1.00
2.06
1.04
12.32
7.17
2.14
1.00
3.01
3.05
3.00
f1 (ppm)

37b

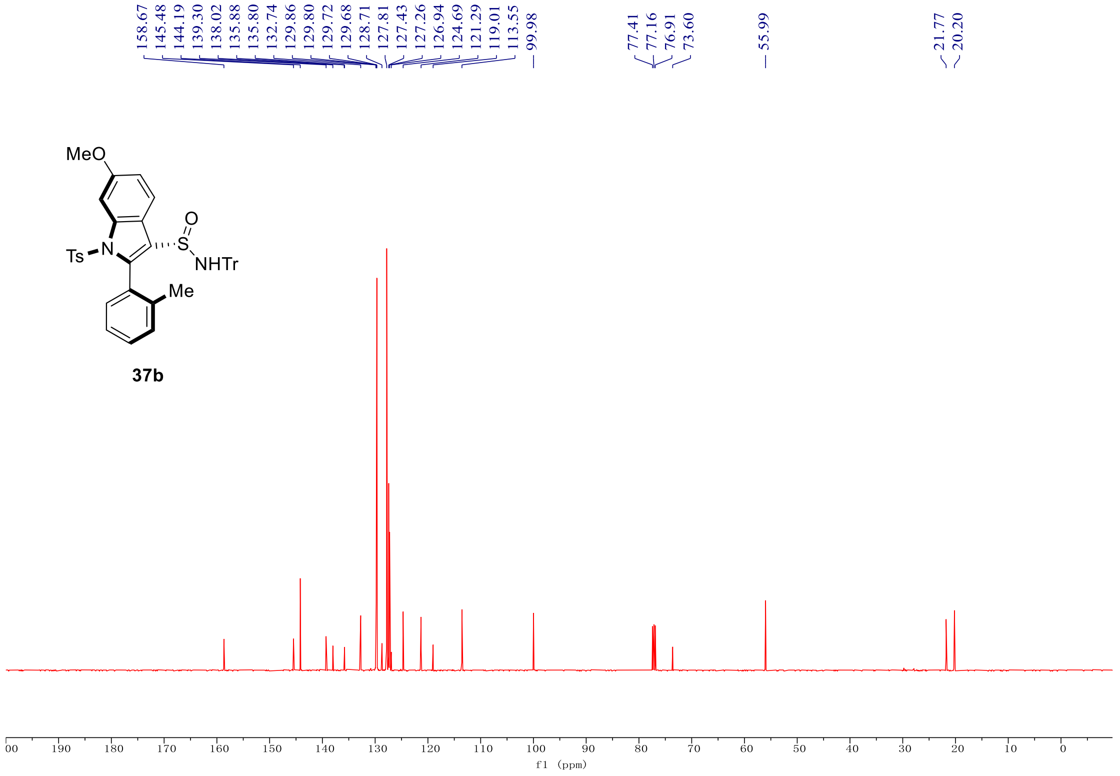
158.67
145.48
144.19
139.30
138.02
135.88
135.80
132.74
129.86
129.80
129.72
129.68
128.71
127.81
127.43
127.26
126.94
124.69
121.29
119.01
113.55
99.98
77.41
77.16
76.91
73.60
55.99
21.77
20.20
MeO
Ts
N
S
O
NHTr
Me
f1 (ppm)

37b

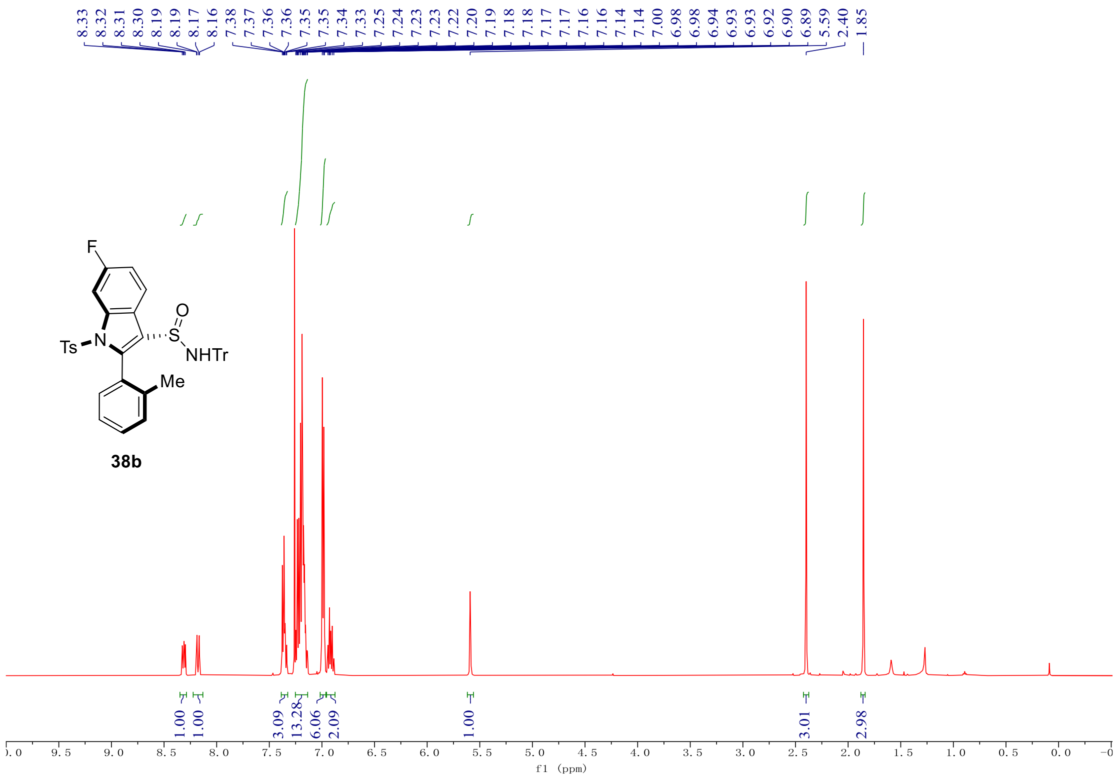

8.33
8.32
8.31
8.30
8.19
8.19
8.17
8.16
7.38
7.37
7.36
7.36
7.35
7.35
7.34
7.33
7.25
7.24
7.23
7.23
7.22
7.20
7.19
7.18
7.18
7.17
7.17
7.16
7.16
7.14
7.00
6.98
6.98
6.94
6.93
6.93
6.92
6.90
6.89
5.59
2.40
1.85
F
O
S
Ts
N
NHTr
Me
38b
1.00
1.00
3.09
13.28
6.06
2.09
1.00
3.01
2.98
f1 (ppm)

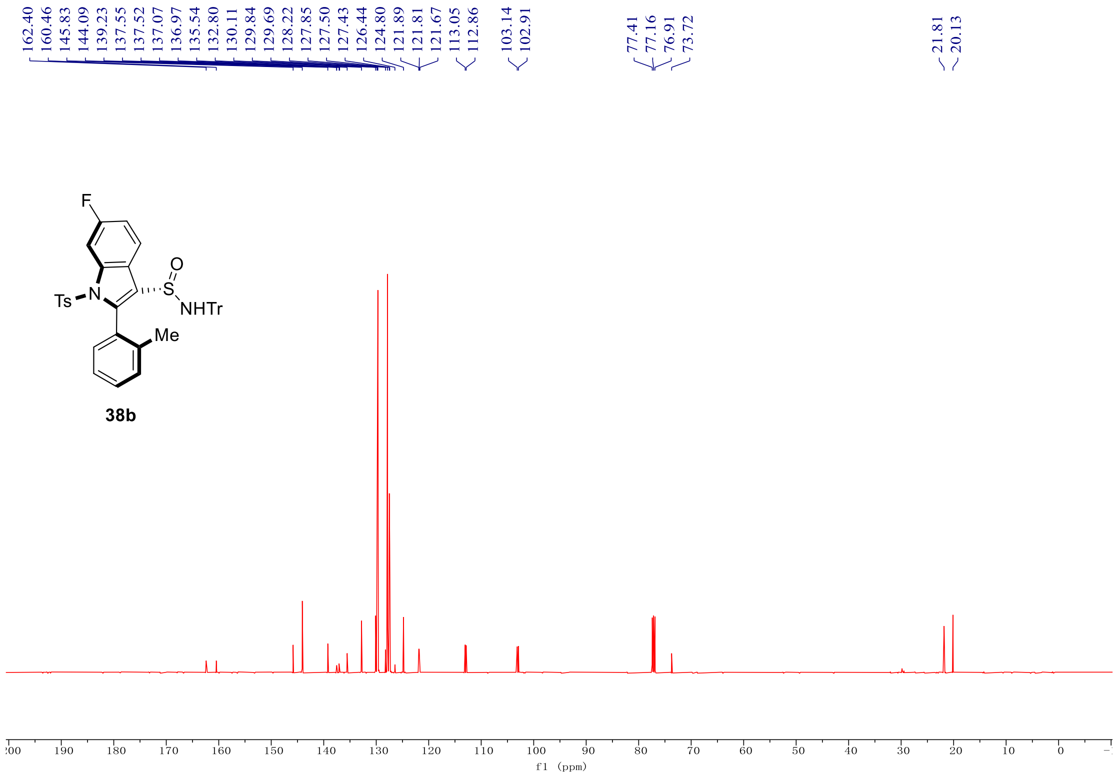

162.40
160.46
145.83
144.09
139.23
137.55
137.52
137.07
136.97
135.54
132.80
130.11
129.84
129.69
128.22
127.85
127.50
127.43
126.44
124.80
121.89
121.81
121.67
113.05
112.86
103.14
102.91
77.41
77.16
76.91
73.72
21.81
20.13
F
O
S
Ts
N
NHTr
Me
38b
f1 (ppm)

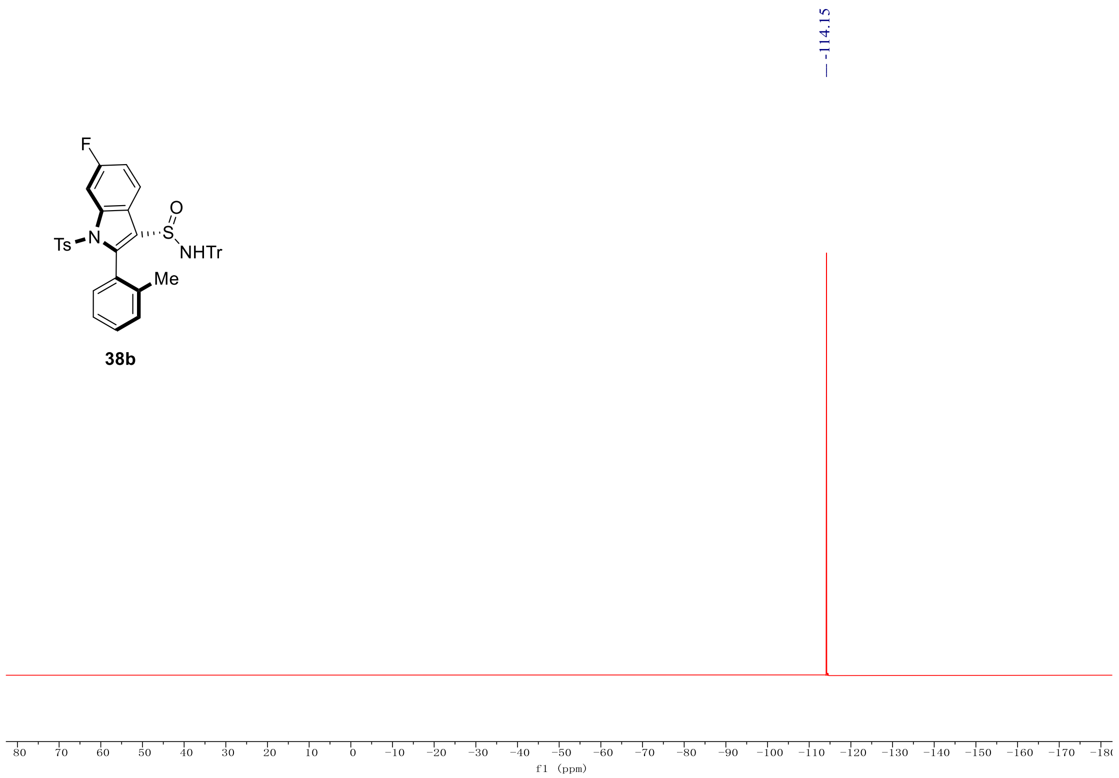

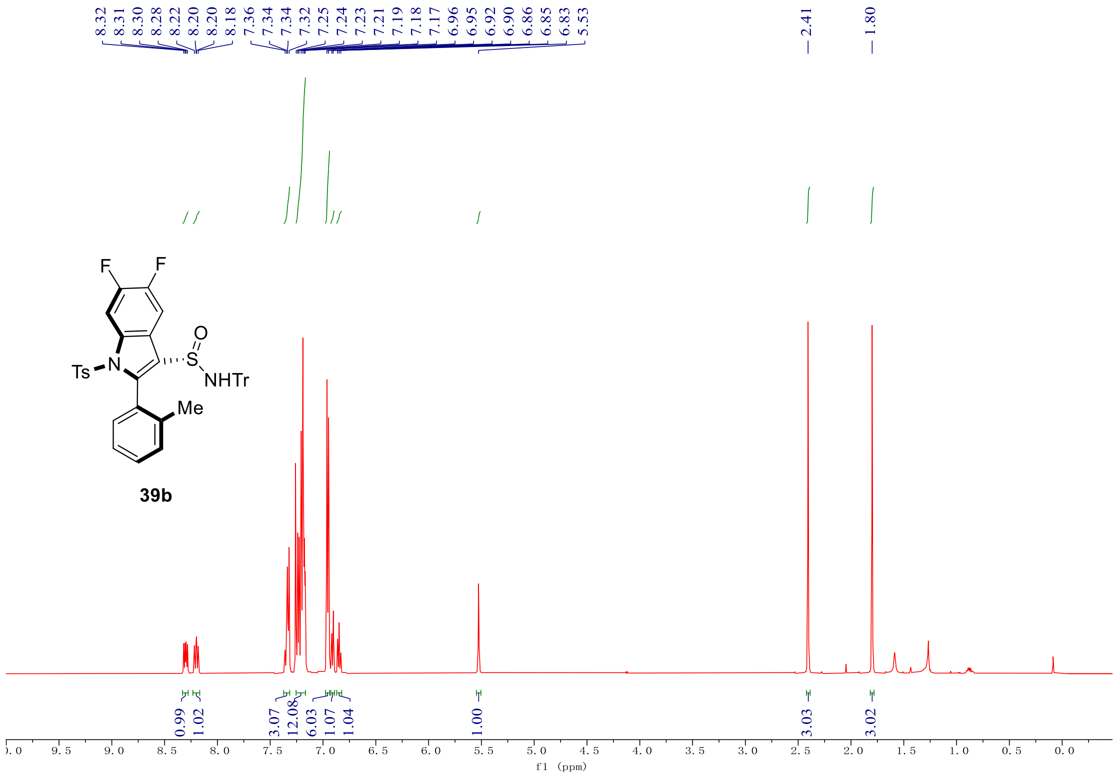

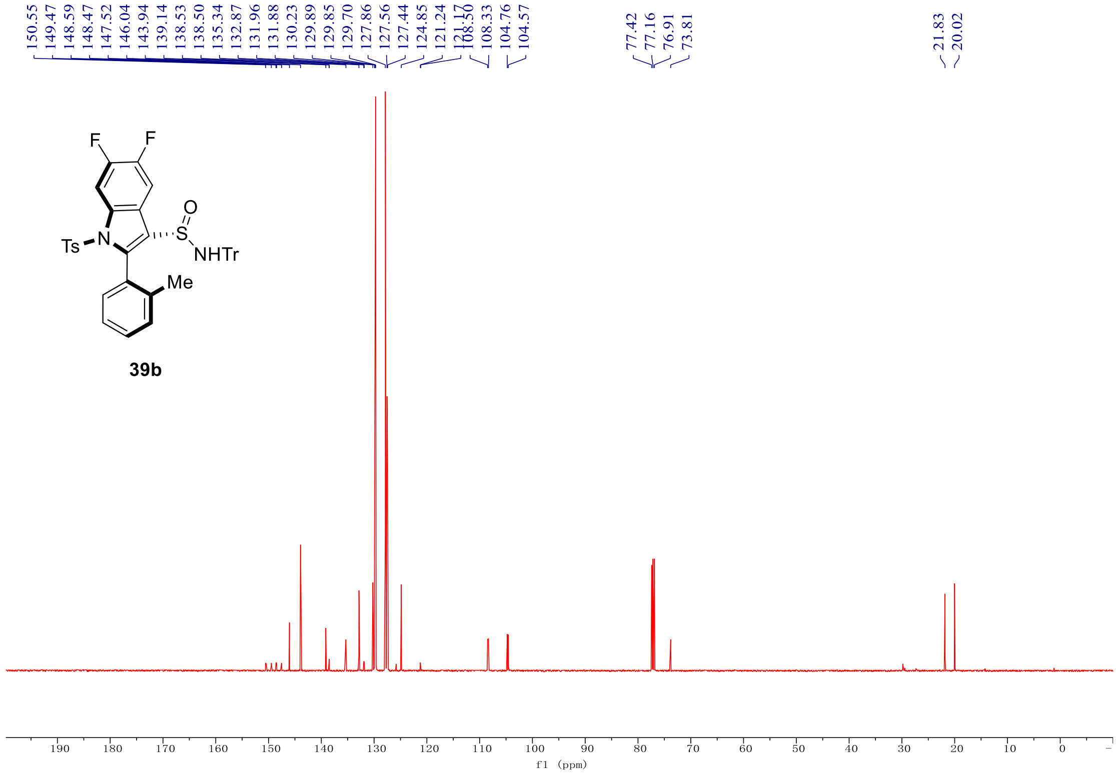
150.55
149.47
148.59
148.47
147.52
146.04
143.94
139.14
138.53
138.50
135.34
132.87
131.96
131.88
130.23
129.89
129.85
129.70
127.86
127.56
127.44
124.85
121.24
121.17
108.50
108.33
104.76
104.57
77.42
77.16
76.91
73.81
21.83
20.02
F
F
O
S
Ts
N
NHTr
Me
190
180
170
160
150
140
130
120
110
100
90
80
70
60
50
40
30
20
10
0
f1 (ppm)

39b

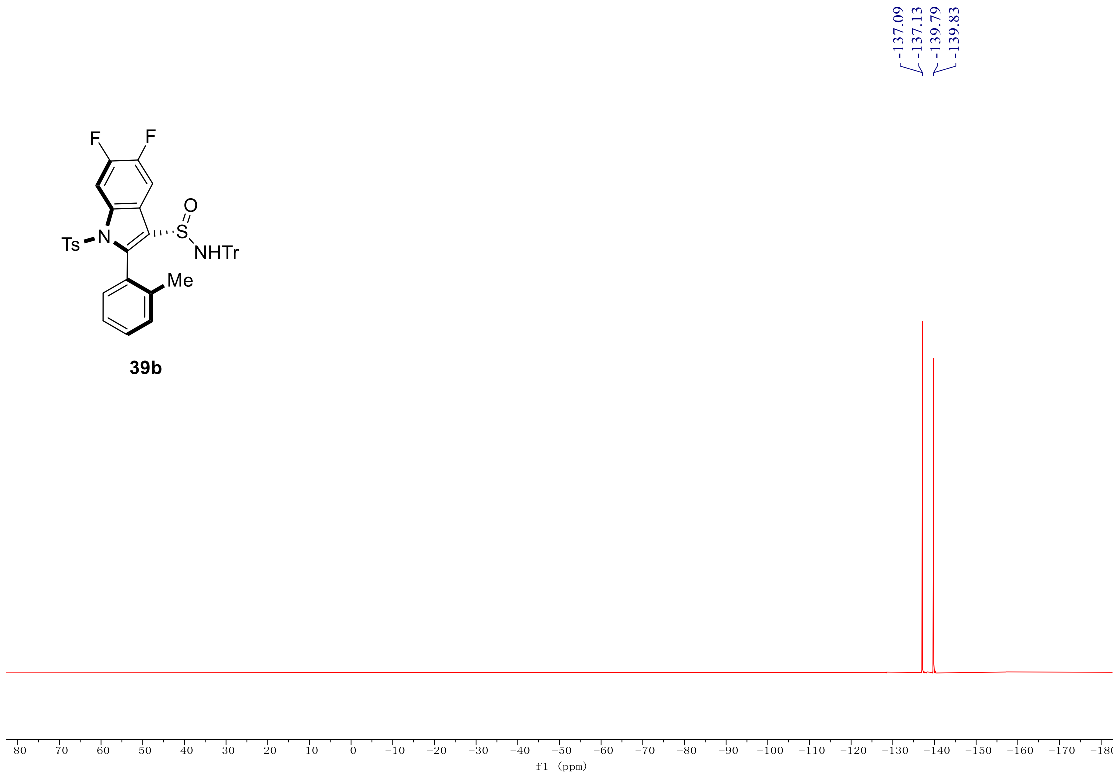
-137.09
-137.13
-139.79
-139.83
F
F
O
S
Ts
N
NHTr
Me
80
70
60
50
40
30
20
10
0
-10
-20
-30
-40
-50
-60
-70
-80
-90
-100
-110
-120
-130
-140
-150
-160
-170
f1 (ppm)

39b

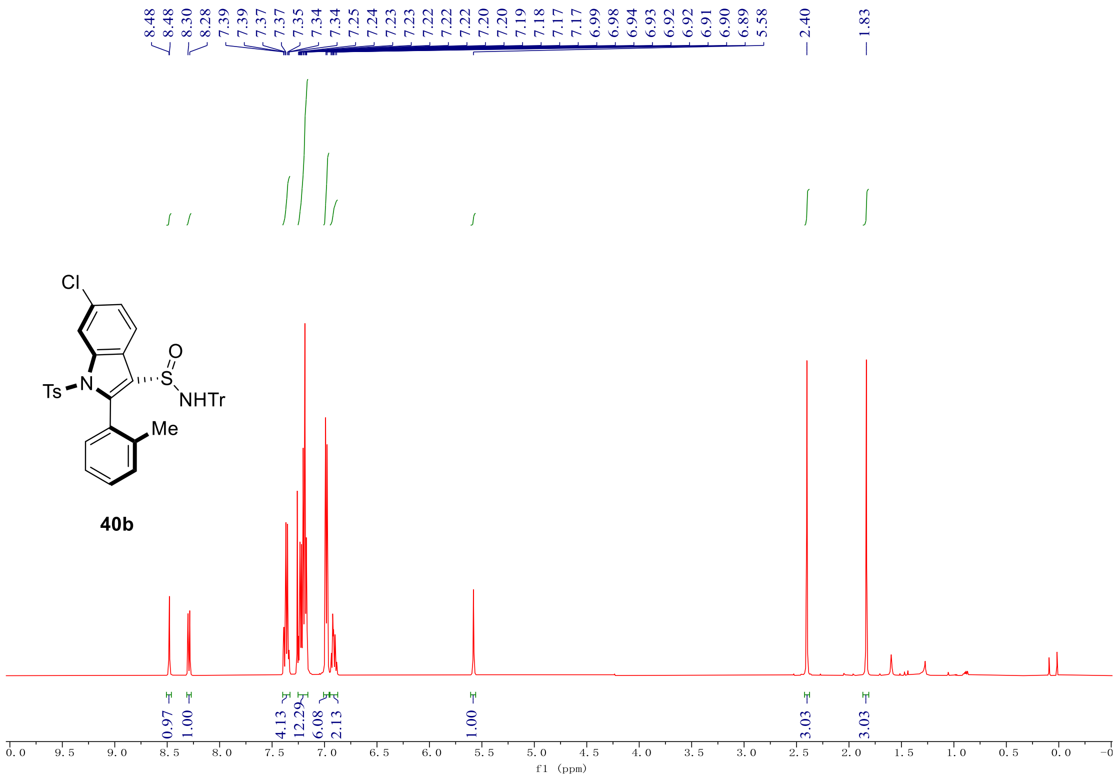

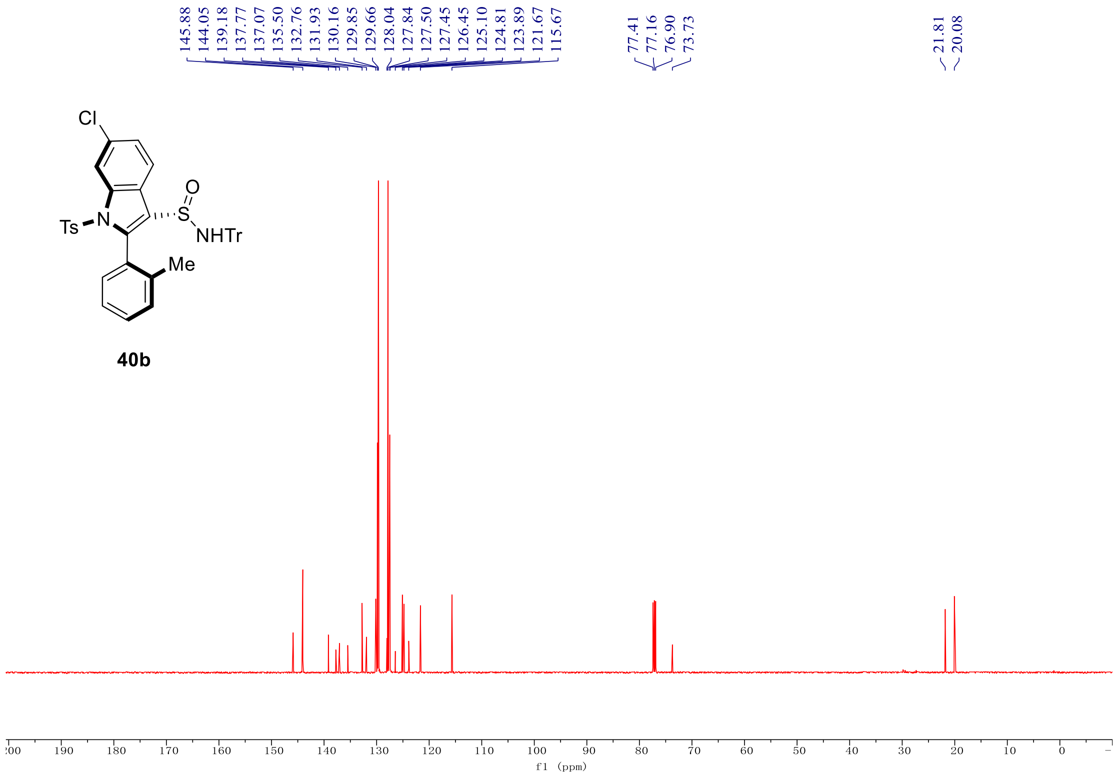

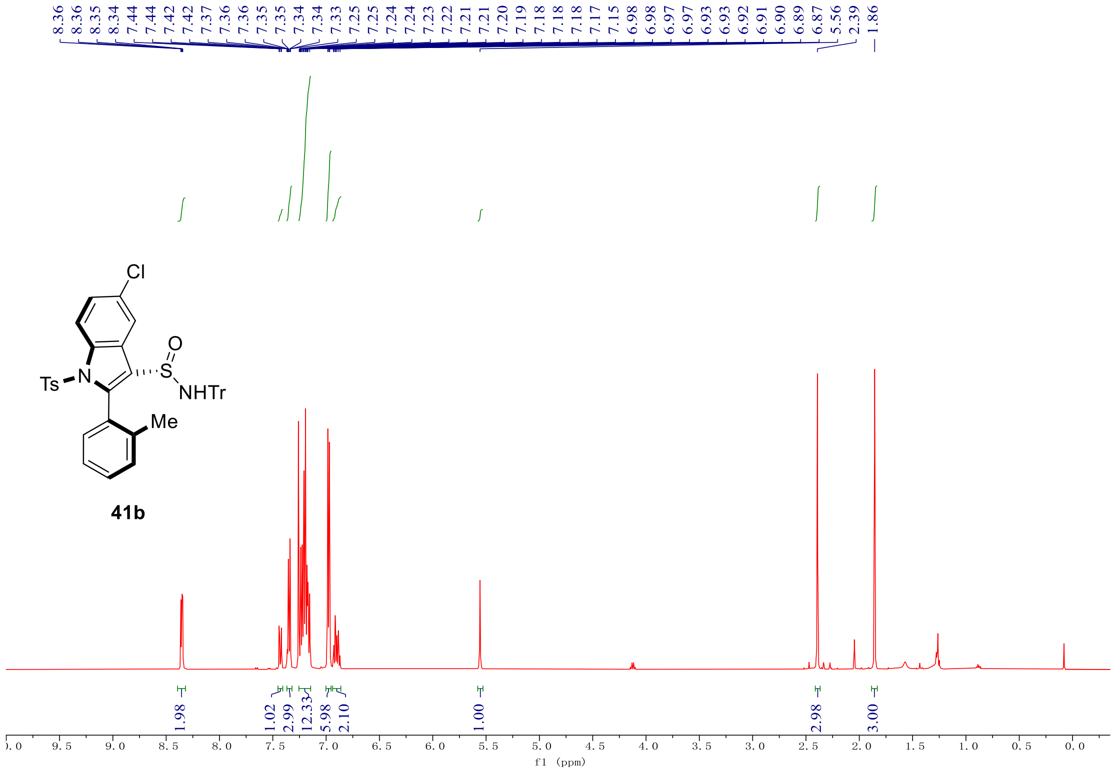
8.36
8.36
8.35
8.34
7.44
7.44
7.42
7.42
7.37
7.36
7.36
7.35
7.35
7.34
7.34
7.33
7.25
7.25
7.24
7.24
7.23
7.22
7.21
7.21
7.20
7.19
7.18
7.18
7.18
7.17
7.15
6.98
6.98
6.97
6.97
6.93
6.93
6.92
6.91
6.90
6.89
6.87
5.56
2.39
1.86
Cl
O
S
Ts
N
NHTr
Me
41b
1.98
1.02
2.99
12.33
5.98
2.10
1.00
2.98
3.00
f1 (ppm)

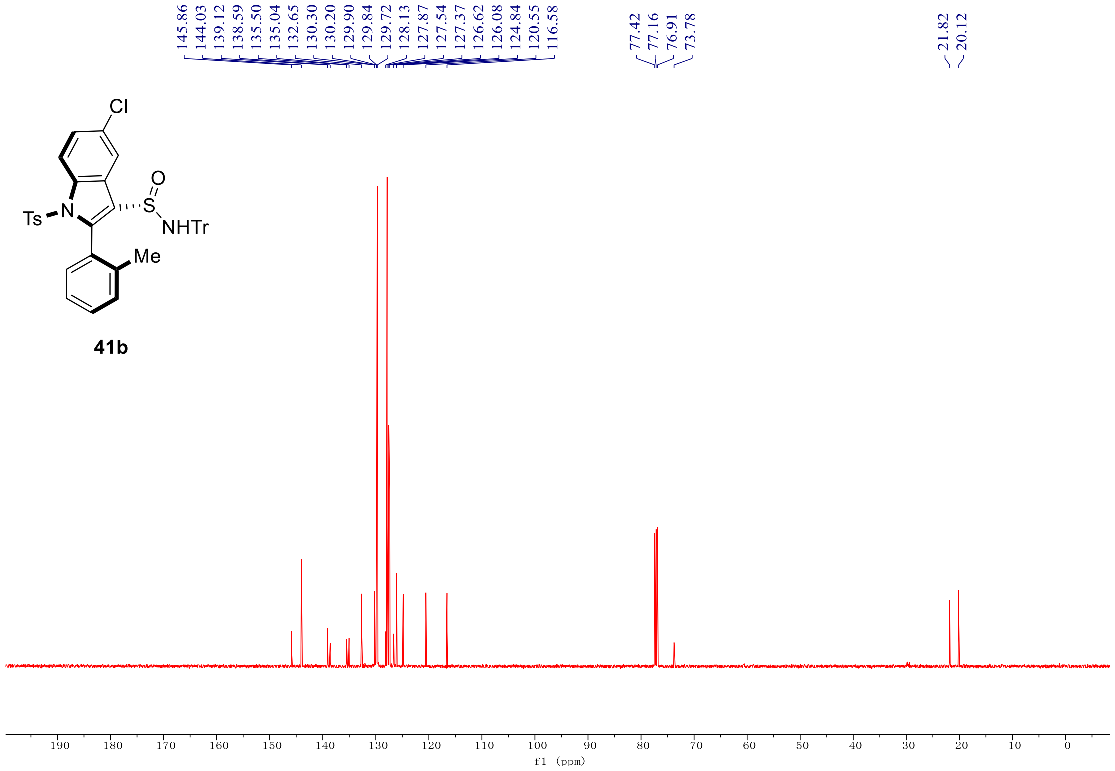
145.86
144.03
139.12
138.59
135.50
135.04
132.65
130.30
130.20
129.90
129.84
129.72
128.13
127.87
127.54
127.37
126.62
126.08
124.84
120.55
116.58
77.42
77.16
76.91
73.78
21.82
20.12
Cl
O
S
Ts
N
NHTr
Me
41b
f1 (ppm)

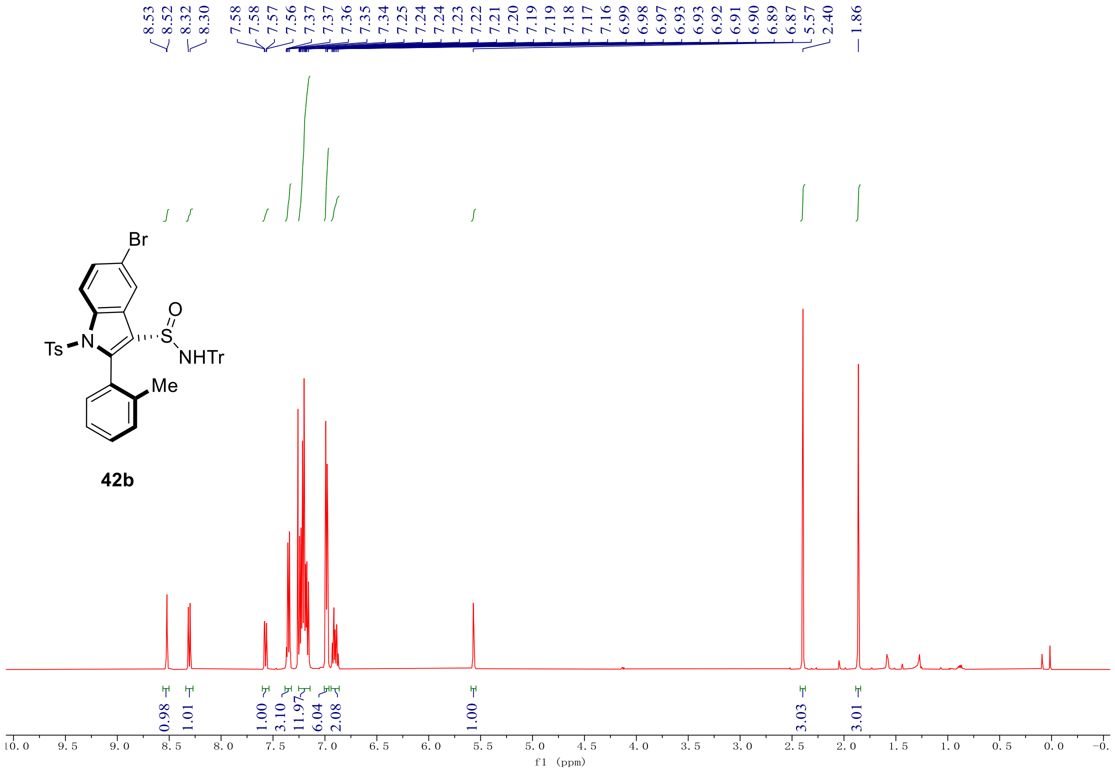
8.53
8.52
8.32
8.30
7.58
7.58
7.57
7.56
7.37
7.37
7.36
7.35
7.34
7.25
7.24
7.24
7.23
7.22
7.21
7.20
7.19
7.19
7.18
7.17
7.16
6.99
6.98
6.97
6.93
6.93
6.92
6.91
6.90
6.89
6.87
5.57
2.40
1.86
Br
O
S
Ts
N
NHTr
Me
0.98
1.01
1.00
3.10
11.97
6.04
2.08
1.00
3.03
3.01
10.0
9.5
9.0
8.5
8.0
7.5
7.0
6.5
6.0
5.5
5.0
4.5
4.0
3.5
3.0
2.5
2.0
1.5
1.0
0.5
0.0
f1 (ppm)

42b

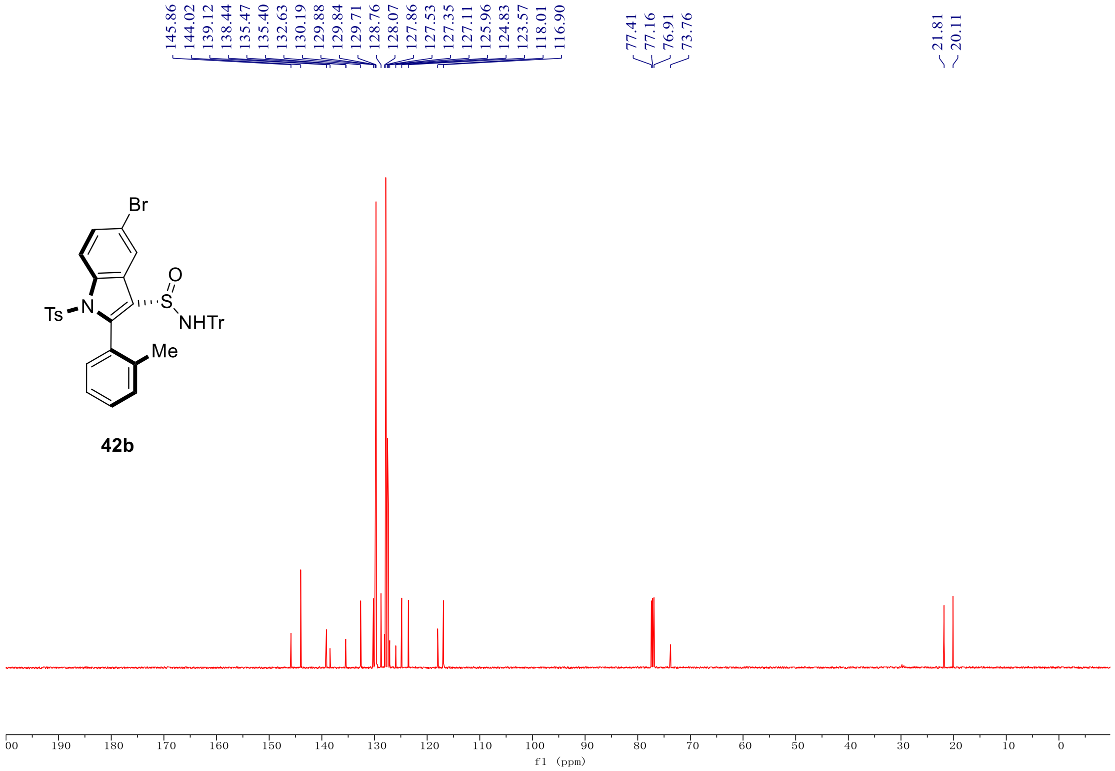
145.86
144.02
139.12
138.44
135.47
135.40
132.63
130.19
129.88
129.84
129.71
128.76
128.07
127.86
127.53
127.35
127.11
125.96
124.83
123.57
118.01
116.90
77.41
77.16
76.91
73.76
21.81
20.11
Br
O
S
Ts
N
NHTr
Me
190
180
170
160
150
140
130
120
110
100
90
80
70
60
50
40
30
20
10
0
f1 (ppm)

42b

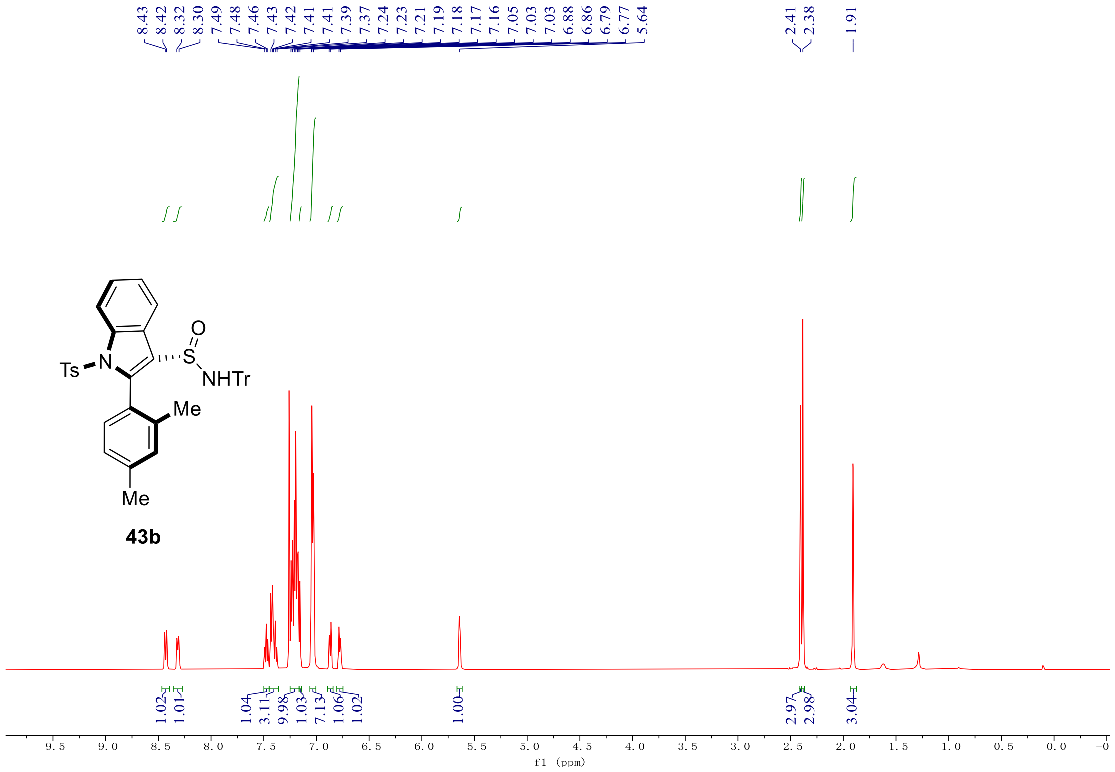
8.43 8.42 8.32 8.30 7.49 7.48 7.46 7.43 7.42 7.41 7.41 7.39 7.37 7.24 7.23 7.21 7.19 7.18 7.17 7.16 7.05 7.03 7.03 6.88 6.86 6.79 6.77 5.64
2.41 2.38 1.91
Ts
N
O
S
NHTr
Me
Me
43b
1.02 1.01 1.04 3.11 9.98 1.03 7.13 1.06 1.02 1.00 2.97 2.98 3.04
9.5 9.0 8.5 8.0 7.5 7.0 6.5 6.0 5.5 5.0 4.5 4.0 3.5 3.0 2.5 2.0 1.5 1.0 0.5 0.0
f1 (ppm)

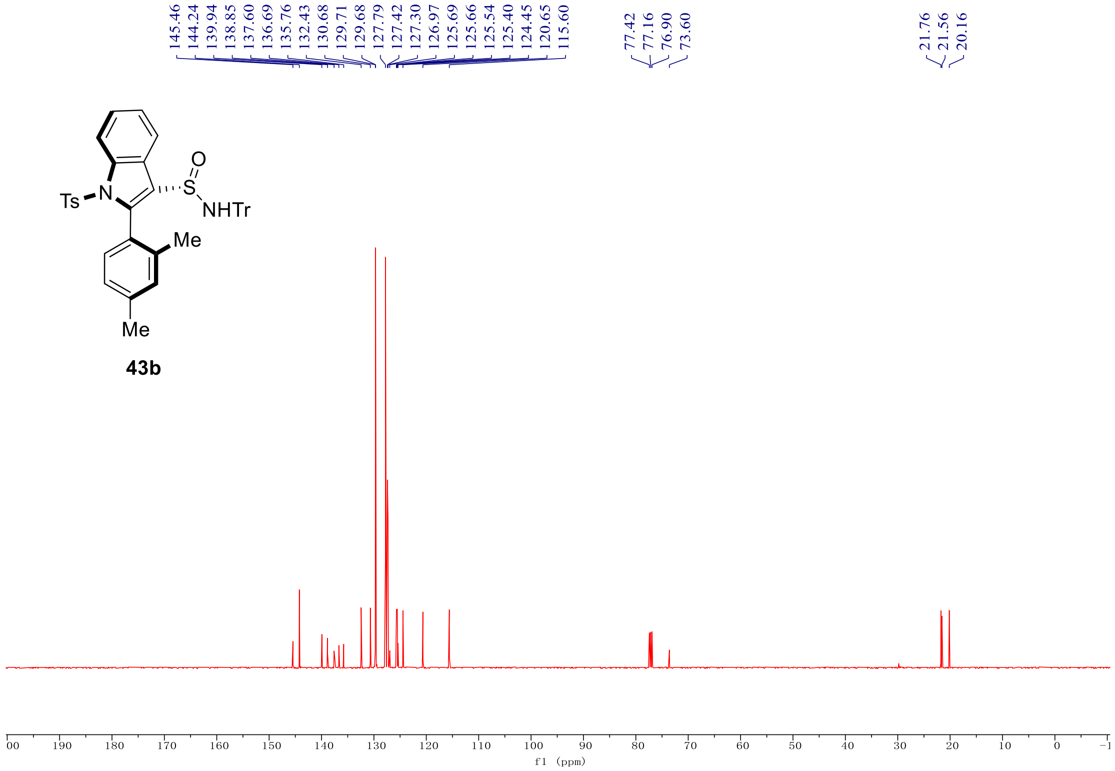
145.46 144.24 139.94 138.85 137.60 136.69 135.76 132.43 130.68 129.71 129.68 127.79 127.42 127.30 126.97 125.69 125.66 125.54 125.40 124.45 120.65 115.60
77.42 77.16 76.90 73.60
21.76 21.56 20.16
Ts
N
O
S
NHTr
Me
Me
43b
190 180 170 160 150 140 130 120 110 100 90 80 70 60 50 40 30 20 10 0
f1 (ppm)

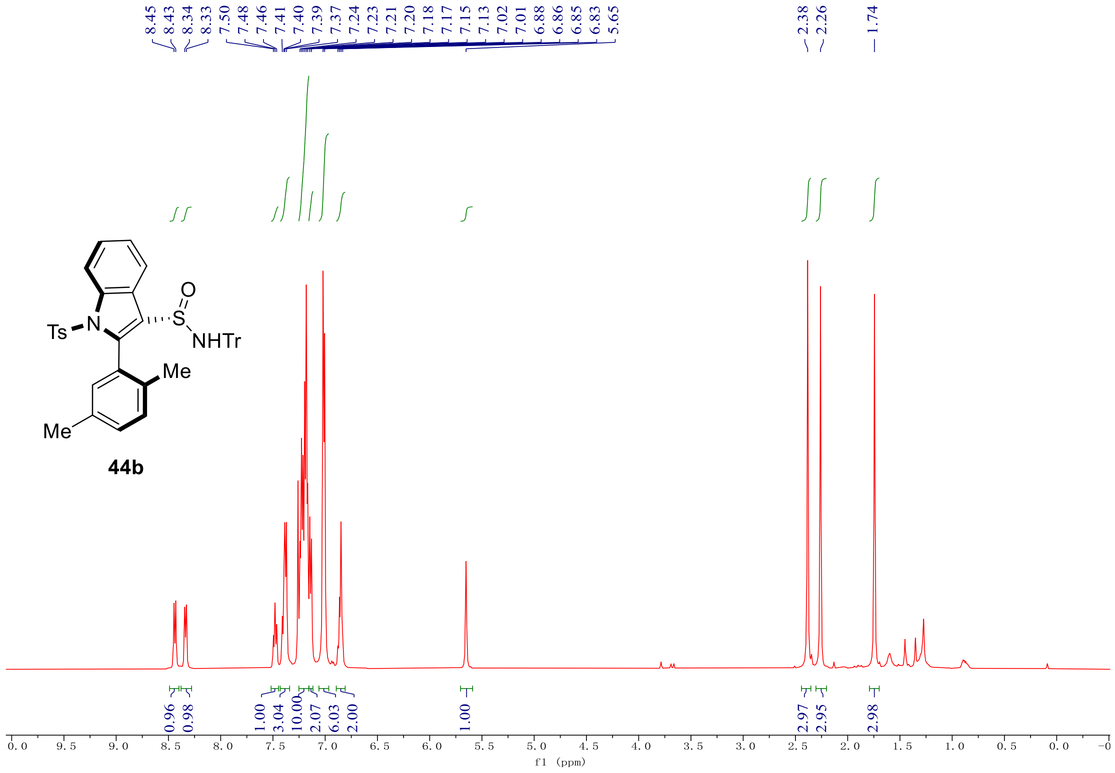
8.45
8.43
8.34
8.33
7.50
7.48
7.46
7.41
7.40
7.39
7.37
7.24
7.23
7.21
7.20
7.18
7.17
7.15
7.13
7.02
7.01
6.88
6.86
6.85
6.83
5.65
2.38
2.26
1.74
Ts
N
S
O
NHTr
Me
Me
44b
0.96
0.98
1.00
3.04
10.00
2.07
6.03
2.00
1.00
2.97
2.95
2.98
f1 (ppm)

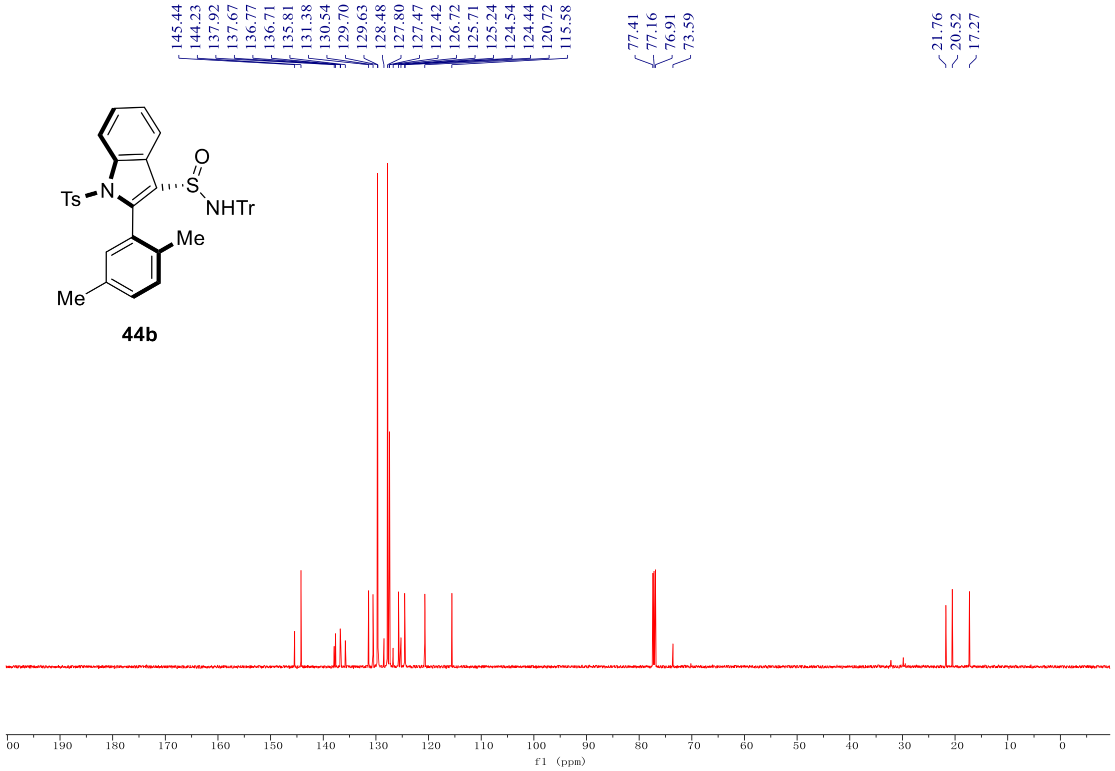
145.44
144.23
137.92
137.67
136.77
136.71
135.81
131.38
130.54
129.70
129.63
128.48
127.80
127.47
127.42
126.72
125.71
125.24
124.54
124.44
120.72
115.58
77.41
77.16
76.91
73.59
21.76
20.52
17.27
Ts
N
S
O
NHTr
Me
Me
44b
f1 (ppm)

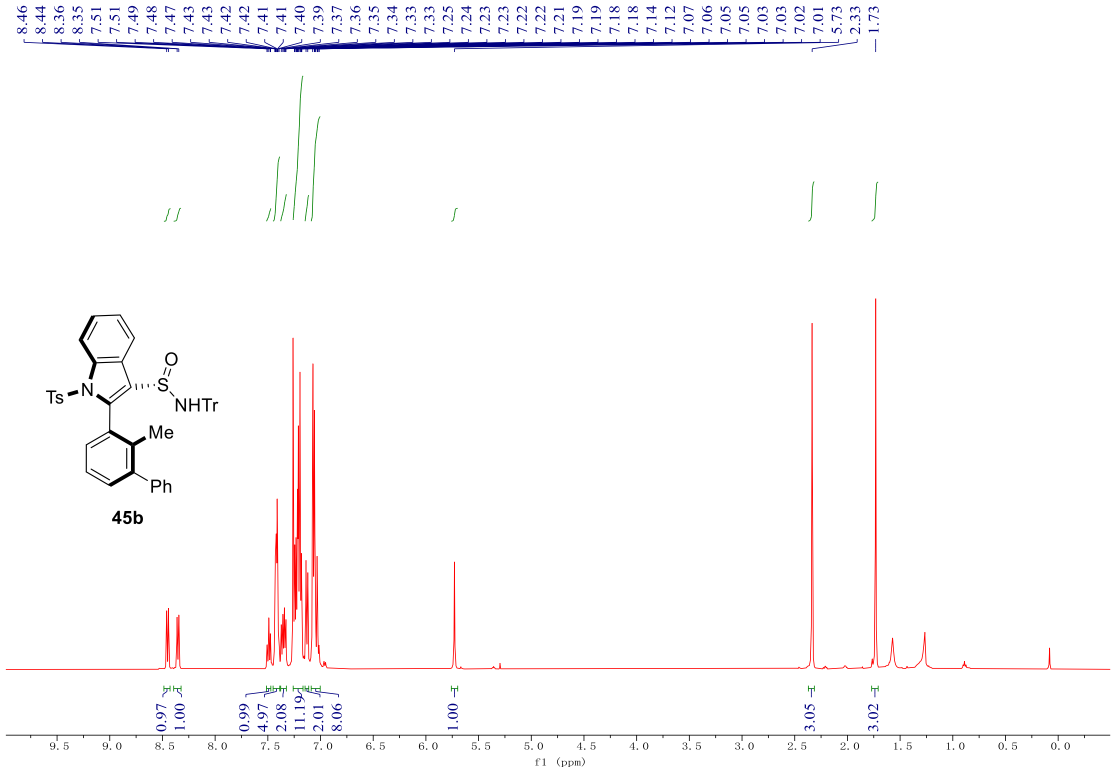

8.46
8.44
8.36
8.35
7.51
7.51
7.49
7.48
7.47
7.43
7.43
7.42
7.42
7.41
7.41
7.40
7.39
7.37
7.36
7.35
7.34
7.33
7.33
7.25
7.24
7.23
7.23
7.22
7.22
7.21
7.19
7.19
7.18
7.18
7.14
7.12
7.07
7.06
7.05
7.05
7.03
7.03
7.02
7.01
5.73
2.33
1.73
O
S
NHTr
Ts
N
Me
Ph
45b
0.97
1.00
0.99
4.97
2.08
11.19
2.01
8.06
1.00
3.05
3.02
f1 (ppm)

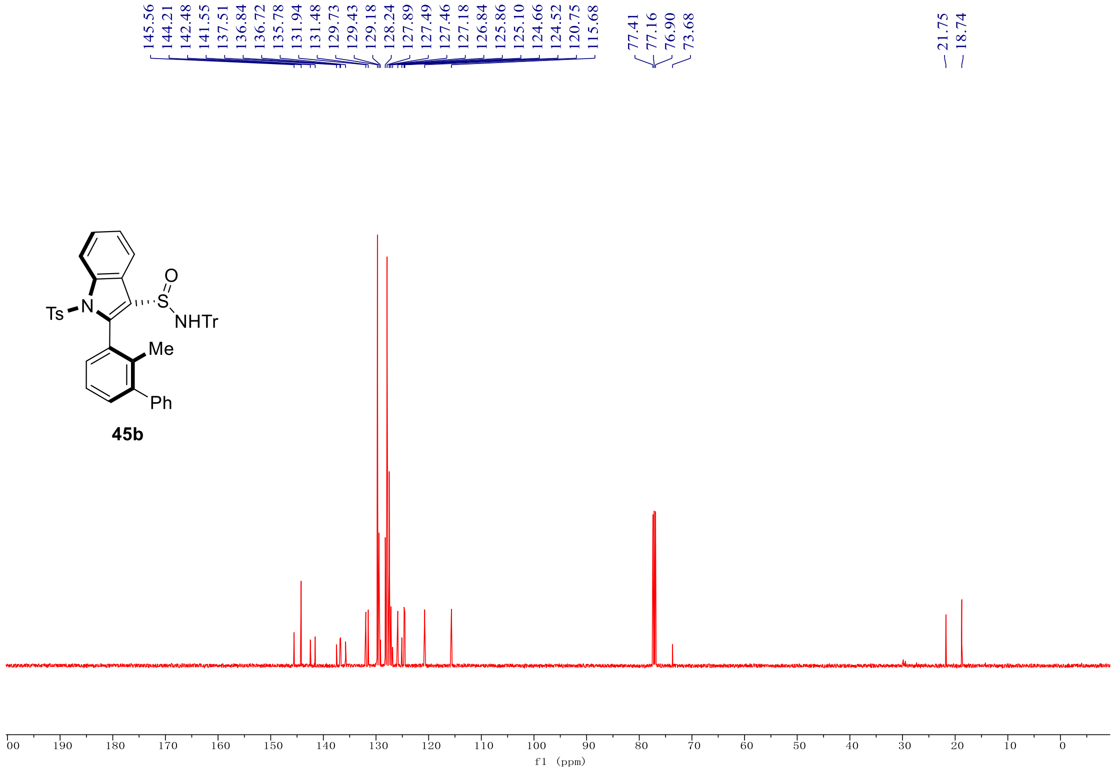

145.56
144.21
142.48
141.55
137.51
136.84
136.72
135.78
131.94
131.48
129.73
129.43
129.18
128.24
127.89
127.49
127.46
127.18
126.84
125.86
125.10
124.66
124.52
120.75
115.68
77.41
77.16
76.90
73.68
21.75
18.74
O
S
NHTr
Ts
N
Me
Ph
45b
f1 (ppm)

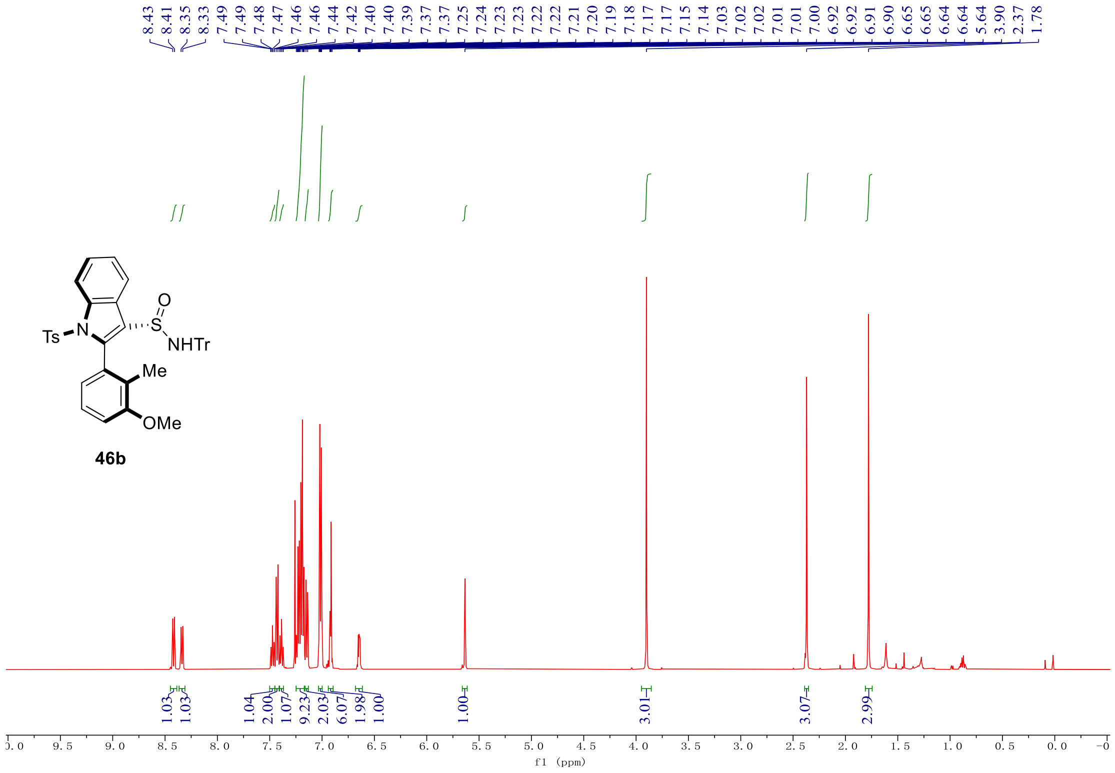

Ts
N
S
O
NHTr
Me
OMe
46b
8.43
8.41
8.35
8.33
7.49
7.48
7.47
7.46
7.46
7.44
7.42
7.40
7.39
7.37
7.37
7.25
7.24
7.23
7.23
7.22
7.22
7.21
7.20
7.19
7.18
7.17
7.17
7.15
7.14
7.03
7.02
7.02
7.01
7.01
7.00
6.92
6.92
6.91
6.90
6.65
6.65
6.64
6.64
5.64
3.90
2.37
1.78
1.03
1.03
1.04
2.00
1.07
9.23
2.03
6.07
1.98
1.00
1.00
3.01
3.07
2.99
f1 (ppm)

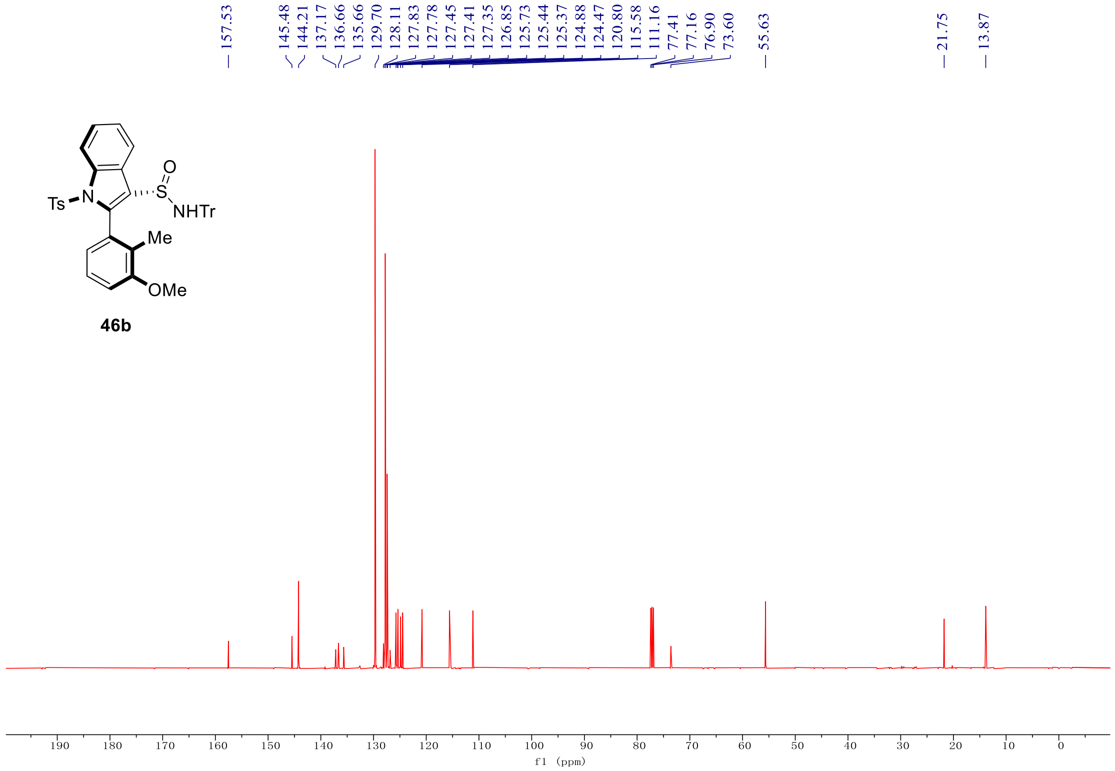

Ts
N
S
O
NHTr
Me
OMe
46b
157.53
145.48
144.21
137.17
136.66
135.66
129.70
128.11
127.83
127.78
127.45
127.41
127.35
126.85
125.73
125.44
125.37
124.88
124.47
120.80
115.58
111.16
77.41
77.16
76.90
73.60
55.63
21.75
13.87
f1 (ppm)

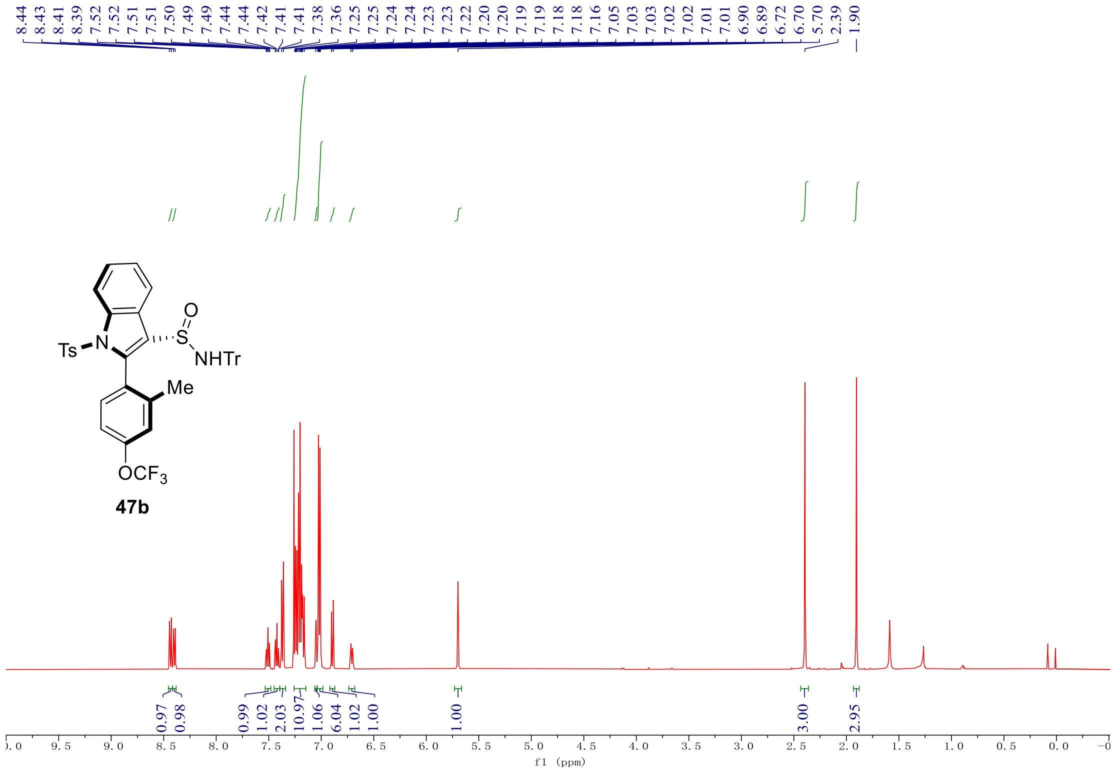
8.44
8.43
8.41
8.39
7.52
7.51
7.51
7.50
7.49
7.49
7.44
7.44
7.42
7.41
7.41
7.38
7.36
7.25
7.25
7.24
7.24
7.23
7.23
7.22
7.20
7.20
7.19
7.19
7.18
7.18
7.16
7.05
7.03
7.03
7.02
7.02
7.01
7.01
6.90
6.89
6.72
6.70
5.70
2.39
1.90
Ts
N
S
O
NHTr
Me
OCF3
0.97
0.98
0.99
1.02
2.03
10.97
1.06
6.04
1.02
1.00
1.00
3.00
2.95
f1 (ppm)

47b

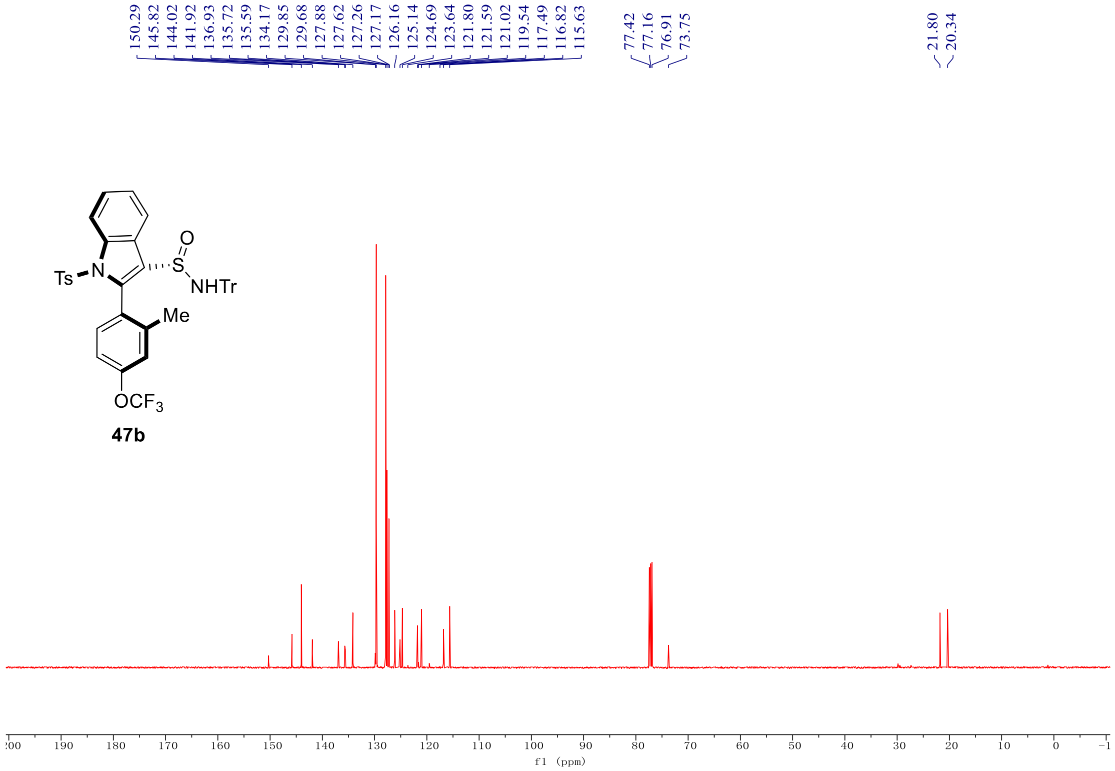
150.29
145.82
144.02
141.92
136.93
135.72
135.59
134.17
129.85
129.68
127.88
127.62
127.26
127.17
126.16
125.14
124.69
123.64
121.80
121.59
121.02
119.54
117.49
116.82
115.63
77.42
77.16
76.91
73.75
21.80
20.34
Ts
N
S
O
NHTr
Me
OCF3
f1 (ppm)

47b

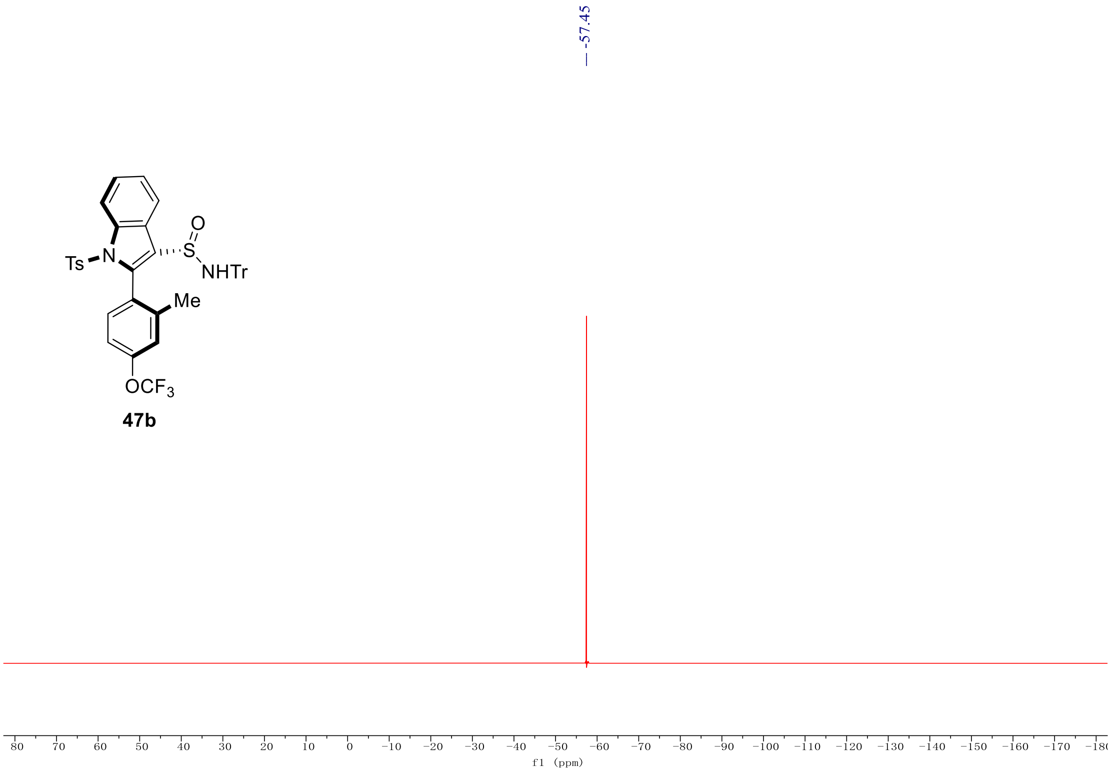
-57.45
Ts
N
S
O
NHTr
Me
OCF3
47b
80 70 60 50 40 30 20 10 0 −10 −20 −30 −40 −50 −60 −70 −80 −90 −100 −110 −120 −130 −140 −150 −160 −170
f1 (ppm)

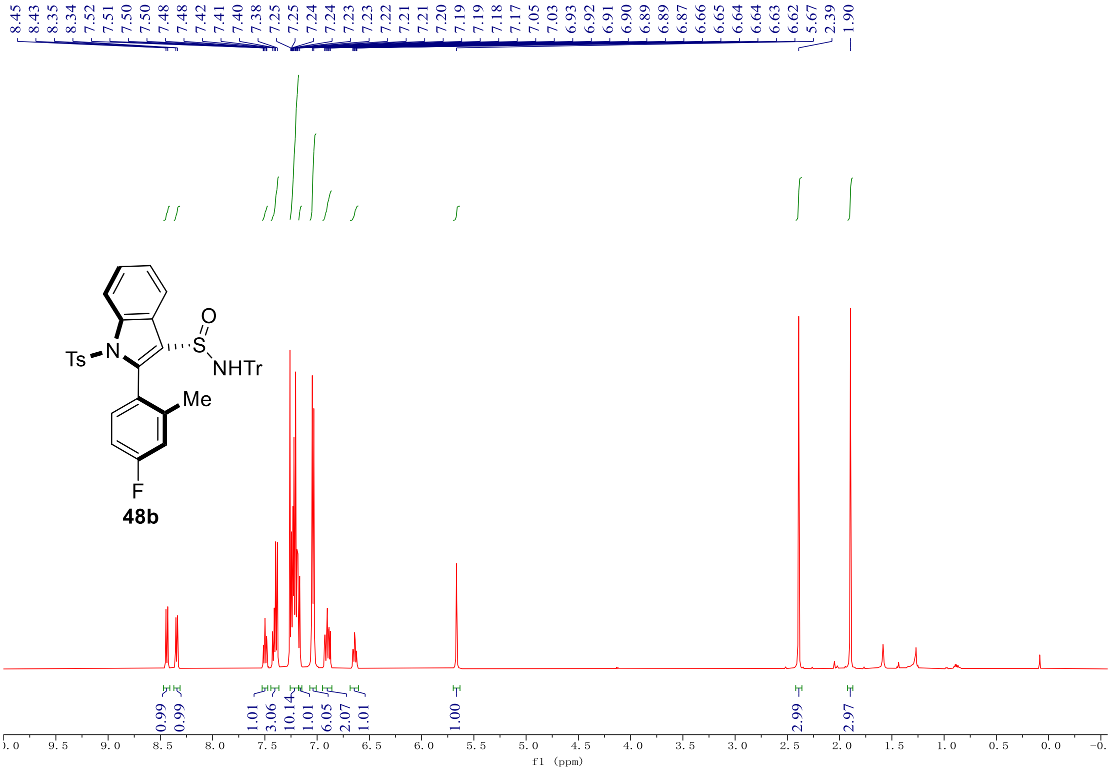
8.45 8.43 8.35 8.34 7.52 7.51 7.50 7.50 7.48 7.48 7.42 7.41 7.40 7.38 7.25 7.25 7.24 7.24 7.23 7.23 7.22 7.21 7.21 7.20 7.19 7.19 7.18 7.17 7.05 7.03 6.93 6.92 6.91 6.90 6.89 6.89 6.87 6.66 6.65 6.64 6.64 6.63 6.62 5.67 2.39 1.90
Ts
N
S
O
NHTr
Me
F
48b
0.99 0.99 1.01 3.06 10.14 1.01 6.05 2.07 1.01 1.00 2.99 2.97
9.5 9.0 8.5 8.0 7.5 7.0 6.5 6.0 5.5 5.0 4.5 4.0 3.5 3.0 2.5 2.0 1.5 1.0 0.5 0.0
f1 (ppm)

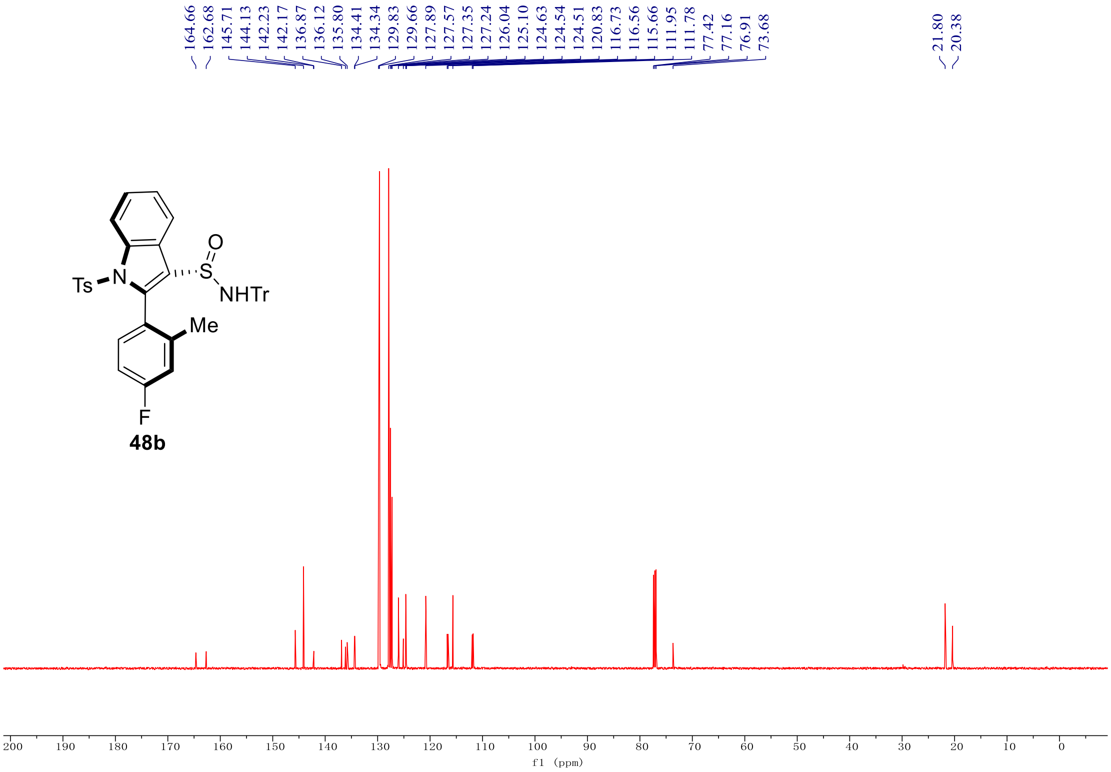
164.66
162.68
145.71
144.13
142.23
142.17
136.87
136.12
135.80
134.41
134.34
129.83
129.66
127.89
127.57
127.35
127.24
126.04
125.10
124.63
124.54
124.51
120.83
116.73
116.56
115.66
111.95
111.78
77.42
77.16
76.91
73.68
21.80
20.38
200 190 180 170 160 150 140 130 120 110 100 90 80 70 60 50 40 30 20 10 0
f1 (ppm)
Ts
N
O
S
NHTr
Me
F
48b

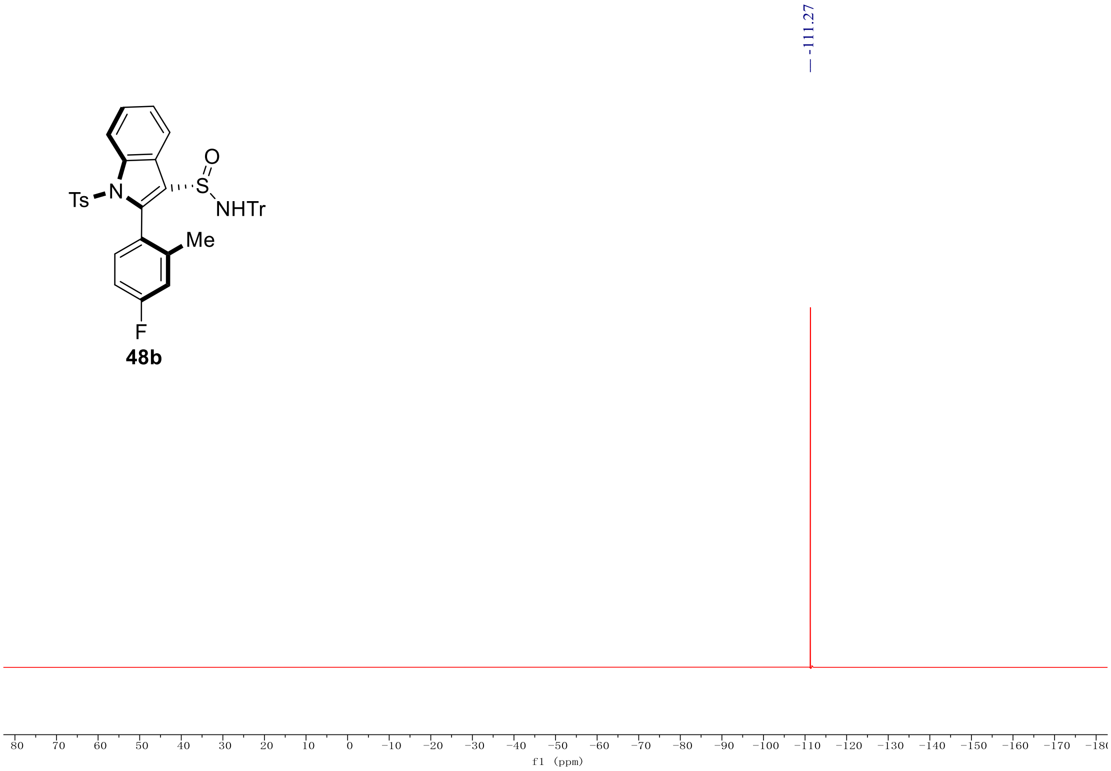
-111.27
80 70 60 50 40 30 20 10 0 -10 -20 -30 -40 -50 -60 -70 -80 -90 -100 -110 -120 -130 -140 -150 -160 -170 -180
f1 (ppm)
Ts
N
O
S
NHTr
Me
F
48b

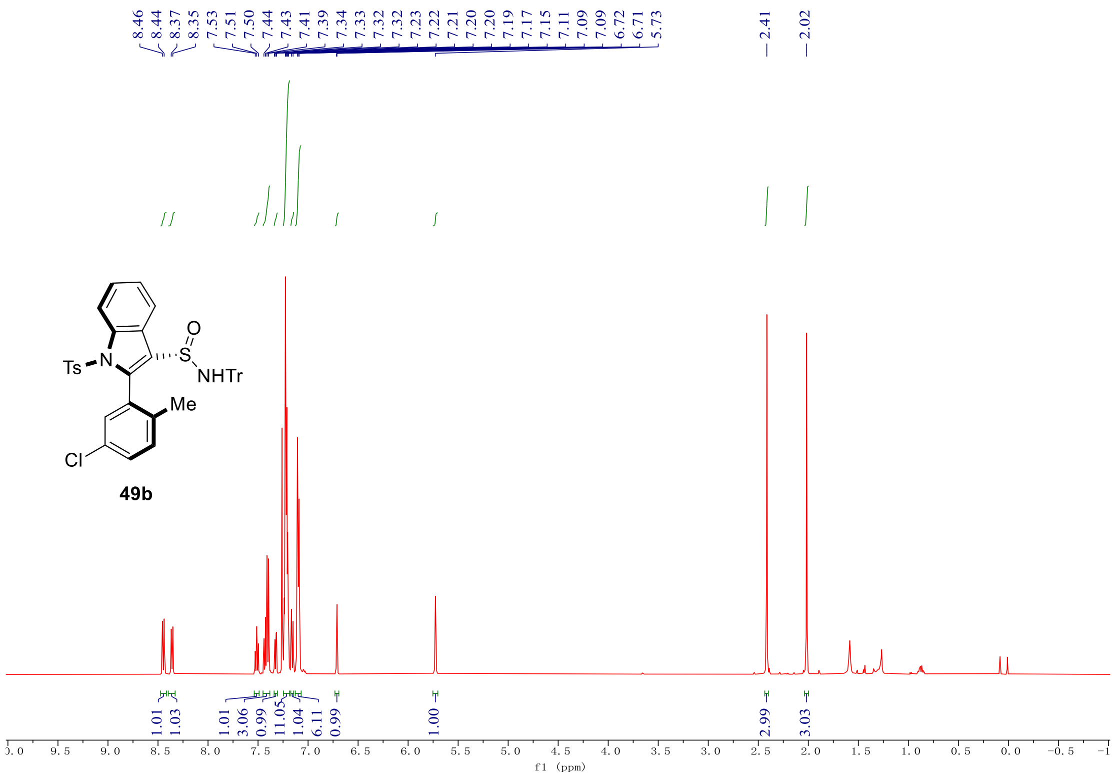
8.46
8.44
8.37
8.35
7.53
7.51
7.50
7.44
7.43
7.41
7.39
7.34
7.33
7.32
7.32
7.23
7.22
7.21
7.20
7.19
7.17
7.15
7.11
7.09
7.09
6.72
6.71
5.73
2.41
2.02
Ts
N
S
O
NHTr
Me
Cl
49b
1.01
1.03
1.01
3.06
0.99
11.05
1.04
6.11
0.99
1.00
2.99
3.03
f1 (ppm)

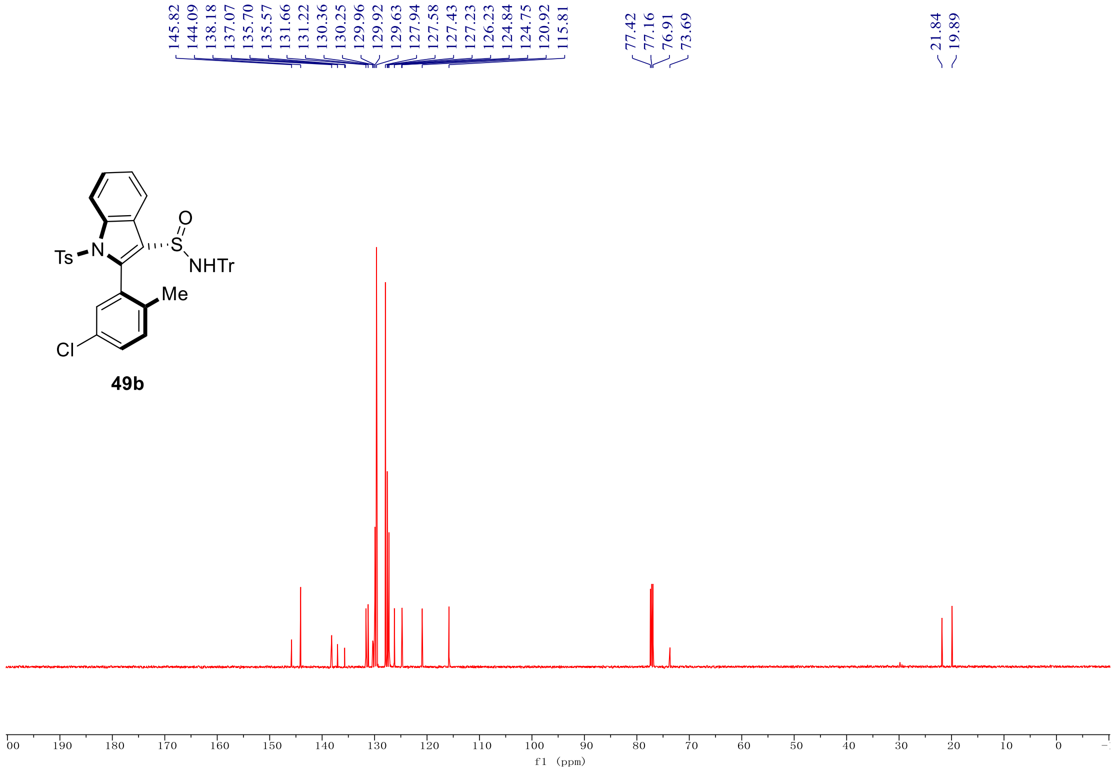
145.82
144.09
138.18
137.07
135.70
135.57
131.66
131.22
130.36
130.25
129.96
129.92
129.63
127.94
127.58
127.43
127.23
126.23
124.84
124.75
120.92
115.81
77.42
77.16
76.91
73.69
21.84
19.89
Ts
N
S
O
NHTr
Me
Cl
49b
f1 (ppm)

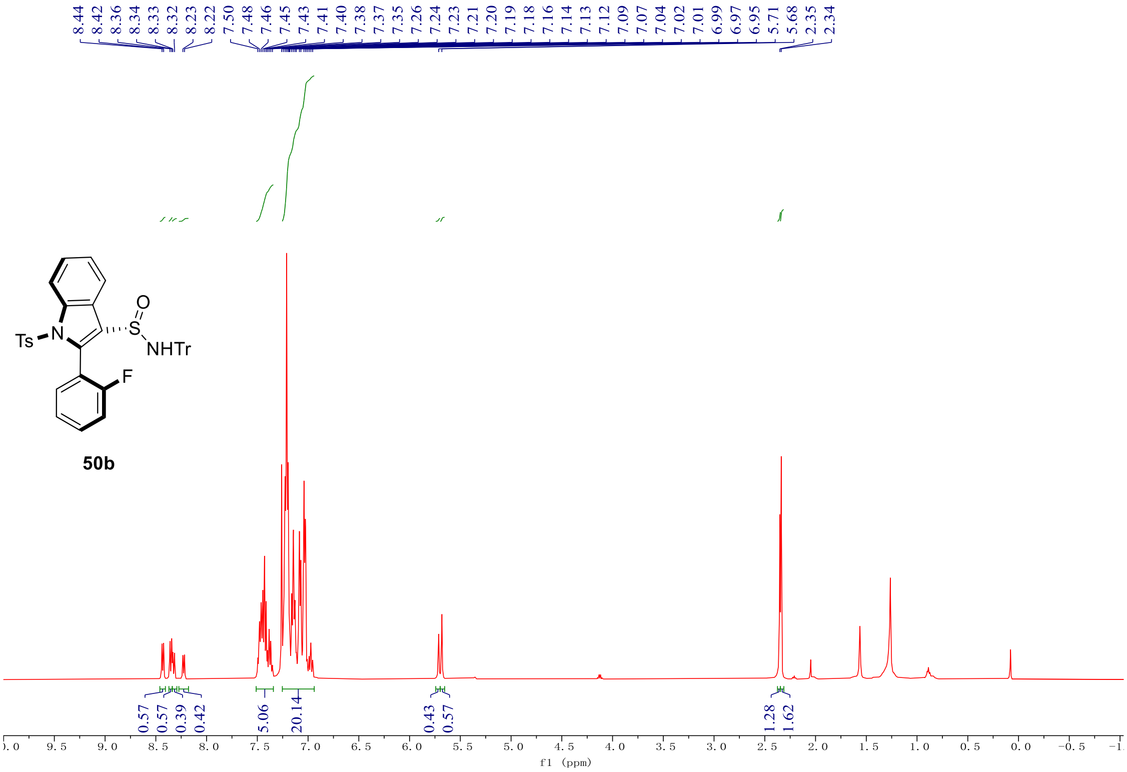
8.44 8.42 8.36 8.34 8.33 8.32 8.23 8.22 7.50 7.48 7.46 7.45 7.43 7.41 7.40 7.38 7.37 7.35 7.26 7.24 7.23 7.21 7.20 7.19 7.18 7.16 7.14 7.13 7.12 7.09 7.07 7.04 7.02 7.01 6.99 6.97 6.95 5.71 5.68 2.35 2.34
Ts
N
S
O
NHTr
F
50b
0.57 0.57 0.39 0.42 5.06 20.14 0.43 0.57 1.28 1.62
f1 (ppm)

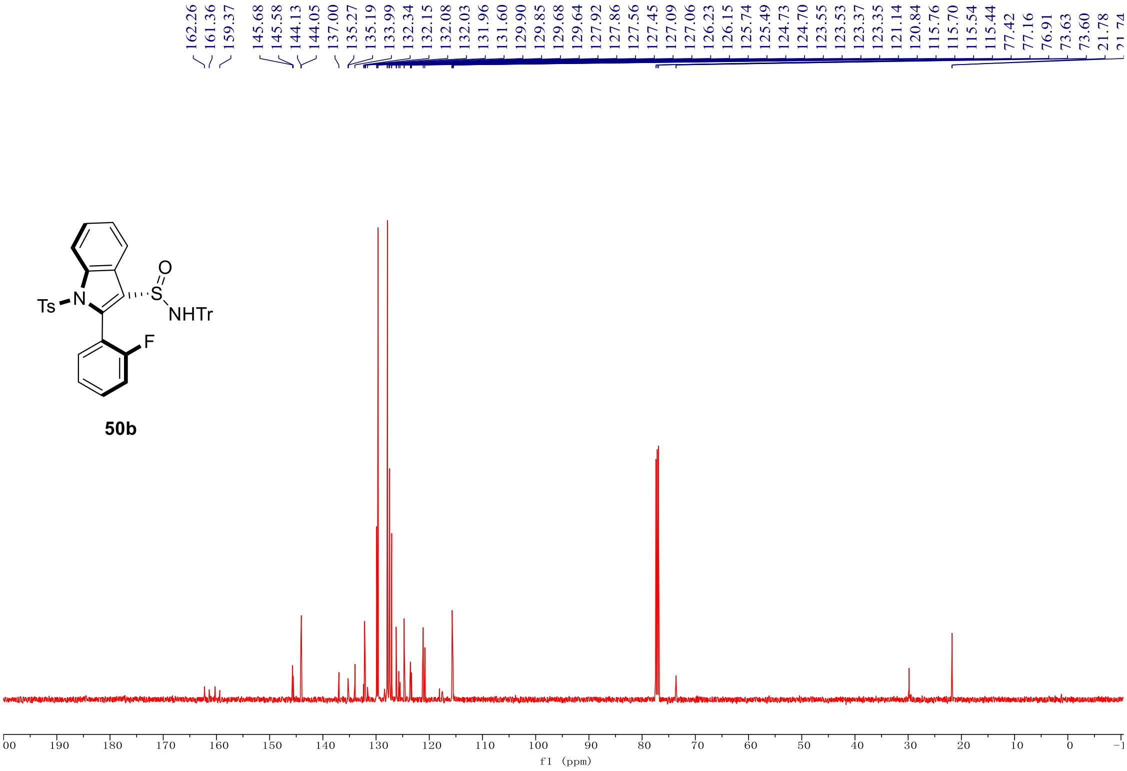
162.26 161.36 159.37 145.68 145.58 144.13 144.05 137.00 135.27 135.19 133.99 132.34 132.15 132.08 132.03 131.96 131.60 129.90 129.85 129.68 129.64 127.92 127.86 127.56 127.45 127.09 127.06 126.23 126.15 125.74 125.49 124.73 124.70 123.55 123.53 123.37 123.35 121.14 120.84 115.76 115.70 115.54 115.44 77.42 77.16 76.91 73.63 73.60 21.78
Ts
N
S
O
NHTr
F
50b
f1 (ppm)

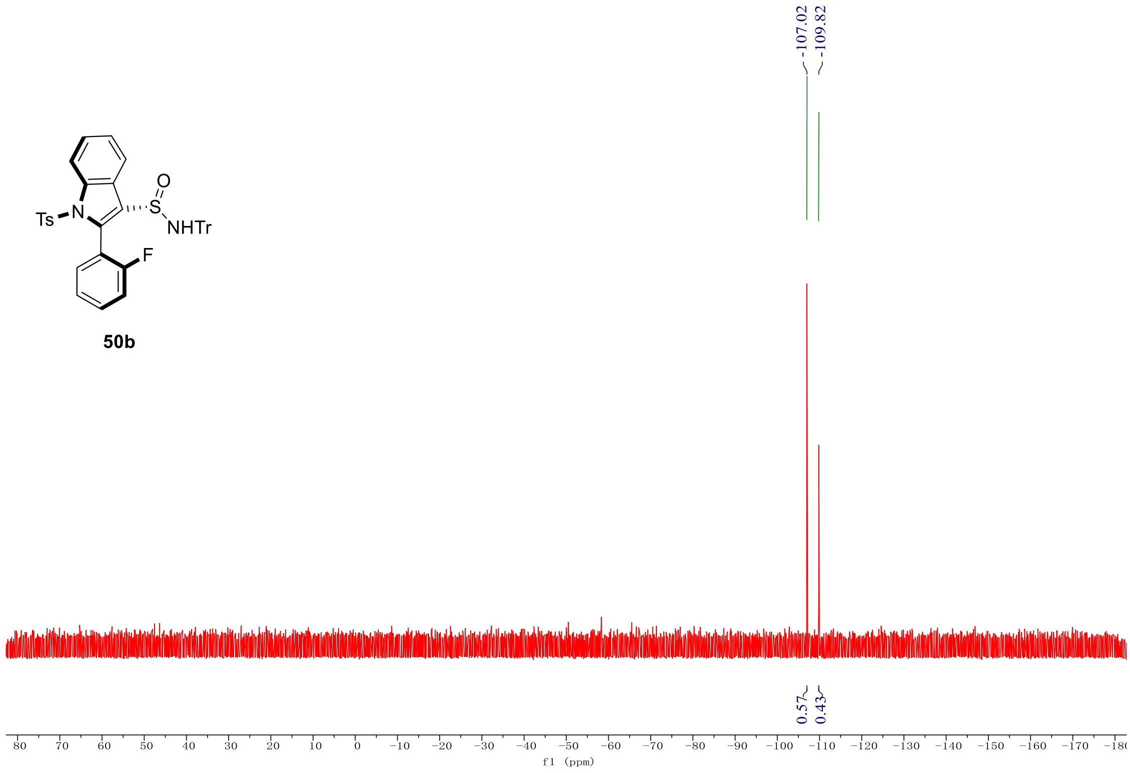
Ts
N
S
O
NHTr
F
-107.02
-109.82
0.57
0.43
80 70 60 50 40 30 20 10 0 −10 −20 −30 −40 −50 −60 −70 −80 −90 −100 −110 −120 −130 −140 −150 −160 −170
f1 (ppm)

50b

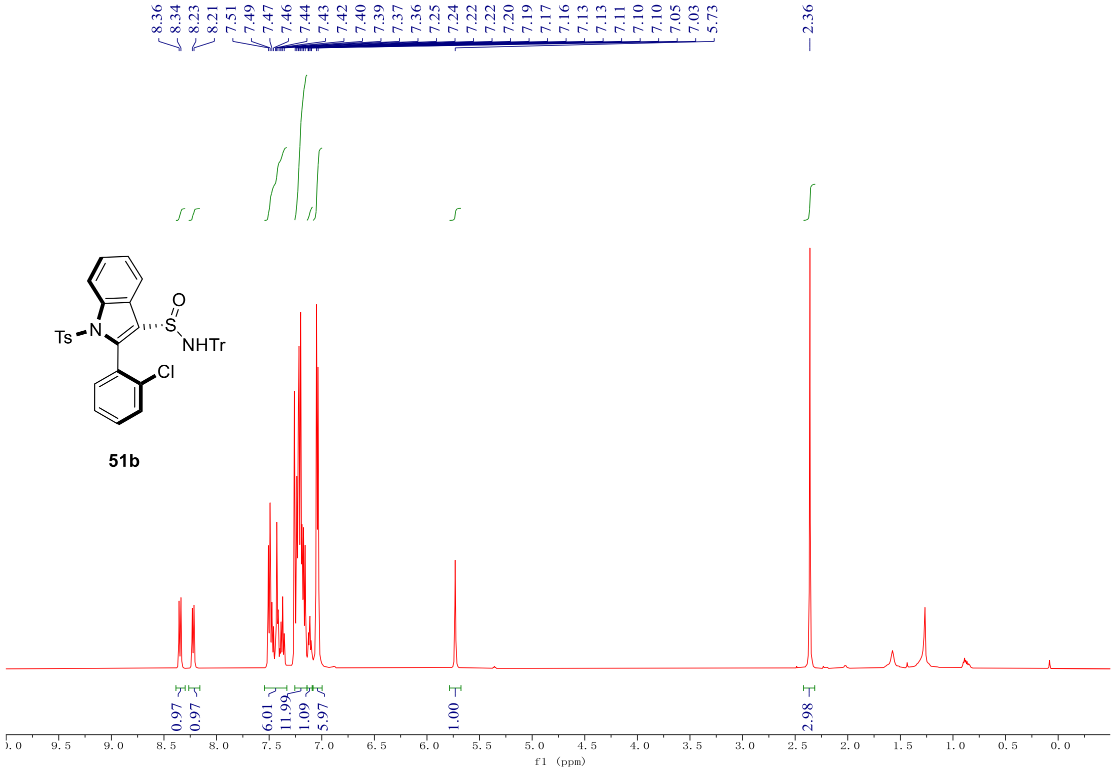
Ts
N
S
O
NHTr
Cl
8.36
8.34
8.23
8.21
7.51
7.49
7.47
7.46
7.44
7.43
7.42
7.40
7.39
7.37
7.36
7.25
7.24
7.22
7.22
7.20
7.19
7.17
7.16
7.13
7.13
7.11
7.10
7.10
7.05
7.03
5.73
2.36
0.97
0.97
6.01
11.99
1.09
5.97
1.00
2.98
9.5 9.0 8.5 8.0 7.5 7.0 6.5 6.0 5.5 5.0 4.5 4.0 3.5 3.0 2.5 2.0 1.5 1.0 0.5 0.0
f1 (ppm)

51b

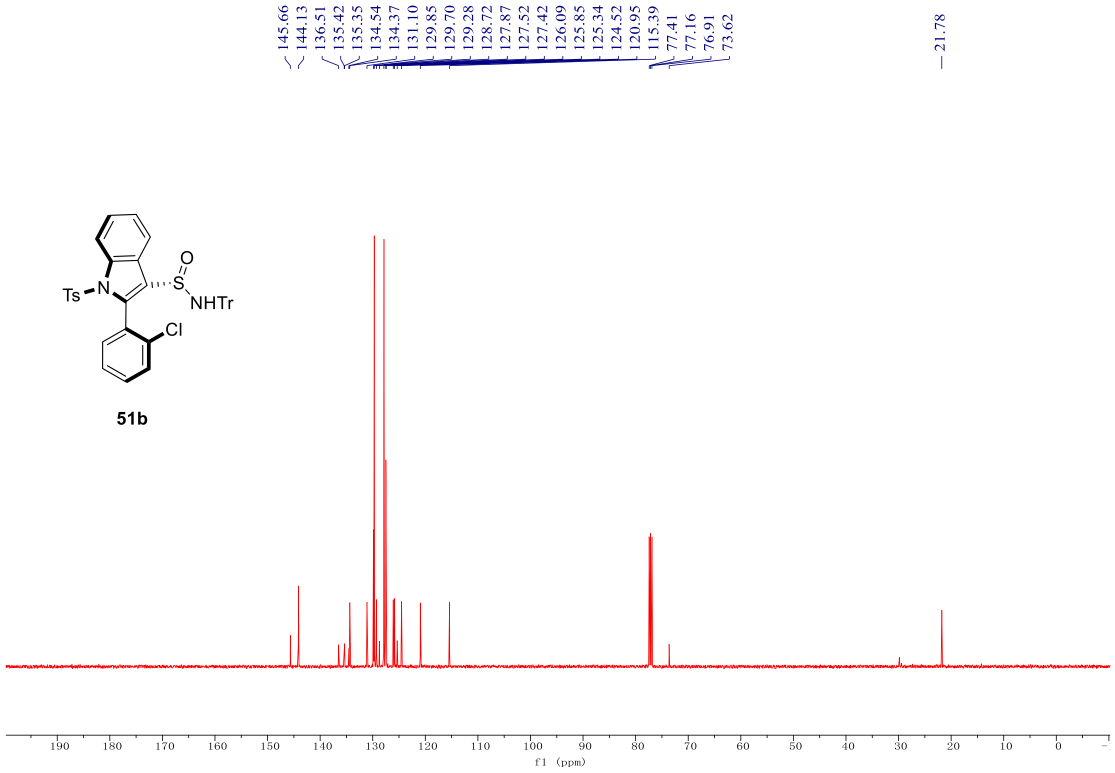
145.66
144.13
136.51
135.42
135.35
134.54
134.37
131.10
129.85
129.70
129.28
128.72
127.87
127.52
127.42
126.09
125.85
125.34
124.52
120.95
115.39
77.41
77.16
76.91
73.62
21.78
Ts
N
S
O
NHTr
Cl
51b
f1 (ppm)

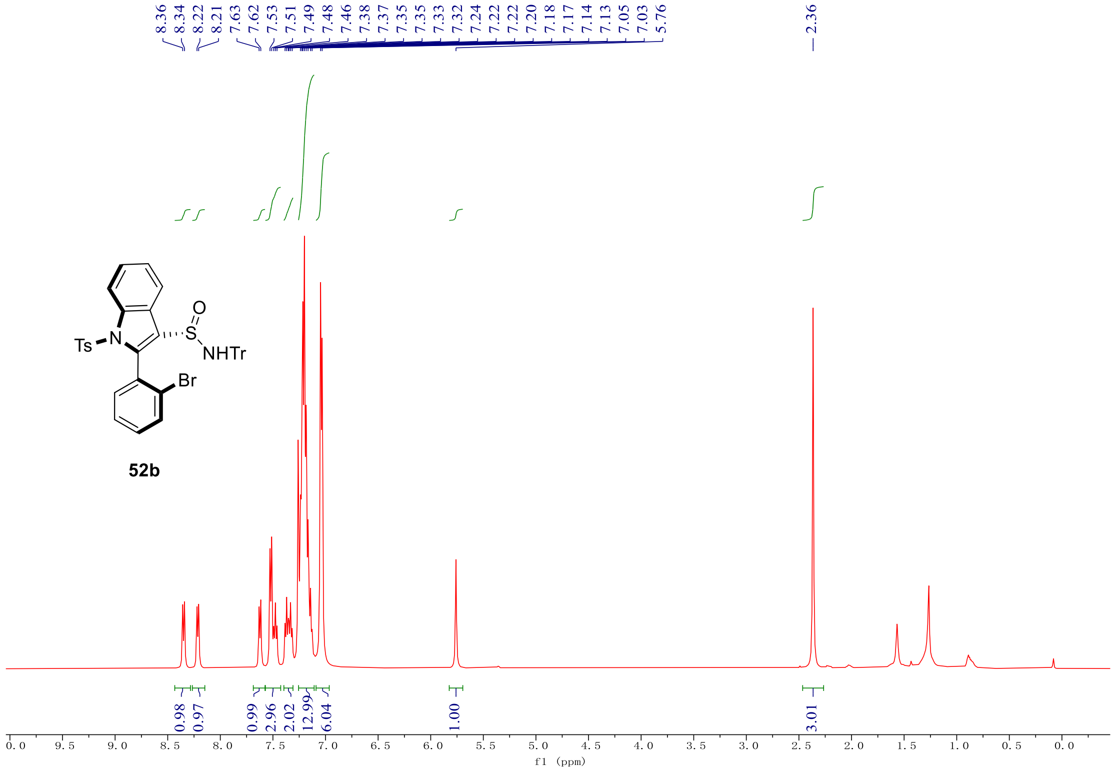
8.36
8.34
8.22
8.21
7.63
7.62
7.53
7.51
7.49
7.48
7.46
7.38
7.37
7.35
7.35
7.33
7.32
7.24
7.22
7.22
7.20
7.18
7.17
7.14
7.13
7.05
7.03
5.76
2.36
Ts
N
S
O
NHTr
Br
52b
0.98
0.97
0.99
2.96
2.02
12.99
6.04
1.00
3.01
f1 (ppm)

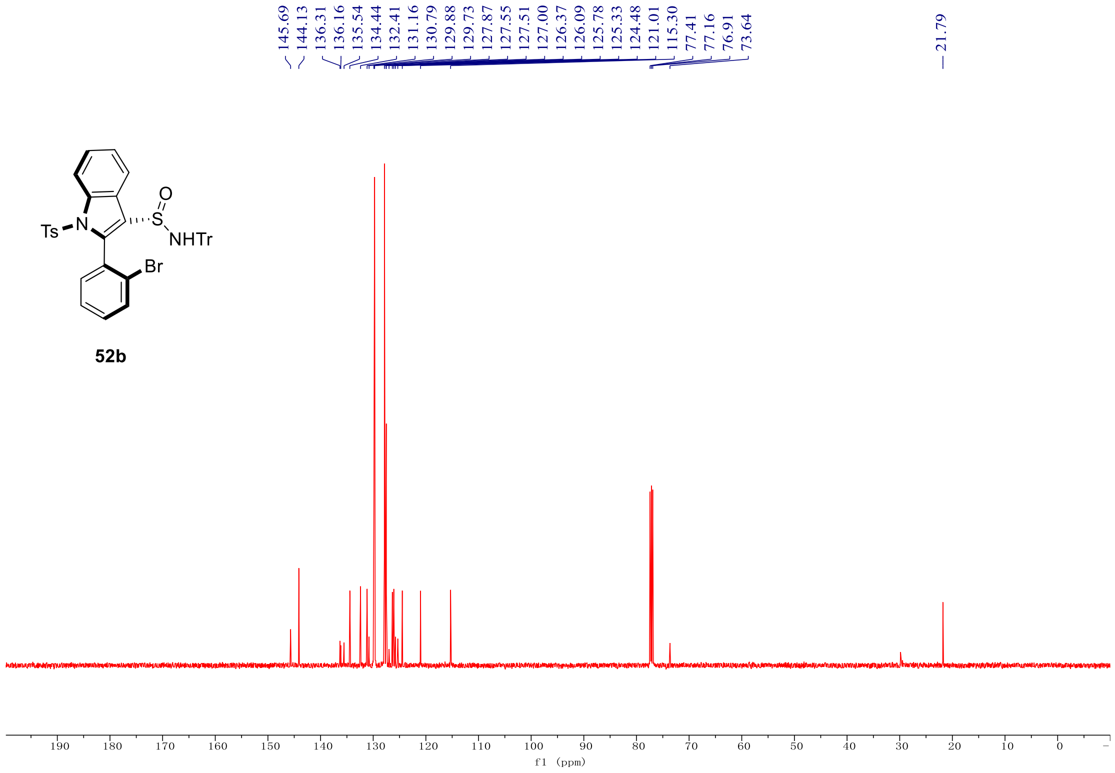
145.69
144.13
136.31
136.16
135.54
134.44
132.41
131.16
130.79
129.88
129.73
127.87
127.55
127.51
127.00
126.37
126.09
125.78
125.33
124.48
121.01
115.30
77.41
77.16
76.91
73.64
21.79
Ts
N
S
O
NHTr
Br
f1 (ppm)

52b

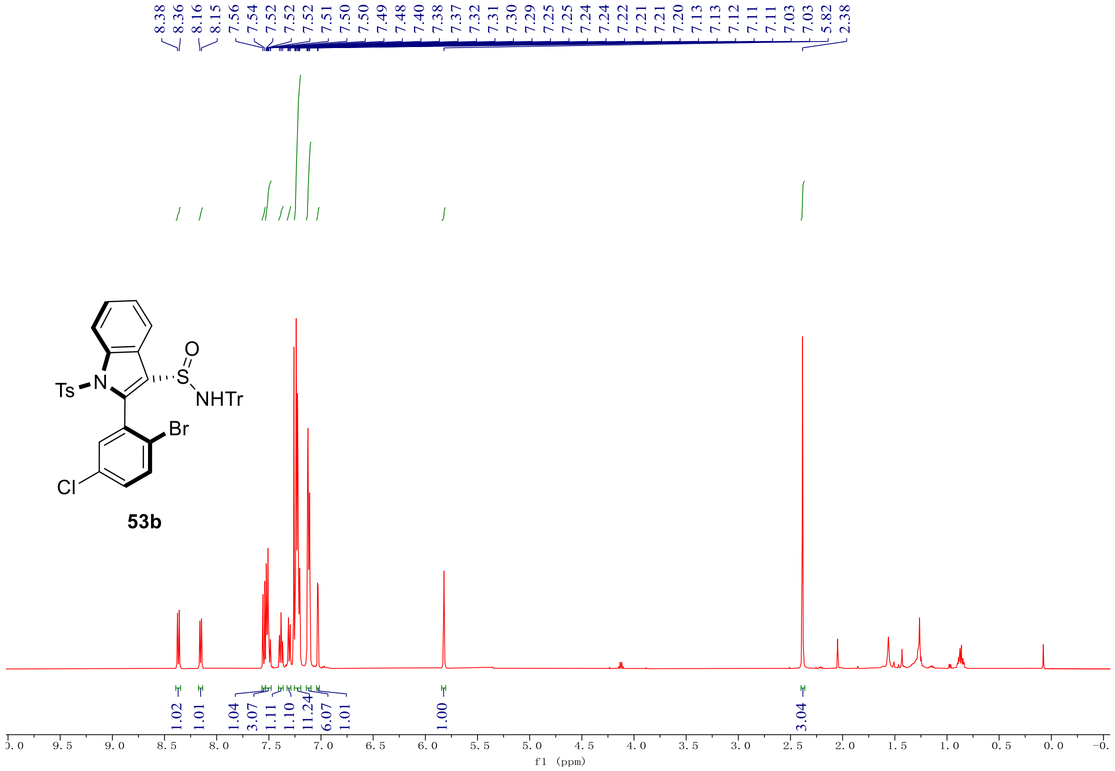
8.38
8.36
8.16
8.15
7.56
7.54
7.52
7.52
7.51
7.50
7.49
7.48
7.40
7.38
7.37
7.32
7.31
7.30
7.29
7.25
7.25
7.24
7.24
7.22
7.21
7.21
7.20
7.13
7.13
7.12
7.11
7.11
7.03
7.03
5.82
2.38
Ts
N
S
O
NHTr
Br
Cl
1.02
1.01
1.04
3.07
1.11
1.10
11.24
6.07
1.01
1.00
3.04
f1 (ppm)

53b

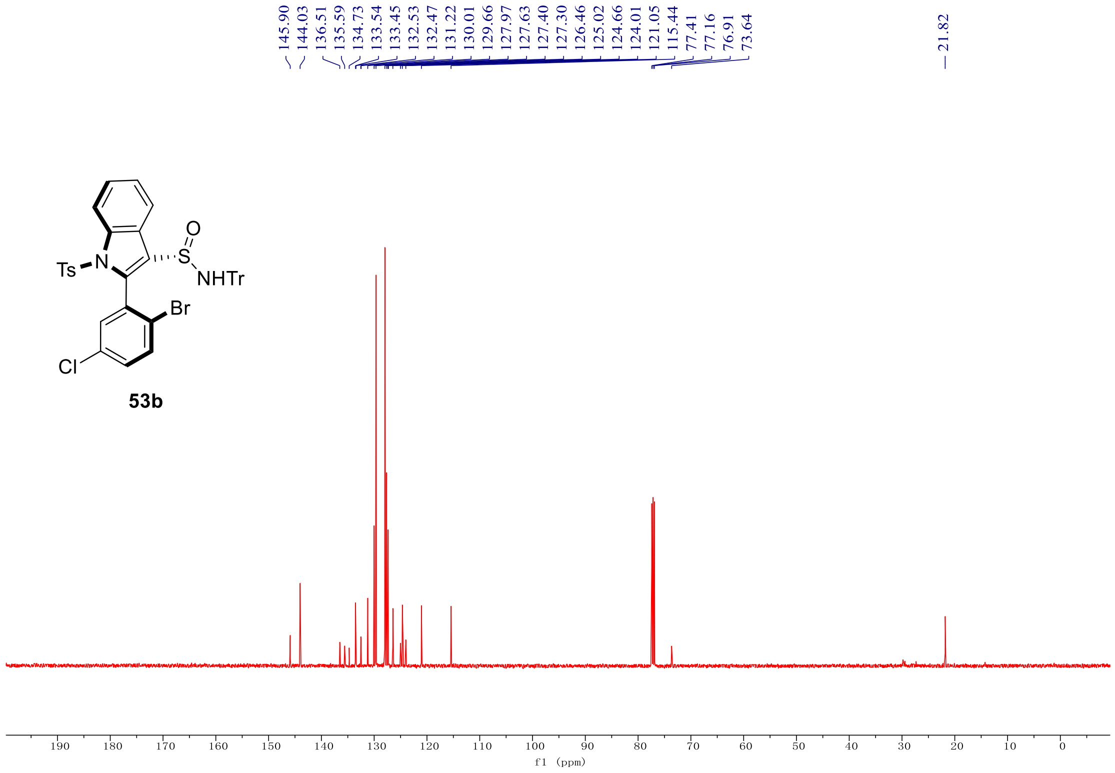

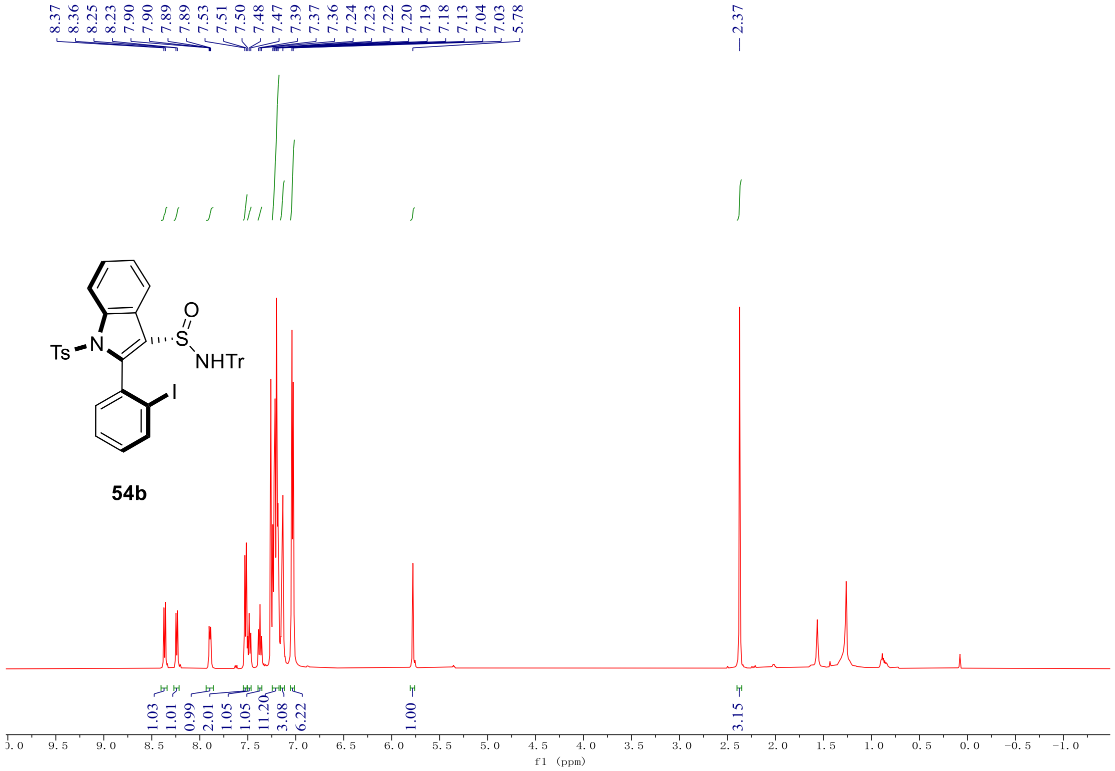

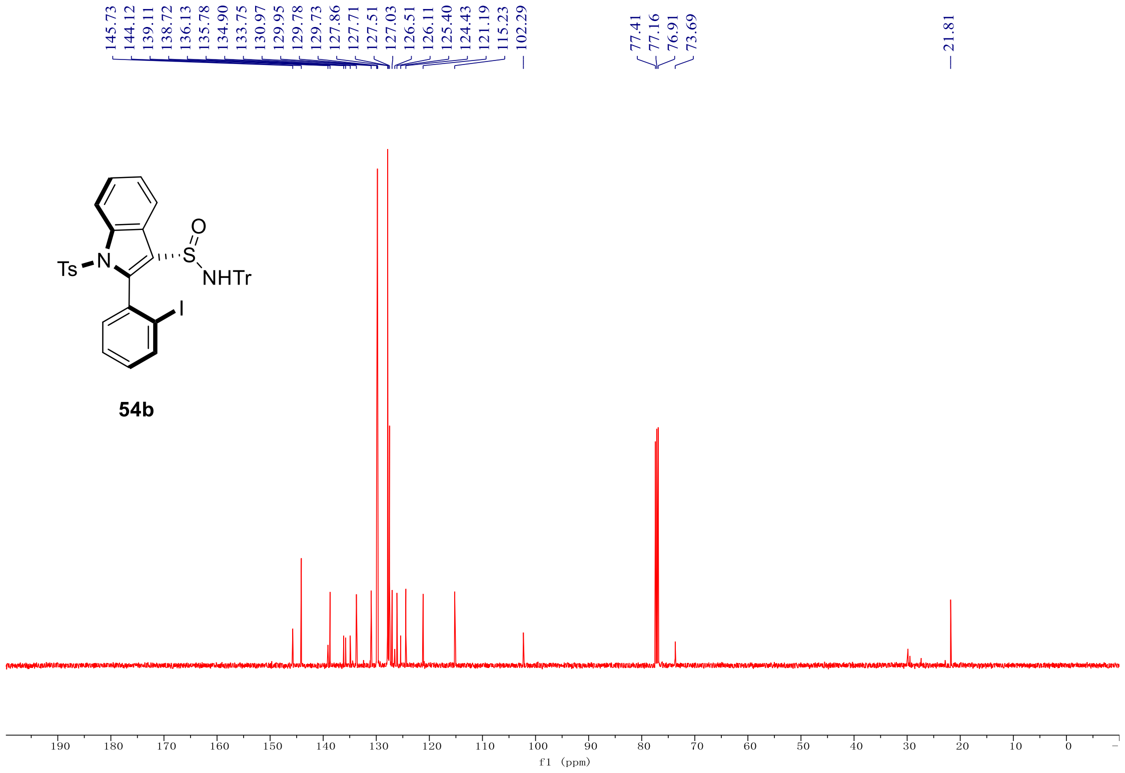

145.73
144.12
139.11
138.72
136.13
135.78
134.90
133.75
130.97
129.95
129.78
129.73
127.86
127.71
127.51
127.03
126.51
126.11
125.40
124.43
121.19
115.23
102.29
77.41
77.16
76.91
73.69
21.81
Ts
N
S
O
NHTr
I
54b
190 180 170 160 150 140 130 120 110 100 90 80 70 60 50 40 30 20 10 0
f1 (ppm)

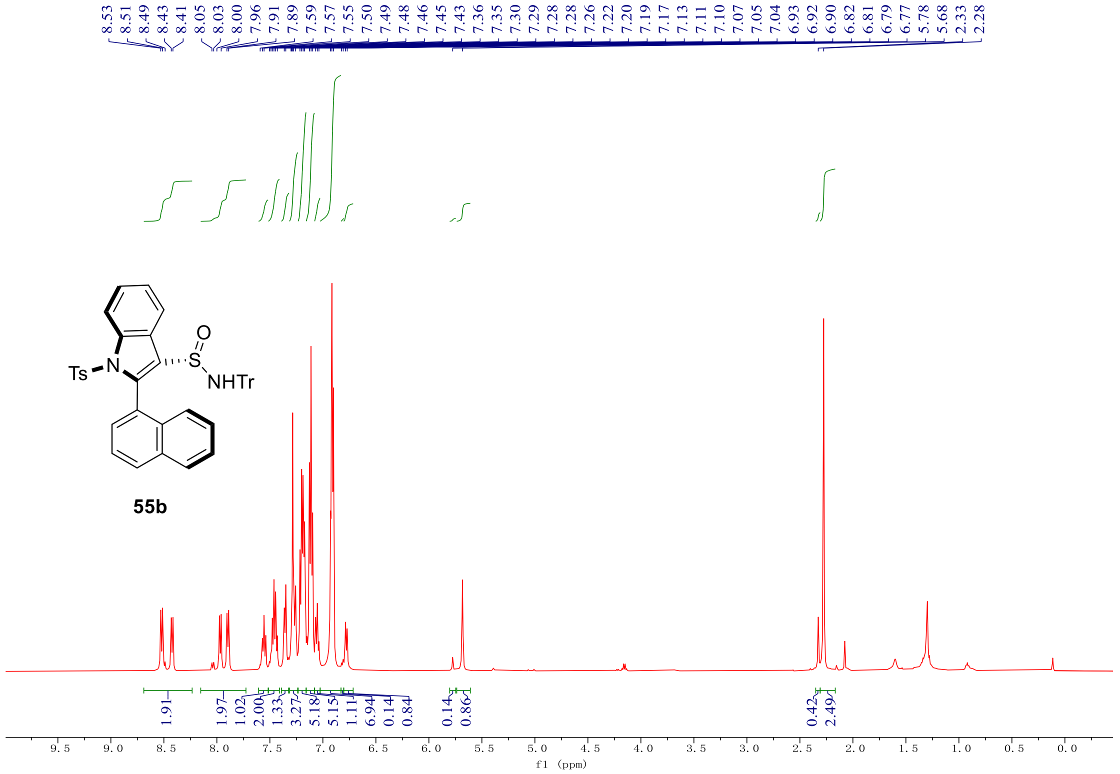

8.53
8.51
8.49
8.43
8.41
8.05
8.03
8.00
7.96
7.91
7.89
7.59
7.57
7.55
7.50
7.49
7.48
7.46
7.45
7.43
7.36
7.35
7.30
7.29
7.28
7.28
7.26
7.22
7.20
7.19
7.17
7.13
7.11
7.10
7.07
7.05
7.04
6.93
6.92
6.90
6.82
6.81
6.79
6.77
5.78
5.68
2.33
2.28
Ts
N
S
O
NHTr
55b
1.91
1.97
1.02
2.00
1.33
3.27
5.18
5.15
1.11
6.94
0.14
0.84
0.14
0.86
0.42
2.49
9.5 9.0 8.5 8.0 7.5 7.0 6.5 6.0 5.5 5.0 4.5 4.0 3.5 3.0 2.5 2.0 1.5 1.0 0.5 0.0
f1 (ppm)

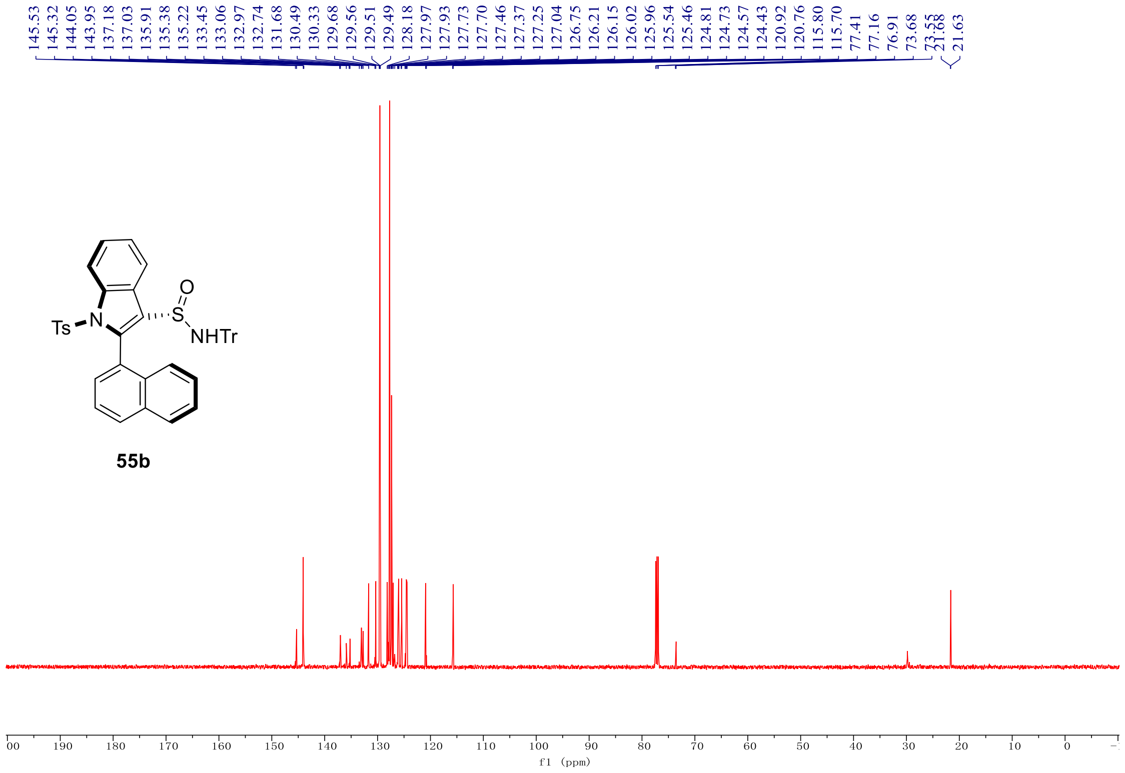
145.53 145.32 144.05 143.95 137.18 137.03 135.91 135.38 135.22 133.45 133.06 132.97 132.74 131.68 130.49 130.33 129.68 129.56 129.51 129.49 128.18 127.97 127.93 127.73 127.70 127.46 127.37 127.25 127.04 126.75 126.21 126.15 126.02 125.96 125.54 125.46 124.81 124.73 124.57 124.43 120.92 120.76 115.80 115.70 77.41 77.16 76.91 73.68 73.55 21.68 21.63
Ts
N
S
O
NHTr
55b
f1 (ppm)

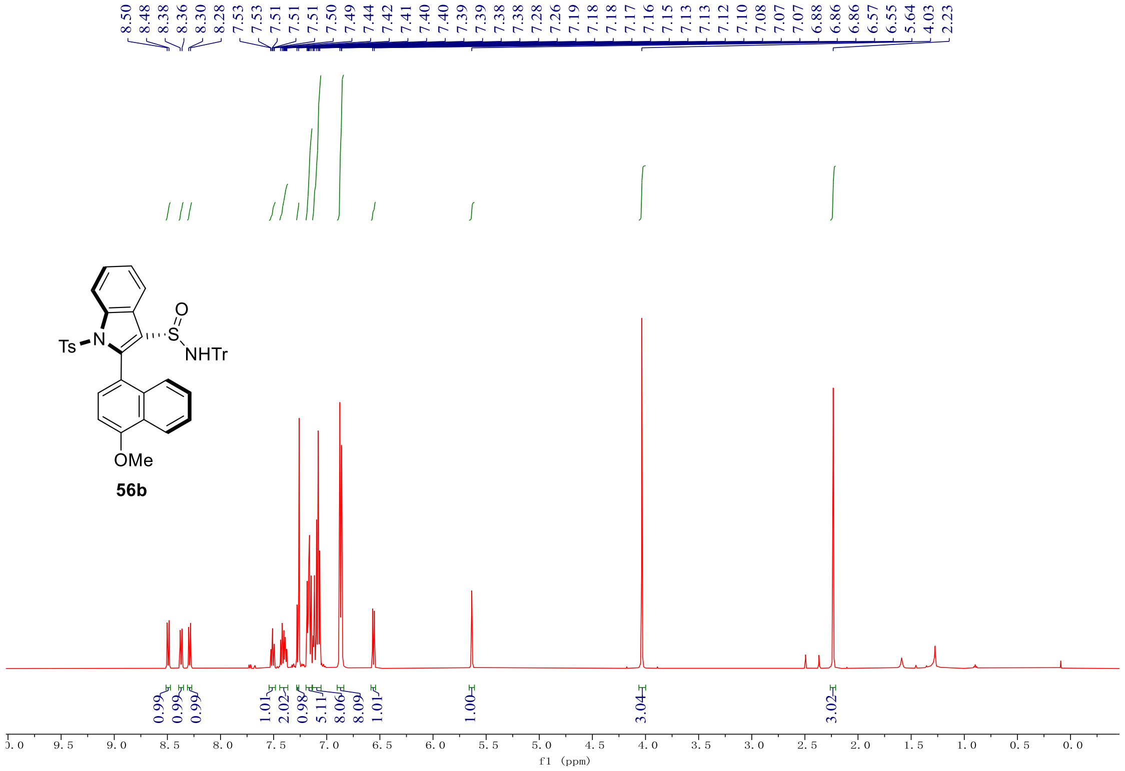
8.50 8.48 8.38 8.36 8.30 8.28 7.53 7.51 7.51 7.50 7.49 7.44 7.42 7.41 7.40 7.39 7.39 7.38 7.38 7.28 7.26 7.19 7.18 7.17 7.16 7.15 7.13 7.12 7.10 7.08 7.07 6.88 6.86 6.57 6.55 5.64 4.03 2.23
Ts
N
S
O
NHTr
OMe
56b
0.99 0.99 0.99 1.01 2.02 0.98 5.11 8.06 8.09 1.01 1.00 3.04 3.02
f1 (ppm)

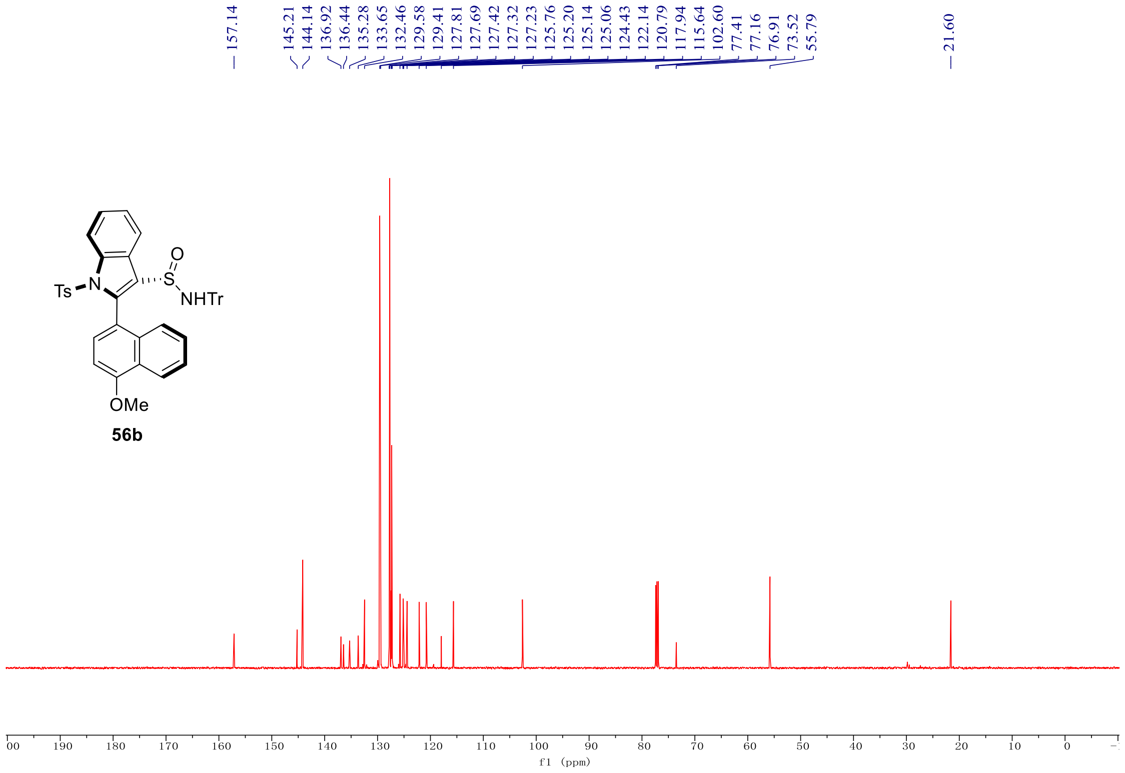
157.14
145.21
144.14
136.92
136.44
135.28
133.65
132.46
129.58
129.41
127.81
127.69
127.42
127.32
127.23
125.76
125.20
125.14
125.06
124.43
122.14
120.79
117.94
115.64
102.60
77.41
77.16
76.91
73.52
55.79
21.60
Ts
N
O
S
NHTr
OMe
56b
f1 (ppm)

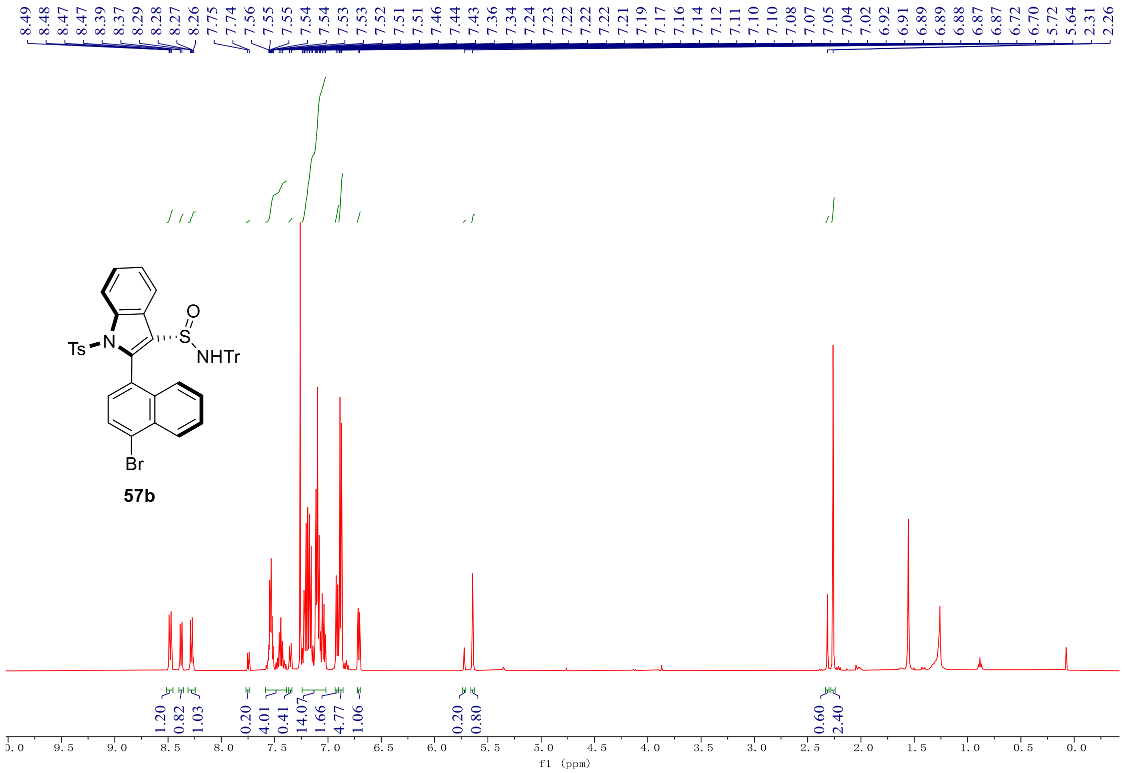
8.49
8.48
8.47
8.47
8.39
8.37
8.29
8.28
8.27
8.26
7.75
7.74
7.56
7.55
7.55
7.54
7.54
7.53
7.53
7.52
7.51
7.51
7.46
7.44
7.43
7.36
7.34
7.24
7.23
7.22
7.22
7.22
7.21
7.19
7.17
7.16
7.14
7.12
7.11
7.10
7.10
7.08
7.07
7.05
7.04
7.02
6.92
6.91
6.89
6.88
6.87
6.87
6.72
6.70
5.72
5.64
2.31
2.26
Ts
N
O
S
NHTr
Br
57b
1.20
0.82
1.03
0.20
4.01
0.41
14.07
1.66
4.77
1.06
0.20
0.80
0.60
2.40
f1 (ppm)

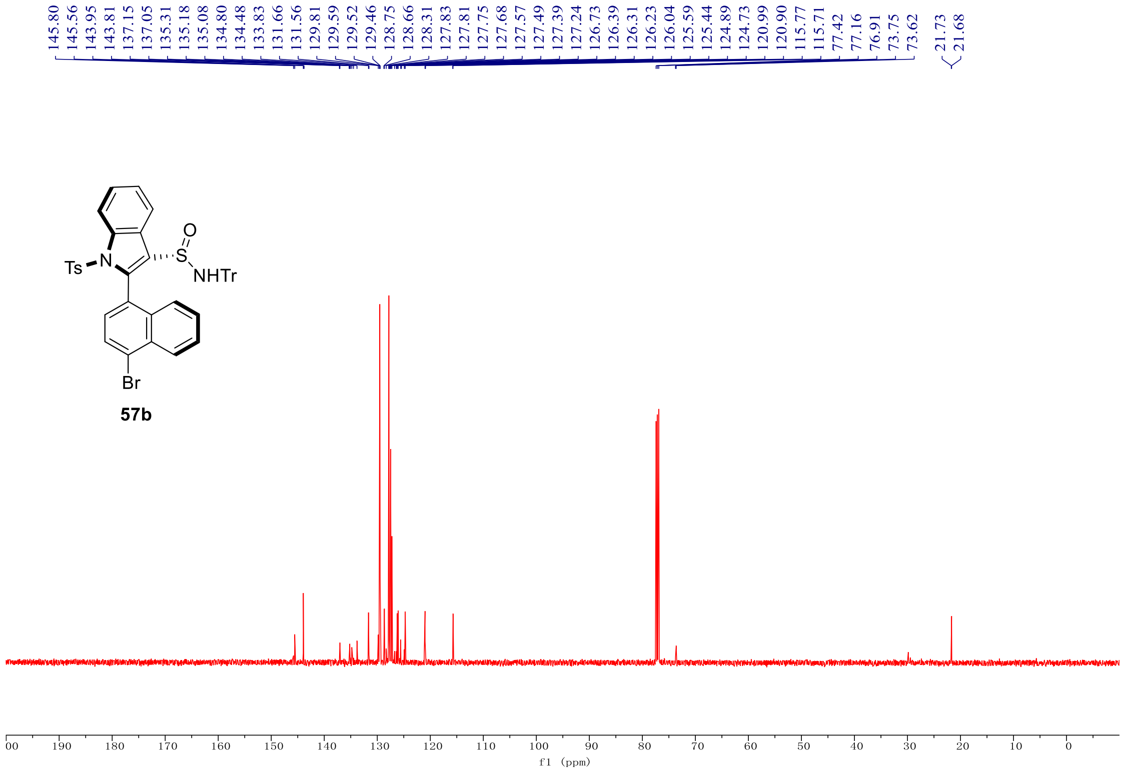
145.80
145.56
143.95
143.81
137.15
137.05
135.31
135.18
135.08
134.80
134.48
133.83
131.66
131.56
129.81
129.59
129.52
129.46
128.75
128.66
128.31
127.83
127.81
127.75
127.68
127.57
127.49
127.39
127.24
126.73
126.39
126.31
126.23
126.04
125.59
125.44
124.89
124.73
120.99
120.90
115.77
115.71
77.42
77.16
76.91
73.75
73.62
21.73
21.68
Ts
N
S
O
NHTr
Br
57b
f1 (ppm)

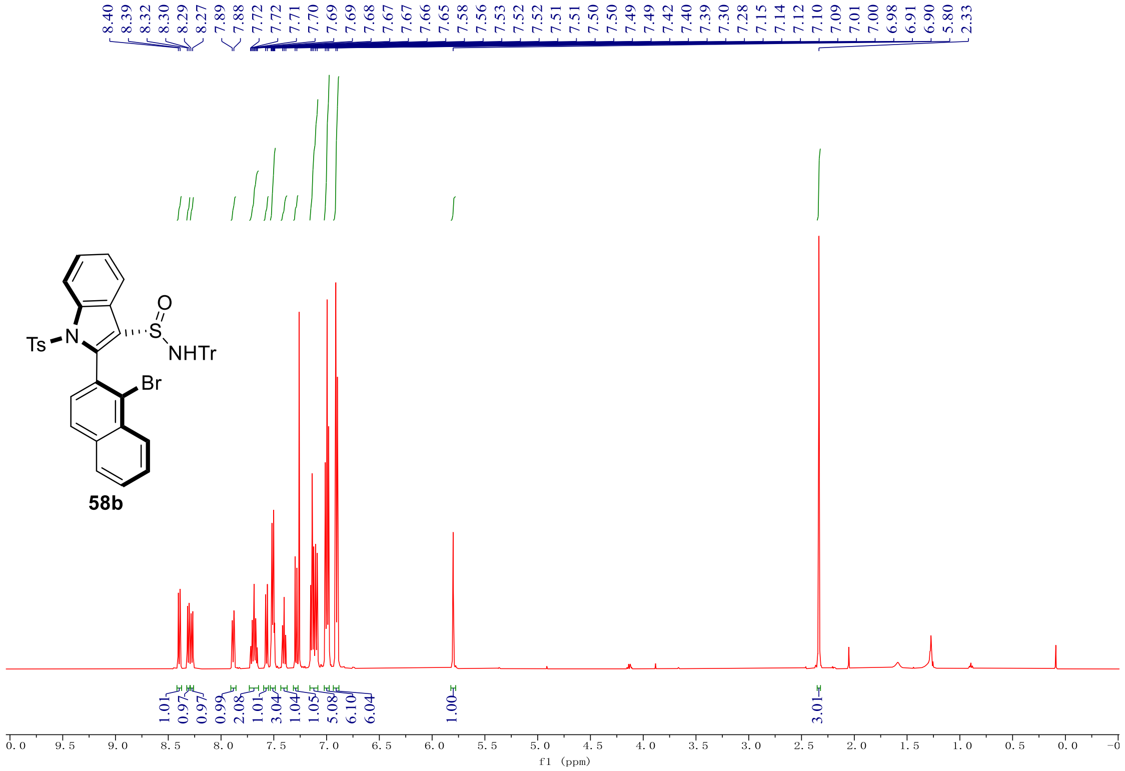
8.40
8.39
8.32
8.30
8.29
8.27
7.89
7.88
7.72
7.72
7.71
7.70
7.69
7.69
7.68
7.67
7.67
7.66
7.65
7.58
7.56
7.53
7.52
7.52
7.51
7.51
7.50
7.49
7.49
7.42
7.40
7.39
7.30
7.28
7.15
7.14
7.12
7.10
7.09
7.01
7.00
6.98
6.91
6.90
5.80
2.33
Ts
N
S
O
NHTr
Br
58b
1.01
0.97
0.97
0.99
2.08
1.01
3.04
1.04
1.05
5.08
6.10
6.04
1.00
3.01
f1 (ppm)

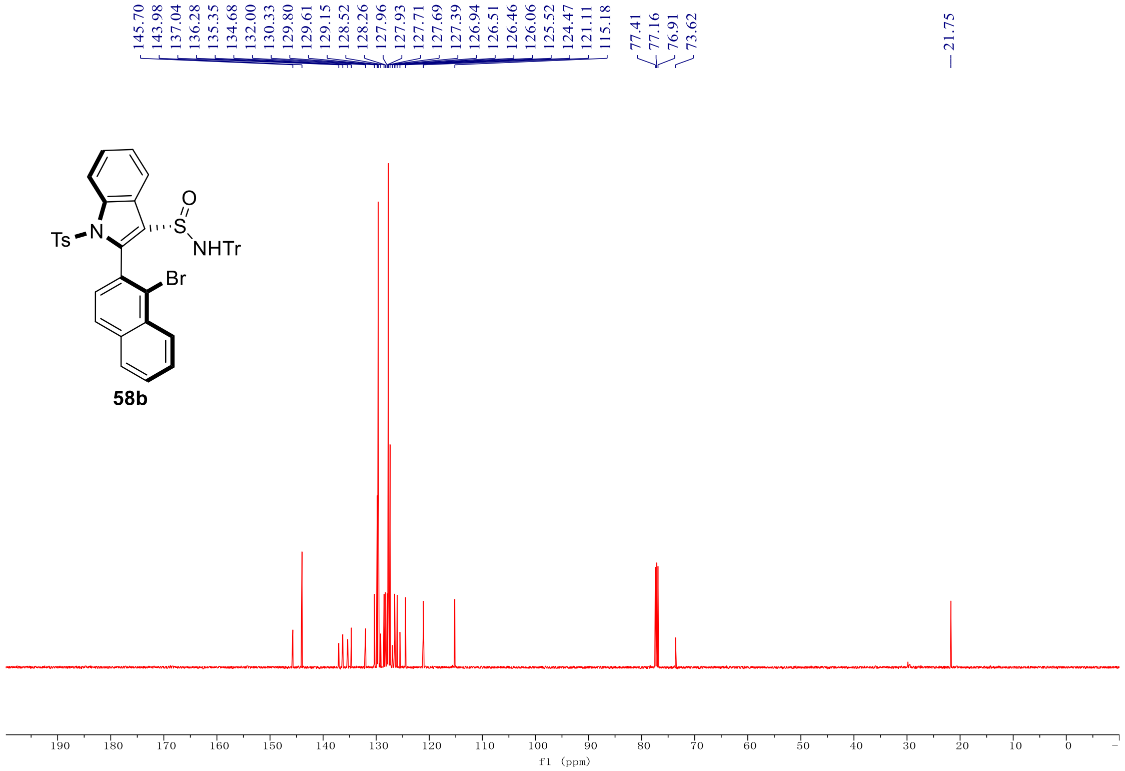
145.70
143.98
137.04
136.28
135.35
134.68
132.00
130.33
129.80
129.61
129.15
128.52
128.26
127.96
127.93
127.71
127.69
127.39
126.94
126.51
126.46
126.06
125.52
124.47
121.11
115.18
77.41
77.16
76.91
73.62
21.75
O
S
NHTr
Ts
N
Br
58b
f1 (ppm)

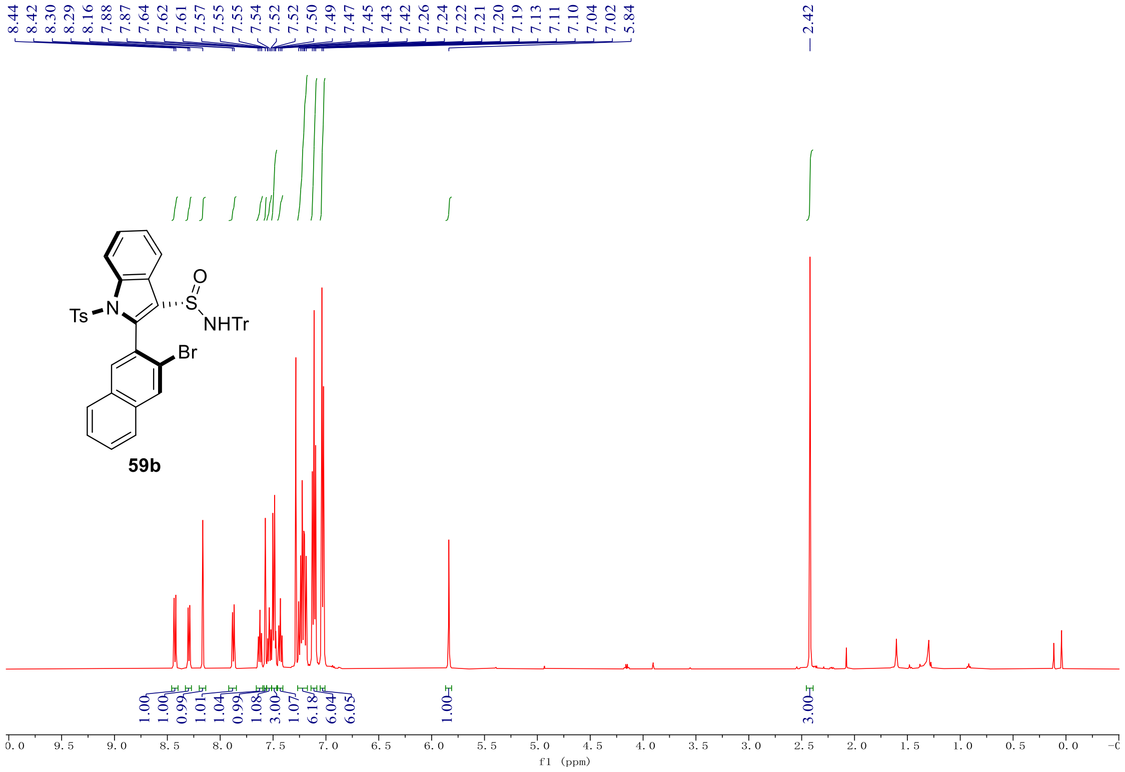
8.44
8.42
8.30
8.29
8.16
7.88
7.87
7.64
7.62
7.61
7.57
7.55
7.55
7.54
7.52
7.52
7.50
7.49
7.47
7.45
7.43
7.42
7.26
7.24
7.22
7.21
7.20
7.19
7.13
7.11
7.10
7.04
7.02
5.84
2.42
O
S
NHTr
Ts
N
Br
59b
1.00
1.00
0.99
1.01
1.04
0.99
1.08
3.00
1.07
6.18
6.04
6.05
1.00
3.00
f1 (ppm)

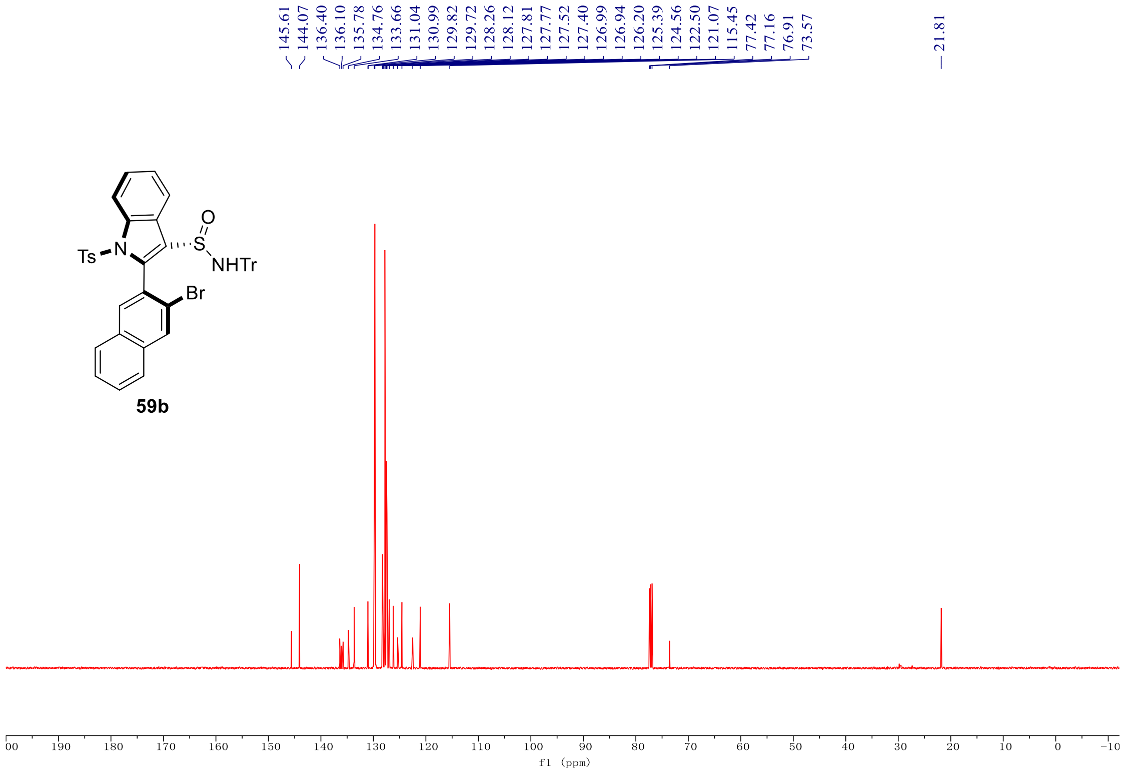
145.61
144.07
136.40
136.10
135.78
134.76
133.66
131.04
130.99
129.82
129.72
128.26
128.12
127.81
127.77
127.52
127.40
126.99
126.94
126.20
125.39
124.56
122.50
121.07
115.45
77.42
77.16
76.91
73.57
21.81
Ts
N
S
O
NHTr
Br
59b
f1 (ppm)

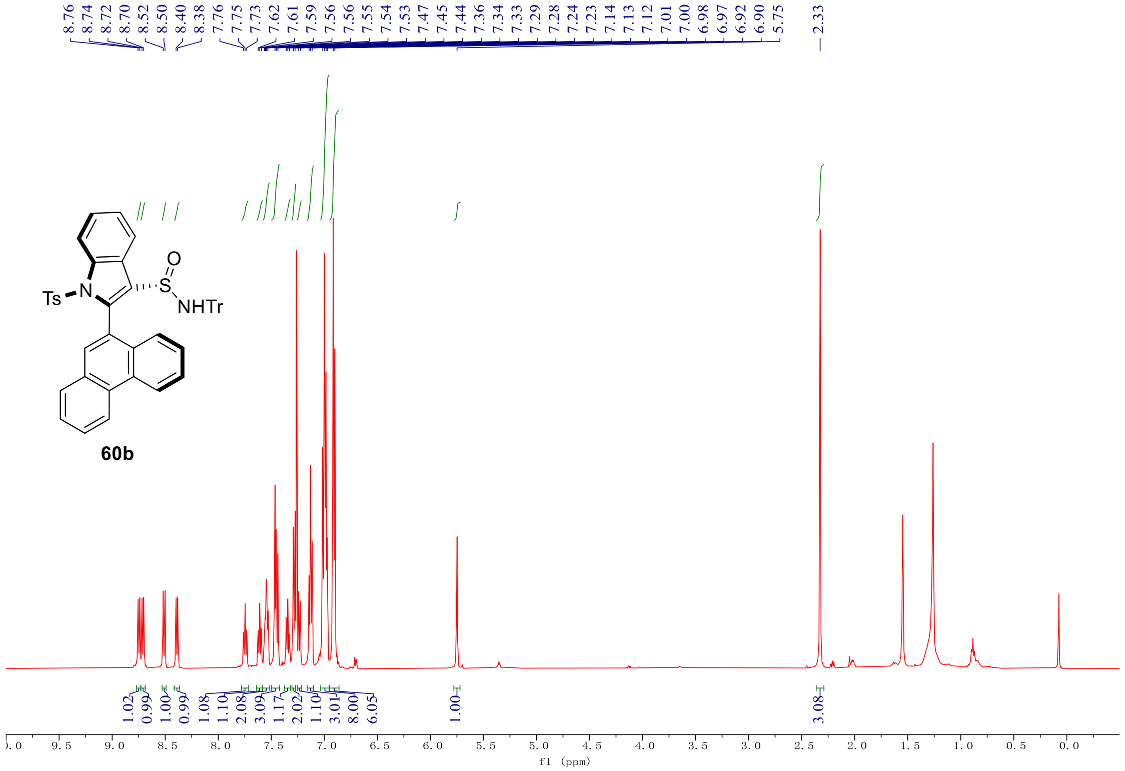
8.76
8.74
8.72
8.70
8.52
8.50
8.40
8.38
7.76
7.75
7.73
7.62
7.61
7.59
7.56
7.56
7.55
7.54
7.53
7.47
7.45
7.44
7.36
7.34
7.33
7.29
7.28
7.24
7.23
7.14
7.13
7.12
7.01
7.00
6.98
6.97
6.92
6.90
5.75
2.33
Ts
N
S
O
NHTr
60b
1.02
0.99
1.00
0.99
1.08
1.10
2.08
3.09
1.17
2.02
1.10
3.01
8.00
6.05
1.00
3.08
f1 (ppm)

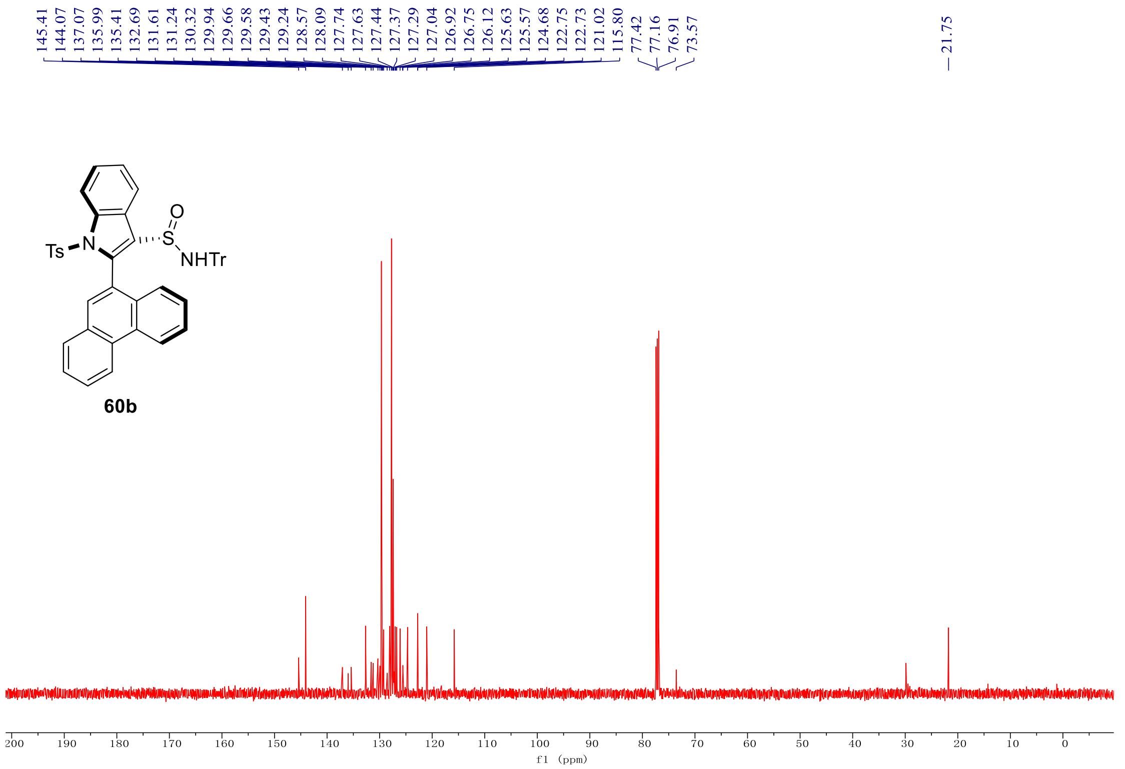
145.41 144.07 137.07 135.99 135.41 132.69 131.61 131.24 130.32 129.94 129.66 129.58 129.43 129.24 128.57 128.09 127.74 127.63 127.44 127.37 127.29 127.04 126.92 126.75 126.12 125.63 125.57 124.68 122.75 122.73 121.02 115.80
77.42 77.16 76.91 73.57
21.75
O
S
Ts
N
NHTr
60b
200 190 180 170 160 150 140 130 120 110 100 90 80 70 60 50 40 30 20 10 0
f1 (ppm)

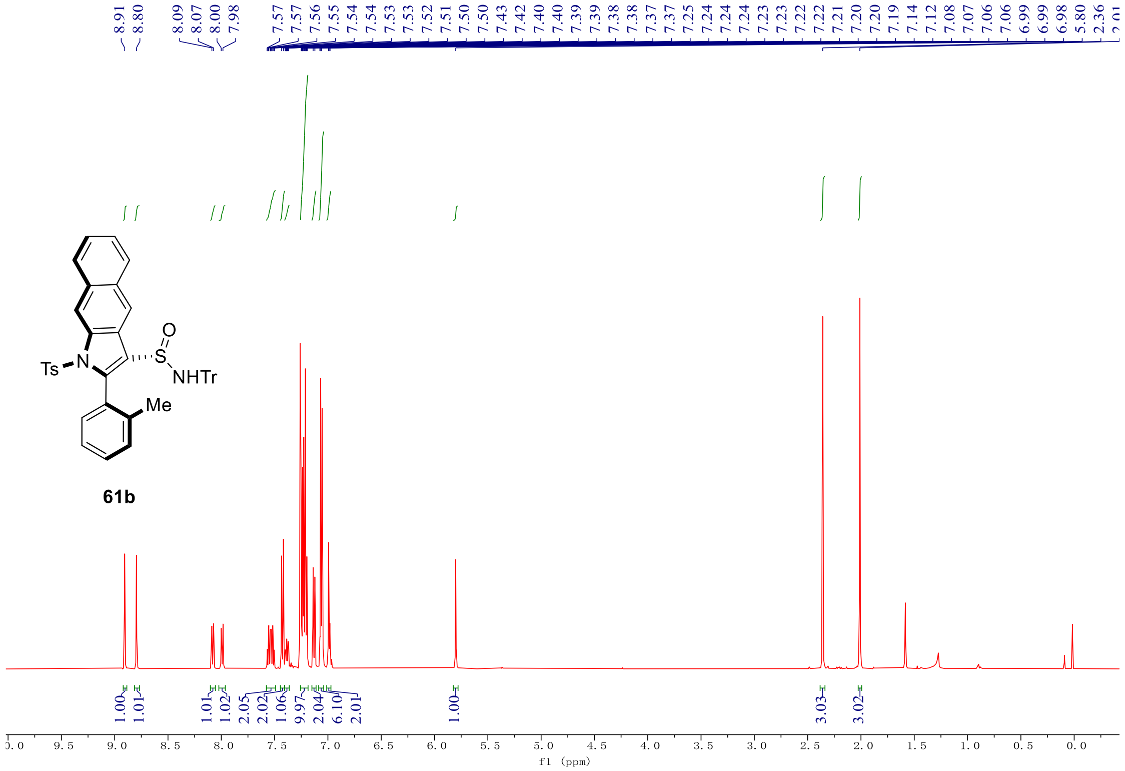
8.91 8.80 8.09 8.07 8.00 7.98 7.57 7.57 7.56 7.55 7.54 7.54 7.53 7.53 7.52 7.51 7.50 7.50 7.43 7.42 7.40 7.40 7.39 7.39 7.38 7.38 7.37 7.37 7.25 7.24 7.24 7.24 7.23 7.23 7.22 7.22 7.21 7.20 7.20 7.19 7.14 7.12 7.08 7.07 7.06 6.99 6.99 6.98 5.80 2.36 [illegible]
O
S
Ts
N
NHTr
Me
61b
1.00 1.01 1.01 1.02 2.05 2.02 1.06 9.97 2.04 6.10 2.01 1.00 3.03 3.02
9.5 9.0 8.5 8.0 7.5 7.0 6.5 6.0 5.5 5.0 4.5 4.0 3.5 3.0 2.5 2.0 1.5 1.0 0.5 0.0
f1 (ppm)

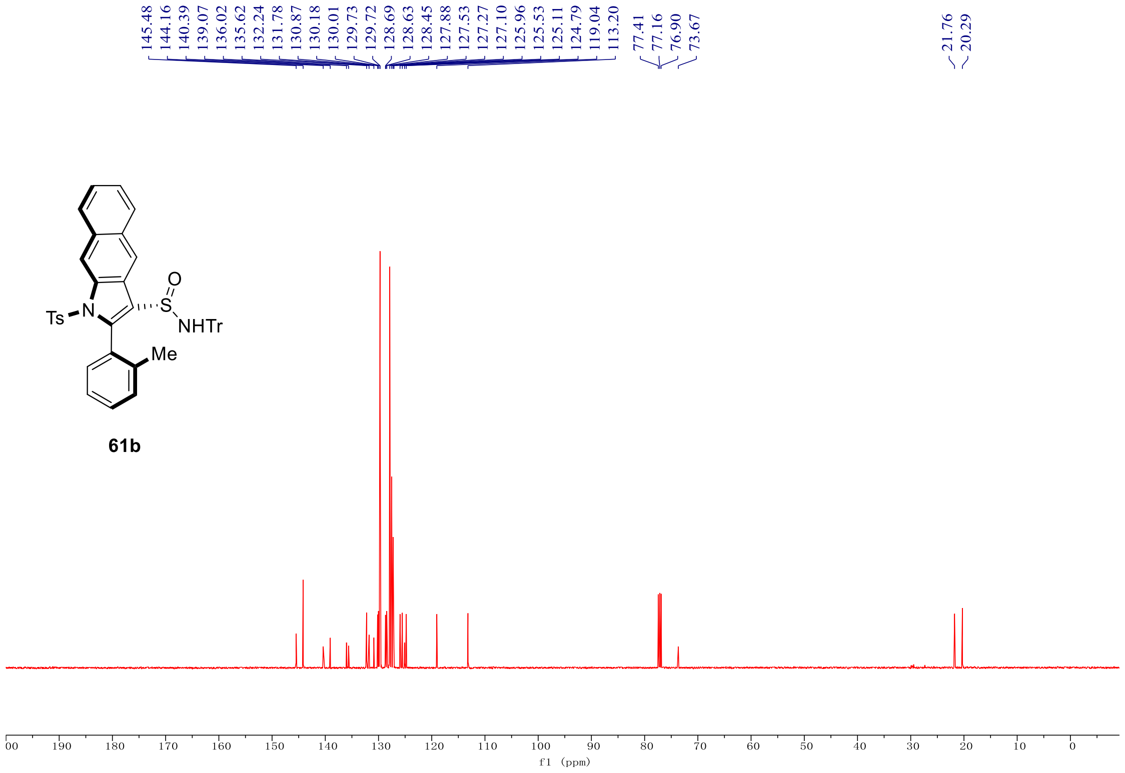

61b

8.3 Synthetic Transformations

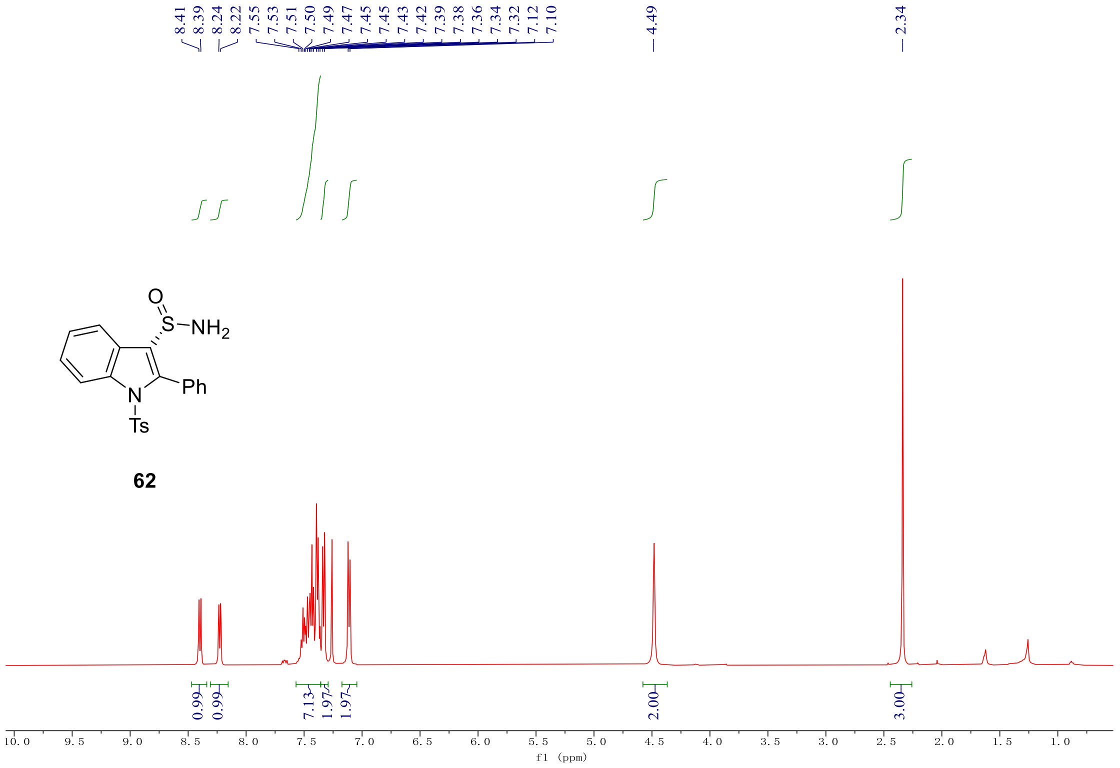

62

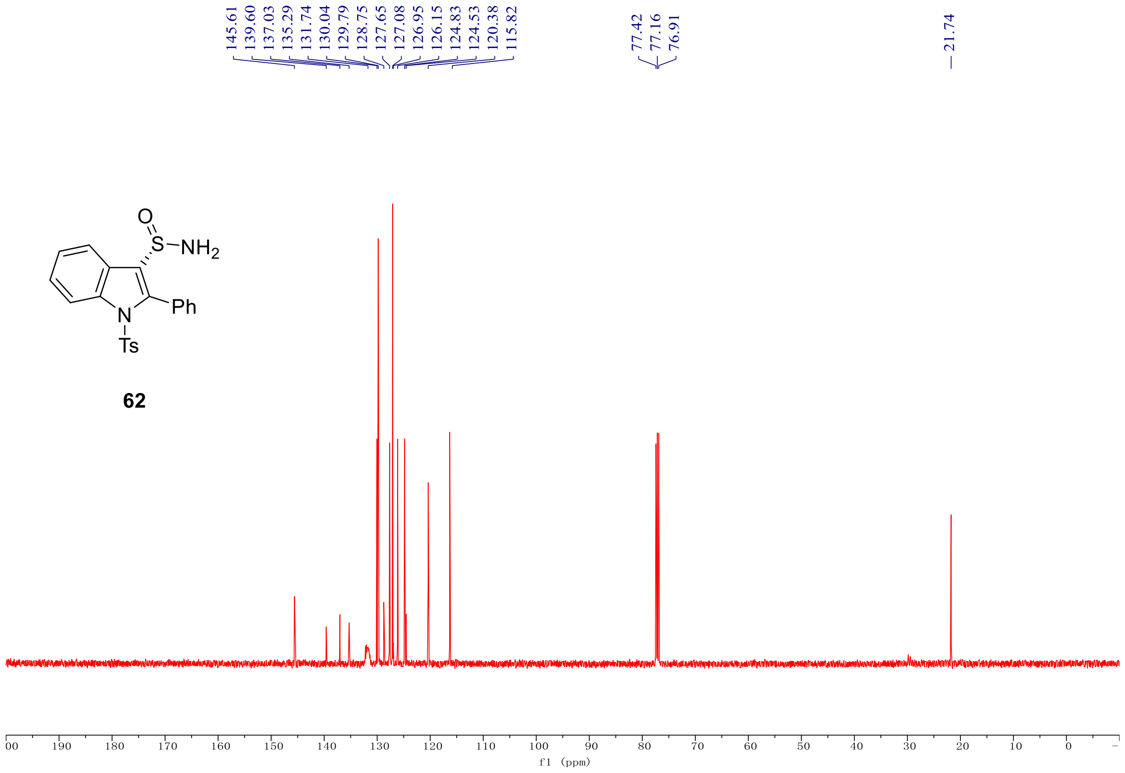
145.61
139.60
137.03
135.29
131.74
130.04
129.79
128.75
127.65
127.08
126.95
126.15
124.83
124.53
120.38
115.82
77.42
77.16
76.91
21.74
62
f1 (ppm)

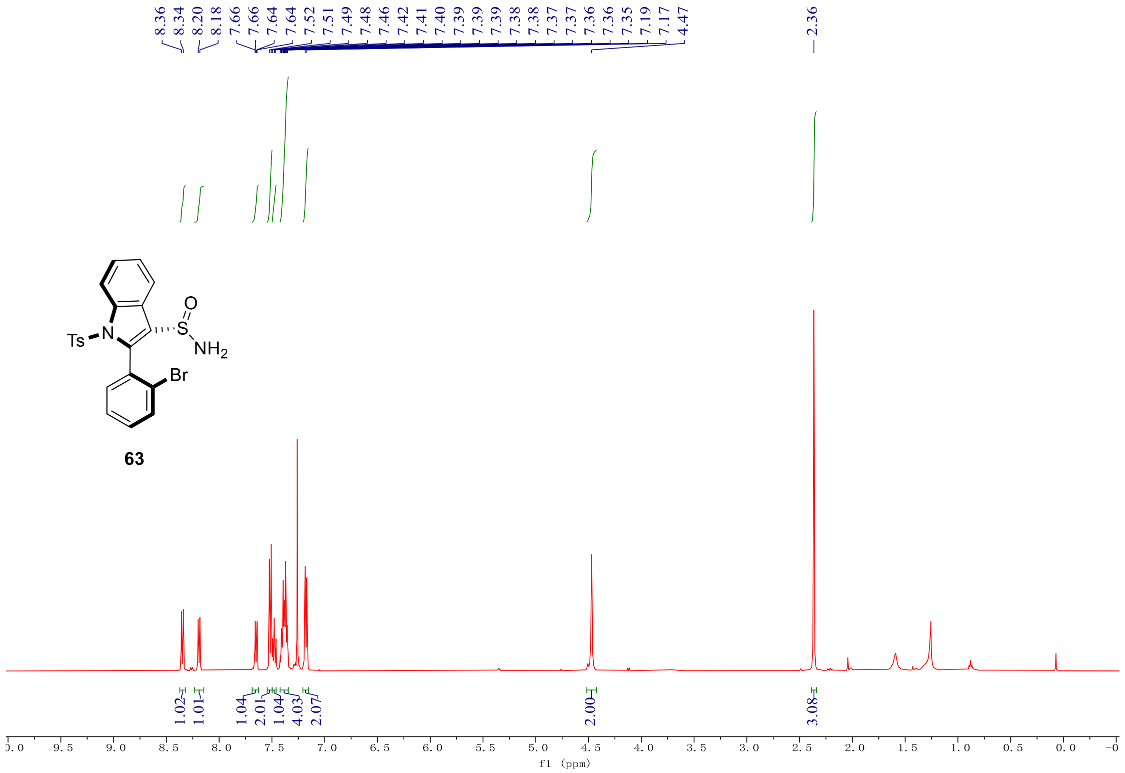
8.36
8.34
8.20
8.18
7.66
7.64
7.64
7.52
7.51
7.49
7.48
7.46
7.42
7.41
7.40
7.39
7.39
7.38
7.38
7.37
7.37
7.36
7.35
7.19
7.17
4.47
2.36
63
1.02
1.01
1.04
2.01
1.04
4.03
2.07
2.00
3.08
f1 (ppm)

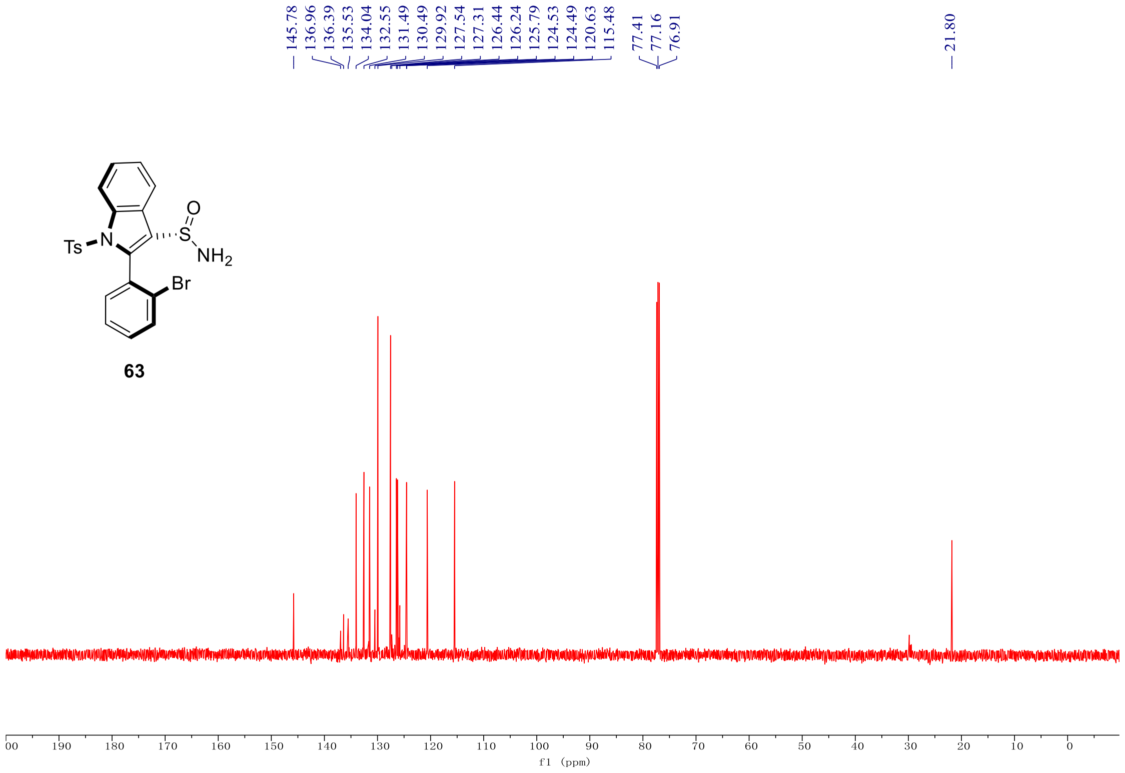

145.78
136.96
136.39
135.53
134.04
132.55
131.49
130.49
129.92
127.54
127.31
126.44
126.24
125.79
124.53
124.49
120.63
115.48
77.41
77.16
76.91
21.80
Ts
N
O
S
NH2
Br
f1 (ppm)

63

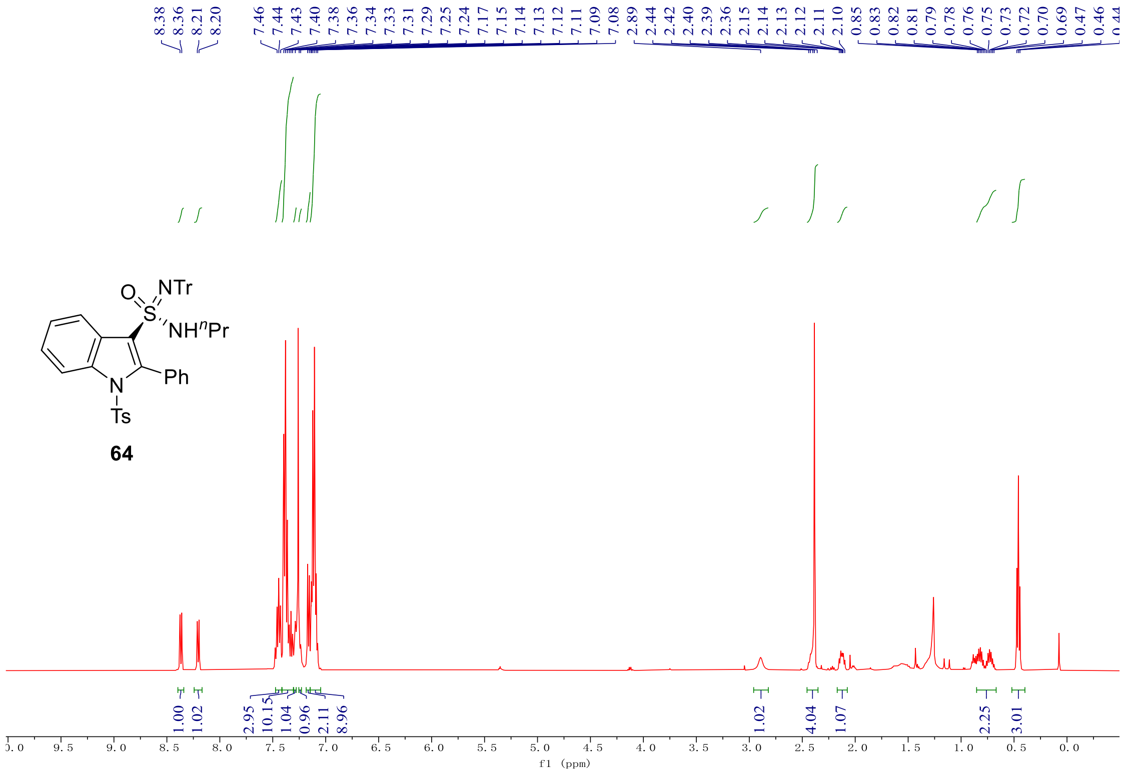

8.38
8.36
8.21
8.20
7.46
7.44
7.43
7.40
7.38
7.36
7.34
7.33
7.31
7.29
7.25
7.24
7.17
7.15
7.14
7.13
7.12
7.11
7.09
7.08
2.89
2.44
2.42
2.40
2.39
2.36
2.15
2.14
2.13
2.12
2.11
2.10
0.85
0.83
0.82
0.81
0.79
0.78
0.76
0.75
0.73
0.72
0.70
0.69
0.47
0.46
O
NTr
S
NH^{n}Pr
Ph
N
Ts
1.00
1.02
2.95
10.15
1.04
0.96
2.11
8.96
1.02
4.04
1.07
2.25
3.01
f1 (ppm)

64

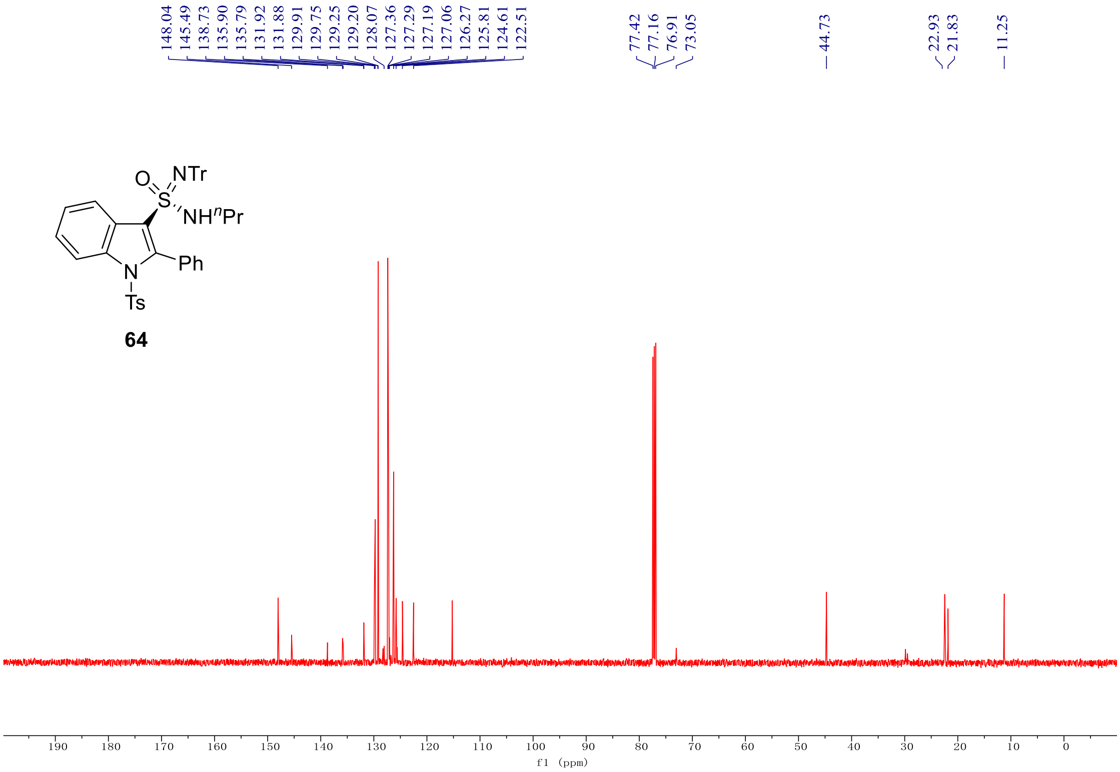
148.04
145.49
138.73
135.90
135.79
131.92
131.88
129.91
129.75
129.25
129.20
128.07
127.36
127.29
127.19
127.06
126.27
125.81
124.61
122.51
77.42
77.16
76.91
73.05
44.73
22.93
21.83
11.25
O
NTr
S
NH^{n}Pr
Ph
N
Ts
190 180 170 160 150 140 130 120 110 100 90 80 70 60 50 40 30 20 10 0
f1 (ppm)

64

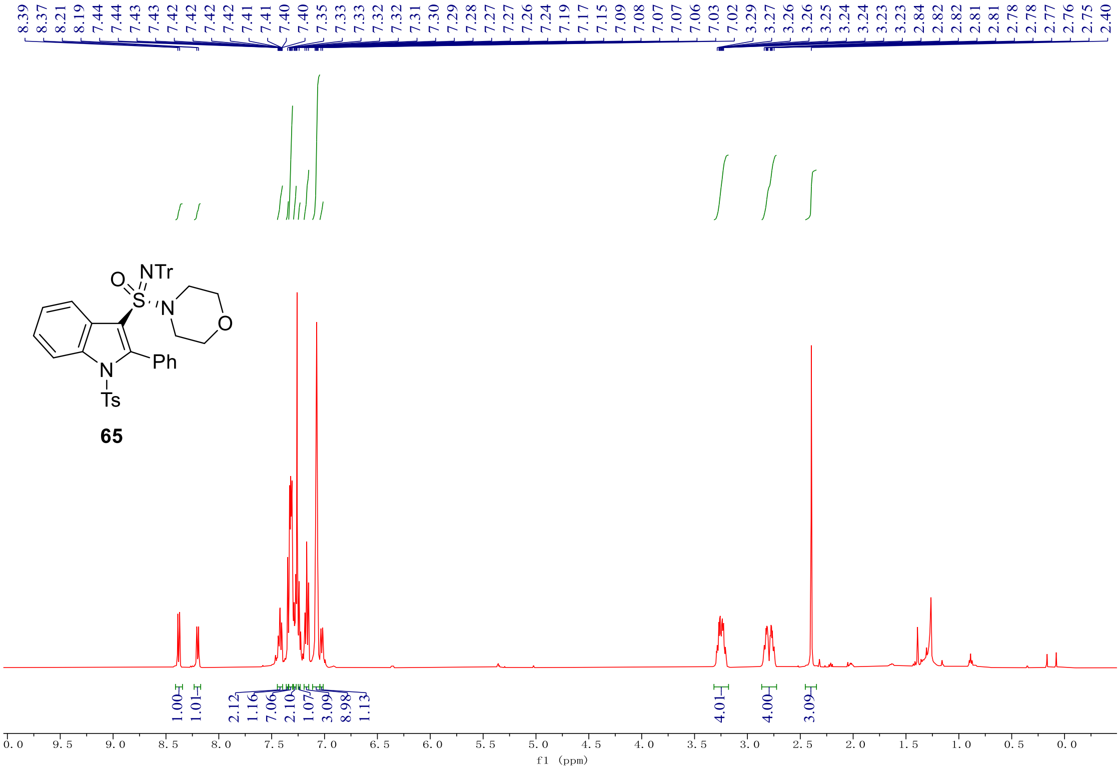
8.39
8.37
8.21
8.19
7.44
7.44
7.43
7.43
7.42
7.42
7.42
7.41
7.41
7.40
7.40
7.35
7.33
7.32
7.32
7.31
7.30
7.29
7.28
7.27
7.27
7.26
7.24
7.19
7.17
7.15
7.09
7.08
7.07
7.07
7.06
7.03
7.02
3.29
3.27
3.26
3.26
3.25
3.24
3.24
3.23
2.84
2.82
2.82
2.81
2.81
2.78
2.78
2.77
2.76
2.75
2.40
O
NTr
S
N
O
Ph
N
Ts
1.00
1.01
2.12
1.16
7.06
2.10
1.07
3.09
8.98
1.13
4.01
4.00
3.09
10.0 9.5 9.0 8.5 8.0 7.5 7.0 6.5 6.0 5.5 5.0 4.5 4.0 3.5 3.0 2.5 2.0 1.5 1.0 0.5 0.0
f1 (ppm)

65

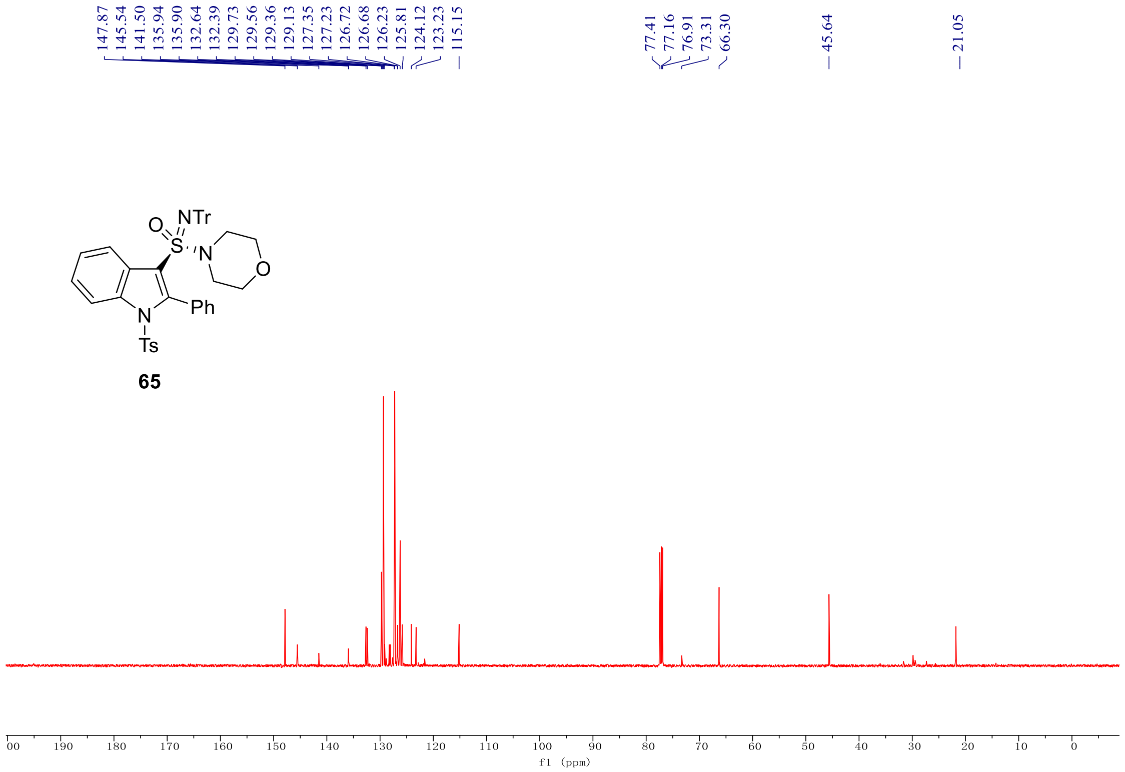
147.87
145.54
141.50
135.94
135.90
132.64
132.39
129.73
129.56
129.36
129.13
127.35
127.23
126.72
126.68
126.23
125.81
124.12
123.23
115.15
77.41
77.16
76.91
73.31
66.30
45.64
21.05
O
NTr
S
N
O
N
Ts
Ph
65
f1 (ppm)

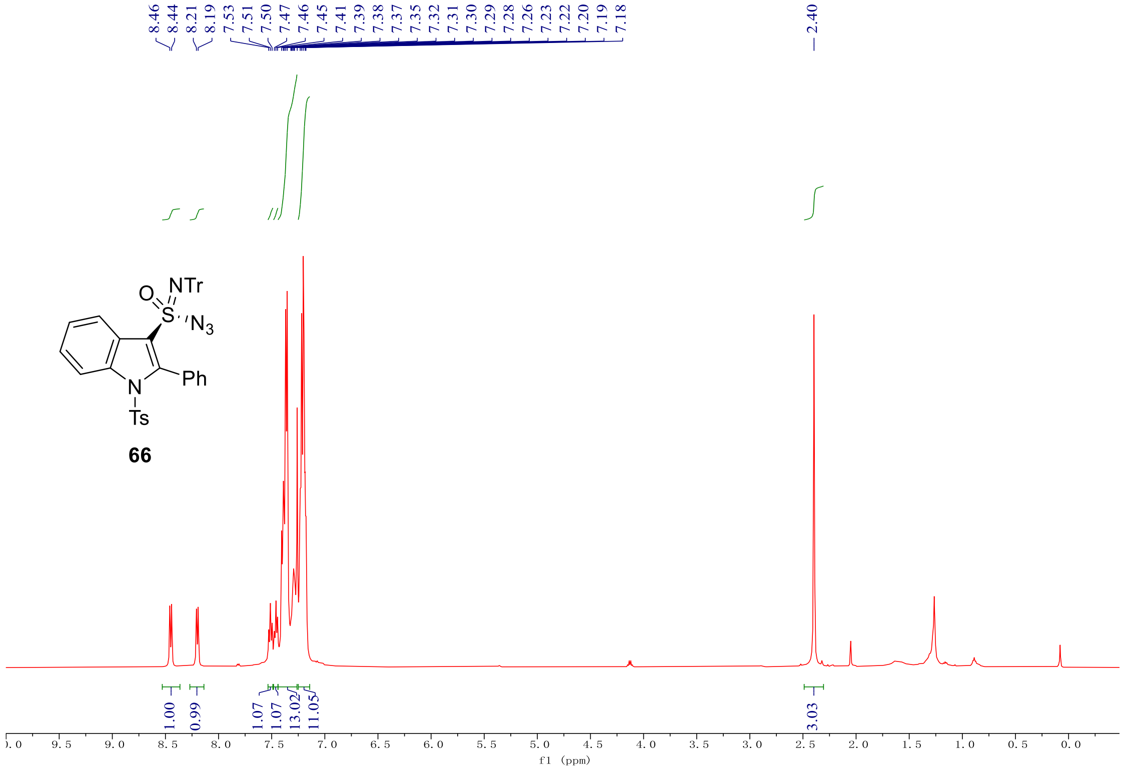
8.46
8.44
8.21
8.19
7.53
7.51
7.50
7.47
7.46
7.45
7.41
7.39
7.38
7.37
7.35
7.32
7.31
7.30
7.29
7.28
7.26
7.23
7.22
7.20
7.19
7.18
2.40
O
NTr
S
N_3
N
Ts
Ph
66
1.00
0.99
1.07
1.07
13.02
11.05
3.03
f1 (ppm)

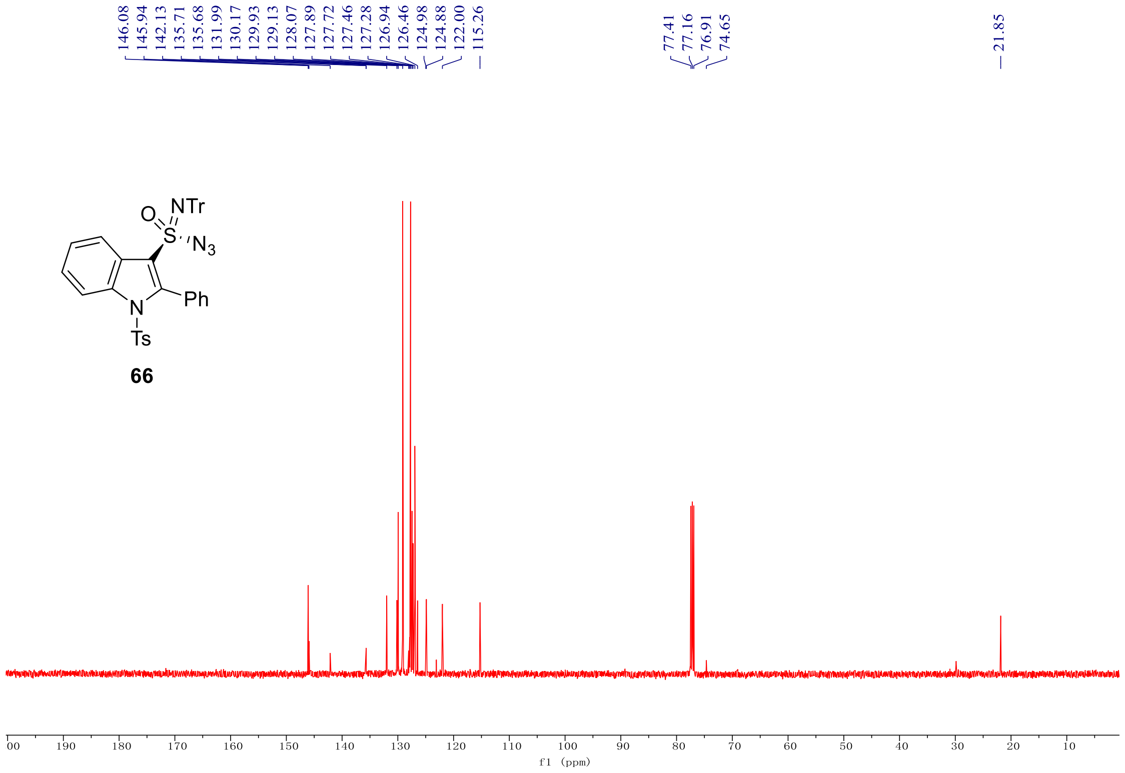
146.08
145.94
142.13
135.71
135.68
131.99
130.17
129.93
129.13
128.07
127.89
127.72
127.46
127.28
126.94
126.46
124.98
124.88
122.00
115.26
77.41
77.16
76.91
74.65
21.85
NTr
O
S
N3
N
Ph
Ts
66
f1 (ppm)

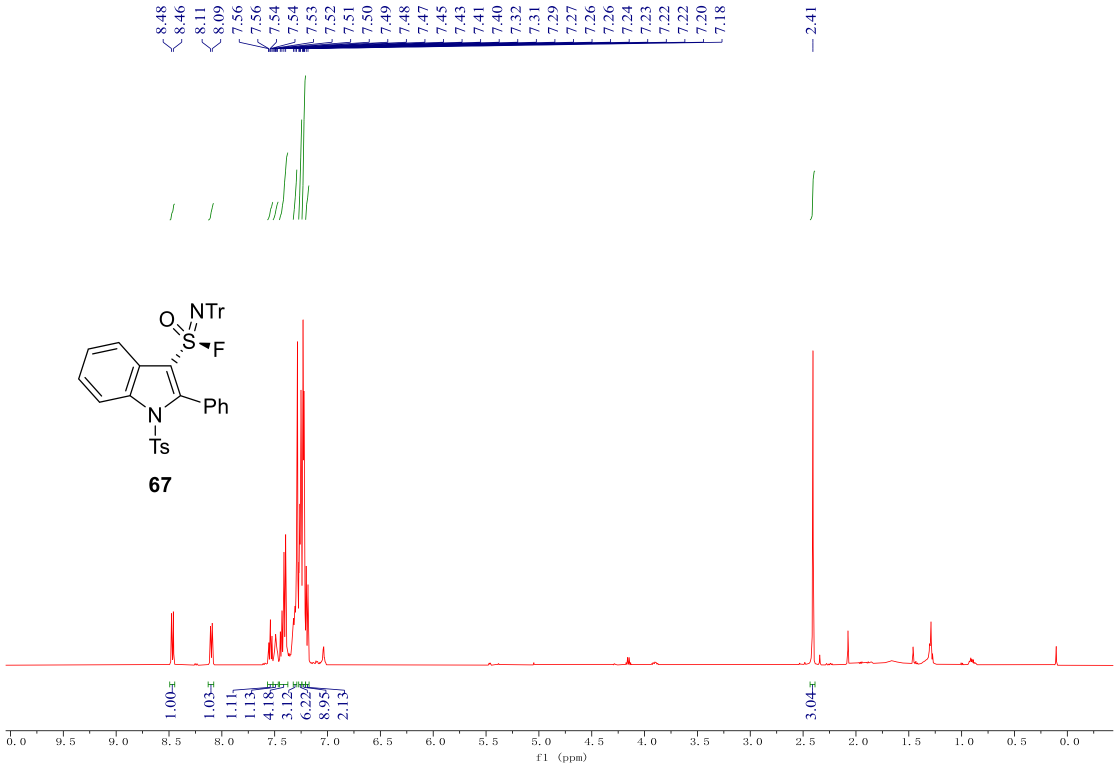
8.48
8.46
8.11
8.09
7.56
7.56
7.54
7.54
7.53
7.52
7.51
7.50
7.49
7.48
7.47
7.45
7.43
7.41
7.40
7.32
7.31
7.29
7.27
7.26
7.24
7.23
7.22
7.22
7.20
7.18
2.41
NTr
O
S
F
N
Ph
Ts
67
1.00
1.03
1.11
1.13
4.18
3.12
6.22
8.95
2.13
3.04
f1 (ppm)

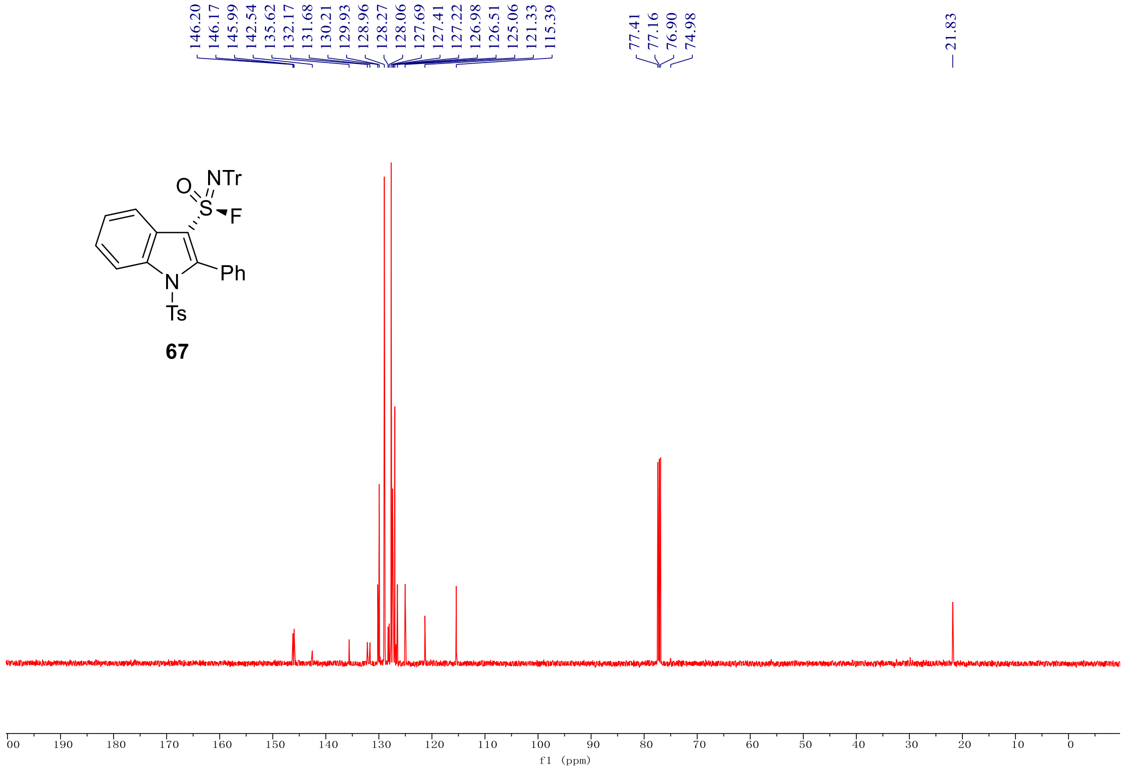
146.20
146.17
145.99
142.54
135.62
132.17
131.68
130.21
129.93
128.96
128.27
128.06
127.69
127.41
127.22
126.98
126.51
125.06
121.33
115.39
77.41
77.16
76.90
74.98
21.83
NTr
O
S
F
N
Ph
Ts
67
f1 (ppm)

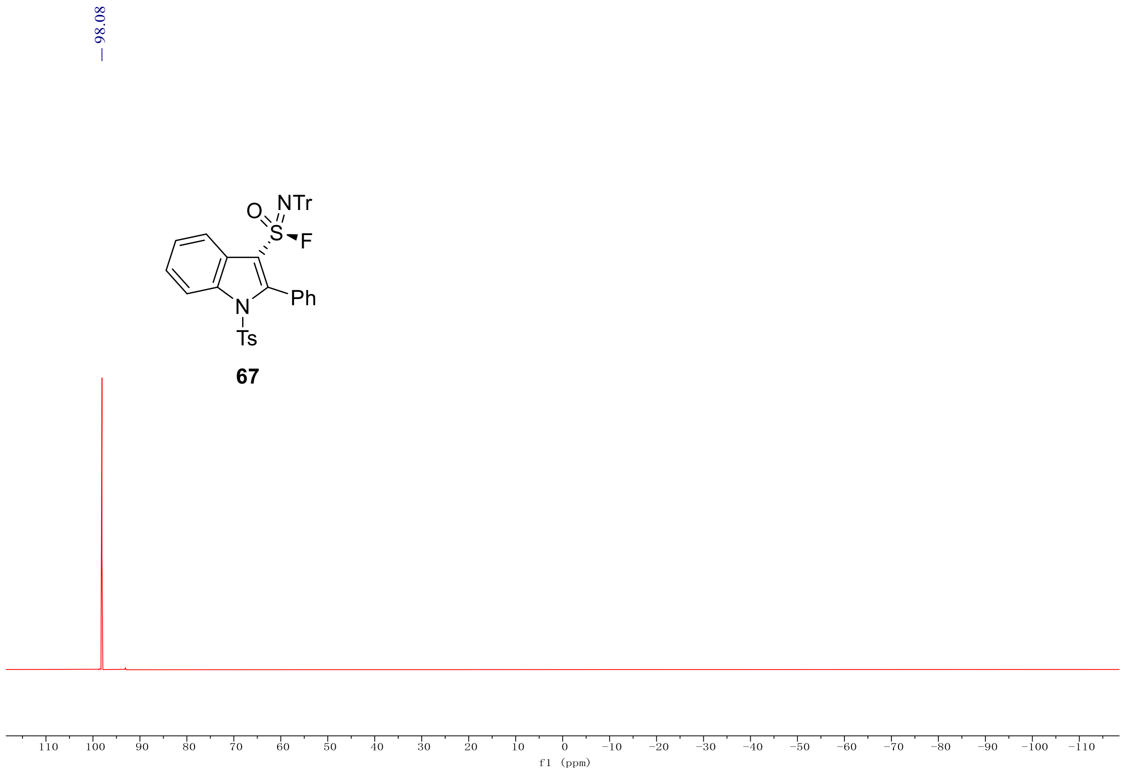
98.08
NTr
O
S
F
N
Ph
Ts
67
f1 (ppm)

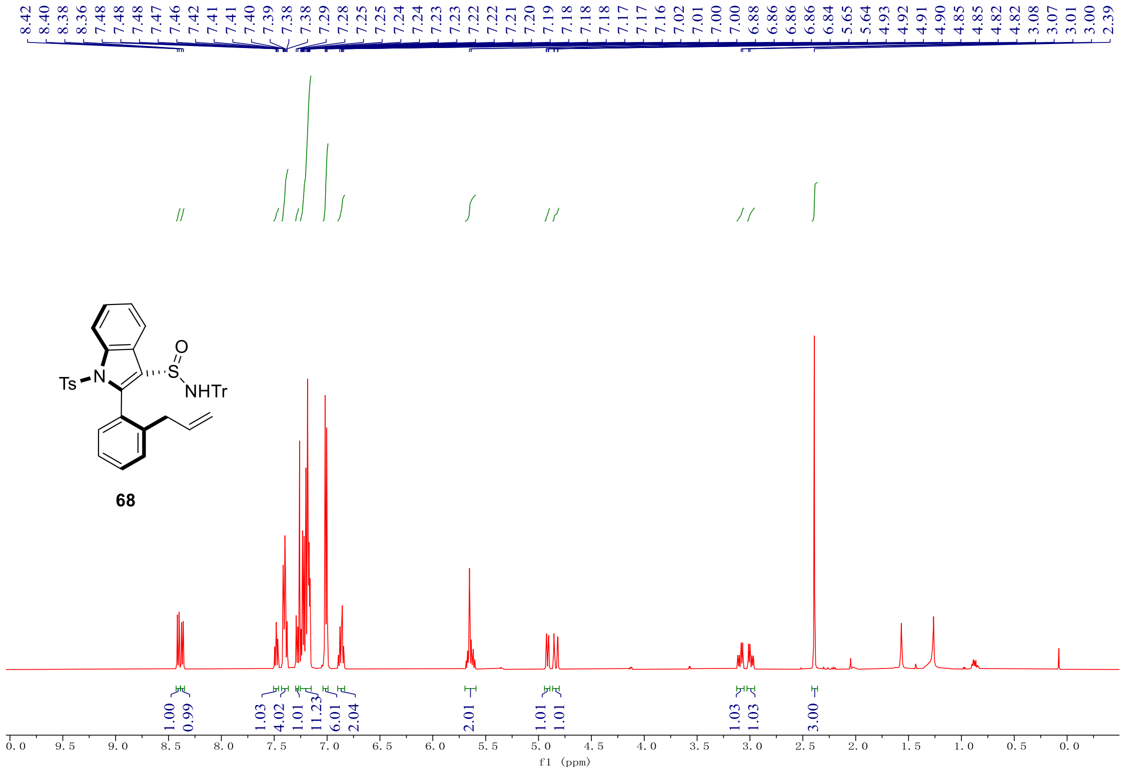
8.42 8.40 8.38 8.36 7.48 7.48 7.48 7.47 7.46 7.42 7.41 7.41 7.40 7.39 7.38 7.38 7.29 7.28 7.25 7.25 7.24 7.24 7.23 7.23 7.22 7.22 7.21 7.20 7.19 7.18 7.18 7.18 7.17 7.17 7.16 7.02 7.01 7.00 7.00 6.88 6.86 6.86 6.86 6.84 5.65 5.64 4.93 4.92 4.91 4.90 4.85 4.85 4.82 4.82 3.08 3.07 3.01 3.00 2.39
Ts
N
S
O
NHTr
68
1.00 0.99 1.03 4.02 1.01 11.23 6.01 2.04 2.01 1.01 1.01 1.03 1.03 3.00
f1 (ppm)

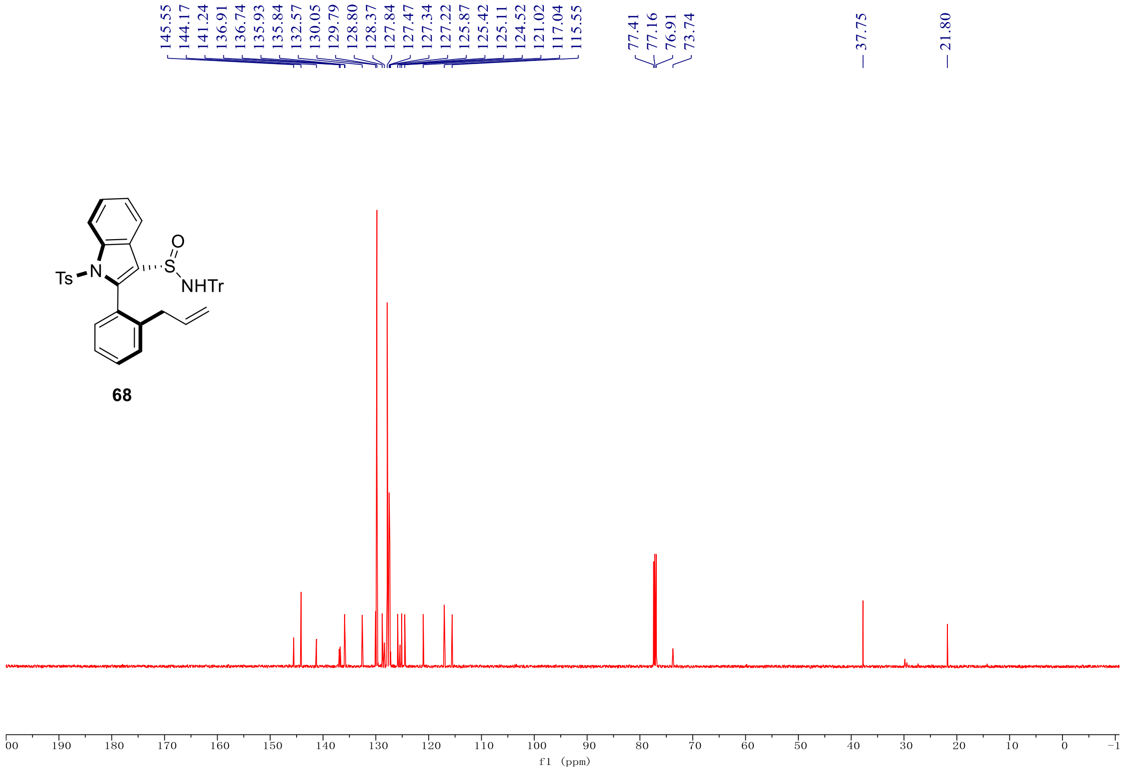
145.55 144.17 141.24 136.91 136.74 135.93 135.84 132.57 130.05 129.79 128.80 128.37 127.84 127.47 127.34 127.22 125.87 125.42 125.11 124.52 121.02 117.04 115.55
77.41 77.16 76.91 73.74
37.75
21.80
Ts
N
S
O
NHTr
68
f1 (ppm)

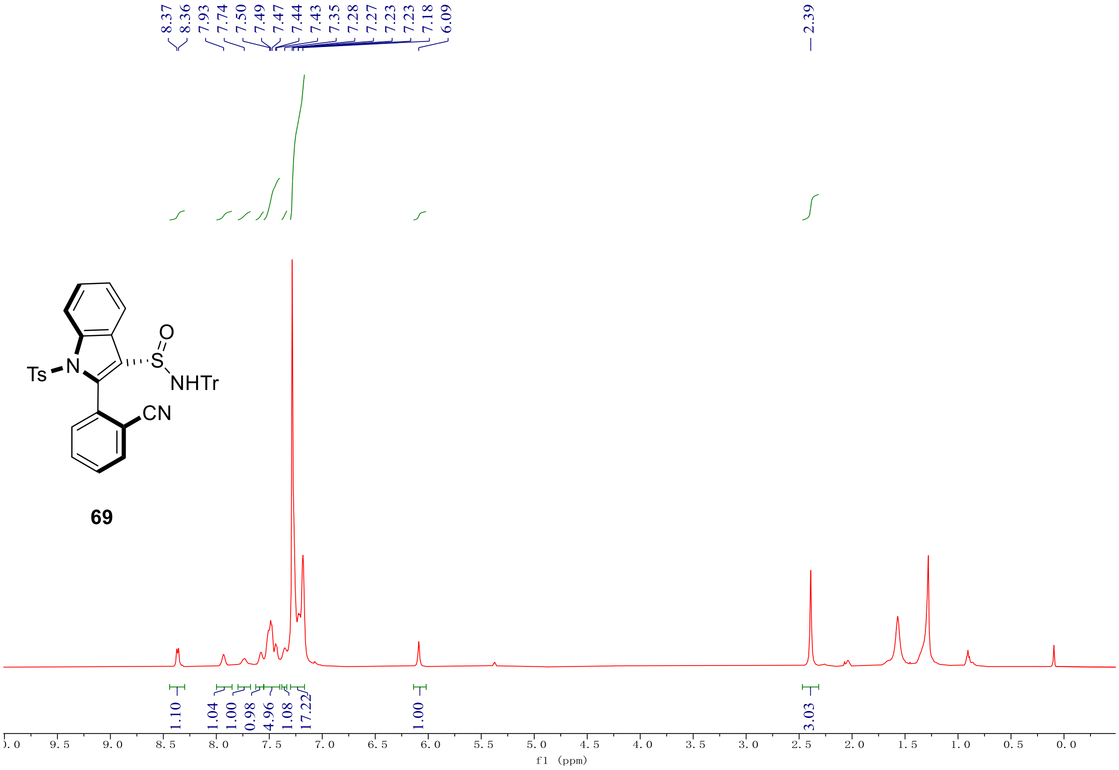
8.37
8.36
7.93
7.74
7.50
7.49
7.47
7.44
7.43
7.35
7.28
7.27
7.23
7.23
7.18
6.09
2.39
Ts
N
O
S
NHTr
CN
69
1.10
1.04
1.00
0.98
4.96
1.08
17.22
1.00
3.03
f1 (ppm)

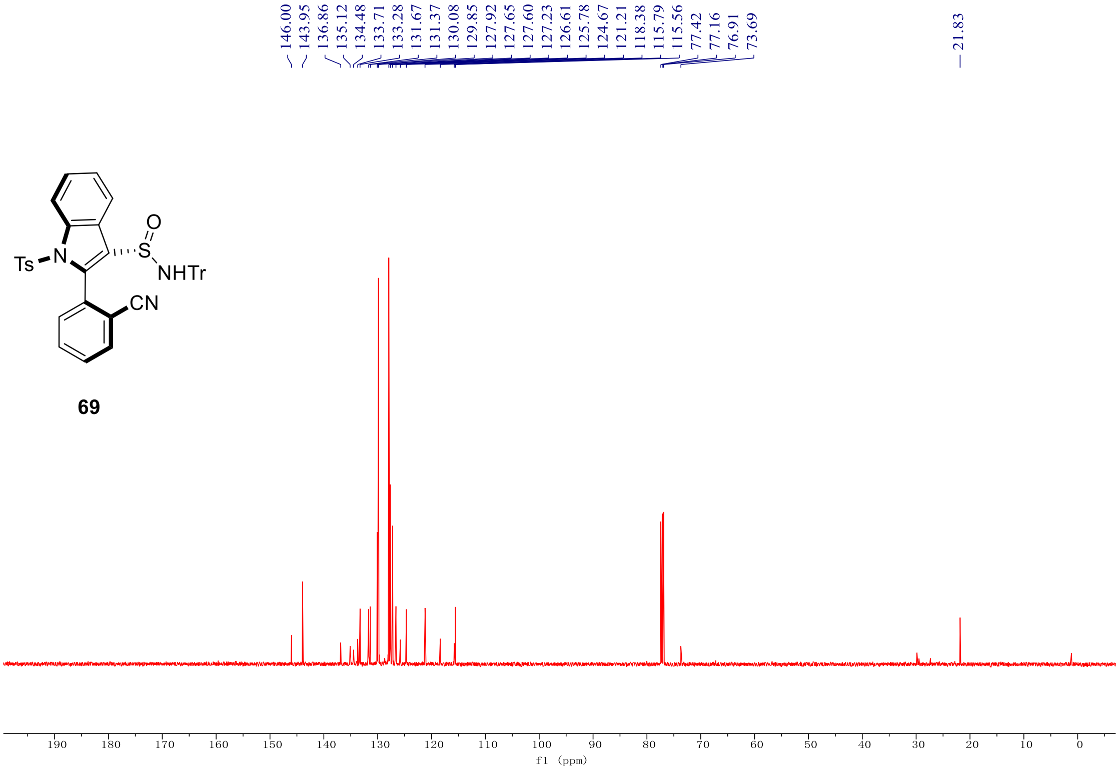
146.00
143.95
136.86
135.12
134.48
133.71
133.28
131.67
131.37
130.08
129.85
127.92
127.65
127.60
127.23
126.61
125.78
124.67
121.21
118.38
115.79
115.56
77.42
77.16
76.91
73.69
21.83
Ts
N
O
S
NHTr
CN
69
f1 (ppm)

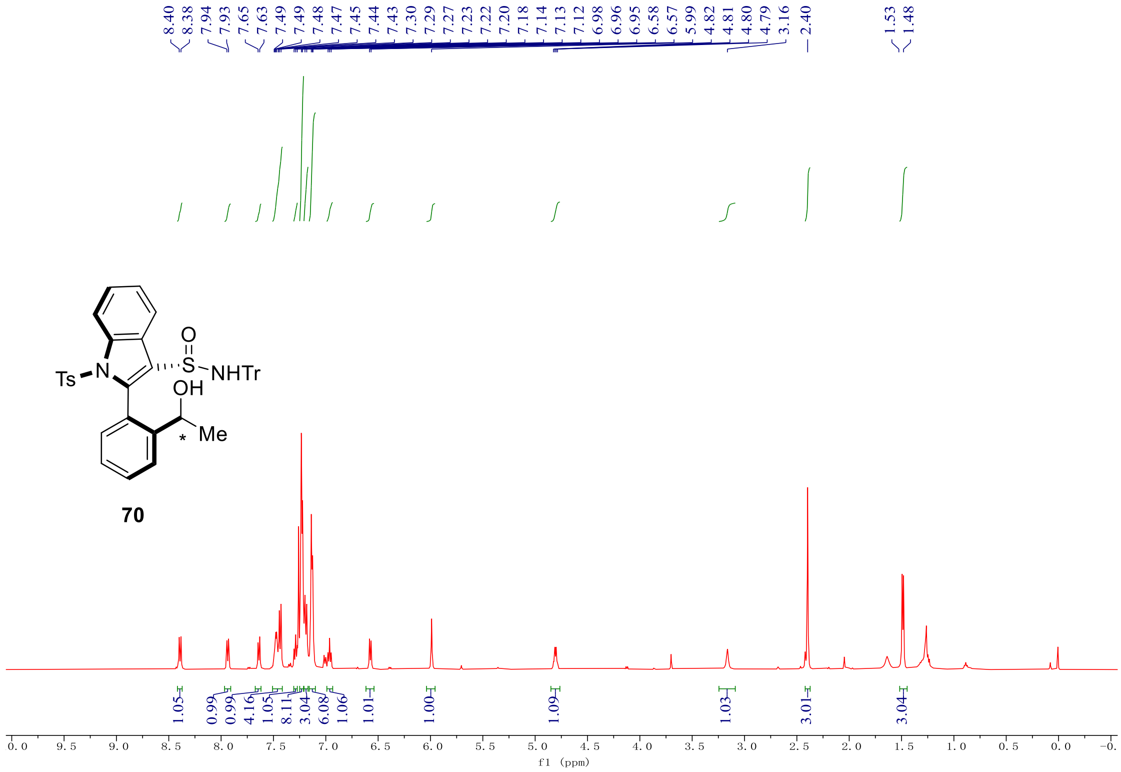
8.40 8.38 7.94 7.93 7.65 7.63 7.49 7.49 7.48 7.47 7.45 7.44 7.43 7.30 7.29 7.27 7.23 7.22 7.20 7.18 7.14 7.13 7.12 6.98 6.96 6.95 6.58 6.57 5.99 4.82 4.81 4.80 4.79 3.16 2.40 1.53 1.48
Ts
N
O
S
NHTr
OH
Me
70
1.05 0.99 0.99 4.16 1.05 8.11 3.04 6.08 1.06 1.01 1.00 1.09 1.03 3.01 3.04
f1 (ppm)

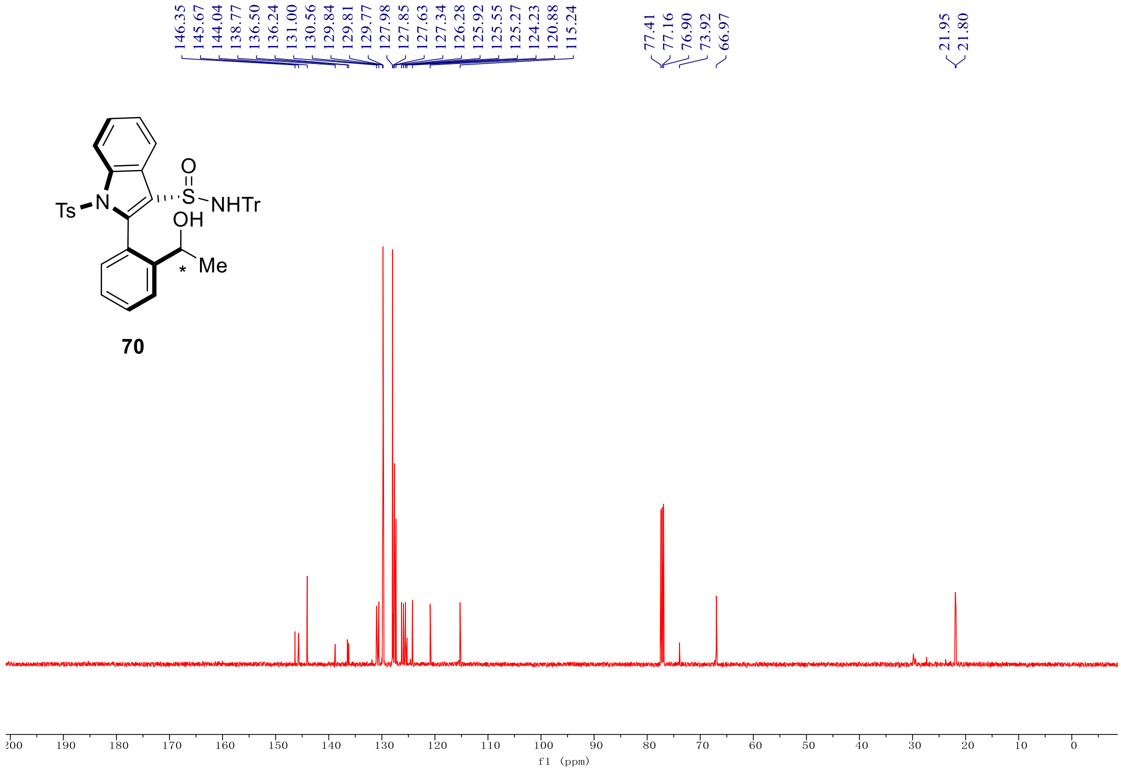
146.35 145.67 144.04 138.77 136.50 136.24 131.00 130.56 129.84 129.81 129.77 127.98 127.85 127.63 127.34 126.28 125.92 125.55 125.27 124.23 120.88 115.24 77.41 77.16 76.90 73.92 66.97 21.95 21.80
Ts
N
O
S
NHTr
OH
Me
70
f1 (ppm)

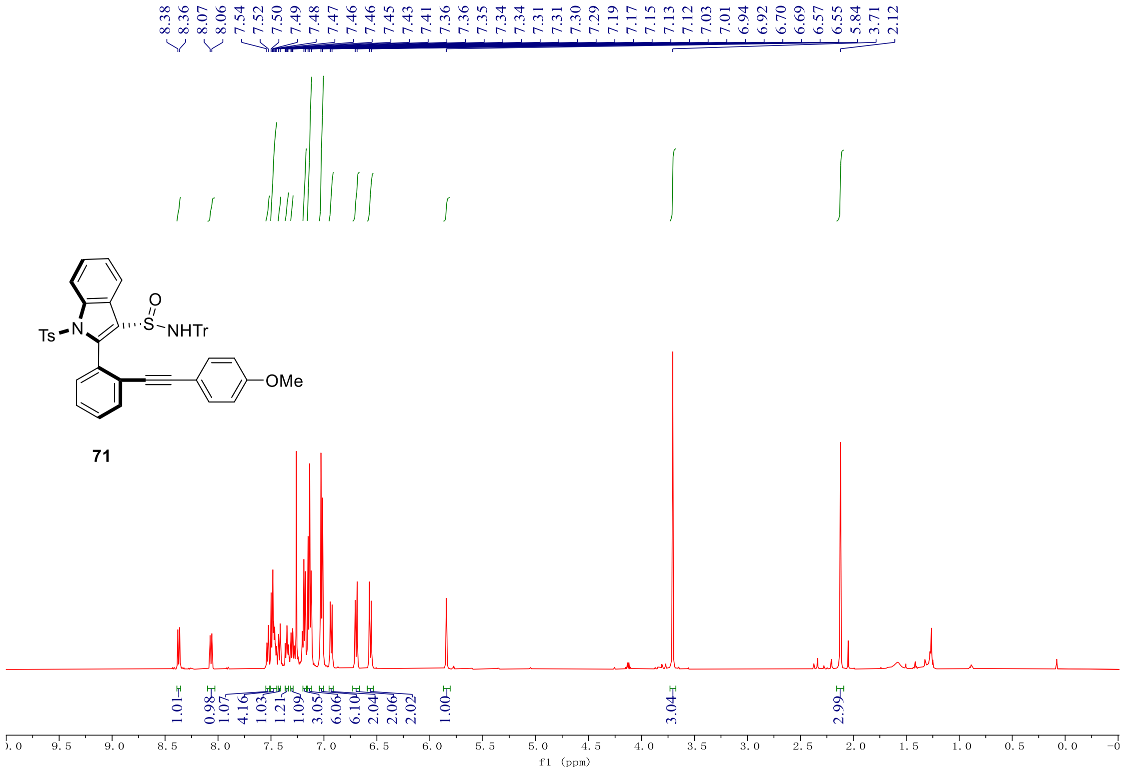
8.38
8.36
8.07
8.06
7.54
7.52
7.50
7.49
7.48
7.47
7.46
7.46
7.45
7.43
7.41
7.36
7.36
7.35
7.34
7.34
7.31
7.31
7.30
7.29
7.19
7.17
7.15
7.13
7.12
7.03
7.01
6.94
6.92
6.70
6.69
6.57
6.55
5.84
3.71
2.12
Ts
N
S
O
NHTr
OMe
71
1.01
0.98
1.07
4.16
1.03
1.21
1.09
3.05
6.06
6.10
2.04
2.06
2.02
1.00
3.04
2.99
f1 (ppm)

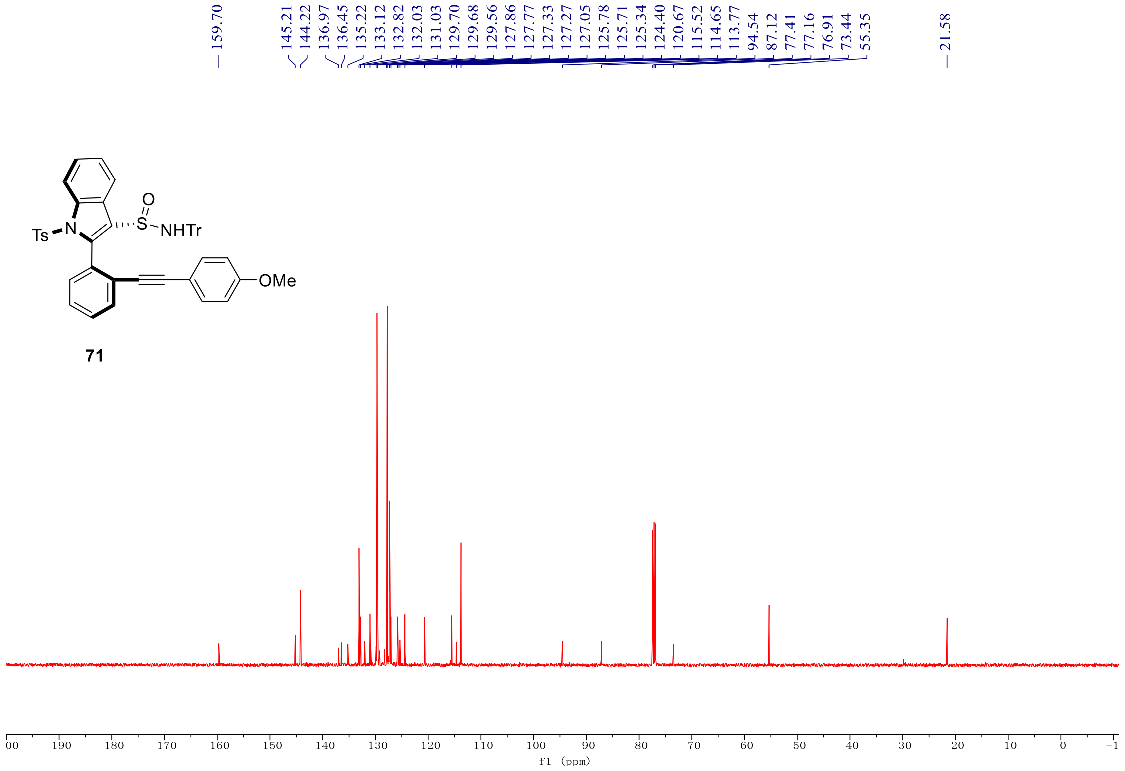
159.70
145.21
144.22
136.97
136.45
135.22
133.12
132.82
132.03
131.03
129.70
129.68
129.56
127.86
127.77
127.33
127.27
127.05
125.78
125.71
125.34
124.40
120.67
115.52
114.65
113.77
94.54
87.12
77.41
77.16
76.91
73.44
55.35
21.58
Ts
N
S
O
NHTr
OMe
71
f1 (ppm)

8.4 Mechanistic Studies

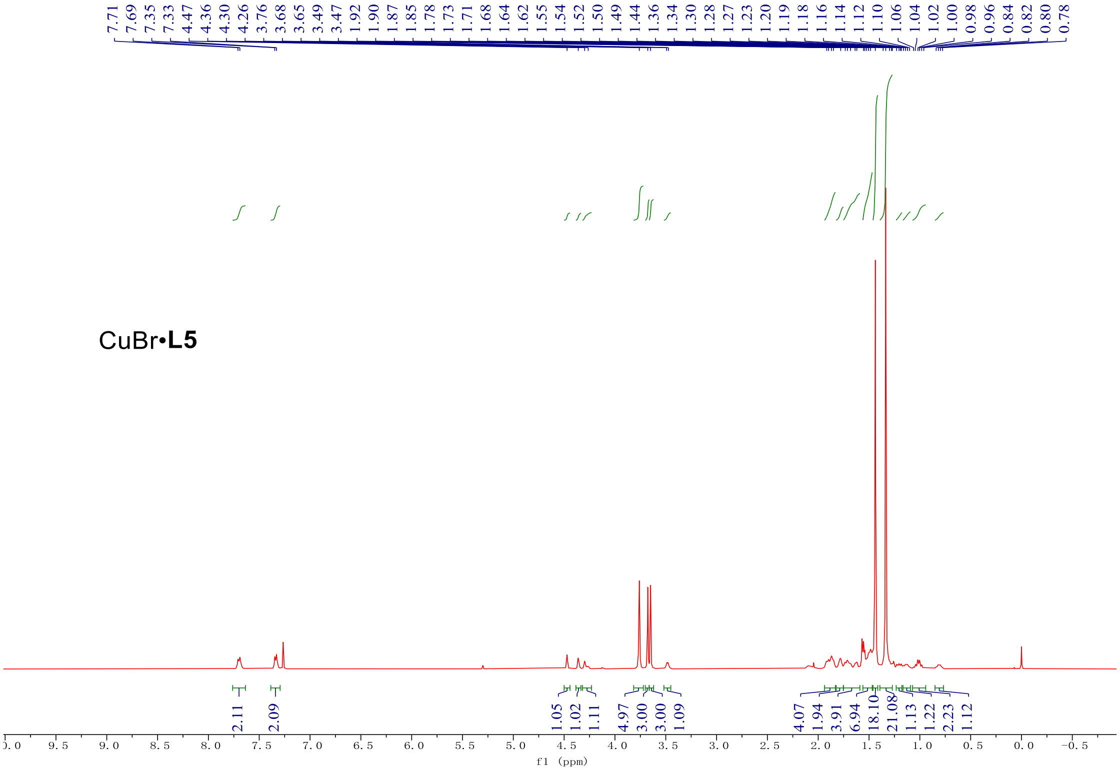

CuBr•**L5**

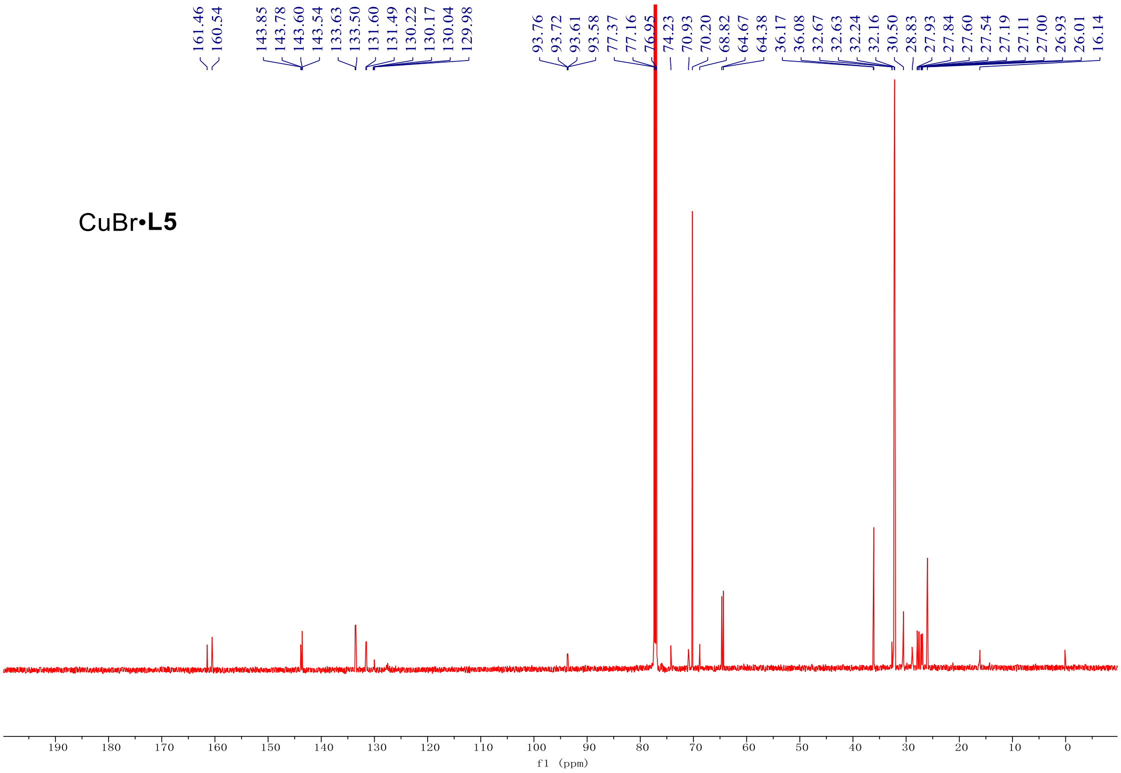

CuBr•**L5**

CuBr•**L5**

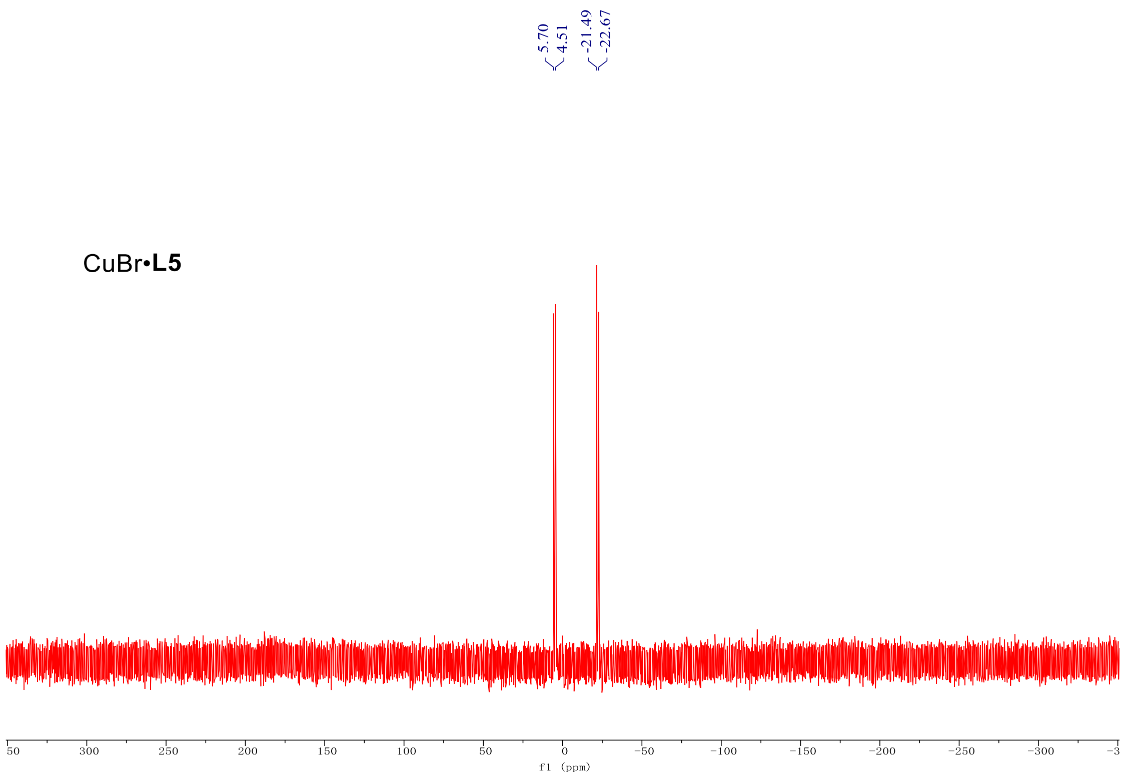

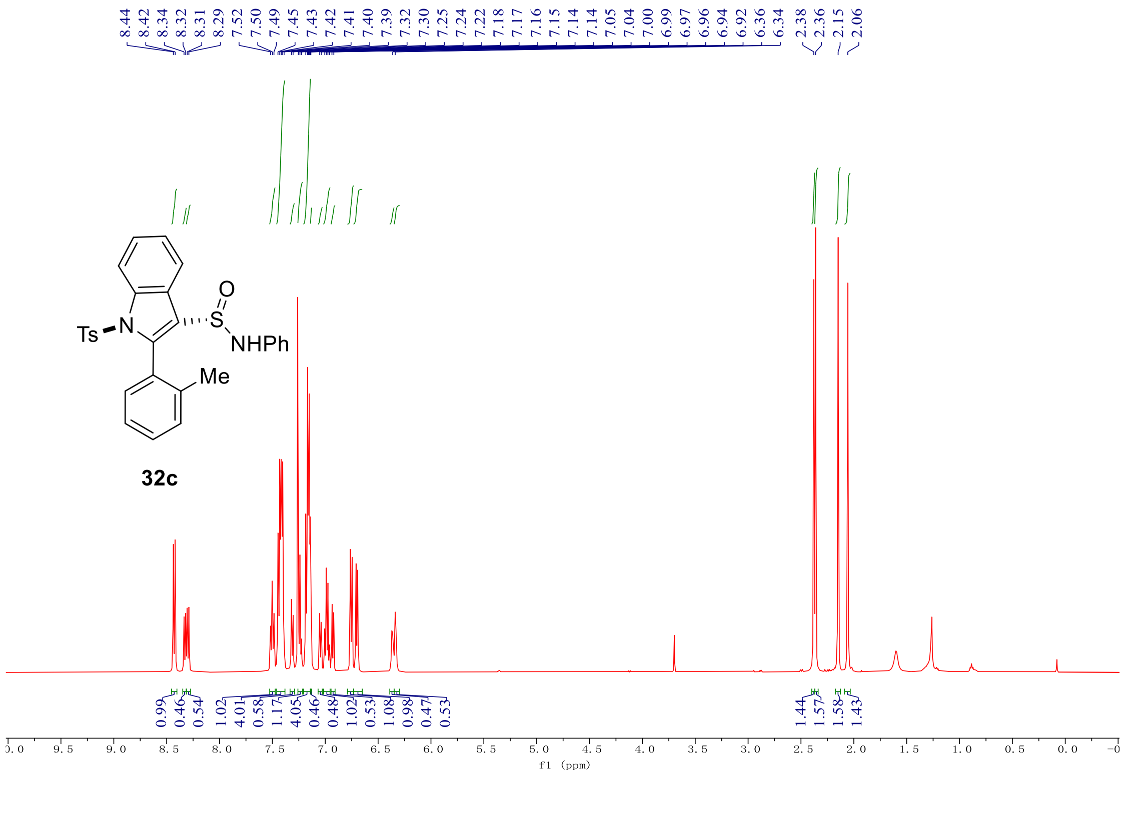

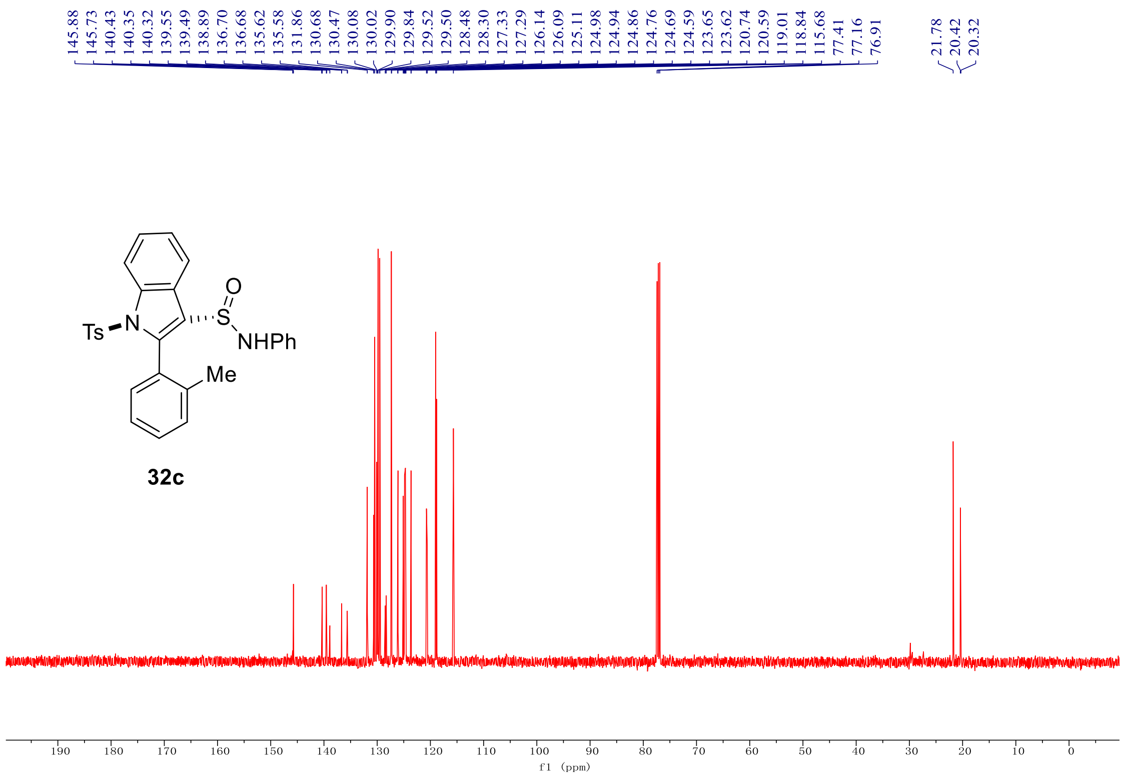

32c

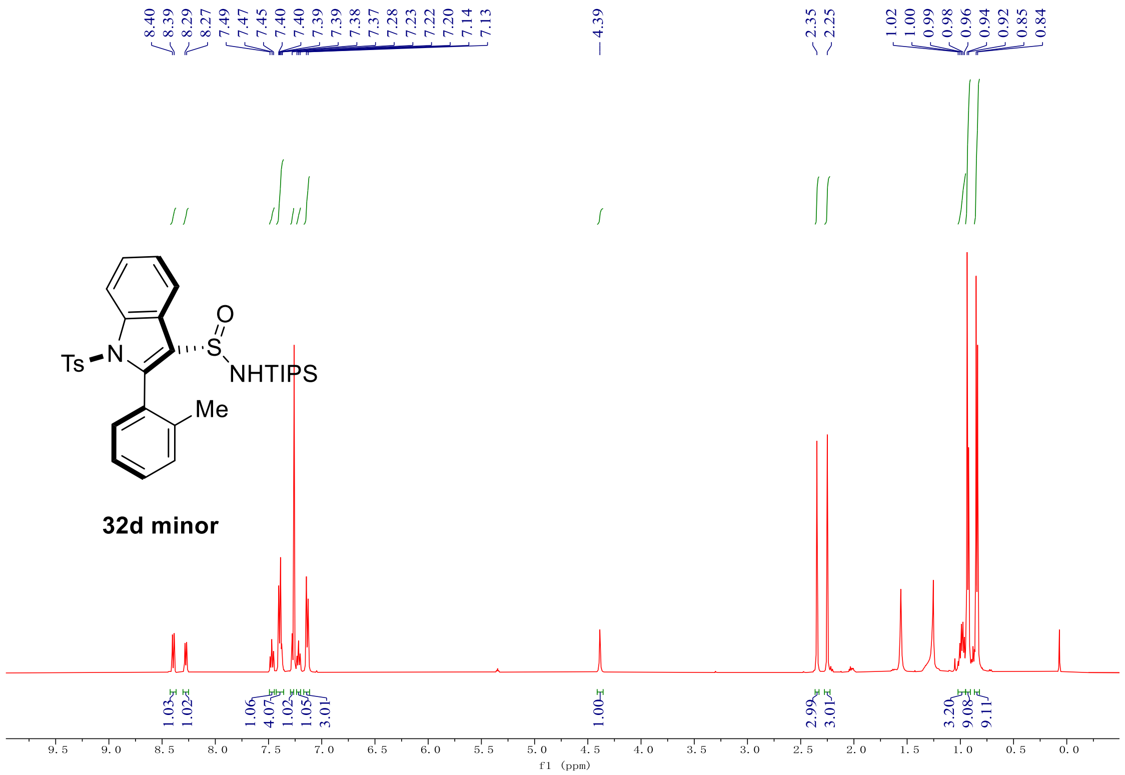

32d minor

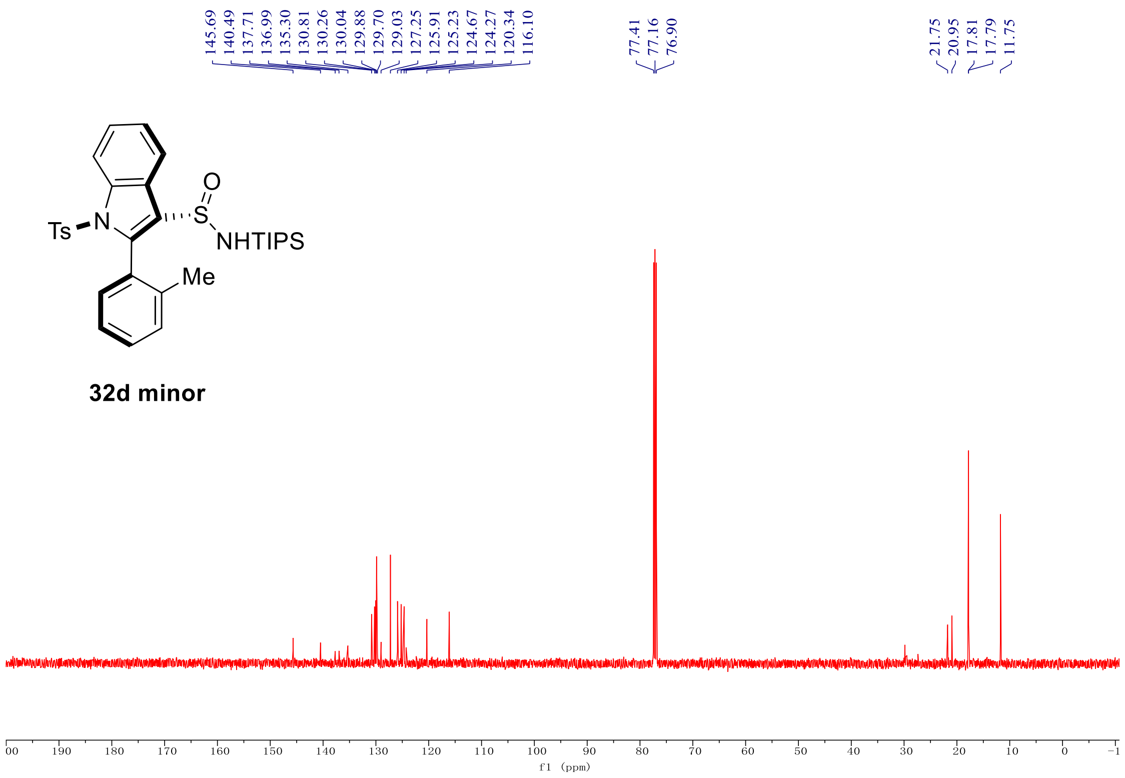

32d minor

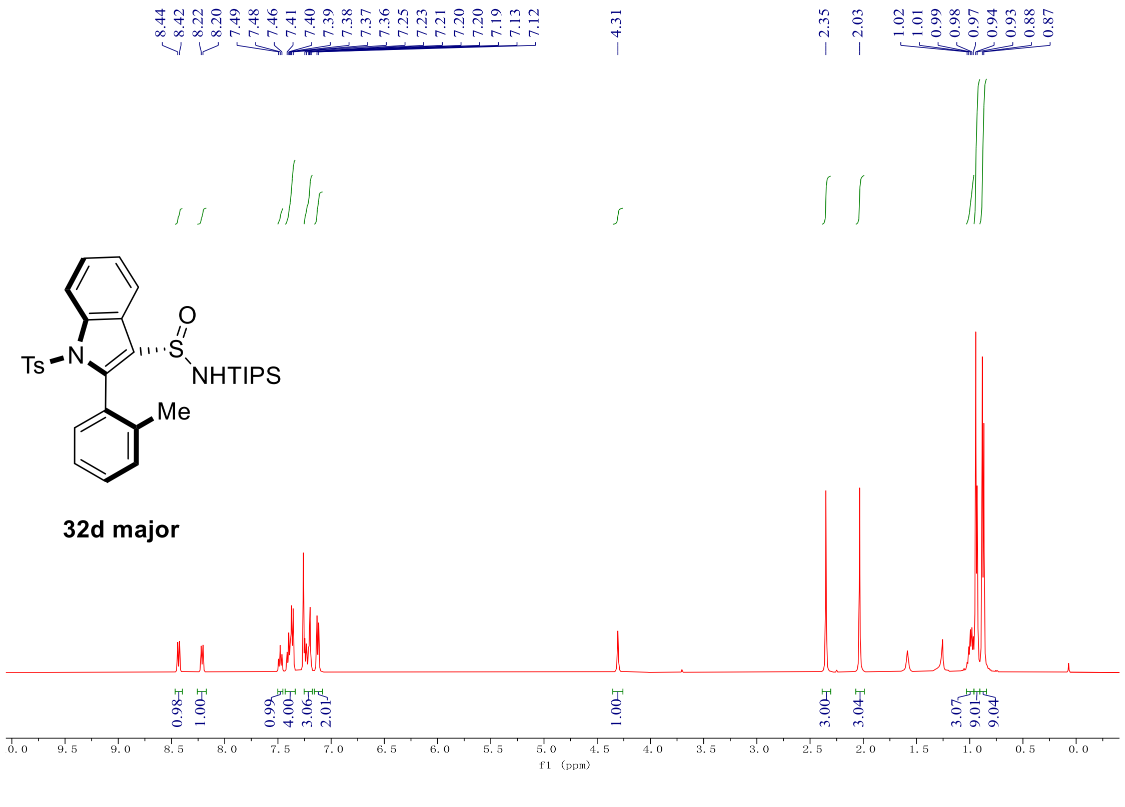

32d major

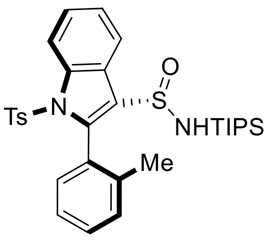

32d major

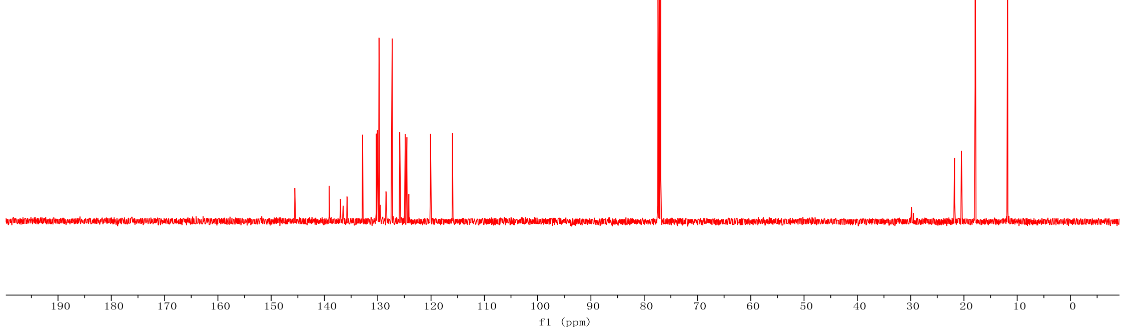
145.57
139.11
137.00
136.51
135.77
132.83
130.26
130.06
129.77
129.56
128.46
127.30
125.86
124.84
124.53
124.16
120.10
115.97
77.41
77.16
76.91
21.76
20.47
17.90
17.88
11.80
190
180
170
160
150
140
130
120
110
100
90
80
70
60
50
40
30
20
10
0
f1 (ppm)

9 Copies of HPLC Spectras

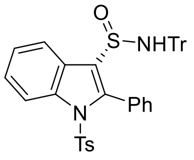

1b

Chromatogram

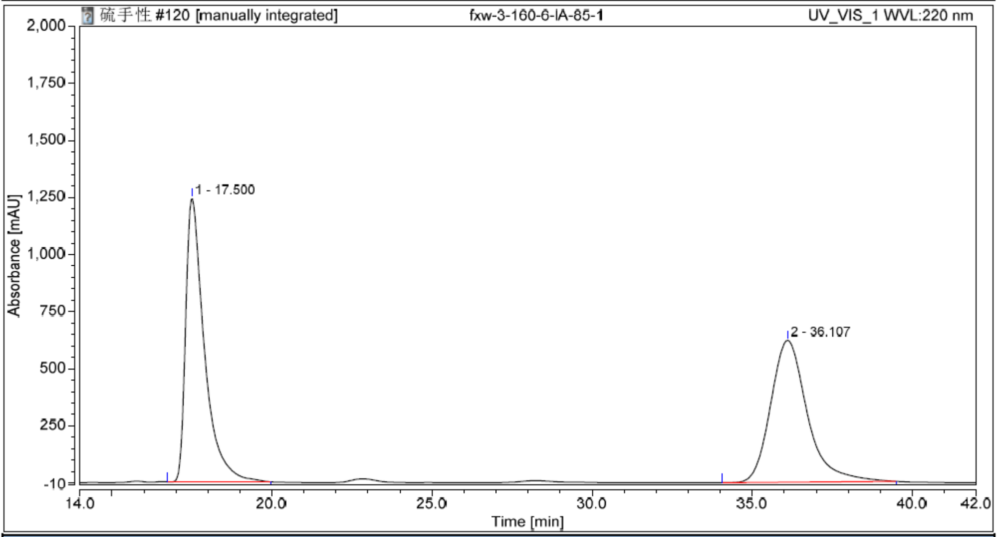

Integration Results

No.	Peak Name	Retention Time<br>min	Area<br>mAU*min	Height<br>mAU	Relative Area<br>%	Relative Height<br>%	Amount<br>n.a.
1		17.500	838.592	1243.518	50.45	66.68	n.a.
2		36.107	823.753	621.381	49.55	33.32	n.a.
**Total:**			**1662.345**	**1864.899**	**100.00**	**100.00**	

Chromatogram

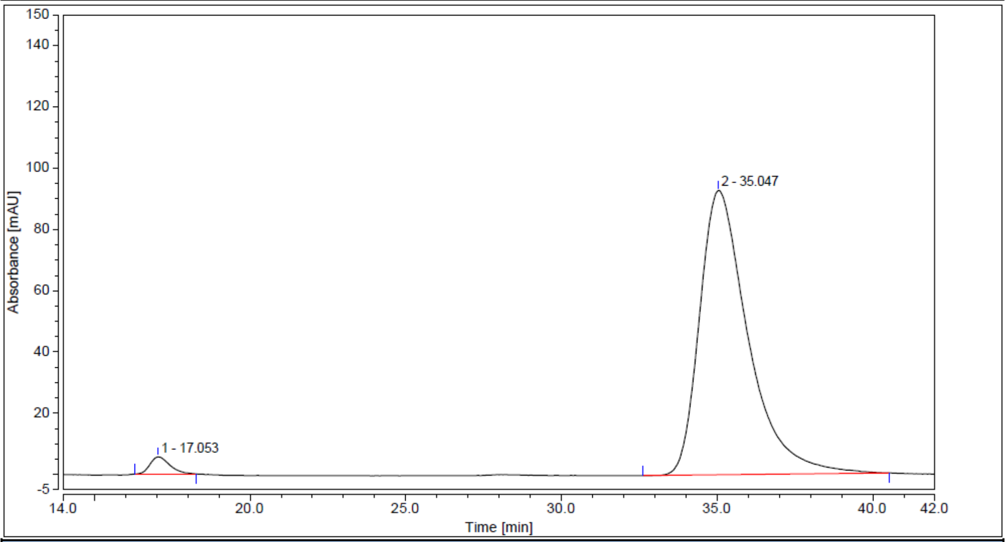

Integration Results

No.	Peak Name	Retention Time<br>min	Area<br>mAU*min	Height<br>mAU	Relative Area<br>%	Relative Height<br>%	Amount<br>n.a.
1		17.053	4.103	5.582	2.39	5.67	n.a.
2		35.047	167.598	92.872	97.61	94.33	n.a.
**Total:**			**171.700**	**98.454**	**100.00**	**100.00**	

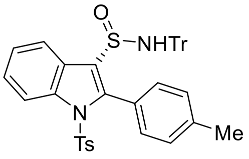

2b

Chromatogram

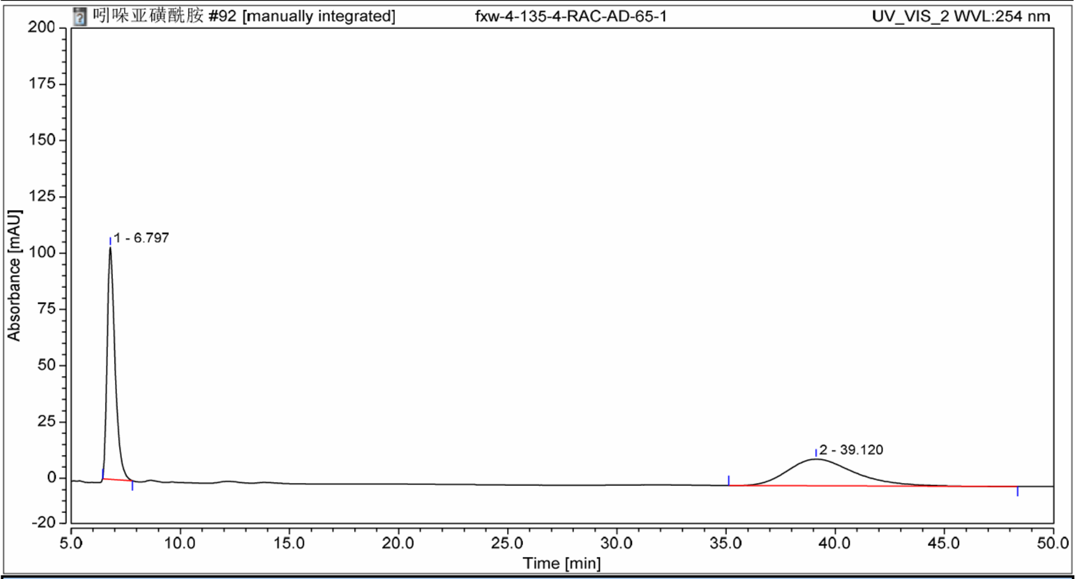

Integration Results

No.	Peak Name	Retention Time min	Area mAU*min	Height mAU	Relative Area %	Relative Height %	Amount n.a.
1		6.797	43.209	103.085	50.68	89.68	n.a.
2		39.120	42.056	11.863	49.32	10.32	n.a.
**Total:**			**85.264**	**114.948**	**100.00**	**100.00**	

Chromatogram

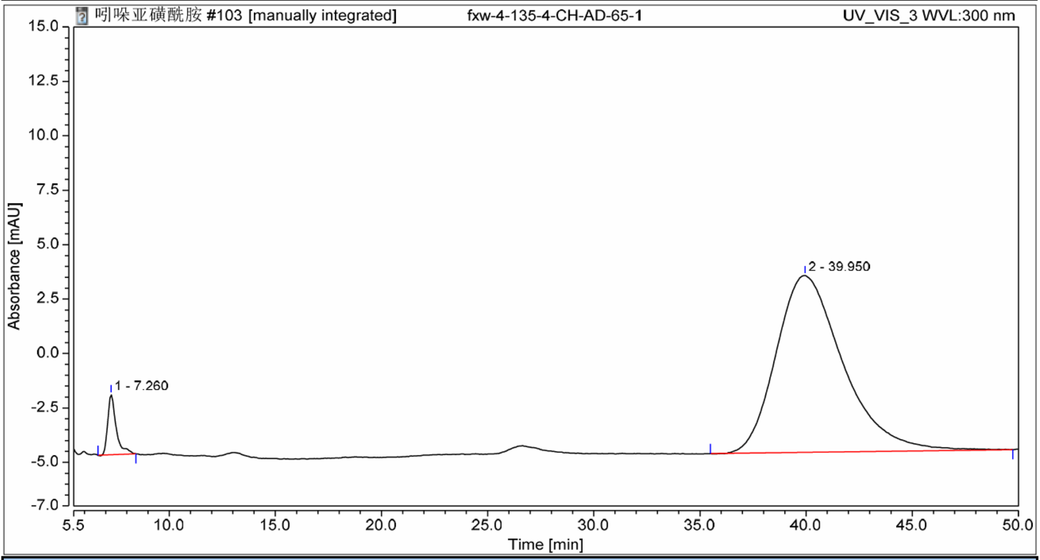

Integration Results

No.	Peak Name	Retention Time min	Area mAU*min	Height mAU	Relative Area %	Relative Height %	Amount n.a.
1		7.260	1.268	2.759	4.10	25.35	n.a.
2		39.950	29.671	8.124	95.90	74.65	n.a.
**Total:**			**30.939**	**10.884**	**100.00**	**100.00**	

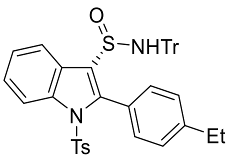

3b

Chromatogram

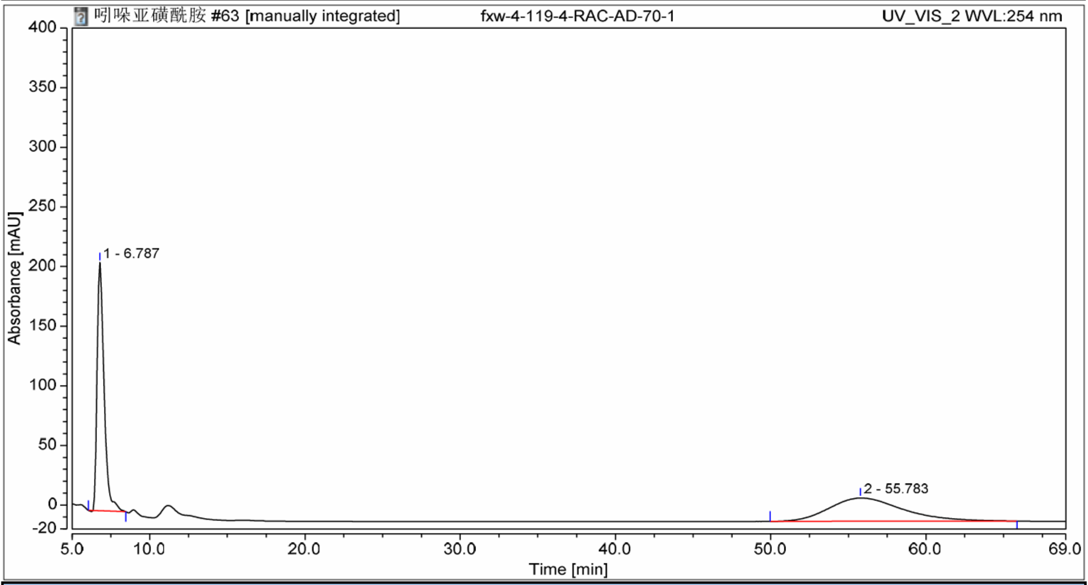

Integration Results

No.	Peak Name	Retention Time min	Area mAU*min	Height mAU	Relative Area %	Relative Height %	Amount n.a.
1		6.787	107.030	208.108	49.98	91.45	n.a.
2		55.783	107.116	19.453	50.02	8.55	n.a.
**Total:**			**214.146**	**227.561**	**100.00**	**100.00**	

Chromatogram

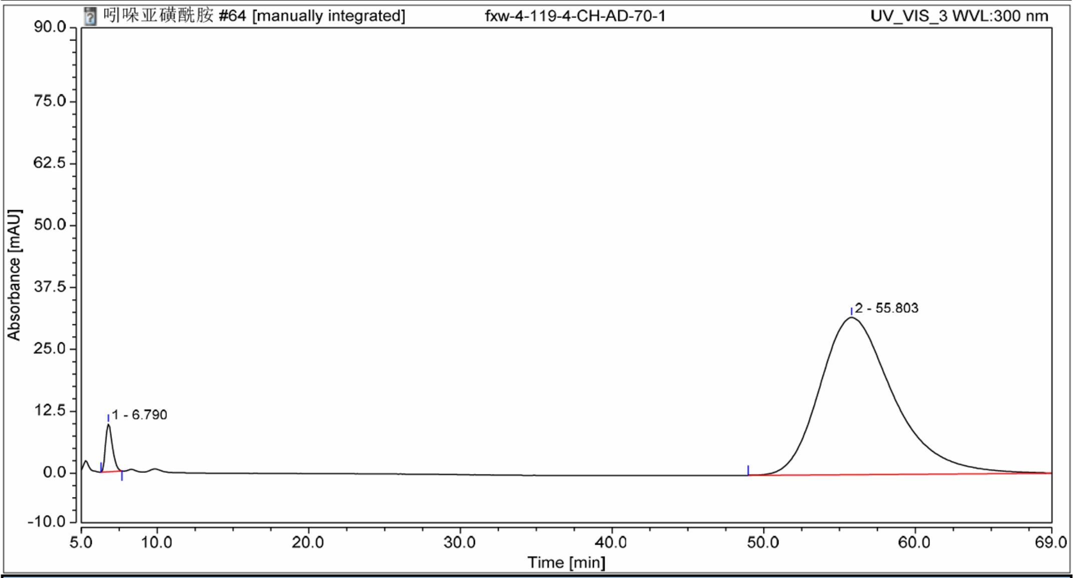

Integration Results

No.	Peak Name	Retention Time min	Area mAU*min	Height mAU	Relative Area %	Relative Height %	Amount n.a.
1		6.790	5.043	9.618	2.74	23.24	n.a.
2		55.803	179.042	31.771	97.26	76.76	n.a.
**Total:**			**184.085**	**41.389**	**100.00**	**100.00**	

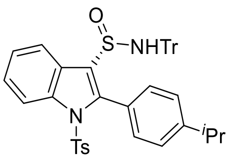

4b

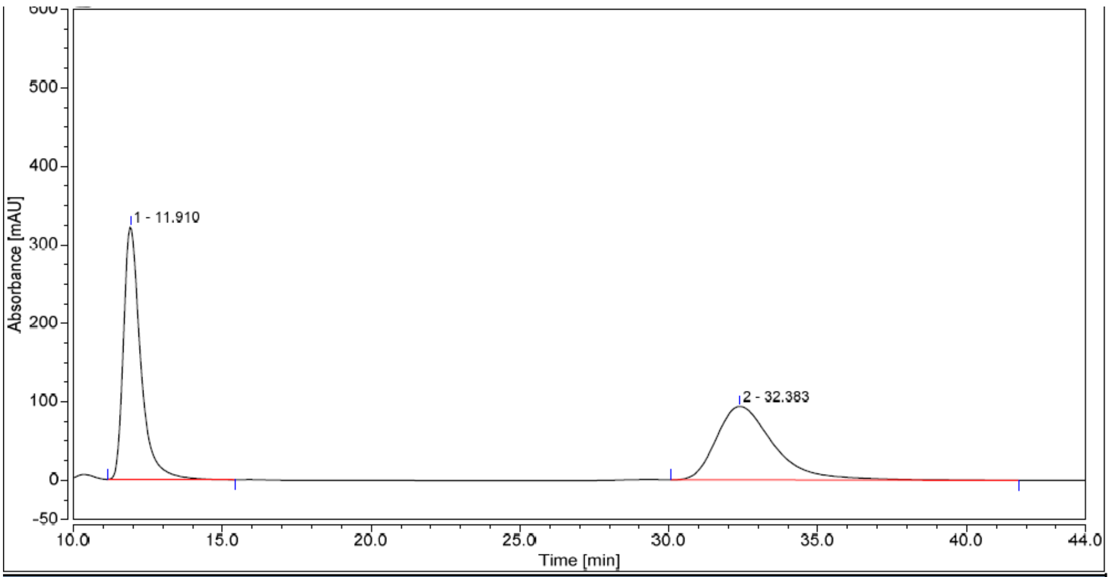

Integration Results

No.	Peak Name	Retention Time min	Area mAU*min	Height mAU	Relative Area %	Relative Height %	Amount n.a.
1		11.910	215.013	321.795	50.79	77.47	n.a.
2		32.383	208.330	93.588	49.21	22.53	n.a.
**Total:**			**423.343**	**415.383**	**100.00**	**100.00**	

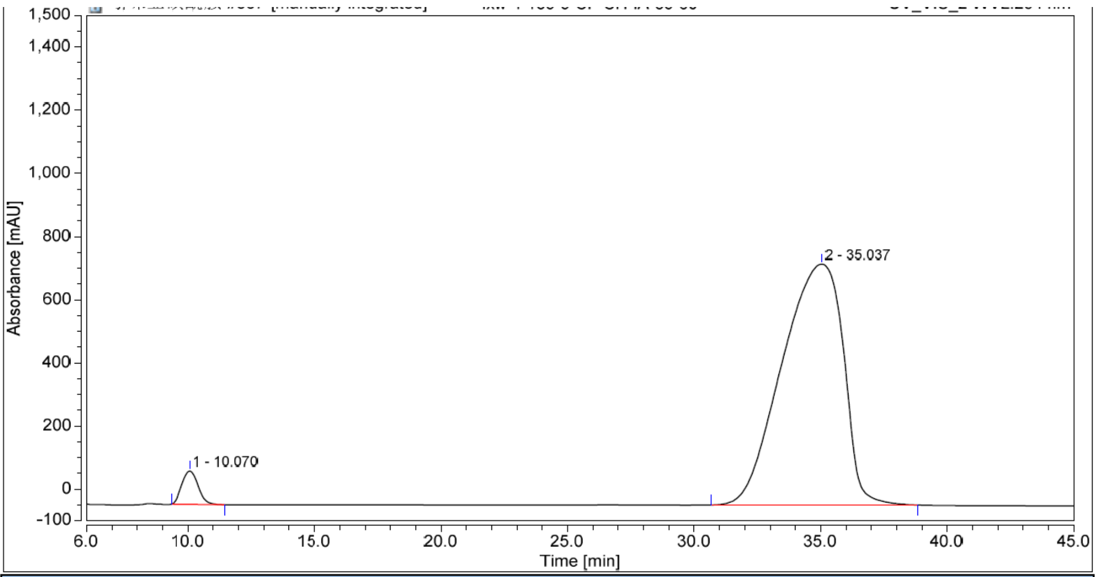

Integration Results

No.	Peak Name	Retention Time min	Area mAU*min	Height mAU	Relative Area %	Relative Height %	Amount n.a.
1		10.070	78.944	105.653	3.55	12.17	n.a.
2		35.037	2146.039	762.715	96.45	87.83	n.a.
**Total:**			**2224.982**	**868.369**	**100.00**	**100.00**	

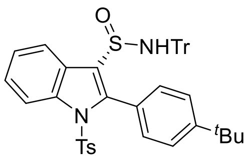

5b

Chromatogram

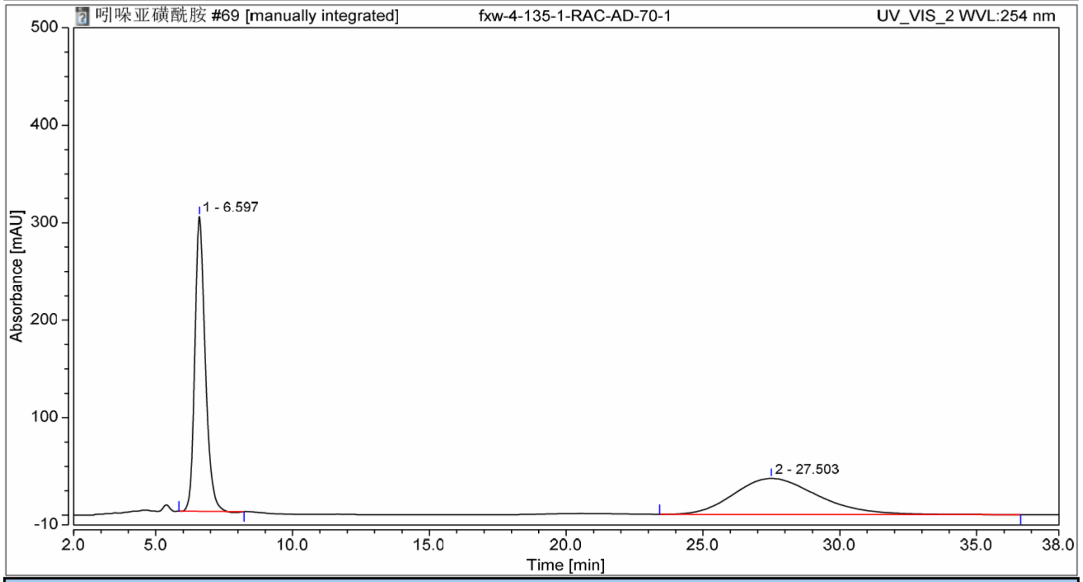

Integration Results

No.	Peak Name	Retention Time min	Area mAU*min	Height mAU	Relative Area %	Relative Height %	Amount n.a.
1		6.597	133.940	302.262	50.43	89.13	n.a.
2		27.503	131.642	36.877	49.57	10.87	n.a.
**Total:**			**265.581**	**339.139**	**100.00**	**100.00**	

Chromatogram

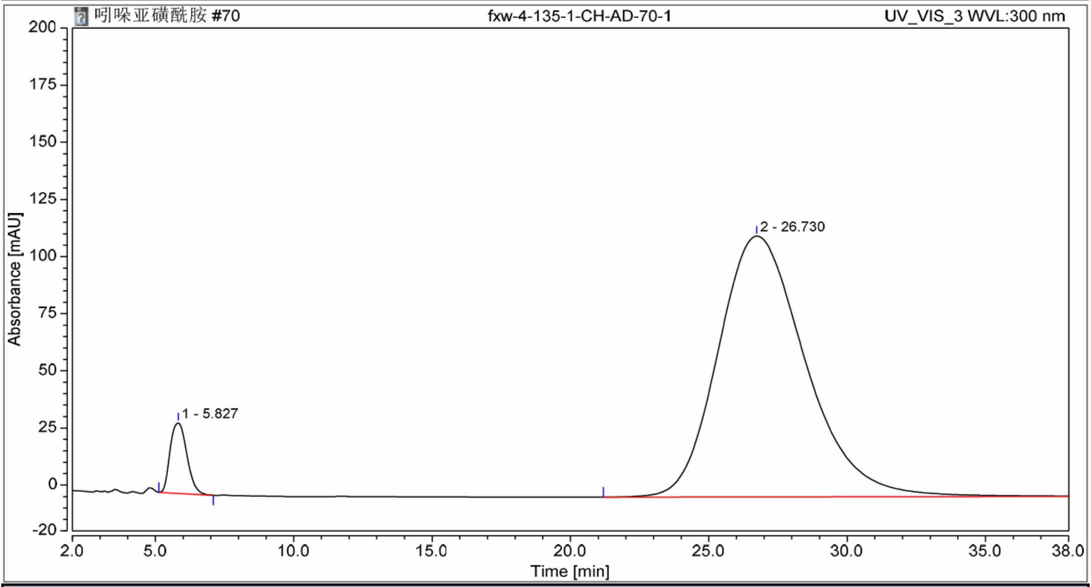

Integration Results

No.	Peak Name	Retention Time min	Area mAU*min	Height mAU	Relative Area %	Relative Height %	Amount n.a.
1		5.827	21.514	30.826	4.89	21.26	n.a.
2		26.730	418.168	114.184	95.11	78.74	n.a.
**Total:**			**439.682**	**145.009**	**100.00**	**100.00**	

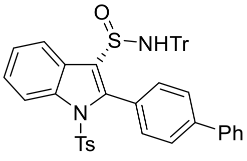

6b

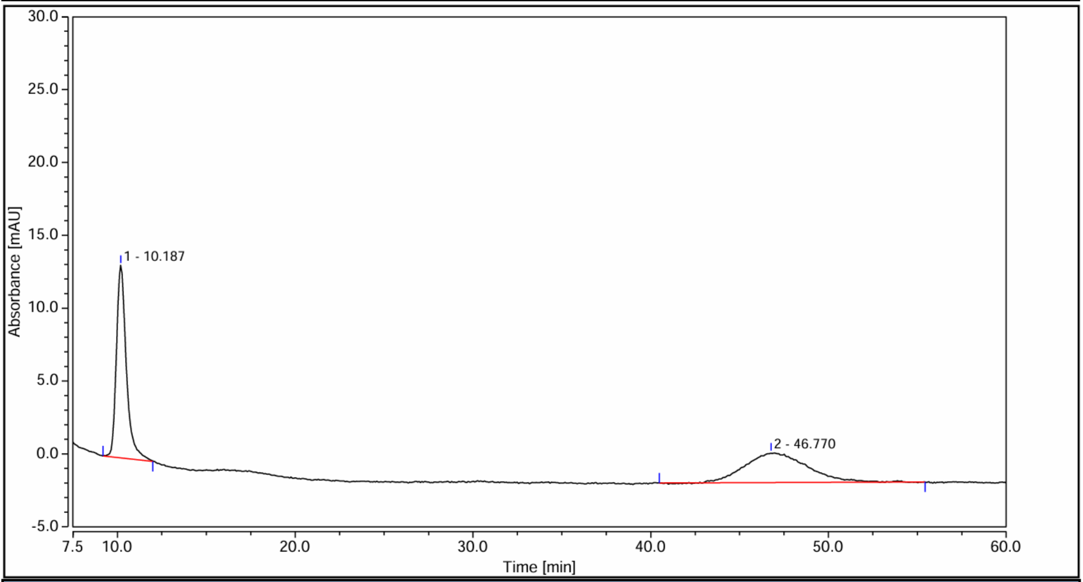

Integration Results

No.	Peak Name	Retention Time min	Area mAU*min	Height mAU	Relative Area %	Relative Height %	Amount n.a.
1		10.187	8.569	13.221	51.24	86.71	n.a.
2		46.770	8.153	2.026	48.76	13.29	n.a.
**Total:**			**16.722**	**15.247**	**100.00**	**100.00**	

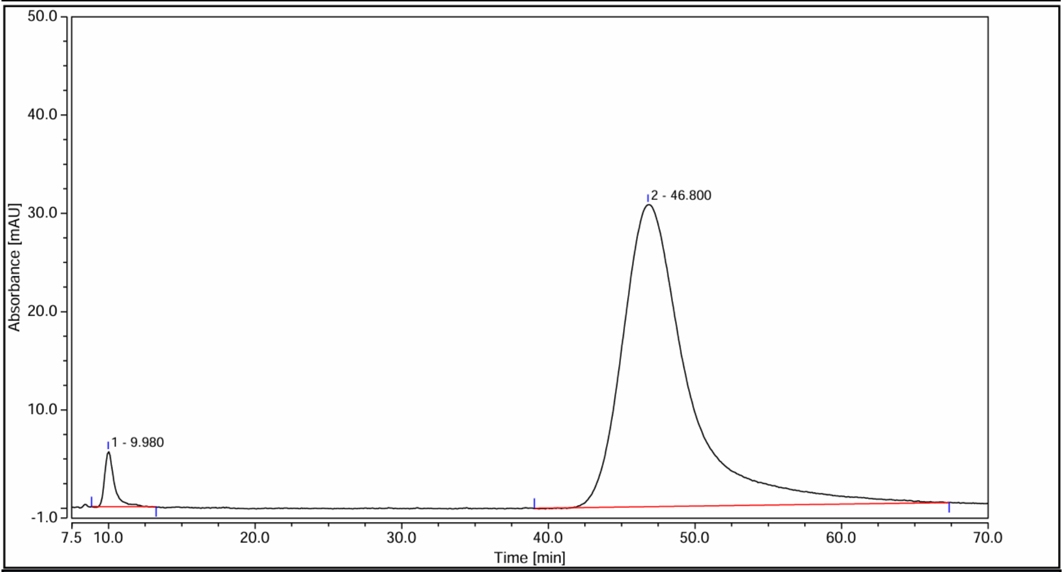

Integration Results

No.	Peak Name	Retention Time min	Area mAU*min	Height mAU	Relative Area %	Relative Height %	Amount n.a.
1		9.980	4.298	5.615	2.65	15.44	n.a.
2		46.800	157.807	30.745	97.35	84.56	n.a.
**Total:**			**162.105**	**36.359**	**100.00**	**100.00**	

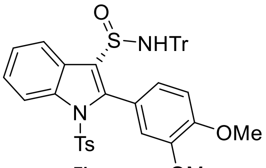

7b

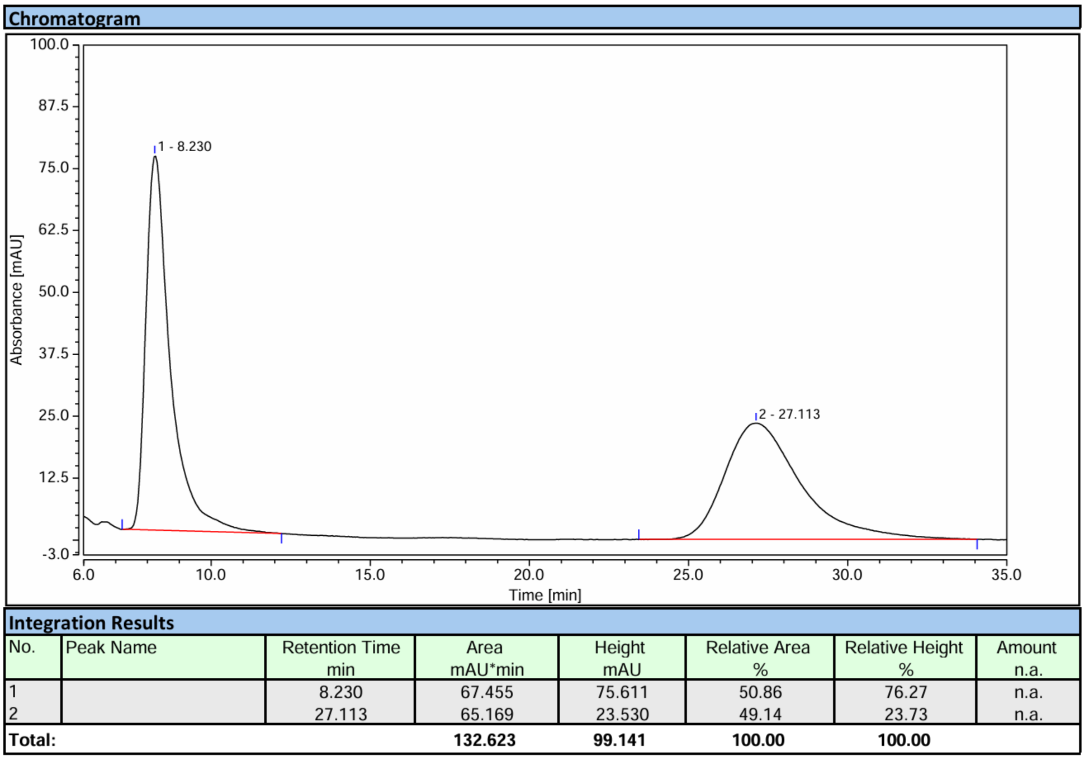

Integration Results

No.	Peak Name	Retention Time min	Area mAU*min	Height mAU	Relative Area %	Relative Height %	Amount n.a.
1		8.230	67.455	75.611	50.86	76.27	n.a.
2		27.113	65.169	23.530	49.14	23.73	n.a.
**Total:**			**132.623**	**99.141**	**100.00**	**100.00**	

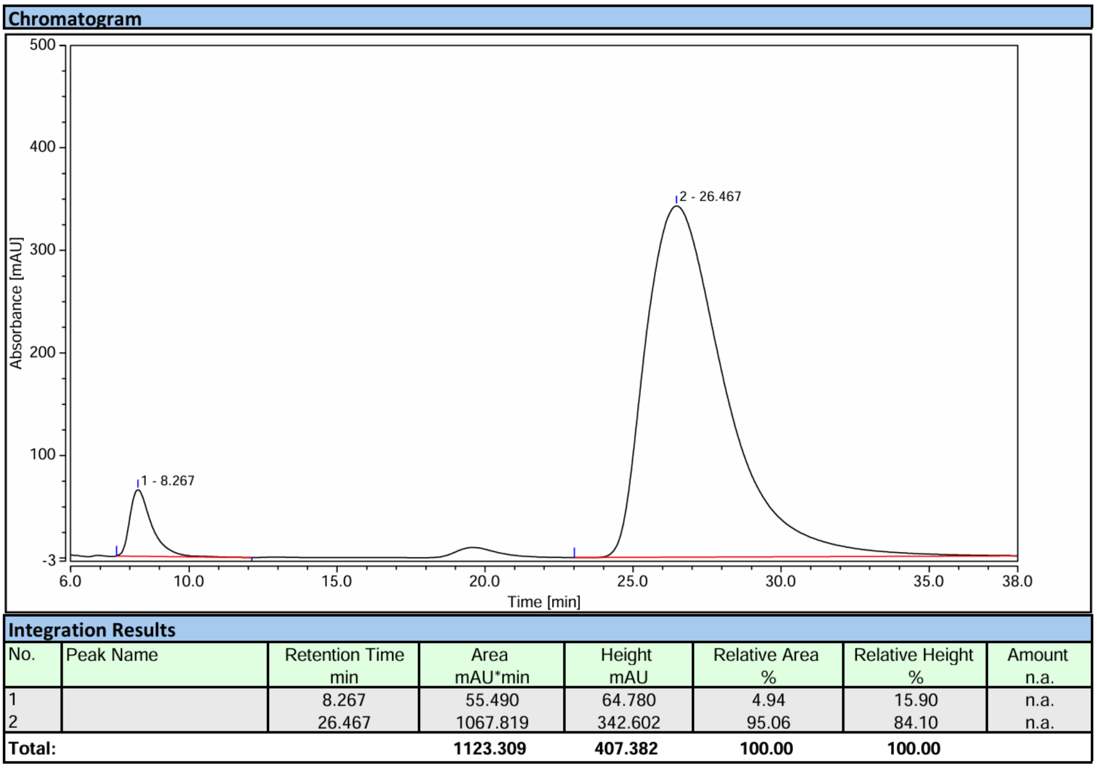

Integration Results

No.	Peak Name	Retention Time min	Area mAU*min	Height mAU	Relative Area %	Relative Height %	Amount n.a.
1		8.267	55.490	64.780	4.94	15.90	n.a.
2		26.467	1067.819	342.602	95.06	84.10	n.a.
**Total:**			**1123.309**	**407.382**	**100.00**	**100.00**	

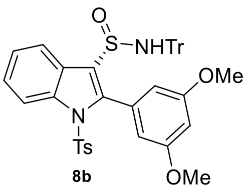

8b

Chromatogram

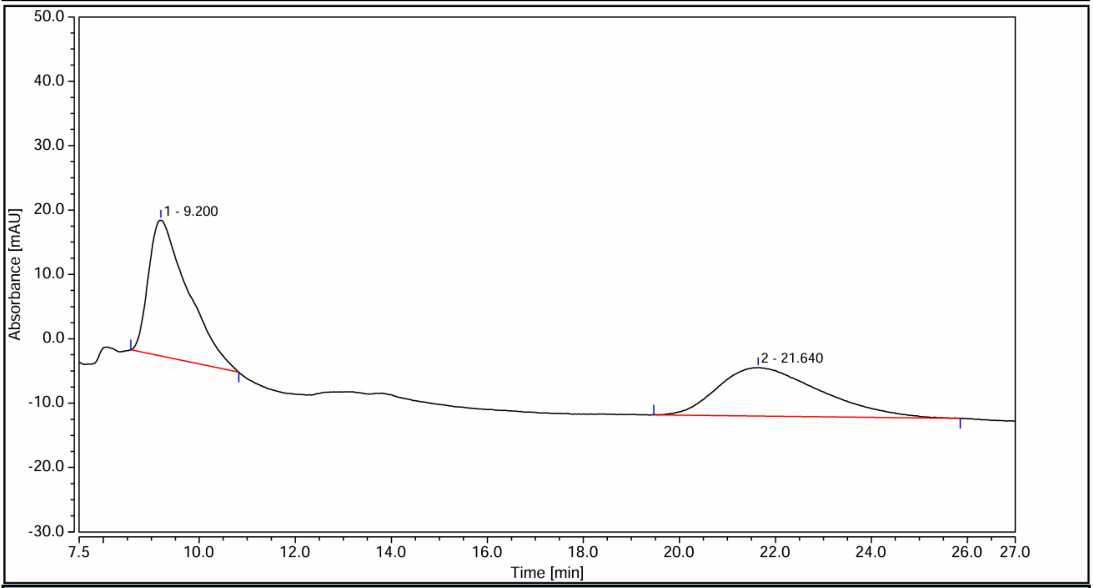

Integration Results

No.	Peak Name	Retention Time min	Area mAU*min	Height mAU	Relative Area %	Relative Height %	Amount n.a.
1		9.200	20.135	21.124	51.98	73.73	n.a.
2		21.640	18.605	7.528	48.02	26.27	n.a.
**Total:**			**38.740**	**28.653**	**100.00**	**100.00**	

Chromatogram

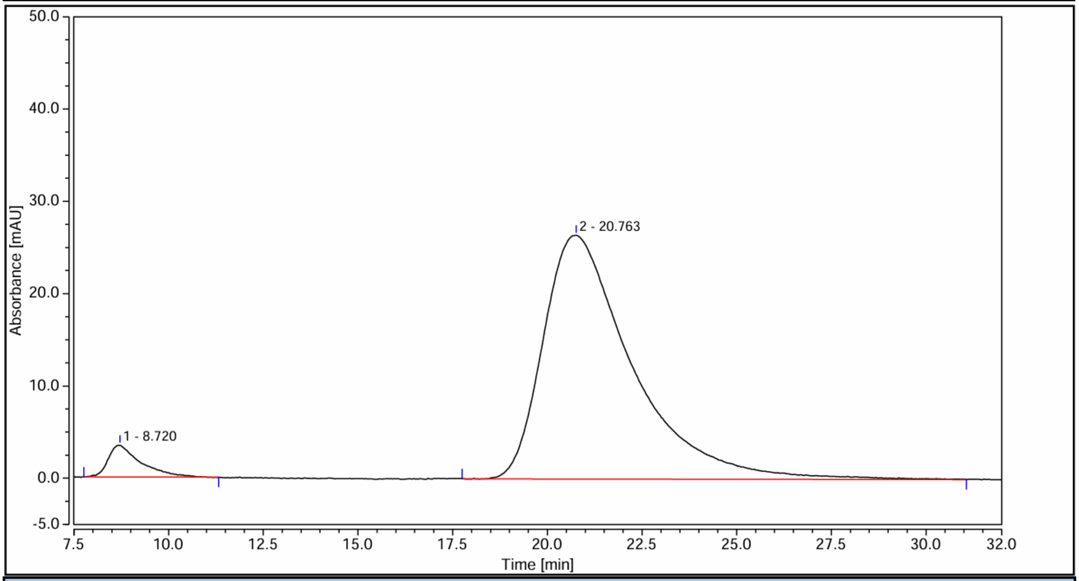

Integration Results

No.	Peak Name	Retention Time min	Area mAU*min	Height mAU	Relative Area %	Relative Height %	Amount n.a.
1		8.720	3.462	3.447	4.60	11.55	n.a.
2		20.763	71.883	26.407	95.40	88.45	n.a.
**Total:**			**75.345**	**29.854**	**100.00**	**100.00**	

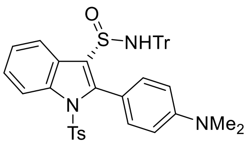

9b

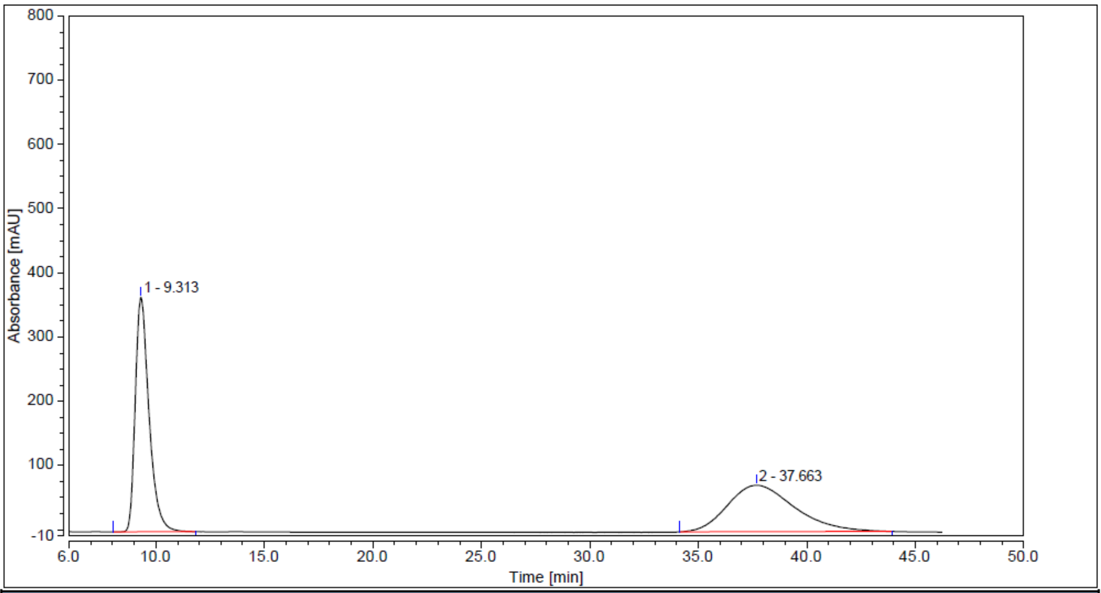

Integration Results

No.	Peak Name	Retention Time min	Area mAU*min	Height mAU	Relative Area %	Relative Height %	Amount n.a.
1		9.313	268.081	365.272	50.86	83.48	n.a.
2		37.663	259.009	72.286	49.14	16.52	n.a.
**Total:**			**527.090**	**437.558**	**100.00**	**100.00**	

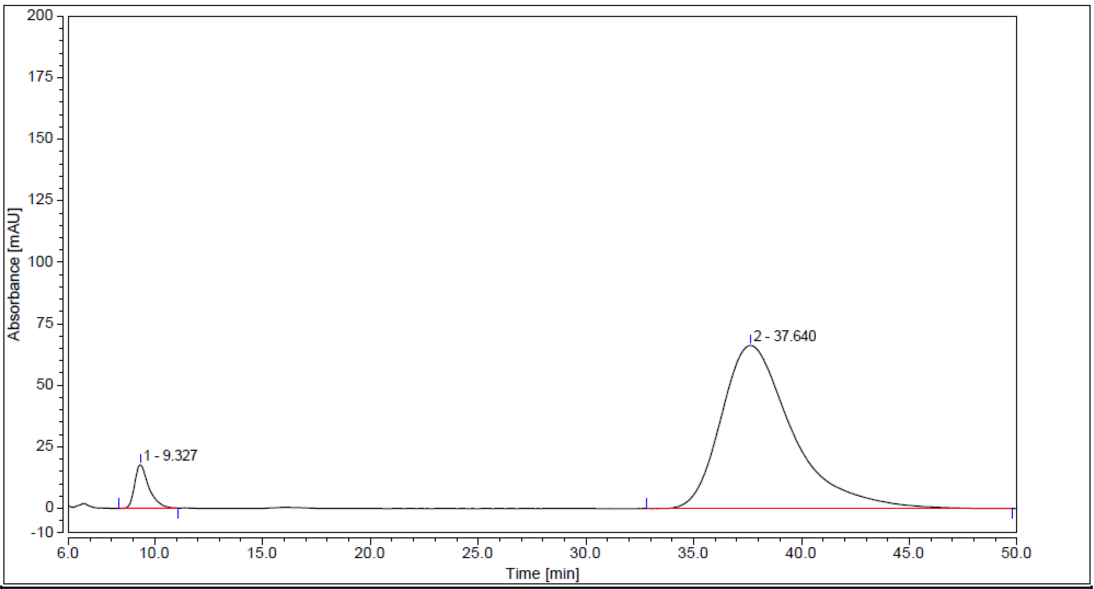

Integration Results

No.	Peak Name	Retention Time min	Area mAU*min	Height mAU	Relative Area %	Relative Height %	Amount n.a.
1		9.327	13.416	17.665	5.02	21.02	n.a.
2		37.640	253.985	66.372	94.98	78.98	n.a.
**Total:**			**267.401**	**84.037**	**100.00**	**100.00**	

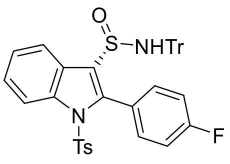

10b

Chromatogram

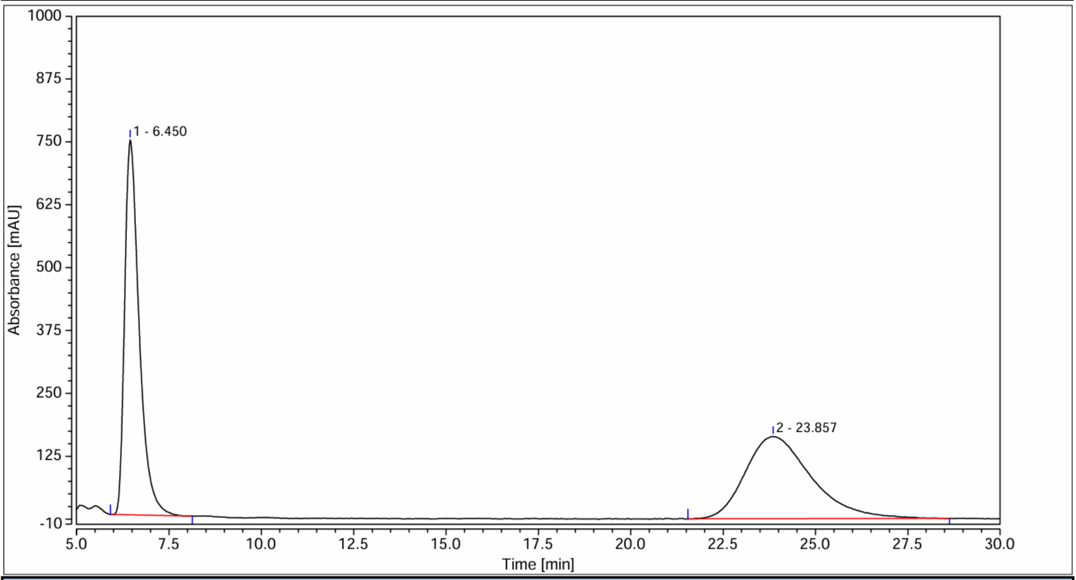

Integration Results

No.	Peak Name	Retention Time<br>min	Area<br>mAU*min	Height<br>mAU	Relative Area<br>%	Relative Height<br>%	Amount<br>n.a.
1		6.450	341.612	745.705	50.22	82.00	n.a.
2		23.857	338.683	163.668	49.78	18.00	n.a.
**Total:**			**680.295**	**909.373**	**100.00**	**100.00**	

Chromatogram

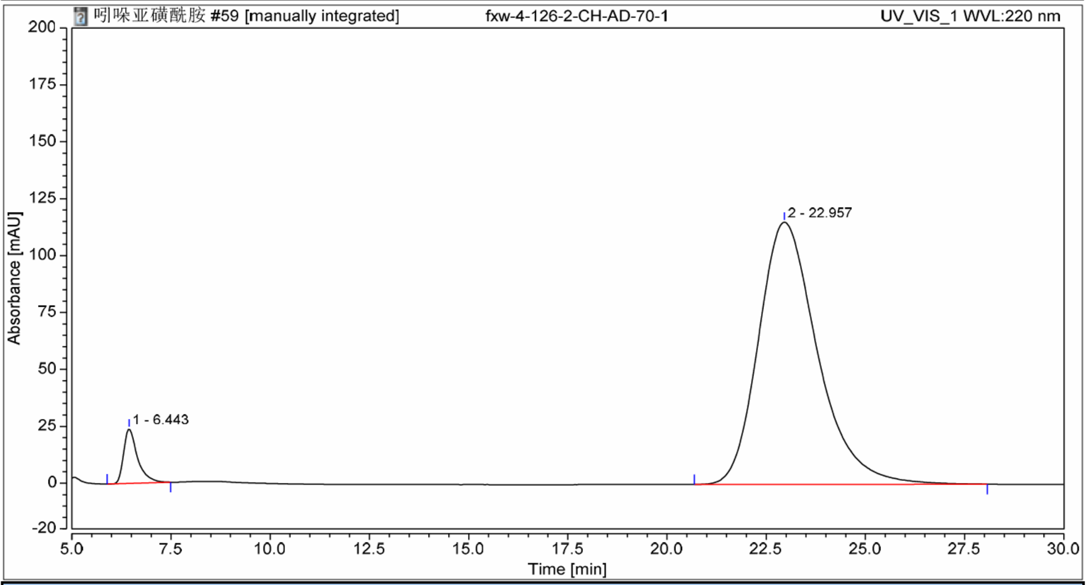

Integration Results

No.	Peak Name	Retention Time<br>min	Area<br>mAU*min	Height<br>mAU	Relative Area<br>%	Relative Height<br>%	Amount<br>n.a.
1		6.443	9.686	23.810	4.65	17.13	n.a.
2		22.957	198.489	115.218	95.35	82.87	n.a.
**Total:**			**208.175**	**139.028**	**100.00**	**100.00**	

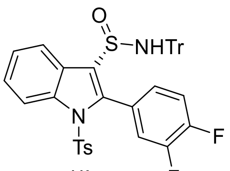

11b

Chromatogram

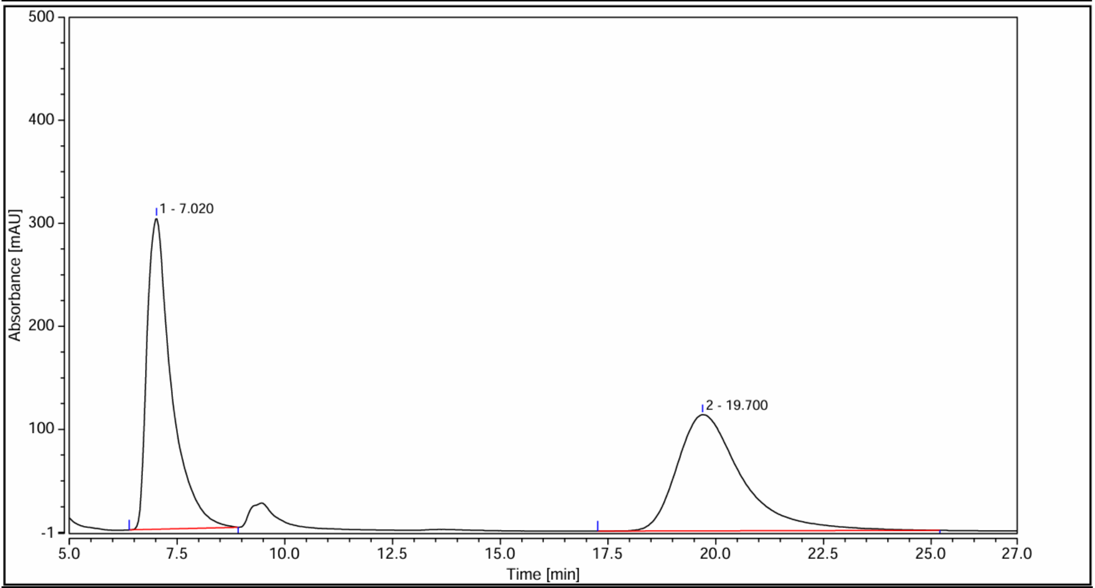

Integration Results

No.	Peak Name	Retention Time min	Area mAU*min	Height mAU	Relative Area %	Relative Height %	Amount n.a.
1		7.020	198.250	301.964	50.29	72.82	n.a.
2		19.700	195.980	112.704	49.71	27.18	n.a.
**Total:**			**394.230**	**414.668**	**100.00**	**100.00**	

Chromatogram

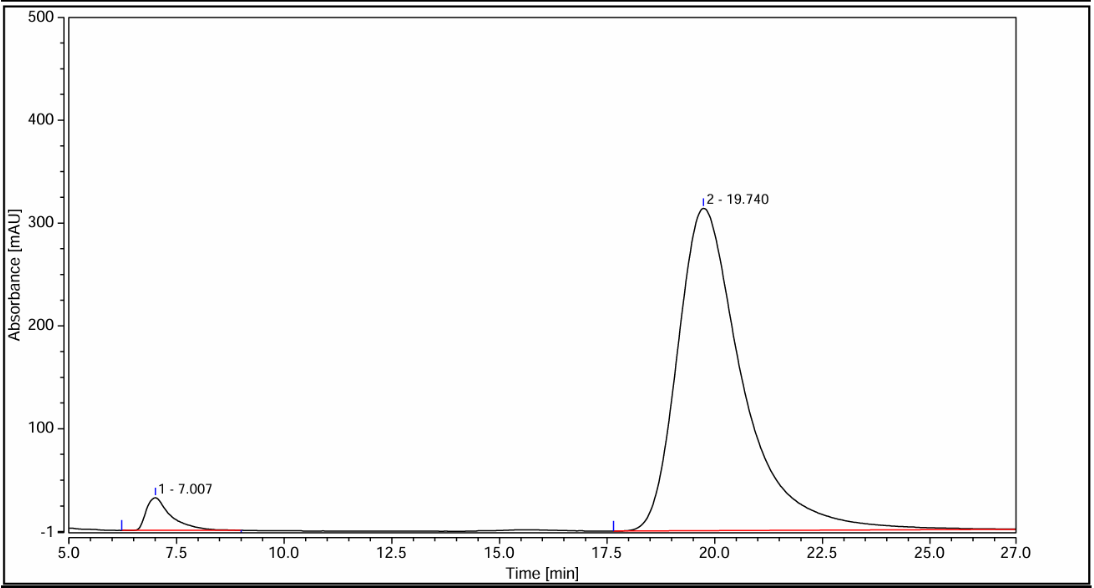

Integration Results

No.	Peak Name	Retention Time min	Area mAU*min	Height mAU	Relative Area %	Relative Height %	Amount n.a.
1		7.007	21.165	31.705	3.71	9.19	n.a.
2		19.740	549.296	313.437	96.29	90.81	n.a.
**Total:**			**570.461**	**345.142**	**100.00**	**100.00**	

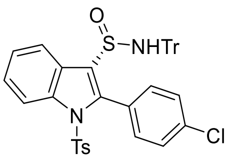

12b

Chromatogram

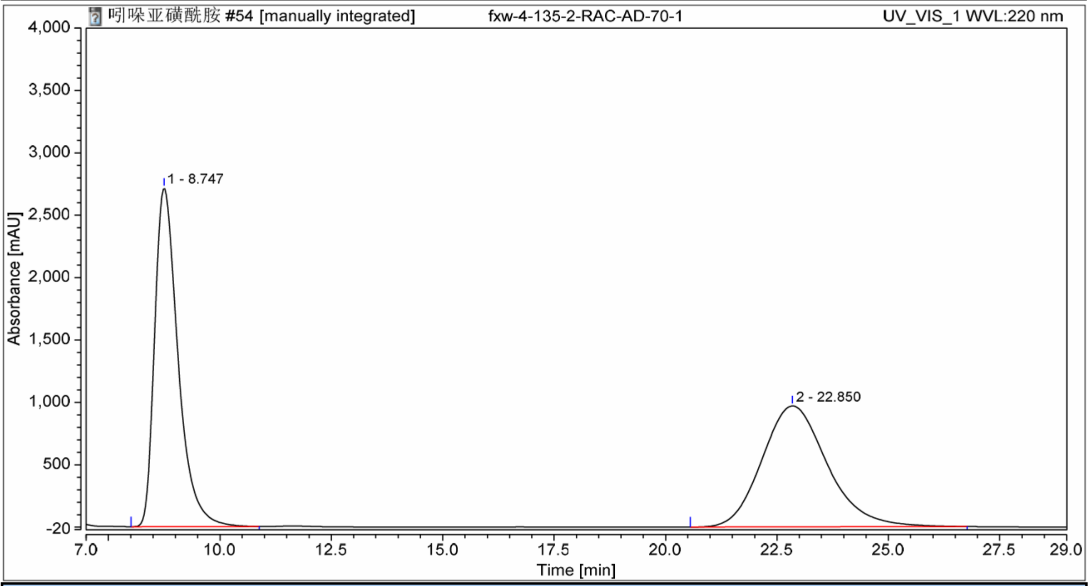

Integration Results

No.	Peak Name	Retention Time min	Area mAU*min	Height mAU	Relative Area %	Relative Height %	Amount n.a.
1		8.747	1648.776	2714.007	49.99	73.69	n.a.
2		22.850	1649.141	969.046	50.01	26.31	n.a.
**Total:**			**3297.917**	**3683.053**	**100.00**	**100.00**	

Chromatogram

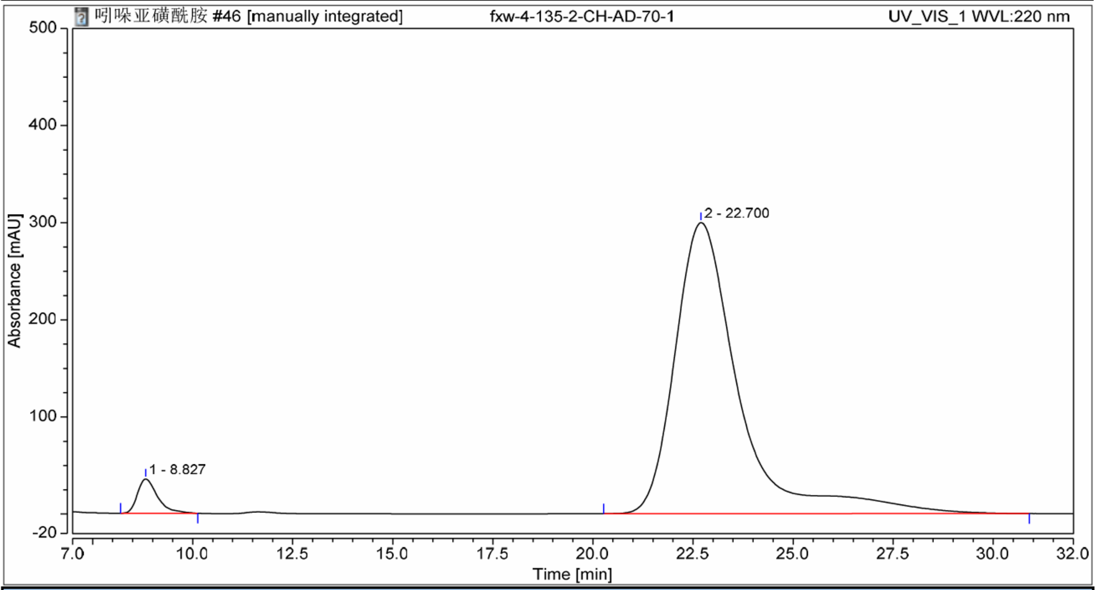

Integration Results

No.	Peak Name	Retention Time min	Area mAU*min	Height mAU	Relative Area %	Relative Height %	Amount n.a.
1		8.827	20.397	35.284	3.55	10.53	n.a.
2		22.700	554.438	299.804	96.45	89.47	n.a.
**Total:**			**574.835**	**335.087**	**100.00**	**100.00**	

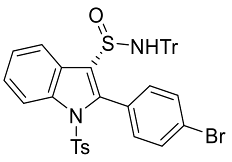

13b

Chromatogram

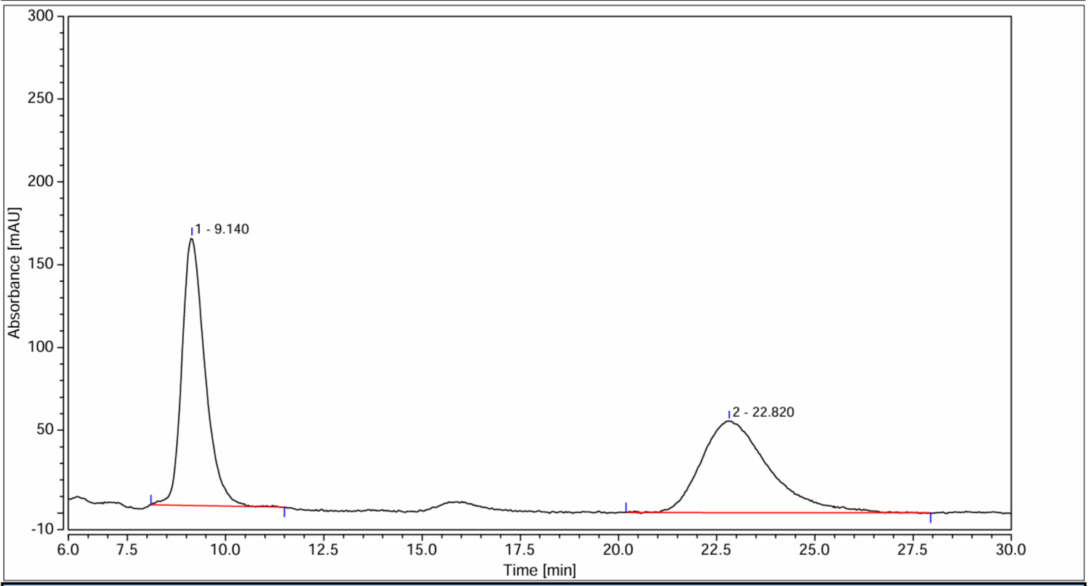

Integration Results

No.	Peak Name	Retention Time min	Area mAU*min	Height mAU	Relative Area %	Relative Height %	Amount n.a.
1		9.140	109.984	161.667	49.89	74.48	n.a.
2		22.820	110.481	55.392	50.11	25.52	n.a.
**Total:**			**220.465**	**217.059**	**100.00**	**100.00**	

Chromatogram

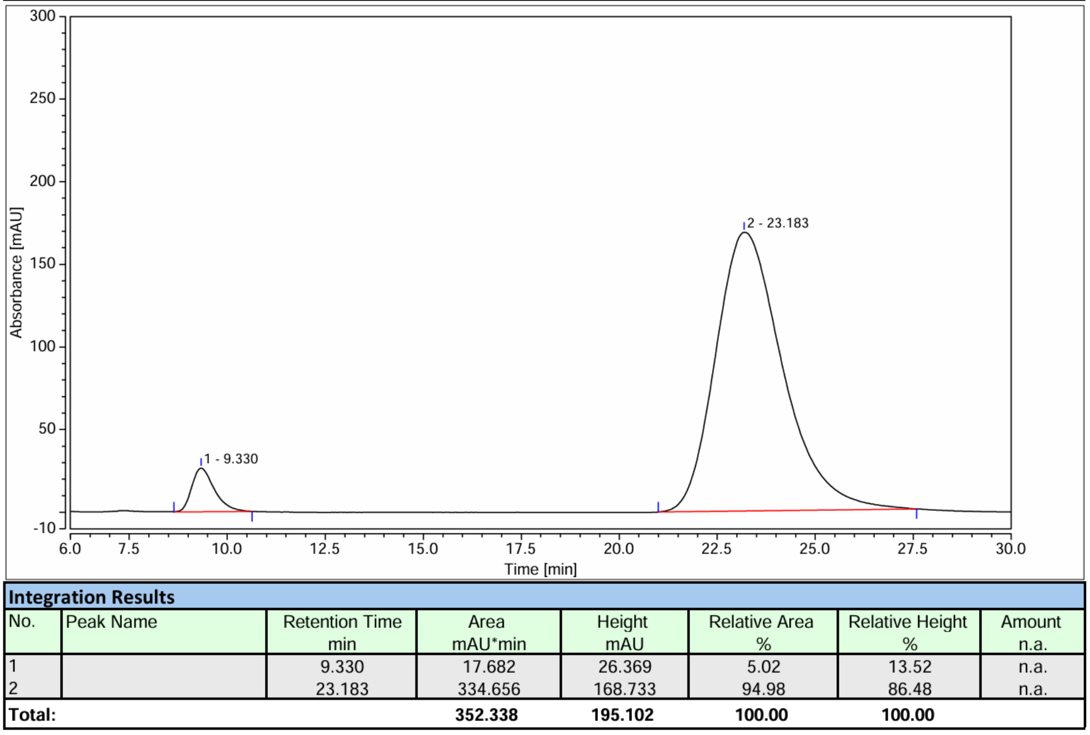

Integration Results

No.	Peak Name	Retention Time min	Area mAU*min	Height mAU	Relative Area %	Relative Height %	Amount n.a.
1		9.330	17.682	26.369	5.02	13.52	n.a.
2		23.183	334.656	168.733	94.98	86.48	n.a.
**Total:**			**352.338**	**195.102**	**100.00**	**100.00**	

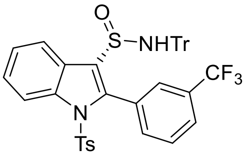

14b

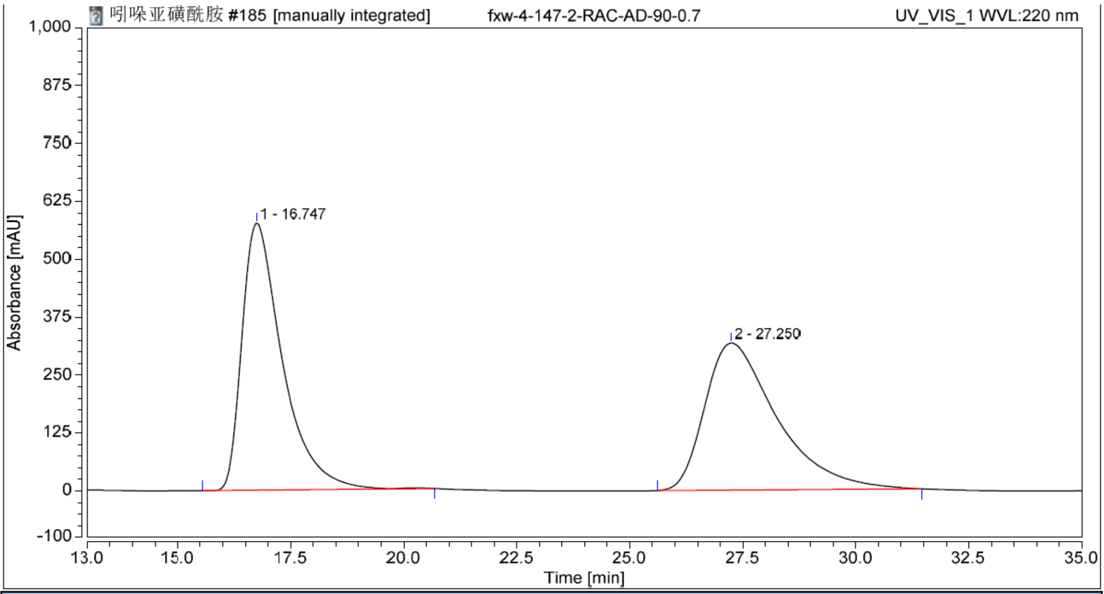

Integration Results

No.	Peak Name	Retention Time min	Area mAU*min	Height mAU	Relative Area %	Relative Height %	Amount n.a.
1		16.747	596.896	576.716	50.48	64.50	n.a.
2		27.250	585.583	317.396	49.52	35.50	n.a.
**Total:**			**1182.480**	**894.112**	**100.00**	**100.00**	

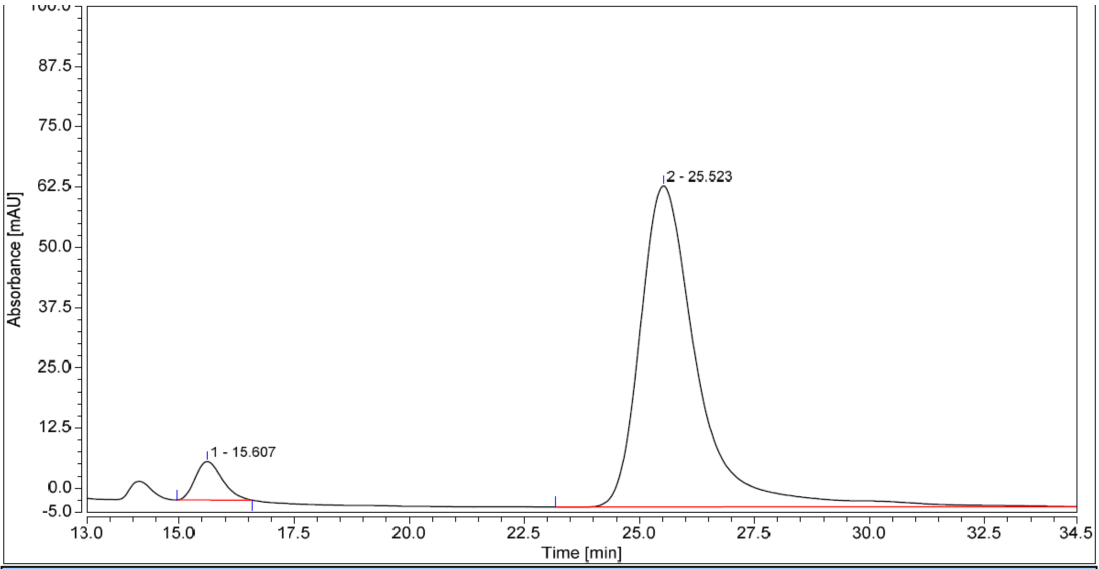

Integration Results

No.	Peak Name	Retention Time min	Area mAU*min	Height mAU	Relative Area %	Relative Height %	Amount n.a.
1		15.607	5.414	7.966	5.32	10.68	n.a.
2		25.523	96.294	66.600	94.68	89.32	n.a.
**Total:**			**101.708**	**74.567**	**100.00**	**100.00**	

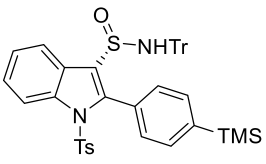

15b

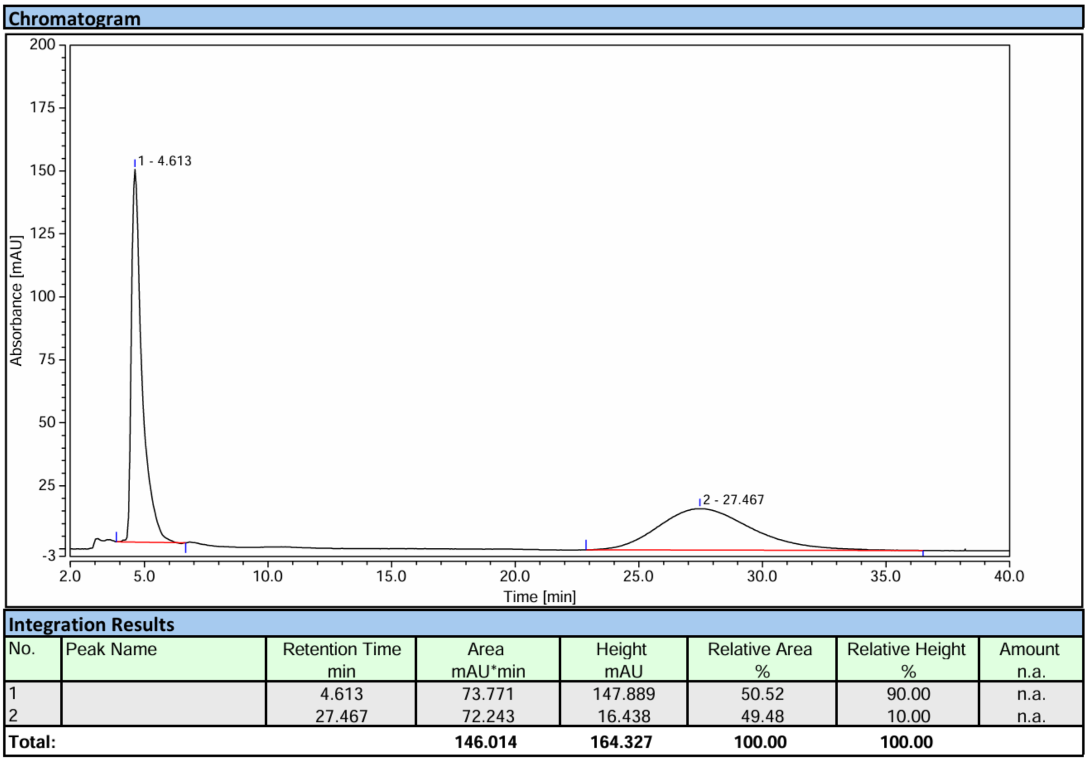

Integration Results

No.	Peak Name	Retention Time min	Area mAU*min	Height mAU	Relative Area %	Relative Height %	Amount n.a.
1		4.613	73.771	147.889	50.52	90.00	n.a.
2		27.467	72.243	16.438	49.48	10.00	n.a.
**Total:**			**146.014**	**164.327**	**100.00**	**100.00**	

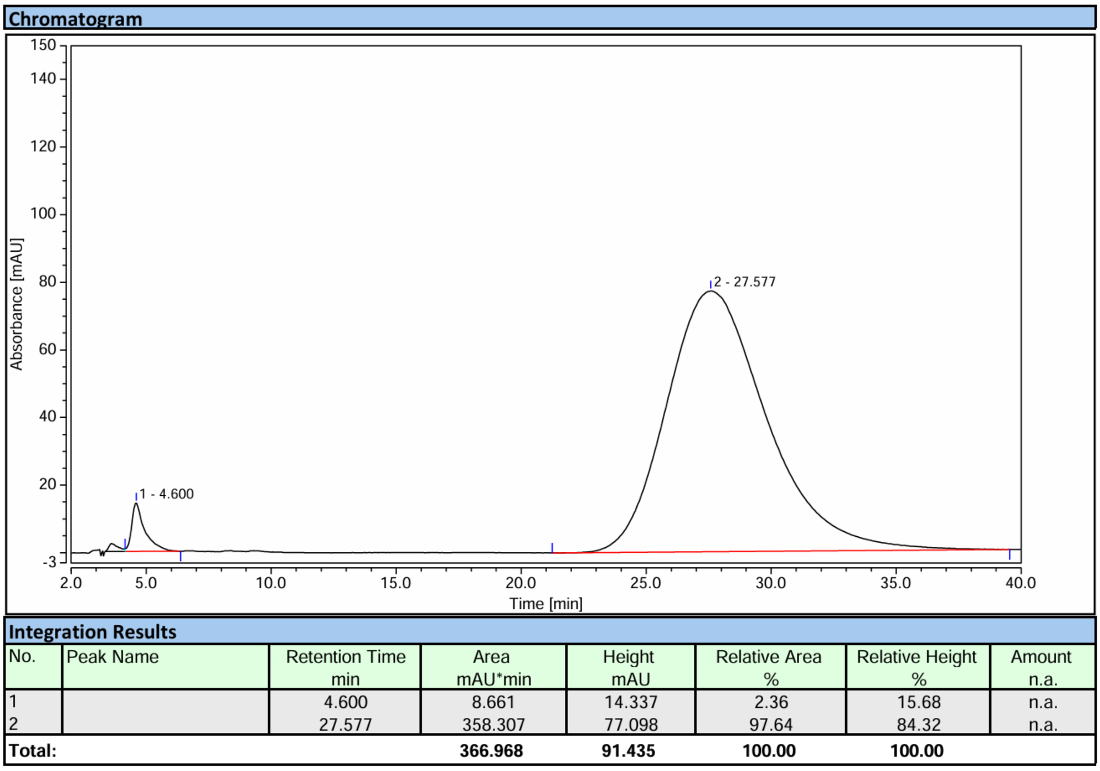

Integration Results

No.	Peak Name	Retention Time min	Area mAU*min	Height mAU	Relative Area %	Relative Height %	Amount n.a.
1		4.600	8.661	14.337	2.36	15.68	n.a.
2		27.577	358.307	77.098	97.64	84.32	n.a.
**Total:**			**366.968**	**91.435**	**100.00**	**100.00**	

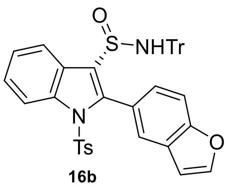

16b

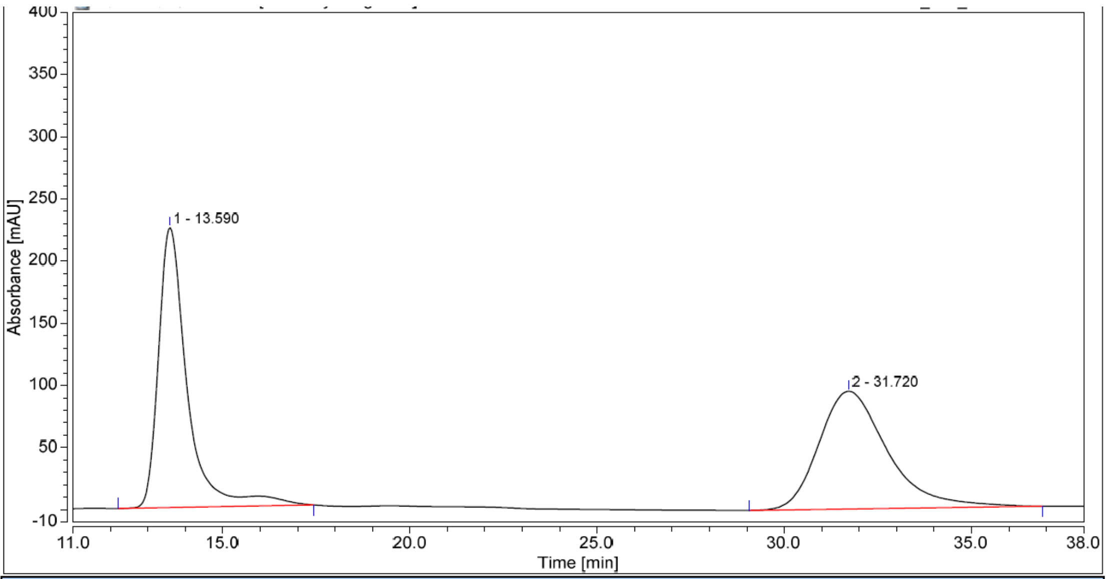

Integration Results							
No.	Peak Name	Retention Time min	Area mAU*min	Height mAU	Relative Area %	Relative Height %	Amount n.a.
1		13.590	200.754	225.220	49.99	70.40	n.a.
2		31.720	200.856	94.701	50.01	29.60	n.a.
**Total:**			**401.610**	**319.921**	**100.00**	**100.00**	

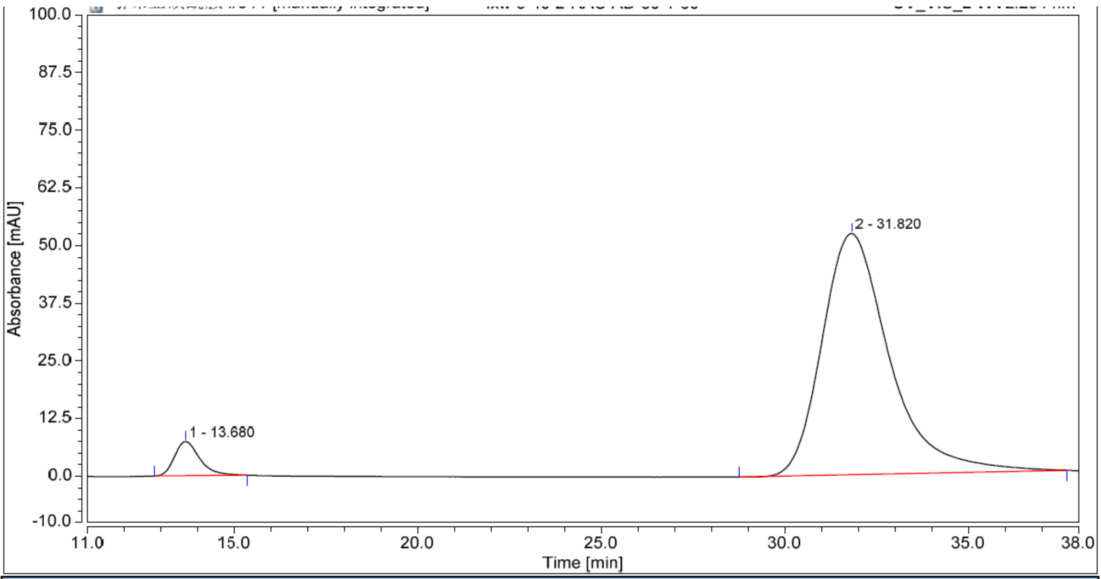

Integration Results							
No.	Peak Name	Retention Time min	Area mAU*min	Height mAU	Relative Area %	Relative Height %	Amount n.a.
1		13.680	5.914	7.420	5.04	12.42	n.a.
2		31.820	111.534	52.311	94.96	87.58	n.a.
**Total:**			**117.447**	**59.731**	**100.00**	**100.00**	

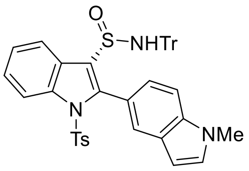

17b

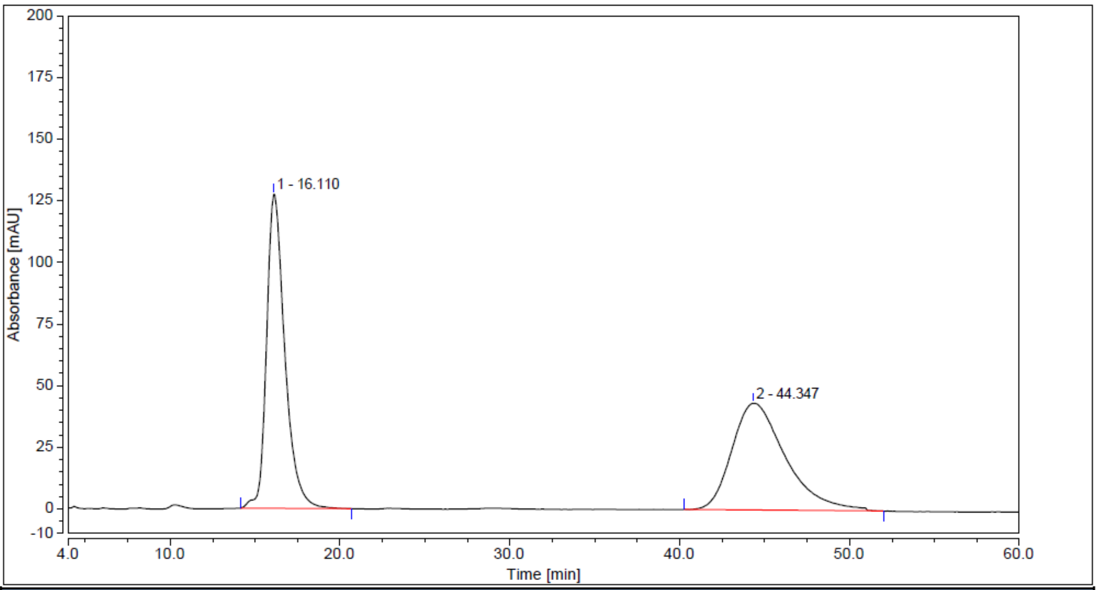

Integration Results							
No.	Peak Name	Retention Time min	Area mAU*min	Height mAU	Relative Area %	Relative Height %	Amount n.a.
1		16.110	158.948	127.584	50.07	74.64	n.a.
2		44.347	158.478	43.348	49.93	25.36	n.a.
**Total:**			**317.426**	**170.933**	**100.00**	**100.00**	

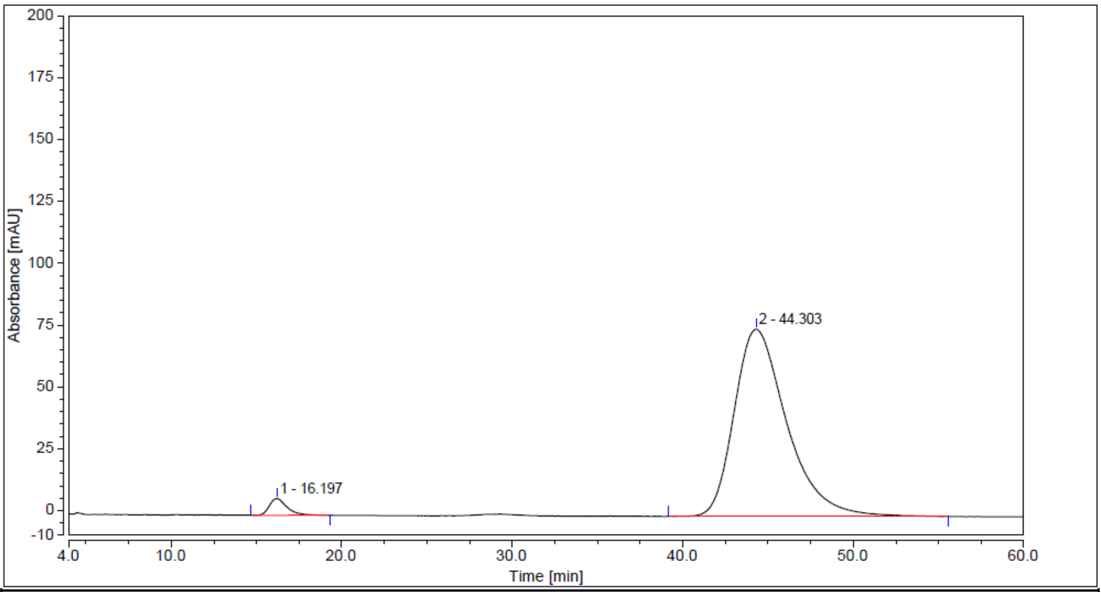

Integration Results							
No.	Peak Name	Retention Time min	Area mAU*min	Height mAU	Relative Area %	Relative Height %	Amount n.a.
1		16.197	8.144	6.813	2.93	8.25	n.a.
2		44.303	270.171	75.791	97.07	91.75	n.a.
**Total:**			**278.315**	**82.605**	**100.00**	**100.00**	

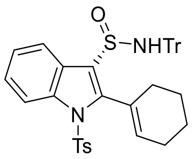

18b

Chromatogram

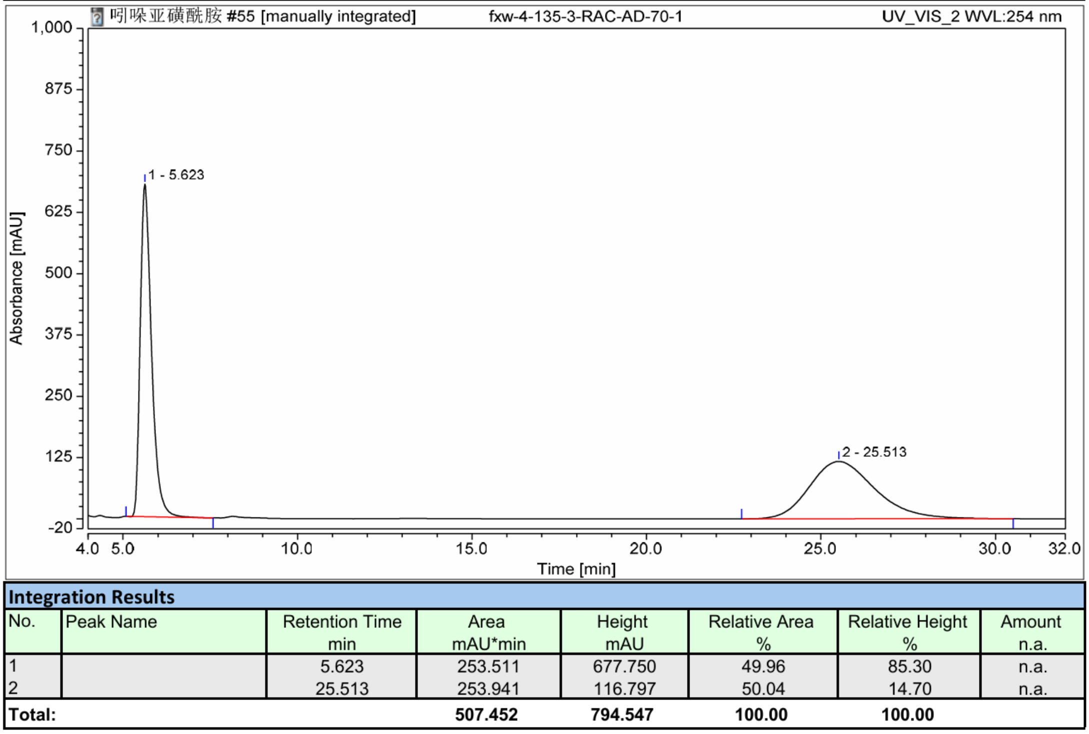

Integration Results

No.	Peak Name	Retention Time min	Area mAU*min	Height mAU	Relative Area %	Relative Height %	Amount n.a.
1		5.623	253.511	677.750	49.96	85.30	n.a.
2		25.513	253.941	116.797	50.04	14.70	n.a.
**Total:**			**507.452**	**794.547**	**100.00**	**100.00**	

Chromatogram

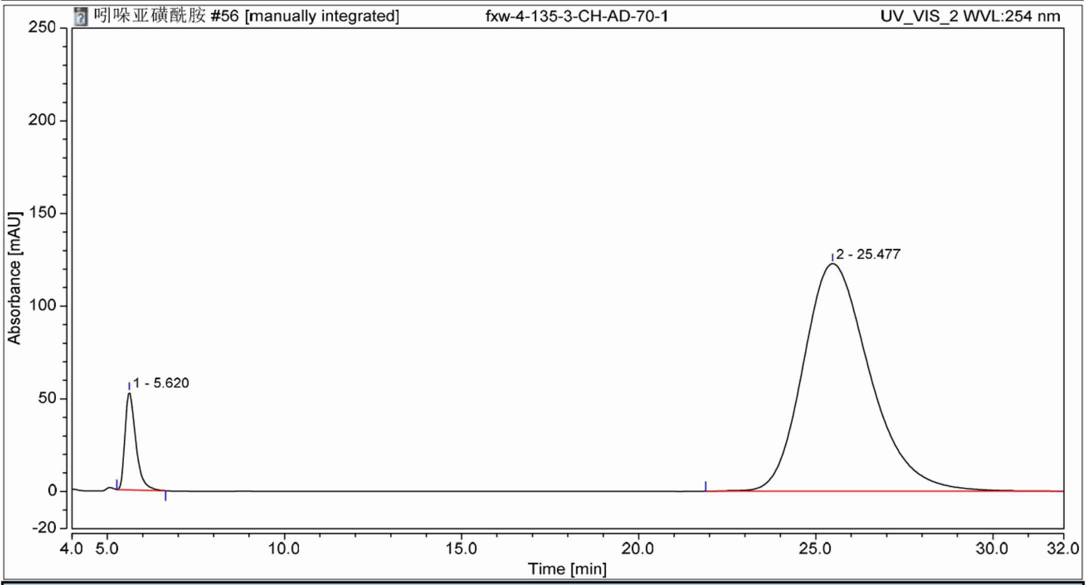

Integration Results

No.	Peak Name	Retention Time min	Area mAU*min	Height mAU	Relative Area %	Relative Height %	Amount n.a.
1		5.620	18.611	52.611	6.50	30.00	n.a.
2		25.477	267.821	122.772	93.50	70.00	n.a.
**Total:**			**286.432**	**175.383**	**100.00**	**100.00**	

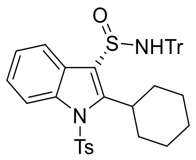

19b

Chromatogram

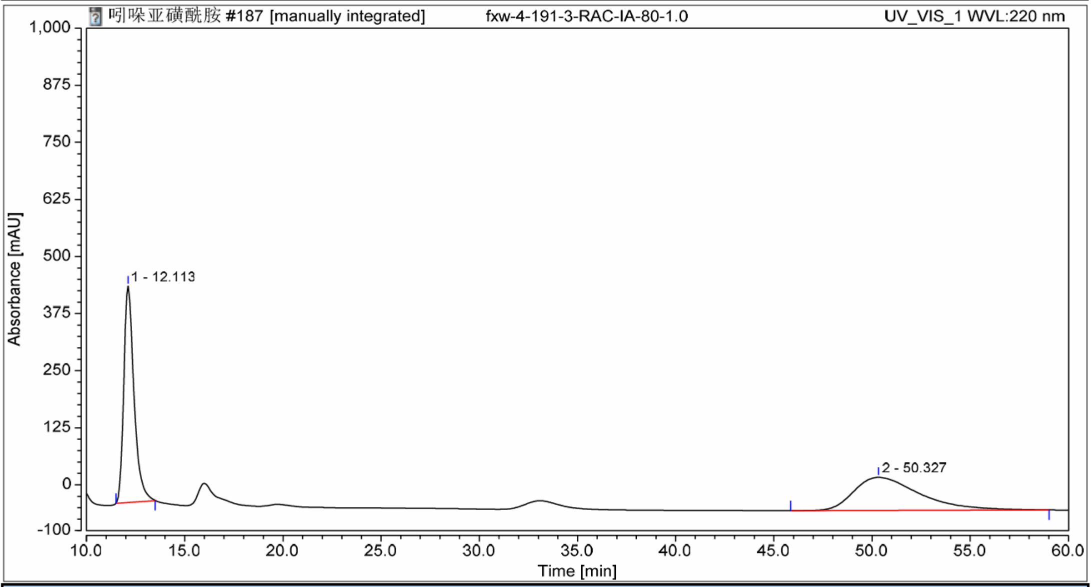

Integration Results

No.	Peak Name	Retention Time min	Area mAU*min	Height mAU	Relative Area %	Relative Height %	Amount n.a.
1		12.113	285.006	473.782	50.44	86.79	n.a.
2		50.327	280.044	72.142	49.56	13.21	n.a.
**Total:**			**565.050**	**545.923**	**100.00**	**100.00**	

Chromatogram

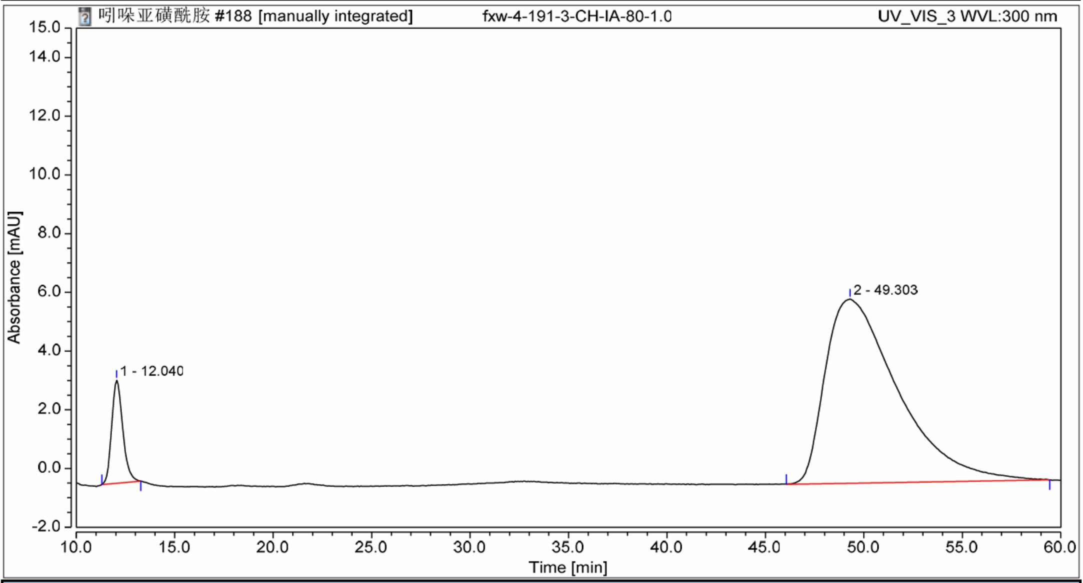

Integration Results

No.	Peak Name	Retention Time min	Area mAU*min	Height mAU	Relative Area %	Relative Height %	Amount n.a.
1		12.040	2.294	3.513	7.88	35.91	n.a.
2		49.303	26.823	6.271	92.12	64.09	n.a.
**Total:**			**29.117**	**9.784**	**100.00**	**100.00**	

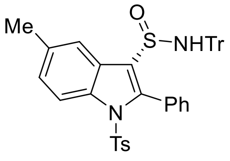

20b

Chromatogram

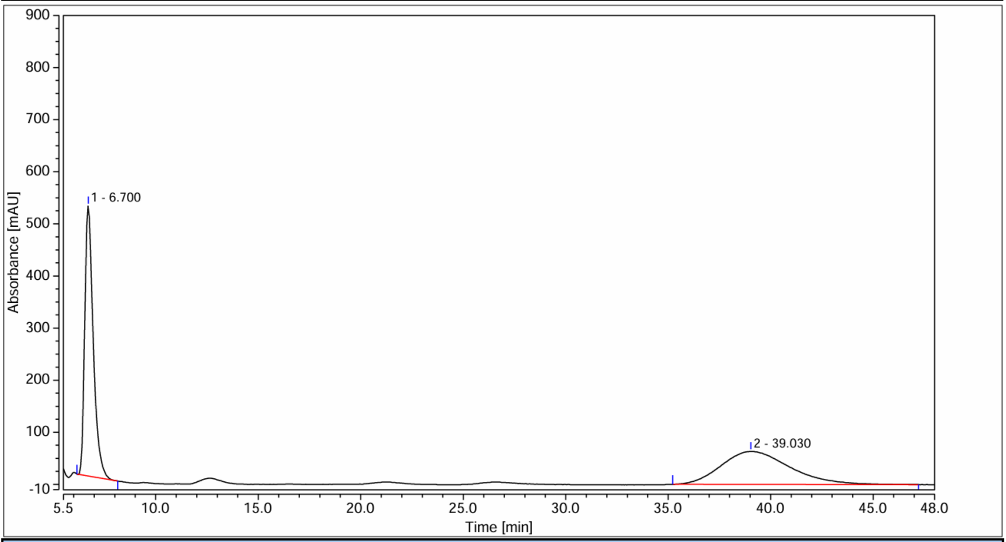

Integration Results

No.	Peak Name	Retention Time min	Area mAU*min	Height mAU	Relative Area %	Relative Height %	Amount n.a.
1		6.700	249.725	518.055	50.31	89.11	n.a.
2		39.030	246.615	63.308	49.69	10.89	n.a.
**Total:**			**496.340**	**581.362**	**100.00**	**100.00**	

Chromatogram

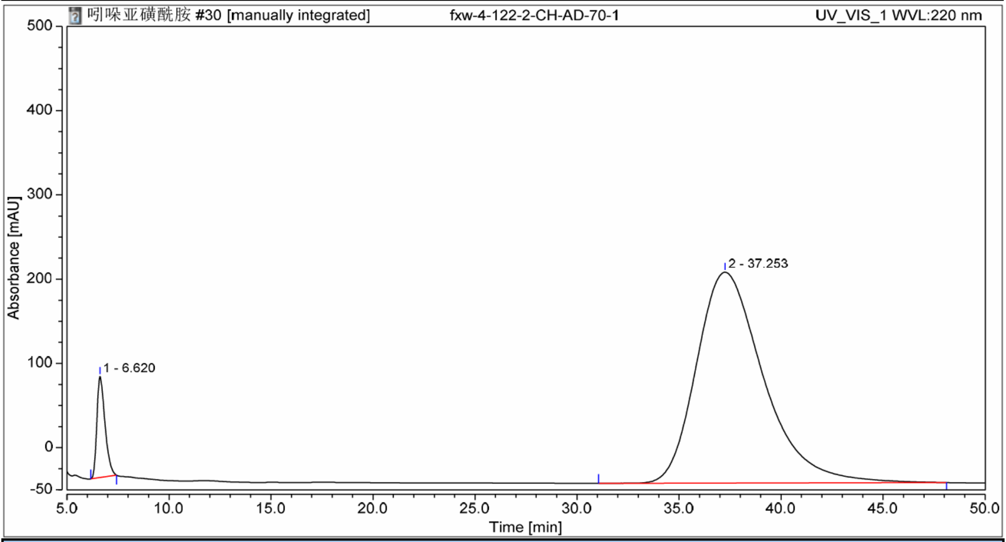

Integration Results

No.	Peak Name	Retention Time min	Area mAU*min	Height mAU	Relative Area %	Relative Height %	Amount n.a.
1		6.620	55.404	120.078	5.50	32.42	n.a.
2		37.253	952.223	250.341	94.50	67.58	n.a.
**Total:**			**1007.627**	**370.420**	**100.00**	**100.00**	

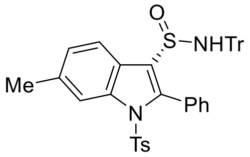

21b

Chromatogram

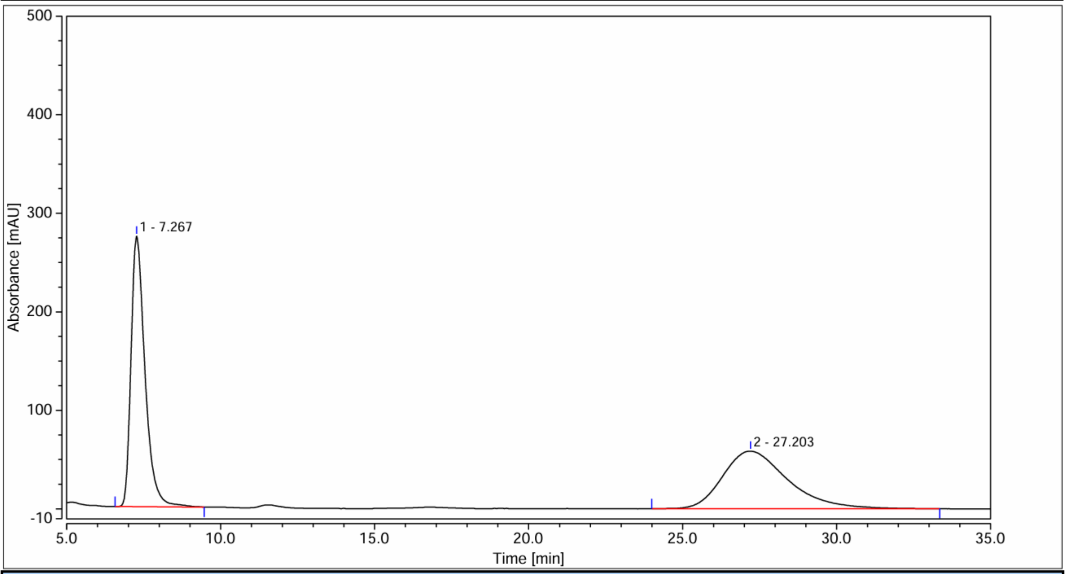

Integration Results

No.	Peak Name	Retention Time min	Area mAU*min	Height mAU	Relative Area %	Relative Height %	Amount n.a.
1		7.267	144.470	274.392	50.30	82.45	n.a.
2		27.203	142.738	58.395	49.70	17.55	n.a.
**Total:**			**287.208**	**332.787**	**100.00**	**100.00**	

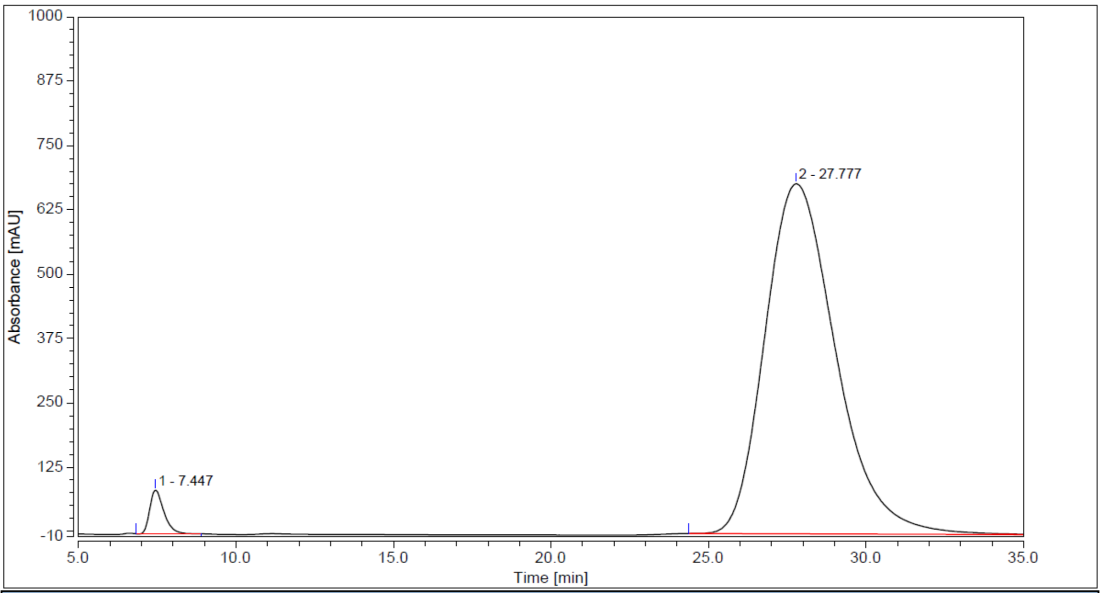

Integration Results

No.	Peak Name	Retention Time min	Area mAU*min	Height mAU	Relative Area %	Relative Height %	Amount n.a.
1		7.447	43.956	85.012	2.42	11.11	n.a.
2		27.777	1771.772	680.125	97.58	88.89	n.a.
**Total:**			**1815.728**	**765.137**	**100.00**	**100.00**	

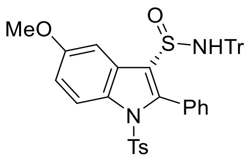

22b

Chromatogram

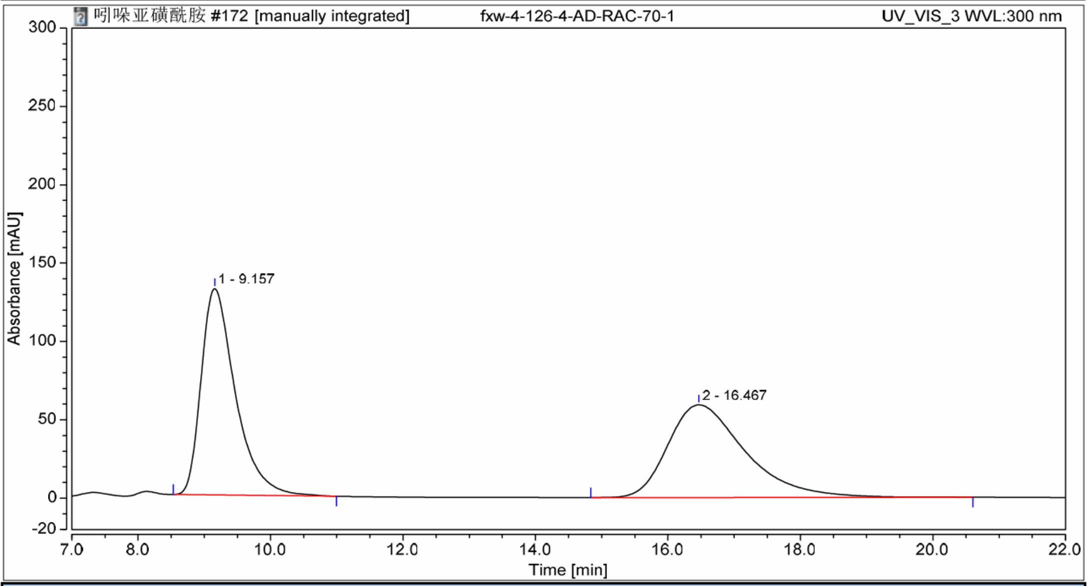

Integration Results

No.	Peak Name	Retention Time min	Area mAU*min	Height mAU	Relative Area %	Relative Height %	Amount n.a.
1		9.157	78.852	131.739	49.66	69.01	n.a.
2		16.467	79.924	59.149	50.34	30.99	n.a.
**Total:**			**158.776**	**190.888**	**100.00**	**100.00**	

Chromatogram

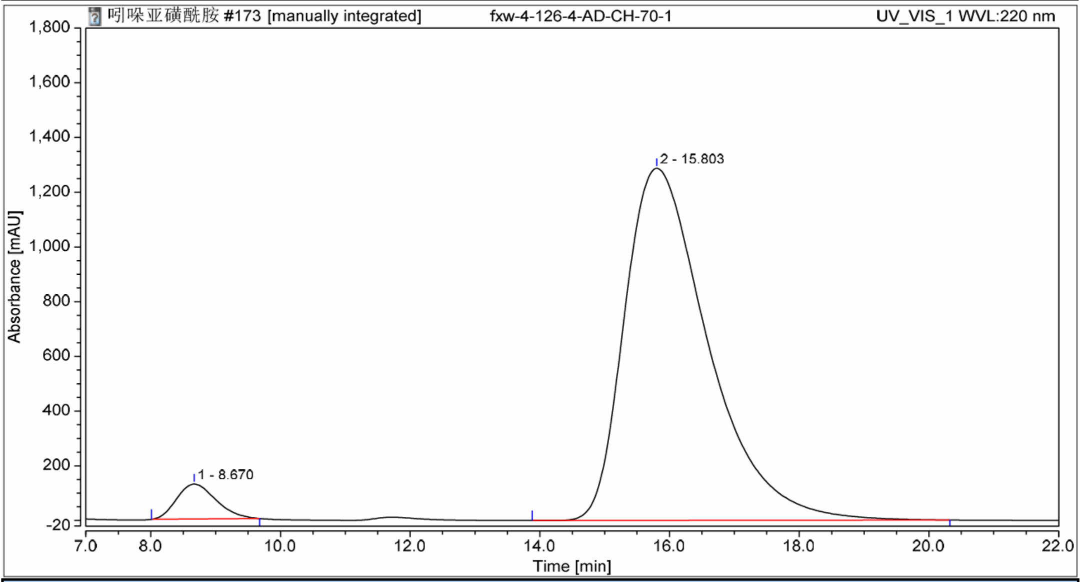

Integration Results

No.	Peak Name	Retention Time min	Area mAU*min	Height mAU	Relative Area %	Relative Height %	Amount n.a.
1		8.670	89.610	127.517	4.46	9.02	n.a.
2		15.803	1921.166	1286.445	95.54	90.98	n.a.
**Total:**			**2010.776**	**1413.962**	**100.00**	**100.00**	

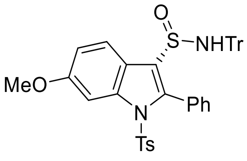

23b

Chromatogram

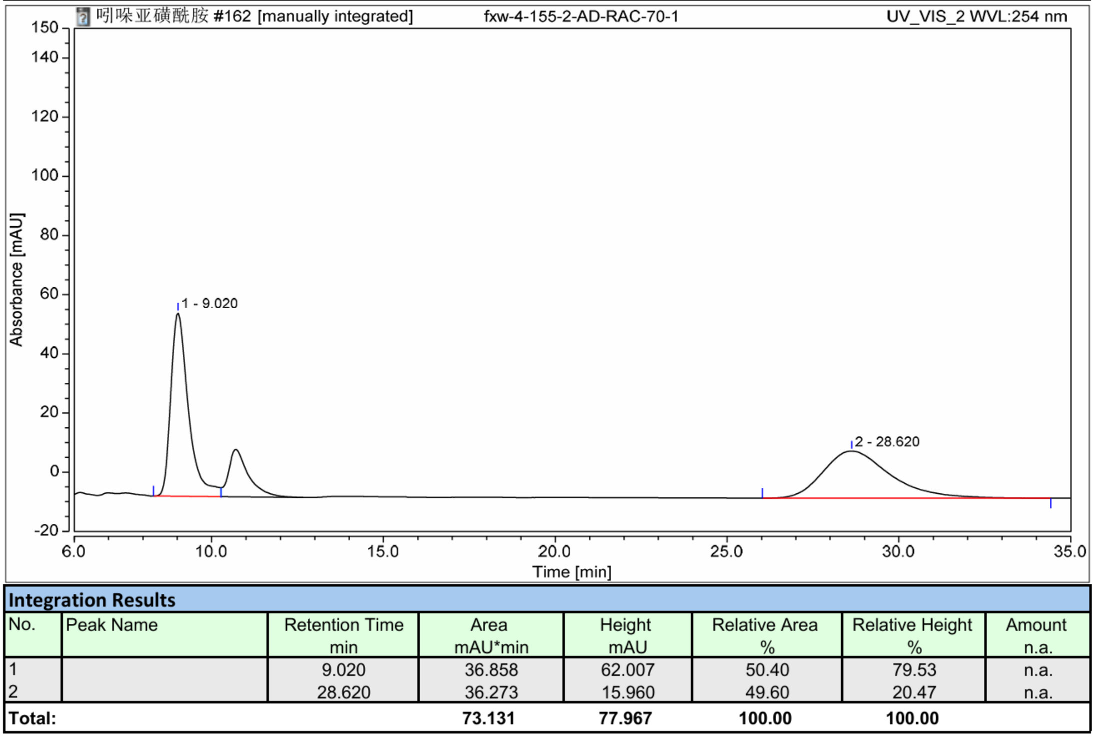

Integration Results

No.	Peak Name	Retention Time min	Area mAU*min	Height mAU	Relative Area %	Relative Height %	Amount n.a.
1		9.020	36.858	62.007	50.40	79.53	n.a.
2		28.620	36.273	15.960	49.60	20.47	n.a.
Total:			73.131	77.967	100.00	100.00	

Chromatogram

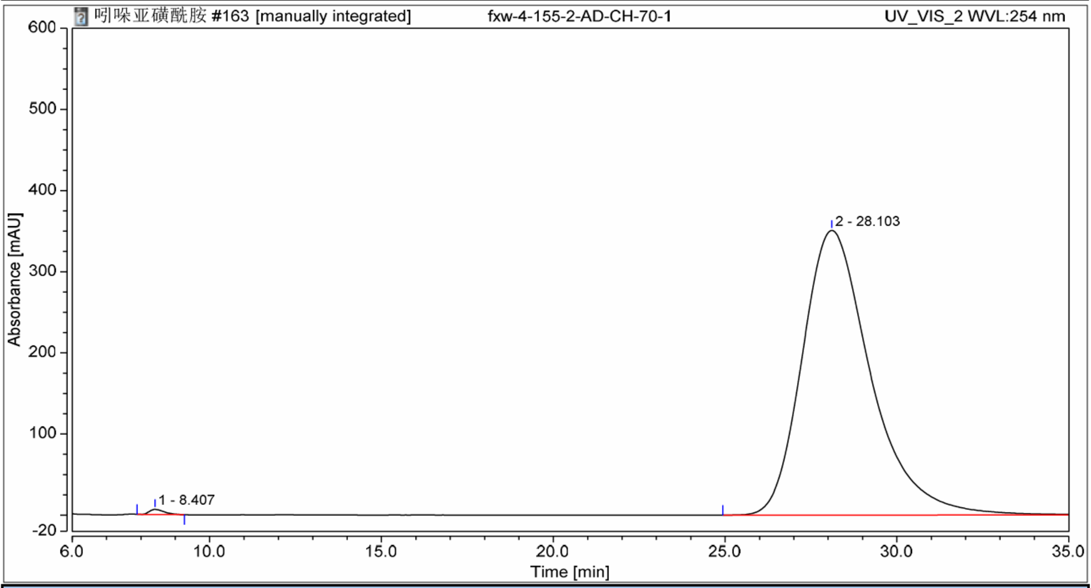

Integration Results

No.	Peak Name	Retention Time min	Area mAU*min	Height mAU	Relative Area %	Relative Height %	Amount n.a.
1		8.407	3.087	6.279	0.38	1.76	n.a.
2		28.103	810.846	350.772	99.62	98.24	n.a.
Total:			813.932	357.051	100.00	100.00	

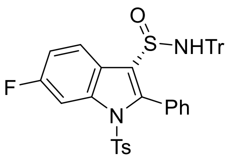

24b

Chromatogram

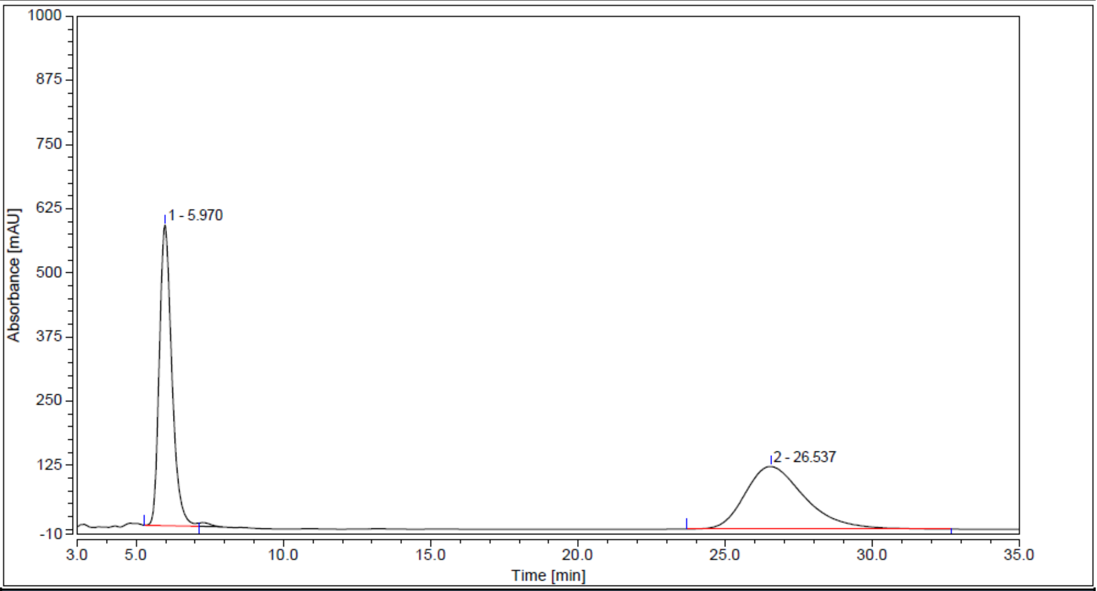

Integration Results

No.	Peak Name	Retention Time<br>min	Area<br>mAU*min	Height<br>mAU	Relative Area<br>%	Relative Height<br>%	Amount<br>n.a.
1		5.970	289.011	585.971	50.17	82.77	n.a.
2		26.537	287.029	122.008	49.83	17.23	n.a.
**Total:**			**576.040**	**707.979**	**100.00**	**100.00**	

Chromatogram

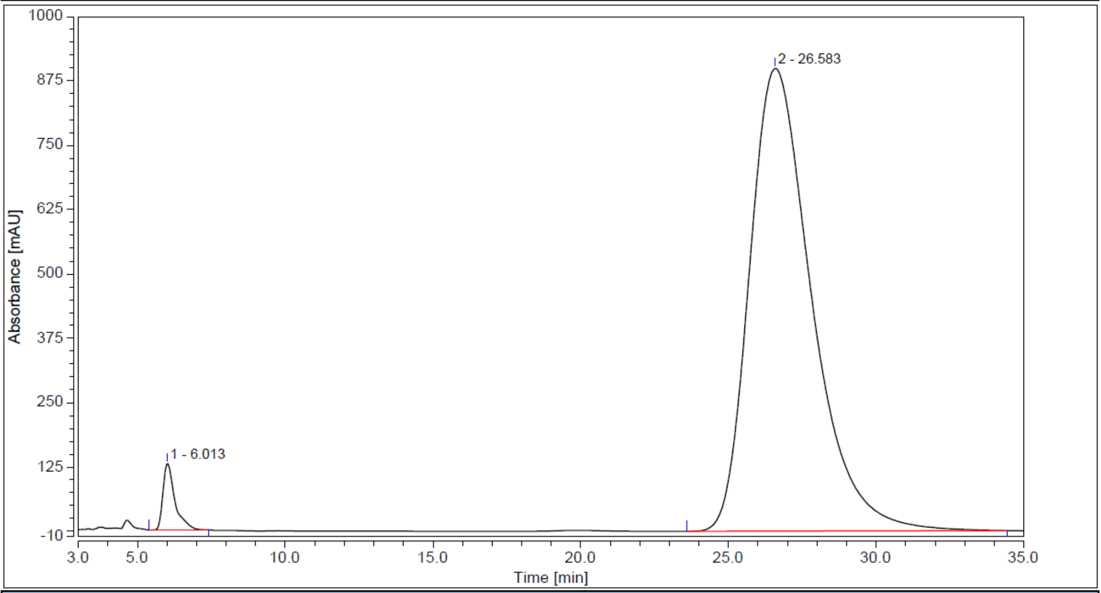

Integration Results

No.	Peak Name	Retention Time<br>min	Area<br>mAU*min	Height<br>mAU	Relative Area<br>%	Relative Height<br>%	Amount<br>n.a.
1		6.013	60.760	129.664	2.70	12.61	n.a.
2		26.583	2186.386	898.810	97.30	87.39	n.a.
**Total:**			**2247.146**	**1028.474**	**100.00**	**100.00**	

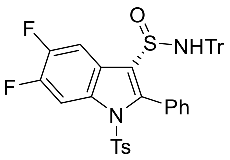

25b

Chromatogram

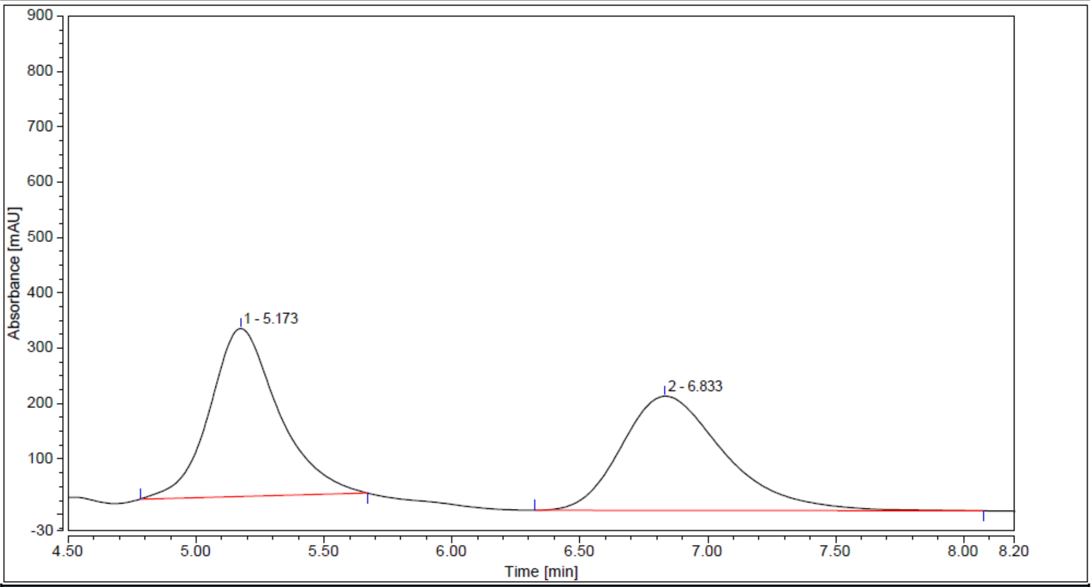

Integration Results

No.	Peak Name	Retention Time min	Area mAU*min	Height mAU	Relative Area %	Relative Height %	Amount n.a.
1		5.173	94.725	303.525	49.55	59.52	n.a.
2		6.833	96.459	206.422	50.45	40.48	n.a.
**Total:**			**191.184**	**509.947**	**100.00**	**100.00**	

Chromatogram

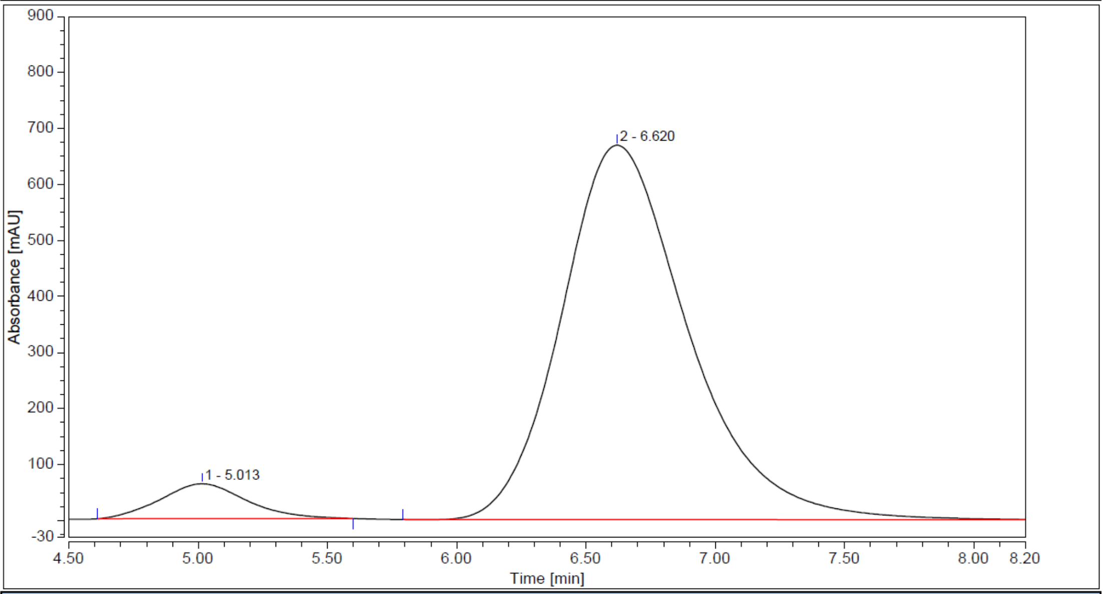

Integration Results

No.	Peak Name	Retention Time min	Area mAU*min	Height mAU	Relative Area %	Relative Height %	Amount n.a.
1		5.013	24.039	62.115	5.93	8.50	n.a.
2		6.620	381.006	668.532	94.07	91.50	n.a.
**Total:**			**405.045**	**730.647**	**100.00**	**100.00**	

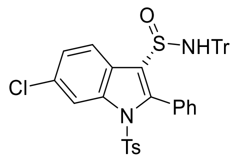

26b

Chromatogram

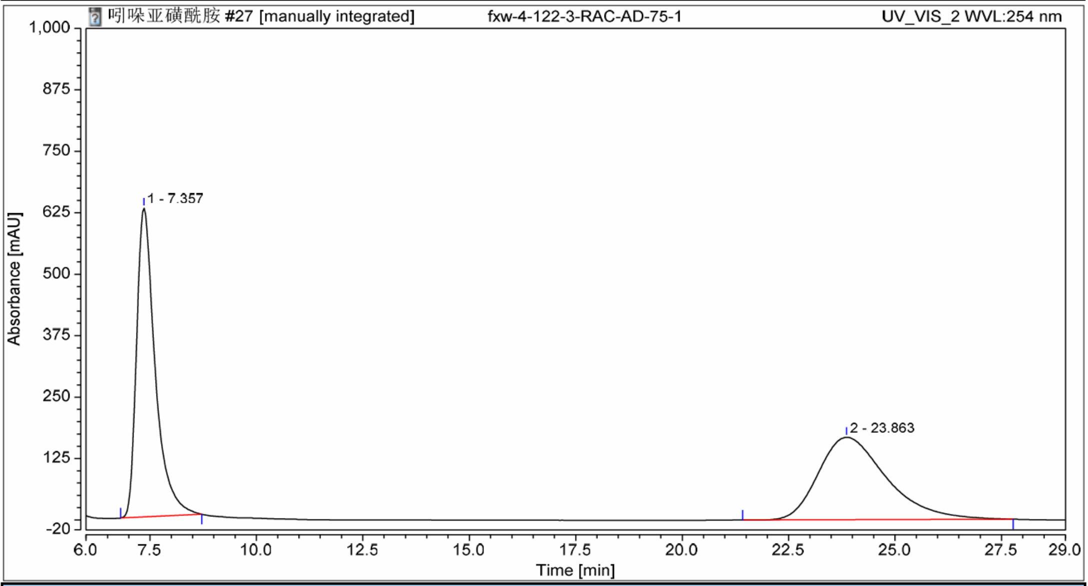

Integration Results

No.	Peak Name	Retention Time<br>min	Area<br>mAU*min	Height<br>mAU	Relative Area<br>%	Relative Height<br>%	Amount<br>n.a.
1		7.357	318.254	628.562	50.57	78.95	n.a.
2		23.863	311.047	167.620	49.43	21.05	n.a.
**Total:**			**629.301**	**796.182**	**100.00**	**100.00**	

Chromatogram

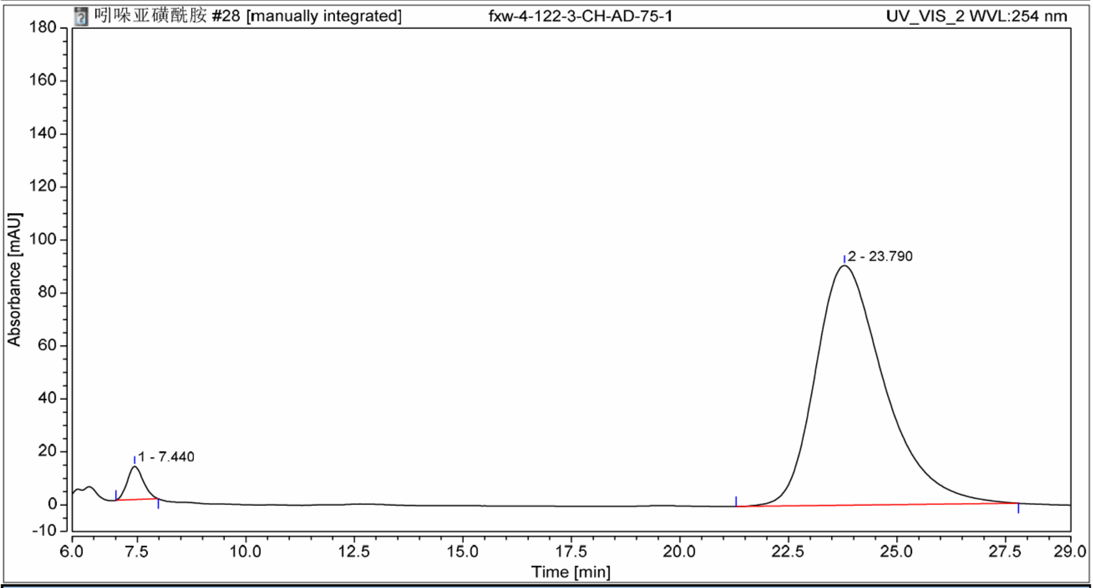

Integration Results

No.	Peak Name	Retention Time<br>min	Area<br>mAU*min	Height<br>mAU	Relative Area<br>%	Relative Height<br>%	Amount<br>n.a.
1		7.440	5.346	12.542	3.10	12.17	n.a.
2		23.790	166.898	90.488	96.90	87.83	n.a.
**Total:**			**172.244**	**103.029**	**100.00**	**100.00**	

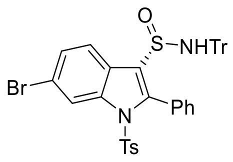

27b

Chromatogram

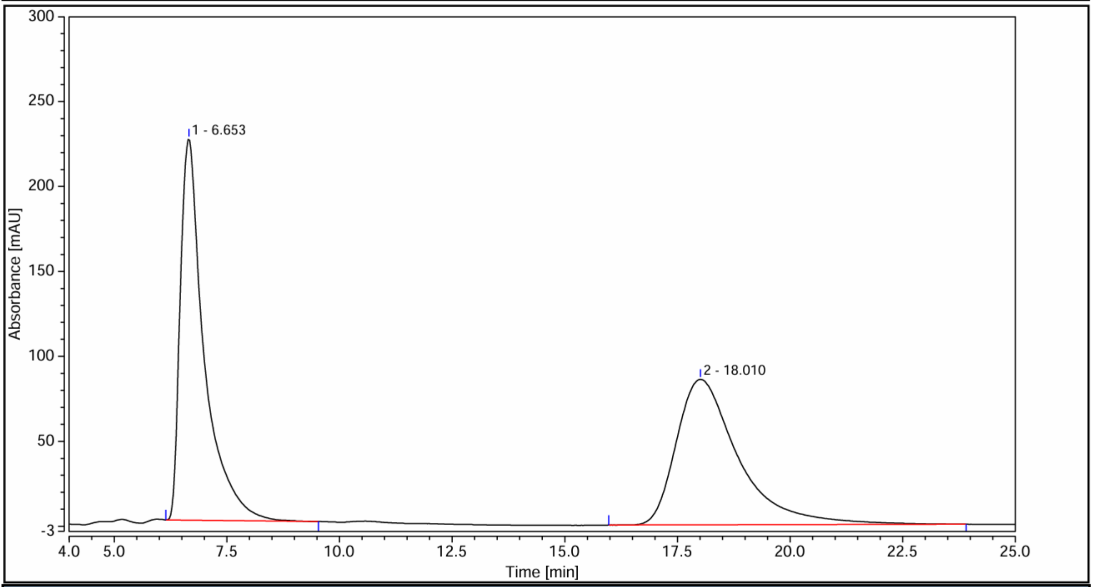

Integration Results

No.	Peak Name	Retention Time min	Area mAU*min	Height mAU	Relative Area %	Relative Height %	Amount n.a.
1		6.653	138.318	224.558	50.18	72.38	n.a.
2		18.010	137.340	85.703	49.82	27.62	n.a.
**Total:**			**275.658**	**310.261**	**100.00**	**100.00**	

Chromatogram

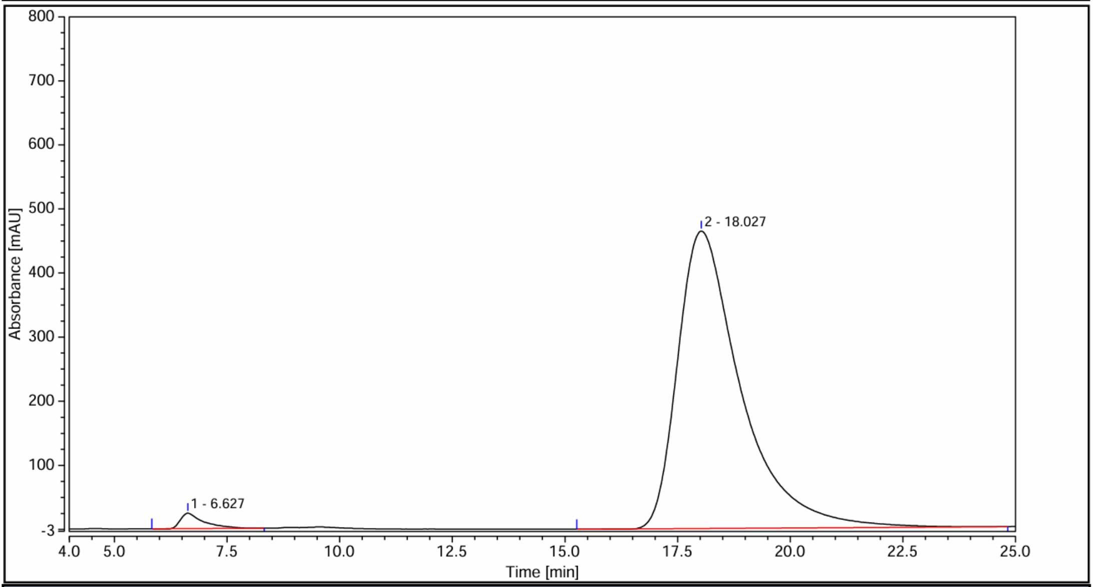

Integration Results

No.	Peak Name	Retention Time min	Area mAU*min	Height mAU	Relative Area %	Relative Height %	Amount n.a.
1		6.627	14.001	24.007	1.82	4.92	n.a.
2		18.027	754.988	464.184	98.18	95.08	n.a.
**Total:**			**768.989**	**488.191**	**100.00**	**100.00**	

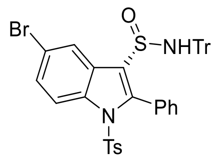

28b

Chromatogram

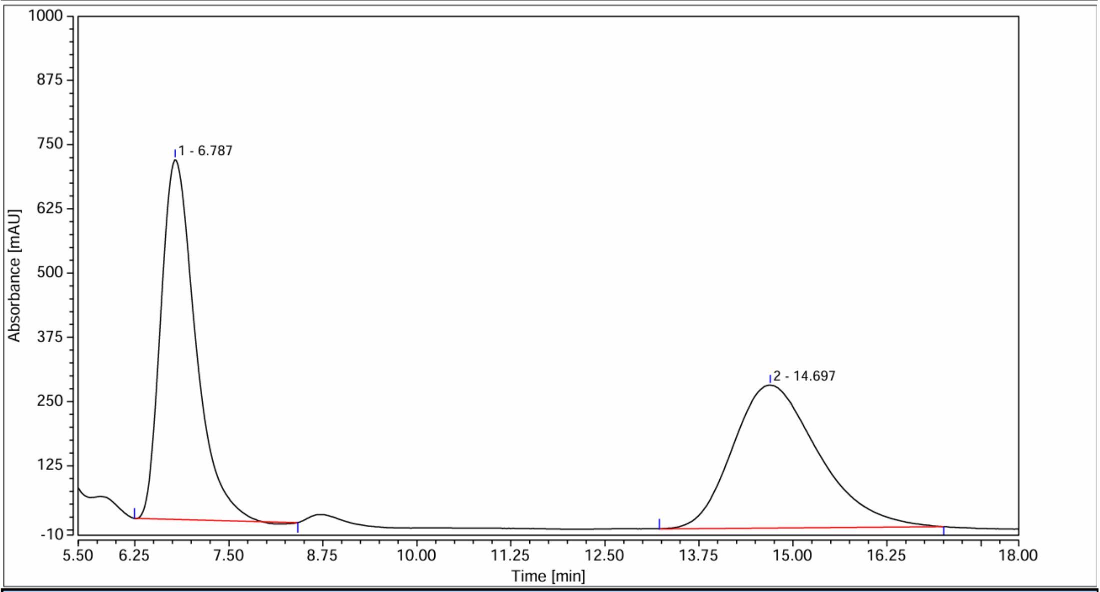

Integration Results

No.	Peak Name	Retention Time min	Area mAU*min	Height mAU	Relative Area %	Relative Height %	Amount n.a.
1		6.787	372.979	700.254	50.33	71.57	n.a.
2		14.697	368.059	278.180	49.67	28.43	n.a.
**Total:**			**741.038**	**978.434**	**100.00**	**100.00**	

Chromatogram

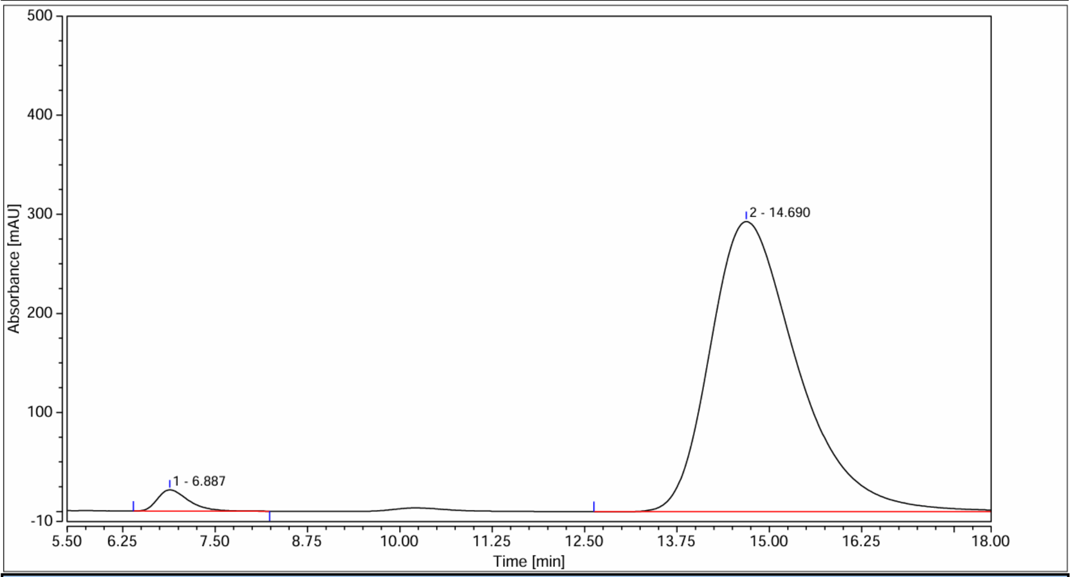

Integration Results

No.	Peak Name	Retention Time min	Area mAU*min	Height mAU	Relative Area %	Relative Height %	Amount n.a.
1		6.887	10.444	21.426	2.54	6.82	n.a.
2		14.690	400.846	292.586	97.46	93.18	n.a.
**Total:**			**411.290**	**314.012**	**100.00**	**100.00**	

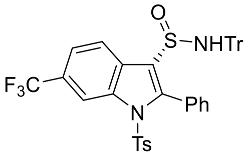

29b

Chromatogram

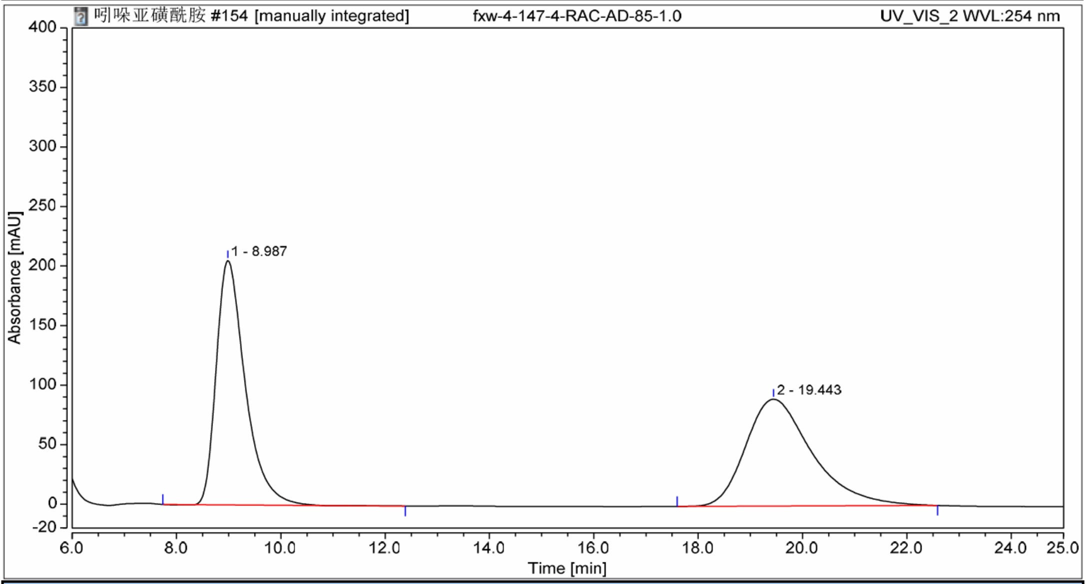

Integration Results

No.	Peak Name	Retention Time min	Area mAU*min	Height mAU	Relative Area %	Relative Height %	Amount n.a.
1		8.987	131.793	205.263	49.93	69.59	n.a.
2		19.443	132.183	89.700	50.07	30.41	n.a.
**Total:**			**263.976**	**294.963**	**100.00**	**100.00**	

Chromatogram

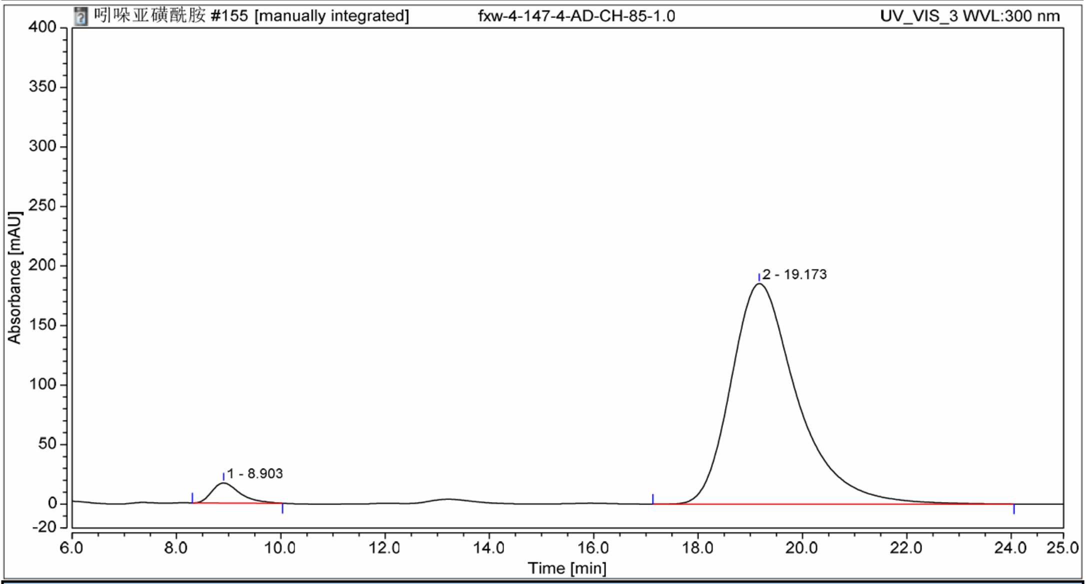

Integration Results

No.	Peak Name	Retention Time min	Area mAU*min	Height mAU	Relative Area %	Relative Height %	Amount n.a.
1		8.903	10.494	16.837	3.71	8.33	n.a.
2		19.173	272.466	185.293	96.29	91.67	n.a.
**Total:**			**282.960**	**202.129**	**100.00**	**100.00**	

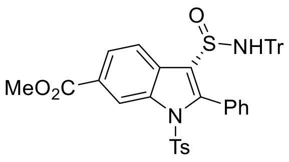

30b

Chromatogram

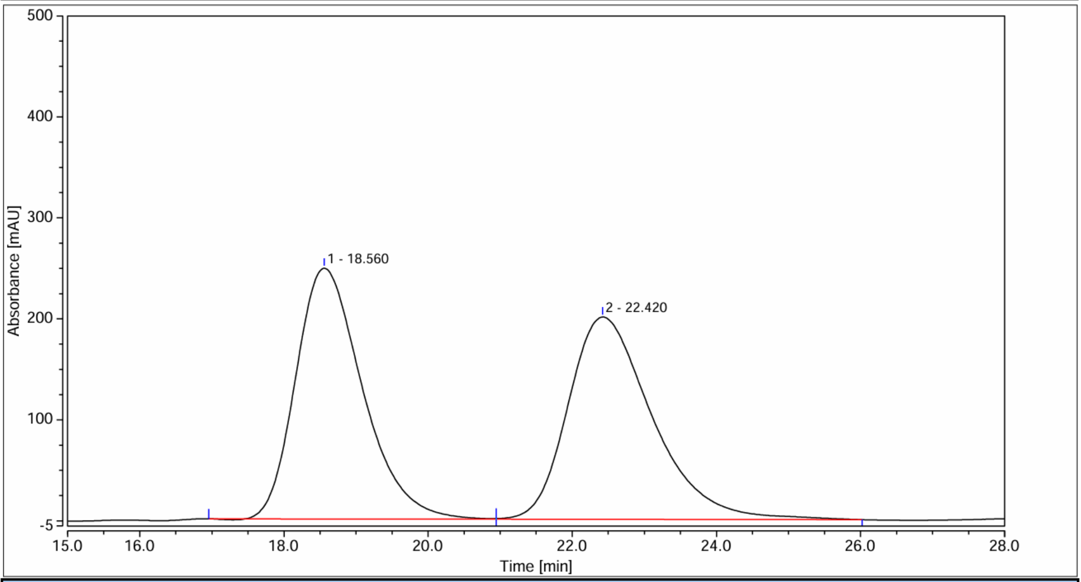

Integration Results

No.	Peak Name	Retention Time min	Area mAU*min	Height mAU	Relative Area %	Relative Height %	Amount n.a.
1		18.560	266.503	248.398	49.54	55.35	n.a.
2		22.420	271.417	200.401	50.46	44.65	n.a.
**Total:**			**537.919**	**448.799**	**100.00**	**100.00**	

Chromatogram

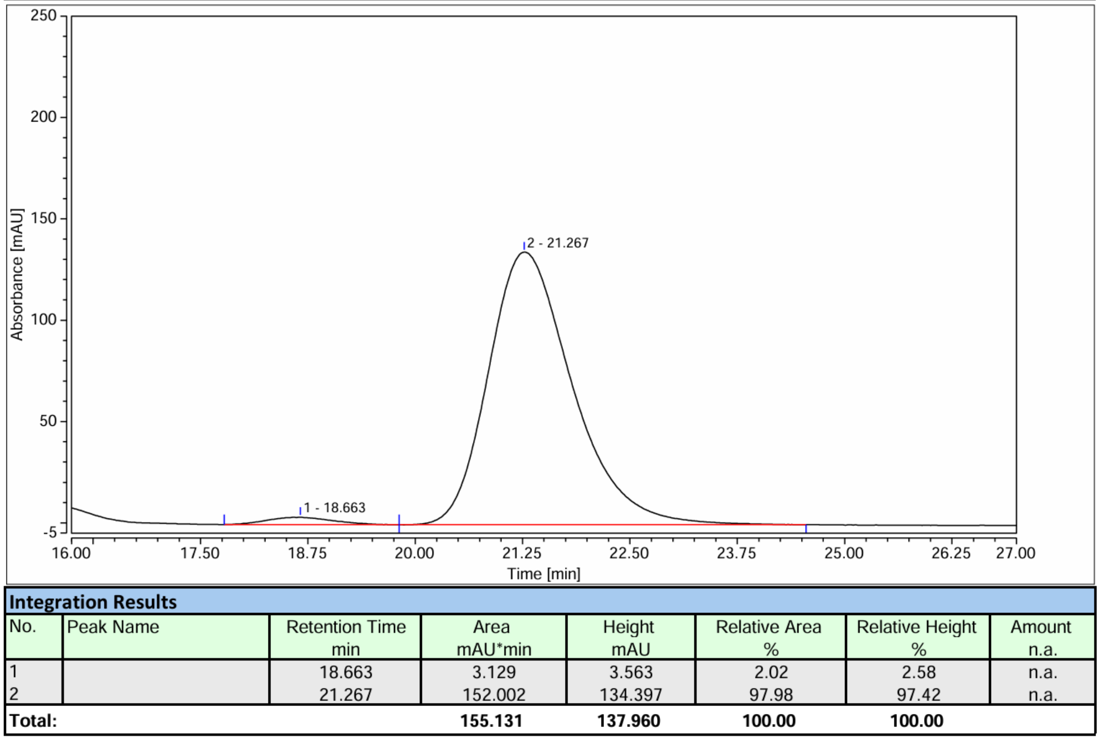

Integration Results

No.	Peak Name	Retention Time min	Area mAU*min	Height mAU	Relative Area %	Relative Height %	Amount n.a.
1		18.663	3.129	3.563	2.02	2.58	n.a.
2		21.267	152.002	134.397	97.98	97.42	n.a.
**Total:**			**155.131**	**137.960**	**100.00**	**100.00**	

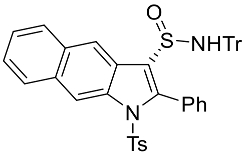

31b

Chromatogram

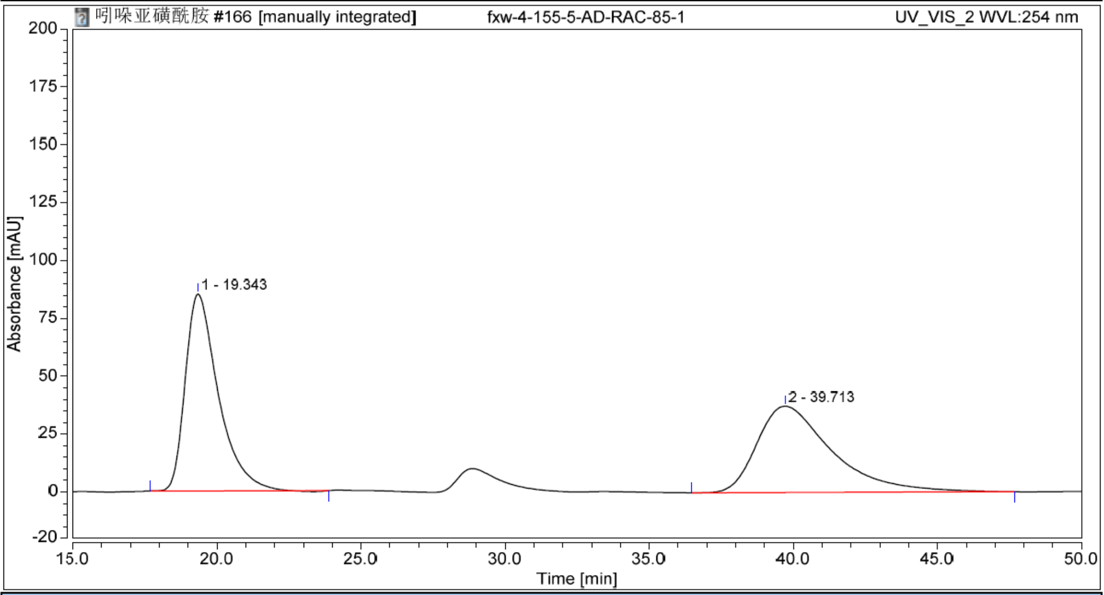

Integration Results

No.	Peak Name	Retention Time min	Area mAU*min	Height mAU	Relative Area %	Relative Height %	Amount n.a.
1		19.343	112.735	85.070	50.15	69.54	n.a.
2		39.713	112.041	37.269	49.85	30.46	n.a.
**Total:**			**224.776**	**122.339**	**100.00**	**100.00**	

Chromatogram

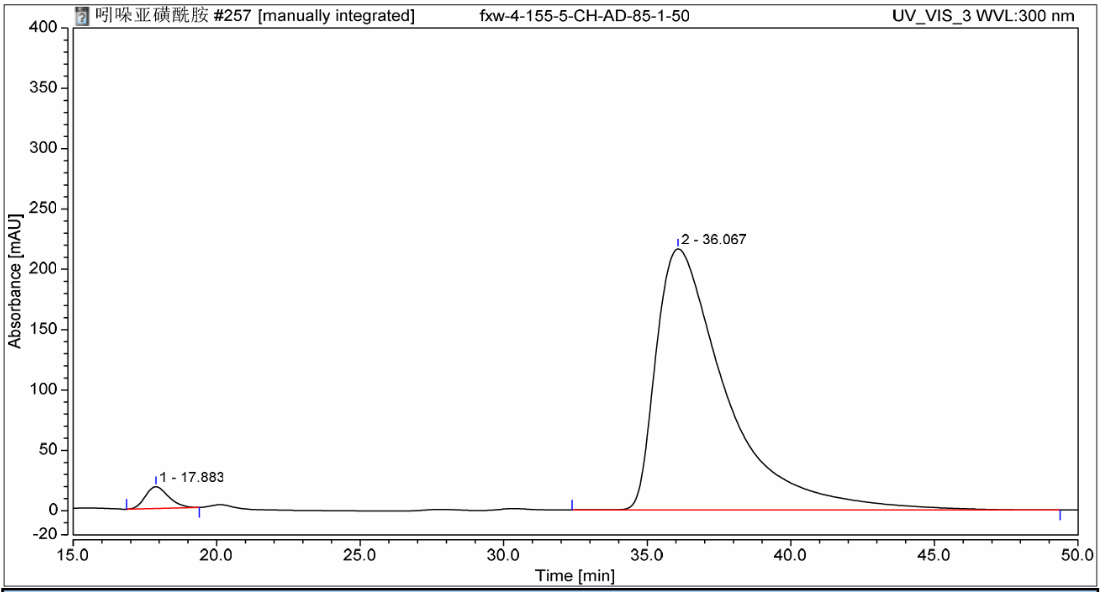

Integration Results

No.	Peak Name	Retention Time min	Area mAU*min	Height mAU	Relative Area %	Relative Height %	Amount n.a.
1		17.883	16.905	18.038	2.68	7.70	n.a.
2		36.067	614.744	216.284	97.32	92.30	n.a.
**Total:**			**631.649**	**234.322**	**100.00**	**100.00**	

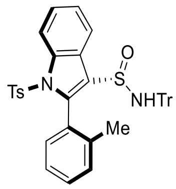

32b

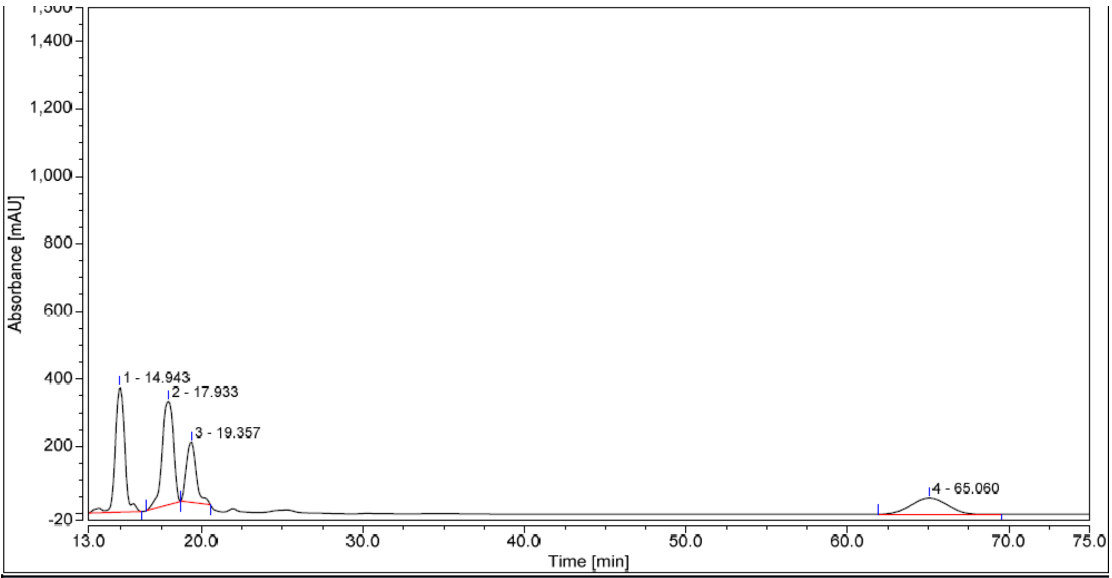

Integration Results

No.	Peak Name	Retention Time min	Area mAU*min	Height mAU	Relative Area %	Relative Height %	Amount n.a.
1		14.943	254.676	370.729	33.65	41.02	n.a.
2		17.933	250.257	306.240	33.07	33.88	n.a.
3		19.357	127.636	178.859	16.86	19.79	n.a.
4		65.060	124.276	48.020	16.42	5.31	n.a.
**Total:**			**756.845**	**903.849**	**100.00**	**100.00**	

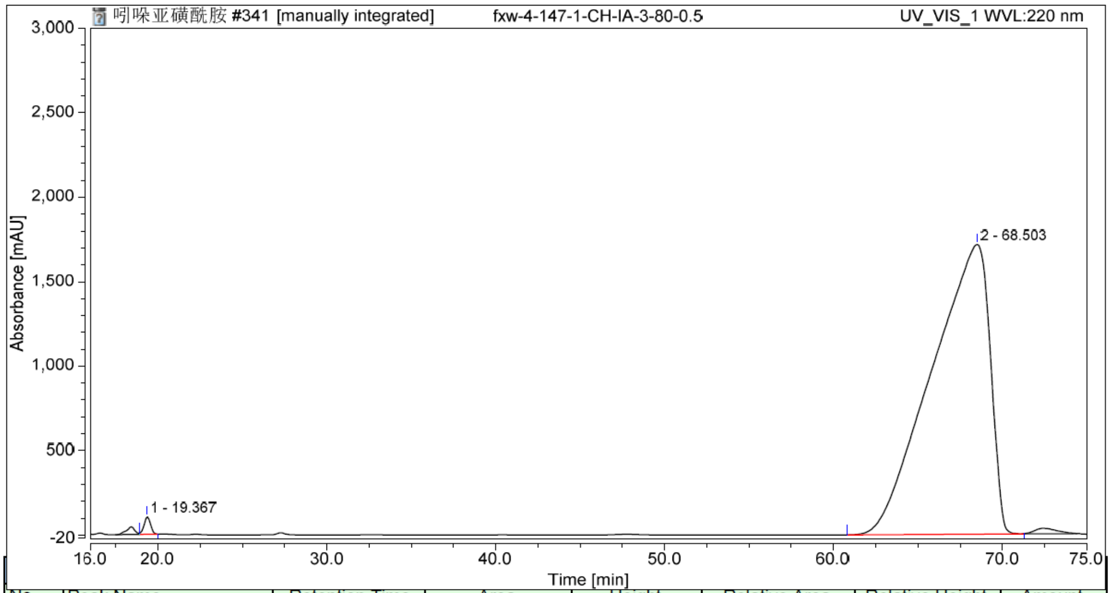

No.	Peak Name	Retention Time min	Area mAU*min	Height mAU	Relative Area %	Relative Height %	Amount n.a.
1		19.367	45.270	102.837	0.65	5.65	n.a.
2		68.503	6869.714	1716.125	99.35	94.35	n.a.
**Total:**			**6914.984**	**1818.962**	**100.00**	**100.00**	

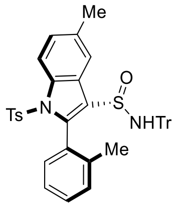

33b

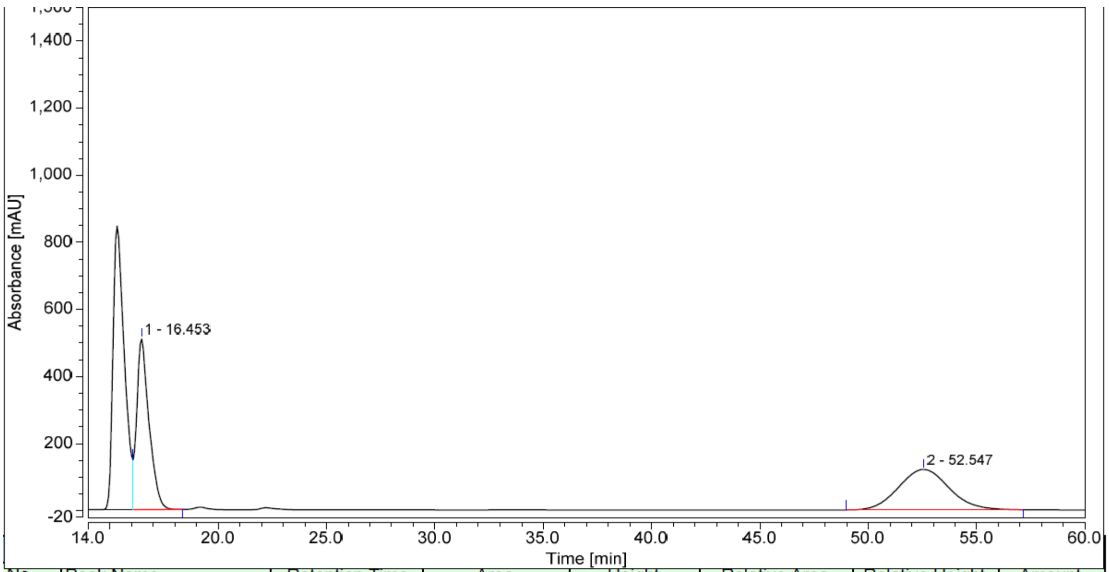

No.	Peak Name	Retention Time<br>min	Area<br>mAU*min	Height<br>mAU	Relative Area<br>%	Relative Height<br>%	Amount<br>n.a.
1		16.453	337.108	508.927	50.72	80.82	n.a.
2		52.547	327.601	120.816	49.28	19.18	n.a.
**Total:**			**664.709**	**629.743**	**100.00**	**100.00**	

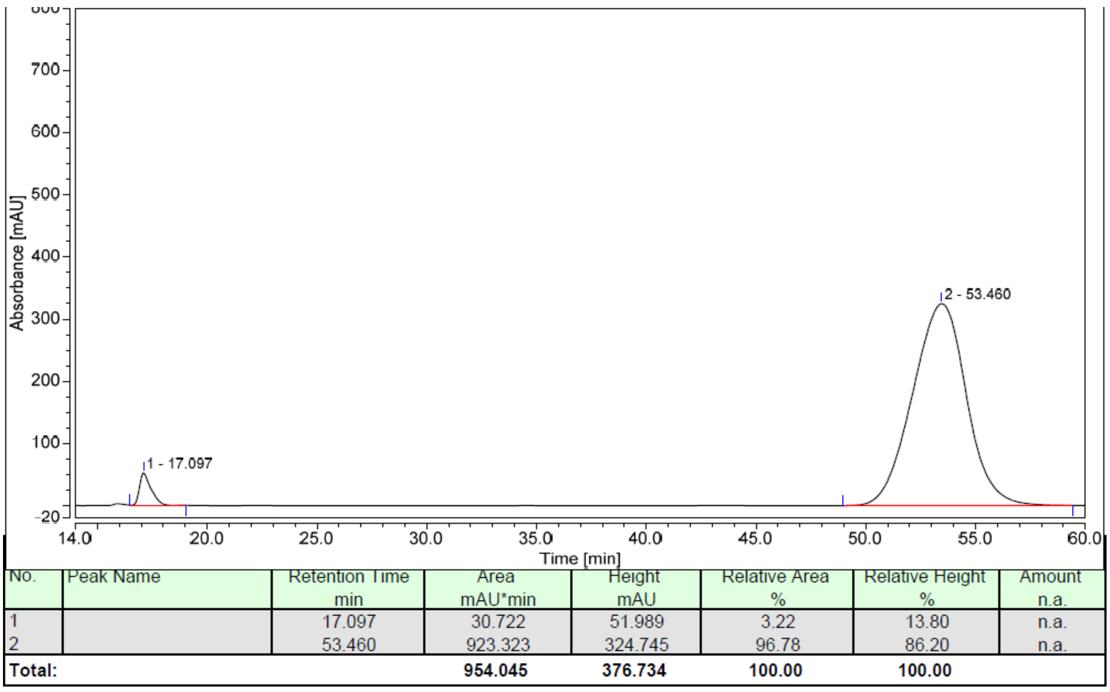

No.	Peak Name	Retention Time<br>min	Area<br>mAU*min	Height<br>mAU	Relative Area<br>%	Relative Height<br>%	Amount<br>n.a.
1		17.097	30.722	51.989	3.22	13.80	n.a.
2		53.460	923.323	324.745	96.78	86.20	n.a.
**Total:**			**954.045**	**376.734**	**100.00**	**100.00**	

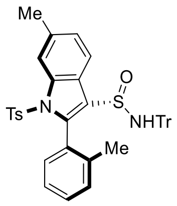

34b

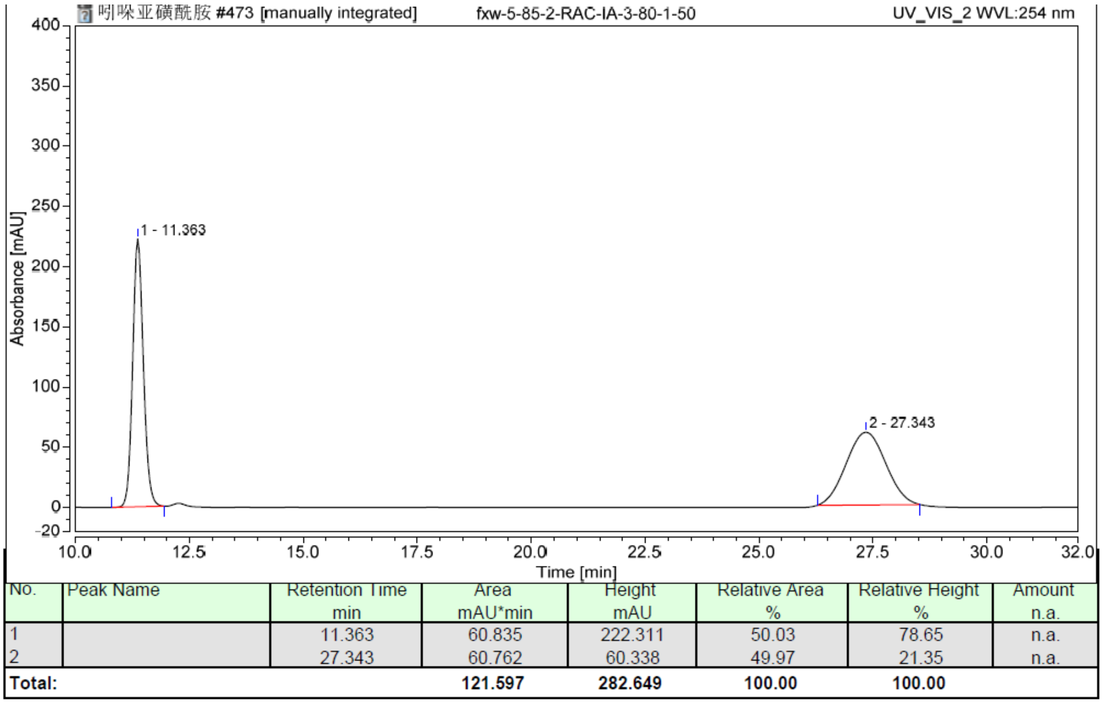

No.	Peak Name	Retention Time min	Area mAU*min	Height mAU	Relative Area %	Relative Height %	Amount n.a.
1		11.363	60.835	222.311	50.03	78.65	n.a.
2		27.343	60.762	60.338	49.97	21.35	n.a.
**Total:**			**121.597**	**282.649**	**100.00**	**100.00**	

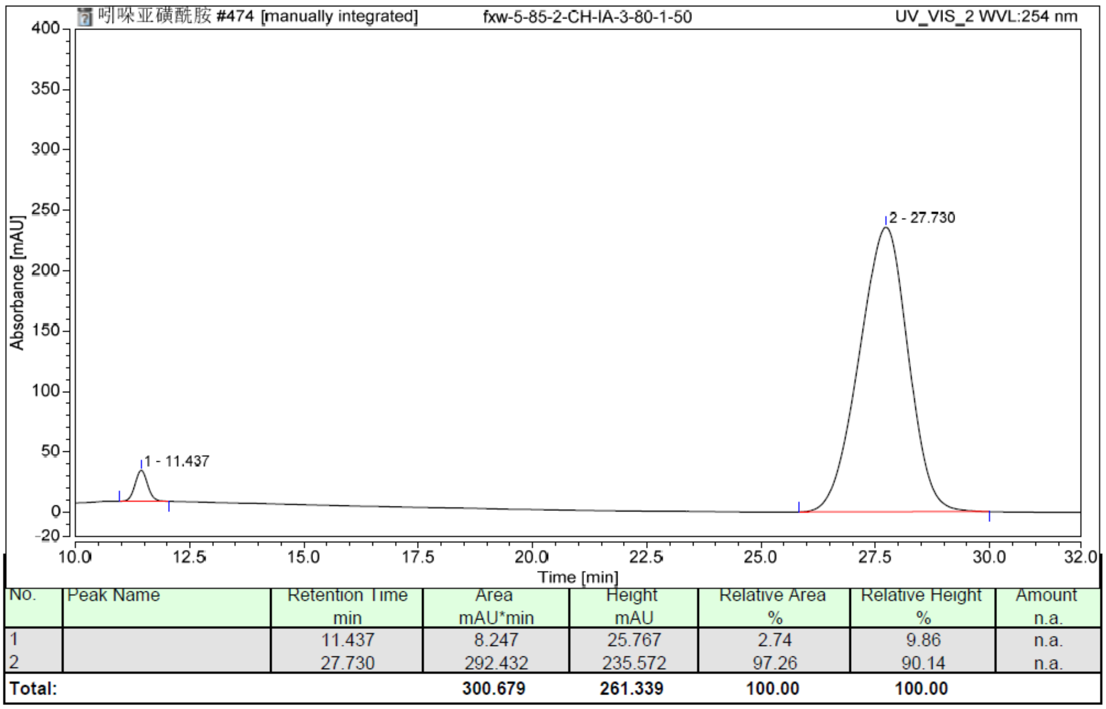

No.	Peak Name	Retention Time min	Area mAU*min	Height mAU	Relative Area %	Relative Height %	Amount n.a.
1		11.437	8.247	25.767	2.74	9.86	n.a.
2		27.730	292.432	235.572	97.26	90.14	n.a.
**Total:**			**300.679**	**261.339**	**100.00**	**100.00**	

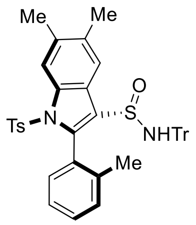

35b

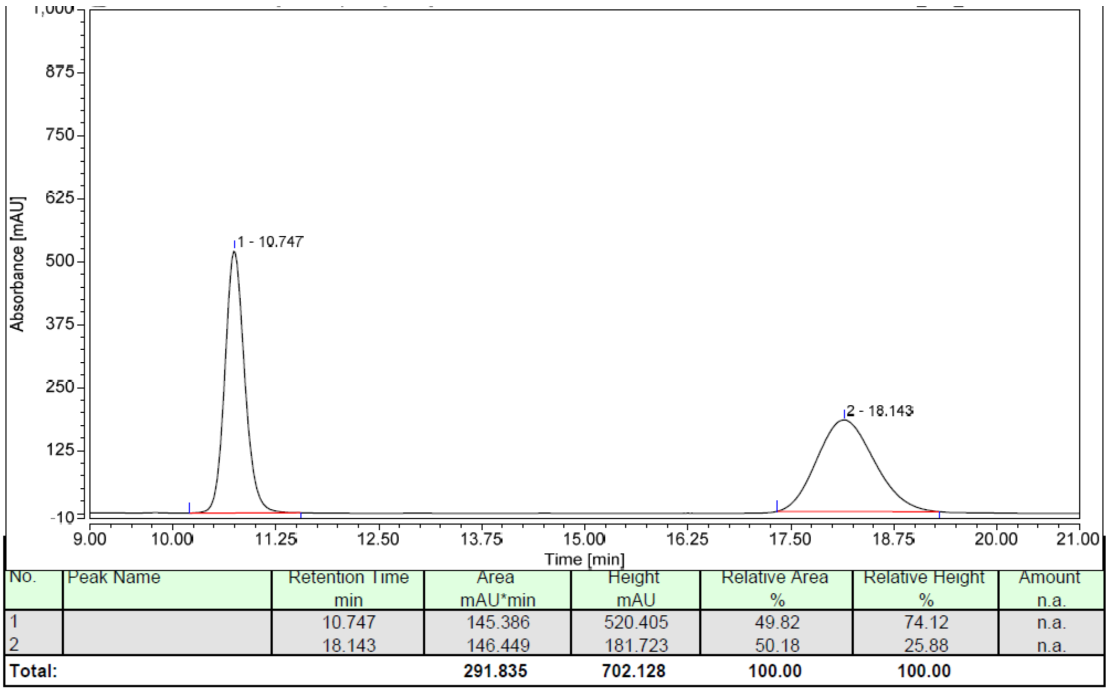

No.	Peak Name	Retention Time min	Area mAU*min	Height mAU	Relative Area %	Relative Height %	Amount n.a.
1		10.747	145.386	520.405	49.82	74.12	n.a.
2		18.143	146.449	181.723	50.18	25.88	n.a.
**Total:**			**291.835**	**702.128**	**100.00**	**100.00**	

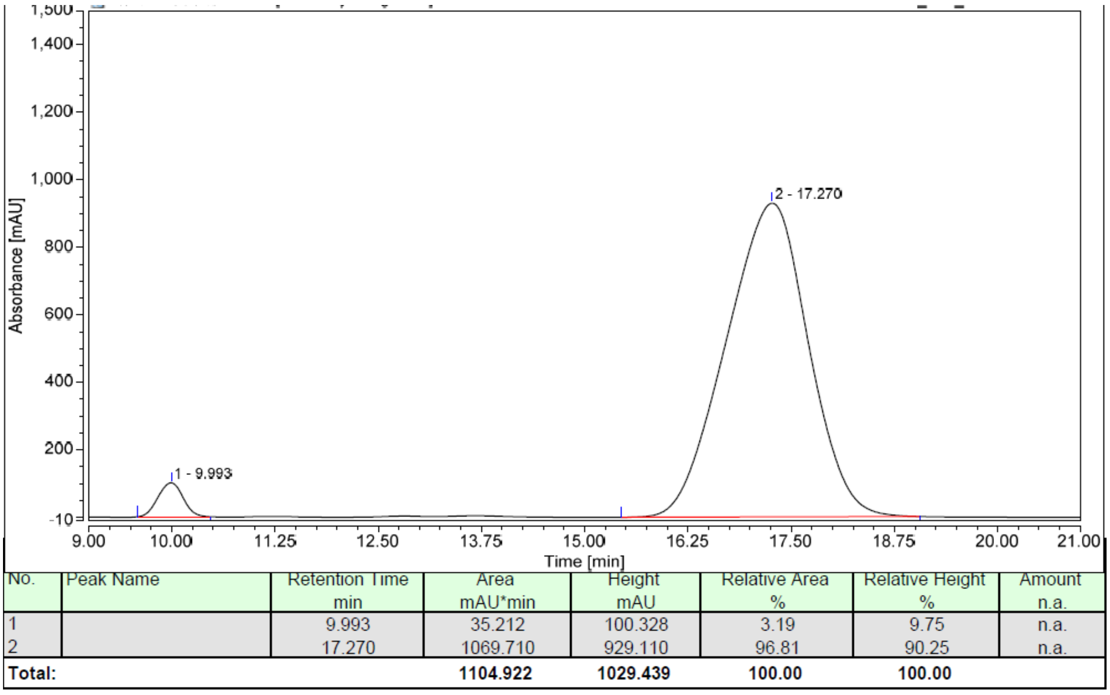

No.	Peak Name	Retention Time min	Area mAU*min	Height mAU	Relative Area %	Relative Height %	Amount n.a.
1		9.993	35.212	100.328	3.19	9.75	n.a.
2		17.270	1069.710	929.110	96.81	90.25	n.a.
**Total:**			**1104.922**	**1029.439**	**100.00**	**100.00**	

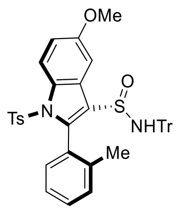

36b

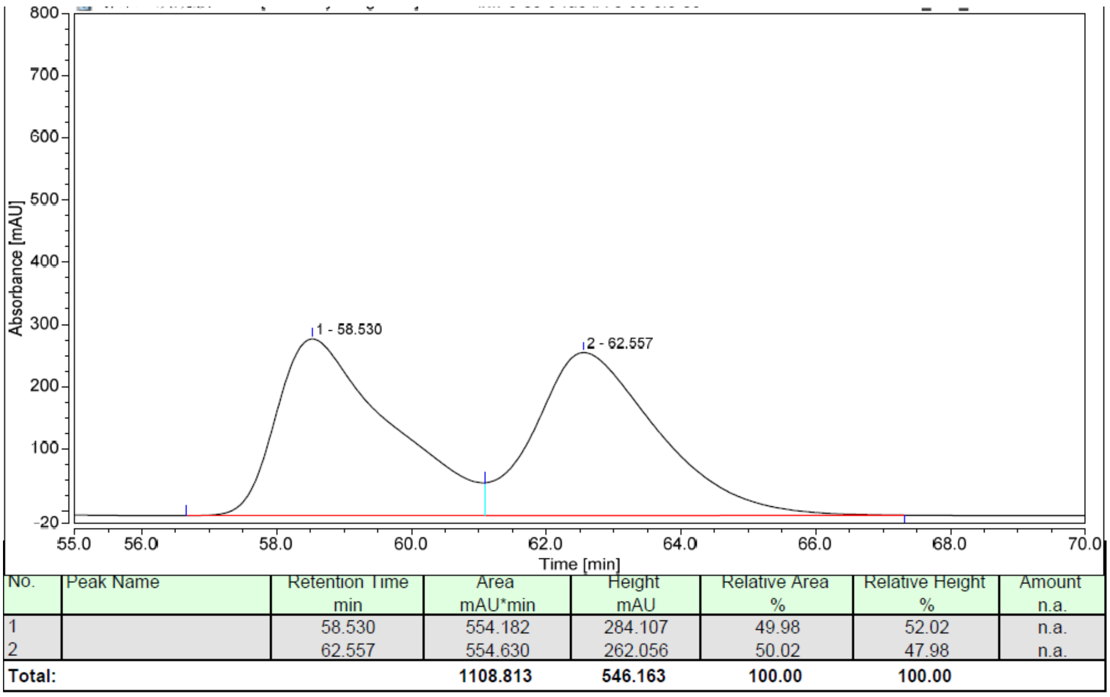

No.	Peak Name	Retention Time min	Area mAU*min	Height mAU	Relative Area %	Relative Height %	Amount n.a.
1		58.530	554.182	284.107	49.98	52.02	n.a.
2		62.557	554.630	262.056	50.02	47.98	n.a.
**Total:**			**1108.813**	**546.163**	**100.00**	**100.00**	

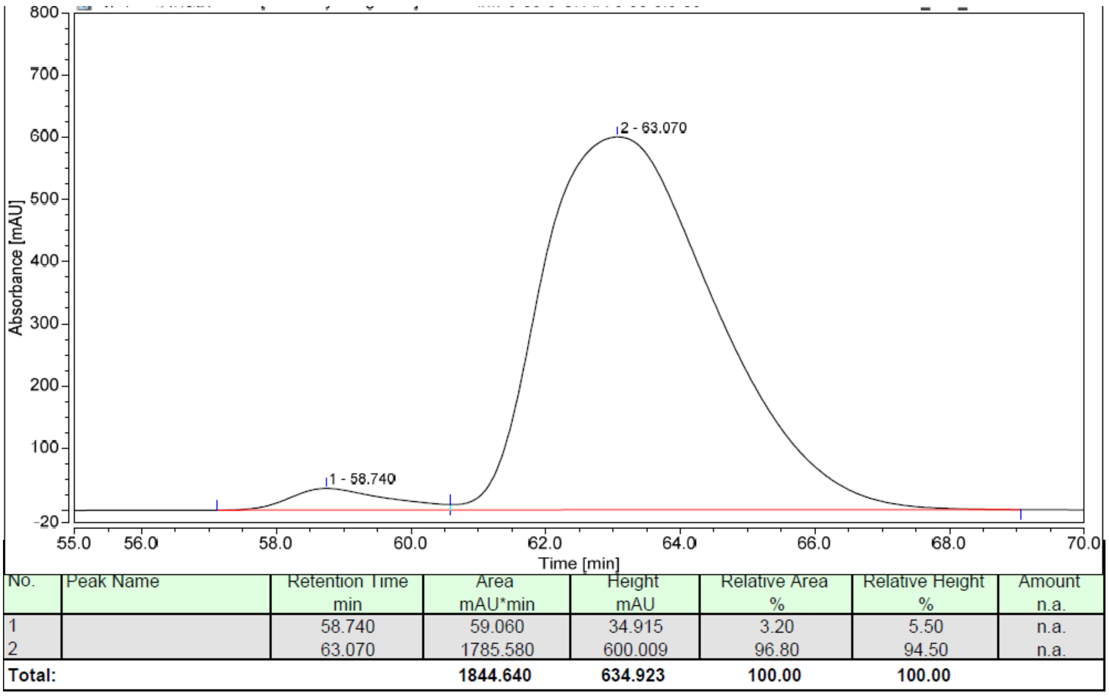

No.	Peak Name	Retention Time min	Area mAU*min	Height mAU	Relative Area %	Relative Height %	Amount n.a.
1		58.740	59.060	34.915	3.20	5.50	n.a.
2		63.070	1785.580	600.009	96.80	94.50	n.a.
**Total:**			**1844.640**	**634.923**	**100.00**	**100.00**	

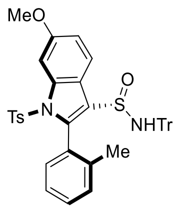

37b

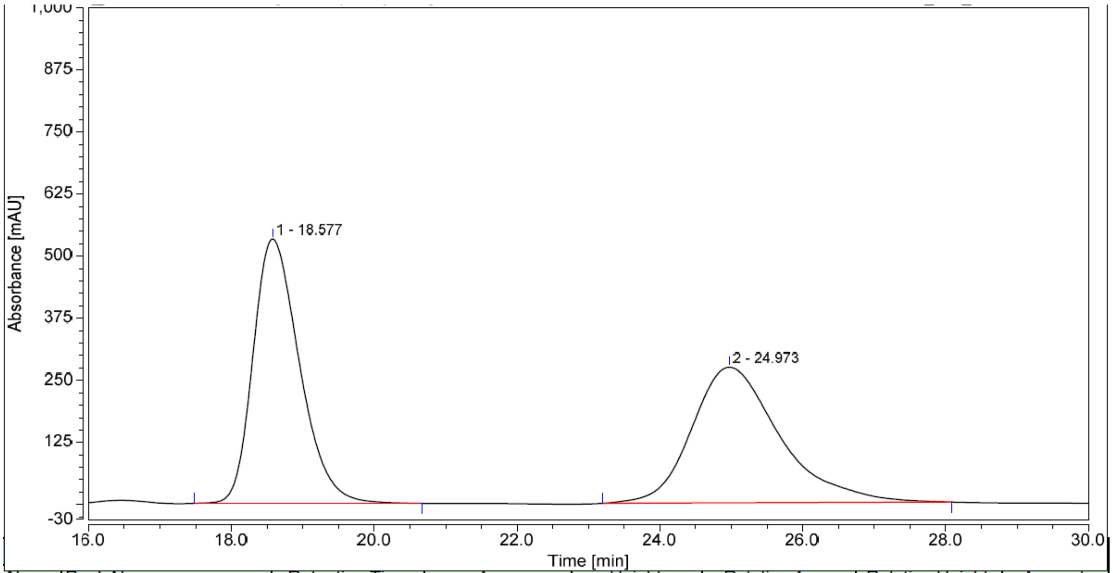

No.	Peak Name	Retention Time min	Area mAU*min	Height mAU	Relative Area %	Relative Height %	Amount n.a.
1		18.577	400.496	532.380	50.65	66.09	n.a.
2		24.973	390.254	273.205	49.35	33.91	n.a.
**Total:**			**790.750**	**805.585**	**100.00**	**100.00**	

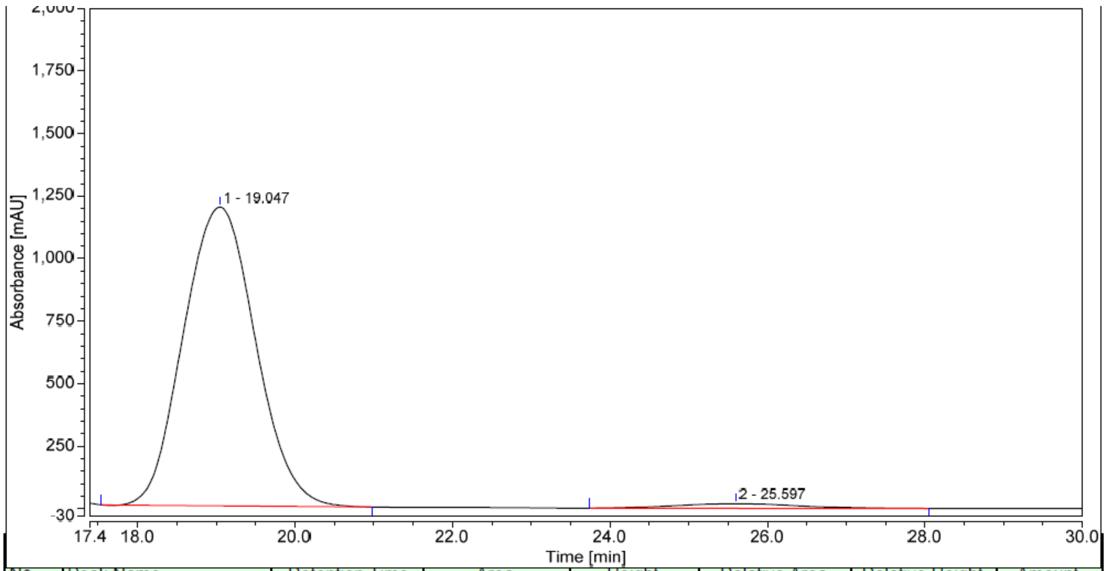

No.	Peak Name	Retention Time min	Area mAU*min	Height mAU	Relative Area %	Relative Height %	Amount n.a.
1		19.047	1265.096	1194.774	97.46	98.47	n.a.
2		25.597	33.028	18.581	2.54	1.53	n.a.
**Total:**			**1298.124**	**1213.356**	**100.00**	**100.00**	

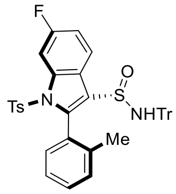

38b

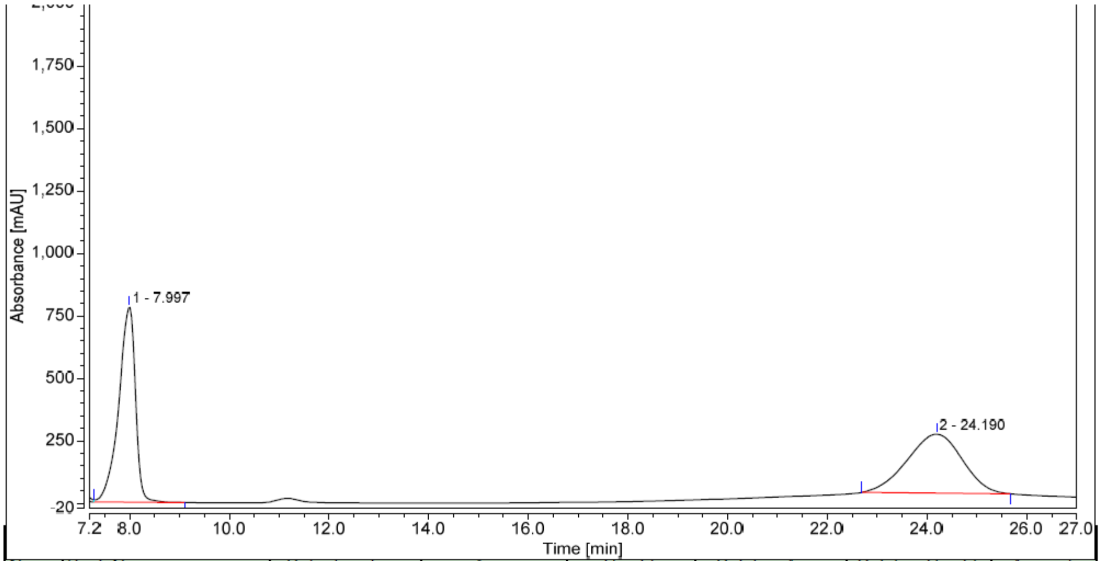

No.	Peak Name	Retention Time min	Area mAU*min	Height mAU	Relative Area %	Relative Height %	Amount n.a.
1		7.997	304.116	781.876	50.17	76.81	n.a.
2		24.190	302.009	235.993	49.83	23.19	n.a.
**Total:**			**606.125**	**1017.869**	**100.00**	**100.00**	

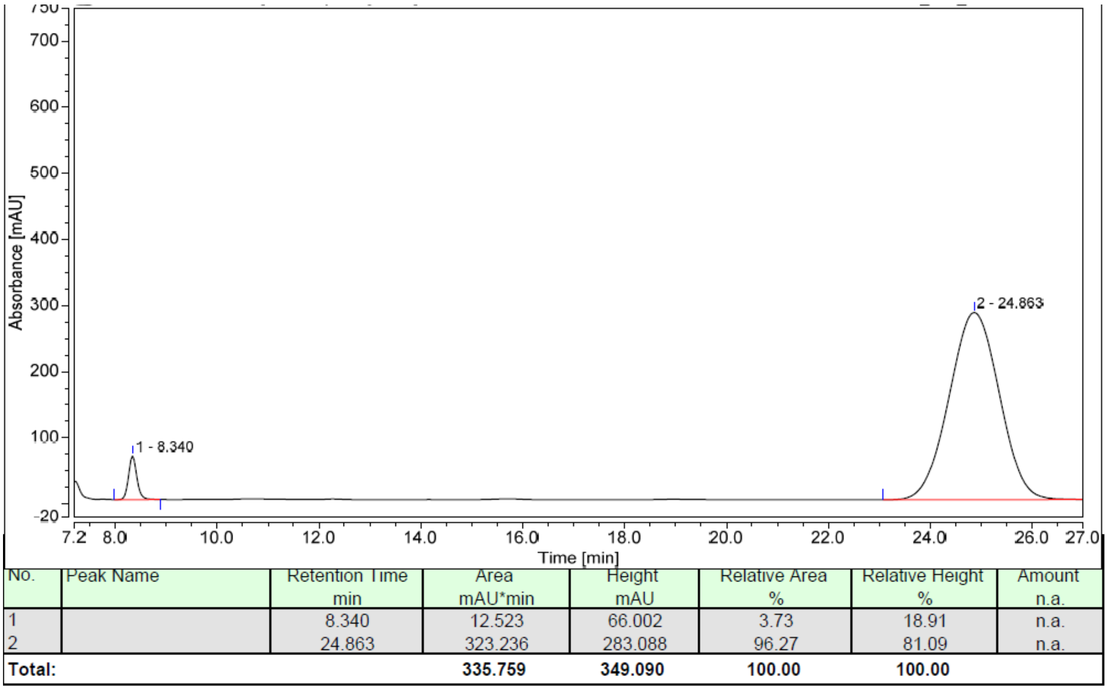

No.	Peak Name	Retention Time min	Area mAU*min	Height mAU	Relative Area %	Relative Height %	Amount n.a.
1		8.340	12.523	66.002	3.73	18.91	n.a.
2		24.863	323.236	283.088	96.27	81.09	n.a.
**Total:**			**335.759**	**349.090**	**100.00**	**100.00**	

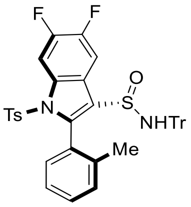

39b

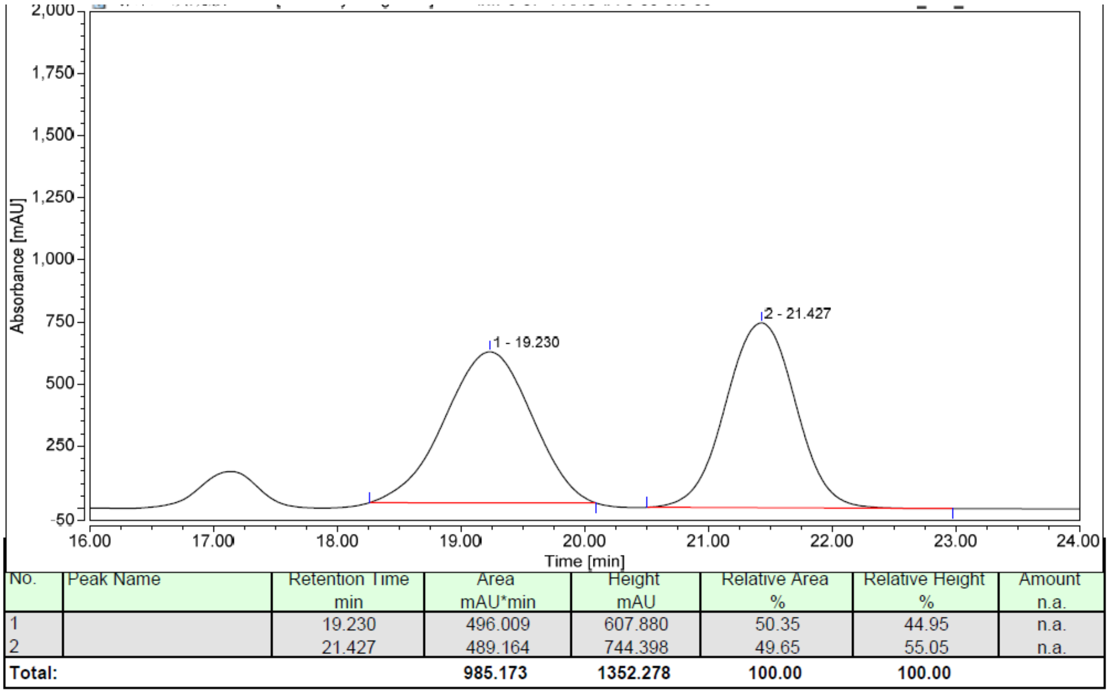

No.	Peak Name	Retention Time min	Area mAU*min	Height mAU	Relative Area %	Relative Height %	Amount n.a.
1		19.230	496.009	607.880	50.35	44.95	n.a.
2		21.427	489.164	744.398	49.65	55.05	n.a.
**Total:**			**985.173**	**1352.278**	**100.00**	**100.00**	

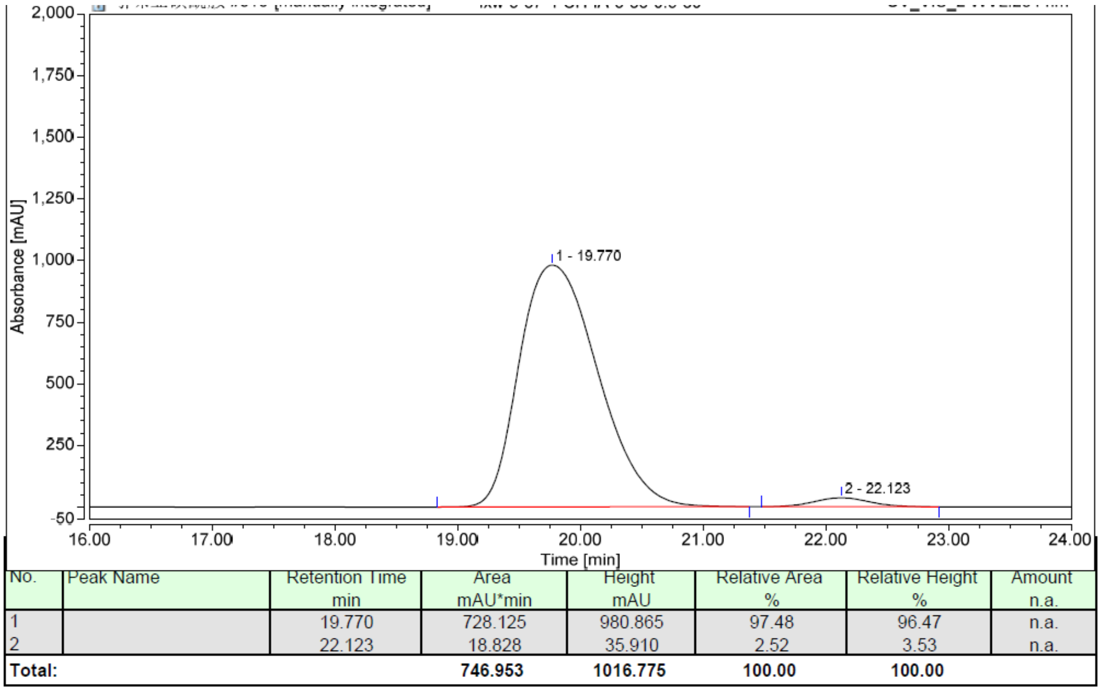

No.	Peak Name	Retention Time min	Area mAU*min	Height mAU	Relative Area %	Relative Height %	Amount n.a.
1		19.770	728.125	980.865	97.48	96.47	n.a.
2		22.123	18.828	35.910	2.52	3.53	n.a.
**Total:**			**746.953**	**1016.775**	**100.00**	**100.00**	

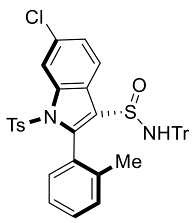

40b

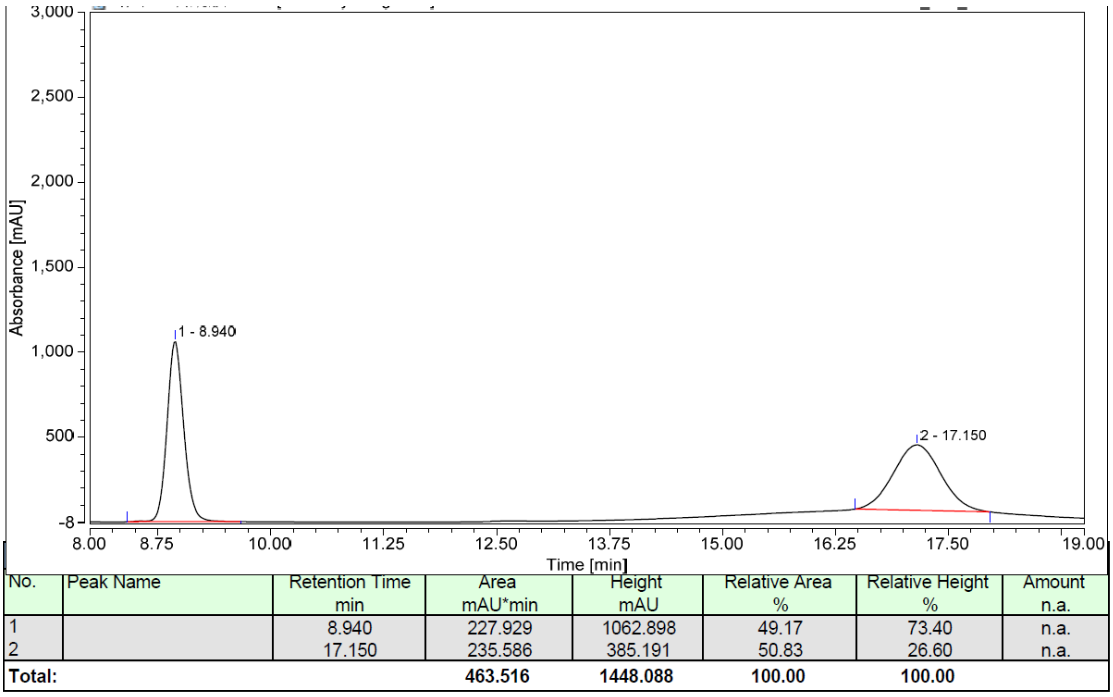

No.	Peak Name	Retention Time min	Area mAU*min	Height mAU	Relative Area %	Relative Height %	Amount n.a.
1		8.940	227.929	1062.898	49.17	73.40	n.a.
2		17.150	235.586	385.191	50.83	26.60	n.a.
**Total:**			**463.516**	**1448.088**	**100.00**	**100.00**	

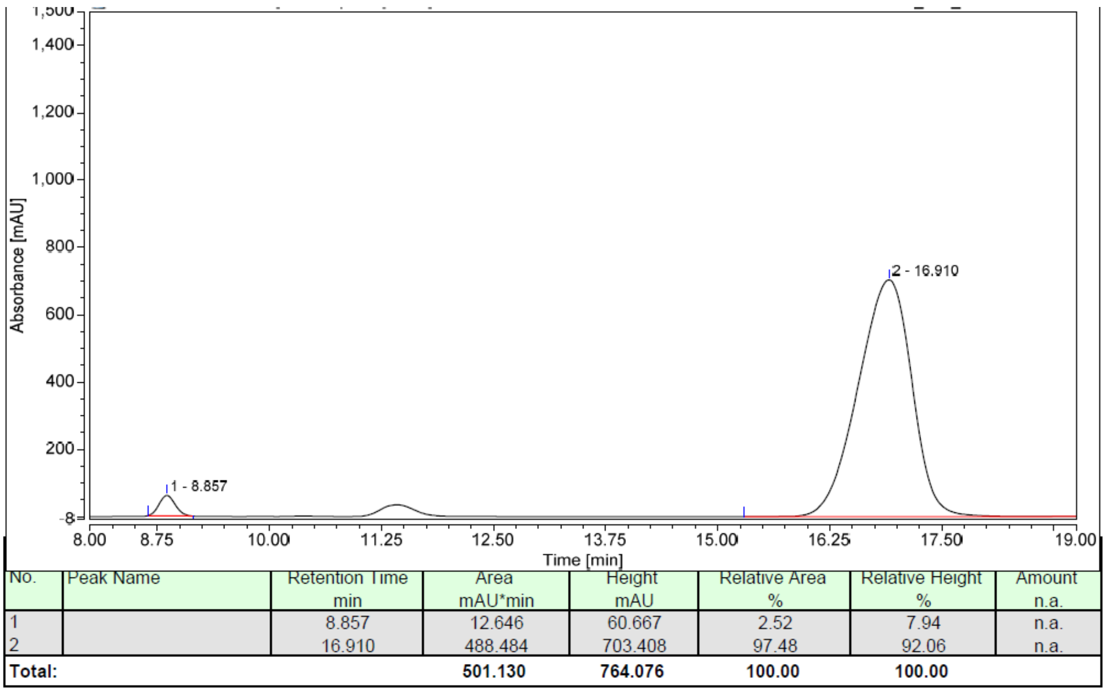

No.	Peak Name	Retention Time min	Area mAU*min	Height mAU	Relative Area %	Relative Height %	Amount n.a.
1		8.857	12.646	60.667	2.52	7.94	n.a.
2		16.910	488.484	703.408	97.48	92.06	n.a.
**Total:**			**501.130**	**764.076**	**100.00**	**100.00**	

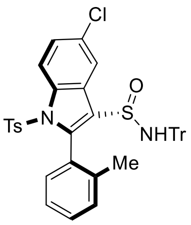

41b

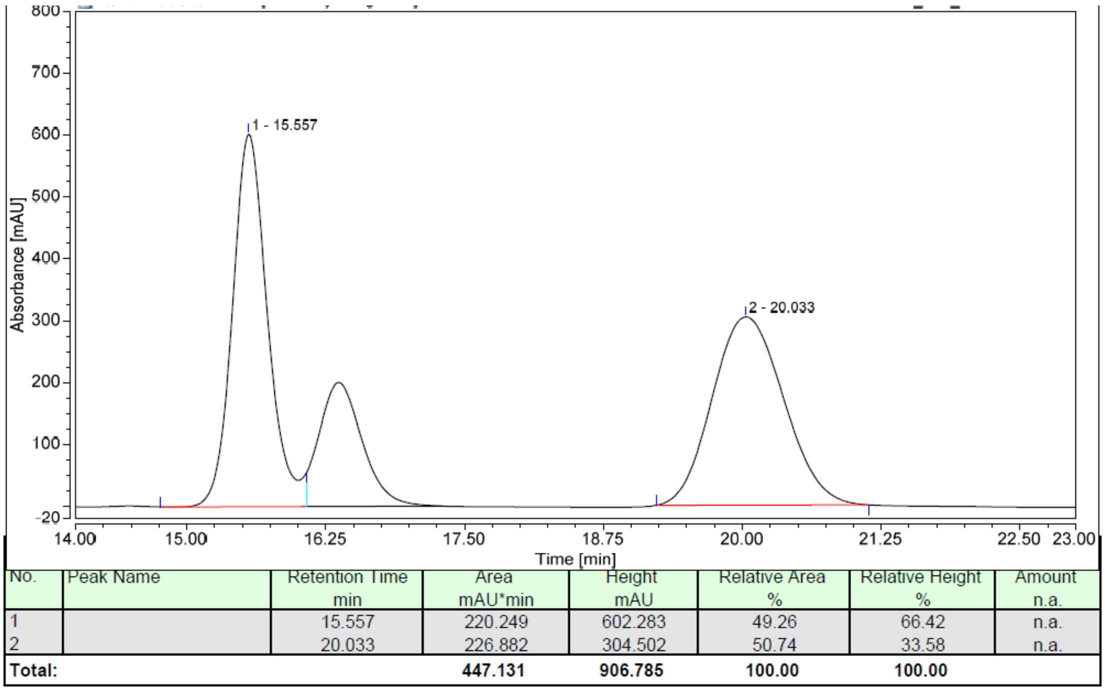

No.	Peak Name	Retention Time min	Area mAU*min	Height mAU	Relative Area %	Relative Height %	Amount n.a.
1		15.557	220.249	602.283	49.26	66.42	n.a.
2		20.033	226.882	304.502	50.74	33.58	n.a.
**Total:**			**447.131**	**906.785**	**100.00**	**100.00**	

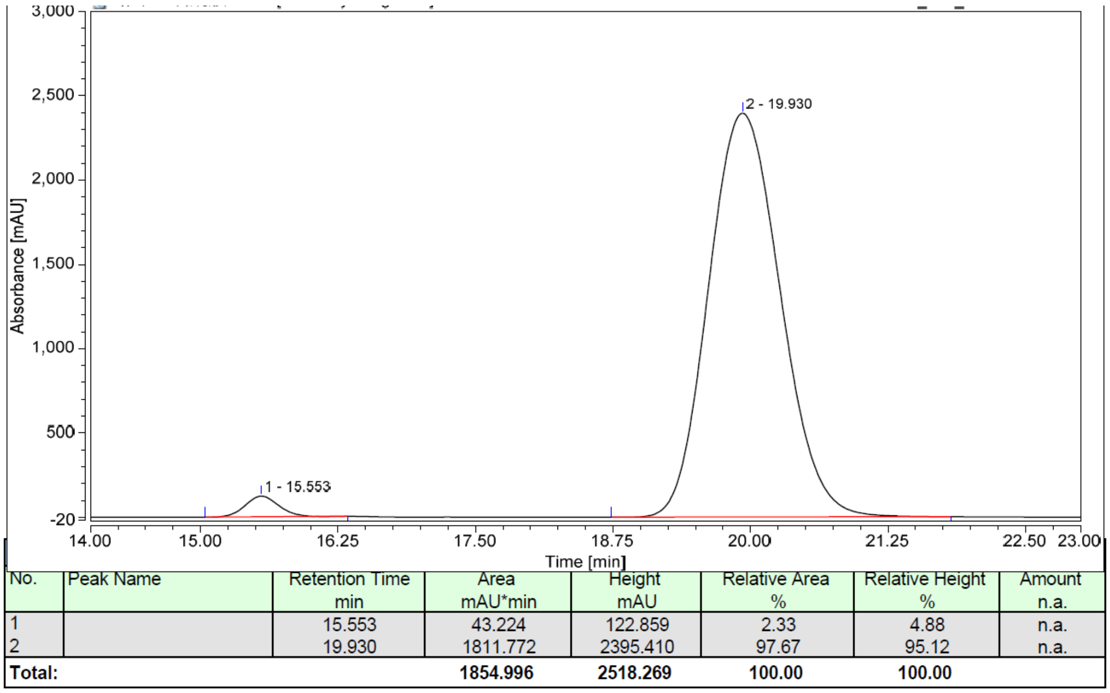

No.	Peak Name	Retention Time min	Area mAU*min	Height mAU	Relative Area %	Relative Height %	Amount n.a.
1		15.553	43.224	122.859	2.33	4.88	n.a.
2		19.930	1811.772	2395.410	97.67	95.12	n.a.
**Total:**			**1854.996**	**2518.269**	**100.00**	**100.00**	

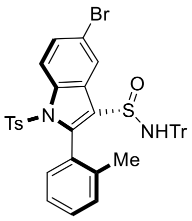

42b

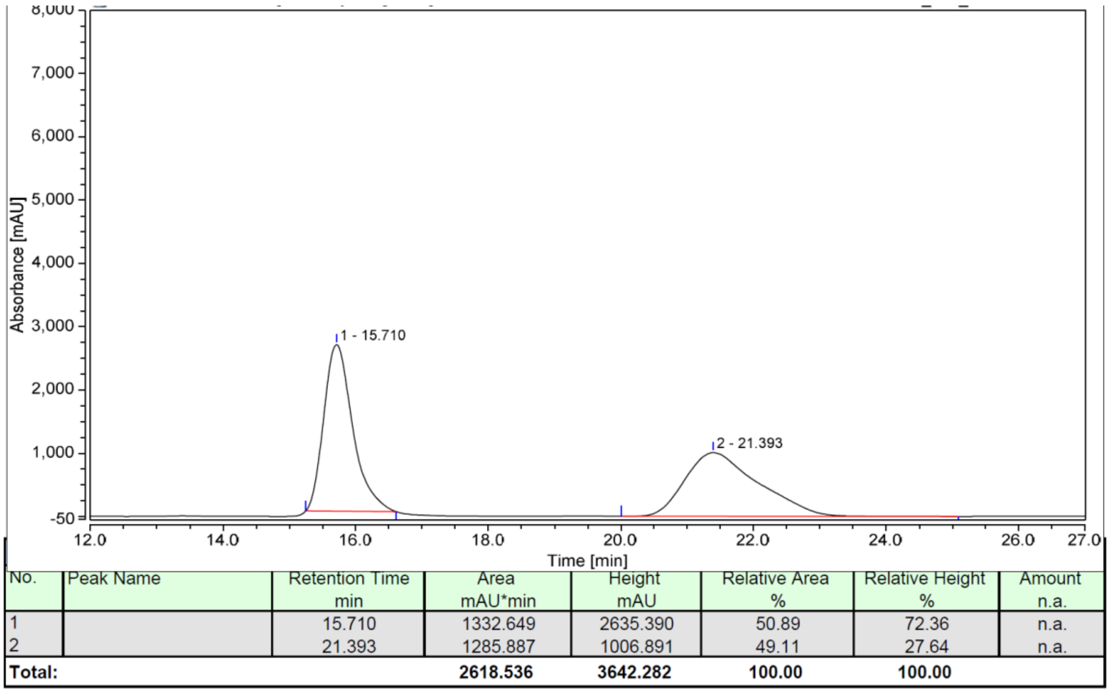

No.	Peak Name	Retention Time min	Area mAU*min	Height mAU	Relative Area %	Relative Height %	Amount n.a.
1		15.710	1332.649	2635.390	50.89	72.36	n.a.
2		21.393	1285.887	1006.891	49.11	27.64	n.a.
**Total:**			**2618.536**	**3642.282**	**100.00**	**100.00**	

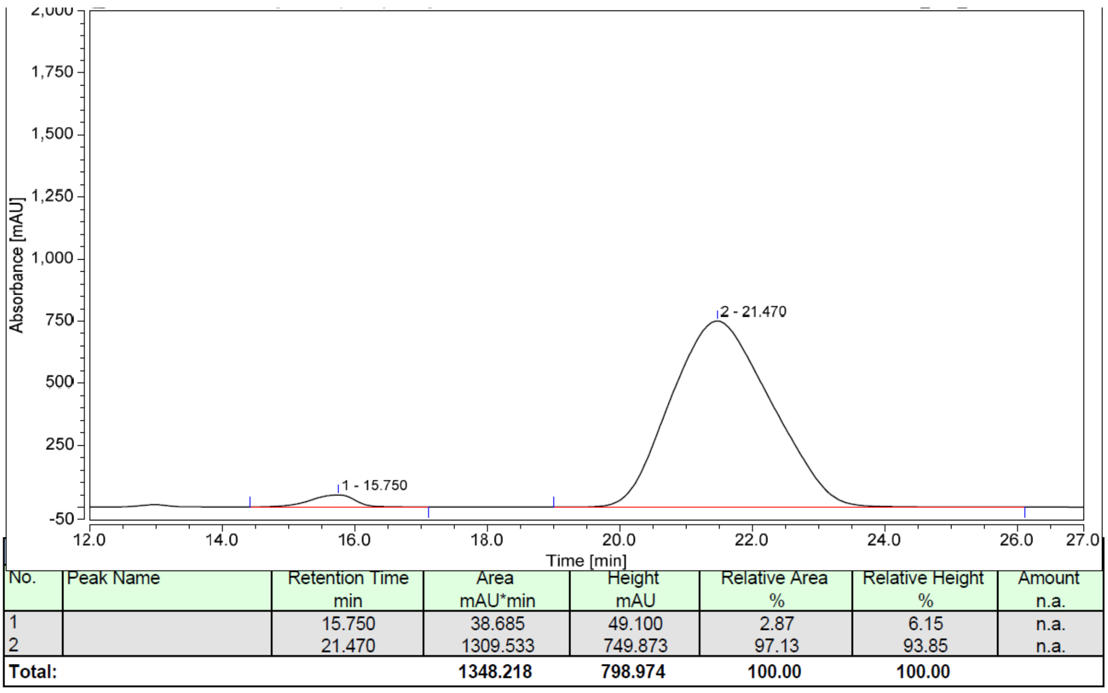

No.	Peak Name	Retention Time min	Area mAU*min	Height mAU	Relative Area %	Relative Height %	Amount n.a.
1		15.750	38.685	49.100	2.87	6.15	n.a.
2		21.470	1309.533	749.873	97.13	93.85	n.a.
**Total:**			**1348.218**	**798.974**	**100.00**	**100.00**	

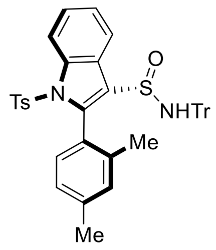

43b

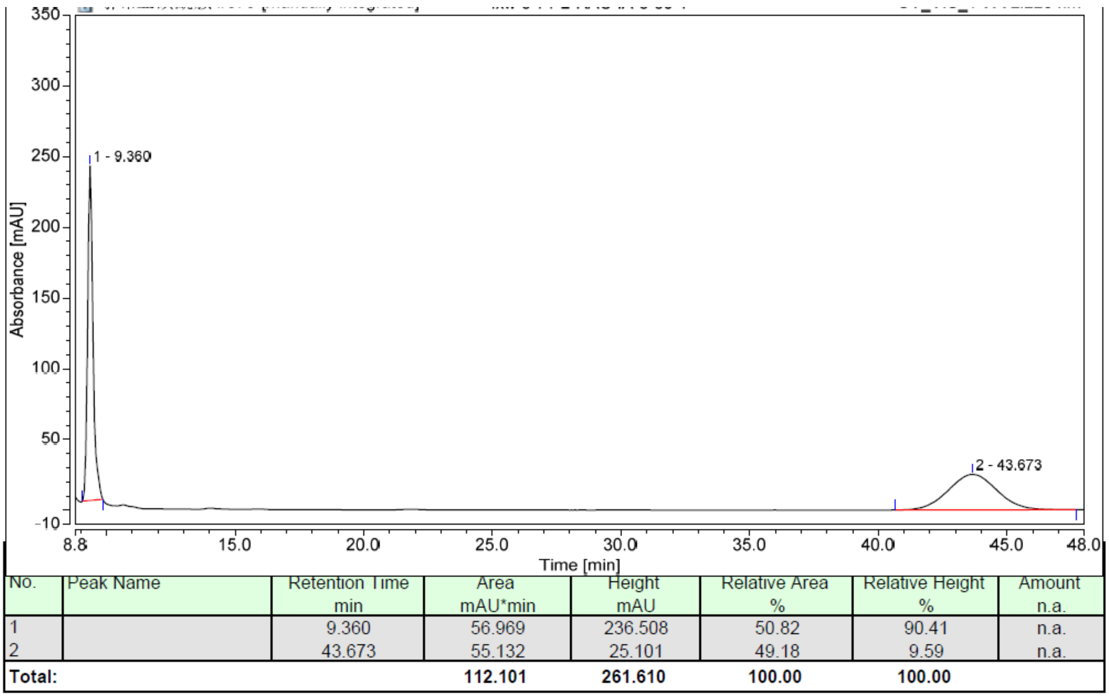

No.	Peak Name	Retention Time min	Area mAU*min	Height mAU	Relative Area %	Relative Height %	Amount n.a.
1		9.360	56.969	236.508	50.82	90.41	n.a.
2		43.673	55.132	25.101	49.18	9.59	n.a.
**Total:**			**112.101**	**261.610**	**100.00**	**100.00**	

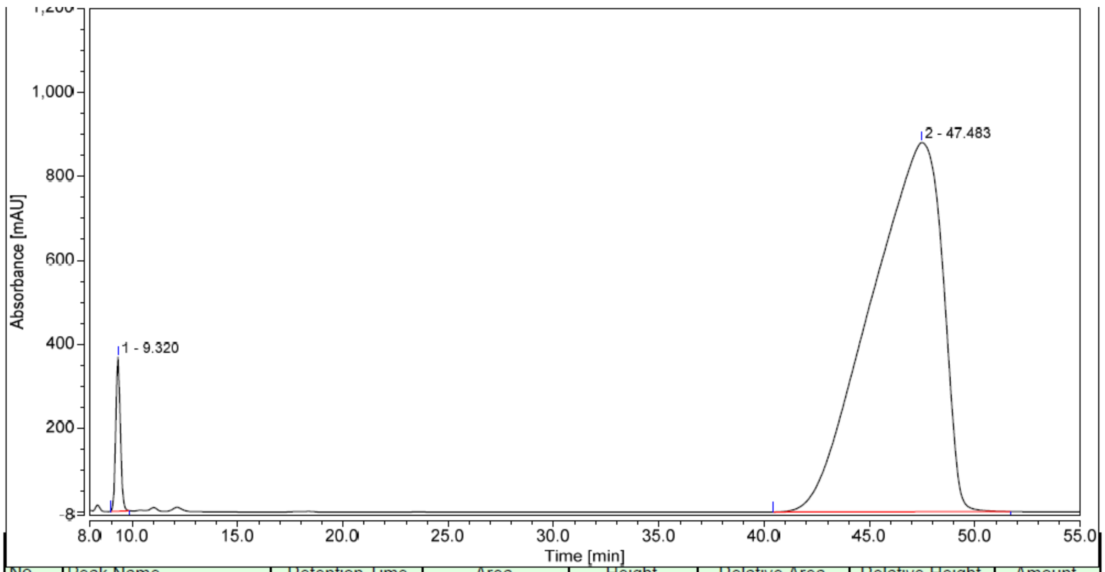

No.	Peak Name	Retention Time min	Area mAU*min	Height mAU	Relative Area %	Relative Height %	Amount n.a.
1		9.320	92.539	367.015	2.60	29.44	n.a.
2		47.483	3466.987	879.644	97.40	70.56	n.a.
**Total:**			**3559.527**	**1246.658**	**100.00**	**100.00**	

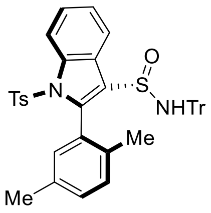

44b

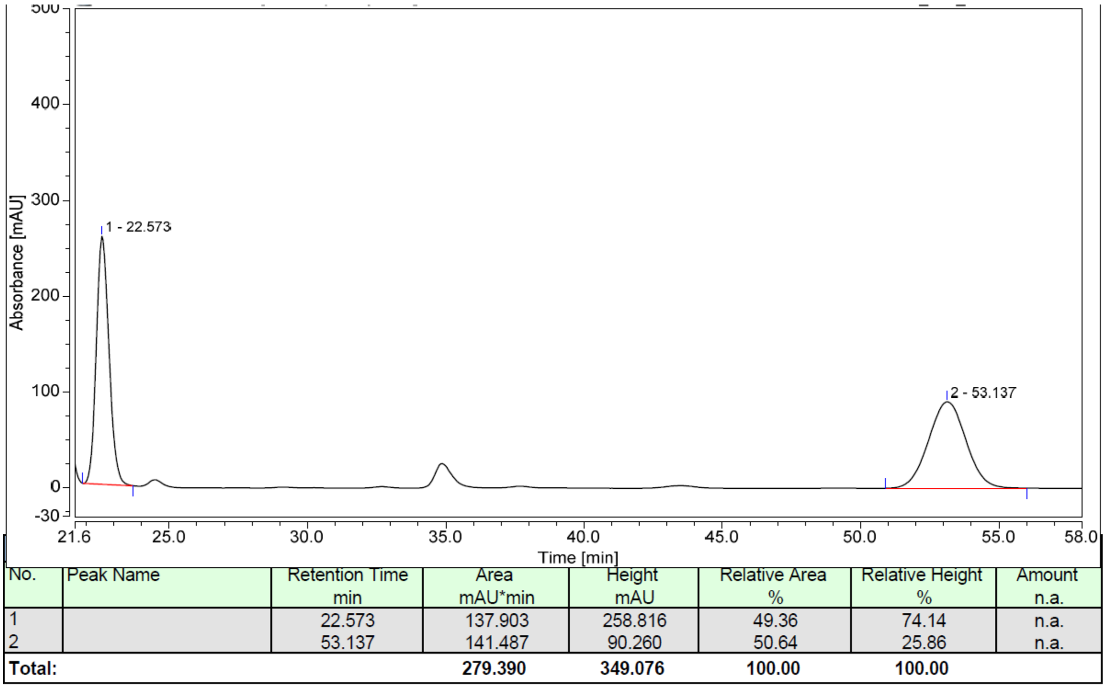

No.	Peak Name	Retention Time min	Area mAU*min	Height mAU	Relative Area %	Relative Height %	Amount n.a.
1		22.573	137.903	258.816	49.36	74.14	n.a.
2		53.137	141.487	90.260	50.64	25.86	n.a.
**Total:**			**279.390**	**349.076**	**100.00**	**100.00**	

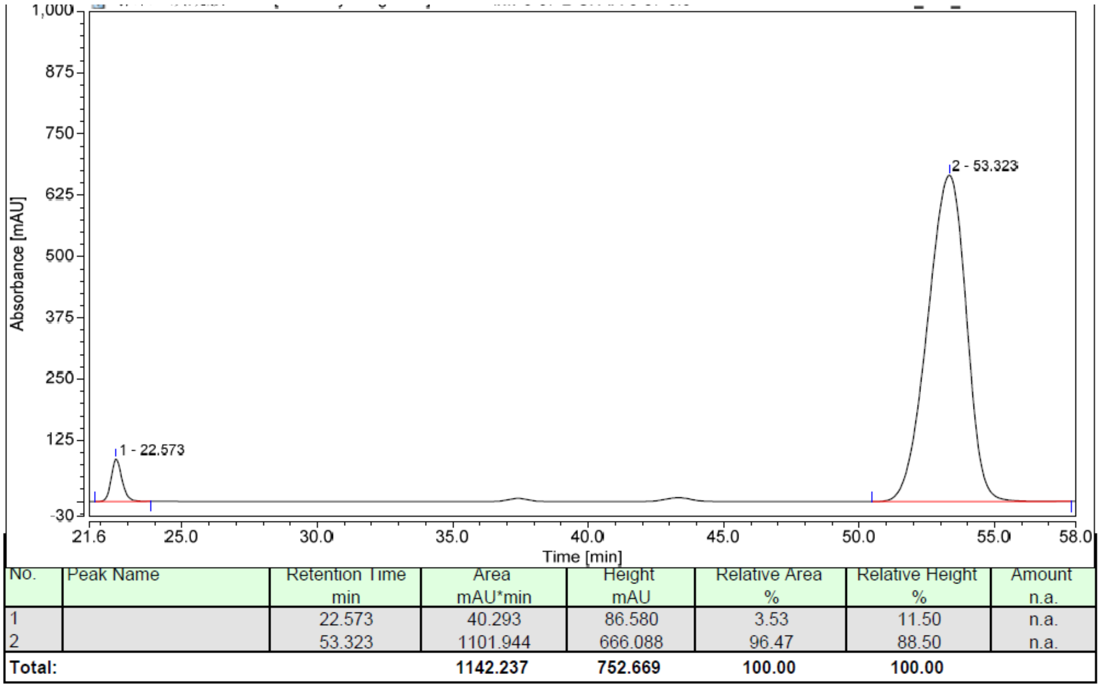

No.	Peak Name	Retention Time min	Area mAU*min	Height mAU	Relative Area %	Relative Height %	Amount n.a.
1		22.573	40.293	86.580	3.53	11.50	n.a.
2		53.323	1101.944	666.088	96.47	88.50	n.a.
**Total:**			**1142.237**	**752.669**	**100.00**	**100.00**	

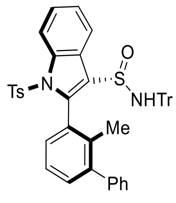

45b

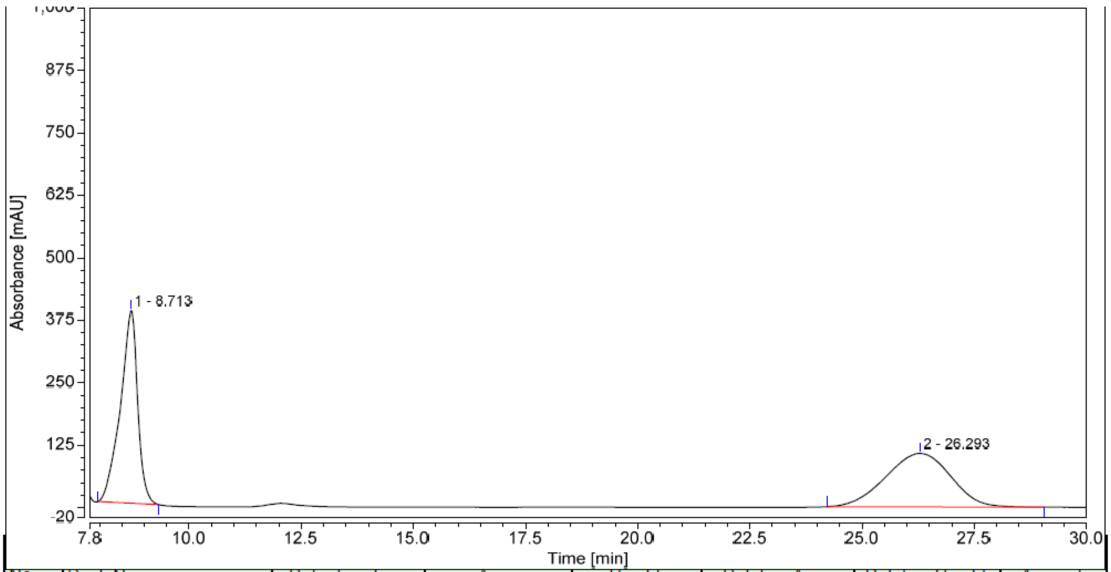

No.	Peak Name	Retention Time min	Area mAU*min	Height mAU	Relative Area %	Relative Height %	Amount n.a.
1		8.713	172.473	385.544	49.02	78.24	n.a.
2		26.293	179.350	107.199	50.98	21.76	n.a.
**Total:**			**351.823**	**492.743**	**100.00**	**100.00**	

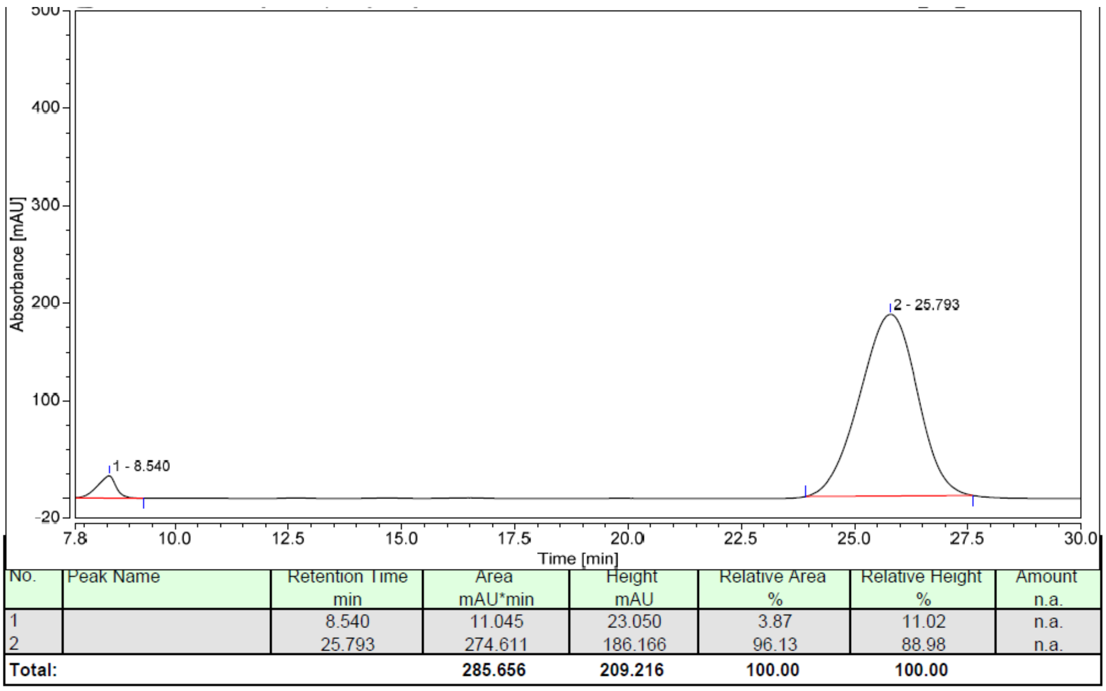

No.	Peak Name	Retention Time min	Area mAU*min	Height mAU	Relative Area %	Relative Height %	Amount n.a.
1		8.540	11.045	23.050	3.87	11.02	n.a.
2		25.793	274.611	186.166	96.13	88.98	n.a.
**Total:**			**285.656**	**209.216**	**100.00**	**100.00**	

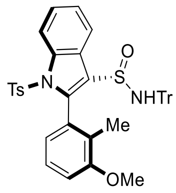

46b

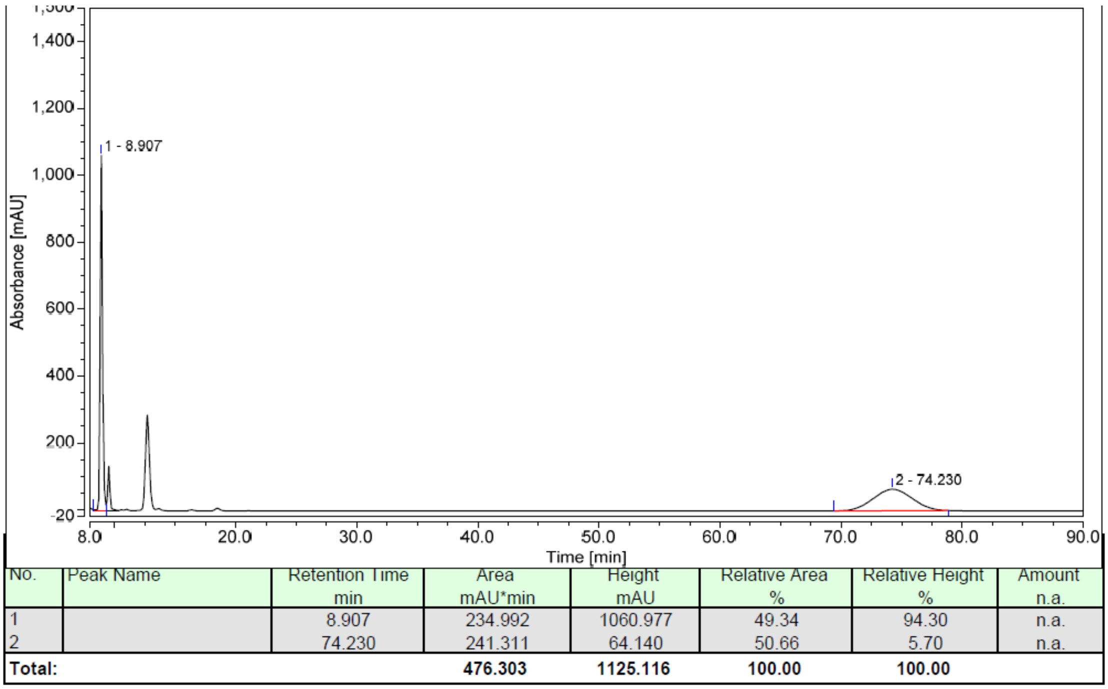

No.	Peak Name	Retention Time<br>min	Area<br>mAU*min	Height<br>mAU	Relative Area<br>%	Relative Height<br>%	Amount<br>n.a.
1		8.907	234.992	1060.977	49.34	94.30	n.a.
2		74.230	241.311	64.140	50.66	5.70	n.a.
**Total:**			**476.303**	**1125.116**	**100.00**	**100.00**	

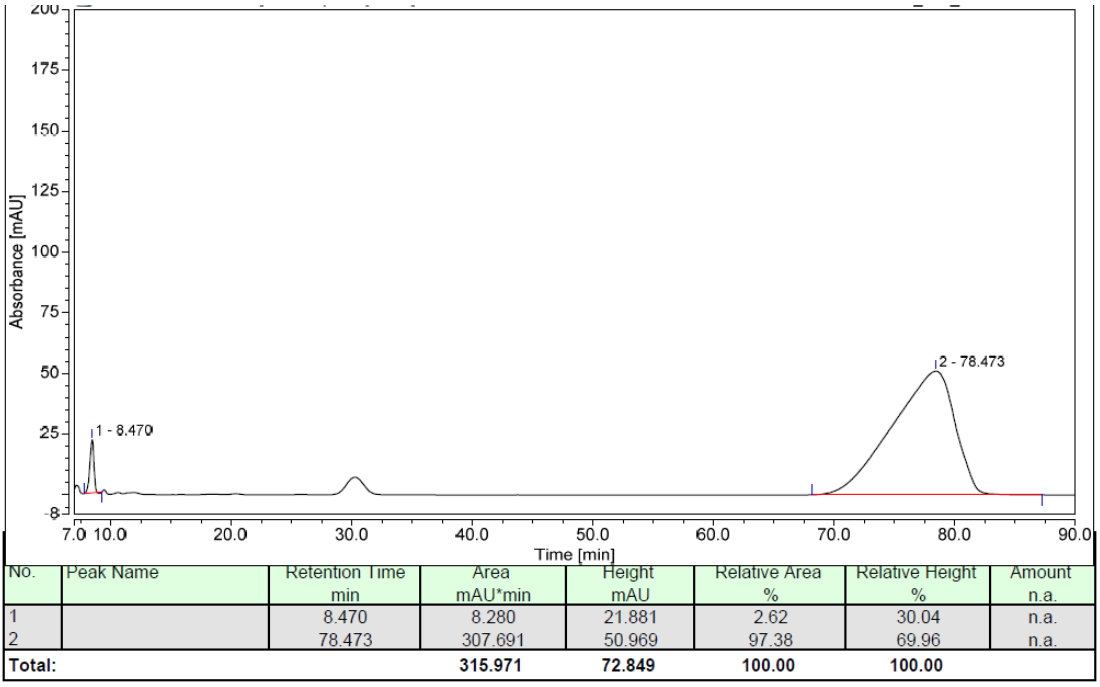

No.	Peak Name	Retention Time<br>min	Area<br>mAU*min	Height<br>mAU	Relative Area<br>%	Relative Height<br>%	Amount<br>n.a.
1		8.470	8.280	21.881	2.62	30.04	n.a.
2		78.473	307.691	50.969	97.38	69.96	n.a.
**Total:**			**315.971**	**72.849**	**100.00**	**100.00**	

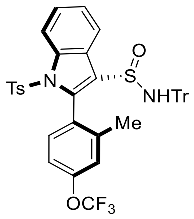

47b

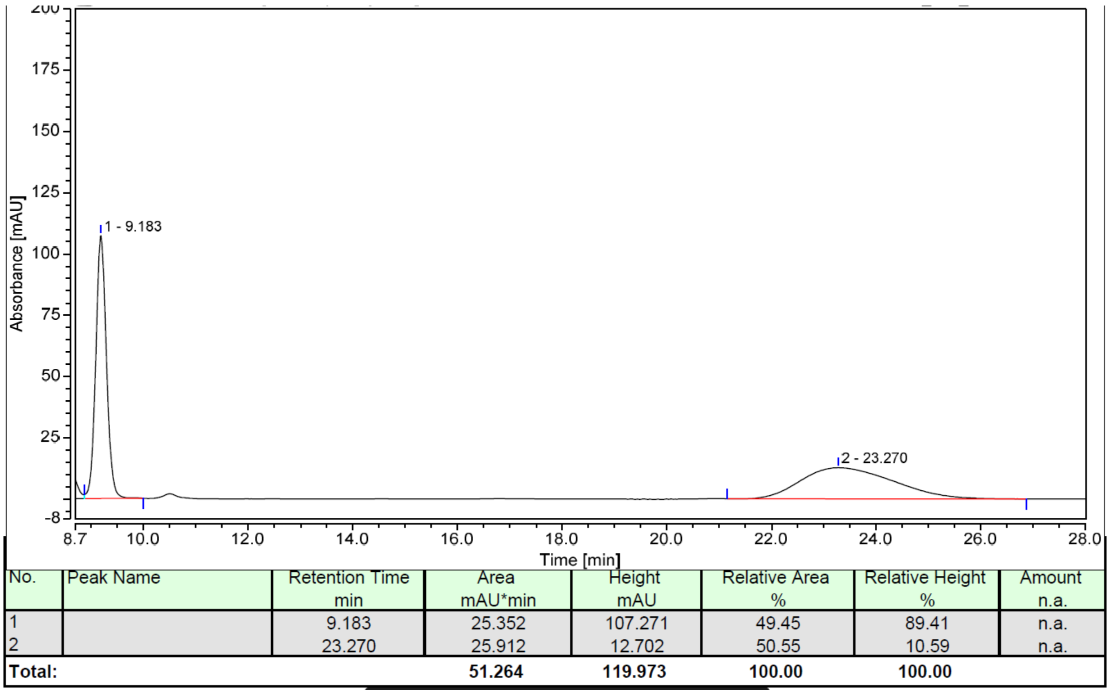

No.	Peak Name	Retention Time min	Area mAU*min	Height mAU	Relative Area %	Relative Height %	Amount n.a.
1		9.183	25.352	107.271	49.45	89.41	n.a.
2		23.270	25.912	12.702	50.55	10.59	n.a.
**Total:**			**51.264**	**119.973**	**100.00**	**100.00**	

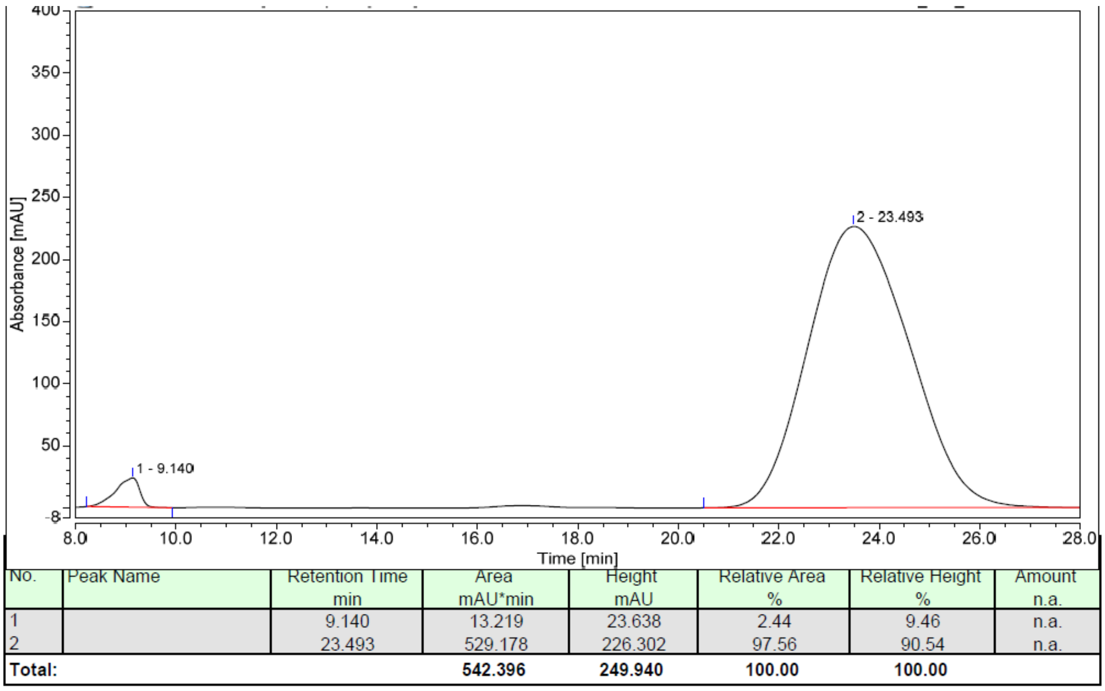

No.	Peak Name	Retention Time min	Area mAU*min	Height mAU	Relative Area %	Relative Height %	Amount n.a.
1		9.140	13.219	23.638	2.44	9.46	n.a.
2		23.493	529.178	226.302	97.56	90.54	n.a.
**Total:**			**542.396**	**249.940**	**100.00**	**100.00**	

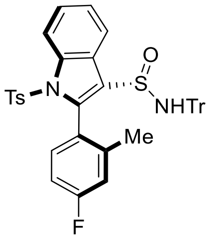

48b

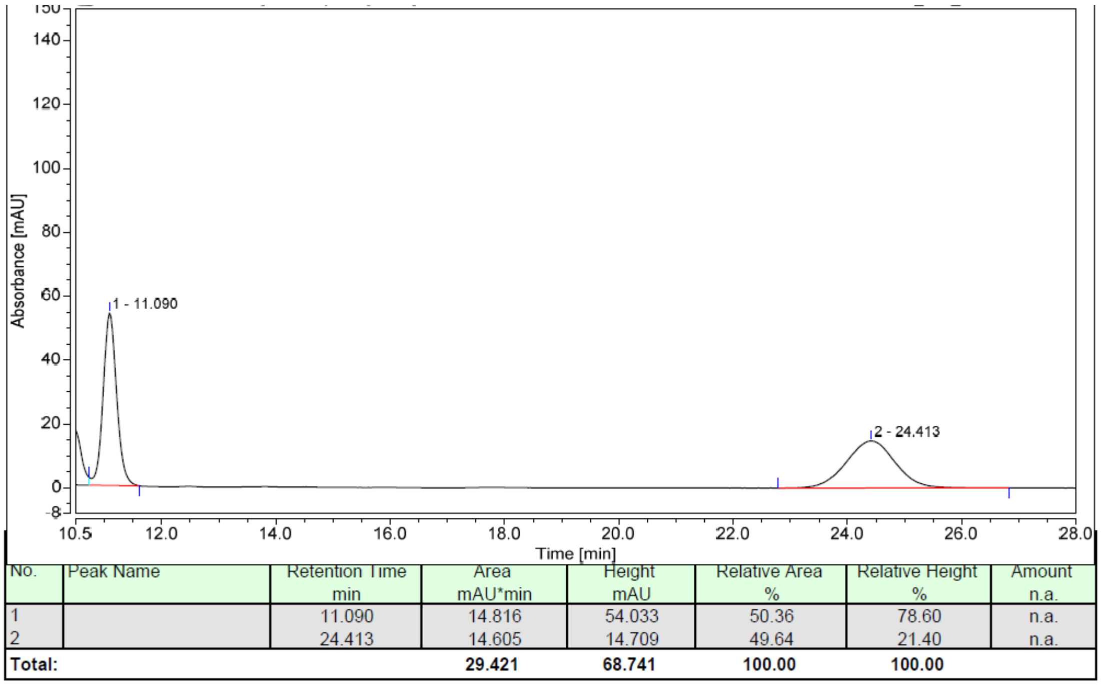

No.	Peak Name	Retention Time min	Area mAU*min	Height mAU	Relative Area %	Relative Height %	Amount n.a.
1		11.090	14.816	54.033	50.36	78.60	n.a.
2		24.413	14.605	14.709	49.64	21.40	n.a.
**Total:**			**29.421**	**68.741**	**100.00**	**100.00**	

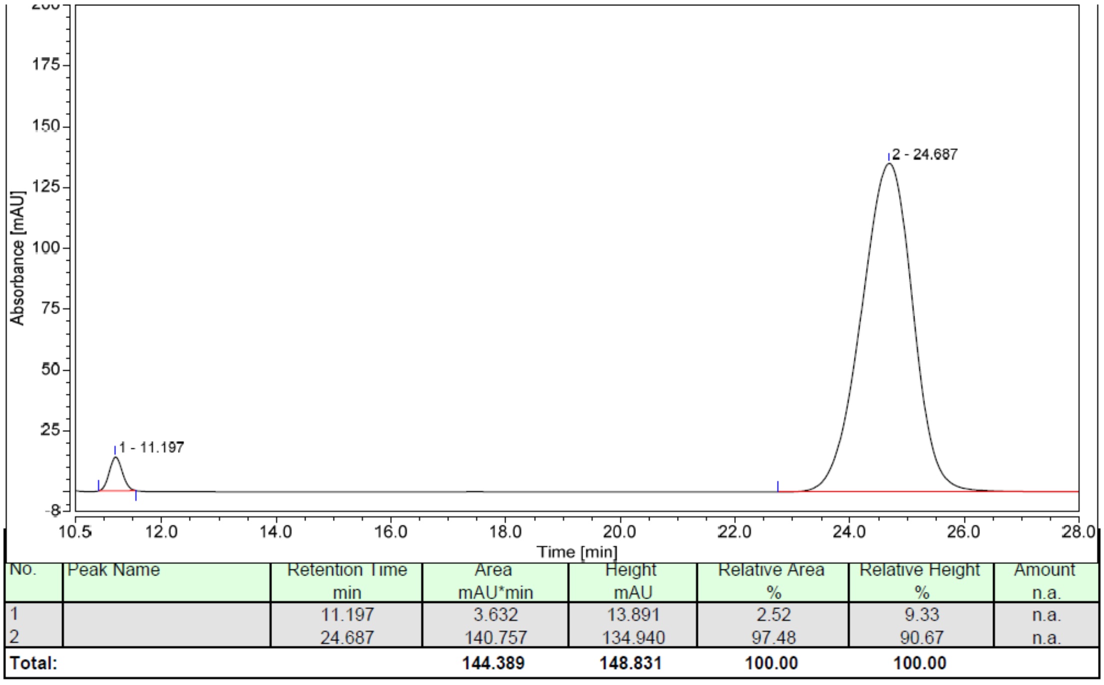

No.	Peak Name	Retention Time min	Area mAU*min	Height mAU	Relative Area %	Relative Height %	Amount n.a.
1		11.197	3.632	13.891	2.52	9.33	n.a.
2		24.687	140.757	134.940	97.48	90.67	n.a.
**Total:**			**144.389**	**148.831**	**100.00**	**100.00**	

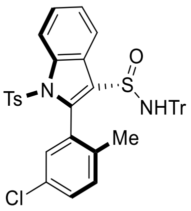

49b

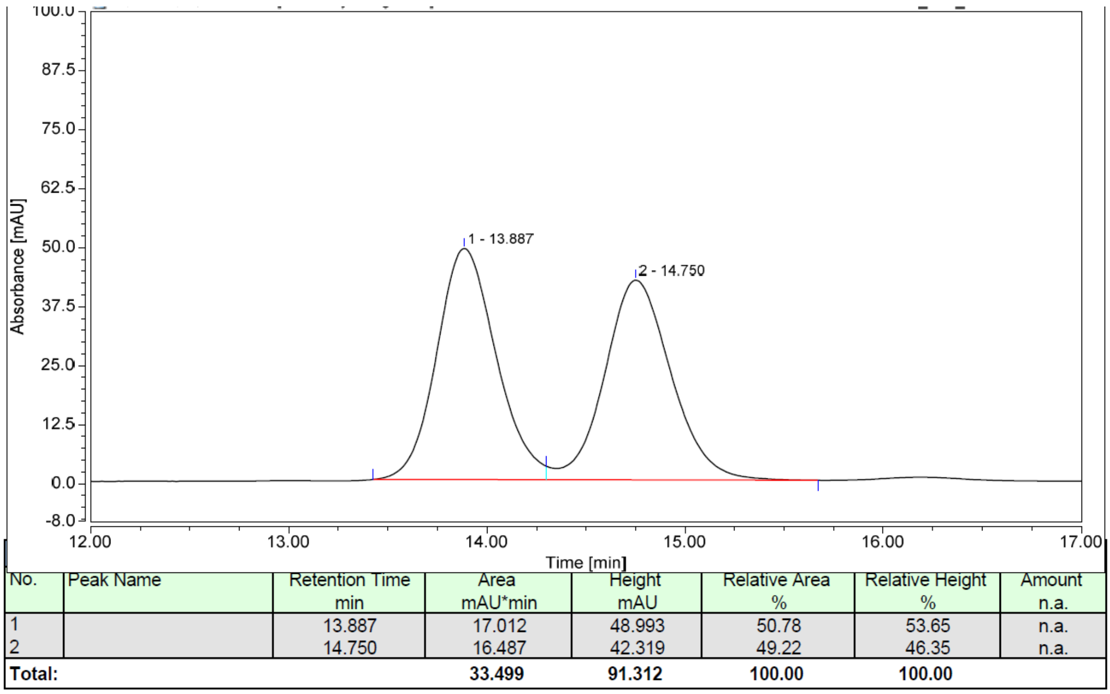

No.	Peak Name	Retention Time min	Area mAU*min	Height mAU	Relative Area %	Relative Height %	Amount n.a.
1		13.887	17.012	48.993	50.78	53.65	n.a.
2		14.750	16.487	42.319	49.22	46.35	n.a.
**Total:**			**33.499**	**91.312**	**100.00**	**100.00**	

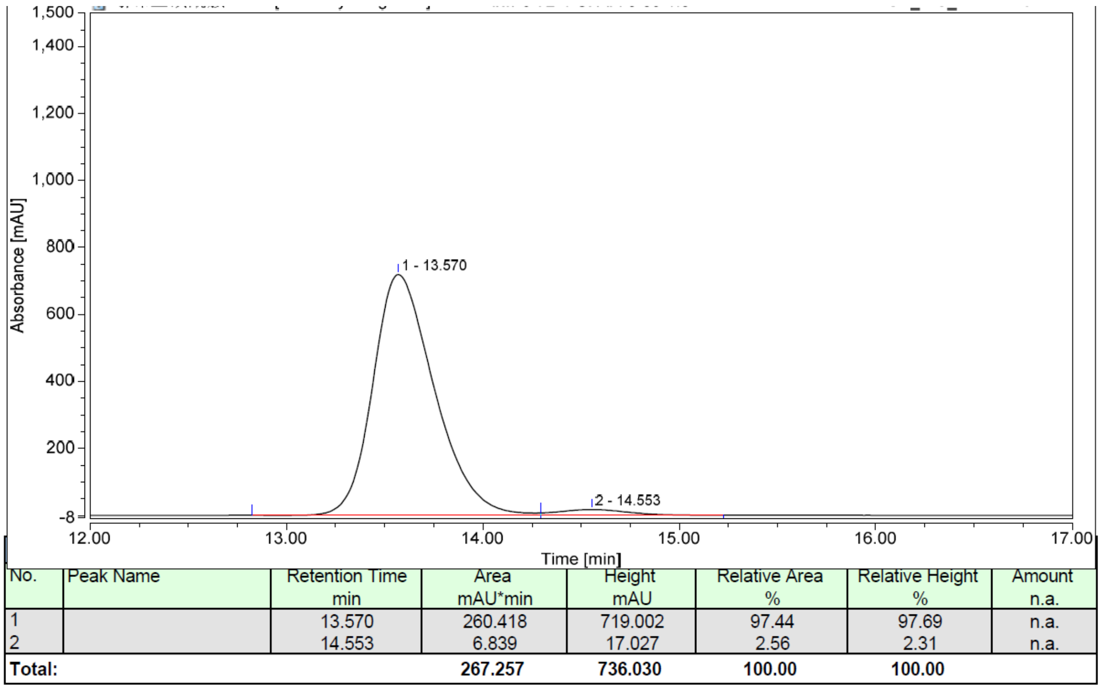

No.	Peak Name	Retention Time min	Area mAU*min	Height mAU	Relative Area %	Relative Height %	Amount n.a.
1		13.570	260.418	719.002	97.44	97.69	n.a.
2		14.553	6.839	17.027	2.56	2.31	n.a.
**Total:**			**267.257**	**736.030**	**100.00**	**100.00**	

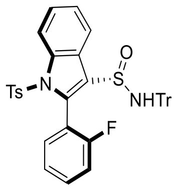

50b

major

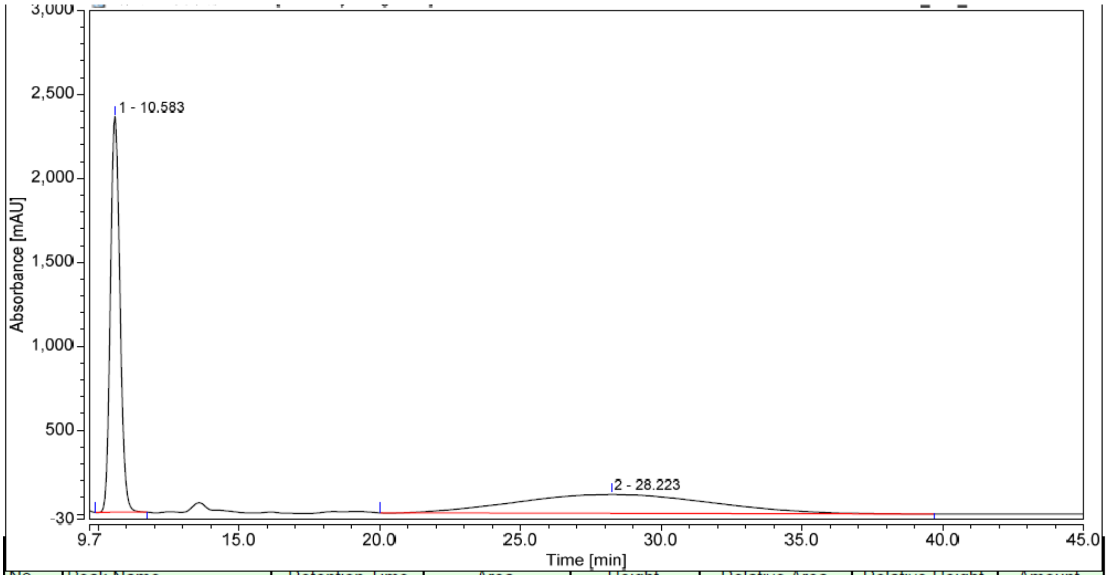

No.	Peak Name	Retention Time min	Area mAU*min	Height mAU	Relative Area %	Relative Height %	Amount n.a.
1		10.583	923.043	2355.964	50.41	95.43	n.a.
2		28.223	907.945	112.907	49.59	4.57	n.a.
**Total:**			**1830.988**	**2468.871**	**100.00**	**100.00**	

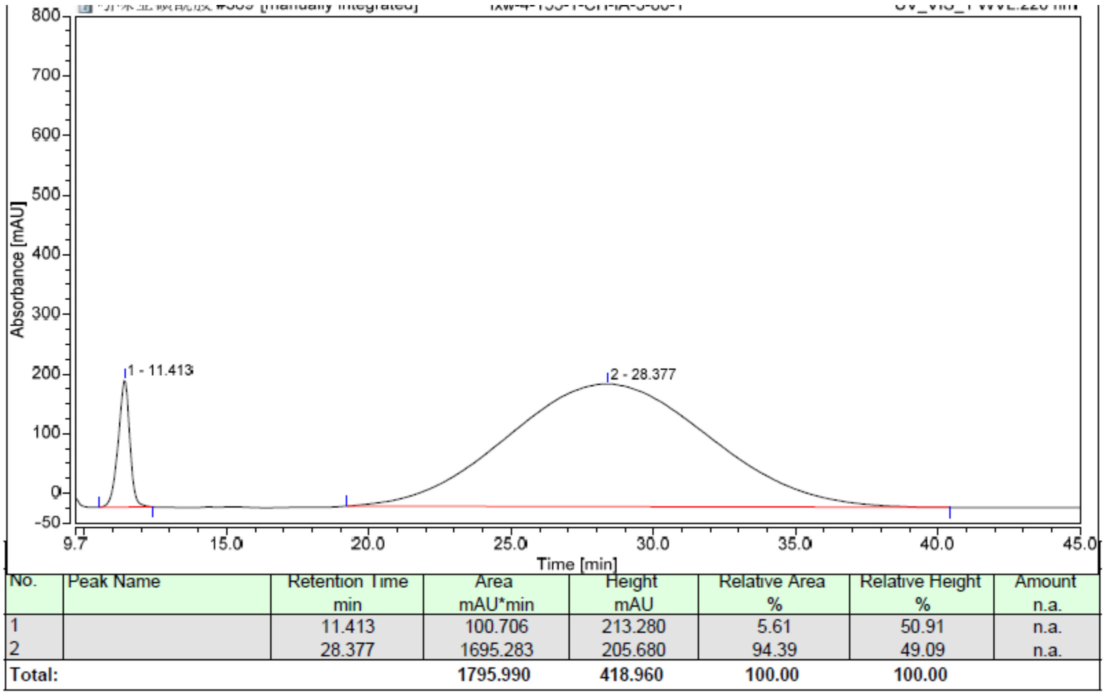

No.	Peak Name	Retention Time min	Area mAU*min	Height mAU	Relative Area %	Relative Height %	Amount n.a.
1		11.413	100.706	213.280	5.61	50.91	n.a.
2		28.377	1695.283	205.680	94.39	49.09	n.a.
**Total:**			**1795.990**	**418.960**	**100.00**	**100.00**	

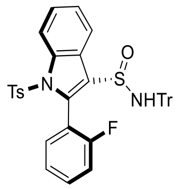

50b

minor

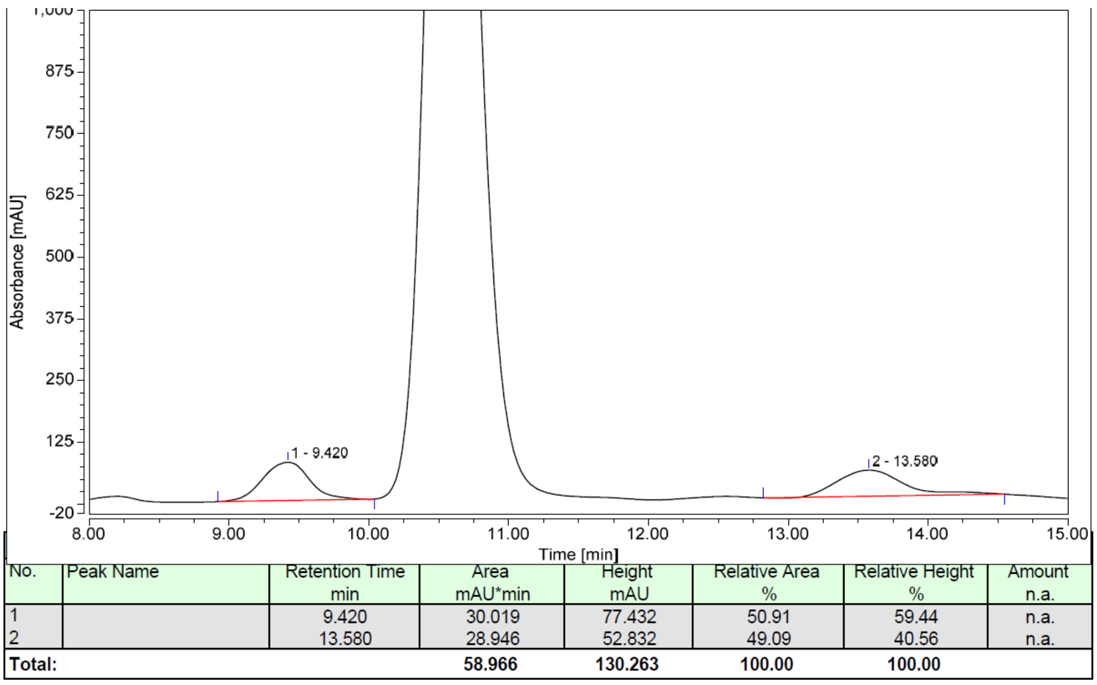

No.	Peak Name	Retention Time<br>min	Area<br>mAU*min	Height<br>mAU	Relative Area<br>%	Relative Height<br>%	Amount<br>n.a.
1		9.420	30.019	77.432	50.91	59.44	n.a.
2		13.580	28.946	52.832	49.09	40.56	n.a.
**Total:**			**58.966**	**130.263**	**100.00**	**100.00**	

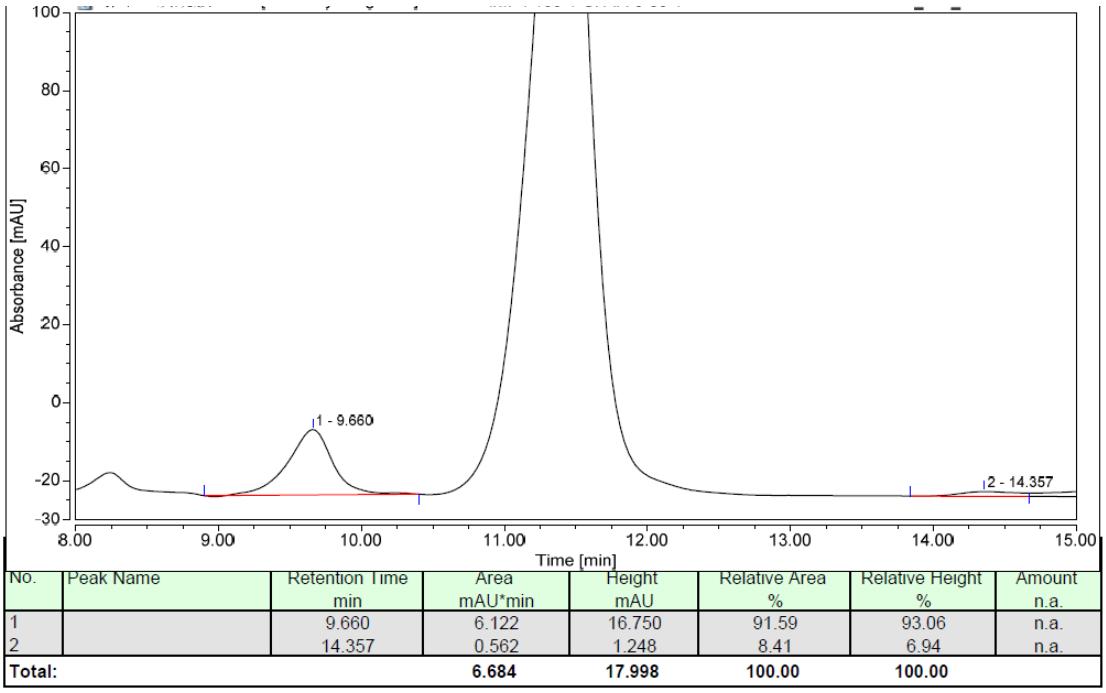

No.	Peak Name	Retention Time<br>min	Area<br>mAU*min	Height<br>mAU	Relative Area<br>%	Relative Height<br>%	Amount<br>n.a.
1		9.660	6.122	16.750	91.59	93.06	n.a.
2		14.357	0.562	1.248	8.41	6.94	n.a.
**Total:**			**6.684**	**17.998**	**100.00**	**100.00**	

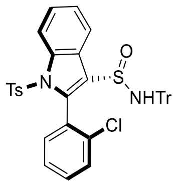

51b

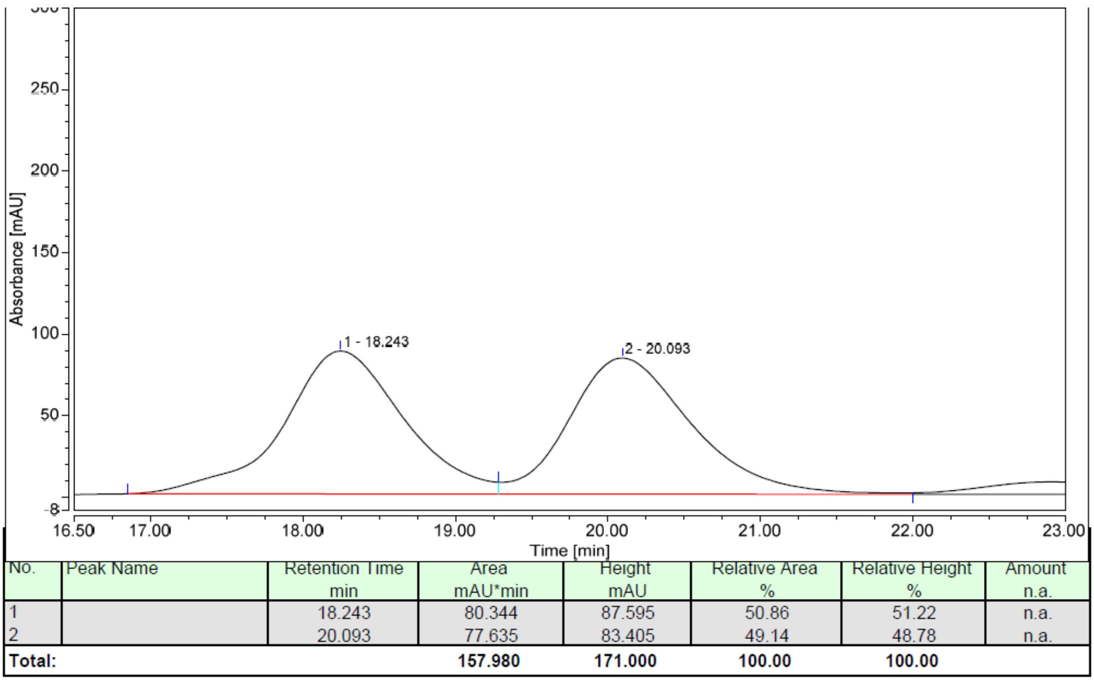

No.	Peak Name	Retention Time min	Area mAU*min	Height mAU	Relative Area %	Relative Height %	Amount n.a.
1		18.243	80.344	87.595	50.86	51.22	n.a.
2		20.093	77.635	83.405	49.14	48.78	n.a.
**Total:**			**157.980**	**171.000**	**100.00**	**100.00**	

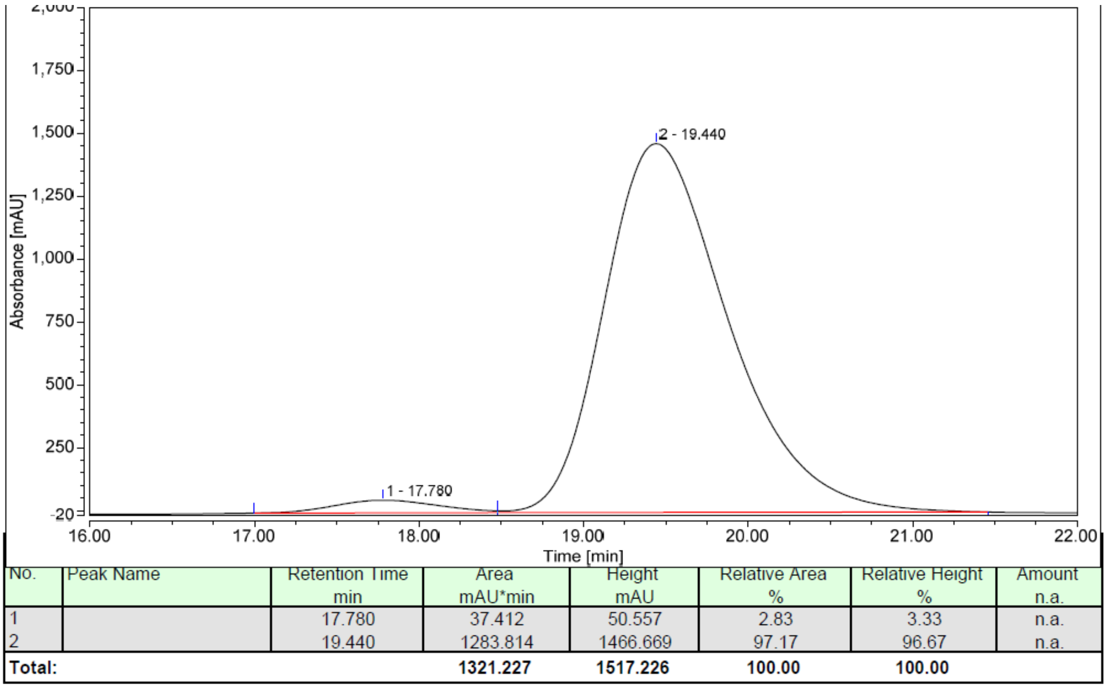

No.	Peak Name	Retention Time min	Area mAU*min	Height mAU	Relative Area %	Relative Height %	Amount n.a.
1		17.780	37.412	50.557	2.83	3.33	n.a.
2		19.440	1283.814	1466.669	97.17	96.67	n.a.
**Total:**			**1321.227**	**1517.226**	**100.00**	**100.00**	

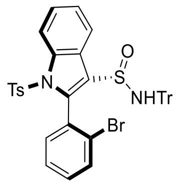

52b

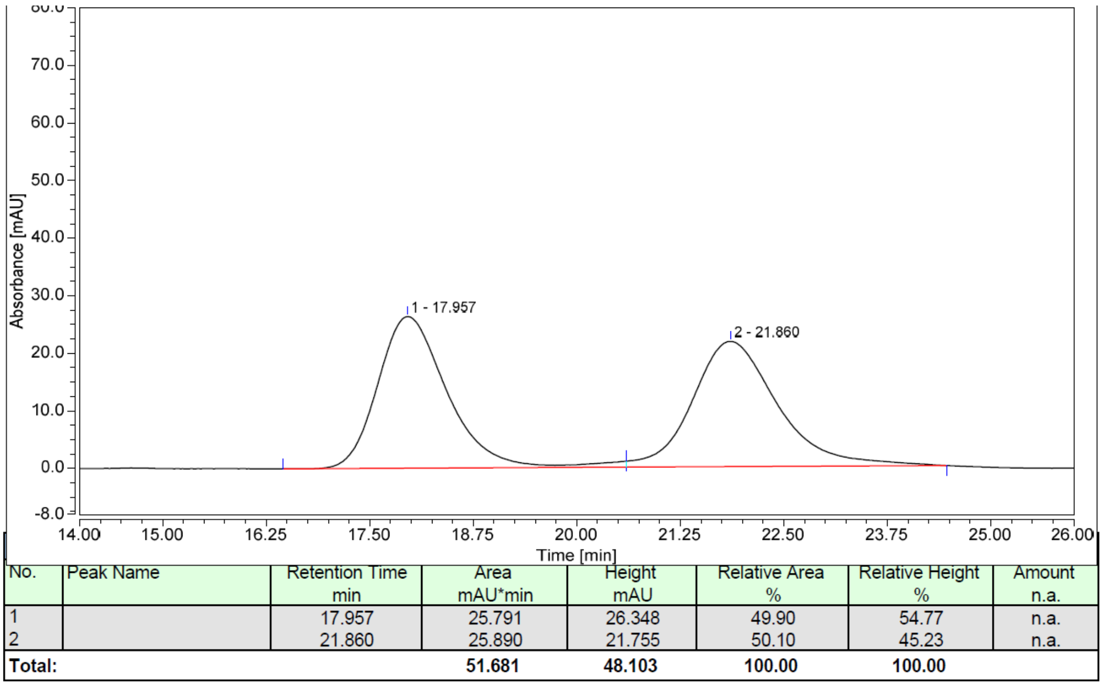

No.	Peak Name	Retention Time min	Area mAU*min	Height mAU	Relative Area %	Relative Height %	Amount n.a.
1		17.957	25.791	26.348	49.90	54.77	n.a.
2		21.860	25.890	21.755	50.10	45.23	n.a.
**Total:**			**51.681**	**48.103**	**100.00**	**100.00**	

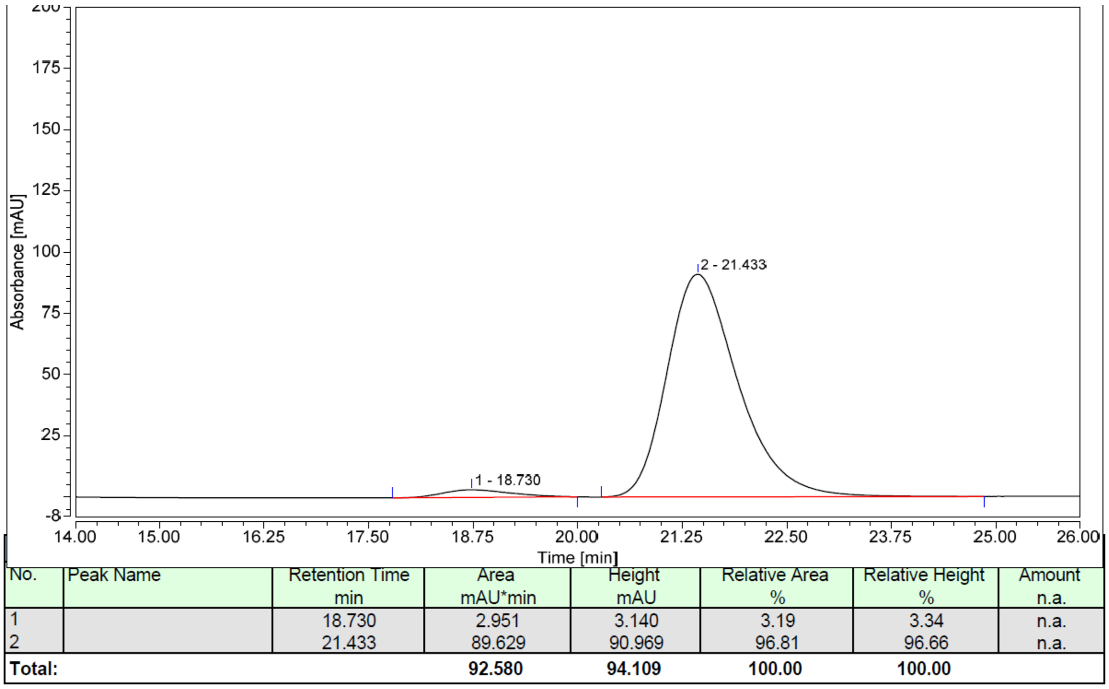

No.	Peak Name	Retention Time min	Area mAU*min	Height mAU	Relative Area %	Relative Height %	Amount n.a.
1		18.730	2.951	3.140	3.19	3.34	n.a.
2		21.433	89.629	90.969	96.81	96.66	n.a.
**Total:**			**92.580**	**94.109**	**100.00**	**100.00**	

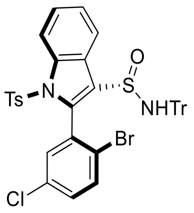

53b

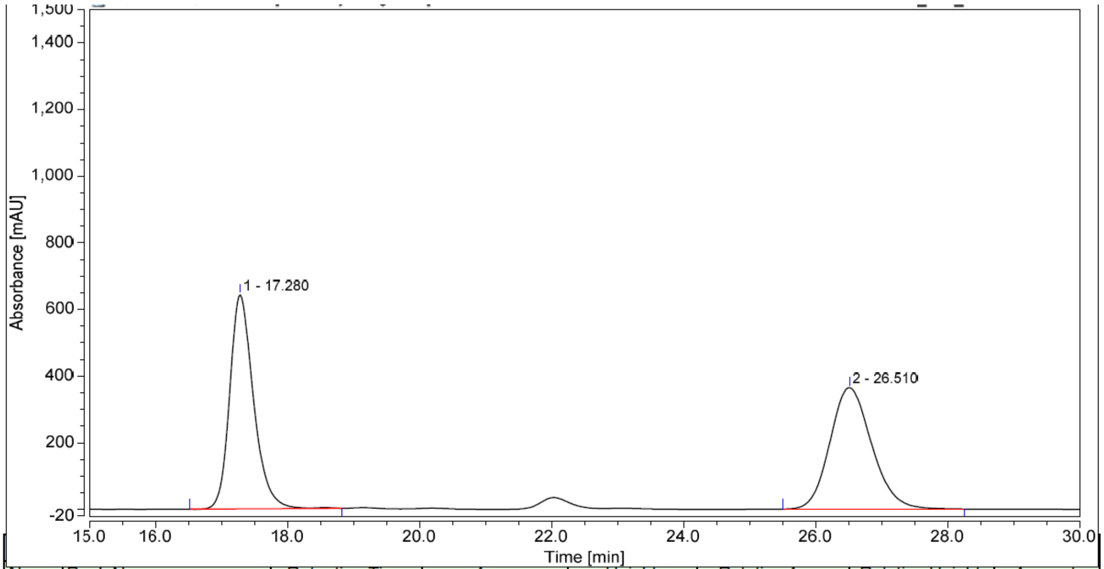

No.	Peak Name	Retention Time<br>min	Area<br>mAU*min	Height<br>mAU	Relative Area<br>%	Relative Height<br>%	Amount<br>n.a.
1		17.280	268.586	642.789	50.17	63.81	n.a.
2		26.510	266.817	364.570	49.83	36.19	n.a.
**Total:**			**535.403**	**1007.359**	**100.00**	**100.00**	

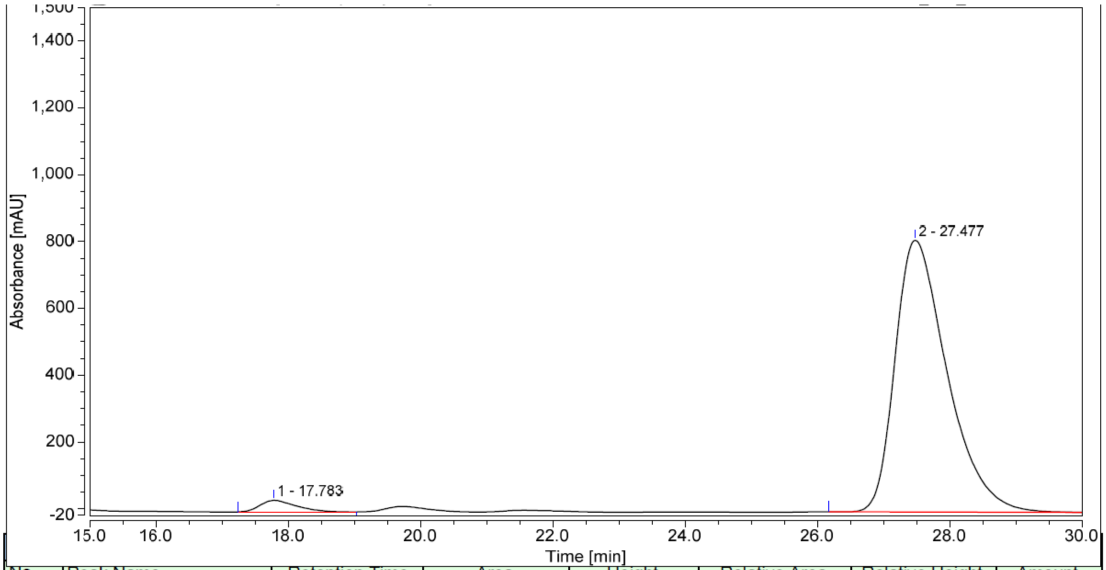

No.	Peak Name	Retention Time<br>min	Area<br>mAU*min	Height<br>mAU	Relative Area<br>%	Relative Height<br>%	Amount<br>n.a.
1		17.783	21.690	34.524	2.84	4.07	n.a.
2		27.477	740.896	813.355	97.16	95.93	n.a.
**Total:**			**762.586**	**847.879**	**100.00**	**100.00**	

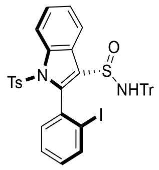

54b

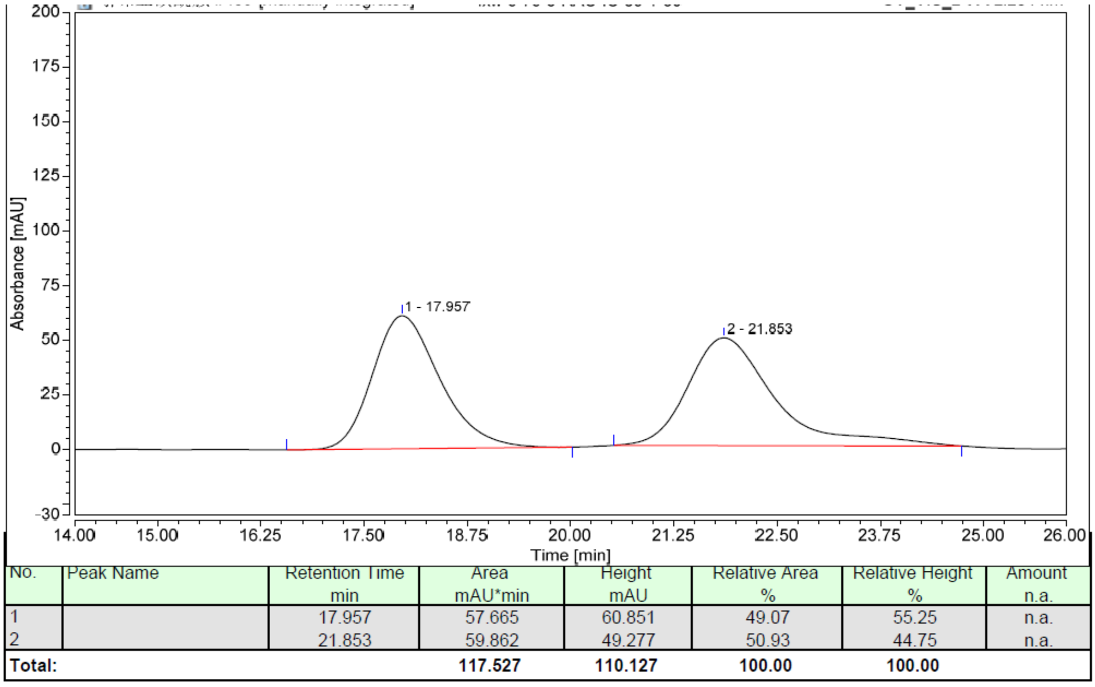

No.	Peak Name	Retention Time min	Area mAU*min	Height mAU	Relative Area %	Relative Height %	Amount n.a.
1		17.957	57.665	60.851	49.07	55.25	n.a.
2		21.853	59.862	49.277	50.93	44.75	n.a.
**Total:**			**117.527**	**110.127**	**100.00**	**100.00**	

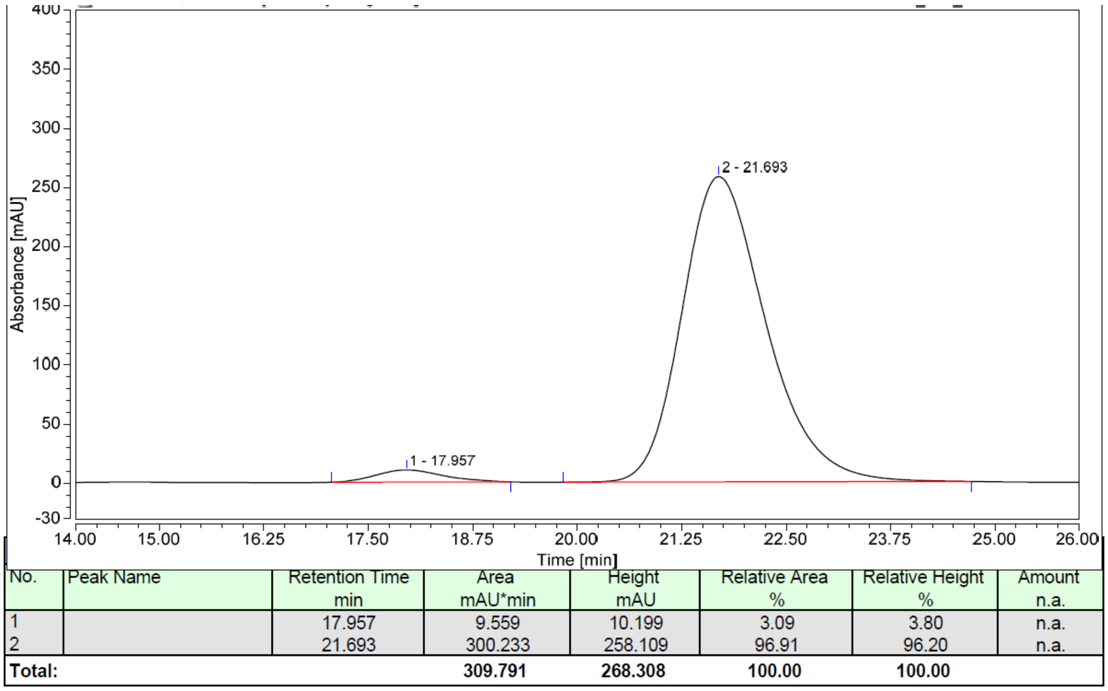

No.	Peak Name	Retention Time min	Area mAU*min	Height mAU	Relative Area %	Relative Height %	Amount n.a.
1		17.957	9.559	10.199	3.09	3.80	n.a.
2		21.693	300.233	258.109	96.91	96.20	n.a.
**Total:**			**309.791**	**268.308**	**100.00**	**100.00**	

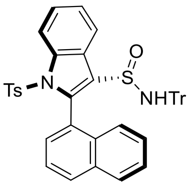

55b

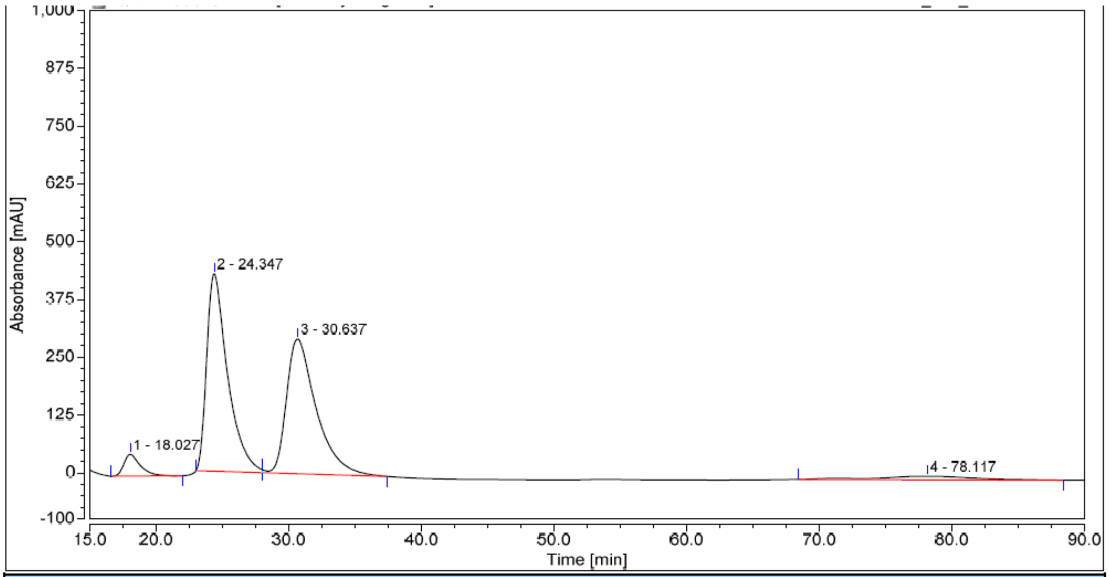

Integration Results

No.	Peak Name	Retention Time min	Area mAU*min	Height mAU	Relative Area %	Relative Height %	Amount n.a.
1		18.027	66.778	47.099	3.99	6.08	n.a.
2		24.347	780.903	427.800	46.68	55.23	n.a.
3		30.637	759.298	291.905	45.39	37.69	n.a.
4		78.117	65.986	7.736	3.94	1.00	n.a.
**Total:**			**1672.965**	**774.540**	**100.00**	**100.00**	

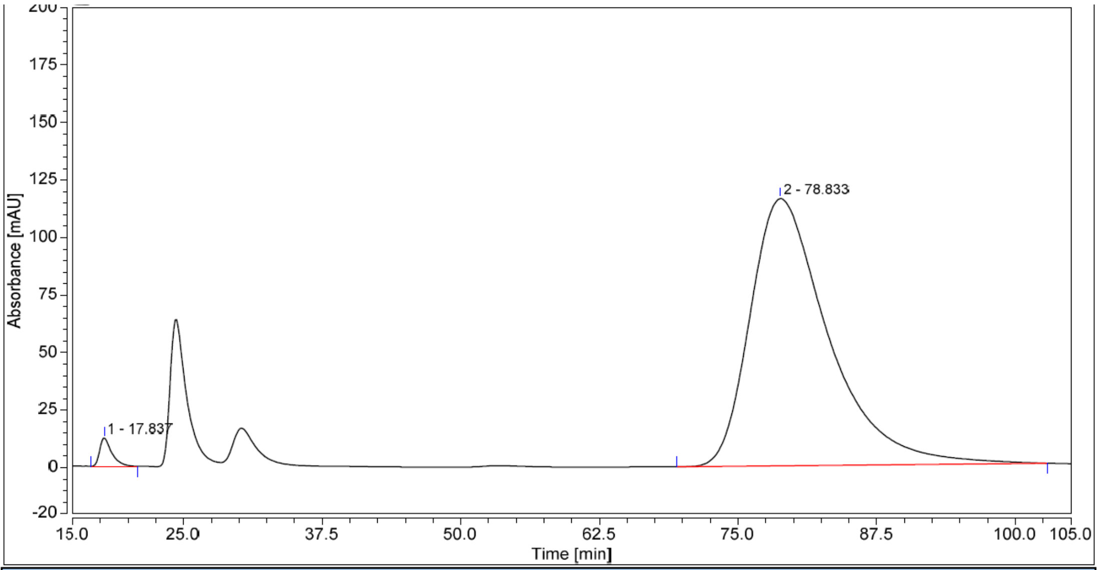

Integration Results

No.	Peak Name	Retention Time min	Area mAU*min	Height mAU	Relative Area %	Relative Height %	Amount n.a.
1		17.837	15.545	12.351	1.61	9.61	n.a.
2		78.833	952.513	116.173	98.39	90.39	n.a.
**Total:**			**968.058**	**128.524**	**100.00**	**100.00**	

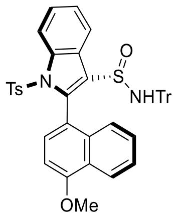

56b

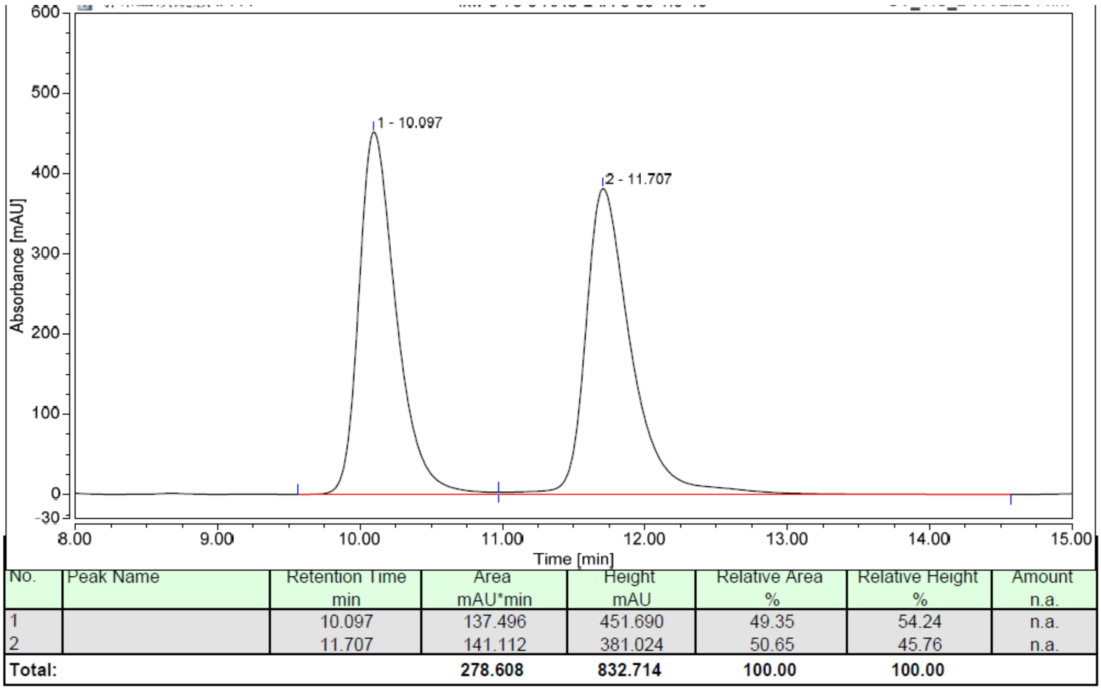

No.	Peak Name	Retention Time min	Area mAU*min	Height mAU	Relative Area %	Relative Height %	Amount n.a.
1		10.097	137.496	451.690	49.35	54.24	n.a.
2		11.707	141.112	381.024	50.65	45.76	n.a.
**Total:**			**278.608**	**832.714**	**100.00**	**100.00**	

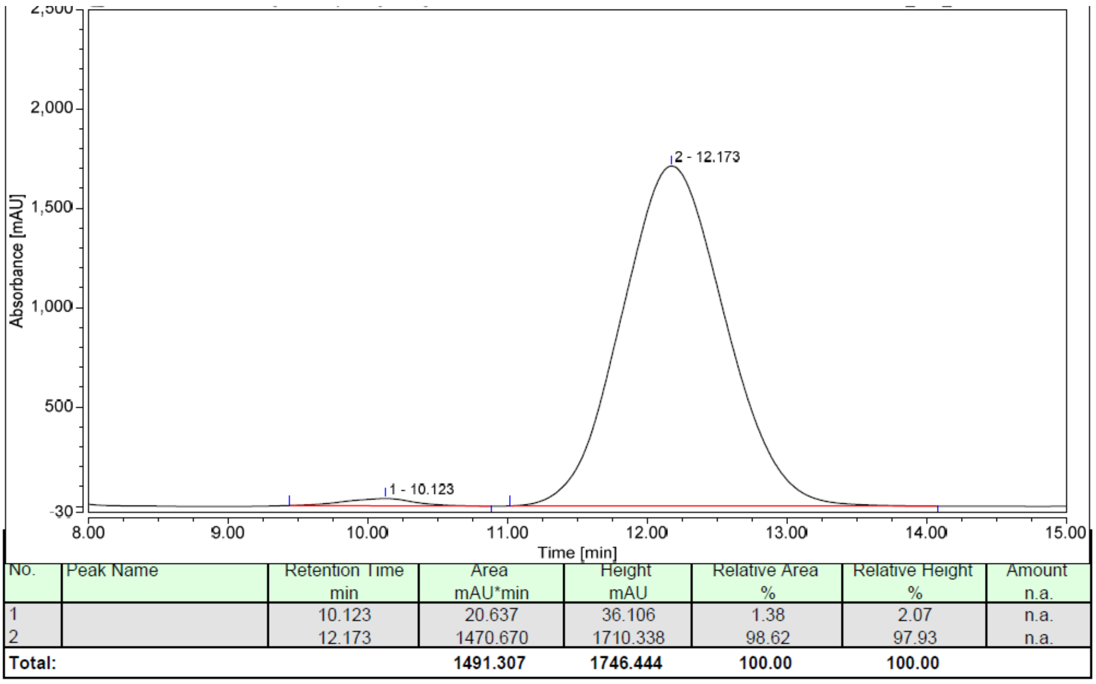

No.	Peak Name	Retention Time min	Area mAU*min	Height mAU	Relative Area %	Relative Height %	Amount n.a.
1		10.123	20.637	36.106	1.38	2.07	n.a.
2		12.173	1470.670	1710.338	98.62	97.93	n.a.
**Total:**			**1491.307**	**1746.444**	**100.00**	**100.00**	

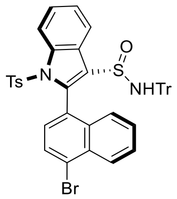

57b

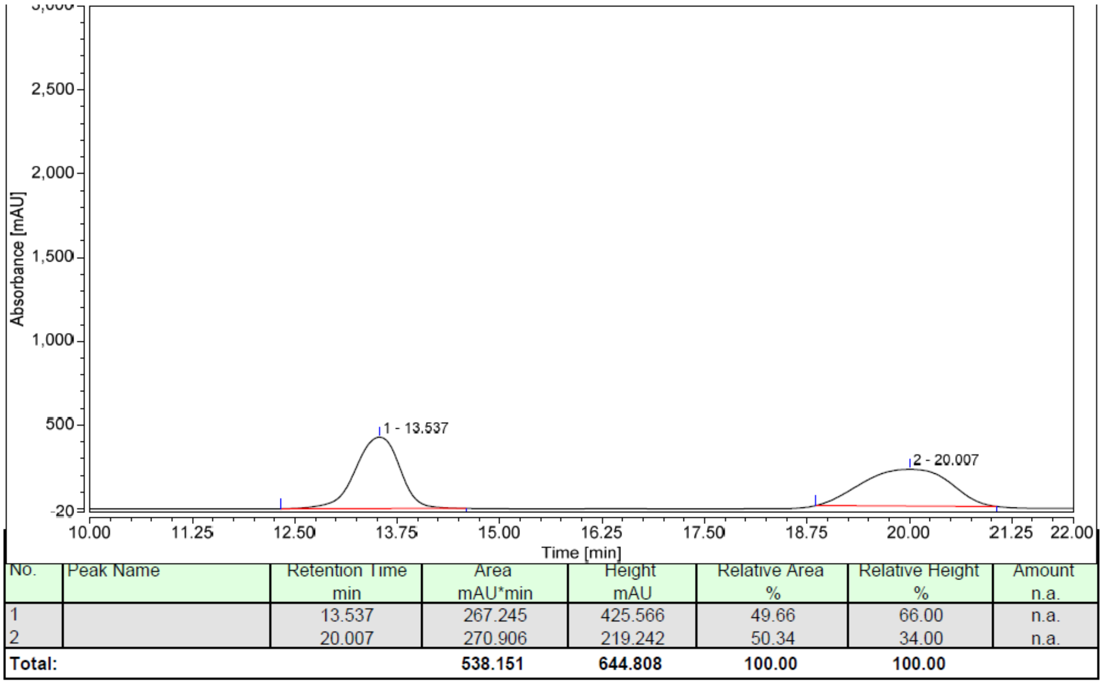

No.	Peak Name	Retention Time min	Area mAU*min	Height mAU	Relative Area %	Relative Height %	Amount n.a.
1		13.537	267.245	425.566	49.66	66.00	n.a.
2		20.007	270.906	219.242	50.34	34.00	n.a.
**Total:**			**538.151**	**644.808**	**100.00**	**100.00**	

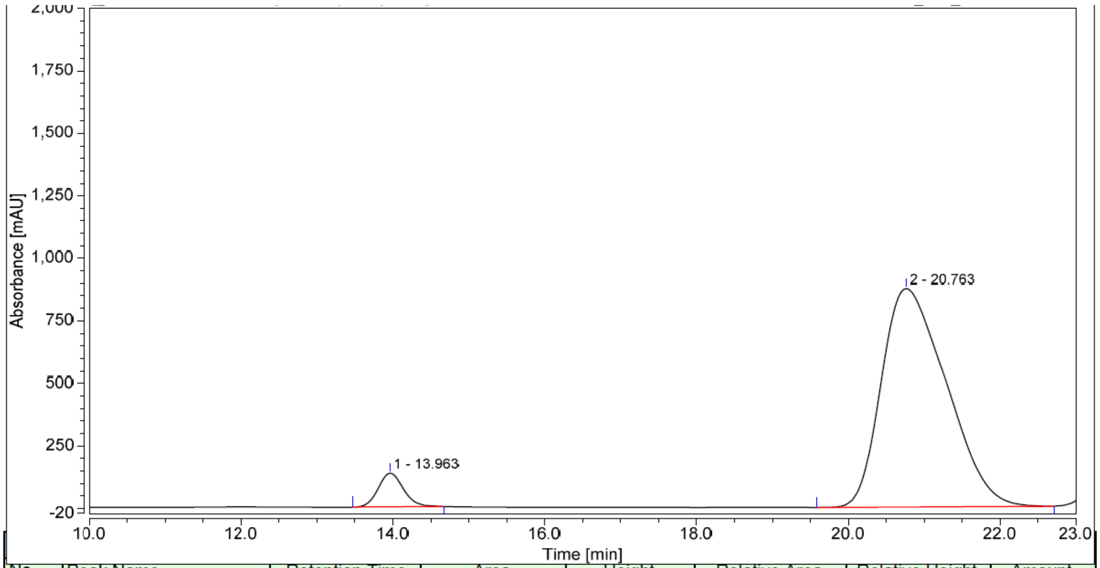

No.	Peak Name	Retention Time min	Area mAU*min	Height mAU	Relative Area %	Relative Height %	Amount n.a.
1		13.963	51.513	134.380	5.63	13.34	n.a.
2		20.763	863.871	872.779	94.37	86.66	n.a.
**Total:**			**915.384**	**1007.158**	**100.00**	**100.00**	

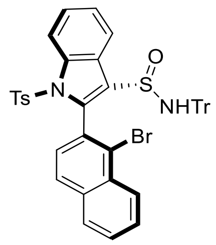

58b

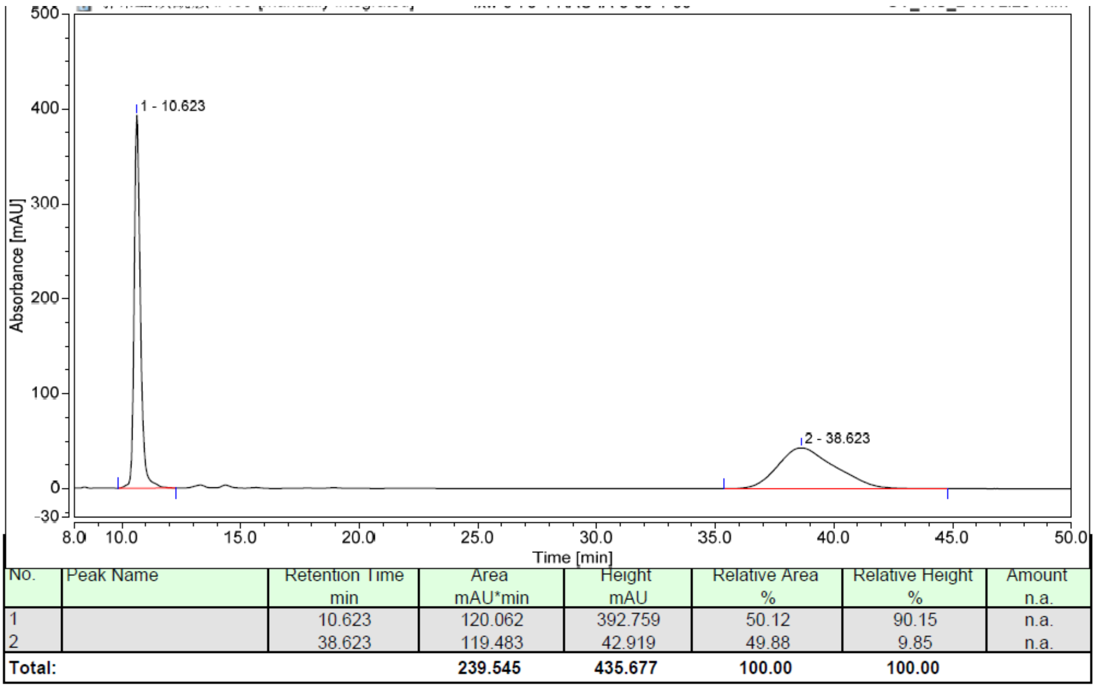

No.	Peak Name	Retention Time<br>min	Area<br>mAU*min	Height<br>mAU	Relative Area<br>%	Relative Height<br>%	Amount<br>n.a.
1		10.623	120.062	392.759	50.12	90.15	n.a.
2		38.623	119.483	42.919	49.88	9.85	n.a.
**Total:**			**239.545**	**435.677**	**100.00**	**100.00**	

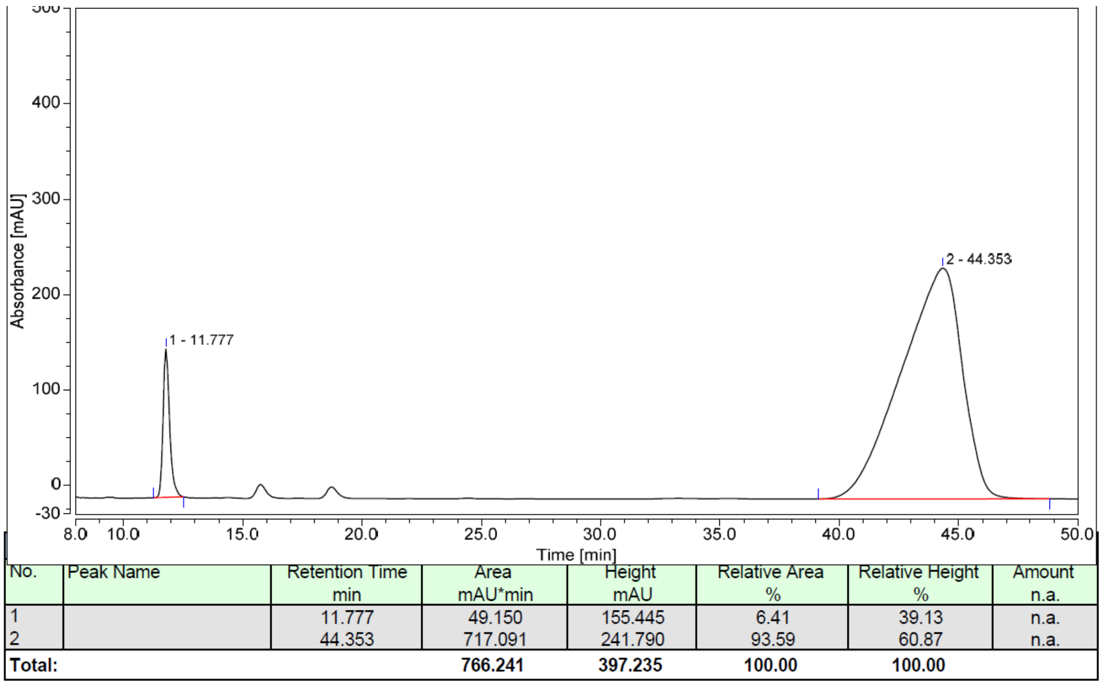

No.	Peak Name	Retention Time<br>min	Area<br>mAU*min	Height<br>mAU	Relative Area<br>%	Relative Height<br>%	Amount<br>n.a.
1		11.777	49.150	155.445	6.41	39.13	n.a.
2		44.353	717.091	241.790	93.59	60.87	n.a.
**Total:**			**766.241**	**397.235**	**100.00**	**100.00**	

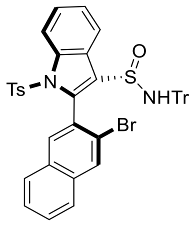

59b

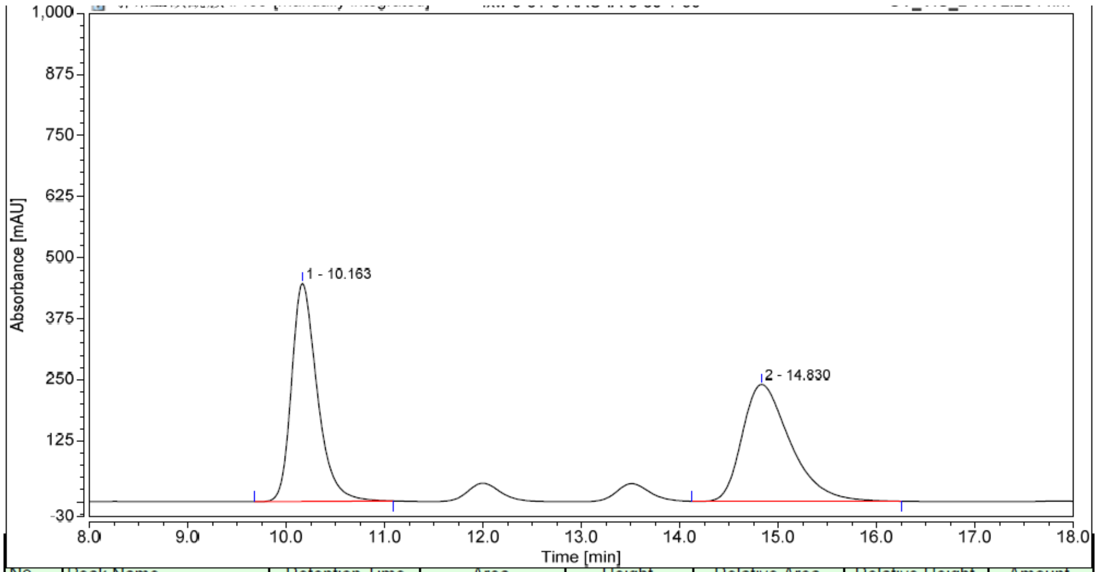

No.	Peak Name	Retention Time min	Area mAU*min	Height mAU	Relative Area %	Relative Height %	Amount n.a.
1		10.163	135.013	446.676	50.06	65.14	n.a.
2		14.830	134.705	239.044	49.94	34.86	n.a.
**Total:**			**269.719**	**685.720**	**100.00**	**100.00**	

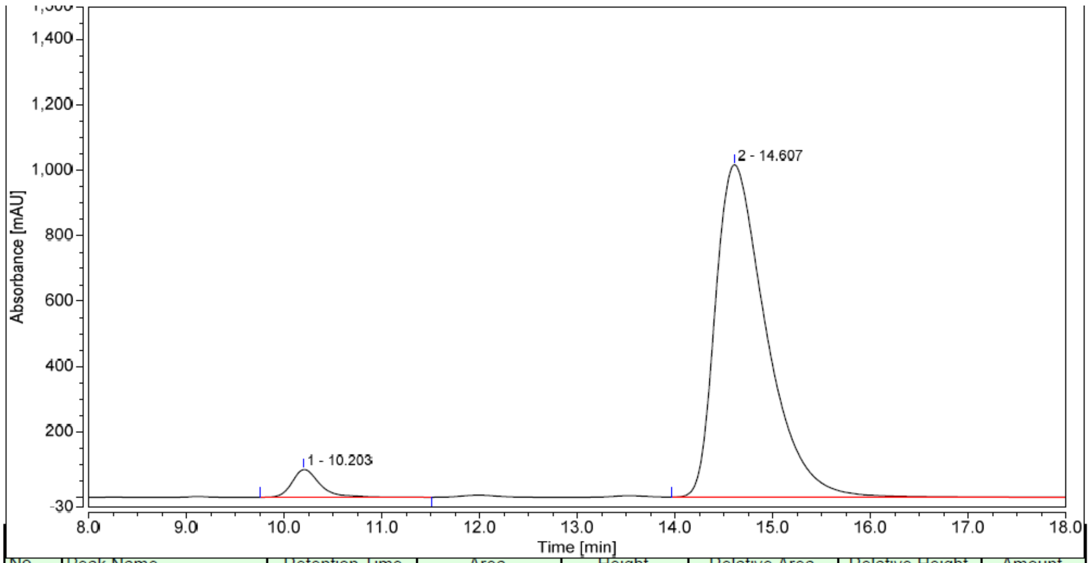

No.	Peak Name	Retention Time min	Area mAU*min	Height mAU	Relative Area %	Relative Height %	Amount n.a.
1		10.203	27.936	84.668	4.38	7.69	n.a.
2		14.607	610.007	1016.913	95.62	92.31	n.a.
**Total:**			**637.943**	**1101.582**	**100.00**	**100.00**	

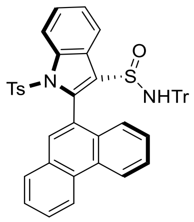

60b

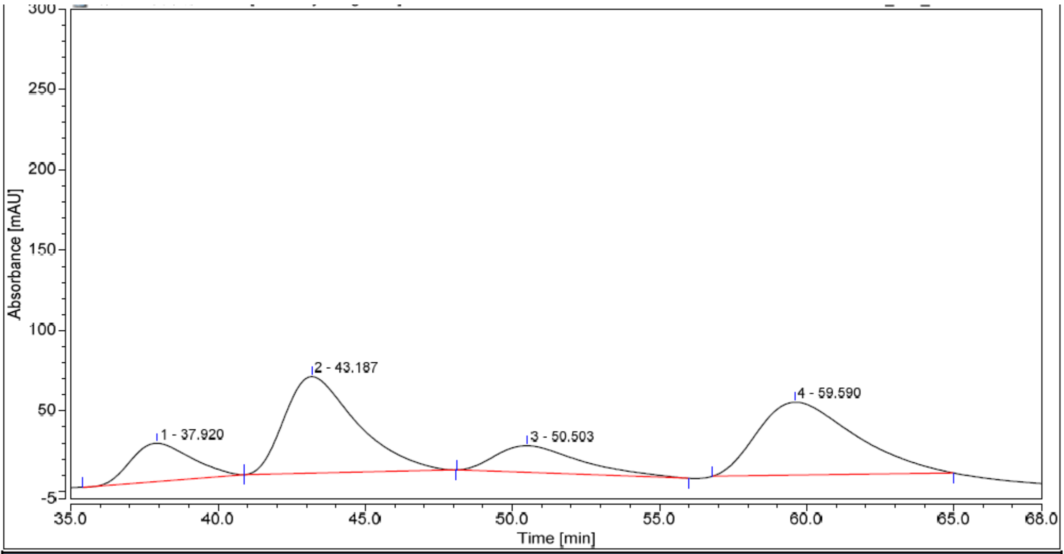

Integration Results							
No.	Peak Name	Retention Time min	Area mAU*min	Height mAU	Relative Area %	Relative Height %	Amount n.a.
1		37.920	56.013	23.774	12.27	16.29	n.a.
2		43.187	170.653	60.107	37.39	41.18	n.a.
3		50.503	55.515	16.681	12.16	11.43	n.a.
4		59.590	174.249	45.388	38.18	31.10	n.a.
**Total:**			**456.430**	**145.950**	**100.00**	**100.00**	

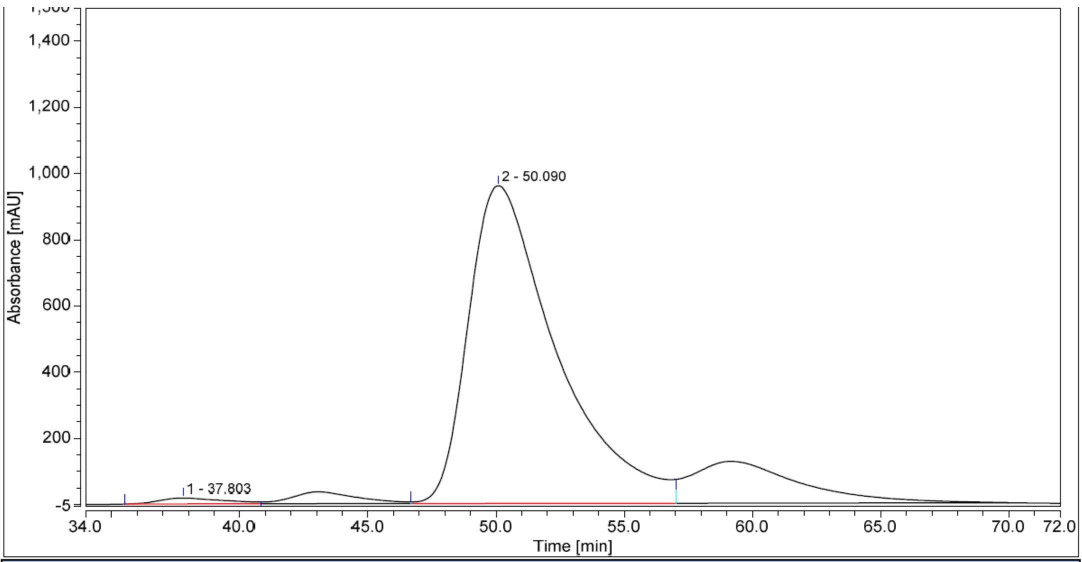

Integration Results							
No.	Peak Name	Retention Time min	Area mAU*min	Height mAU	Relative Area %	Relative Height %	Amount n.a.
1		37.803	52.635	18.161	1.36	1.86	n.a.
2		50.090	3814.401	959.852	98.64	98.14	n.a.
**Total:**			**3867.036**	**978.013**	**100.00**	**100.00**	

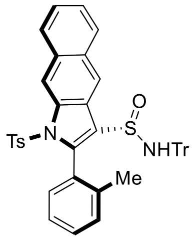

33b

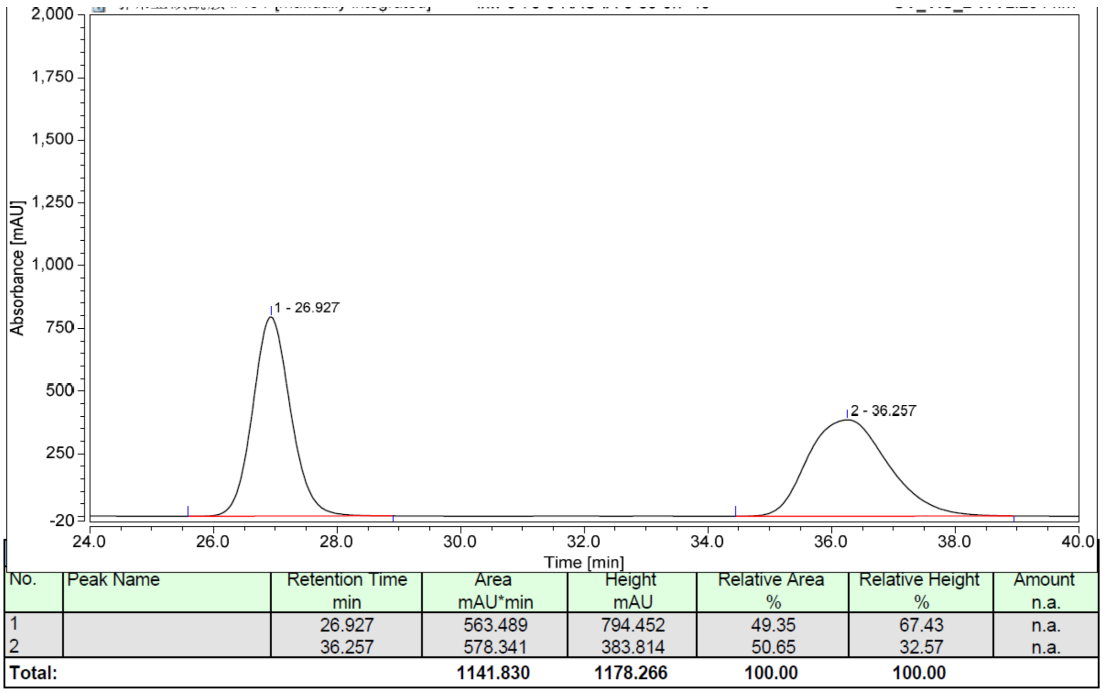

No.	Peak Name	Retention Time<br>min	Area<br>mAU*min	Height<br>mAU	Relative Area<br>%	Relative Height<br>%	Amount<br>n.a.
1		26.927	563.489	794.452	49.35	67.43	n.a.
2		36.257	578.341	383.814	50.65	32.57	n.a.
**Total:**			**1141.830**	**1178.266**	**100.00**	**100.00**	

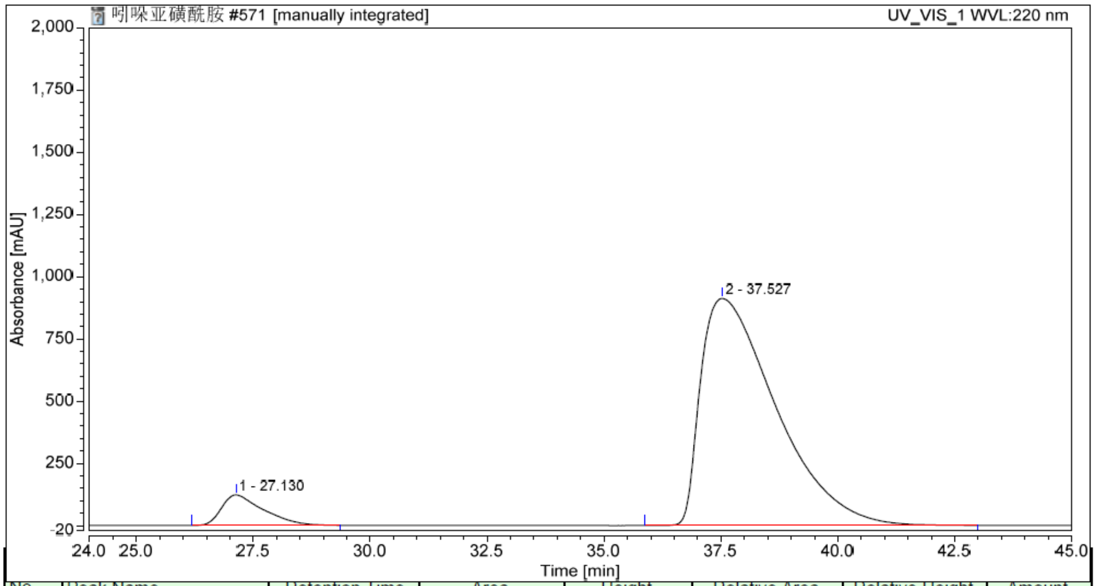

No.	Peak Name	Retention Time<br>min	Area<br>mAU*min	Height<br>mAU	Relative Area<br>%	Relative Height<br>%	Amount<br>n.a.
1		27.130	127.504	122.469	6.97	11.83	n.a.
2		37.527	1701.986	912.496	93.03	88.17	n.a.
**Total:**			**1829.490**	**1034.965**	**100.00**	**100.00**	

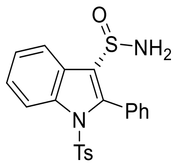

62

Chromatogram

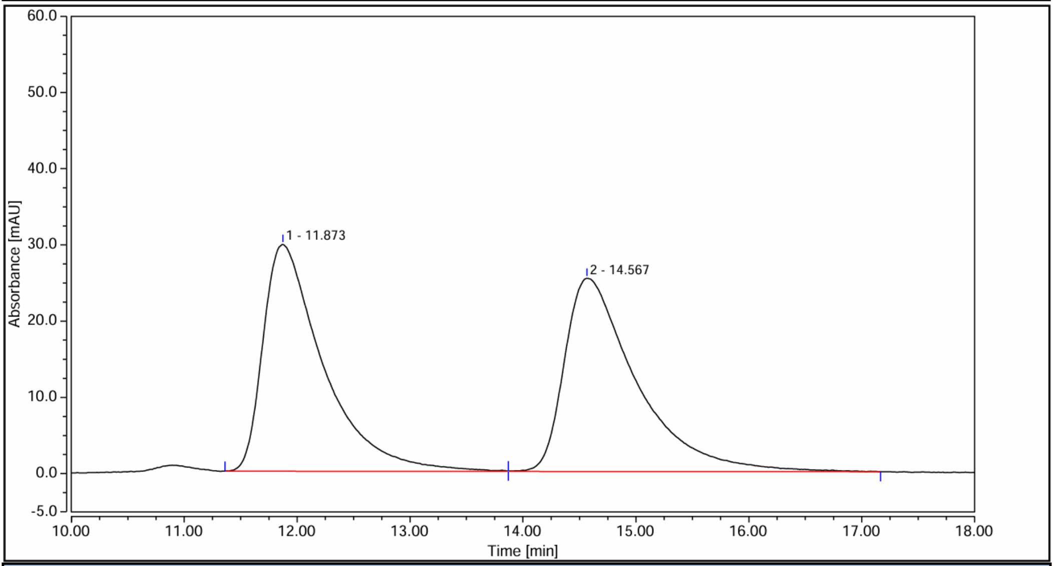

Integration Results

No.	Peak Name	Retention Time<br>min	Area<br>mAU*min	Height<br>mAU	Relative Area<br>%	Relative Height<br>%	Amount<br>n.a.
1		11.873	18.414	29.746	49.62	54.00	n.a.
2		14.567	18.697	25.339	50.38	46.00	n.a.
**Total:**			**37.111**	**55.085**	**100.00**	**100.00**	

Chromatogram

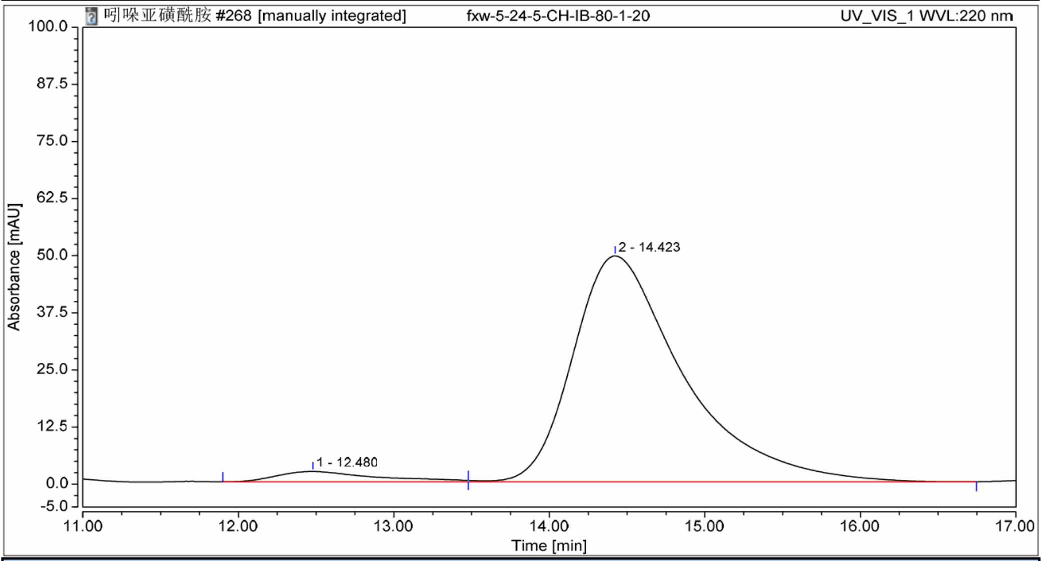

Integration Results

No.	Peak Name	Retention Time<br>min	Area<br>mAU*min	Height<br>mAU	Relative Area<br>%	Relative Height<br>%	Amount<br>n.a.
1		12.480	1.665	2.263	3.94	4.38	n.a.
2		14.423	40.605	49.443	96.06	95.62	n.a.
**Total:**			**42.270**	**51.706**	**100.00**	**100.00**	

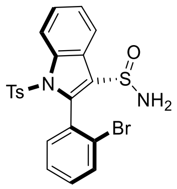

63

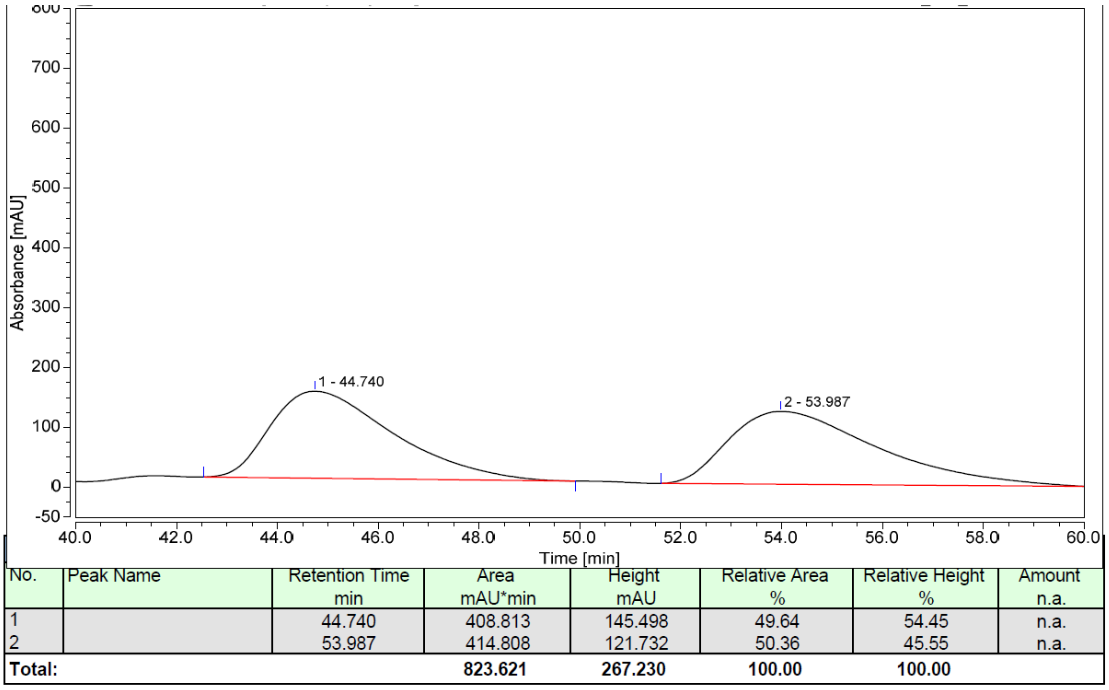

No.	Peak Name	Retention Time<br>min	Area<br>mAU*min	Height<br>mAU	Relative Area<br>%	Relative Height<br>%	Amount<br>n.a.
1		44.740	408.813	145.498	49.64	54.45	n.a.
2		53.987	414.808	121.732	50.36	45.55	n.a.
**Total:**			**823.621**	**267.230**	**100.00**	**100.00**	

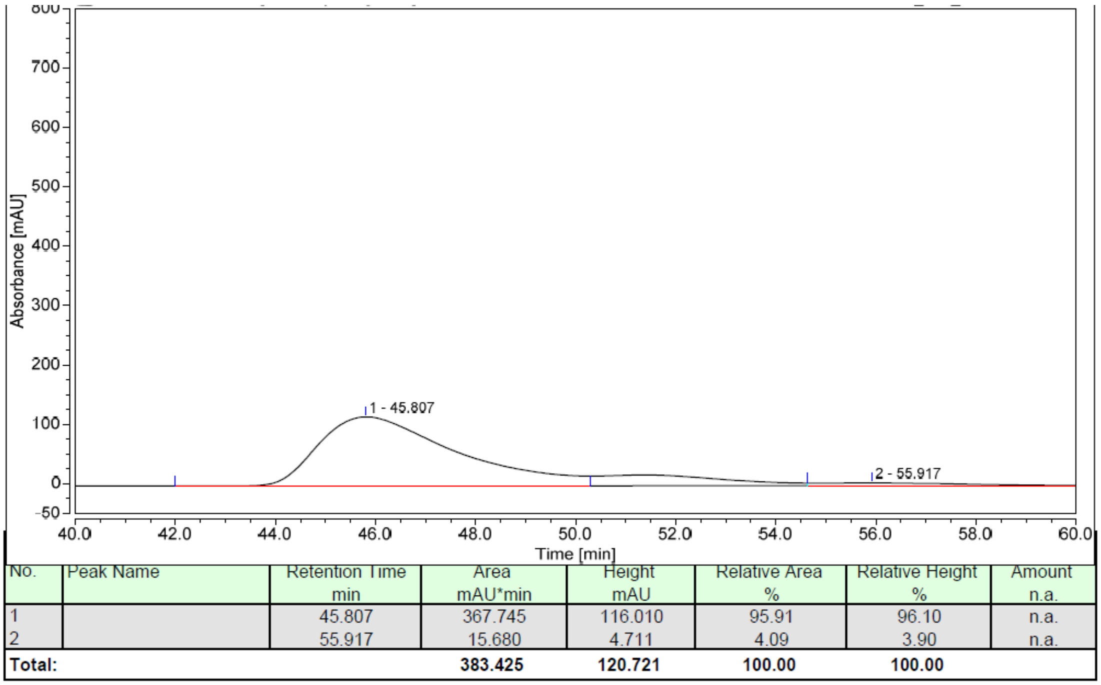

No.	Peak Name	Retention Time<br>min	Area<br>mAU*min	Height<br>mAU	Relative Area<br>%	Relative Height<br>%	Amount<br>n.a.
1		45.807	367.745	116.010	95.91	96.10	n.a.
2		55.917	15.680	4.711	4.09	3.90	n.a.
**Total:**			**383.425**	**120.721**	**100.00**	**100.00**	

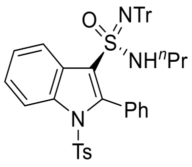

64

Chromatogram

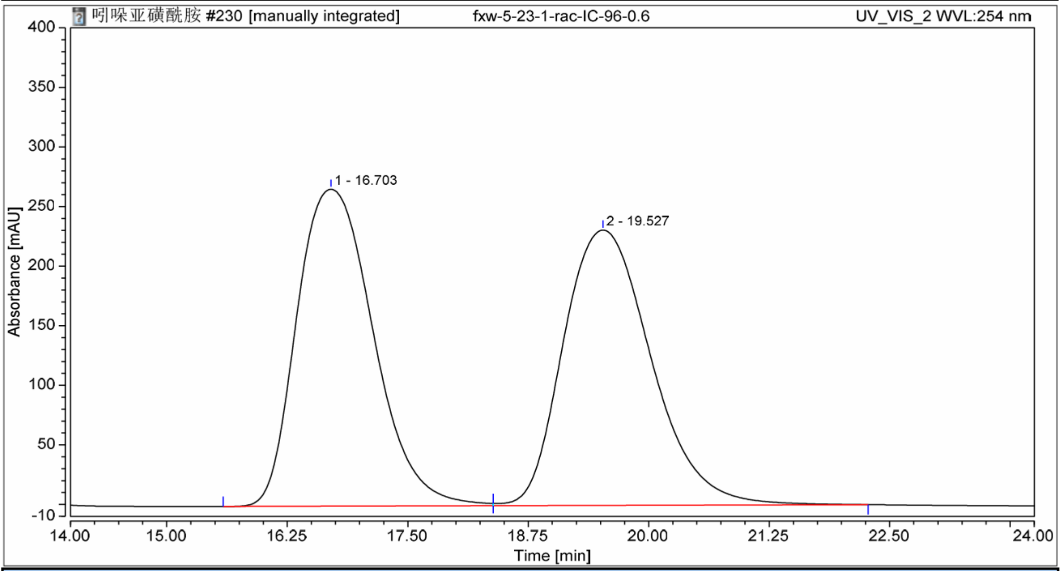

Integration Results

No.	Peak Name	Retention Time min	Area mAU*min	Height mAU	Relative Area %	Relative Height %	Amount n.a.
1		16.703	244.298	266.034	50.04	53.51	n.a.
2		19.527	243.935	231.105	49.96	46.49	n.a.
**Total:**			**488.233**	**497.140**	**100.00**	**100.00**	

Chromatogram

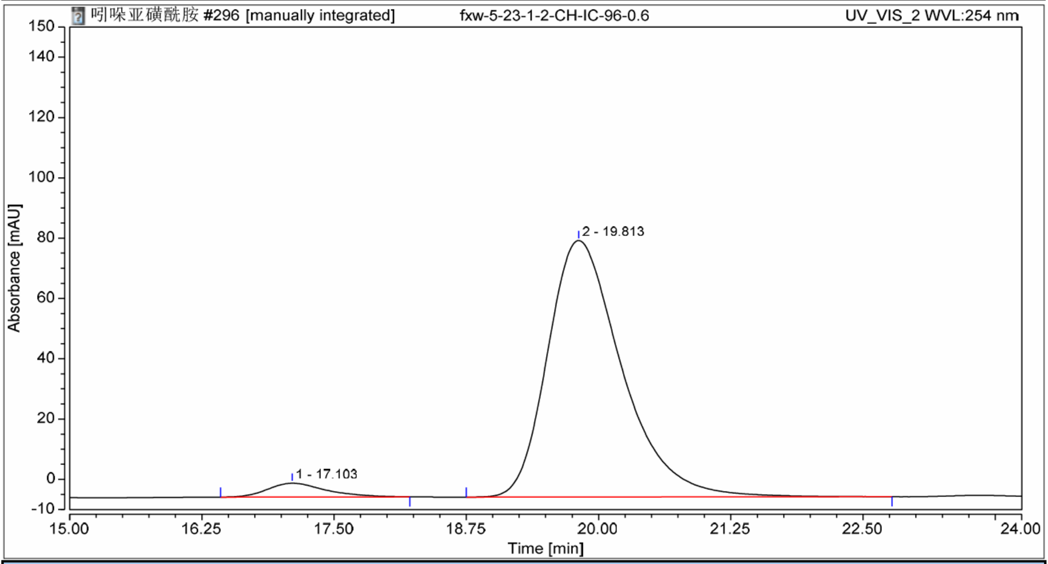

Integration Results

No.	Peak Name	Retention Time min	Area mAU*min	Height mAU	Relative Area %	Relative Height %	Amount n.a.
1		17.103	2.910	4.559	4.04	5.09	n.a.
2		19.813	69.172	85.080	95.96	94.91	n.a.
**Total:**			**72.082**	**89.638**	**100.00**	**100.00**	

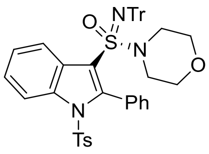

65

Chromatogram

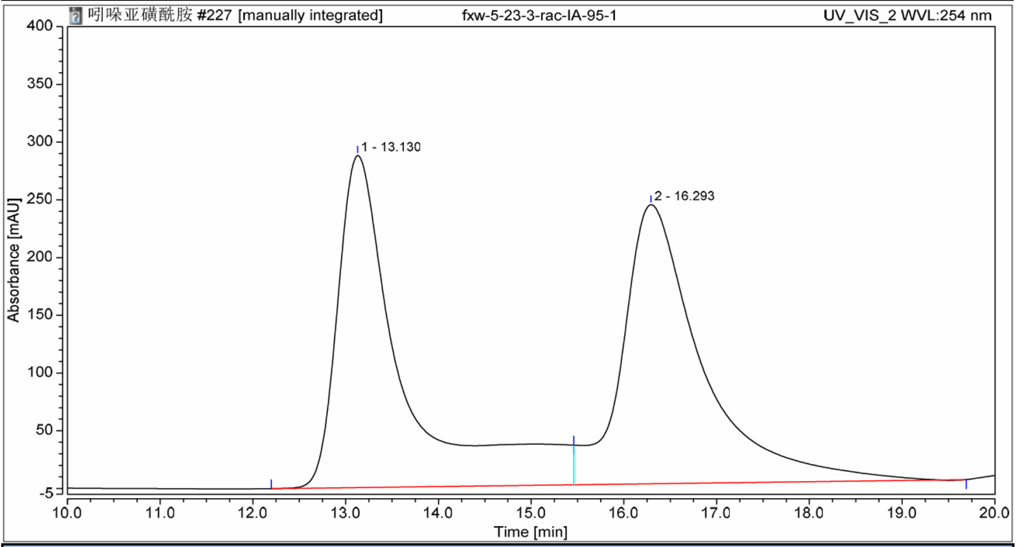

Integration Results

No.	Peak Name	Retention Time min	Area mAU*min	Height mAU	Relative Area %	Relative Height %	Amount n.a.
1		13.130	234.597	287.653	49.48	54.34	n.a.
2		16.293	239.559	241.686	50.52	45.66	n.a.
**Total:**			**474.156**	**529.338**	**100.00**	**100.00**	

Chromatogram

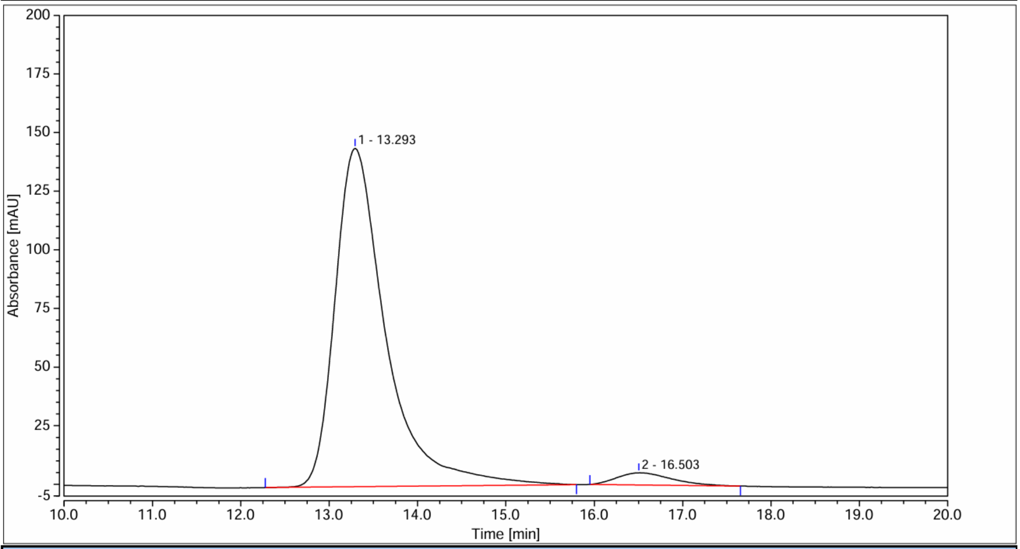

Integration Results

No.	Peak Name	Retention Time min	Area mAU*min	Height mAU	Relative Area %	Relative Height %	Amount n.a.
1		13.293	96.088	144.250	96.30	96.54	n.a.
2		16.503	3.692	5.165	3.70	3.46	n.a.
**Total:**			**99.780**	**149.415**	**100.00**	**100.00**	

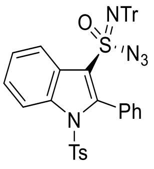

66

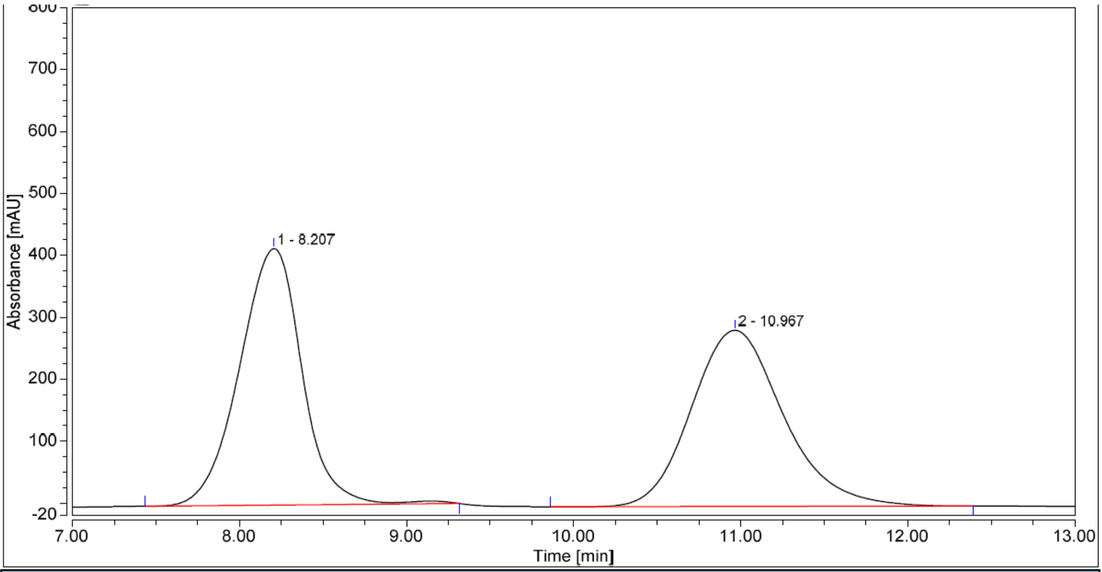

Integration Results

No.	Peak Name	Retention Time min	Area mAU*min	Height mAU	Relative Area %	Relative Height %	Amount n.a.
1		8.207	179.731	414.151	49.75	59.29	n.a.
2		10.967	181.553	284.330	50.25	40.71	n.a.
**Total:**			**361.284**	**698.482**	**100.00**	**100.00**	

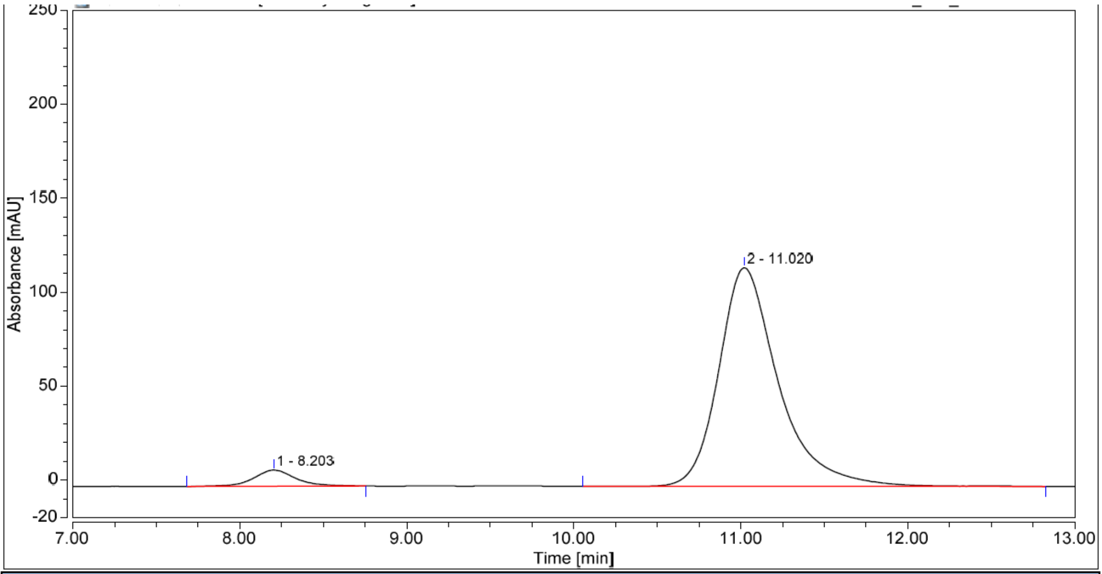

Integration Results

No.	Peak Name	Retention Time min	Area mAU*min	Height mAU	Relative Area %	Relative Height %	Amount n.a.
1		8.203	2.486	8.571	4.92	6.87	n.a.
2		11.020	48.066	116.262	95.08	93.13	n.a.
**Total:**			**50.551**	**124.834**	**100.00**	**100.00**	

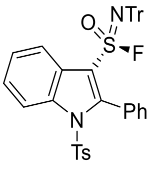

67

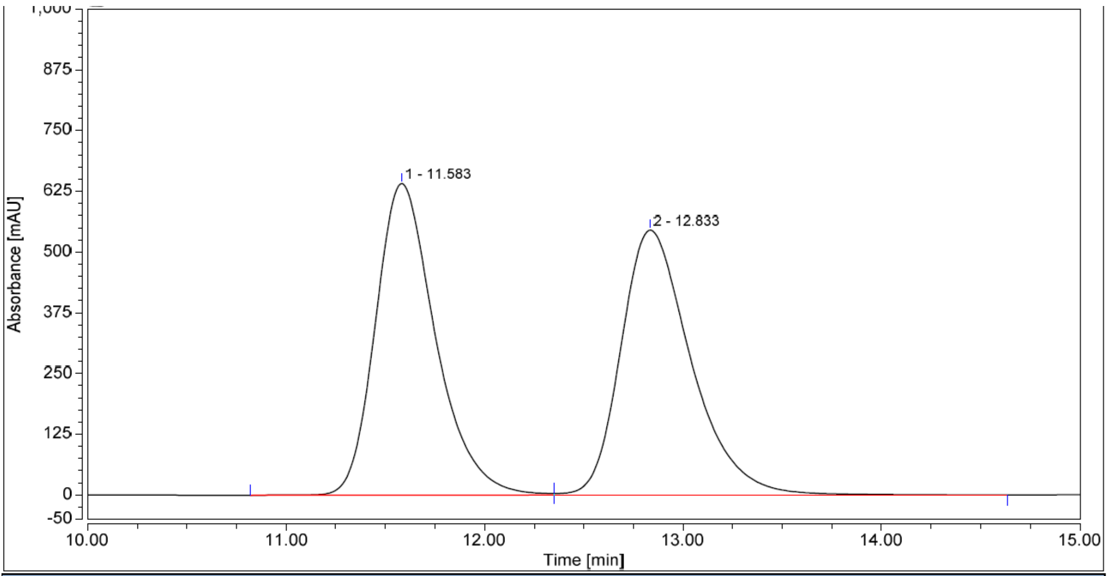

Integration Results							
No.	Peak Name	Retention Time<br>min	Area<br>mAU*min	Height<br>mAU	Relative Area<br>%	Relative Height<br>%	Amount<br>n.a.
1		11.583	224.649	641.326	50.12	54.05	n.a.
2		12.833	223.529	545.210	49.88	45.95	n.a.
**Total:**			**448.177**	**1186.536**	**100.00**	**100.00**	

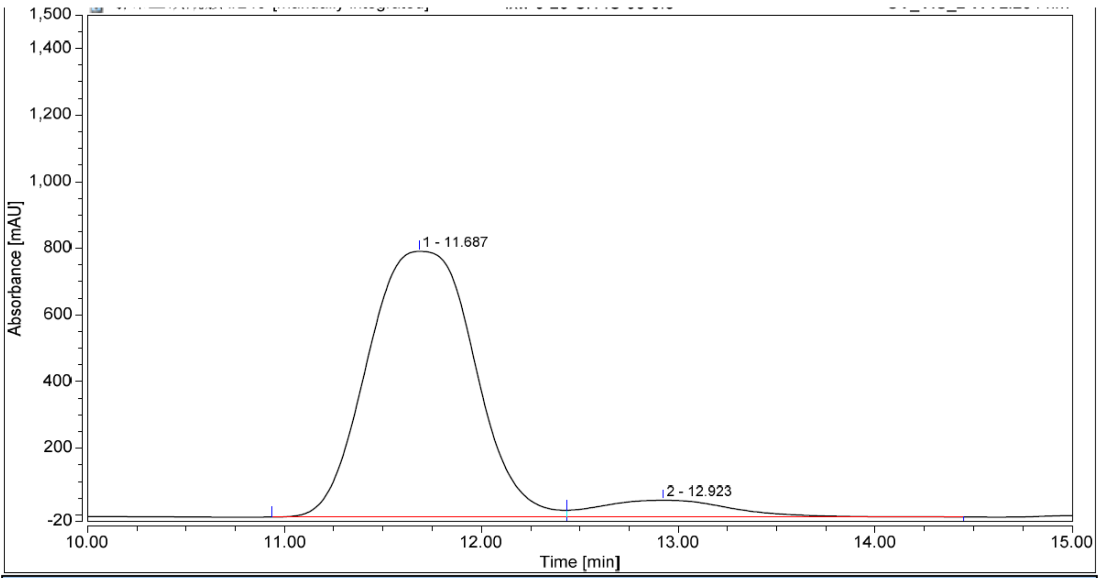

Integration Results							
No.	Peak Name	Retention Time<br>min	Area<br>mAU*min	Height<br>mAU	Relative Area<br>%	Relative Height<br>%	Amount<br>n.a.
1		11.687	499.246	797.843	92.69	94.04	n.a.
2		12.923	39.375	50.574	7.31	5.96	n.a.
**Total:**			**538.621**	**848.416**	**100.00**	**100.00**	

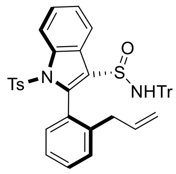

68

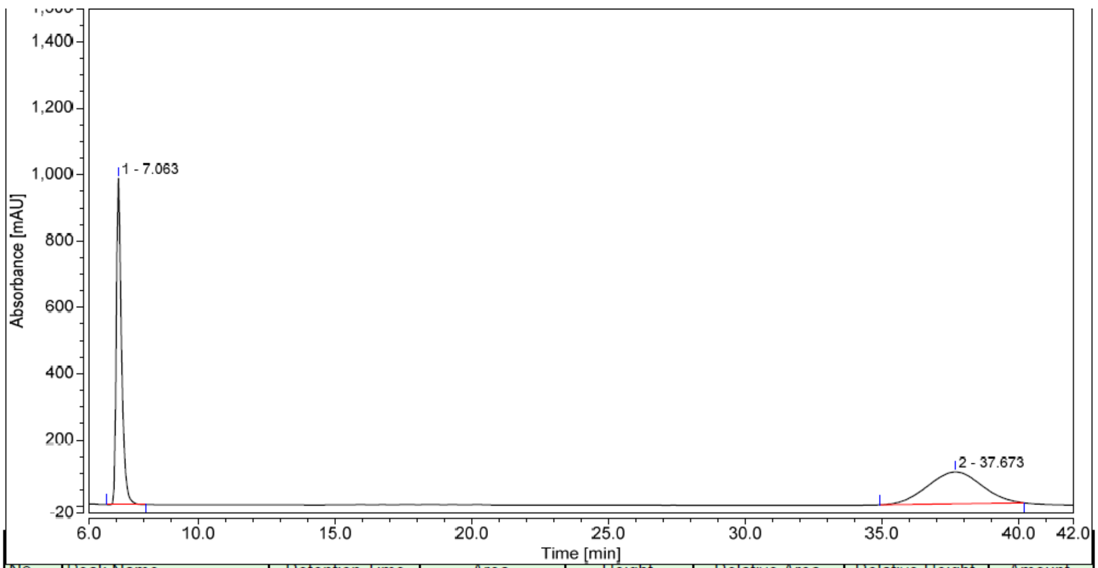

No.	Peak Name	Retention Time min	Area mAU*min	Height mAU	Relative Area %	Relative Height %	Amount n.a.
1		7.063	216.121	984.222	49.47	91.12	n.a.
2		37.673	220.715	95.969	50.53	8.88	n.a.
**Total:**			**436.836**	**1080.190**	**100.00**	**100.00**	

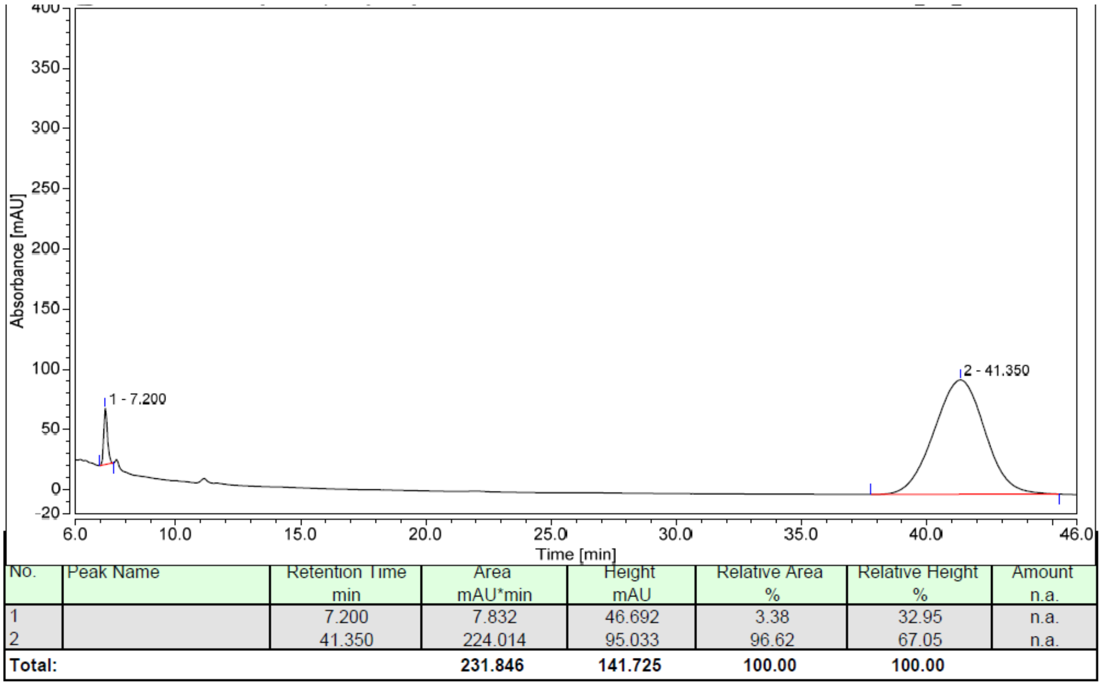

No.	Peak Name	Retention Time min	Area mAU*min	Height mAU	Relative Area %	Relative Height %	Amount n.a.
1		7.200	7.832	46.692	3.38	32.95	n.a.
2		41.350	224.014	95.033	96.62	67.05	n.a.
**Total:**			**231.846**	**141.725**	**100.00**	**100.00**	

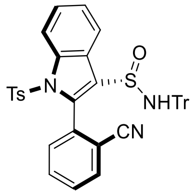

69

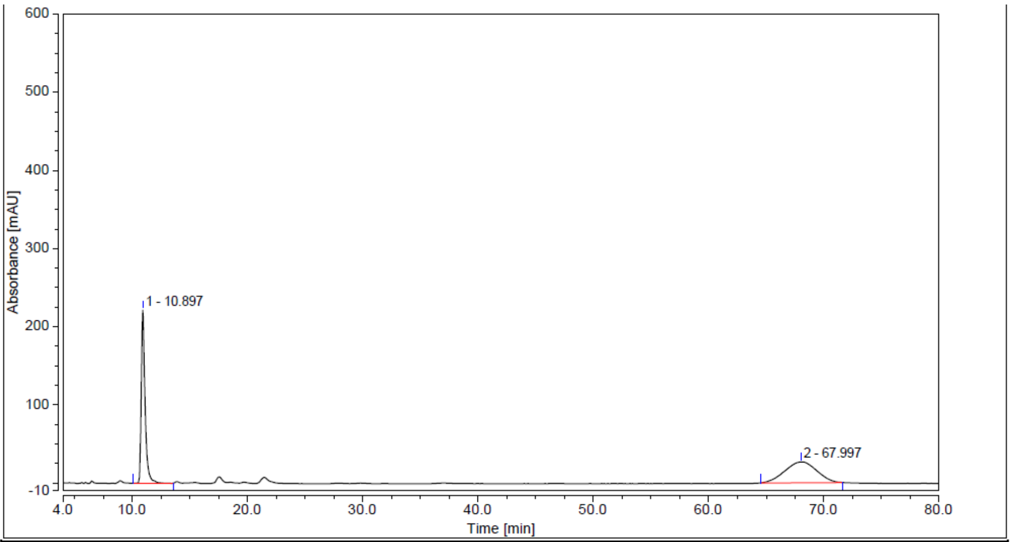

Integration Results

No.	Peak Name	Retention Time min	Area mAU*min	Height mAU	Relative Area %	Relative Height %	Amount n.a.
1		10.897	83.189	221.647	49.24	89.23	n.a.
2		67.997	85.748	26.763	50.76	10.77	n.a.
**Total:**			**168.937**	**248.410**	**100.00**	**100.00**	

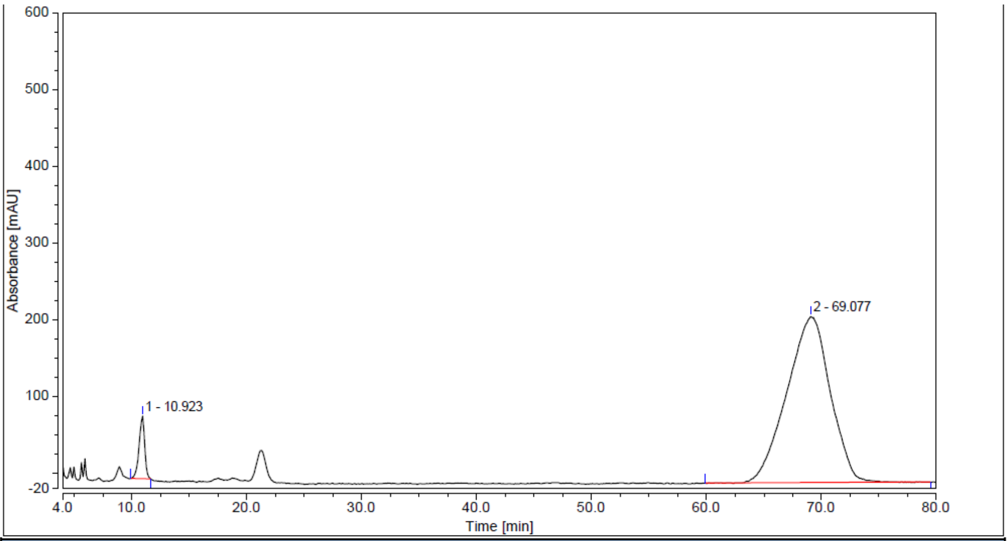

Integration Results

No.	Peak Name	Retention Time min	Area mAU*min	Height mAU	Relative Area %	Relative Height %	Amount n.a.
1		10.923	46.119	82.395	4.65	27.53	n.a.
2		69.077	945.047	216.904	95.35	72.47	n.a.
**Total:**			**991.165**	**299.299**	**100.00**	**100.00**	

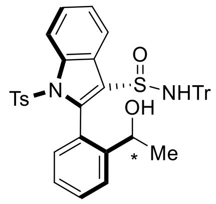

70

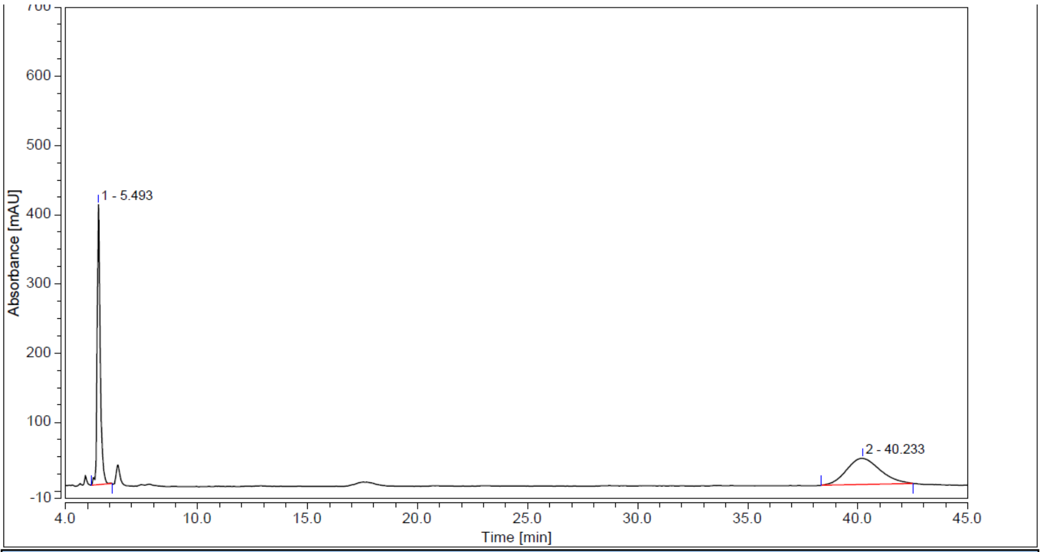

Integration Results							
No.	Peak Name	Retention Time min	Area mAU*min	Height mAU	Relative Area %	Relative Height %	Amount n.a.
1		5.493	65.474	404.547	49.53	91.47	n.a.
2		40.233	66.717	37.737	50.47	8.53	n.a.
**Total:**			**132.190**	**442.283**	**100.00**	**100.00**	

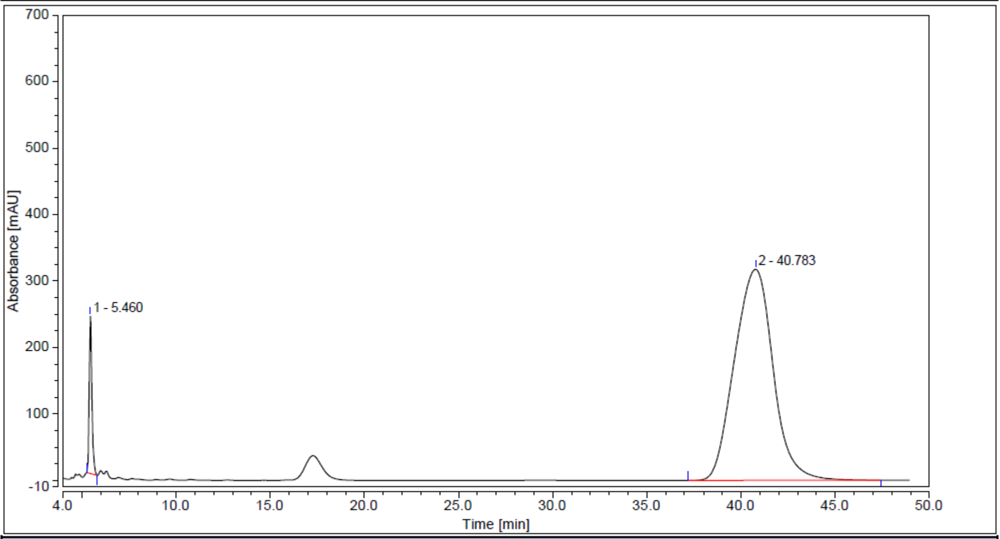

Integration Results							
No.	Peak Name	Retention Time min	Area mAU*min	Height mAU	Relative Area %	Relative Height %	Amount n.a.
1		5.460	39.531	236.841	4.98	42.70	n.a.
2		40.783	754.521	317.836	95.02	57.30	n.a.
**Total:**			**794.052**	**554.676**	**100.00**	**100.00**	

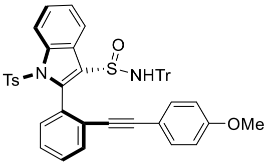

71

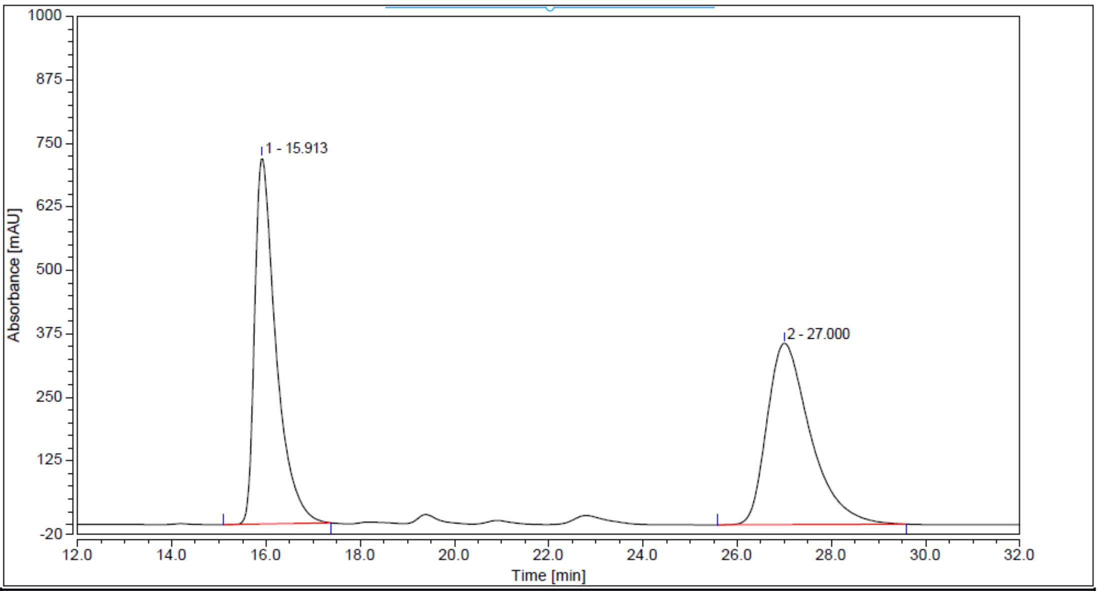

Integration Results							
No.	Peak Name	Retention Time min	Area mAU*min	Height mAU	Relative Area %	Relative Height %	Amount n.a.
1		15.913	378.094	720.740	49.81	66.88	n.a.
2		27.000	381.014	356.885	50.19	33.12	n.a.
**Total:**			**759.108**	**1077.625**	**100.00**	**100.00**	

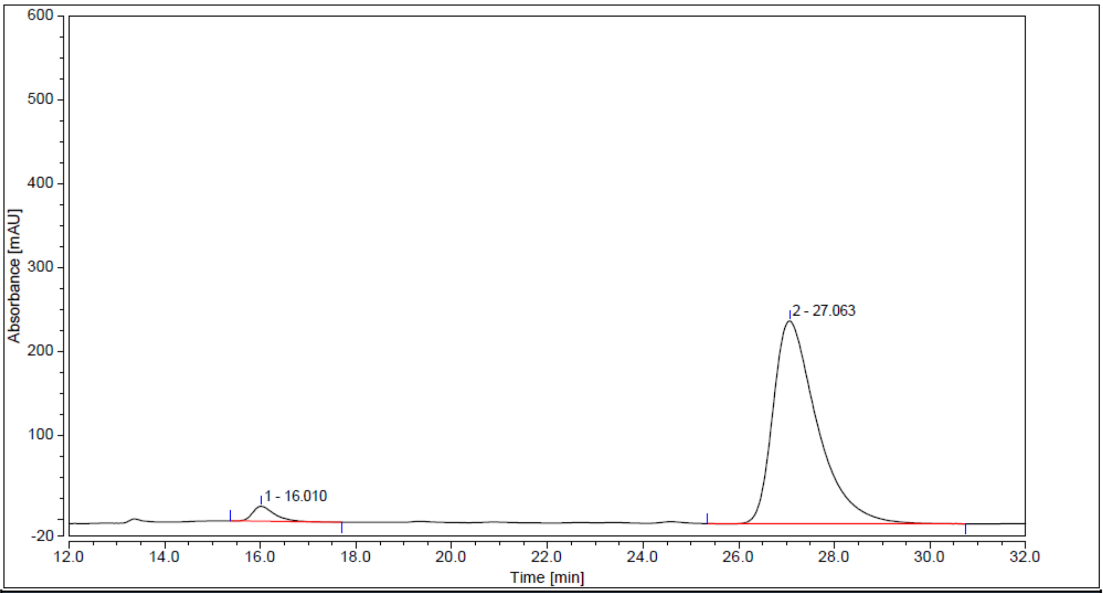

Integration Results							
No.	Peak Name	Retention Time min	Area mAU*min	Height mAU	Relative Area %	Relative Height %	Amount n.a.
1		16.010	9.463	17.873	3.54	6.89	n.a.
2		27.063	257.511	241.507	96.46	93.11	n.a.
**Total:**			**266.974**	**259.380**	**100.00**	**100.00**	

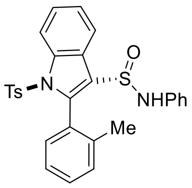

32c

major

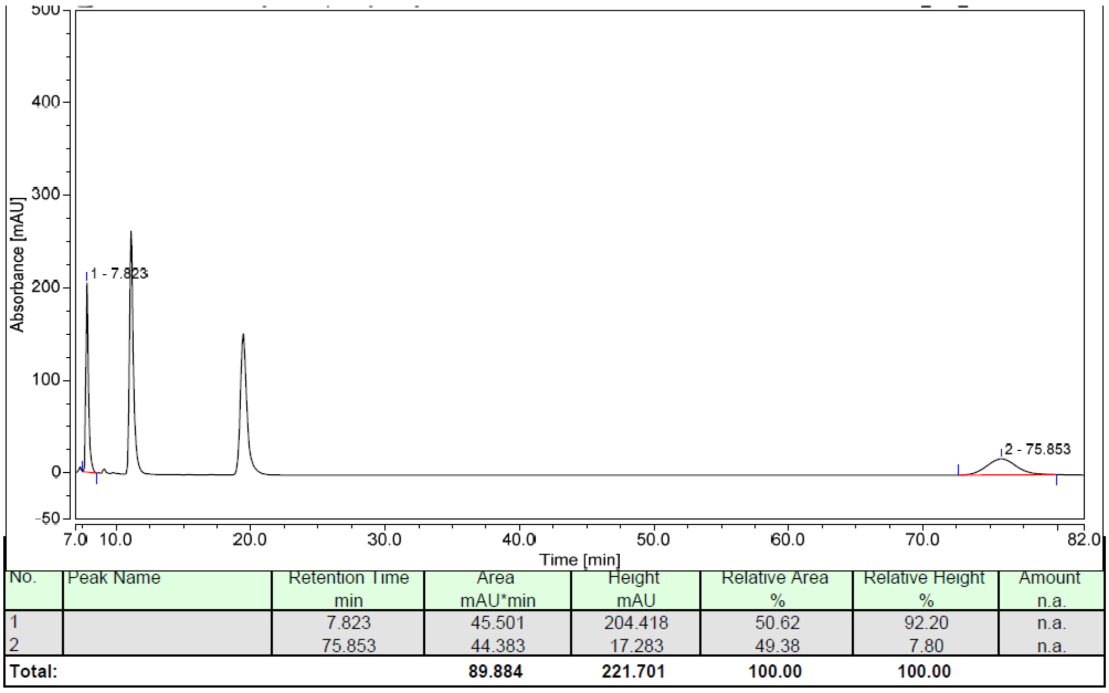

No.	Peak Name	Retention Time min	Area mAU*min	Height mAU	Relative Area %	Relative Height %	Amount n.a.
1		7.823	45.501	204.418	50.62	92.20	n.a.
2		75.853	44.383	17.283	49.38	7.80	n.a.
**Total:**			**89.884**	**221.701**	**100.00**	**100.00**	

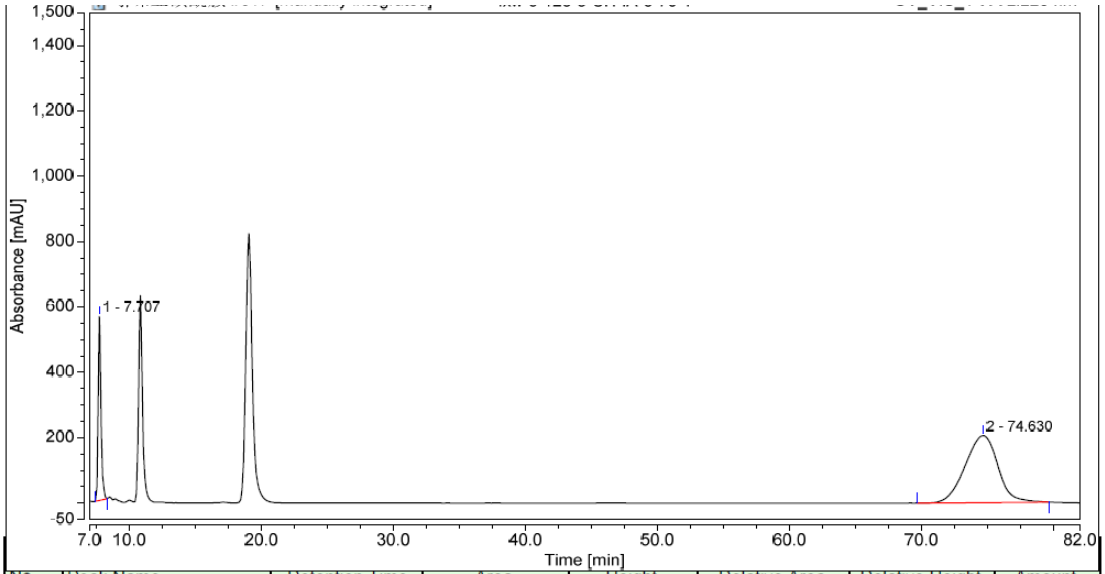

No.	Peak Name	Retention Time min	Area mAU*min	Height mAU	Relative Area %	Relative Height %	Amount n.a.
1		7.707	136.264	562.928	18.82	73.36	n.a.
2		74.630	587.814	204.459	81.18	26.64	n.a.
**Total:**			**724.078**	**767.387**	**100.00**	**100.00**	

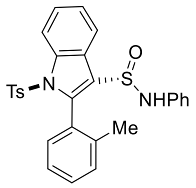

32c

minor

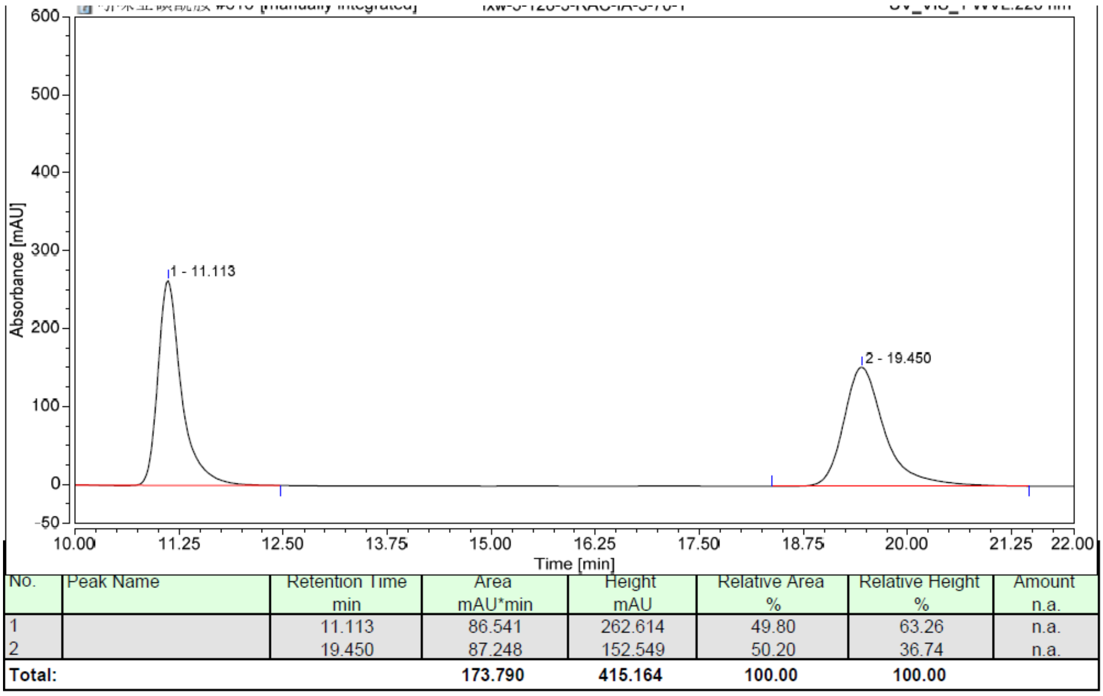

No.	Peak Name	Retention Time min	Area mAU*min	Height mAU	Relative Area %	Relative Height %	Amount n.a.
1		11.113	86.541	262.614	49.80	63.26	n.a.
2		19.450	87.248	152.549	50.20	36.74	n.a.
**Total:**			**173.790**	**415.164**	**100.00**	**100.00**	

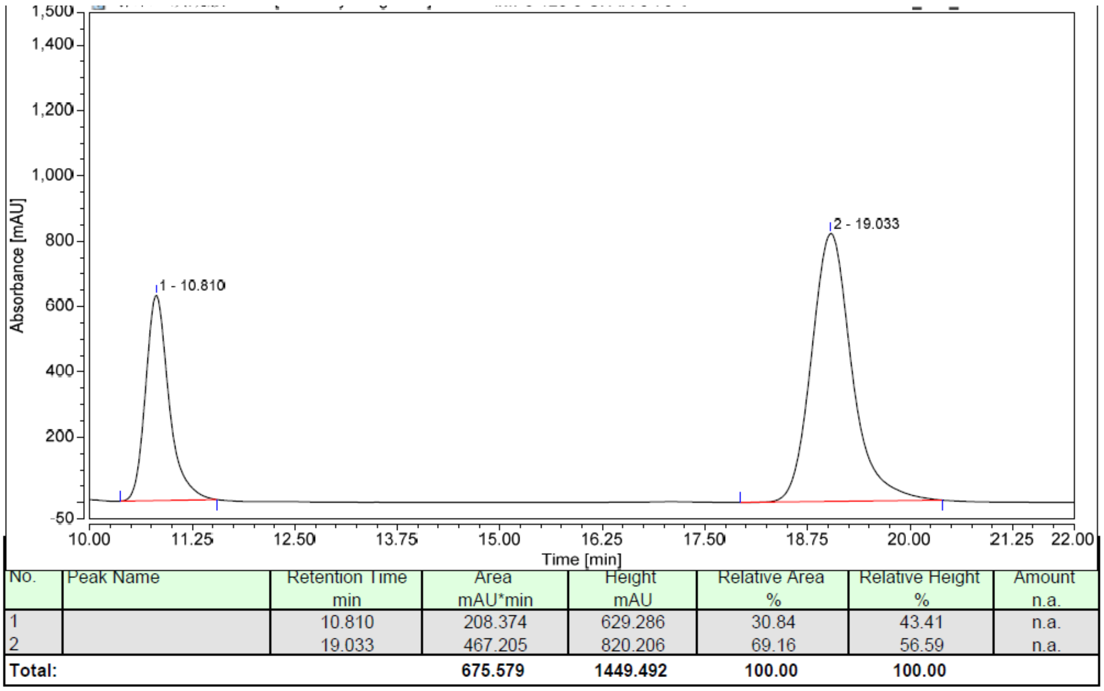

No.	Peak Name	Retention Time min	Area mAU*min	Height mAU	Relative Area %	Relative Height %	Amount n.a.
1		10.810	208.374	629.286	30.84	43.41	n.a.
2		19.033	467.205	820.206	69.16	56.59	n.a.
**Total:**			**675.579**	**1449.492**	**100.00**	**100.00**	

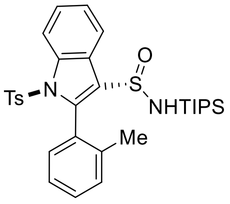

32e

major

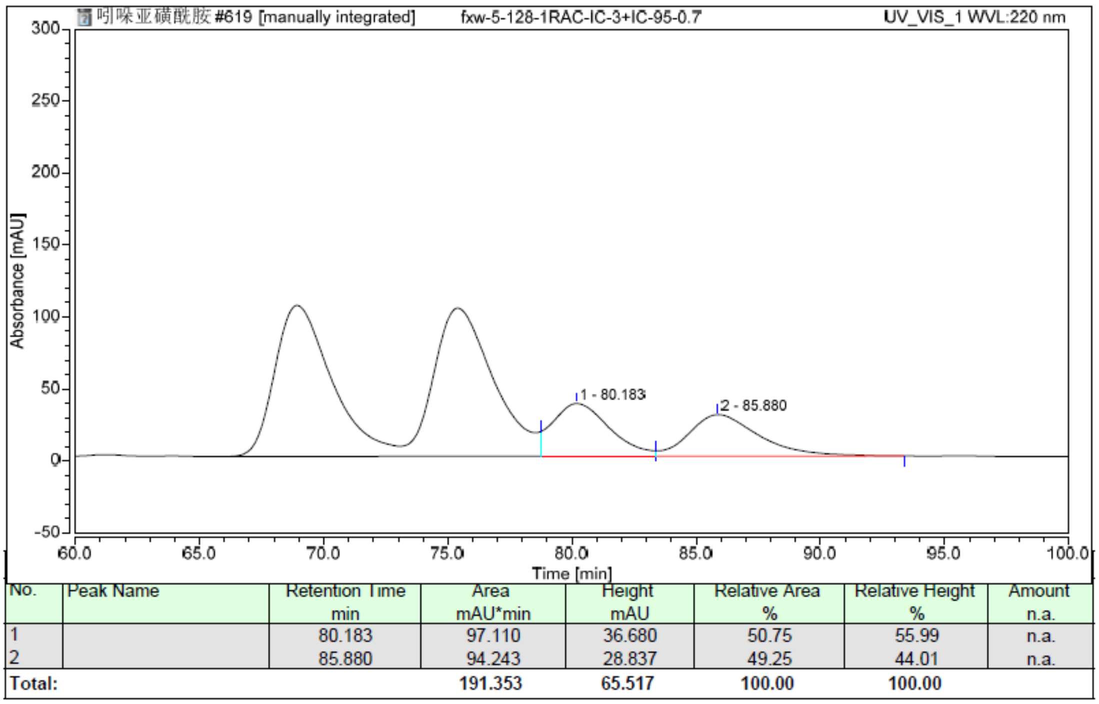

No.	Peak Name	Retention Time min	Area mAU*min	Height mAU	Relative Area %	Relative Height %	Amount n.a.
1		80.183	97.110	36.680	50.75	55.99	n.a.
2		85.880	94.243	28.837	49.25	44.01	n.a.
**Total:**			**191.353**	**65.517**	**100.00**	**100.00**	

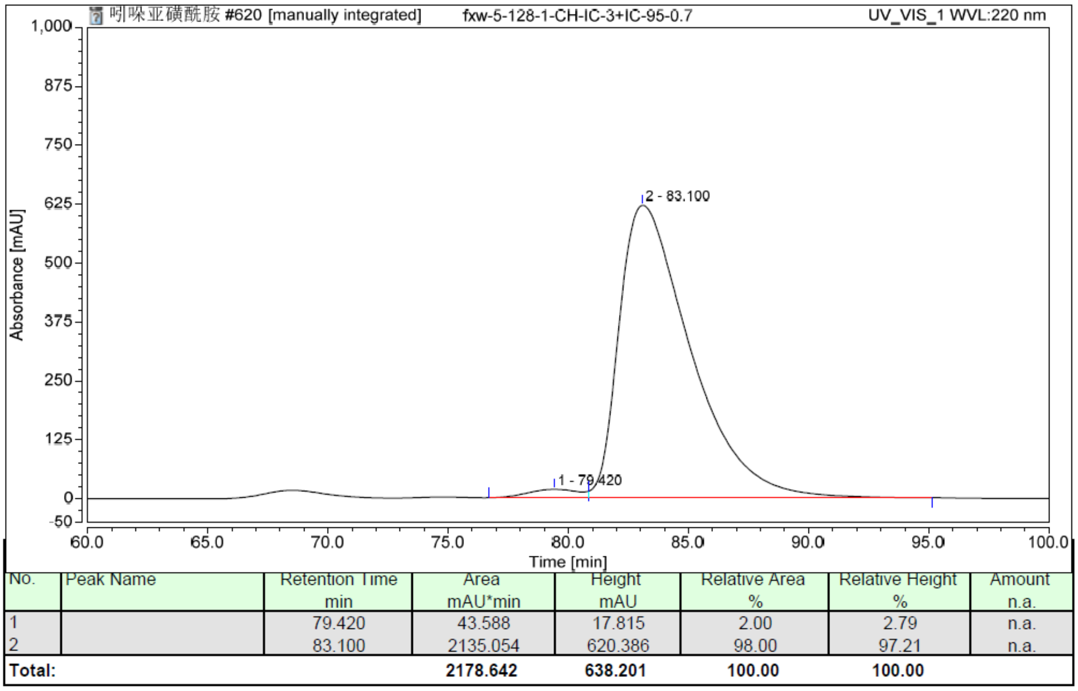

No.	Peak Name	Retention Time min	Area mAU*min	Height mAU	Relative Area %	Relative Height %	Amount n.a.
1		79.420	43.588	17.815	2.00	2.79	n.a.
2		83.100	2135.054	620.386	98.00	97.21	n.a.
**Total:**			**2178.642**	**638.201**	**100.00**	**100.00**	

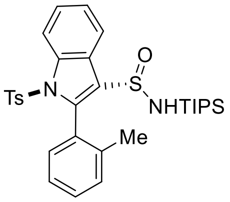

32e

Minor

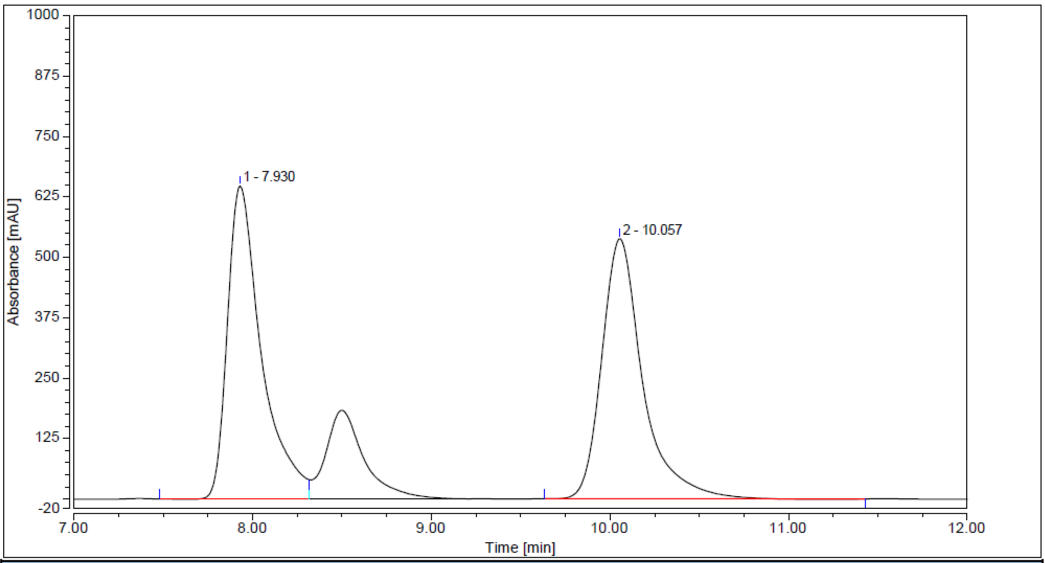

Integration Results							
No.	Peak Name	Retention Time min	Area mAU*min	Height mAU	Relative Area %	Relative Height %	Amount n.a.
1		7.930	142.431	647.542	50.00	54.62	n.a.
2		10.057	142.420	538.077	50.00	45.38	n.a.
**Total:**			**284.852**	**1185.620**	**100.00**	**100.00**	

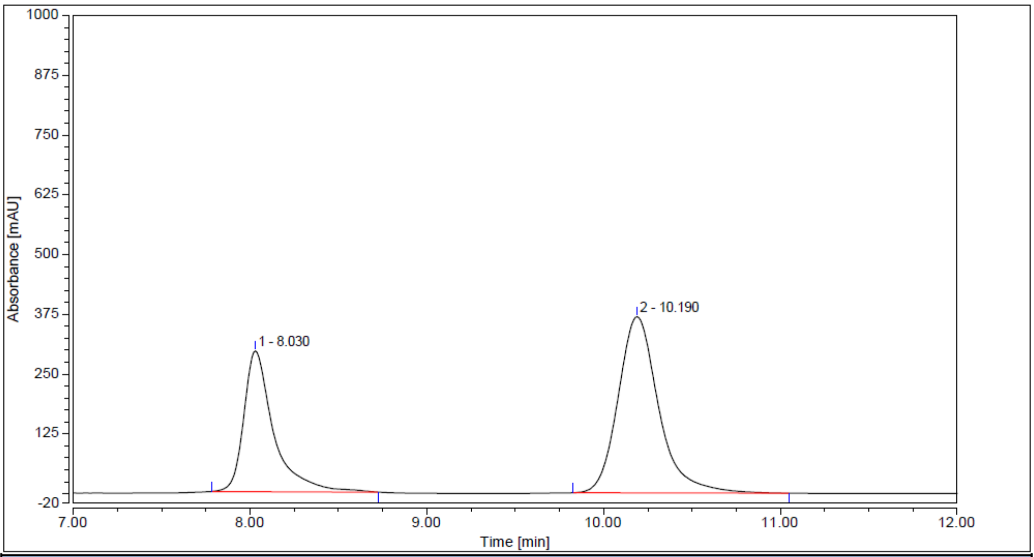

Integration Results							
No.	Peak Name	Retention Time min	Area mAU*min	Height mAU	Relative Area %	Relative Height %	Amount n.a.
1		8.030	57.636	294.400	37.05	44.42	n.a.
2		10.190	97.925	368.363	62.95	55.58	n.a.
**Total:**			**155.561**	**662.763**	**100.00**	**100.00**	

10 References

(1) Liu, Y.; Pan, Q.; Hu, X.; Guo, Y.; Chen, Q.-Y.; Liu, C. *Org. Lett.* **2021**, *23*, 3975−3980.

(2) Wang, B.-C.; Hu, F.; Bai, J.; Xiong, F.-Y.; Chen, P.; Li, J.; Tan, Y.; Guo, Y.-L.; Xiao, W.-J.; Lu, L.-Q. *Angew. Chem.Int. Ed.* **2024**, *63*, e202319728.

(3) Ding, M.; Zhang, Z.-X.; Davies, T. Q.; Willis, M. C. *Org. Lett.* **2022**, *24*, 1711−1715.

(4) Meng, J.; He, H.; Liu, Q.; Xu, H.; Huang, H.; Ni, S.-F.; Li, Z. *Angew. Chem. Int. Ed.* **2024**, *63*, e202315092.

(5) Whyte, A.; Bajohr, J.; Arora, R.; Torelli, A.; Lautens, M. *Angew. Chem. Int. Ed.* **2021**, *60*, 20231–20236.

(6) Guo, S.; Chen, J.; Yi, M.; Dong, L.; Lin, A.; Yao, H. *Org. Chem. Front.* **2021**, *8*, 1783–1788.

(7) Luo, W.; Chen, Q.; Mo, X.; Jiang, J.; Xie, P. *Chem. Commun.*, **2020**, *56*, 11953−11956.

(8) Yu, L.-Z.; Wei, Y.; Shi, M. *Chem. Commun.* **2017**, *53*, 8980–8983.

(9) Ishida, T.; Kikuchi, S.; Tsubo, T.; Yamada, T. *Org. Lett.* **2013**, *15*, 848–851.

(10) Harutyunyan, S. R.; Lopez, F.; Browne, W. R.; Correa, A.; Pena, Di.; Badorrey, R. Meetsma, A.; Minnaard, A. J. Feringa, B. L. *J. Am. Chem. Soc.* **2006**, *128*, 9103–9118.

(11) Gaussian 09, Revision E.01, Frisch, M. J.; Trucks, G. W.; Schlegel, H. B.; Scuseria, G. E.; Robb, M. A.; Cheeseman, J. R.; Scalmani, G.; Barone, V.; Mennucci, B.; Petersson, G. A.; Nakatsuji, H.; Caricato, M.; Li, X.; Hratchian, H. P.; Izmaylov, A. F.; Bloino, J.; Zheng, G.; Sonnenberg, J. L.; Hada, M.; Ehara, M.; Toyota, K.; Fukuda, R.; Hasegawa, J.; Ishida, M.; Nakajima, T.; Honda, Y.; Kitao, O.; Nakai, H.; Vreven, T.; Montgomery, J. A., Jr.; Peralta, J. E.; Ogliaro, F.; Bearpark, M.; Heyd, J. J.; Brothers, E.; Kudin, K. N.; Staroverov, V. N.; Keith, T.; Kobayashi, R.; Normand, J.; Raghavachari, K.; Rendell, A.; Burant, J. C.; Iyengar, S. S.; Tomasi,

J.; Cossi, M.; Rega, N.; Millam, J. M.; Klene, M.; Knox, J. E.; Cross, J. B.; Bakken, V.; Adamo, C.; Jaramillo, J.; Gomperts, R.; Stratmann, R. E.; Yazyev, O.; Austin, A. J.; Cammi, R.; Pomelli, C.; Ochterski, J. W.; Martin, R. L.; Morokuma, K.; Zakrzewski, V. G.; Voth, G. A.; Salvador, P.; Dannenberg, J. J.; Dapprich, S.; Daniels, A. D.; Farkas, O.; Foresman, J. B.; Ortiz, J. V.; Cioslowski, J.; Fox, D. J. Gaussian, Inc., Wallingford CT, **2013**.

(12) Lee, C.; Yang, W.; Parr, R. G. *Phys. Rev. B* **1988**, *37*, 785–789.

(13) Becke, A. D. *J. Chem. Phys.* **1993**, *98*, 5648–5652.

(14) Grimme, S.; Antony, J.; Ehrlich, S.; Krieg, H. *J. Chem. Phys.* **2010**, *132*, 154104.

(15) Grimme, S.; Ehrlich, S.; Goerigk, L. *J. Comput. Chem.* **2011**, *32*, 1456–1465.

(16) Dolg, M.; Wedig, U.; Stoll, H.; Preuss, H. *J. Chem. Phys.* **1987**, *86*, 866–872.

(17) Nicklass, A.; Dolg, M.; Stoll, H.; Preuss, H. *J. Chem. Phys.* **1995**, *102*, 8942–8952.

(18) Ditchfield, R.; Hehre, W. J.; Pople, J. A. *J. Chem. Phys.* **1971**, *54*, 724–728.

(19) Hehre, W. J.; Ditchfield, R.; Pople, J. A. *J. Chem. Phys.* **1972**, *56*, 2257–2261.

(20) Hariharan, P. C.; Pople, J. A. *Theor. Chem. Acc.* **1973**, *28*, 213–233.

(21) Tomasi, J.; Persico, M. *Chem. Rev.* **1994**, *94*, 2027–2094.

(22) Cances, E.; Mennucci, B.; Tomasi, J. *J. Chem. Phy.* **1997**, *107*, 3032–3041.

(23) Zhao, Y.; Truhlar, D. G. *Theor. Chem. Acc.* **2008**, *120*, 215–241.

(24) Clark, T.; Chandrasekhar, J.; Spitznagel, G. W.; Von, P.; Schleyer, R. *J. Comput. Chem.* **1983**, *4*, 294–301.

(25) Krishnan, R.; Binkley, J. S.; Seeger, R.; Pople, J. A. *J. Chem. Phys.* **1980**, *72*, 650–654.

(26) Legault, C. Y.; CYL View, version 1.0 b; Universite de Sherbrooke, Sherbrooke, Quebec, Canada, **2009**; http://www.cylview.org.
