# Supplementary material for: Copper-Catalyzed Asymmetric Cyclizative Sulfinamidation: Forging Indole-Based Stereogenic Sulfur(IV) Centers and Atropisomeric Chirality
Source: ACS Cent Sci. 2025 Aug 1;11(9):1762–72. doi: 10.1021/acscentsci.5c00909 (PMC12464752; doi:10.1021/acscentsci.5c00909)
Supplement: Supplementary file 3 [file oc5c00909_si_003.pdf]

oc-2025-00909j.R1

Name: Peer Review Information for "Copper-Catalyzed Asymmetric Cyclizative Sulfinamidation: Forging Indole-Based Stereogenic Sulfur(IV) Centers and Atropisomeric Chirality"

First Round of Reviewer Comments

Reviewer: 1

Comments to the Author

In this manuscript, Shi et al. report a copper-catalyzed asymmetric cyclizative sulfinamidation that produces indole-based stereogenic sulfur(IV) centers. The reaction between ortho-alkynylanilines and TrNSO proceeds efficiently in the presence of a copper/Josiphos catalyst system and LiOt-Bu in 1,3-dioxolane at 40 °C, affording indole-based sulfinamides with excellent enantioselectivities. This methodology demonstrates a broad substrate scope of ortho-alkynylanilines without compromising yields or enantioselectivities. Furthermore, when sterically hindered ortho-alkynylanilines were employed, atropisomeric chirality was induced with excellent selectivities. Mechanistic investigations, including control experiments and DFT calculations, support a pathway involving cyclization to a Cu–indole intermediate, followed by sulfinamidation. The mechanism underlying enantioselectivity is clearly described.

The reaction displays a broad substrate scope and high enantioselectivities for both the stereogenic sulfur(IV) center and atropisomeric chirality. The mechanistic study effectively clarifies the reaction pathway and enantio-determining step. While enantioselective synthesis of sulfinamides using TrNSO and atroposelective indole construction have been previously reported, the current strategy—which involves indole formation followed by sulfinamidation—offers a valuable and practical approach. However, the conceptual novelty does not reach the level expected for publication in ACS Central Science. Thus, I do not recommend publication in ACS Central Science. That said, I believe the manuscript is suitable for publication in JACS after addressing the following minor points:

(1) It would be helpful if the authors could comment on whether they attempted the synthesis of other heterocycles, such as benzofurans or benzothiophenes, in addition to indoles.

(2) In Table 2, it would be informative to discuss whether the enantioselectivity decreases when smaller alkyl substituents (compared to cyclohexyl) are used on the alkyne.

(3) On page 6, right column, line 3, the sentence “This result excludes the possibility that indole is formed first in the reaction system, indicating that indole formation and subsequent sulfinamidation occur in a concerted manner.” should be reconsidered. Since the reaction proceeds via formation of an indolyl–Cu intermediate followed by sulfinamidation, the phrase “indole formation and subsequent sulfinamidation occur in a concerted manner” appears contradictory.

Reviewer: 2

Comments to the Author

The author performed a highly innovative copper-catalyzed asymmetric nucleophilic cyclization and sulfinamidation strategy for the simultaneous construction of stereogenic sulfur(IV) centers and atropisomeric chirality in indole-based scaffolds. The work addresses a significant gap in the synthesis of chiral sulfur-containing pharmacophores and demonstrates exceptional synthetic utility, mechanistic depth, and broad substrate scope. The originality is suitable for ACS Central Science Therefore, this referee recommends publication of this manuscript in ACS Central Science after some revision.

1.Page 3, Table 2: were simple alkyl groups (e.g., methyl, ethyl) investigated as R^2 substituents in this catalytic system? If so, what stereoselectivity trends were observed?

2.According to the control experiment, no reaction occurred when pre-formed indole 72 was subjected to the reaction conditions (Scheme 2c). However, the DFT calculations still modeled the axial chirality of the indole ring being constructed before sulfur chirality

establishment. Could the catalytic cycle alternatively proceed with sulfur chirality forming prior to axial chirality generation?

3.Page 8, Refence 2(j), Page 9, Refence 8(c): the format of the horizontal lines between the page numbers is incorrect.; Page 9, Refence 4(j): the format of the horizontal line between ‘N–N’ is incorrect.; Page 9, Refence 10(f): replacing ‘Wang,F.’ with ‘Wang, F.’ to comply with standardized spacing conventions.; Page 10, Refence 19(b): the literature reference preceding this text is missing its (b) sub-entry and replacing ‘Yang, G.-f.’ with ‘Yang, G.-F.’ to comply with standard author name capitalization conventions.

4.In the Supporting Information, Page 357, Reference 3(a): replacing ‘20231 –20236’ with ‘20231–20236’ to comply with standardized spacing conventions.; Reference 4, Reference 5, Reference 7: the format of the horizontal lines between the page numbers is incorrect.; Page 358, Refence 12(b), there is an extra "v" at the beginning of the sentence.; Reference 15: replacing ‘Truhlar, D. G.’ with ‘Truhlar, D. G.’ to comply with standardized spacing conventions.; Reference 16(b): replacing ‘Binkley,J. S.’ with ‘Binkley, J. S.’ to comply with standardized spacing conventions.

5.In the Supporting Information, Page 11, Compound 48a: replacing ‘IR (ATR):3331’ with ‘IR (ATR): 3331’ to comply with standardized spacing conventions.; Page 20, compound 7b: IR data is incorrect; Page 31, Compound 29b: IR data should be complied with standardized spacing conventions.; Page 34, Compound 36b: there is a missing comma in the HPLC data.; Page 35, Compound 39b: replacing ‘3days’ with ‘3 days’ to comply with standardized spacing conventions.; Page 36, Compound 40b: the punctuation of the IR data is incorrect.; Page 41, Compound 49b: IR data is incorrect.

6.In the Supporting Information, Page 9, Compound 43a: the 13C NMR data exhibits inconsistent decimal precision. This formatting inconsistency persists in subsequent compounds' NMR data. Please standardize all 13C NMR values to uniform decimal places throughout the document and verify all entries. In summary, there are numerous formatting errors in the Supporting Information. Please conduct a thorough check and make the necessary corrections.

Reviewer: 3

Comments to the Author

In the provided manuscript by Shi and co-workers, a Cu-catalyzed 5-endo-dig cyclization followed by sulfinyl trapping is described to prepare indoles bearing S-chiral sulfinamides

in high enantioselectivities. Depending on the substrates used, some reactions also provide access to products bearing atropisomeric axes with good diastereocontrol and in high ee's. Several examples of conversion reactions of the sulfinamide groups are also presented along with detailed mechanistic investigations by DFT calculation. While it's not necessarily clear if the molecules produced actually have any value, the work overall is well done and provides complex products in high ee's that would be difficult to access other ways. The Supporting Information is very thorough. I would recommend acceptance for publication in ACS Central Science. The only small revision I would request is that 1,3-dioxolane is not a typical solvent one would use. The authors must have tried many other solvents and investigated other reaction parameters than what is given in Table 1 of the manuscript. I would recommend the authors add the additional reaction screening data including other solvents tried to the Supporting Information. This information would be valuable to readers wanting to use the chemistry.

Author's Response to Peer Review Comments:

Formatting Needs:

Author Affiliations: Please move author affiliations to the first page of the manuscript under the author list. Please use separate labels for each affiliation next to the authors' names.

Response: We added it.

Author List: Please include the email address(es) of the corresponding author(s) on the first page of the manuscript.

Response: OK.

Graphics: If a figure has parts labeled (i.e. a, b, etc.), each part must be mentioned in the figure caption.

Response: OK.

References: Number references individually, with only one citation per reference. Do not group references.

Response: OK.

Supporting Information: Please add a "Supporting Information" heading to the title page of your Supporting Information file.

Response: OK.

Supporting Information: Please number all pages in the following format: S1, S2, S3, etc.

Response: OK.

Synopsis: ACS Central Science requires a brief synopsis. The synopsis should be no more than 200 characters (including spaces) and should reasonably correlate with the Table of Contents (TOC) graphic. The synopsis is intended to explain the importance of the article to a broader readership across the sciences. Please place your synopsis in the manuscript file after the TOC graphic and label as “Synopsis.”

Response: We have added the synopsis in the manuscript file after the TOC graphic and label as “Synopsis.”

Reviewer: 1

Recommendation: Publish elsewhere JACS

Comments:

In this manuscript, Shi et al. report a copper-catalyzed asymmetric cyclizative sulfinamidation that produces indole-based stereogenic sulfur(IV) centers. The reaction between ortho-alkynylanilines and TrNSO proceeds efficiently in the presence of a copper/Josiphos catalyst system and LiOt-Bu in 1,3-dioxolane at 40 °C, affording indole-based sulfinamides with excellent enantioselectivities. This methodology demonstrates a broad substrate scope of ortho-alkynylanilines without compromising yields or enantioselectivities. Furthermore, when sterically hindered ortho-alkynylanilines were employed, atropisomeric chirality was induced with excellent selectivities. Mechanistic investigations, including control experiments and DFT calculations, support a pathway involving cyclization to a Cu–indole intermediate, followed by sulfinamidation. The mechanism underlying enantioselectivity is clearly described.

The reaction displays a broad substrate scope and high enantioselectivities for both the stereogenic sulfur(IV) center and atropisomeric chirality. The mechanistic study effectively clarifies the reaction pathway and enantio-determining step. While enantioselective synthesis of sulfinamides using TrNSO and atroposelective indole construction have been previously reported, the current strategy—which involves indole formation followed by sulfinamidation—offers a valuable and practical approach. However, the conceptual novelty does not reach the level expected for publication in ACS Central Science. Thus, I do

not recommend publication in ACS Central Science. That said, I believe the manuscript is suitable for publication in JACS after addressing the following minor points:

Response: We sincerely appreciate the reviewer's insightful comments and constructive suggestions. We have carefully revised the manuscript to address the points raised, aiming to enhance its overall quality and impact. We believe these revisions have significantly improved the manuscript, making it suitable for publication in ACS Central Science. We are also grateful for the reviewer's suggestion regarding JACS and have ensured the revised manuscript meets the high standards expected for both journals.

(1) It would be helpful if the authors could comment on whether they attempted the synthesis of other heterocycles, such as benzofurans or benzothiophenes, in addition to indoles.

Response: We appreciate the constructive suggestion. During the revision, we conducted preliminary attempts to synthesize benzofurans and benzothiophenes under the standard reaction conditions. However, these heterocycles proved to be unreactive and failed to yield the desired products. We have incorporated this discussion into the main text and included the corresponding examples in Table S1 of the Supporting Information for further reference.

(2) In Table 2, it would be informative to discuss whether the enantioselectivity decreases when smaller alkyl substituents (compared to cyclohexyl) are used on the alkyne.

Response: Thank you for your valuable suggestion. During the revision, our investigation revealed that the use of smaller alkyl substituents on the alkyne, compared to cyclohexyl, significantly impacts enantioselectivity. Specifically, indoles with methyl, phenethyl, isopropyl, and cyclopropyl groups at C2 position demonstrated a notable decrease in enantioselectivity. This trend underscores the critical role of steric effects in controlling enantioselectivity in this transformation. We have incorporated this discussion into the main text and included the corresponding examples in Table S1 of the Supporting Information for further reference.

(3) On page 6, right column, line 3, the sentence “This result excludes the possibility that indole is formed first in the reaction system, indicating that indole formation and subsequent sulfinamidation occur in a concerted manner.” should be reconsidered. Since the reaction proceeds via formation of an indolyl–Cu intermediate followed by sulfinamidation, the phrase “indole formation and subsequent sulfinamidation occur in a concerted manner” appears contradictory.

Response: Thanks for your constructive suggestion. we have changed the related description. This outcome suggests that the reaction does not proceed through the

generation of free indole species. Instead, it indicates that the formation of the indolyl–Cu intermediate and the subsequent sulfinamidation step occur in a concerted manner.

Reviewer: 2

Recommendation: Publish in ACS Central Science after minor revisions noted.

Comments:

The author performed a highly innovative copper-catalyzed asymmetric nucleophilic cyclization and sulfinamidation strategy for the simultaneous construction of stereogenic sulfur(IV) centers and atropisomeric chirality in indole-based scaffolds. The work addresses a significant gap in the synthesis of chiral sulfur-containing pharmacophores and demonstrates exceptional synthetic utility, mechanistic depth, and broad substrate scope. The originality is suitable for ACS Central Science Therefore, this referee recommends publication of this manuscript in ACS Central Science after some revision.

Thanks for such positive evaluation.

1.Page 3, Table 2: were simple alkyl groups (e.g., methyl, ethyl) investigated as R^2 substituents in this catalytic system? If so, what stereoselectivity trends were observed?

Response: Thank you for your valuable suggestion. During the revision, our investigation revealed that the use of smaller alkyl substituents on the alkyne, compared to cyclohexyl, significantly impacts enantioselectivity. Specifically, indoles with methyl, phenethyl, isopropyl, and cyclopropyl groups at C2 position demonstrated a notable decrease in enantioselectivity. This trend underscores the critical role of steric effects in controlling enantioselectivity in this transformation. We have incorporated this discussion into the main text and included the corresponding examples in Table S1 of the Supporting Information for further reference.

2.According to the control experiment, no reaction occurred when pre-formed indole

72 was subjected to the reaction conditions (Scheme 2c). However, the DFT calculations still modeled the axial chirality of the indole ring being constructed before sulfur chirality establishment. Could the catalytic cycle alternatively proceed with sulfur chirality forming prior to axial chirality generation?

Response: Thank you for your feedback. I apologize for any confusion caused by the previous statement. The experiments in Scheme 2c clearly show that the reaction does not involve free indole species but rather proceeds through a concerted mechanism involving the formation of the indolyl–Cu intermediate and subsequent sulfinamidation. This aligns

with our computational results, which indicate that axial chirality is established before sulfur chirality.

3.Page 8, Refence 2(j), Page 9, Refence 8(c): the format of the horizontal lines between the page numbers is incorrect.; Page 9, Refence 4(j): the format of the horizontal line between ‘N–N’ is incorrect.; Page 9, Refence 10(f): replacing ‘Wang,F.’ with ‘Wang, F.’ to comply with standardized spacing conventions.; Page 10, Refence 19(b): the literature reference preceding this text is missing its (b) sub-entry and replacing ‘Yang, G.-f.’ with ‘Yang, G.-F.’ to comply with standard author name capitalization conventions.

Response: We modified them.

4.In the Supporting Information, Page 357, Reference 3(a): replacing ‘20231 –20236’ with ‘20231–20236’ to comply with standardized spacing conventions.; Reference 4, Reference 5, Reference 7: the format of the horizontal lines between the page numbers is incorrect.; Page 358, Refence 12(b), there is an extra "v" at the beginning of the sentence.; Reference 15: replacing ‘Truhlar, D. G.’ with ‘Truhlar, D. G.’ to comply with standardized spacing conventions.; Reference 16(b): replacing ‘Binkley,J. S.’ with ‘Binkley, J. S.’ to comply with standardized spacing conventions.

Response: We modified them.

5.In the Supporting Information, Page 11, Compound 48a: replacing ‘IR (ATR):3331’ with ‘IR (ATR): 3331’ to comply with standardized spacing conventions.; Page 20, compound 7b: IR data is incorrect; Page 31, Compound 29b: IR data should be complied with standardized spacing conventions.; Page 34, Compound 36b: there is a missing comma in the HPLC data.; Page 35, Compound 39b: replacing ‘3days’ with ‘3 days’ to comply with standardized spacing conventions.; Page 36, Compound 40b: the punctuation of the IR data is incorrect.; Page 41, Compound 49b: IR data is incorrect.

Response: We modified them.

6.In the Supporting Information, Page 9, Compound 43a: the 13C NMR data exhibits inconsistent decimal precision. This formatting inconsistency persists in subsequent compounds' NMR data. Please standardize all 13C NMR values to uniform decimal places throughout the document and verify all entries. In summary, there are numerous formatting errors in the Supporting Information. Please conduct a thorough check and make the necessary corrections.

Response: Thank you for your careful inspection. We have further corrected the corresponding errors to make the supporting information more rigorous.

Reviewer: 3

Recommendation: Publish in ACS Central Science after minor revisions noted.

Comments:

In the provided manuscript by Shi and co-workers, a Cu-catalyzed 5-endo-dig cyclization followed by sulfinyl trapping is described to prepare indoles bearing S-chiral sulfinamides in high enantioselectivities. Depending on the substrates used, some reactions also provide access to products bearing atropisomeric axes with good diastereocontrol and in high ee's. Several examples of conversion reactions of the sulfinamide groups are also presented along with detailed mechanistic investigations by DFT calculation. While it's not necessarily clear if the molecules produced actually have any value, the work overall is well done and provides complex products in high ee's that would be difficult to access other ways. The Supporting Information is very thorough. I would recommend acceptance for publication in ACS Central Science. The only small revision I would request is that 1,3-dioxolane is not a typical solvent one would use. The authors must have tried many other solvents and investigated other reaction parameters than what is given in Table 1 of the manuscript. I would recommend the authors add the additional reaction screening data including other solvents tried to the Supporting Information. This information would be valuable to readers wanting to use the chemistry.

Response: Thanks for such positive evaluation. We have added detailed optimization of the reaction conditions in the supporting information, including solvents, ligands, Cu catalysts and bases (see Table S2-S5 in supporting information).

oc-2025-00909j.R2

Name: Peer Review Information for "Copper-Catalyzed Asymmetric Cyclizative Sulfinamidation: Forging Indole-Based Stereogenic Sulfur(IV) Centers and Atropisomeric Chirality"

Second Round of Reviewer Comments

Reviewer: 1

Comments to the Author

In this revision, the authors have appropriately addressed the previous concerns, including the examination of other heterocycles, the effect of small substituents, and clarification regarding the reaction mechanism. Although favorable results were not obtained for the heterocycle scope or small substituents, these findings nonetheless provide valuable information for readers. The authors have also responded appropriately to the requests for revisions in the Supporting Information. Taken together, I believe that the manuscript is suitable for publication in ACS Central Science.

Reviewer: 3

Comments to the Author

The new draft has adequately addressed my previous review's requested revisions.

Reviewer: 2

Comments to the Author

This manuscript presents a groundbreaking copper-catalyzed asymmetric cyclizative sulfinamidation strategy for the synthesis of indole-based sulfinamides with simultaneous control of stereogenic sulfur(IV) centers and atropisomeric chirality. This work is innovative and well-organized. Notably, one of the HPLC data in the supporting information lack of accuracy: compound 42b, there are four peaks in the spectra of the chiral products; Hence, this manuscript is recommended to be published on ACS Central Science after the mentioned issue is corrected.

Author's Response to Peer Review Comments:

To Reviewer: 2

This manuscript presents a groundbreaking copper-catalyzed asymmetric cyclizative sulfinamidation strategy for the synthesis of indole-based sulfinamides with simultaneous

control of stereogenic sulfur(IV) centers and atropisomeric chirality. This work is innovative and well-organized. Notably, one of the HPLC data in the supporting information lack of accuracy: compound 42b, there are four peaks in the spectra of the chiral products; Hence, this manuscript is recommended to be published on ACS Central Science after the mentioned issue is corrected.

Response: According to your suggestion, we have provided an accurate HPLC graph of 42b in the supporting information.
